# Detection and characterization of a novel bat-borne coronavirus in Singapore using multiple molecular approaches

**DOI:** 10.1099/jgv.0.001307

**Published:** 2019-08-16

**Authors:** Xiao Fang Lim, Chengfa Benjamin Lee, Sarah Marie Pascoe, Choon Beng How, Sharon Chan, Jun Hao Tan, Xinglou Yang, Peng Zhou, Zhengli Shi, October M. Sessions, Lin-Fa Wang, Lee Ching Ng, Danielle E. Anderson, Grace Yap

**Affiliations:** ^1^ Programme in Emerging Infectious Diseases, Duke-NUS Medical School, Singapore; ^2^ Environmental Health Institute, National Environment Agency, Singapore; ^3^ Sungei Buloh Wetlands Reserve National Parks Board, Singapore; ^4^ Wuhan Institute of Virology, Chinese Academy of Sciences, Wuhan, PR China; ^5^ Saw Swee Hock School of Public Health, National University of Singapore, Singapore; ^6^ Department of Pharmacy, National University of Singapore, Singapore

**Keywords:** bats, *Betacoronavirus*, capture enrichment, Singapore

## Abstract

Bats are important reservoirs and vectors in the transmission of emerging infectious diseases. Many highly pathogenic viruses such as SARS-CoV and rabies-related lyssaviruses have crossed species barriers to infect humans and other animals. In this study we monitored the major roost sites of bats in Singapore, and performed surveillance for zoonotic pathogens in these bats. Screening of guano samples collected during the survey uncovered a bat coronavirus (*Betacoronavirus*) in *Cynopterus brachyotis*, commonly known as the lesser dog-faced fruit bat. Using a capture-enrichment sequencing platform, the full-length genome of the bat CoV was sequenced and found to be closely related to the bat coronavirus HKU9 species found in Leschenault’s rousette discovered in the Guangdong and Yunnan provinces.

## Introduction

Infectious diseases continue to be a major threat to modern society and coronaviruses (CoVs) are one of the most notable virus families responsible for recent, highly pathogenic viral disease outbreaks. Over the last two decades, major outbreaks of deadly CoVs have been reported in humans and livestock, including severe acute respiratory syndrome (SARS) in 2003 [1, 2], Middle East respiratory syndrome (MERS) in 2012 [3] and most recently, swine acute diarrhoea syndrome (SADS) in 2017 [4]. These outbreaks have had a significant impact on the economy, global travel and society, and should serve as a warning for other emergent CoVs. While Asia used to be considered to be the ‘hot zone’ for such outbreaks, the emergence of MERS highlighted that such events can happen anywhere in the world.

CoVs are a family of viruses within the order *Nidovirales*, and the subfamily *Orthocoronavirinae* can be divided into four genera, *A* *lpha* *coronavirus*, *B* *eta* *coronavirus*, *D* *elta* *coronavirus* and *G* *amma* *coronavirus*, based on antigenic reactivity and/or genome sequences (https://talk.ictvonline.org/taxonomy/). CoVs in general can cause disease in a variety of domestic and wild animals, as well as in humans, whereas alpha- and betacoronaviruses predominantly infect mammals. SARS-CoV and MERS-CoV belong to the genus *B* *etacoronavirus*, under the subgenera *S* *arbecovirus* and *M* *erbecovirus*, respectively [5]. These viruses are genetically distinct [6]. SARS-CoV originated in China and spread worldwide, infecting approximately 8000 individuals with a overall mortality rate of 10 % during the 2002–2003 epidemic [7]. Since emerging in 2012 in the Middle East, MERS-CoV has spread to 27 countries and has infected humans with an estimated mortality rate of 35 % [8].

In addition to SARS-CoV and MERS-CoV, the betacoronavirus human coronavirus OC43 [9], and the alphacoronaviruses NL63 and 229E-CoV have been reported to cause infections in humans [10, 11]. The novel HKU2-related bat coronavirus, SADS-CoV, was identified as the aetiological agent responsible for a large-scale outbreak of fatal disease in China that caused the death of 24 693 piglets [4]. Although SADS-CoV was shown to be incapable of infecting humans in its current form, it remains to be seen whether further adaptation or a related virus can spill over into human populations in the future.

Bats have been identified as a natural reservoir for a variety of viral diseases, with RNA viruses accounting for the overwhelming majority of emerging pathogens [12]. Notably, bats have been recognized to carry exceptionally diverse CoVs from which SARS-CoV, MERS-CoV and SADS-CoV are thought to originate [4, 13].

In light of these recent events, it is probable that bat-borne CoVs will continue their zoonotic emergence and cause more human SARS- or MERS-like outbreaks. One of the ongoing challenges that scientists face with these zoonotic crossover events is ensuring early warning or rapid detection and diagnosis so that effective countermeasures can be implemented. Unfortunately, current detection methodologies and our lack of understanding of cross-species transmission make the prediction of future spillover events nearly impossible. Active surveillance remains our best defence to ameliorate the impact of these events. With limited funding available for these surveillance systems in regions where they are likely to be needed the most, it is currently impossible to achieve sufficient breadth and depth to comprehensively detect all potential spillover events. An investment focused on surveillance of CoVs in bats is considered to be most impactful based on past data and the current trend of emerging infectious diseases worldwide [14].

In 2013 the National Environment Agency (NEA) and the National Parks Board (NParks) initiated a bat surveillance programme in Singapore. Through an island-wide survey, bat species, bat day roosts, population counts and age demographics were determined at each location. Guano samples were collected from three major roost sites and other opportunistic sampling, such as harvesting of dead bat carcasses and oral and rectal swabs from live catches, was performed. While screening these samples for pathogens of public health concern in Singapore, CoVs were detected in two guano samples and a rectal swab collected from a lesser dog-faced fruit bat.

The CoV genome is the largest among all known RNA viruses (~30 kb) and whole-genome sequencing is not a simple task, especially from clinical or surveillance samples with limited genetic material [15]. To increase the speed and success rate of full-genome sequencing of novel CoVs, we have developed a probe-based enrichment method to specifically capture CoV sequences using a library of 120 nt probes. The library contains probes targeting all known CoV sequences [published by the National Center for Biotechnology Information (NCBI) and unpublished]. This new platform was first validated with cultured MERS-CoV and subsequently applied to sequence and characterize the novel CoV detected from bat samples collected in Singapore.

## Methods

### Ecological data gathering and guano sampling

Roost surveys were performed for the identification of species, determination of the size of roosts and guano collection. Twenty-seven locations were selected for survey based on reports of bat activity collected from social media reports and public sightings from January to March 2014 as well as bat sightings from field surveys of major green areas. As bats are known to utilize alternative roost sites for different functions, only day roosts were considered for the ground survey to reduce the possibility of double counting [16]. Information such as bat species, population counts and age estimates were collected from the day roosts. Photographs of the roosting bats were taken to assist and complement survey counts. Individual bats were identified by their eyes, head, nose and wings using a taxonomic key [17]. For estimation of the age of bats, it was assumed that nursing bats were juvenile and all others were adults.

Repeated surveys were performed at five roost sites [HortPark, Singapore Zoological Garden, Singapore Botanic Garden, Rifle Range Flyover (RRFO) and Lower Peirce Reservoir] and three major roost sites were also identified (East Coast Park, RRFO and Singapore Zoological Garden) for periodic guano sampling. During sampling, groundsheets were placed under the roosting bats and guano samples were collected from the groundsheets and put into universal transport media.

In addition, opportunistic rectal swabs were collected from bats that were accidentally caught in mist nets during the NParks bird-ringing programme or provided by the Wildlife Reserve Singapore (WRS). Any bat carcasses found in a nature reserve (SBWR) or donated by pest control companies were sent to the laboratory for testing. Guano samples and rectal swabs were placed in universal transport media and bat carcasses were kept refrigerated before processing in the laboratory. Bat carcasses were swabbed in the oral and rectal cavities before dissection. Tissues (heart, liver, spleen, lung, kidney, intestine and brain) were then harvested.

The geographical distribution of bat roosts in Singapore was plotted in QGIS 3.2.3 (QGIS Development Team, 2018) using the basemap of Singapore downloaded from Onemap (Singapore Land Authority, 2018). Project coordinate system WGS 84 projection (EPSG:4326) was used for plotting.

### RNA extraction and PCR screening of bat samples

A total of 107 guano samples, 14 rectal swabs and 6 bat carcasses, collected over a period of 3 years, were screened for the presence of CoVs [2], Japanese encephalitis virus (JEV) [18], rabies-related lyssavirus [19], leptospirosis [20] and hantavirus [21]. Nucleic acid was extracted from tissue/guano/swabs using the AllPrep DNA/RNA Mini kit (Qiagen) according to the manufacturer’s instructions. RNaseOUT (Invitrogen), a ribonuclease inhibitor, was added to each RNA prior to storage at −80°C. RNA was reverse-transcribed using SuperScript Reverse Transcriptase (Invitrogen) according to the manufacturer’s instructions. The cDNA was used as the template for conventional PCR for the detection of coronavirus, hantavirus and leptospirosis with Phusion Flash High-Fidelity PCR Master Mix (Life Technology). Any PCR products were purified using a PCR purification kit (Qiagen) and sequenced by Sanger sequencing. RNA was used as a template for real-time PCR using the QuaniTect SYBR green kit (Qiagen) according to the manufacturer’s instructions for the detection of rabies-related lyssavirus and JEV.

The primer sequences for the detection of coronavirus, JEV [18], rabies-related lyssavirus [19], leptospirosis [20] and hantavirus [21] are listed in Table S2 (available in the online version of this article).

### Cells and viruses

Vero B4 cells (RRID: CVCL_1912) were maintained in Dulbecco's Modified Eagle Medium (DMEM) supplemented with 10 % foetal bovine serum (FBS) at 37°C and 5 % CO_2_. MERS-CoV (accession number: NC_019843) was propagated and amplified in Vero B4 cells in DMEM supplemented with 5 % FBS at 37°C and 5 % CO_2._ PaKi cells [22] were maintained in DMEM supplemented with 10 % FBS at 37°C and 5 % CO_2_. WIV1 (accession number: KF367457.1) was propagated and amplified in Vero cells in DMEM supplemented with 5 % FBS at 37 °C and 5 % CO_2_.

### RNA extraction

Vero B4 cells were infected with MERS-CoV under BSL3 containment and harvested for RNA extraction when cytopathic effect (CPE) was evident. RNA samples were verified as being inactive according to the Duke-NUS ABSL3 protocol prior to removal from the facility. RNA was extracted from both MERS-CoV-infected Vero B4 cells and uninfected Vero B4 cells using the Total RNA Kit I (Omega Biotek) according to the manufacturer’s instructions. RNA was extracted from WIV1-infected Vero cells using the Total RNA Kit I (Omega Biotek) according to the manufacturer’s instructions. The total amount of RNA was quantified using a Nanodrop (Thermo Scientific).

### Design and assessment of CoV probe library

For the solution-based targeted-enrichment methodology, biotinylated DNA probes were designed against 90 coronavirus genomes from the NCBI database and our unpublished studies that are known to infect bats. The probes were 120 nucleotides (nt) in length and complementary to the viral genome. To capture this diversity, we took an iterative approach to the design, where we first designed baits targeting the conserved regions of the genome and then generated baits targeting the variable regions. The full list of 4303 probes designed for CoV can be found in Table S1.

To assess the CoV probe library, next-generation sequencing (NGS) libraries were prepared from 1 µg of PaKi cellular RNA spiked with serially diluted MERS-CoV RNA. A volume of 2 µl of the diluted MERS-CoV RNA was added to cellular RNA of a bat cell line (PaKi) and used as the template for the library preparation. Each of the samples used for library preparation was assigned a unique index barcode and sequenced and the remaining library was subjected to enrichment using the CoV probes before sequencing. Both the enriched and unenriched libraries were sequenced on the Illuminia MiSeq (read length of 2×250 bp) and analysed.

### Preparation of Illumina DNA libraries from viral RNA

Illumina libraries were constructed from total RNA using the NEBNext Ultra Directional RNA Library Prep Kit for Illumina (New England Biolabs) in conjunction with NEBNext Multiplex Oligos for Illumina (New England Biolabs) according to the manufacturer’s instructions with minor modifications. Briefly, 5 µl of total RNA was added to first-strand synthesis buffer and random primers before incubation at 94°C for 2 min in order to generate RNA fragments larger than 500 nt. Following first-strand and second-strand cDNA synthesis, double-stranded cDNA was purified using Mag-Bind RxnPure Plus beads (Omega Bio-Tek) and eluted in 60 µl nuclease-free water. In order to obtain a library size between 400–600 nt, size selection of the libraries was performed using Mag-Bind RxnPure Plus beads (Omega Bio-Tek) in a two-step selection by adding 35 µl and then subsequently 15 µl of beads to the reaction. The library was eluted in 20 µl nuclease-free water and amplified by PCR. Libraries were purified using the MinElute PCR Purification kit (Qiagen), eluted in 25 µl nuclease-free H_2_O, visualized on a 1.5 % agarose gel and quantified using a Bioanalyzer High Sensitivity DNA Assay (Agilent).

### Targeted enrichment of viral library

Targeted viral enrichment was achieved using a custom-designed library of 4303 biotinylated, 120mer xGen Lockdown probes (Integrated DNA Technologies). Prior to the capture of viral sequences, 1 µl each of xGen universal blocking oligo TS-p5 and TS-p7 (Integrated DNA Technologies), matched accordingly to the library index, was added to 20 µl of library DNA and 0.5 µl of 5 µg Cot-1 DNA (Invitrogen) to block the binding of baits to non-viral regions of the library fragments. Blocked libraries were ethanol-precipitated and resuspended in 2.5 µl H_2_O, 3 µl NimbleGen hybridization solution and 7.5 µl NimbleGen 2X hybridization buffer (Roche). Following a 10 min incubation at room temperature, resuspended libraries were denatured at 95 °C for 10 min and cooled on ice before the addition of the CoV probe pool. A total amount of 3 pmol of probes was added and hybridized to the libraries for 4 h at 65°C. To capture virus-specific library fragments, 100 µl magnetic M-270 streptavidin Dynabeads (Life Technologies) was added to the hybridization reaction and the mix was incubated for a further 45 min at 65°C, with shaking at 2000 r.p.m. in a ThermoMixer C (Eppendorf). Streptavidin beads were washed to remove unbound DNA using a SeqCap EZ hybridization and wash kit (Roche) according to the manufacturer’s instructions. A post-capture PCR amplification of 20 cycles with P1 and P2 primers (Illumina) was performed and the enriched library was purified using the MinElute PCR Purification kit (Qiagen). The purified, enriched library was eluted in 25 µl nuclease-free H_2_O, visualized on a 1.5 % agarose gel and quantified using a Bioanalyzer High Sensitivity DNA Assay (Agilent).

### NGS sequencing analysis

The prepared libraries were sequenced on a MiSeq machine. The resulting reads were then subjected to processing in the VIPR4 pipeline (https://github.com/nf-core/vipr/). Briefly, adapters were trimmed and quality-assessed by Skewer [23] and FASTQC (https://www.bioinformatics.babraham.ac.uk/projects/fastqc/), respectively. Host reads were then removed by the decont.py script (https://github.com/CSB5/decont/blob/master/decont.py). The remaining reads were then assembled into contigs using the BBTools Tadpole module (https://jgi.doe.gov/data-and-tools/bbtools/). The contigs were then polished by incorporating unassembled viral reads through an iterative process until no further viral reads were incorporated (https://github.com/nf-core/vipr/). A final mapping against the assembly was performed with BWA [24] and low-frequency variants were then identified by LoFreq [25]. Coverage and variant allele frequency graphs were produced by Bedtools (https://bedtools.readthedocs.io/en/latest/) and ViPR Tools (https://github.com/nf-core/vipr/).

### Phylogenetic analysis

Multiple sequence alignment of the full-genome sequence of BtCoV92 coronavirus and 2211 unique coronavirus sequences present in the NCBI database was carried out using a fast Fourier transformation method in MAFFT v6.940b. An approximately maximum-likelihood phylogenetic tree was generated using the generalized time-reversible model of nucleotide evolution in FastTree v2.1.7 [26]. FastTree uses SH-like local supports with 1000 resamples to estimate and validate the reliability of each split in the tree. From the tree, the branch containing the sequence from BtCoV92 coronavirus sequence was selected and a more robust maximum-likelihood phylogenetic tree was created using RAXML [27] with 1000 bootstrap replications. The trees were visualized using FigTree v1.4.2 (http://tree.bio.ed.ac.uk/ software/figtree/).

The complete N protein sequence from BtCoV92 and the partial N sequence from BtCoV22/29 were aligned with the representative coronavirus sequences using BioEdit version 7.0.5.3. The appropriate phylogenetic model was selected using ProtTest3.4.2. A maximum-likelihood phylogenetic tree based on the N protein was constructed with the LG+F amino acid model with 1000 bootstrap replicates on mega 7.

## Results

### Active surveillance of the Singapore bat population

**Fig. 1.**
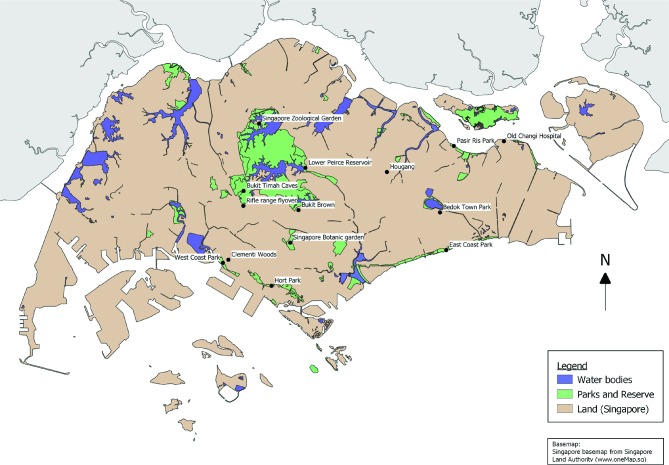
Geographical distribution of bat roosts in Singapore. Locations surveyed for the presence of bats include Bedok Town Park, Bukit Brown, Bukit Timah Caves, Clementi Woods, East Coast Park, Hort Park, Hougang, Lower Peirce Reservoir, Old Changi Hospital, Pasir Ris Park, Rifle Range Flyover, Singapore Botanic Garden, Singapore Zoological Garden and West Coast Park. Singapore basemaps available from the Singapore Land Authority at www.oneMap.sg.

From 2013–2016, an island-wide survey was conducted by NParks and NEA to determine the species, demographics and population size of bats at different locations in Singapore. A total of 27 locations were surveyed and bat roosts were found in 15 of these locations (Fig. 1). During the collection of bat ecological data (Table 1), three major bat roosting sites at East Coast Park, RRFO and Singapore Zoological Garden were identified for surveillance and periodic guano sampling.

**Table 1.** Surveillance of bats in Singapore

Location	Date of survey	Bat species	Adults	Juveniles	Total count
HortPark	12 December 2013	Lesser dog face	20	1	21
	13 January 2015	Lesser dog face	29	0	29
	16 December 2013	Whiskered bat	1	0	1
*Singapore Zoological Garden	21 February 2014	Lesser dog face	154	50	204
	19 November 2014	Lesser dog face	181	12	193
*Singapore Botanic Garden	13 March 2014	Lesser dog face	21	1	22
	28 April 2014	Lesser dog face	10	1	11
*Rifle Range Flyover	25 March 2014	Cave nectar bat			4117
	20 November 2014	Cave nectar bat			3767
*Lower Peirce Reservoir	04 April 2014	Lesser dog face	22	3	25
	16 January 2015	Lesser dog face	30	0	30
Bukit Brown	09 April 2014	Lesser dog face	5	0	5
Clementi Woods	27 May 2014	Lesser dog face	17	6	23
Bukit Timah Caves	11 July 2014	Dusky fruit bat			
Hougang Block	14 August 2014	Lesser dog face	16	0	16
Hougang (Reality Park)	14 August 2014	Lesser dog face	11	1	12
Bedok Town Park	21 October 2014	Lesser dog face	13	1	14
East Coast Park	6 November 2014	Lesser dog face			92
Pasir Ris Park	19 November 2014	Lesser dog face	21	2	23
Old Changi Hospital	14 January 2015	Lesser dog face	27	0	27
West Coast Park	28 January 2015	Lesser dog face	27	0	27

*Denotes locations with repeated visit.

Thirteen out of 15 roost sites only harbour lesser dog-faced bats (*Cynopterus brachyotis*). Cave nectar bats (*Eonycteris spelaea*) were found at RRFO and whiskered bats (*Myotis* sp.) at HortPark. The species and population of bats in the Bukit Timah Caves were undetermined due to insufficient light to allow for counting and visual inspection of the bats without disturbance. Based on a previous survey, the bats were identified as dusky fruit bats (*Penthetor lucasi*) [28, 29]. We were unable to distinguish the number of adult and juveniles bats found at the Rifle Range Flyover and East Coast Park, as the bats were roosting under the flyover and on tall trees and our field equipment was unable to capture the information accurately.

Based on the two visits to RRFO in 2014, this location had the highest population count of cave nectar bats roosting under a bridge (3767 on the first visit and 4117 on the second visit). Among all of the sites, Singapore Zoological Garden (24.5 %, 50/204) and Clementi Woods (26 %, 6/23) had the highest percentage of juveniles, which may be indicative of a maternity roost. Ninety bats were estimated to roost on the trees along the coast at East Coast Park. Other locations had moderate population of bats (fewer than 30). Five locations were revisited after 10–11 months, and the population observed was consistent, with the exception of that at the Singapore Botanic Gardens, which showed a 50 % reduction.

One hundred and seven bat guano samples and 14 rectal swabs collected from 2013 to 2016 were screened for the presence of zoonotic pathogens. Represented in the cohort were lesser dog-faced bats (44.3 %, *C. brachyotis*), cave nectar bats (23.6 %, *E. spelaea*), dusky fruit bats (17.1 %, *P. lucasi*), naked bull dog bats (0.7 %, *Cheiromeles torquatus*), Malayan flying foxes (10 %, *Pteropus vampyrus*) and undetermined bat species (0.18 %).

### Identification of a CoV-positive sample through active surveillance

A total of 107 guano samples, 14 rectal swabs and 6 bat carcasses were collected and screened for the presence of CoVs, JEV, rabies-related lyssavirus, leptospirosis and hantavirus. Three samples collected from lesser dog-faced bats (*C. brachyotis*) were found to be positive for CoV by PCR targeting a 440 bp region located within the RNA-dependent RNA polymerase gene (RdRp) [2]. This set of primer targets motif A corresponding to MGWDYPKCD and motif C corresponding to MMILSDD within the RdRp gene, which is strictly conserved among known RNA-dependent polymerases [30–33]. CoV sequences (BtCoV22 and BtCoV29) were detected in guano samples collected from the Singapore Zoological Garden and SBWR, respectively. One CoV sequence (BtCoV92) was detected in the rectal swab of a bat carcass collected from SBWR. Apart from the CoV sequences, no other viral sequences were detected in any other sample. Based on the phylogenetic analysis on the nucleotide sequence of the 440 bp RdRp PCR fragment, the three BtCoVs clustered with the species *Rousettus bat coronavirus HKU9* in the genus *Betacoronavirus*. BtCoV92 shared 96 % sequence similarity with BtCoV22 and BtCoV29, which were 100 % identical with each other. They also shared 91–95 % sequence identity with coronavirus detected in *Cynopterus* spp. and *Rousettus* spp. found in Asian countries such as Malaysia, Thailand, PR China and Cambodia.

### Design of CoV probe library for enrichment

A probe library to cover all known alpha- and betacoronaviruses was designed and constructed from all available non-redundant genome sequences (full length or partial). The algorithm BaitMaker [34] was then used to design a custom probe set for coronaviruses (Table S1). Briefly, BaitMaker utilizes a k-mer-based search pattern strategy to identify regions of conservation and variability within a viral species. Non-redundant, non-overlapping probes are then designed to target these regions with a minimum of 85 % identity between the probe and the targeted sequence. The source code for BaitMaker software is freely available at: https://umasangumathi.github.io/BaitMaker/.

### Assessment of CoV probe library with MERS-CoV

Generic NGS enables unbiased pathogen discovery, but suffers from issues of low sensitivity and high per-sample cost, mainly due to high background of host genes in most samples. After NGS, removal of host background followed by a *de novo* assembly strategy is usually used for pathogen discovery when viral RNA is abundant. However, this approach fails when the amount of viral RNA is low.

To overcome this issue and to fully realize the power of NGS in pathogen discovery and investigation, several groups (including ours) have now developed an enrichment-based NGS strategy [35–37]. To improve the signal-to-background noise ratio, NGS libraries are incubated with a set of biotinylated viral capture probes predesigned according to known viral sequences, followed by enrichment with streptavidin-coated beads. From the assessment of the CoV probe library, there were at least 100 times the number of reads mapped in the enriched samples as compared to the unenriched samples for each dilution of MERS-CoV RNA (Table 2). The enrichment strategy was also evaluated on WIV1, an uncharacterized SARS-related coronavirus. An increase in the depth of coverage in the enriched sample compared with the unenriched sample was demonstrated (Fig. S1).

**Table 2.** Validation of CoV probe library with MERS CoV RNA. Comparison of the number of reads generated and mapped to the reference sequence (accession number: NC019843.3) before and after enrichment. The generated libraries were spiked with 1000 ng of PaKi cellular RNA

PaKi cellular RNA	MERS-CoV RNA	Unenriched NGS libraries	Enriched NGS libraries	
Quantity (ng)	Dilution	Quantity (ng)	Total reads	Reads mapped	% mapped	Total reads	Reads mapped	% mapped	Fold enrichment
1000	Undiluted	2	462775	202	0.044	1354075	134148	9.9	225
1000	1 : 10	0.2	473779	37	0.008	1689629	24 224	1.4	175
1000	1 : 100	0.02	461147	18	0.004	1790036	10 448	0.6	150
1000	1 : 1000	0.002	450053	23	0.005	1684569	9053	0.5	100
1000	1 : 10 000	0.0002	359827	18	0.005	1669207	10 324	0.6	120

### Genome characterization of the novel bat CoV

Sequences obtained from the three BtCoVs detected in lesser dog-faced bats were analysed and assembled with Geneious (version 9.1.8). Sanger sequencing was used to fill in the gaps after NGS. Due to sample quality and availability, all remaining RNA extracted from BtCoV 22 and BtCoV29 was pooled for sequencing. Prior to pooling, all individual sequences from BtCoV22 and BtCoV29 were analysed and they were 100 % homologous; henceforth we will refer to this sample as BtCoV22/29. From this pool, 47.4 % (13 983/29 512 bp) of the genome sequence of BtCoV22/29 was obtained through a combination of NGS (18.3 %) and Sanger sequencing (31.7 %) with overlapping regions (GenBank accession number MN187553). BtCoV22/29 was 98.2 % similar to BtCoV92. The full-length genome for BtCoV92 was obtained with 69.1 and 50.1 % of the genome covered by NGS and Sanger sequencing, respectively, with overlapping regions (GenBank accession number MK492263).

The CoV detected in the rectal swab from *C. brachyotis* is the first reported full-genome sequence of bat CoV found in Singapore. The genome of BtCoV92 comprises 29 569 nucleotides (excluding the polyadenylated tail at the 3′ terminus) with a G/C content of 38.55 %. A putative transcription regulatory sequence (TRS) motif, 5′- ACGAAC-3′ is observed preceding each open reading frame (ORF) except for gene E with the TRS motif, 5′- TCGAAC- 3′ (Table 3). The 5′- ACGAAC-3′ TRS has also been shown to be the TRS for SARS-CoV [38].

**Table 3.** Coding potential and putative transcriptional regulatory sequences of BtCoV92

Bt92-CoV ORF	Nucleotide positions (start–end)	No. of nucleotides	Predicted size (aa) of protein	Nucleotide position in genome	TRS sequence	Distance (nt) from TRS to ATG
ORF1a	227–20823	20 598	6866	70–75	TTGA**ACGAAC**TAAAA	151
Spike	20777–24601	3825	1275	20729–20734	TTGA**ACGAAC**TAGT	41
NS3	24598–25290	693	231	24589–24594	ATAA**ACGAAC**AGAA	3
E	25290–25520	231	77	25280–25285	GCAG**TCGAAC**TATA	8
M	25523–26188	666	222	24595–25500	TTGA**ACGAAC**AAGA	22
NP	26243–27634	1392	464	26232–26237	TTGA**ACGAAC**CAAT	5
NS7a	27670–28272	603	201	27663–27668	TTGA**ACGAAC**CAA	1
NS7b	28296–28889	594	198	28290–28295	TTGA**ACGAAC**ATGA	0
NS7c	28931–29326	396	132	28873–28878	GGTT**ACGAAC**ATCT	52

The genome of BtCoV92 is similar to that of the other CoVs, with a characteristic gene order of 5′-replicase ORF1ab, spike (S), envelope (E), membrane (M) and nucleocapsid (N)-3′.

**Fig. 2.**
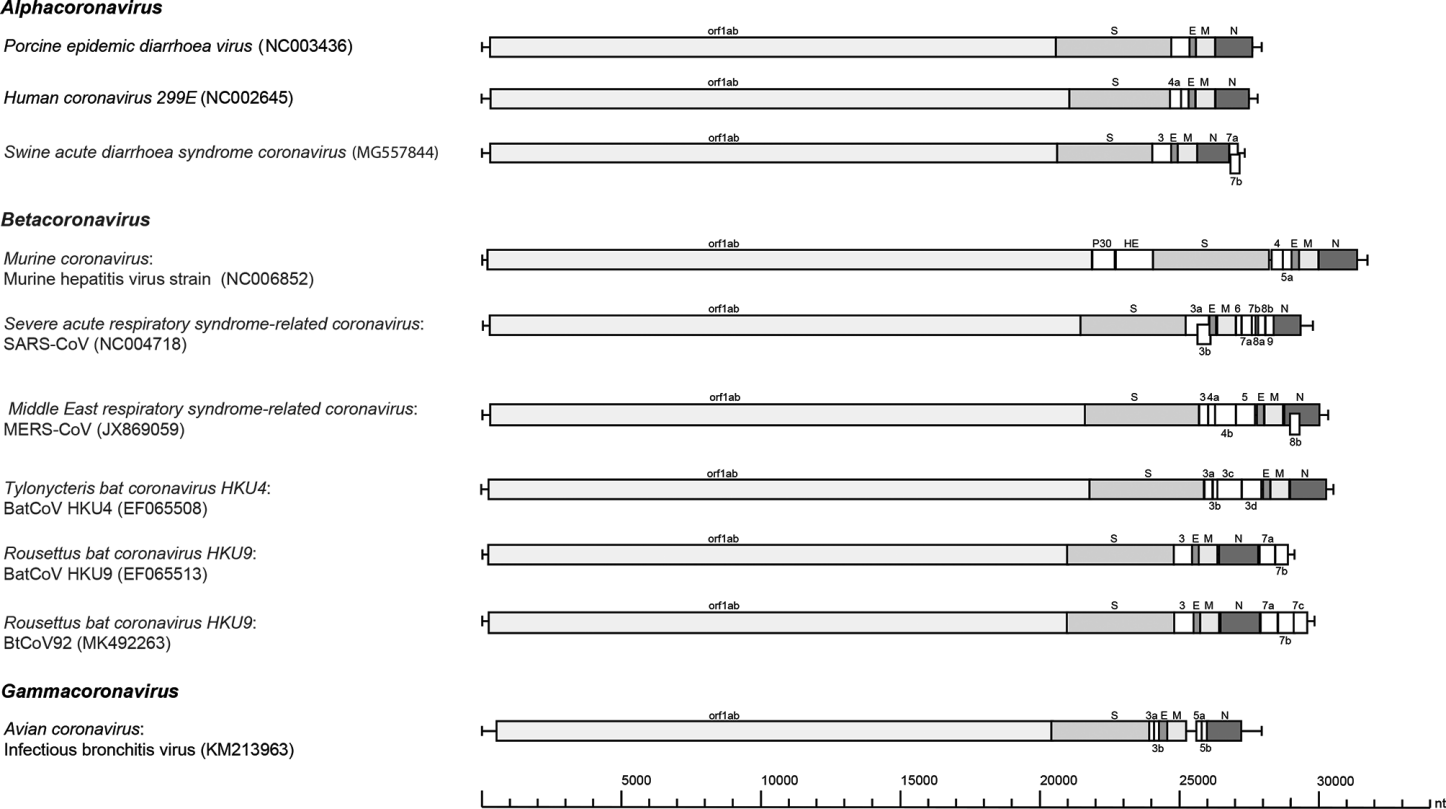
Genome analysis of BtCov92. Genome organization of BtCoV92 (in bold) and a representative coronavirus from *Alphacoronavirus* (porcine epidemic diarrhoea virus, human CoV-299E and swine acute diarrhoea syndrome coronavirus), *Betacoronavirus* (murine coronavirus, severe acute respiratory syndrome-related coronavirus, Middle East respiratory syndrome-related coronavirus, Tylonycteris bat coronavirus HKU4 and Rousettus bat coronavirus HKU9) and *Gammacoronavirus* (avian coronavirus). ORF 1ab (orf1ab), the gene for haemagglutinin esterase (HE), the spike protein (S), the envelope protein (E), the membrane protein (M) and the nucleocapsid protein (N) are represented by grey boxes. The genes for nonstructural proteins are represented by white boxes.

**Fig. 3.**
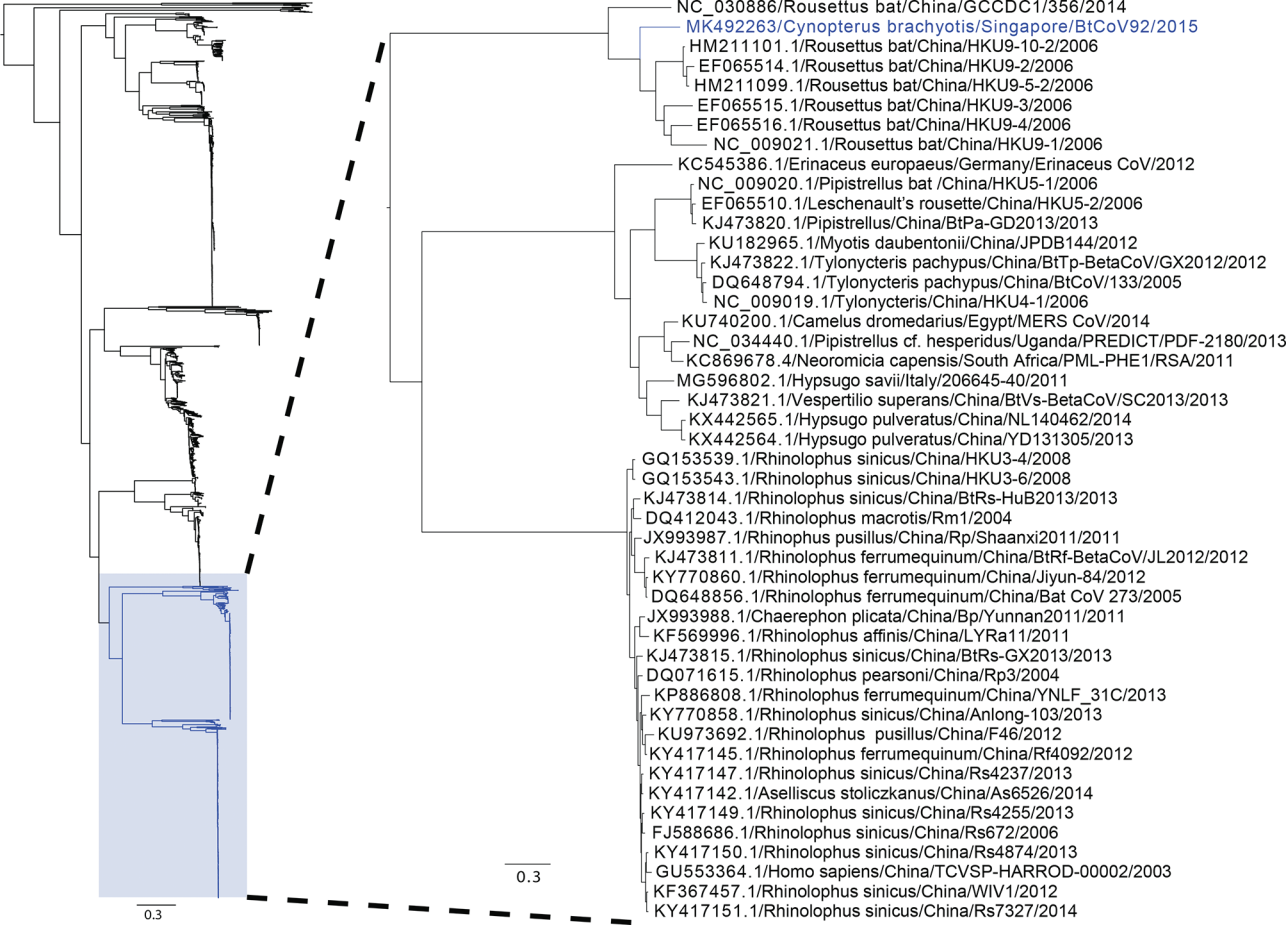
Phylogenetic tree of the full-genome sequences of coronavirus. Multiple sequence alignment of the BtCoV92 coronavirus sequence and 2211 sequences present in NCBI database was carried out using a fast Fourier transformation method in MAFFT v6.940b. An approximately maximum-likelihood phylogenetic tree was generated using the generalized time-reversible model of nucleotide evolution in FastTree v2.1.7. FastTree uses SH-like local supports with 1000 resamples to estimate and validate the reliability of each split in the tree. From the tree, the branch containing the sequence from the Bt92 coronavirus sequence and 58 sequences from NCBI was selected and a more robust maximum-likelihood phylogenetic tree was created using RAXML with 1000 bootstrap replications. The trees were visualized using FigTree v1.4.2.

**Fig. 4.**
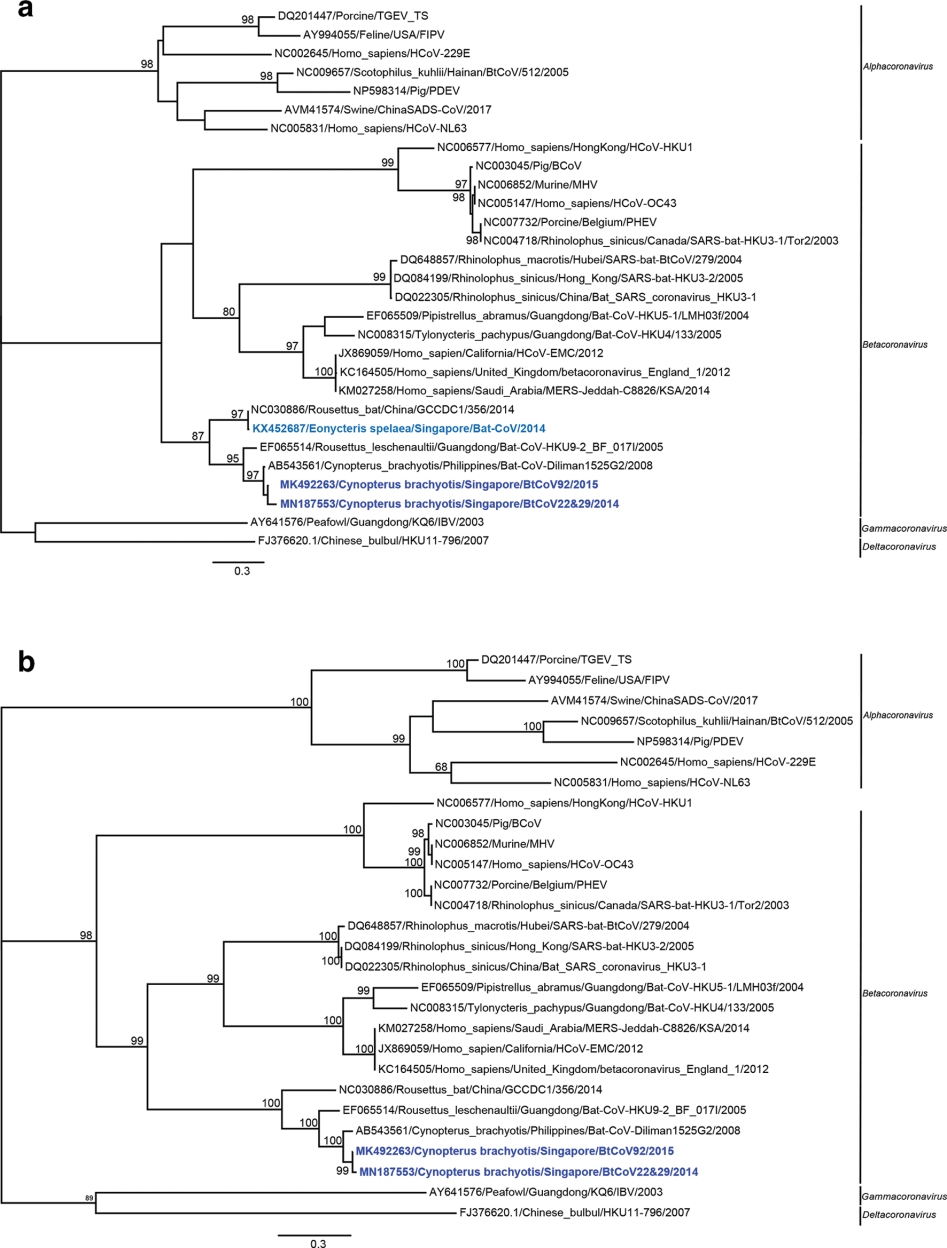
Phylogenetic tree of amino acids sequences encoding the coronavirus nucleocapsid protein (N). A partial N protein sequence of BtCoV22/29 and the complete N protein sequence of BtCoV92 were compared to the representative coronavirus species based on (a) the available partial N protein sequence of a betacoronavirus (KX452687) previously detected in *E. spelaea* and (b) full N protein sequences. Maximum-likelihood trees were constructed using the LG+F amino acid model (best-fit model determined by mega7) with 1000 bootstrap replications. Only bootstrap values >80 % are shown.

The BtCoV92 contains four accessory genes interspersed within the structural genes, one ORF encoding none structural protein NS3 between the S and E genes (ORF 3) and three ORFs encoding NS7a, NS7b, NS7c downstream of the N gene (Fig. 2). NS7c is not typically reported in HKU9, but was only found in the diliman-1525G2 virus found in *C. brachyotis* in the Philippines [39]. Based on the available partial genome sequence, NS7c was also present in BtCoV22/29, sharing 100 % sequence identity with the NS7c found in BtCoV92. BtCoV92 is closely related to the species *Rousettus bat coronavirus* HKU9 found in Leschenault’s rousette and an unidentified species *Rousettus* sp. found previously in the Guangdong and Yunnan provinces [40] (Fig. 3). Bat coronavirus HKU9 and bat coronavirus GCCDC1 are two closely related yet distinct betacoronaviruses. GCCDC1 is genetically less diverse than HKU9 and has a p10 gene from reovirus in its genome [41]. HKU9 consists of five lineages and BtCoV92 does not group within any of the five lineages, which makes it a new one [41]. The S and N proteins of BtCoV92 showed 61–72 % and 74–80 % identity to those of the HKU9 species, respectively. We found no recombination between BtCoV92 and HKU9 (NC_009021.1 and HM211101.1) with the Simplot program (data not shown). The N protein of BtCoV92 showed 90 % identity with that of the diliman-1525G2 virus and they clustered in the same branch in the N protein phylogenetic analysis (Fig. 4a and b). Similarly, when using a partial N protein sequence (461 amino acids, 8 missing amino acids from position 351–358 of the N protein), BtCoV22/29 clustered together with BtCoV92 and diliman-1525G2 virus during phylogenetic analysis (Fig. 4b). In a previous study, a betacoronavirus (KX452687) was detected in local *E. spelaea* [42] and when comparing the N protein partial sequences with those of BtCoV92, BtCoV22/29, these sequences clustered under a different branch from KX452687 in the phylogenetic tree (Fig. 4a).

## Discussion

In 2015 [39], emerging diseases with epidemic potential were identified by the World Health Organization (WHO) as targets for outbreak preparedness, and SARS-CoV and MERS-CoV were both in the list [43]. Delayed identification and detection of SARS-CoV during the outbreak from November 2002 to April 2003 resulted in wide spread of disease and increased human fatalities [44] . Therefore, surveillance remains key for early outbreak alerts and disease interventions.

In this study, we reported the characterization of the complete genome sequence of a novel coronavirus in Singapore from lesser dog faced bats (*C. brachyotis*). These bats were the most frequently counted bats during the island-wide surveillance and are commonly found in parks, where their food sources (fruit trees) are widely distributed. They are well adapted to living closely with humans, which inevitably increases the risk of disease transmission and the emergence of zoonoses [45]. Previously, flaviviruses (Phnom Penh virus, Carey island virus and Jugra virus), Nipah virus and betacoronaviruses have been found in *C. brachyotis* [39, 46, 47] . Obtaining the full-genome sequences for CoVs from field samples has always been challenging for multiple reasons: (1) the CoV genome (26–32 kb) is the largest non-segmented RNA viral genome known to date [15]; (2) CoV genomes are genetically highly variable due to the high frequency of recombination among CoVs [48] [48, 49]; and (3) the absolute amount and relative percentage of viral nucleic acid is generally very low in clinical/field samples. In the current study, we developed a CoV-targeted probe-based enrichment strategy to improve the efficiency of CoV genome characterization. As a proof of concept, we applied this CoV-enrichment NGS strategy to rapidly characterize the first complete CoV genome sequence in Singapore from a novel CoV detected in *C. brachyotis*. Genetically, this newly found CoV belongs to the genus *Betacoronavirus* and is closely related to the species *Rousettus bat coronavirus HKU9* [50] and diliman-1525G2 [39].

As for any probe-based enrichment NGS strategy, our probe library is designed to detect both known CoVs and novel CoVs genetically related to known members of the family. We believe that the same approach is equally applicable to both outbreak responses and active surveillance programmes, such as the US PREDICT project [51] and the proposed Global Virome Project [52].

## Supplementary Data

Supplementary File 1
