# Supplementary material for: Detection and characterization of a novel bat-borne coronavirus in Singapore using multiple molecular approaches
Source: J Gen Virol. 2019 Aug 16;100(10):1363–74. doi: 10.1099/jgv.0.001307 (PMC7079695; doi:10.1099/jgv.0.001307)
Supplement: Supplementary File 1 [file jgv-100-1363-s001.pdf]

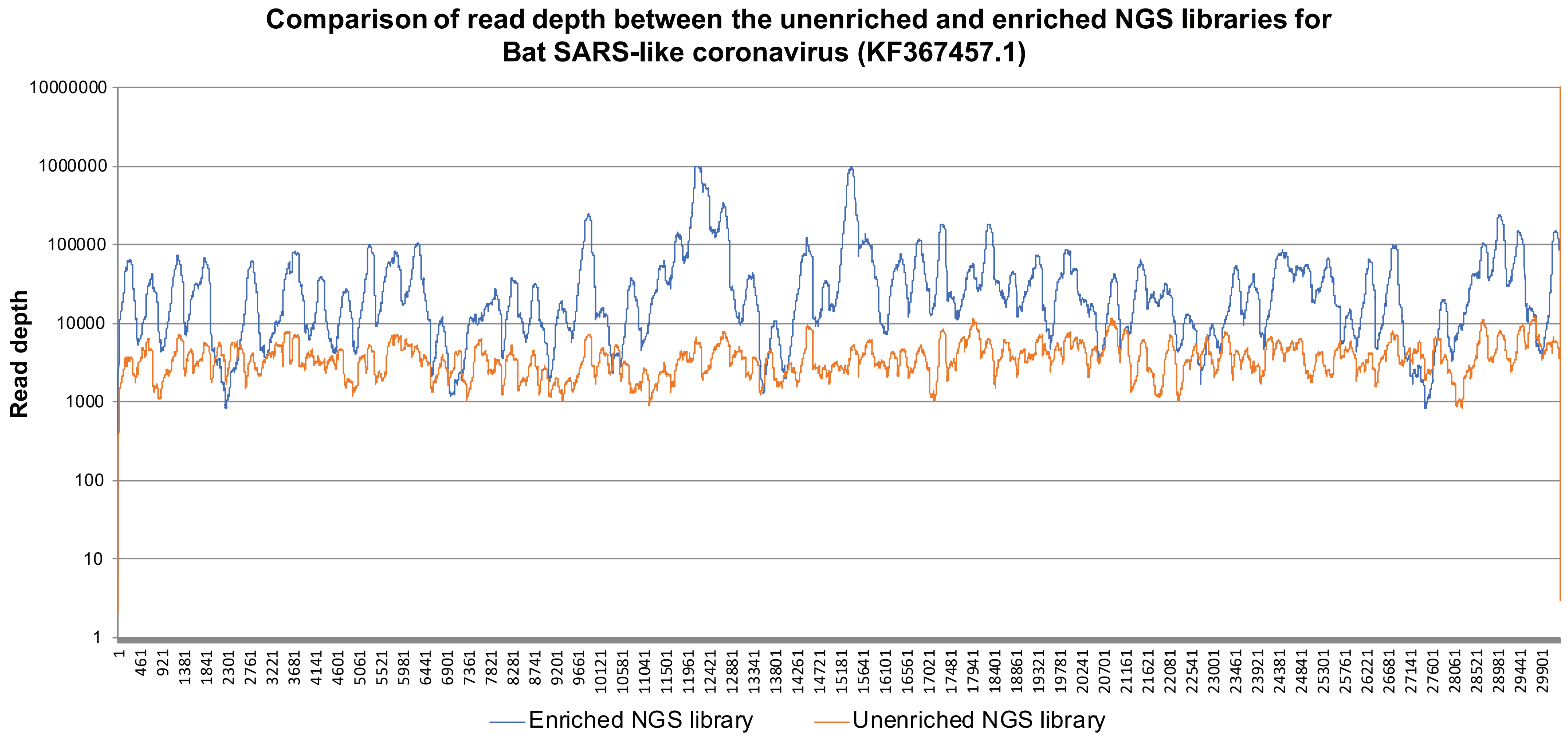

Comparison of read depth between the unenriched and enriched NGS libraries for
Bat SARS-like coronavirus (KF367457.1)
Read depth
10000000
1000000
100000
10000
1000
100
10
1
Enriched NGS library
Unenriched NGS library

Supplementary table 1: Probes designed against 90 coronavirus genomes.

	Bait sequences
**CoV-bait-1**	AGTCGTTCTGCCATCTCTGATGCCCTCTTCCACCATCTTGTCATCACCAAAGTTTCTGGGCTTGTTTTTAGTACGAGCTCCAAAAACAGAATCAACATCTTTTCCTGGAGCCAGAACACG
**CoV-bait-2**	TTGACAACAAAATCCACACCATTCGAAAACCCGATCTGGAGTGGCACATTAGTTCCAACATTAGAGCCGCAGACGTGAGCACCTTCGACGTCCATACCAAGCCAGCCTCTGACATGTCGC
**CoV-bait-3**	TTGAGAGTGGGTATGCATCAATAGCAAGTGACACATAACGCTCTAACAAAACAACAGAATCTGTCTTTGCGACGTCATCAACAAAGACACCAGCTGACAATATTCTGCTAGGATCTGGGT
**CoV-bait-4**	AAAGATTGCATCACCTATGCGTGAGACACAAGATTTAGCACGAGGAACAAAGTACATAGTACCATCACAACAACCCATGTAAGCATAAACAACGCCACCAAAGGTTAAAGTGAAAGACTT
**CoV-bait-5**	TGTGAGTGCAGATTGAGAAAAAAACCCAAATGATGAATAAACACTGGTCGGACTTGAATATGGCACCAAAACACAGATCGTGCCAAGTTTGTATGAGCGATAAATTGTGTGTGGGGTTTG
**CoV-bait-6**	GTGGATGTAGATTTATAAATAACAGTGTCGGAATCGTAGTCTGCCTTTTCAAAAGTAAACTCGCCGACCTGATACTTAGAATCTTTTGAAATCTGAAAACACGTGAAAACAGAGTTCCTG
**CoV-bait-7**	CAATACAAGCTGCAGGTCCAGCAGGACTCATAAGATTGTTAATATCTATAGCGCCTAGAAGAGGCGTTAACGAGTACATCAGTAGACACACTAAGTACTTCATATTCCGAAAAGATAGTC
**CoV-bait-8**	TAAAGAGTTGCTACTAATATTAGCCAAAACAGTAGTATGAACTAATTCAAAGAATAATTCCCAATTACCCTCTGAGTAGGCTGCCAGCGTATTAGAAGAGGCATAAATAACAAAACCTAA
**CoV-bait-9**	AGATATTATAATCAACACACCTATCTAAAACCACATTATTTACAAAATTAGCAGTGACAGTAGGTACTGTTTCAGAAGTAAATATACGACTGCCTGGGCTATGTTTAATAAACACAAGCA
**CoV-bait-10**	CCTTACCTTCTCCAACTATGCCATCTCCTTCTGCCTTAATGGAGCGCTGCTTCAGCTTACCAGGAATAATTTCATTATTCTGGAGCTTGACAATACGCTCACACCCAAGGACCAGGGGCC
**CoV-bait-11**	GCATATGTTATACAACAACTTTTCAGTGTACTGTCAGAATTGTTCATAAATTCTTGGACAGAAAACAAACCCATTTTTGCAAGGCGCACTTGTGATATAAGAAGATGCATACCACCAATG
**CoV-bait-12**	CAATGCATTCTTCACAAAGCCGCACAAACAAATCTTTGTCTGCTATATATGATGGTGCCCTAAATGTGGTTTCTTGTGACACAGTACAAACTTCATCTCTAACCTTAACGTCGTAGAAAC
**CoV-bait-13**	GTCGCTAGAAAAACACTCAGTAATAACAGTGTAATGGCACTACCCTGGGACATAGCCAGAATAAGCCAGTCAAACATTGTTTAATTTCCTGTGTCGAAGATCTCGTTGATAATTTCAACG
**CoV-bait-14**	TAATCTTCATGTACATTAAAAGCAGGGTACAACTTAGAAATGGTGCTGTACTTCTCTTGGTTCACTAAAACAGGTGCCTTGAGGCTACCAACATTATGAGAAGTTAAAACAAACAACATG
**CoV-bait-15**	AACAGAATTTAACTCGTCAAGTACAGCGTCCACAGAGATGCCGATGGACTTGACAGTGTCCATAAATCGTTTAGTTAATGAATGTGAACCTTTTCGATGGGTTCGTAACTTTCAAATTGT
**CoV-bait-16**	ATGGCACCCGACGTATCTAAAATAGCTAACCCACCATCTGCTAAATAACTAAAATTAGCAGCAGAATCAGTCACATTAGTAATAAAACCTTGGCCTACTCTGCCATATATATTATAGGCA
**CoV-bait-17**	ACTCAATTGGTAAATAACTACGGATCATCTCTATGACCTCTGGTGGGTATATGGCCCCCCTAGTTGTTACAGCGGCTGTCCTAGGCTCCACAACCCTACAAACTGCGTAATCGTGATAAA
**CoV-bait-18**	AGATAGCACGACCATCTTTCCAAAGGAAGAAGTCATGAATAGCTAAAGCGCCACTATATTTAAGAATGTCATATATGGACTGCTCGTGTTCCATAACACTCTTAGTGCAGCGTTTTACAA
**CoV-bait-19**	ATAAGTGTTCAACGTCAATTGCATTTCAGTGCGATTTGGAATGGTAGCAGCGAATTCCTTCAAAGTTTTATTAACGTCAATGTAGTCAGGCAAAAGCTCTGGCAACTCATTGATAGTCAA
**CoV-bait-20**	AAGATAAGCATTTACCTGCTGAAGAAAGTAAGGAAGGTTAGGATTAGTTTTAGGTACAGACATTTTGTAGTGATACTTGATTTCAACAGTATCACCAACTTCTTTAGTAGAGACTCTCGA
**CoV-bait-21**	ATCACTGGCAAAGGCCAATGTCACAAGGTTGGACGACATACCTGCTAGTTATCGGTTTCAAGCACGACGGAACGTACTATCCACACAATGGCCAGACGGAACTGAACCAAAGGCAGCCAC
**CoV-bait-22**	ATGTTCAAGCTCGACACTAGCAACTTCAACACGTGAGAAAGTGATAGGATTTTCACCACCACTGAAAGTCTTAACTGTGTTTAGACCTGCTTCAACAGCGACATTGAATGTGTCTACAGC

**CoV-bait-23**	ATTACCATCCTCCATTTTATAGTTAGAATGAGGAACCAAATCAGAATAACGTTTGGAACCCTCAAACAAACCGTCTTTGTAGCAATAAACATGAGTACCACCAAGGCCTTCGAGTTTTGT
**CoV-bait-24**	TGAATTAGACCGAGATTGATTATTACTCTTGCTCTGACTACGAGAGTCAGAGCGTGAAGCAGGTCTGCTGTCAGAGTTAACAACCTCAAGACTGTCAGGCAGTGTAAAGTCAAATTCAGG
**CoV-bait-25**	AGTCACAAAAGCATTAAGTGCAGCAAGACGTCCTGTAATCAATCTGTCAACCTGCTGATCTGCTTCCAAAGTATCAAGCCTATTATACAGATCTTCTATAGAAGAAGAAATGGCTTGAAA
**CoV-bait-26**	CGGAGGTCCTTAGCCATCTGGGTATTGACGTCAAAGCAGAACGGGTCAAGCTTCCGGTAGTGAGCATCCTCATCGTATGTTGAGGATGCGACAAAACCATCAGAACTCTGAACTGCAATG
**CoV-bait-27**	ACCATGTAACGGTATGTGTGTTACTAAGAAAAAGAGACCTGACACTATGTAATTGTAAGCACGCCAATTTACAAATGCACTAGTGTAAGCAAAGAAATATTGTGCTGTAACAACTAACAA
**CoV-bait-28**	ACTGGGTTGTTACCATTAATTTGGACAGCCAAATCATGGTCAGTTTTAAAATTGTCAGAAAGTGCAACATAAGTGGCTGCGTGAGAGGGTGGAAGTAAGTCGTCACCCCTACTACAATCT
**CoV-bait-29**	CAAACTTGCAGTTAGCACCAACAGGACTCAGCGACAAACAGAACTTGTTAAAAGTCAAGTAATTGTTCAATTTATCAAATGAGAAAGGACAAGTACCAGTTTTTATAACAGCTGTAGCCT
**CoV-bait-30**	ACATAATTAATCTGTCCCTGTGAAACATCACATTCACCATCATCATAAAAGAAAAATGGCATGGGCTTAAGCTTAGCAAAGGCACGCTTGTCATATGCAGGTGTGTGGAAGGCATGGTTG
**CoV-bait-31**	GGCAGCAACCCAGGTAGAAGGACGGAATCAATGGAGTCGGTGGGCAGGCACAACCTTCGAAAGTGCCCTTAAAGGAAAGCGCACTACGTTGTTTTACTACACAGCGACTCCTGTCAAACA
**CoV-bait-32**	AACAATTGTGACTGTGCCAGATGGTACAAGATACCTGTCCAATTTATCAAAAACAGCTTCAGCATCACCCGGTTCACCTTTCTTAGCACCAGCAACATGGTACAAAAGTGCAACAAAACC
**CoV-bait-33**	ACCTCTTGACTACTATATTTCTTATGTTTTACATCATAATCATATGAAGTGAATTCATCACCAACATAAAATCTGTAAAATCCATCTGAACATTCAACTTTTGTGACTTCTTGATGAGAT
**CoV-bait-34**	ATATCACGTTCAAACGGACGTAATTTACCATGACGTAAATAACGATATTTATAATTATAATTACCCGTCGACGTCGCATCTATATTACGCGTATTCCACGCTAATACACAACCCATAAAA
**CoV-bait-35**	TAACCCAAGTAATTCTGGAAGTCTCGATGTTATCGTTGGGAGGATATTCACCATATTTGAGATTTCCTCTGGGACCTGTGCCAGTATAGTAGAAGGCATAGGATGGAGGAATTTGAGTAC
**CoV-bait-36**	CGTACGCTGAGTGGTAGATGTAGTTGGGTTCAGAGACAGCCTGCTTACCATAGAATGCGTAGAGGAGTCTCTGTTTGAGATTGAGATGTCCATTAGCTTGCACCGCAGTTGAGGGTGCAA
**CoV-bait-37**	CAAACCACACCTGCATACTGAACATTACCAAGCTGAAAGATACGTTTGAATGATTCAAAATCACTTACGACTATGGTAAGTTTAGTAGCACATGCTGCAGGTATTATAGAAAGTGGTACT
**CoV-bait-38**	TTTACAGCATTCTCAACCTTATTAAGGGCTCGAGAAATAGTAGTCAATGAACTAACTGTTTGTTGAATGGCATTACTAACACCGCTAAAAGCCACAGTAATATTACCCATTGCAGCATTA
**CoV-bait-39**	AATGTGGACTTTCTCAATTTCGTAACGTTGTAAGCGTTACCGCCACAGTCACTACAAGATTTGAGTAAACAGTTGTAACAACCACAACAACCAGTAGCAAGACAACACCATAGCATAAGT
**CoV-bait-40**	CATCAATGTCAAACATCTCACACTTAAGCATGTGAGTCATAGACATTAAAGGCATTAAATAAGAATAGTATGACTCCAAGTCAACACACCCATTACCAGGTTGAGTAACTATAAAGTCAC
**CoV-bait-41**	TGGGTACTAACCTACCTCTCCAGCTAGGATTTTCTACAGGTGTTAACTTAGTAGCTGTACCGACTGGTTATGTTGACACTGAAAATAACACAGAATTCACCAGAGTTAATGCAAAACCTC
**CoV-bait-42**	CCGGCGATTCTAACGTATGATATACTATTTTATCACCACGTTTTAAAAATTCTACACCTAATTCCGTACGTTGACCCGAATACGACCAATCACGATACGAACCCGCTAACGATACCGTTT
**CoV-bait-43**	AGTCTTAGAACCTTGCTTGGCAACCCAGAAAACTCCAACTTGCCGTTCACGGAATTTTAGATCAGCATGAGGTCCAGTGCCGAGGTAATAGAAATGCCAATTAGAAGGCAGATCTTTCCT
**CoV-bait-44**	TCTTCTAATTTTAACGGCGAATCTTGCGAACGTTTCGCTAAACCTATCATTAAATGTAAACCACCTAATTGACCATGCGAAAAATCACCATATACTATATGTTCAAACGCATAACCTTCT

**CoV-bait-45**	CTAAATTTTCAGTGCCACAATGTTCACAAGTGGCTTTCAGAAAGTCGCACGTCTGCCATGAAACTTCATCGCAATGATTACATTTCATCAAGGTAGACAAGTGCATATTGTTACACTCCT
**CoV-bait-46**	GACACGGGAGACAAACAACACTATTTGTTTACCAACCCAAGCTAAATTTGCTGGAACATTTGCCACACGAGCACCTGCTACACCAGCTATTACAGCTATAGTAATGGGACAATCCATGGA
**CoV-bait-47**	ATATCAAGGTTTAGGAAGTCCTCCTCCATAGTACTACGTGGCAAGAAATCTGCAGTAGTACGTCCTTGAGTGAAGTAGCCATCAAACTGATCAACAAACTGACCATTTTTCCGGACATAG
**CoV-bait-48**	CATCAAATGCCAAATTAATGCAAGATGTAACAATCAGAGCAGGTATTAGAAAGACCTGGAAAAACAATGTCTTATGCTTGAGTGTGAACATCAAAAGTGAGGACAGCAATGACAAACACG
**CoV-bait-49**	TGTGGCTTTAACTGGAACTAGGACATGTTGACAGGTGCTAACTAGCTCCCTACAGTTGGGTTGGTCGCCATCTACACAATGATACTGAAAATTAAATTTGAAGGTTAGCGAACTAGCTGC
**CoV-bait-50**	CAGAAGGTTTTGTGTATGGTAACAAAATGCAAATAGTTCCAATTTTATAAGATCTGTAAAAGGTATAGGGCCCATCAGTTGTCAAACCAGAAAACCAAGAATATGAACCAAAGACACACT
**CoV-bait-51**	CAGGTGGTTCCACAGTCCTGCTTGCTGCTACAAGAATGTCATAAATATGACGATCATTGACCACAATGTACACTTGCTTCTCGCAAGTGGCAACACAGGCCTTGTAGCTTTCAACTGGGT
**CoV-bait-52**	TGCCAAGGTAAACTCTTGTATAAGAGCTAGTGGGTCTAATTAAAGTATACGTAGTACCATCGAGCGTCGTCGCGCTCGGTGTTACTGGTGTACTTGTAGTATAAGACACAGTGGCTGGCT
**CoV-bait-53**	CGTCAGACACATAATTGTCTATGTCATTATCTATTATAAGCGTACCTTCTGGAAGCCATTGCTTTAATACTGTAGTACCAGGAGCTACACCTTTATCACTACCTGCACCAAAATGCATAA
**CoV-bait-54**	AAGTCGGCTATTTATGACACTAAGATCATAATTAGTACACATAGAGACCTCATCAACTACAACAATGTCACAGCTTACTTCTGGTAAAGCATTGACAGTACAAAACAAGTACTGCGCATT
**CoV-bait-55**	TAAGAAAAATGGGCCAAGCAATGTTTTCAACGTTTTCAGGTGCAATTTCTTTAGCATGTACAATTTTACCATCATTATCACGAATGTCTATAACATTCCAGATAACGCCGGCATAGTGCA
**CoV-bait-56**	TGCATTGAGGTGTGTAAGGAACAAATCGATACGATCGCGTCAATGCGCATGTCTGCCACTAGTCACACACAGAACCCAGAGGGACAGATGACCGCCATAGGTGAGACGGTAAAACCTATC
**CoV-bait-57**	TACGACCAAAACCACAAATAGTTTTTTCATGGTGTGTTAACGAAGTGGTTACCAAAGTTTAGTAACTTACCATTATTTCTAATGAGCAACTTACCCTTCCTTAGTAATCCAATGACCATT
**CoV-bait-58**	TCAGCAGGTTCAAGCAGAATGGTTTCAGACCAATCAACAATAGCAATGGTACCATCATCTACAACTGGCACATCTGCATCTCTAAAGGATAATGCATCACCTTGTTCAATAACTGAATAG
**CoV-bait-59**	AAAAAGGTGACTATGGTGATGCTGTTGTGTACAGAGGTACTACGACATACAAGTTGAATGTTGGTGATTACTTTGTGTTGACATCTCACACTGTAATGCCACTTAGTGCACCTACTCTAG
**CoV-bait-60**	AGTCTCAGTCCAACATTTTGCCTCAGACATGAACACATTATTTTGATAATAAAGAACTGCCTTAAAGTTCTTAATGCTAGCTACTAAACCTTGAGCCGCATAGTTACTGTTATAGCACAC
**CoV-bait-61**	GTAAATTCCACGATACCGTACCTAATATTTCTTCTTTCGCATTCGGCGCTTCCGACGGTAATACATAAAACGCCGATTTACATTTTTTAACGCCGTTTTCGCTTCTTCTAACGTATAAC
**CoV-bait-62**	TGAAAGAAGTGCATTTACATTGGCTGTAACAGCTTGACAAATGTTAAAGACACTATTAGCATAAGCAGTTGTAGCATCACCGGATGATGTTCCACCTGGTTTAACATATAGTGAGCCGCC
**CoV-bait-63**	ACATTTTGGAAAGTCCCGTTGATTTTGGTGCCAAAACAAACTCCCATTGACGTCAATGGGGTGGAGACTTGGAAATCCCCGTGAGTCAAACCGCTATCCACGCCCATTGATGTACTGCCA
**CoV-bait-64**	AGCCCACGAAGTAGGCGGCGGCGCTGGTGCCCCAGGTGTCCTGGGCGGGGCTGAAGGCGGTCAGGATGGCGCGGAAGTTGGTGATGTTGATGCCCAGGGGCAGCTTGAAGATGGGCTTCA
**CoV-bait-65**	AATCACTTTCGGTAAGTGCAGCAGCTTTACGACGGCGACTCCCATCGGCAATTTCTATGACACCAGATACTCTTCGACCGAACGCCGGTGTCTGTTGACCAGTCAGTAGAAAAGAAGGGA
**CoV-bait-66**	GACATCGTGCCTCGCGGTCACTGAGACACGCGCCATACCTGGTGCAAGCATGCGACCGGGGACATTTCAGAACTATTTCTTACGTAATCAGCTTATTGAAGCTCTGAAGGGTTCGGCCGT

**CoV-bait-67**	TCGCTACAAGAGCGCCCTTGTGTATAAATGGAGTCACTAAATTGCAAAAACTCACCATTGAGTTTTTTATAAATATAAAATTTAACAGGTTGTTTAATGGTTTCACTATCACGAATAATG
**CoV-bait-68**	TCTTTCCATGTACACCAAAATGACCTACATGAAAGTTCTACTGACAAATGTGTAGGTTTTAAATCATCACATTCAAAAAGATGTTTAGCTGTTACAATTGTAAAATCATCTATAGCAAAG
**CoV-bait-69**	ACATCATGTGTAATAACACCTTTGTAGAACATTTTGAAGCATTGAGCTGACTTCTCCTTGTGTGCTTTTAGCTTATTGTCATAAACTAAAGCACTCACAGTGTCAACAATTTCAGCAGGA
**CoV-bait-70**	ATTAATAGAGAGTGAACCAACATAACCCCATTGTTGAATGTCTATGACATATGGATTGTAAACATAGTCACAGCCCAATGCATGTTTAAAACAACAATAATCATTACTAACAGAGTTGTA
**CoV-bait-71**	CACCGCATCTTAGAACTGTTTGAGAGCCACATACTACACAAAGACCAGCAGCTTGTAGTACTGTAGACTTCTCATACATGCTAGCATAAAAGCTTTCATCCCAAAACTTGGACTCCTGTT
**CoV-bait-72**	CAATAAACCAGTGACCAAAGCAGTTGCATTTGCAGGCAAACCAAGTTGCAGTGAACTGATGGAAGTCAAATTGTACCAACCATCAACACAGTACATACCAACAGTAGCATATGTGACATT
**CoV-bait-73**	GACAAATGTAACATTGCAGTCTGCAATACGGGCAAAGTCAGCTATCGTAGGTATGCGTGGCTCAAACATGATGCGAGATGTAATTTTAAAACCAGCATCATCTTTATAAAGAGCCAGATT
**CoV-bait-74**	TTCCTAACCAACCTCTAACATGACGCATGGCAAAATCTCTAGTACAGAACAAACTATGACTGCCAGGCATACTAACGTCAAACCTGAAACCCATATATGAGATCACATGTTCATAAGTGC
**CoV-bait-75**	AATGAGTCGGCTAACAATTGCTGGTTTTGTTGCAGCACATCAGTCTGTAGTGCAACATAATTAAGTCGAGATTGAACAGCTAGTGAGAAGGGTATAGCTGATGCGGCTGTGATACCGCCG
**CoV-bait-76**	GCGCAAGTGCTGGTAAAGATGTAGGGAGTCCTGGCCATTGAAACACGTTAAGCATATCAAATCGCCAGCACAAAATGCATTATAGTCGTAGTGGGTGGTTGTGTTATAGACACACGGCCA
**CoV-bait-77**	AAGCATACGCGCCAGGGACATAGACAGTGTAAACACCATTATCCCAATGAATTCTTACAGTTTCACCGGAGACAGTGAAACCAAGTATACGATTAATTTTGTCGGTGGTTTTAAAATCAA
**CoV-bait-78**	TAATTGACTTCATCAATTATATCAGTCTCCATAGCCACAGCATCATAAATAGGCTCAGGGTTAGATGGCACAATTGGACTAAACTCTGGAGCAGCAGGGTTCATGGGATGAGATTGGGTT
**CoV-bait-79**	AAGGTCTCTGGAACGCTGGCTTTTTCAAGTGAGGTGTAGGAGTAGACCCCATCACTTAGATTAAAGACATTCATGTCACACGCCAACCTGTCTAGTGGGGTGTTGCAATACTTAATTTTC
**CoV-bait-80**	CCATGTGATTGGTACTCATCGTGGGATATGTTAATGTTGATGCGGATATTGTCGGAAAGGCCAAAAGTGTTATTCCAGCCTAGACAGATAGGGTTTTGGGAGTCATACCAGTGGTCCTGA
**CoV-bait-81**	CGTGCGCATTTTGGCTAAAAATTTGTCAACATCTGGAAGATCCCATTGCTGAGGAATGGCACCTTCCTCATCATCAGGGTCCGTAGTGGATTCAGGCACGTAGTCGTCCTCCTCTACGTA
**CoV-bait-82**	TTCCGCATCCTTAGCTGAACAGTCGGTCTCATAGGTGTCCTCATCAACAGGGTCTACTTCTTGAACCCCATTATCGAGGTCCTCATCCTGATCATCAAATTGCTCAGGTGGATTGAACTG
**CoV-bait-83**	CAATAGCTAACTCACGGAATGGTAGCCCTCATGGGACTACCACTCAAAACGGCGGGTGAATCACCGGTAAGTAATTTGAGTACGCCTACTCTGAAAAAGACCGGCGTCTATATGAGAGAG
**CoV-bait-84**	CCCTGTATGCCCTCAGGTTGGTATTGGAAGTTGTAATAAGCAGCAACCATAACCACAGGTAGCAAATACACAGTCAAAAAGGTGTGCTTATGCTTTATAAACATAGCCATGAAACCCACA
**CoV-bait-85**	ATTAGCATGTAATACATTACCGTCAATAATTGGGTCTGTTGCAAAATCACCCAAGTAATTAACACCAATCAAAAATGCTTCTGATGAAGAAGTGTTAACACTAGTACAGAACATAGTCCA
**CoV-bait-86**	CTTCTGCAACATGCCCTTGGTATAAACATCAATGGCCTTTGCTATGCCGCCACCGTGAGACAAATTCTCATTTGCAGCATTAACAACAAAGTCACATGGAAGTTTAACAAGGTCACTAAA
**CoV-bait-87**	TTAAATTCAGAACTAAACAAGTTATAACATTTCTCCTCTAACTCAAAAGAAGATGTAGAACATTGCTTAGTAACAAAATATAAATCAACAACACCACTATTAGGTATTGGCAAATTAATG
**CoV-bait-88**	AGTACTCTTGTTAGCCATATAACGAGAAATAGCATTCAAGGTGTACTCAACGTCACCAGGATCTCCCTTAGAAGAATTCTGGTACCAGTACAACCAGTGCACAAACTTCGCTACTTTGCC
**CoV-bait-89**	GATAGAGTTTTTCCTGCACATTAGAATCAGTACCACGAGGGGGTACTACGTGTAAAATACATGCAACACCTAACTTCTCTGCATCAAAAGCAGGTGTGTTTAACACACCAGTAAAGGGTG

**CoV-bait-90**	CATCAGTAAATGTTAGCTCTTTGGCTTTAACAGCCATAAAGTCCTTTGAATTAACCCAACCATCAGTACGTGCACTCTCAAGAAGATTTCCAATAGCACGAAGAGTTGGTTGAACACCAA
**CoV-bait-91**	CCAACATGAGAGCCGTTACCTAATTCCAAGTGGTGCATGTACACAAAATAAAGAGTTCCATTTTCTAATACATAACCTACTGACCCACAGGTACCAGCAATAAATGAGCCTTTAATAGTA
**CoV-bait-92**	ACCAAGTAGAGGAATGAAGACGTGTTTCTCGTCACCATTATCGTACTTACGTTCAACGTAGTTAATAATTGCGTTGTATGTTGCTAGTAGTGCGTGGGCTGGTTGTGGGCCTTTGTAATC
**CoV-bait-93**	TAGCTGTGCTATAATCATTGAGCATCGTCTCCAAAAGGTATTCTGCGTCCCCTGAATCACCCTGTTTCAAAGTTAGAGCTGCATAACAAAAGTTTACAAAAGGCATAACATCCTCTTTCT
**CoV-bait-94**	AAATATGCATCATTTCCAATGCAATTCTCATTAATAACAAATCCTTCACTCTCCATGATAAGGCGCATACCGAAGCTAGGCAAATTTTGAACTTTTCCTTTTGCCACTGCGGTAACCACC
**CoV-bait-95**	ATGCTCATTACGATGCACATTACAGTGTTGGTGATGGTTGAGGCTAAGGGACCCTACATAACCCCACTGTTGTATGTCAATACAATATGGGTTGTATAGGTAATCACAACTAAGTGAATG
**CoV-bait-96**	CTCCATTGAGTTGTTGGTCCACGTACTAAAAACCACTCATTATTATAACTAACCATATGGTGTGGTATAATGGAAGGTAAAAAGGATGTTCTTGACAATGCAAATTCATGTTGGTCTTTA
**CoV-bait-97**	CAAACACTACAATGTTAGTAAAATTACAGTGAGCCGTACAGAATTGTGCATCTGACCATGTCATACCATTAGCTGGTGCAGTCATGGCCACAGAAGCTGCACTAAAATTTTTACTAAAAG
**CoV-bait-98**	CAATAAGTGTGCAACAAATAATAGTTGCATCTAAAAATGCACCACAATAATATAAAAGTGGGCAATAAAGAACAATGACAGCAAAACGCGCTGCCAACATAATATAATTGCGACTCAAAG
**CoV-bait-99**	TATGTCCAGTCATACGGGCTCCCATACCTACGCTGCTGCATCTTGAGAGCTGGGTAATCAAGACCATCCATACCAGACATGCACATGTGGTGGAACCAGCCTTCGCTAGTCTCATTAACT
**CoV-bait-100**	ACCAGGACCTCAGCAAGGACTGGTAGATACCTCCCTTAACTTTGACGCGCAGAGAGCGACAATATCTTAGCTCAGACGTGCCAACGTACAAAAGCTTATCACGGGAAACGGGCCGAGATG
**CoV-bait-101**	TATTACCACGTGAGATGCTAAAATTGTGAGCACGCACCAAGTTTTGCTCAGTGTCATAATCAACAATCTTAGTAACATCTGACGCAATAACGTGTCTTGGAGCATAAACCTGATCACCAA
**CoV-bait-102**	GAAGTCGTTCAATTCTCCTTCAAAATCAGTAGTGCAAACAGTCTCATTAACAGACTAGACAATAATCACGTTAAGCGGTGCTATGAGCAAACCTGGATTACAACCATCGGGCATCTCGTT
**CoV-bait-103**	TTTTCTTGTTTAGTTTAAACTAAATGTAACAATCTTTCATTTTCGGAAAATGAATCTGCCATCGTCATAATAGCAGAATAGTCGCCATTCTTTGCACGTACATAGAATGCCCATCCAGTC
**CoV-bait-104**	AAATACACTTAACATCTGGAGTTGGTAGCTCTTTGACCTGGTCTTCACCAAAAGTCACAGTTTTACCACCTGCCTTACAAACAACATTAACTGCACCAACCTGAGACATTGGGGCATAAT
**CoV-bait-105**	CTAAGGTTATACCACCGGCTAAGGAGGCAGTGTACATAGCCATTTTATCAGCGTTGACAACACCAGGCAAAACCATAATACCATTGTAATATTGAGCACAAACAAGGTCAGCTATGTCTA
**CoV-bait-106**	AGATTTAGAGCAGCGTTCAGAGTCTCTCCGTAATACTACAGAGGAGCTCCAAAGTCTTATATATAATATCAACAACACACTAGTTGACCTTGAATGGCTCAACCGAGTTGAGACATATAT
**CoV-bait-107**	AACCGGCTTAACTAGAGACAGGCGTTAAGGTCTAATGAACAACTTCGGTTCAGATGGGGCGGGAGCTGAACCATAGACCCACAAGTACCAGCAATGAATGAACCCTTGATAGTACCCTGA
**CoV-bait-108**	AACACAATTCACAGAGAGTACCATTACAATAATCAAAAACATTAAAATGGTAATCACTGTACGTATTATAATCATAACAGAGGTAGCCTAGTGTAAACCAGTTAGCTAATCTTACTGCAC
**CoV-bait-109**	TACAAAATCACCGAAATCGTAAAAATTGCCATTTAGATCTTGGTTATCAAGAGTTATAATACCTATGTACCCTTTTGCTACAATAGCATCACAAAACGCTACACATTTAAGCATAGCGTT
**CoV-bait-110**	AAAGGTTGTTCAATTATATCAACCTCTTCTCTAACAGAGTCATCAACAATACATTCTAATTGATGAAAATCAGTGCTCGCTTCAACAACACAACCATTACTATCATTACTAATAGGCCAT
**CoV-bait-111**	CAGGTTTAACAGGGACAAACTTGTTCTCATAAACAAGTTCAGAAACTGTATTAACTATTTCAGCAGGACATCTATAACATTTATGAAGAAAAACATCAGGGCCTATAGCACACATACGTT
**CoV-bait-112**	CGAGCACCATCATAGACAGCATTATTAGCAGCATCATAAACTACATAGTGACCGTTATCAAAAGAACCTGAAAATGTTGTATAAGCAATACCATTAAGCAACTTAGAAGGTTGTGAAATT
**CoV-bait-113**	CGTTCGTAGTAAGCGTAAAACGCTCATACGAACCCTGAATACTATTGTCAAAACAAACACAAACATCCTCATTAAAATCAGTGTATTTACATACACTCTTAGTATATGAGGTAAAAGGTC

**CoV-bait-114**	CAAAGAGTCTAAGTGTATCTTTAACATCATTAGCAAGTTTATAGTCCCTAACATCAGTCCAATCAGACGTTGCAAGCCTATTAAAAACTTCAACATCTAAGGAACCAGTTGCTGAATTTT
**CoV-bait-115**	TTTTGAACTRGGTTCRKTAGGATAAGTAACAGTACAGCACCTCAAAGTTGTGTCAGAAGCAGTGACAAAATCATCAGCTTTCAAAACACCCATTTACTAAGCCTAAACTGTGATATCAA
**CoV-bait-116**	TTGAATGATAACCAAAGTGCATCAAAAGGTCCATGTGGTGCATGAAGTCCATTAGCAACCATGTATTTAAATTCAGCAGCACTCACCTTAAAATCATAAACACCCATAGTACATTTAAAA
**CoV-bait-117**	ACCATCATAGACAGCATTATTAGCAGCATCATAAACTACATAGTGACCGTTATCAAAAGGGAGCCGAAAACTGATGACCGGCTGTACTCTCGATAGGGTTTGATCACATCATTCCCGCGG
**CoV-bait-118**	GCAACATAACACCACTGCCCACTGAAACCTTTCCATGTTTCTTAACATGAGCACTAGACAGATGCTGCAGCTCATTATTTGTGTAATCATTGATGGCCTTAGCAACACCACCACCATGCT
**CoV-bait-119**	TTGAAGGTTTCAAAAGTACGTCCCTGTGTGTAGTAACTATCAATTTGTTCAACGTACTCACCATTCTTGCGCACATAGATGTACCATGTGACAGGTTTGTTTCCAACAGTACTTACAGGT
**CoV-bait-120**	CCAGCCAGGCTTCTAGTGCTGTCCCAGTGAAGGCTGCGTTCTTCCGGGCCTGCTCCACTTGGGCAGGATTGATGTGCAGGCTGAGGACTGGGTTTAGGCTCTGGAAACTCTGCTCCATGT
**CoV-bait-121**	AAACTACCTCCTGTTCATTAACAACAGCTTGTTCAGCCATTATGTGACAAACACCATCAATTGGTTTCTCAAGTGTGATTTGGGTTGGTTCCTGTTTACACAATTGTTCTTGTGCATCAA
**CoV-bait-122**	TCCAATTCCTTTATACTTACGCTGAATTGTGGAAATAATCGCCCTAACATCCATACAGACTGGCATAACTTTCCTAGTTTCCTCAGCGTGAGCTAACATTTCACGCAAGTTCCAGGCAAC
**CoV-bait-123**	GTATCACAGGGTTTATCCATATAGTGTTTGTATAAATAAAGAGTTCTGCCCAAAACATTGTGAAGAAAACGCTAAATAAAAACATTGTTTTTAAACCAAAAATAACCTTACCACTTTGCA
**CoV-bait-124**	ACCTTATAATAATAATCCCCAAAATTAGCTTCACGAGCTATATATGCAGGGTTATTAAGAACTAAACTACCAGATTTACAAAGAGCTGTAGATTGTGCAGAGCCATTRTAAACATATGGT
**CoV-bait-125**	CCAGGGTGGTGTTAAAGCTGCTTTCACGATACACAATAAATTTTTCACGCACCAGGGTGCTGTTGGTAAACGGATAAAAGCCATAGCTAAAGTTGCCGGTGTTATACTGGCACGCCAGCA
**CoV-bait-126**	AACCTGCTGTAGTAGCTGTATTGTAAGACTCTAGGTACTCACGATACTCTTCAGCATGCTTTAAACAAACTGCACCACCTAAATTGCATCTTGTGATGCAAGTGGCGGATTTCAAAGGTA
**CoV-bait-127**	GTAATCCACACAGTAACCACTACCTACTGTGAGATCACATGTTTGAACAACACTAGAAGTACTATTATCAGCATTGACAACACAACCAAGATAGCTATCAAAATAGTTAATAGGTTGCAG
**CoV-bait-128**	ATTAGAATACGATATTTGACTATGTGGCGTATAACACTGCACTCCATCAGCAGAAAGTGCATGTGTAATAAAGTGCAACACATGGTAACCATATCGTAACACTTTGGTAGGGACGTTAAA
**CoV-bait-129**	CTCTGTAGTATAAAACTCAAAACAAACATTTTCATAATCCTGAATAACAGATTCAAGATCAACAAAATCCGACATTTCTACATCTTCTTCAAATTGTGAATCAGATGTCTCCACACATGG
**CoV-bait-130**	ACGGTTCCAGACTGGAGGAACAAGAAAATTAGTAACTGCAATGCTTAGAGGGAAATGCATAAATGGTGACAGAAACTTAGGTTCTGTCAAATTGTGCAAAGCCCTATAGGCGTAATAAAT
**CoV-bait-131**	TACCGCTTGAGTATCTTCACATCTTTAAACACAAGCCCACTAACACCACTCATACTGAACTTGTTAGTGCTTGTGTGATAATCGATGATGTCTTCATCCCTCTGAAGGAAATGAATTGTG
**CoV-bait-132**	AGCAAATGTGATGGCAAATCCCATGAACACAACGTTCACGTGGTAAATGGCAGCAAGCACAAAGCATGCAAAAGTGAGTGGCCACATGAGCCAAAGAAGCACAAGCTTTAGTATATACAG
**CoV-bait-133**	ACACAGCGTTAACTATTACTGTGCCTGTCTCTTGTGAGACGAAGGAGTACATTTAACGTGTGACAAAAGCCAAACGTAAGTTCGTTTAGACTCCTTTCAAGTGACCTTAGAGAGGCACAT
**CoV-bait-134**	ACGAGAAGATGTCCTTCTCAGATATTTTCATGTAATCTATGTGAGCTTCTTCCCAACACATATCTGCAAGACTTGAGGGCGCTACACCATAGCATTGTAAACTATAGACGTCAGCATTGT
**CoV-bait-135**	ATGGAGCTGCTTGTGGAAAGGACATGAGGTGCATACCTCTGCCACAGAAATCAAGTCTCTGCGACTGTCCTTGCACACACTCTGACATCTTCTGTGCTGCTAATTTAGCTGATGCACGTA
**CoV-bait-136**	AAACTGCATAACAAAACCACAATCTTTCAAATCAACACCATAAAAAGCATTTACAACTGCATCTGCCTCCATATAAAAACAACTCATAATGTCAACTAAATTATGTTGACGTAAAATAAT

**CoV-bait-137**	GCCCTTACAAACTTATATTTTGGGGTTTCAGGGTTAATTTGGTCAACACCCAACTGAAGTACTGATCCTGTCATTTTGTAGGACACTACTTTAAGATATGCTGTTCCATATTGAACAGTA
**CoV-bait-138**	TGACAATTACACCAGTTCCCTTATAACCATAAATGTGATAAGTAATACATTCATTTAAAACTAGCGGTGTGGTCTTTTGTTTATAACCACACACAGTAGCAGATGTTCTAGTGGGTTTGA
**CoV-bait-139**	ACTACAATGTCTGAAAAATTGCAGTGAGCCGTACAAAATTGCGAACTTGACCATTCCATACCGTTAGACGGTGCTGTCATAGCTACAGAAGAAGCACTAAAATTCTTACTCCAATGTATA
**CoV-bait-140**	ATACAACCACCATCATAACAATCAAAATATTTATCTACTACTTCTACTACAAATAATAATTGACGTATATCACACATCGTCGGTAAATTATAACGATAATAATCATAATCCGATATCGCC
**CoV-bait-141**	TGACATGTAGCTCCGCGCGGGTGGCCGTGCTTCTGCGCGCACTGAATTACTATGTTCACAGCAAACAGGGTCGGGCGTACCAGCAGCGAATCATTCGTCACGCGCCGACGGTTAAACCCG
**CoV-bait-142**	GTACTAGCATAGACCCAGTTTAATTCGTGTTCAGTTGCATAACAAAGCCATGTATCAGATATACCTTTTCGGTTCTGTGTACACGGCCTGCAAACACAGCCTGGCCTCAGCATCGAGGGC
**CoV-bait-143**	CTAGCCCGCTCGGACGAGGGAATGAAAAACGGTGGCCACCTGTAAGATTCGTAAATGTCCCATCGCGATTCTGGATTCATATTAGCCCGAGCTCCACTCTCTCAACAGTACGACCAGCCT
**CoV-bait-144**	GGGTAACTTGTTCAAAAGAGTCCTCAACGTTTGCAACTGCCACAGTTTCTTCTTCGACATCAGCAGTTGAGACATTCTCTTCTTCAATTTGTTCAACATCTTCGTTGTCAGAAATCGAAG
**CoV-bait-145**	CAACTAATTGGCTATACCTCGTTTAGTCGCTTAAGCGCCTGCCCGTAGGGAGGTGAGGTTTAAAAGGCTAAAGAACAAATGCCAAAATTGGCACTTGCAAGTTTTATAAAAGCCTTAAAG
**CoV-bait-146**	AGAATTGTTGCACACTTGTTAGCTATGTTTGCACCCACAGCCAAACTCGGAACAGTTCCCTCGTCGGTCGCCCAGAAATCCACAAATAAGCATGCCGGTGGGCCATGCATACCATCGACC
**CoV-bait-147**	TGGTACTGTCATAGAAAACAATGTGTTAAAATTGGCACAATGAATGATACAATCGTCACTAGTACAATCAGAACAATTTGGGTGATATGTGCGATCCCAGTACTTAAAGTATTTTTGGAA
**CoV-bait-148**	GTCCATTGCCAGAACCATTAGTAAGCATCTTGACACAATTACGGACAGGCTTAACGCCACCTCGAACAGCATCAACATAAGTTTTTGCCGGGTCAACAGAAAAAGCACAAGATGTAAGCA
**CoV-bait-149**	CTACTACGCATAGTCACATGAAAGGCTCCTTGTGGTTTGCCGTTATAAGCAGCTAAAACAGTAAAAGTCTCACCAGGCTTAACCACACCAAATGTATATTTTGGCGTACGAGAATTTTGC
**CoV-bait-150**	GCAGCCTCCCTATATGCAGCAGTATCCATTTTACCGCTGTAATACCTATATTTATTATACAAACTCAAATATCTATTAAAAGCAACATCAGACAAAGAATTCTTAAGCTTACAATAAGAA
**CoV-bait-151**	AATGCTTGTGGTTGGCAACTACAAGGGTTACCTCCACAATGATCACTTTTAATAGCAAGACCCGAACAGTGCTCACCTATGCCAACTAAGTATTTAGTTTGGGGGCACTTGTTAGATGTA
**CoV-bait-152**	GTTGCAGCTTTGCCGCATTCACATTTTTGTGGTTTTCCAATCTTAACAAAGTATCTCATAGTTGTAAGTTCAAGACCACCAGCCCAAAGCACAAAGATCAGAATGTCTGCTAAGCCATAG
**CoV-bait-153**	CGCAGCATCCACAACAACCAGTGGAAATGCAGCAGAACACTAGTAATGACACAACAAAGATGAGAACAATGAAAATAATCAACCAAACCCACCACGGCCACTTGATATATGTCTCAACTC
**CoV-bait-154**	GTAAAATCGGTAAAATTACAGTGTGCAGTACAGAATTGGGCAGTAGTCCATGCCATACCAGAAGTAGGTGCTGTCATGGCTACAGAAGCAGCAGTAAAATTCTTACTCCAAGAGATAGTA
**CoV-bait-155**	CCAATCGTGTTGCAGATGCTGCAGGTATAATACTAAGAGGCAAGCAACCATTACGTGCTTGTTCCATTATAGTATTAACAGATGACATGTCTAACTTTTTAAGCATTCCAAATAGCAAAC
**CoV-bait-156**	GCAAGTTGGATAGGTCTTTAACACCTGTGTTTGGACGTTCTACACCCGTGATGTCCAGACCAAACTTATAACCAACATACAATTGTGCAAACACAAAATCACCCAACATTGTTCTCGCTA
**CoV-bait-157**	ACATACAATCGTTTCGCTGTGTAAACTCGTCGTCAGAAACCTCATGAGAAACGTCAACTTGTTCAGTTGAAGTCTCAGAAATGCACTCTGATTCACCTTCTTCTAAATCTGAAGCCTCTA
**CoV-bait-158**	GCTGTTGCTTCACAAAGTTTTCCGATGTTATAGTTATAAGGAGTAACCATTGCTTGATGGTAACGTGCAATGGCTGCGAATGAAAAGATGTAATAGGCGGGAGAACCACGAGCGTAGGCA

**CoV-bait-159**	AATATTGCCAACTGCTTGATTGAAGGAGTCAGCAAGAATTTTCTGGTTCTCTTGCAGGACATTAGTCTGCAGAGCAACATAATTAAGACGTGCCTGAACTCCAGTTGAAAAAGGAATAGC
**CoV-bait-160**	CAAAACAATTATTTCTATGTTGTTTCAAATGCATAACGCCATTAGTACGACTAATGGGCCAGTTAGCATGACAGAGTTTAAGGTAGGTTACATACTGCTGCAGGGTGAGATCAAGCTCTT
**CoV-bait-161**	TGATCATACACATCACGCACAGTGGCTGTTGCATATGACTTCTTAATTGACTCTTCAAGTGCTTTTATTGTTTCAGCAGCAAAAAGCATTAATGATAATGGTGCACGATTGGCCTTTTGA
**CoV-bait-162**	GGCAAAAACCCAAGTGCTGAGACCAGGGTTTTATATGATATCAGCGTACCATCGGTAACGCCAACTAATTTTGCAAGTGGTTCATCACAGCGATACTCTGCGCCGCAGTCATGCCACGTC
**CoV-bait-163**	TAGGTAGTTGTAAATTCTGGCGTTGCCGGAATTAGAAGTTGTGATTGTGTGTAAAGCTGCATTAGAGGACCTGTCGCCCTGGTAAATTAACACAGCCAAGTCACGATCATCAGTGGATTC
**CoV-bait-164**	ATGTGTGTCATGGACATGATAGGCATTAGGTATGAATAATAGGATGACATATCACAACAGCCATTACCAGGCTGTGTACTAATGAAATCTCCAAAGTCATAAATCTGACCTAGAAGATCC
**CoV-bait-165**	GAATCATGAGATGCGTGTATATTTGTGATTTGGTTGTTTACATTGGTAGTAGACTGGCTAGTACCCATGTAAACTAATTTGGTAGTGGTATGGTGTAAACACCATGCGTAGTAAAATCAA
**CoV-bait-166**	TACATTTGAGCATAAAAAGCCTCTTGCAAAAAGTTGTTAACATACGACATGTCAGTGAGTGTTTGGAAGGAATCTAGGATTCCATCCTGCAACTCCTGAGCCAAAACTCGTATGTAATCT
**CoV-bait-167**	GCCTCCTCCGTTTCAAAAACGGATAAGGAGCATTGCTTACTGACAAAATATAGGTCAACTAAACCTTTATAAGGTATAGGTAAATTACAGCGCTGAGTTTTAAATCTAGCGCAGTTAGTC
**CoV-bait-168**	TGGATGGCACAGTGTGTAATGTAAGTCGAGCAGCTGCACAGGAAAGCGCAAAAGCAAAGGCATACACTGCACAGATTGCCAATATAGTGTAAACTACACGCCGCGATGGTACAGCACCAG
**CoV-bait-169**	ATAATCAAGCATAGCCTTAGCAAGATGTGCATAACAAGCAAGACGATAGTCAGCTTCACTAGCACTACCACTATAGTACTTATACTTGTTGTAACTAGCAGCATACTGTTTAAGCTTCTC
**CoV-bait-170**	CTGGCGTACCTTTGGTACGTAACTGAAATCGCTGTAAATCATACAAACTATACGATGATAACTGCATTAAAGTACTATTACGCCAAAAGACGTAATTAGCATGAGCCTGGTCACCATCAA
**CoV-bait-171**	AAACAATGTGTAACAAACACTGTAAAATCAGTAAAATTACAGTGAGCCGTGCAAAATTCAGAGGTAGACCACCGCATACCGGCAGGTGGTGCTGTCATAGCTACAGAAGCAGCACTGAAA
**CoV-bait-172**	CTGGATTACTAGTGTAAACGACCCAAAATATAAGTAAAGCAGTAAACAACACTTTGCATAACACAGTCCTATAACTAGCCACTAAACTCTTAGCGCCAGCCTTCAATAGACAAAAGAAGA
**CoV-bait-173**	CCAGTATTGGTAAAGCTTCCTTTCGCTACTGCCGGTTCGCTTCCACCCAACAAATTCCCATCTAACGGGAGACACGTATCCAGGATTATACAGAACATCAGCCGGTCGTGGGCCGTATCG
**CoV-bait-174**	CCTTCAACCCATATTGCTTTAAGACTAATAGCGTCCAAGACTGGATAATAAATTGGCGCTTTCGAATTTTCATCAGTGTCAGGTGTTAACTTGTAAGTGAAACCATCCTTTGACCTAAAG
**CoV-bait-175**	AGCACCTACACCTTTAATGTTATAAATGTCATAATGATGACACACATTTAAATGTATATATTGTAATGGAGGATGTTCAACTCCATAAATAATATTACCCATTTCAAAACTAATACCTAC
**CoV-bait-176**	TGGCAACGAAGAGCTTCTGAGGAGTGGTTCCGGTAGCGTGTCCAGCGCTGTAGACGTACATATCGCCGTGGTCACCCTGGTATGGGAACAGGCCTTGGTATGTGATAGTGATGTTAGAGT
**CoV-bait-177**	TACCACAAAACCCATACCGCAAAGATTGCGACTTAACACATTCTTCTATCTTTTGCATAGCCAACCTACGAGATTGTTCAGCTTTATAAGCCGCCAACTGTAGAGATGTAACATAAGTGG
**CoV-bait-178**	TAACAACTTTCTGATACTGTGTTTGAAAGTATTCAGTAGTTACTGCAAGCGTCAATTCTAATGGAACACTAACATTTGCTGTAACATTTAGTAAAGGAACAACTGTTGTATCAACACCTT
**CoV-bait-179**	AATACCACCTGTAGCTAAGACAGTTTCAGATAAAACCTGAGCGCATTCATTATACAACCTATAAATGCGCTCAGACCAAGTACAACAGTTAGTGTGTTTGCGAGCAAGTACTAAAGATGC
**CoV-bait-180**	AATGCATTAATAACCCAAACTAACATTATTGTAAACACAACGGTAGCATAAGCTTTAAAAACACCTTGAAACTTTATGACCATTGCAAAGATTAGTACAACAGCGACTAATATTAAAAAC

**CoV-bait-181**	CCAGCCCATGCACGTACAACAACATACTCAGTAGGCATGAGGACTGTGTGAAATAACATTATACCGTTAGGAGCAGCGTTAGCGATTGAAAATAAATGAGTGCCATTTCCACAAAAACCA
**CoV-bait-182**	AGGTCTGAGAATCTACAATGAGCAGTACAAAACTGTGATGTAGACCAACTCATACCTGAAGCCGGTGCTACCATAGATATAGCAGCAGCTGTGTTGTTGTAAACATCTGTAAGCAAACCA
**CoV-bait-183**	AAAACCCAGATACTTTAATATCTGATTTAACAGTTGGAACTGTGTTGTATGTGAGACCATTAGCAACATAAAAAGTTGCAAAAGCTACAACATACACAAAAGCTATAACTGCAAAAAGCT
**CoV-bait-184**	ACAATTAATATTACCAACCTCCTGAAAAGACAGTGCTGTAAAATACAGCTCAGGTGGTACTTGCTCTAAGCGACAACCAAAATTGCCACAAAAAGATTGCTGTGTTTTATTATAATAAGT
**CoV-bait-185**	AACTTATCTAGCAATCGACCCTGAGTACAAATAGTATCAATGGTAACAAACTCACCATTAACACGGCAATAAATGTATAATTTCTCGCCTTCAACGGGAACATTATTAATTGTCAAAGCT
**CoV-bait-186**	AACCATAAGTAGACTGTACTGCGATTTTCCACGCACTAAAATTAACAAACCGATACGGTCGCGTGTATCCCAAATACCATCAGAGTTGTGTTCGCGTACATCAAAATAAACACCATGAAC
**CoV-bait-187**	GACAATATACAATATTGTGTAACCAAACACCATTAAGTGTTGATCCATTATATGTTACAGATGTCATACATCGTTCTACCATACCTGAAGGATGTAAAAGTATCTTTATACCAGCCTGCA
**CoV-bait-188**	GATTTGGTACGTCTCCAAAGGCGAATGCTGTTGACAAAATACATTACCCACAGCACACCCGTGACGCAAGCCATAAGGATGCTAAACGCAAAGAAAACCCAGTTCACATTAAAGCTGGCA
**CoV-bait-189**	TGACCATTTGTTGCATTTCTAACATCAACAAAGATATCACCAGTAGGTGTTATCTTATTTATAGTAAAATATTTCTCTATACGAGAACATACTTCAGTATAATTATCTATAGGCCTACTA
**CoV-bait-190**	AATAACAGCTGGAATCAAAAACATTTGCAAAAACAATGTTTTATGCTTCAAAAGACTCATGCACAAGATAGAAACACCACTGACAATCAAAAACAATGGTGTAATATAAGCTGGAGAAAC
**CoV-bait-191**	TAGAACAAAGCAAACCTTAGCAGAAGCATTGAGCAAGAATCTAATGAATGCATGATGAATACAGCGCAGCCTATGCACGTAGTAGTCAAAAGCATTTTCATGGATAAAAGCCCAACAGAA
**CoV-bait-192**	GTACTTAAATTCAGCTGGGCTAACCATAAAATCGTAAACACCAAGTGAAAGTTTACAAAACCTGTTAACCCAGTACAAAATACCATAAAACACACAACAAACATAGCCAACAGCAACGTA
**CoV-bait-193**	AGGATCAATAGTTTTAACTGCCACATCTTCTGAAAAAGCAACACCAGCACCACCAAGCTTTCTAAAGCGCAGTGAATGAATAGCACTACAATCAGTGGGGTAATAAGAATCACCTTCCAC
**CoV-bait-194**	TTGTACTTAAGTTTTGTAACATGTTTAACCAAAGGATTCATATCATAAAAATGATACTTAACACCAGCTTTACGAACAGCTGGTATACCTTTTGGATTACCTATGTCATGGACTACATTA
**CoV-bait-195**	GGTTGGGTAGTATCCTCACGTGGTTGCAGAATACGCAATGCTCCGTCTTTGCAAACACCTAGCGAACTATATGTTAGTACAGGTTCAGAACAACTACTATTACCATCAGTCATATAGTAA
**CoV-bait-196**	TAGTAGTAGGTTACGTCTAAAAGTAGACGCCTTAAACAGTGTGAAGTAAATCACAAACACACTGGTAAAGAAAAAGCCAGAAGCAATAGAAAATTGCCTAACTGGCTCTAGCTGCTGGTC
**CoV-bait-197**	GCTGTAAACGTGTGTGGACGTAAGTTGTGAAAGGGGTTGGATGGCGAGCATGGCTTGGCGCGTGACACTTTTGATAGTATGTGTTTCATTAGCATGTTGGTCATATCCTGTAACTATGAT
**CoV-bait-198**	TCTGGGGCTCRTCAAGATTCCCAGACAAATTCTGCACTTTGGCCAACTCTAATCTTGATCCAAAGAAAAACGCACCAGCTGTGGGTGCGAGTTCTGCAAGAATGGGGAACTGTGGRTCAC
**CoV-bait-199**	GATGTTAAATTATCTACATCTGAAGTTATGGCTACACATATTTTCTTACCGTTGTGAGTGACAAAGAATGATTTAACATCACTCTGCCTGTCAGCAGCATCTAAACCAAGCGCCTGAGCT
**CoV-bait-200**	CATGCACAGCAGAATTAAGTGAATTAAATGCACCAGAAATTGTCTCCATAGCCTTATTAAAGGAATCTGCTAGAAGCTTCTGATTATCTAACATAACACCTGTTTGCAGAGCTACATAAT
**CoV-bait-201**	ACCCACCAAAACAGCCAAAACATCCACCACAGCAACCTGTGCATAGGAAGATTGTTACGACAATTATTATAAAAGTACAAATGGCTAAAAAGATAAGAAGCCACACCCACCATGGCCATT
**CoV-bait-202**	AACCTTTTCACTTGCACCCAAATAATTAACACCAATCAAGAATGCTTCTGAAGAAGATGCATTCACTGCTGTACAAAACATTGTCCACCATGCACAATCCTGTGCAATGTCATATAAACT

**CoV-bait-203**	AGGATCACCAACATATACTATATGTGAGTAAGACAATCTAGCATTAACACTGCTAAGTTCATAATTAGTAAGCATAGATATCTCGTCCACGACGACTATGTCACACTTTATGTCCGGTAA
**CoV-bait-204**	CGGCATTGCATAAATCCACTAGCGGCGGCCTCAGAATAGTATGAGACGGCTTCACTAGCAGTGCAAAAGCCAAAAGCTGAAATTTCTGCATCATTGGCAAAAGCCAATGTAACATGGTTG
**CoV-bait-205**	ATGGGTATAACATAATTGCCAGGACTAACTAAGACATCCTTGTATGCCTGGTACAACTTTGATTTAACATCACCAGACTTTCCAGTTGGAGGTACAATATGGGCAATACACGAGTACCCA
**CoV-bait-206**	AAAAATTTAAAGGGATCCAAATCCTTGTCTATTTGTACAAAGCGACCTTTATACAAACACTGACCATTAACAGTATTGTGTGGTACATGAGCACGACAATAGACACATATAGAGGCTCCA
**CoV-bait-207**	TAAAAGAGGTTTGTACACACCTCTATTTAATAACAATTGTTTATGCCACACATCTGACACACCTAGCAACTCCCGCTGGTGTAAATTGCCACACTTGTGTTGAACATAATCAAGGTACAA
**CoV-bait-208**	CAAGAACCATACCTCTAATAATAGACTTTGTATTTTTAAGAAAATAAAGATAAACAACCTCAACCTTACCACCAACTTTCATACCAAATTTACATGGCGGGTCAAGATCAACAAAAATTT
**CoV-bait-209**	TATAATGATACTTCTTAGTCCGCCACCAAGCACATCTAAAGTTAAGTAATGTTGACACCACGGGTCGCCAACTCGATCAGGGAATATCTGCGCCGTACGTACAAGACTCGGTTACTCATT
**CoV-bait-210**	AGACAAATCTTAAAGCTAAGACAGTTGTTGTAACAATGCCATCAATGTAGTAACCATATGCTATAAGCATAGGCCCTATAAGTGTCATTGATACAATCTTAAACATTAAGATACCGACAC
**CoV-bait-211**	GCTATTGCATAAGACTGCTTAGATGCTTCTTCAGTAGCACGTAATGTTTCAGCAGCAAATAATTTTAAAGGTTCTGTAGTAGTATTAGCAAGTGTATAATCACCGCTTTCACTCCAATCA
**CoV-bait-212**	GTGCACAAATTAGGTCCCTAGCACTAGGTGGTCCTTTTTTCATGCACTCGTCATATCCTTTCATATAACCTGGATCTGATAATGTAACCTTATCAAAAAGCAGATCTTCTAAAGCACTGC
**CoV-bait-213**	CCACCCATACAGATGCTACTGGCAGAAACATTATAAGCCAAGATGCATGCATAACAACAGCTGGTTCATTAGTCACATAAAAAGTGCTATAATAATAAAATGCACTATAAGGTACTATTA
**CoV-bait-214**	GAGACAAGTTAAGCATAGTGGTATTAATCTGACTGAGAGAACCAAAATTAGGTATCTCAGACGTAATATTTTTATAAAACTCTTCCAGCTCATCCTCAAAAGTAATATCAGTGTTATTAC
**CoV-bait-215**	TCACCACTAGTTGTACCACCAGGTTTTATGTAAAAACCACCTGTACAATGTACAACCTCTGTGAGTACTTGAGCTAACTCATTAGAGAGTCGATAGAATCTATCACTATGTGTGCAACAA
**CoV-bait-216**	TATCAGTGGGTTTAACAAGTCTGCGTAGAGATGTTGTAAGATCATTAGCGACTTCTTCACAGATGAAGGTGTGCCCTAAACCTGTGGTGTCGCAATCCACACAATTCCAATTATGTCTAC
**CoV-bait-217**	CTTCCCTCTCATCATCAGTAACATTGTAATTTTTCTCAAAAAGACAATCCTTAATATTAAACCACTGCTGTAAACTATTTTTTAAGTTACCATCAGTAGTTGCATCACAACTAAAATTTT
**CoV-bait-218**	CGCAACGTCTTCTTGGTTGCGCACTTGACCGCACACGCCGATTTTAGGGATAGTCCATAGCTCAAGATCAGCGCACTGCTAATCTTGTTGACAACAAGCTTATTACTGCTAATTTTTAGC
**CoV-bait-219**	GACAAAACAATCATGTATAGCAAGGCACCTAGTCATGATAGCATCACCACTAGCTACATGCTCATTTCTATGAATGTTGCAAACTTCATGATGATTCATGCTCAAAGAACCTGTGTAGCC
**CoV-bait-220**	AACTATCATAAAACAACTTACTACCACTTAACAAAAACTCTAAATAACTTTAACAACTCAAATACTAATATTCTCTACCAACCACCACAAACATCAATCACTTCTACTATTCTACAAAAT
**CoV-bait-221**	CTCTTAGCAACATTCATAGCAGTACGTAACTGCTTAATGAGCTGAGGTGAAGAGCCATTAGAAACTGCTTCTTCATAATTATGTCTTGCATTTTCATATGCAACATATGAAGGCATAGCA
**CoV-bait-222**	CTTTCTTGACCAAGAAGCGCTTAAGAACACCAACATCTGAAAACACCATATCGGTGTCCAGAGTGAAGGATTTCTTAGCATGGTCATAGTAGACAGTGGTCTTGCCCCTCTGCAAGAACA
**CoV-bait-223**	TGTCGACCTGAACATTGCCTTTACACATAAGCTTAAAACACTGTTTACTATCTGGATGAACTGGCAAGAATTTGTTGTCATAGACCATTTCTGACACGGTCCTAACAATTTCAGCAGGGC
**CoV-bait-224**	TGTCTGAAAACTCATCTACAGCACGCCGCCTACGACGTGTATCCTCAGTAAGTTCAAGGGTTGTTTCTGGTAACACAAGTTTTAGAACATAATGCAAGTCTTGCAAAGCTATTATCAAAG
**CoV-bait-225**	ATTCAAATCTTGGTTATCAAGTGTCAAAATGCCCACGATACCCTTTTCAACCATGGCATCACATAGGCGAACACATTTGAGCATAGCGTTTGCCACAATGACACCTAACTGTGCATAGAC
**CoV-bait-226**	ACCACCCTGTTGATAAAAATGAAAGTAAAGCCAATCAGAATTAACATCGTACGTAAAATTACGCCGATATAAACAGGAGGCCACAGCCTTCTCATAATGCCACAACTCTTTGCGTGGTGA
**CoV-bait-227**	GCCTGCTCTGTTTGTTTGCCAGCTTGAAGTCTGACAGTGGCACCAATAAAACCAAGAACAGCACCTCGCCTTAACGTGTTAAGGTTTTTAACGAAGTACAAGTACTTAATTTGTGTACCA

**CoV-bait-228**	TCCGTCTTAACAATGTCATCAACAAAAACACCTGCTGACAAAATTCTAGAAGGGTCTGGGTAAGGTAGGTAATAATCACCATCTGGTCCTACAATTTGTAGCGTGTGTTGCGAACAAAAT
**CoV-bait-229**	AAACAGACCTTCCCGAGCCTTCAATATAGTAACCTTGAGGGAGTACCGTACCAGGCGGAAACCTAGTCGGAATAGCCTCATCACTGCTTGGGTCCCGATCGACAACGTCAGCTGGGGTAT
**CoV-bait-230**	GCAGAATTGGTTGCAGCAAACCCTTCCTGTATAGCACCTATCGCGTTGTTAAATGCACTTGCTATCATTTTCTGATTTTCACTAAGAACATTCATAGTGACACCAAGACCATTAATTCTA
**CoV-bait-231**	AATCAATTCCAGTGGAAAACCCTAATTGAAGTGGGAAGTTAGTCCCTATGTTGTCACGCGTAGCATGAGCGCCTTCAGCATCGAACCCAACCCATGCTCTAACACGTTTGACAGCCTCTT
**CoV-bait-232**	CTGGATTGGTCGGCAACAAGACCCCTTCCTCTCAATATTTCAACTATATCATCATGCAATTCTTCAAAACCTTTAACATCAAGAACTAGACATCGACCTAGCATATGAGGATTTTCAAAA
**CoV-bait-233**	GTCTGCTTCATAGCAATCAGCAAGTTGTAATTGATATGTAGCACATGTCAACCAACAGTTATTAGCAACCTGTTTTAGCACACGAATTCCATTAAAGACTGTAGATGGGTAGTCAAACAT
**CoV-bait-234**	CTTCGGTGTCAACAAGAGAAACTTGCTCAACAAATTCTTCAACATCTACAACTGTAACATCAGCAGCTGAGACGTTCTCTTTTTCTATTTGTTCAACATCTTCGTTATCAGAAATCGAAG
**CoV-bait-235**	TGCAGGCTCTTAACGGACTCATTATGAATCGCATAACTATCAACAGTAAGCAAACGGTTGATATTAGCGCTAACAGCCTGAAATATGTTGAACACGGAATTGGCATAGGCGGTTGTAGCA
**CoV-bait-236**	TTTATCCTGAGGTGTTGGTTCGGGTGCGCAGATGAGTCCTGCAAGCATGTTGTTGGGATCTGGTACGTACTGGTACTTTTCGTTGATTTTGCAAATATGTGAGGAACAGAATTCGTGAAT
**CoV-bait-237**	TTGCGGCTTCCTGTTATTTTACAAGGGATTTTGTGGCATCCTGGTGTTCACGATCCCGGCAGAAGGCGTCGGCCAGCTGTAACGGAGAAGACGTGCTGACCGCCACGGGTCCGAATACCT
**CoV-bait-238**	CTGGACGAATCCAAACTTAAAACATTATGGCAAATAACCAGGAACGCTAGGATCGAGGTTGAACTAACCTCCATGTGTAATGCTGTACTTAACAAAAGGGCTGGTCTATCACAAATGCGC
**CoV-bait-239**	ATAAGTGGTGCGGTCATTACAATTAGAACCTACACAACCCAACAAACCAGTGAAGTTATAGGTAGTGTCGAATAAAGCATTCTCACTACGAACTACATCAGCTGTAATCTCTGAAACTAG
**CoV-bait-240**	ATCCACTGACAAAACATTCTGGCATACTACCAGAAATGCAACAATAGAACTAGAACTAAGTTCTAATTGCAATGCAGCTCTTAATAATAATCCAGGTCTCGCGCATATGCGATAAAGCAA
**CoV-bait-241**	TTTCAGGCATGAATGACCATGCTGAACCTGTGCGGCATAACAAGCGGAAGGAGCTAACAAAATAGCTCAACCACATGAGCCCTGAAATACACGCAAATGCAATGGCAAAGCCAAAGGCAA
**CoV-bait-242**	TGTGTGTTCTCCAAAATAACACTGCTTATATAAATAAAAACAGTATAATCATAACCATGCTCCACTCTATCCAAGAGTAAAACACACTTACTAAACAAACTGACAATTTTTGCACTGTCA
**CoV-bait-243**	AAACCTTCCAGACGACTGAAAATAATGTTAATAGATGATGAAATAGCCCCAAAAGTATTGCCTAACTGCAAAACCAAAGTTTGCAGCTGTGTGGCATGTTGATTTATGACAGCTTGAATC
**CoV-bait-244**	TATAATAGGTCAGAAATAGCAGAACGCTTAGAATTACAACTCGAACCTAAACAGCCCATAAGTGCTGTAAAGTTGTAAGTTGAATCAAAATTACTATTCTCAGTTGGCACAATGTCTGAA
**CoV-bait-245**	CAGAATTAAAGCCACAGTGGTTGTGGAGTGTACATACTCCTCAGCACCCTGTACAACATCATTAATGAGCTGTACATCTTGTGGGGGTATAGTGGGAGATGGATTAAAAGCATCGACCAC
**CoV-bait-246**	TATTATAGGCTACAATACCAGACACCTTAAACAAAGGAGGCTTGGTAACTAATAACCAAAATCTCATCAACAATTGATAAAAAATACACAAAAGACCCACGCACTTTGCACCTGGATTAG
**CoV-bait-247**	GAGTCTAATGCACAATCAGCTACATTAATAGGCCTGCCCGCCAATGAATATTGTACGAGCAATTCACGCTTCACTAGAGGGGCTACATACAAATTAACCAAATCACCAACACAATTGCCG
**CoV-bait-248**	CTATCATACCAATTGTCTTATTAAAATCTATACCAAACGCAAAATCATTACCATCAAAAATATCAACCCACTCACCACTACTAGTAAAAAATGGGGCTGGCCAAACAAATGGATGAATAT
**CoV-bait-249**	AAAACTTGCAAATGCAACAGTCCAGAAGTCAGTGGTACTAGCAGTTGTGATGTTAAAATTAACTGCTTCTATTGAACCGAGATCAAAATACTTAAACCCATTGATATAAACCTGACCAGT

**CoV-bait-250**	ATAACTATAACATGGTGGATACAAACATGAAAACACCGACGTTACAATAGCAAACGGCCACATAAGCCACAAAGTAATAATCTTAATCCAATAAAAGAACCTGTTACGGGAGGCATAACC
**CoV-bait-251**	GCCATGAAGGTCACTACCACCATGCACGCACCCTGGAAGTTCCAAATGGTGCATATAAACAAATTCAACACCTTCATTAGTCACATTATAACCAACACTACCACACGATCCTGACATGAA
**CoV-bait-252**	CTCCCGGTATCTCAATCATTCTACAATAACAATATAAGGGGTGCATTTTATCACACTCCTCTAATAACAATATAAACACAATAATGGAAAGCCCGTTTATAATCGCGGGTGTTTCCTCAT
**CoV-bait-253**	TAATCAACTGTGGCAACATATCAAGTGGAACTACATTACAAGATGCGTACACTGTCAATATTACCATAACAAGCCAATAACCCAACCTAGATAGAAAACCTTTAGTGGCCTTCTTTACAT
**CoV-bait-254**	CAGACAATACTTGCGCCGCCTCATTAGCGAGCCTATAAATGCGCTCATTCCAGTCACAACAACCTTTATGCCTGCGTGCTAATATTAGACTAGCACTAAGTCTTAAAAGTGATGGCACAG
**CoV-bait-255**	TTCCCCCTGAAACCACATACTGTTCATTAATGTCGCTACAAGGATTAACTTTATAATAACTGGTTAGATTGTCACTATGTAAAACAAAAGTGTCTATGGAACCTGTAGCATCTAGCAAAA
**CoV-bait-256**	TACGCGCGTGAAGTCAAGTCCAGGATGGTGAAACCCAGCGCTGTCCTGGCTGTTTTCCCCCGCGGCCCTTACTCCCAGCAGTTCCTTTGGTCTTATTGGCATTGCCGCAGGCTTCGGGTC
**CoV-bait-257**	AATTTCTTCAGAGCTACCTTGCTCTTCAAAATATTCACACATTCTGGTATAAAACACTGCATGAACTTCCTCAATGGTACACTCTGTTTCAAATACAATTTCATCAATTGCCTTATCAAG
**CoV-bait-258**	AGGGCAGCAAATACCATATTTCGTTCATCAGTATTTCCACTGTCAGACTCATACCCATCAGGTAGGTCTTCCTGTGGTTCATCATCATCTTCATTATCTGAGTCACTATCCTCAGGAAAC
**CoV-bait-259**	GTTCACCTGCTTTACTATCCACATTATAATATATCCTGATGCGGGGTCCGAAGAAAACAGACCAACACATGCCATATGCATATTAATATAAAACTCACCAGATGACCCAAAGGTCTCAAA
**CoV-bait-260**	CACTAAAATTACAGTGAGCCGTACAAAACTGATTAGCAGACCACTCCATACCAATAACTGGTGCAGTCATTGCAACGGAAGAGGCACTAAAATTTTTACTCCAATGAATAGCACCAAGAG
**CoV-bait-261**	TAAAGCGGACCTGTACATTTTAGAATCACAATCTGATCCCAAACAACCCATAAGTGAGGTAAAGTTGTATGTAGTGTCAAACTGCATGTTTTCATTACGTACAACGTCCGATGTGAGTTC
**CoV-bait-262**	AGTATTATAAGCTACAATGCCAGAAACCTTAAATATAGGCGGCTTAGTAGCTAACAGCCAGAACCTCAGCAATAGCTGATAGAACAAACAAAGCAATCCAACACATTTTGCACCAGGGTT
**CoV-bait-263**	TAACTTAACAGGTTTACAAAGCATCTGTGAAAAATACACACATGCATTCTTAACCTGATTTACATTGGAACCATTATTGTTAAAAACAATAAAGTCTGTGATTATGCTACAATTTTTAAG
**CoV-bait-264**	TACGAACGTGCAACTCTTACCCCGGGGTGGCTACGTTTAGCATTTAATACTAGTATGTTATTCTAGCTCTTCCACCGCTTGGCAACATCTGGAGGCAGGCTCGAAACAGGGCCGAAACAA
**CoV-bait-265**	AGCATATATACTGGGTAACATAGGGTAAGGACCCTCTAAACACAGTACTATAACGTTCAACACAAAAGCCAAAATATTAGTAAAAATGACTCCTGAATAATCACCAAATGCTCTCCTAAA
**CoV-bait-266**	CTACGTTGACCATCACTCTCAAAGTAACAATAAACATAACCATTGCGAACCTCAACAGAAGTAACCTCATAATAAGCTTCATCAGTATGAATTATGGGCCTTTTAAATTGTGCACTTAAA
**CoV-bait-267**	CCACGAGTCTGTAGTCCAATCCCAACCTTCCTGATGGCACAATCTAATACAGGAGACTAAATCATTAGTGTCAACATCTGACTCTAAATCCTTTGCCTCCGTTCGCGTGGATCCAACAAA
**CoV-bait-268**	GCTCGCATAGACAAACGAGGATAATCAGGATTAGCTAACTCATCCCTAGAACACATAACATGCCTAGGACATATAACAAAGTTATCCAGCCATATACCATTAAGGGTCATGGAACCATAA
**CoV-bait-269**	TTGCATGCGCAAAGCAGGATAGTCTAAACCATCCATACCAGCCATACACAAATGATGTAACCAACCAGCACCTGTTTCATTAACAAGAACATAGTCACAAGGCATGACAATGCCTATAAA
**CoV-bait-270**	TCACTAGTACGTTTACGAGTGTTCGCATCATACACAGATATAAGAGTGCCATTTACAGCATACATATAATGACCTGCCGTAACTGGACCCCTAAAAACAATGGCAGTCTTCCAATTGCCA
**CoV-bait-271**	AGGCAAGGGATCAACAGGTAATGAATTAAAACTATTTGTTGTTTCAACAACAAGAGAAGACAACAGTGGTACTAATTTTTTCCACGTGTTAGGCTTATTAACAAACAGTATAGGTTTACC
**CoV-bait-272**	ATGTCACTAACAACACCTACCACCATAGGACAACCACGCGAATTAACATAACTACCATATCTATTAGAATACCAGTTTTCAAAAGCACTAAATTTGTTAGCAAAGCACTGATCTGCAGAA
**CoV-bait-273**	TTAACAGCTCTCTGTTTAAGCTTACCAGGCATGATTTCATTATTCTGCAATTTGACAATTCTTTCACAGTTAAGGAACAGAGGCCATGCTAGATTTTCAACATTCTCTTGTGTAACTTCT

**CoV-bait-274**	TAGTTAATAGCATCATGCAACTTGTCACAATCAGACTCATCATAAAAGAAAAATGGCATAGCCTTTAATTTTGCAACACAGCGTTTATCAAATGCCAGTGTATGGAAAGCATGTTTGTTA
**CoV-bait-275**	TAAGCTCAGCCGCAATCGCAGAAAGATTTTCAGCACGAGCAGTTAGATCATTGACCTCTTGGGTCAAGTTGAGCACTGTTGAGTTGTAAATATCAAGATTTAAATCAGGTGTGGTGTGAT
**CoV-bait-276**	CAATCACGAGGTGCACCAATAGCAACTGCCAATTCCTGACTAGTCTTAAAACTGTCAGAAAGTGCCATATAAGTAGGTGCGTATGCGGGACTAAGAATGACCTCATTGCGTATGCAGTTC
**CoV-bait-277**	ATAAAGCATCATGAATTTAACATAGACACCGCCAAACAAAAACAACAAAAATGCTACAACCGAAGGGTAAACCTTAAGCGCAAATGGCACAGTTATGTAATCCCATTTAACATTAAGATG
**CoV-bait-278**	TTCAACTTCATCTCCTTCACTTGATGCACCAGAGTCAGACTCATCAGCGGTATTACTGATGTCATACTGAGAGATCATAATACCATCTGGGTTTTTGATGTCAAAGCCACCACAGGTATC
**CoV-bait-279**	ACACTGAAATGATTTAAAAGTAGGGTACTTATTTACACTAACTGTTAAATTATAGAAAAGCCGGCCTTTTTTCATAGCAGAAACACGTATAGAATGCTGTGGAATGGAGCCAGTTATAGG
**CoV-bait-280**	CTGCTTAACTAAACTACTATTAGTAAAAGGATAAAAGCCATCTGAAAAATTGCCAGTATTATACTGGCATGCTAACAAGCCTCTAGGTGACCCATCACACAAAATAACATCTTGTGCAGT
**CoV-bait-281**	TTTTATAACAATGAGTAACAAATACAACAATATTAGTAAAATTACAATGGGCTGTACAAAATTGGTTAGTAGACCATTGCATACCTTGTAAGGGTGCTGTCATAGCTACTGAAGATGCAG
**CoV-bait-282**	GTAAATATTCTGTCCTAATACTCATACTAAAGTTGGTGGGAATACTAATATTCCCAGTAACCGTGGGTGCAATCTTGACTTGGCCAGACTGAGATGGGACATAGCCAATACTGCCAGATT
**CoV-bait-283**	TAAAGCATTATCATAGCACTGCTGAACTTGAAAAAAAGGTTGTGAATAACACAAATCAGTCAAAAAACGAGACTGTTGAAAAATAGAATTAAGCAGTGCAGCAAACAAAGAATAATTAGG
**CoV-bait-284**	CCGCTACTATTACGCAATAACAACCTACCACTTTTGATCAAGCCTAAGACCATGTCATTAACATCACTATCTTTTAAAGTGACAACGACAGTGGCCTTATGCTTACACTCAAACTTACTC
**CoV-bait-285**	ATAACGCATGTCTTGTGCGATAGCGATCTGTTTACAGTCGGCTACATTTCGGACACTTCGGGTGAGGCGATAGGAGTGGTCGTGTGCAAATTGGTTGCGCTATTGGAGCTGTTCTCCGCA
**CoV-bait-286**	CTTCCAGCAAACATCAACATTCAAATTTCTTAAGAGCTTAAGCTCTGGGTGGTGCCTAACACTTCGCTTAGCAAATAGTTCGACGGCCACATTAGTAGGGAATGTCGTGTTATTTTTAAA
**CoV-bait-287**	CCGAGAGCGTTTGATGCCTTTATCAGCAATCAACATTCTTATGCGACCACACTGCCGCAATTGCACGGACGCACCTAAATGGTACGGATTCCATGGTCTTACGCGGAAATAGAATTATCC
**CoV-bait-288**	CTTCTTACATTTTGCTGCACACGACTCTTGGGCGTTGCAGCACTCACATTATCCTTTTCAACTTGCCCTTTCTTCTGCTTGCGCTGAAATTTTGGGCTCAACAATTGCGCACCCTCTTCT
**CoV-bait-289**	ATCCCCAGCGTTCTGGGAACAATCTGCTACAGCAATGGGAAGTCCATTATCATCATAAGACACAAGCAAATCCCGCTTACTTATATATGCTAGATACAAATTATTCTGAAAGGCCAAACA
**CoV-bait-290**	ATAAACACATGCAGACCTAGCTTGACCCGGGTCACATGCAACTCCAAGGATGACCCATGGTCGGCGCGTCAATGTTCGCTTCACCGCGCTTAACACTGGTTGTAGAGACGCTCTTCCGAT
**CoV-bait-291**	CATAAATTAATCTCATTATGCAGACCAACACAATAATTCCACTCTTTTGAATTAGATTCAACATGCATTTTAGACAAAAGACCGAGCAAAACAACATTCGTACACTTCATCTCTGTAAGT
**CoV-bait-292**	GTAATAAGGTACCAAATTAGCATAAGCATCACTAATATTAAAAGTGGGGTGCAGCTTGTAAATTGAAGCATAGCGTTCCTGATTGGCAATAGTGGGAGCTCTAAGCGGCTGTACATTGTG
**CoV-bait-293**	AAAGAATGGCATTTGTTTGAGCTTAGCAAAAGCTCGCTTATCAAAAGCTGGTGTGTGGAATGCATGGTTGTTCACATAAAGTGAACCACCATTACACCCTTCGAGGTTCAACTTAGAACG
**CoV-bait-294**	CTCTTGTACAGTCCAAACACATTACCATTAGCACAGAGTGGGAATGATAATTGTGGTTTGTGTTCAGTACAGTAATAATTAAGACCTCCCAAAAACAGTTTTGTAACATCATTAACAGTA
**CoV-bait-295**	CAAGGTTGGCAATAATTATATTTAAAACACAAATCCTCAAGATCTGTGACACCAAATTCTCTAGTAATAAGAGAATATTTATAGCTAGGCCTCCAAACTTCCATATATAAAGTTTAGATT

**CoV-bait-296**	AAAAGATAACATAATCATACTCAGAACCCTGAGAGGAGTCAACGGTTTGAGTGGAGAATCCTGCCATGGATGCCTTGACATTCATAGCATTGTAAGGTGATATAAAGGTCACGTTATCCC
**CoV-bait-297**	TTCATAATGTTTAGATTTAGATTAAATAATCTGAAGTGGGTGGTATAACTTGTTCACTATAAGACTTATACAACTGCATACCTCTTTTATAAACGTAAGCCGAAGGTGAAATAATAAAGA
**CoV-bait-298**	TTCGCTTTTGGCGAGGTTTTGTTAAAATTTTATGCCTGATTTCCTTGGCATTTTGCTTAGTGACTTGCTGAGGTTTAGAATCTTTACCAAGCTTGGCTAAAACAAGATTAGCGATCTCAT
**CoV-bait-299**	GTAACAACTGCTTTTGTTGACCATCAGCTGTTTTAAAAGAACGCCGGTTGTGTTTATACCAATATCCTTTTGCTTCAGAAGGGGGTATCCCGTAAGCAATGGGTACTCCTTGACCATCTG
**CoV-bait-300**	TGAGACCAGCAATCAAATACCAACTCAGAAGCCACCAAGGTGCAAGAGTACTATATGCCACAACATAGCTAACATACCATATCCATGAATATGTAACACCACGTGTAGCTAAAAAGTACA
**CoV-bait-301**	CAGGCATCTAAATCATCAGCACACACAGTTGCCTTCGCCTCAGCAATCCCTTCCCTGTCGCTTGCCTCTCCGACTTCGGTTTTCTCAGCAGAGGCGATGGGAGTTTGAGATCCGACAGCA
**CoV-bait-302**	AACTATGATCCTCAGGAGTAAAATCATTTGAAATGTGCTGAGTATCAGCACTTAAATTAAGAGAGTCAAACGATTCAGGTGATTGTTTAACACCTCTTTTACGCTGAGGTTTAGGACTCA
**CoV-bait-303**	TATTTGACAATGTATGGTACATTTGTTCCTGTGACATTAACTGTGAGGACACGATCTTGGGATGACTCATAGATACCTGTGTACTTGGCCTTGATAGCTGCGAACCATTGTGTGACTTGT
**CoV-bait-304**	AACATGGCATCCAAACCTTGCAGCTTTGACGCCATGTCACACTTTAAACAACGCCAGTTAGCATGAGCTTTAAAGCTACACTGACTTAAAAAGTAATAGGCAAAATCTGCCACATAACCA
**CoV-bait-305**	TTATTATTTGACTGCTTGAAAGCAAAATAATTGCCACAAACAACTACTGGCCACTTACACATGCCAAGCATATTATAGTACTTCAGCAGTTGTGGTTCATCAAAACCAAAAGCATCTTTA
**CoV-bait-306**	CTTTTTGAATGATGGCAACAAGTGGTTTAGAAGCTATAGGCAGTGCTTTACCATTAACCATGCGCAAAGTTGCACCAGGGTAAAACAGGTAATCAGCATAAGCCATAGCACCATTAGTAA
**CoV-bait-307**	TAGTTGATATGTAACAGGGTTCAATACTGTTACAACACGAACTAGGCACATAGGTTTCATGAGACCCTCCAAGAGGGTAAGTCAATTTAGTTCGTTTCTCTGCCATAACGGCCTGGAAAT
**CoV-bait-308**	TCTGGGCTAAACCCAAAATATAAGACATGTTAGCTGGTTCACATGGGAAGTTAGCAAATGAATTGTTAGAAGCCCATGCATTAAAATCGGTCTCAGATACTTCAGCTTGTGCTAACCATC
**CoV-bait-309**	GTCAGTTGGTGTTAGAACAGTGTGCAAAAACAGGATGCCATTGGGTGCAGCCTGTGTAATAGAAAATAAGTGTGAACCATTTCCGCAGAATCCAAATCTGTCAGATTGTGATTTCACACA
**CoV-bait-310**	GTATGGATTTAGTTGCTACATCAGCAACACCTACAACAACAGGACAATTATGACTATTAACATAATCTGCCTGCAAATAAGCATCAAAAGAAACAAACTTATTTGAAAAGCATGTATCTG
**CoV-bait-311**	TCAAAATTAAAGAAGTTGTGCAAAGTTTGGCTGTGCACGGTGCCCAAAACAATAGCCTGTGGAAACTCCCACTCTAAATGAGTGGGTTGCACAGGTAGTATTGTATGAACACAACAAGTC
**CoV-bait-312**	GCTACTTCTTCAAAGCTAGGGACTGGCCATAAGCAATTAACTAATAGAGGCTGTTTGGTAACTAATTTACCATTAACCAACGTGGAGCTATTAGTTAGGAGGAACCAATTATTAAAACTA
**CoV-bait-313**	GTTGCAGTGGACTTGTATATAACAGTATCGGAATCATAATCAGCCTTTTCAAAGGTGAATTCACCGACCTGATATTTGGAGTCCTTAGAAATCTGAAAACACGTGAAAACAGAGTTGCGG
**CoV-bait-314**	TCACTGCACGTGGACCTTTTCAAAAACTTCGTAAGGTTGAAGTCTAGGACCCCTACAACAACCTGAAAAACAAGCACAGCAGCAGCCGCAGCATCCACAACAACCCGTGGAAATGCAGCA
**CoV-bait-315**	GTGTGCGGCGTGCAGCCATCACCACAAGTTCTGCCTTCAGCTAAACAGTCGTAATTATACGGTTCATGCTTCCCGCTACAAATGCACATAACATTACGATCTTTCGCTATAATGTGATGA
**CoV-bait-316**	AATTAAGTGCTATAAGACAATAAACTACTATATAAACACCTACTGTATAACAAGTAAGGCGTTCCGACATAGTGAGACCAAAAACCCATTCATACAAACTTCTTAAATAGGTATTTGGAA
**CoV-bait-317**	GTAAATGTAGCGCAAAAGCTACCTTGGTTACGACACCAAGCGTAATTAGGAACTCTACAATAGCCTACTTTGGGAAATTGATAATTATATTTAAAACAAACATCTTCTAGTTCTGAGACA

**CoV-bait-318**	AAGCTGTTTATGCCAAACATCAGCGAGACCCAAACCTTCTCTCTGCTTTAGGTCACTACACTTGTACTGGACATAATCCAAATACAGTGCTTTACAAACAGCTTCTGTTTCTTTGACAAT
**CoV-bait-319**	GTGTAACAAACACTGTAATATCTGAAAAGTTACAGTGTGCAGTACAAAACTGGCTGCTAGACCAATCCATACCTGGTTGCGGTGCCGTCATAGCTATAGAAGAAGCATTAACAACACGAT
**CoV-bait-320**	GCGGGCATAATTTGATGAGTCGGCGGCAAGGCTGTCCATCACGTTACCAGCGGAGCGCGCCTCGTCTTGCCTAAGGCGACGTTATTCGTTTTGACAGGTGGCTATAGAACGCCCAGAAAT
**CoV-bait-321**	TGGGAATTCTCCTCCACTCTGGGATGTCTTTGAGGTCACGTTCTTTCGAAGTGGCTCTGGATTTGTTCTTCTTAGGTGTATTTTTGCCGCTGCTGTCAGACCTTTCCTGTTTGGGCTTCT
**CoV-bait-322**	AATACACAATCCACATAATAATGGCCACTATGGTAAAAACTATAGAAAAACCAAGATACACATTATTCAACGCGTATACGCAGTTGAAAATAGTTAAGATGATAGTAAGTGGCCACATAA
**CoV-bait-323**	AAATCACCTATAACAGCAAAAGCCGTTGGTAAGGAAATTAAAAGTATCAAAAACATGTTTAGATTATGGTCTAAGCATCATGCAGCCTAGTACCATTATCCACCATAAAATATAACAACA
**CoV-bait-324**	TACCTTAAAACCGCCTACGAAAACTGTTATGAAAACAGTAGTAGGCTTAATATTAATAAACCAACAAATGTGTGGTAATCCAATGTGCTCGACCTCAATGGGTCTTAAATCAGCCATAAA
**CoV-bait-325**	CTTTTGACATCAACAGCTGAGTTAAGATCATACTGTGAAATCATAACACCATCTGGATTATTAATGTCAAATCCGCCACAAGTGTCATAAATGAAGTAACCTTCAAGTTCTACACCCTGT
**CoV-bait-326**	TAGTGCCATTTTCATTAAAAGACAACATCATAGTTGTATGTTTTAAGTTACCCACTATATAAGCAGCTTGGAAGGCCTGATTTGTGGGTTTTAAAGTTGTCACAACCTTATAATTGGTAA
**CoV-bait-327**	ATTAAAGAGCAGTGTCTGGAACAGCTCTCAGGCGCAACCGGACACGGAATAACGGCGGCTAACATCCGCGCATTTGTCTACATCGCTATTTTACTAAACCTAAACGGTGATTGGATTACT
**CoV-bait-328**	GAATTCTTCAGTACAGGCACCTACTGTCACAAATATTTCTTTGAGAACTTCACAGTTCTTTTCATCAAAATTTCTGATGGCGTAACAAAGATCCATCATTGTGTACTTTGTAAGATTCCT
**CoV-bait-329**	GTGGCATTGTACACATTGTACACACAACCAGACAGCGTGTCAAAGGTTGTCACGCTATTTGTGATGTTGTCCTGCACTACAGTCCGCCAGAATAATGATGCTGGCATACTTACGGCTACT
**CoV-bait-330**	GAGGTGTAAACAAGATCACCATTTAAATATACGGATGTTAAATTATTAACACACTGAAAAGATTTAAAAGTAGGGTACTTAGCTACAATAAATGTTAAATTATAAAAAAGCTGGCCATTT
**CoV-bait-331**	GGGAGTAAATGTCATCAATAGAACTAGAAATAGCTTGGAAGTTGTGTTGCAGCTGTACGGTAAGTTGAGTCAAAGCTGCACCCTGCGAGTTAACAACCTCTTGAACCTTAGTAAGCGCAT
**CoV-bait-332**	GCGGTTTCATTTAAACCGCTATCAGCATTGGAGTCAACCGTATCTACGGCCCCCAAACTAGGGGTTTCAACTTGGTCCACAATTGACTCCTGCACCTCTACAGCCTCTGCCCAATCAATA
**CoV-bait-333**	CGACTGTACTCTCAAACTCAAAACCACTATGGTCCAAAGTGTGAAAAACACTAGCATTCTCAAAGCCGTAGAACGCATCCCAATCAACAGACGGTGTAGCAACACTAACAGGTTCATCAG
**CoV-bait-334**	CATCCACGTACATGTTGGATTGCATAATTCTTTGTACAAAACAAAGGATTATAACCATCAGGTACTACGTCTAATTTAAAACCCATCAAATTAACTACATCTTCAAATGTTGGATTAACA
**CoV-bait-335**	CAACATCATCAACAGCACTCGACCTATAACAATTGTCATAGACAGCGCGCTGTAATTTCTTAACATATAGATTGTTGCACACATTACTATCAACAGACAAAAGCCTATTAATGTTGGAGC
**CoV-bait-336**	CAGGAGCTAATGAAAGATAGATACAGGTTAAGTCCAACCCAGTGGTAATATTCAAGGTGGAATTAAACCCATATAAAATCCCTGTATCAATATTATAATAATACTGACTATCATCATAAT
**CoV-bait-337**	CGGGTTGCCTTACATTAATATGACTAAAAATCTATAGCAGCAAAATCCTAATAAAACTAAAAAAATTAATCGAAGCACCACTTTTAATAATCTAGTTTCTGCTAAACTCCTATATAAAAT
**CoV-bait-338**	AATCTATCCTTTTTAACTATATAGTTAAAAAGTTGGCGATATTTAATTACATTTTTAACCACTCCCGGCACTTTTGGTGTATAGGAATGGTAGTTAGGAATATCTGCTGGTTGCATATCA
**CoV-bait-339**	AGCAGCATTAATAGCAAAAACAAGAGATCTACCTACAAGAGCAACATATGCAGGCACATCAGGAATTGGTCTCATATTTTCAAATTCAAAAACAGTTCCTACAACAATTGGGCATGATTT
**CoV-bait-340**	GTATAATACCAACACCGGCGTGGCCGTAAATATTGTATTCTGTACACACATTCTGATACACTACCGACAAATCTTTCACACCAACATCTGGTGTTTGCACACCCAGATGGTCTAAGCCAT

**CoV-bait-341**	ATTCATCTGAGTAACAGTGGGTAGCACATTGCGCTTAGTGTATGCAAACAGCTCATCCTG GTCTTCGTAGGATAGAGATTCATAGTAAAGACGGGCCTTTCCAAACTTATTGAAAGGAAA
**CoV-bait-342**	TCATAATTCGTGCACATAGAAACTTCATCCAACACAACAATGTCAACATTAATCTCAGGGA TTGTGTTGACTGTAGCAAAGATGTACTGTGCTGAAGTGTTATTGACCTTAAAGCCAGAA
**CoV-bait-343**	TAGATATCTCTGCAAAGTTAGGACCATGCGAGGTAACATTCTTGAAAAATTCTTCCAATTC ATCCTTAAAATCCACATCAGTAGAATTCTCAAGGAGTGGAGGTGGTAAGTTATTCTCAA
**CoV-bait-344**	GTAATATTTGCGAAAAATTAAAACCACCAAAATATTTTAACGTCGGCGTTTTATACATTTG TTTTACTTGCGCAAATACTTCACGCGTATTACGATCTTGTTCCGCCGCTATACCCGATA
**CoV-bait-345**	AATGAGTAACAAACACTACAATATTAGAAAAATTGCAGTGAGCAGTACAAAATTGTTCTG TTGACCACGACATGCCTATACTGGGTGCTGTCATGGCTATAGAAGAAGCACTAAAATTCT
**CoV-bait-346**	GAAAGTGCAGACCTGTAAGTACTACTACAGCTGGAACCTACACAGCCTAGGAGACCAGT AAAATTATACGTTGTGTCGAAATGAGCATTTTCACTACGCACAACGTCAGCCGTAATTTCA
**CoV-bait-347**	TAATTGATAGAACCCTGACAGCTATCACACTCACCGTCATCATAAAAGAAAAACGGTATT GCCTTAAGTTTGGCAAATGCACGCTTATCAAATGCAGGAGTGTGAAATGCATGGTTATTG
**CoV-bait-348**	CTGGTAGTTGGTCCCTAGTCAAATTGACATAGGTGACCACACAACTCTCAATTTGAACAA AATCACTAACGGTAGGTTTTCTAGGTTCAAACATACGTCGCGATGAAACAAAATATTCCG
**CoV-bait-349**	TATCACCTTCAAACAACTGAGTTGAGATCTTAAGCTTTAACAACGCTGGTAAAACATCAA GAGCAGTAGCACCCAAGTACCATGCAACCAAAAACCATGGCGCGAGAGTAAAATAGGC AA
**CoV-bait-350**	AGTCTGGCACCCATTGCAAGTGCTTGTTCAATAGTTTGACATGCAGACACATATTGTGTTA ACAATTTGTTACATCTAGGGTTACCATTACAAACGTATCTTGCACAATCTATTGATACT
**CoV-bait-351**	TGCACGAACATCTTCCAAAGACGACCGCCATCAATGTTAAGTAACACATCGGGGCATTTA GGATTTAGATGTTCCAGCAAAGCTTTAGACTCGGATGCCTGCACAACAATGTCAGAGAG A
**CoV-bait-352**	ATCAAAGATAAAATAAAGGAAATTAAAACACCAACAGGCACCATTAACATAAATAAATCT TTAGAAAAATAATAAACTGCTGACATCATCAACCATTCACTTAAAAACATACAACAAAAT
**CoV-bait-353**	TCCACAGAAACTGTTAAATTATAAAATAAGTCACTAGGGCCAGTACCATTAATTTTCATAG CAGCAATACGAATATAGTTTTGTTGAATAAAACCTGTTAAAGGACAAAACTGCTGTCCG
**CoV-bait-354**	ACACATTGGGCGCTGGGGTCTCCGCAAAAAACCACGCAATTCCATCCTTACGATAGTCCA ACTGGAGAAATGAAGTCCAAGCCACACGACGAGTGGGTAGAGGTAACTTAGAAGTTAA CC
**CoV-bait-355**	ACTTAGTACAAAGTTCAACAAACTCATCATCATCAAAGTAGTAAGATGGTGCCTTAAAAG TGTGGGTACACTGTATATAACACCTGTCACCCTTAATAATGGTACTGTACTTTTTATAAA
**CoV-bait-356**	ACATCGCAACGGCCTACTTTACAGTATTCCATTGCCATTAACTTCACAATGTGCACACCTG CATCTATAACTTCTGGAAACCTAATGTAGCCAGCGGTCTCATAGAATGGGTACAGCACA
**CoV-bait-357**	TATATATATCATAATCCACACATTCATCCAAAGTAATATTATTATAAAAATGATTTACAGA AGTAGGCACATCTGTTCGTGTCTGCACACGAGACCCAGGTTGCCTAGTAATGATAACCA
**CoV-bait-358**	AACTTCTGGGGCAACATTAACAATAGTGTCAATAACATCAGTTACAACACTGTCTGAAAT AATAACATTATCAGTAGTACATGCATTGAGTTGTTCACCAATAGTCTGGTCTGCTGCGAC
**CoV-bait-359**	ATAGTGTGACTTCATCAAAAATTAGTGTACAACCTGCGTGAGCTGAAACTGCACCTGGTG TTCCTAGGAGAATTGCTTTACCGTTTTGATTGGATGTGTAGTTGTATGGGTTGTTGTTGA
**CoV-bait-360**	ACCCTTAAGTGGACGTACCTTAAGCTTACCAGGAATAACCTCATTATTCTGAAGCTTAACA ATCCTCTCACAATTAAGGATTAAAGGCCATGTCAAAGCAGTATTATTTGCAGTAATTTC
**CoV-bait-361**	GTTCATAGCACGCTTAAGTTGTTTGACGAGCTGAGGTGATGATCCATTATTAATAGCCTCT TCATAACTTTGACGAGCACTCTCGTAAGCTATAAACGAAGGCATATTAACAAATGTAGA
**CoV-bait-362**	TTTTCTAGGTCCTACAACATTAAGTACGCTGTATTTCTTACAACGTATCATGGCACTAGAA CCAACTTTGACTTTACCATTTGTCTTTACATACTTATTTGACAAACTCTGCAATTCATT
**CoV-bait-363**	CCACAAAGAAACTAGTAAAAAGGTAGCCCAATGAAGACGTTGCATAATGAATGGGTGCG AGATGAGACTCAACCACTTTCAAATGCTCTTTAGGCAAATTAAGATCAGAGTTGATACTG C

**CoV-bait-364**	ATAAGCAACGACATAACTAGCATGCCAAATCCATGCATAATTAAGATTTCTGACAGCCAAAAAGTAGAATGTTGCATACACAAACATGAATAACATGTTTTGTGTAAACACATAAGACAA
**CoV-bait-365**	ATTAAAATCTACATTCAAAGTAGAAAGCAATGTAGCATCAACAAGCTTAATTGGTTTGCAAAGCATCTGAGAAAAGTACACACACGCATTTCTTATTTGAGAAACGTTTGTACCAGTGTT
**CoV-bait-366**	ACTACAAGCTACACATGATGGTTTTTCACAACCAAAACAAACGTGCTTGATAAACATAAGAGCTTTGTACACAATAAATGTGACAACAATCTCATCACCAAAAATGTCAAAAGGAATGAT
**CoV-bait-367**	CAATTCCTAAGAACTGCTGTGCCTGGGGCAACACCCTTATCAGATCCAGCACCTAAATGAAGCACCCGCATCTTATGTGGAACACACATAGATGTGGTATTTAAGTACTGACACAACTGA
**CoV-bait-368**	ACAGATACAAACTTAACAATGGCATTGTTAAGAAGAACATAATCACCGACAGCCTTAAAAGACTGACCAGCAAATGTTACAGTATTAACTGTAAGGTCAAAAAGTGTTTCCACAAAGCTC
**CoV-bait-369**	CTTGTTGCAAGAAAGCAGGAAGGTCCTTGTTTGATTTGTGAACCTTCATTTTGTAAACATACATAATTTCAACTTCATCACCACGTTCTCTCGTTGAAACTTCGCCACCAAACAGCAATG
**CoV-bait-370**	ATGCTAAAAGCAAACATGGTCCAGTTGTTGCCAAAACTTGACCAACTGTCAAAAATTGACAGGGCAAGTACAAGAGGCCACAGCAGCCACAGAATTGTCATTTTCAGGATGTAAAGCACA
**CoV-bait-371**	AAGTTTTGGGTTGAATAAGGTCCTGGTGTACGGTCCACATTCCATGCTTTAACAAAAGTGGTGCCATTAATGATGACTTCATCAAGTTCACCAAAATAGTCTTTAAACTCCCATTCTTGT
**CoV-bait-372**	TCTTCTTTAAGAACATCCGTTTGCCCCACATTTTCAGGCTGTTCATCAGCAATTGAATACTGAGAAACCATAACAACTTTTGTAAAATCATTTCCACCTTCTTCATCATAGACAAAGTAC
**CoV-bait-373**	TCCTGAACAATATTAAACCGCTGAAGACGTCTTTGTACCTGCTCAACAGCTTCAAGTTGGTCTTGCTCAGTCATATCTGTCAAAAACTTCACATTATATGAGCCCGCCTGTGGTGAAAAC
**CoV-bait-374**	GGACATATTTACCCTTGTACTTACAATAACCATCCATAGTGTTATGAGGTACATGGGCTCTGCAGTAAAGACAAACAGATGCACCACCATATGAGTCTTGGTTAGAGTTTGCATCAACGG
**CoV-bait-375**	GTAGTACTGTTGAGATATTGACACAATTGAGTATATTTAACAACATTGAACATAATGCCACTAGGTAACTTCAAACCCGCACCGTAATTATACAAATTACATGGTTCTAAGCACATACGT
**CoV-bait-376**	GAAGGGGCTTATTCCAGGCATTTGGTGTTGGTGTATCTTCCTTTTTAAGTTGGAGAAGTTTGGGTTTTGTTGAACTACCAACCCAGGGAACAAAGGTCTTCAGTGATGTTTCTGCTGATG
**CoV-bait-377**	TAGTCTTCAACGGGGGACAATACTCAGTGGAACTATGGCTCGGAGGGGCCAATGGGCTTCTCTAAGCTTTTTTCTTGGCAGTCAGATGACACGGGGACGAATAAAAGCGAAGCCGTTTAT
**CoV-bait-378**	GTAAATAAAGTAGTAACATTAAGTTCACGATAAACAATAAATTTTTCCCTAACTAAACTAAAATTAGTAAAAGGATAAAACCCATCTGAAAAGTTGCCATTATTATATTGGCATGCTAGC
**CoV-bait-379**	TTTAAACTGCGGCTTAATATTAACCCTGTTCTTTGCGGTACTTGAGCTATAACTTTAACATCTCTAGTCCTAAAAGCCTTAAAAGCTAAACTAAACATGCTAAATAACCACACAAGAAAT
**CoV-bait-380**	GTCTGCTTGGTGCTCTCGAGTTGTCACGAGAGTTGTTCTGAGAGTTATTTTTAGATTGAGGCCTAGAGTTGTTTCTGCTCTGAGGCCTGCTGTTACTTTGTGATCTAGAAGGAGGGCGAG
**CoV-bait-381**	TGTTGAAAGTCACGTGCAAGCCAGACAATAGGAACATTTTCTTTGATAAGCACATTGTGATTGACAACCTTGGCATTAAACCGCATACAAGTTGCCACATCATGCGTAGACAATTGTTCT
**CoV-bait-382**	GTGACGCTGGGCATAATGTTGGGATCTATCCACTTAAAAGTGACAGTCACCATACTTTTAAGCTTAGGTGGGTCAGGTACACCTTCCATCACTGCGAGGACGAAACCTACGCAGGGCTAA
**CoV-bait-383**	AACACCATCTTTGCGAACGTAGTAATACCATGTAACAGGTTTATCTTCATGACTTGTGATGGGACAACCATTAAGATAGCCATAATTCAACTTTATAGCAGATAGTTTCTTAACAGCTGT
**CoV-bait-384**	AACTGGTAGTAAACTTCGGTGAAATAACCATGTATTACTACATAGTCTTTTACACCTGAATGCCAATCCTCAGAATAACCACCAATTTGGTAATCTTCCTTAAGTTTTGGTTGGGTAGTT
**CoV-bait-385**	TGCAGCGGTATTTGCTGTGGTGTAGTAGTGGGAGGTGTAGCAGTAGTAGCATTAGCTTCTTCTGCATACATAACGCCTAAGAAGGCGCAAAATAGAATAATGTACTTAATCATTCTCGTT
**CoV-bait-386**	GTCCTATTATCCTTACCTATAAACTCCTCATGTTCTGAGAAGTCTATAAGATAATGAGCAAGTGACTTATCATCAAAACTAACACCAACAGAAACGTTAATAAATGAGTGAGTCACATAT
**CoV-bait-387**	TGTGTACAGGTGTCAAAGGTAGAGACACACTCTTCGAGTGAACCCTTAACCACAAAACCTGCAACAGCTTGAACTTTAACCACACGCCAACATGCCACATCATCCACATTACAGATGTGT

**CoV-bait-388**	TGGATTTCCCAGAGTGCAGATGCATATGTAAAGGTGTTACCATCACAAGTGTTCTTGTAG GTACCATAATCAGGGACAACAACCATGAGTTTGGCTGCTGTAGTCAATGGTATGATGTTG
**CoV-bait-389**	ACATCTGTGCAGCAGCATTATCGGCCATTCTATTCAATTTACGCTGAACGGACAACTCCAA ATCAAATTCACTCTTTGCAATATTCATGGCCTTAAGAAGTTGTCTAACAACCTGGTCCG
**CoV-bait-390**	GTTAAACAAGGAGATGATTACGTGTACCTGCCTTACCCAGATCCATCAAGAATATTAGGC GCAGGCTGTTTTGTCGATGATATTGTCAAAACAGATGGTACACTTATGATTGAAAGGTTC
**CoV-bait-391**	CAAACCACGTCAAATAATCAATTATGACGCAGGTATCGTATTAATTGATCTGCATCAACTT AACGTAAAAACAACTTCAGACAATACAAATCAGCGACACTGAATACGGGGCAACCTCAT
**CoV-bait-392**	ACCACCACAAACTCATCAAATAACTCTTCAATAATAACCAATTTAATCATCTAAACCACTAT TAATATTAATTAAAACACCCTAACCTCAAAAAAATCTAAATTCCTCCTTACCATACTA
**CoV-bait-393**	TGACGCGCTTCAGGTGTGGGCCAGATCTCGCATGCGCGAATCGGGGGTCCGTGTCAATA ATGCCGAGGTTGTCTGGGAGGGCTTAGAGGCTCGGTCATGGGTTACCGTATCCTGCGTG TG
**CoV-bait-394**	CCCTCTTAATTACATCAAAAATTTTCTCACACACACAAACAAATCAATATACACTCTTTCCA AACAACTTAATTACATCAAATCAAAAAAAAATATCTACTACTACCATAACCATAAACA
**CoV-bait-395**	GGGCATAGAGCATCCTGCGTATGAACGGTCTGAGACCTGAACAAGGATGCTGAAAAAG CCATAAGTTCGTTAATGCGCTACGCACATTGGTATAAGTAAGGTATGAATTTAAATTCATT A
**CoV-bait-396**	GTGTGAGATGTGGTTATATATACTTTCTCCAGTTTTGAGGTGAAGTCAGTTTACTCAGGG GTTGTTGAAAAATGAAACAAAGGGTCTGATTCTCGTTCTTTGAATTTAGATTCGAGAGCC
**CoV-bait-397**	ATTAATGCTTGAACCATTATCAACCTGAACATTACCCTTGCAAAAGATTTTAAAACACTGC TTGCTATCTGGGTGCACAGGAATGAATTGGTTTTCATAGACCATCTCAGACACAGTACG
**CoV-bait-398**	ATACCCAAAGGGACTTGTACAAATTTACCACGTAATTTACATATACCATCTACATCTGGAT GCTCTACACGTGCACGGCAATAAATACAAACTGAGGCACCACCATAAGAATCTTGGTTA
**CoV-bait-399**	TGATTTGAAGTATAAACATGATAGCCAGGCACATAGATTTCAGAACCTAAAATACACAAT TTATCAACATCCCATGGATAGGAATTATTACACTTTAATTTAAAAAATGTGTATTTATGG
**CoV-bait-400**	GAAAAGGTCATAGTATGAACTTCCATATATAACAATAGCCAAACCAACCTTCTCTTGAGG ATCCCAAACATTATTATCTGAATTAAACTCACGTACATCAAAATAAACACCATGAACATT
**CoV-bait-401**	AGACCCGAGTCTTAATAATGTAAATTAACATATTGCATGACGATCCGGGATCTGATCTAT TAACGTAGGATCACAATAGCAAAGGGGTATTGATATGATTTGATGCATGCACCTCATCTG
**CoV-bait-402**	AAGTGACACCATAAATTTTATGACCTTTATGTGCAAATTGATACTGAAATTGTTTGTCAAC AAGACAGTCTTTAGGTGCAACAGGAGGGTCACGTGGTAAGGTCGGGACTTGGACGGAA G
**CoV-bait-403**	ATATAATACCAGTACGTTGGGGTACACCAGCTAGAACTTTAACATCCCTCTTACGAAAGG CCCTAAAACAGAGACCCAAAATACTAAGGATGTACAAAAACACTGTAATTAAATTACGTG
**CoV-bait-404**	CATAGCAGGGTCTGCTGCATACACAAGTAGTTCACGCAATGACAAACGTTGAGCATGTT GACGAATATCTTGATTCATCACCACACCAAGCTCCCTATAATGATAACCAGTTGAAACTAC
**CoV-bait-405**	TTTTGTGTAATCATCAATTGCTTTTGCAACACCACCACCATGACTCAAGTTCTCATTTGCCG CATTGACAACAAAATCATAATCCAGATTAACGAGTTCAGAAAACTCTGCTCTGTAAAA
**CoV-bait-406**	AAAAATAAATACCAAGAGAAGCATTGTTCTTGGAGTTACCGACACCAATGGCAAATTCTC TACCATTTTTGTAGTAACTCACAAAAATAGCCTTAGCACCATGAGTAATATTAGCCACAC
**CoV-bait-407**	CAACAAAGTTAACACCTGTTGAAAACCCTATTTGCAAGGGTAGGTTTGTACCAACATTAG GGCCACAGGCATGAGTGCCTTCAACGTCAAAGCCAATCCAGCCACGCACTTCGCGTATTG
**CoV-bait-408**	TGTGACTGTGATTGCATTCACTTCCGGGTTCCATGCCCAAAAAGTTCGAGCACGTCGGAA AAGTCTGAAACTATTTACGAAATACATCACCCACATAATGAGTGTTGATACGGCCATAAA
**CoV-bait-409**	GCCAGTGTGAAGGTAACCGCCGCATCCTTTACATACACAAATTTATGGCCTAAAAACTTA AATGGTTCACCTTTCTTAAGTGCTGCCACAAGTTCAACCACACTAAACGATGAGTAGATT
**CoV-bait-410**	TGTCTACTGCACTGAGTGGAGTTTCAAGATTGGTGTTTCTCTTGTGGAAGGCTGAACACA TTTTGAGGCATTGGTCAGATTTGACGCGCAGTTTGATGGTAACTGTTAATGTGCCTTCTG

**CoV-bait-411**	ACGCGATACGCATGAGTGGAAGAACACAGGAGTATGAGGGGTAAGATTAAGAGCATCATGATGAGTTAGTGCTAAGTGTCTTGTAGAAAGATTGAGGCTGAAGCGAAGTGGGCTGCTGTT
**CoV-bait-412**	ATGGACCCTGTTGTTAACTGGGTGGAGCTTAGGCCACCGTGGATGCGGCCCAACTTACTTATCCAGAGCACGGTCTTGCTCGCTTCACCAGGAAACTTCCAGTGTTCGGTAGACGAGGGA
**CoV-bait-413**	ATCAACATATGCAGCCTGCTCTGAAAAAGAACATGGCGTAACAGAATAAACAGCACCACTAGTGACATTCTTAAAAGCTAACAACTGTCCAGAATCAGATGTATAATAAACACCTGCCAA
**CoV-bait-414**	AGTAGTACAGTCGTACCTCTAACACACTCCTGATAGTGATATAGCTCGCAAGATGTAAATACAATCAATGTCAGGAAGAGAATAATTTTCATGTTCGTTTTATGGATAATCTAACTCCAT
**CoV-bait-415**	TATATAACGCTAAATAACGATTATATTGCGTTAACGGTAATAACGTTTCCGAACGTAATTTTAAATACATTTCTTTATTTAATAAAAACGTACATAACGCCGCTTCTTCAAACGTCGAAA
**CoV-bait-416**	CTATTGTAGATACATGTGCTGGGGCTTCTCTTTTGTAGTCCCAGATTACAGTATTAGCAGCGATATCAACACCCAAATTATTGAGTATCTTAATCTCTGGCACTGGTTTAATGTTACGCT
**CoV-bait-417**	CCTTGGAGTTGGATCTTGACTGATTAGCACTTTTACTCTGACTACGAGAGTTAGAGCGTGAAGTGGGTCTGCTGTCAGAAGTCACAACTTCAAGACTATCAGGCAAAGTAAAGTCAAAAA
**CoV-bait-418**	TCATTAACGTCCATACACGACGCGCCGCATCATCATATACCGTACGCGCCGTCATTAATATTAATAATACTAACGCCGACGCATACATTACACAATCTTTTAAACGATAACCCGATAACG
**CoV-bait-419**	ACCAATCAACGCGCTTAACAAAGCACTCATGGACTGCTAAACATCTAGTCATGATAGCATCACAACTAGCCACATGTGCATTTCCATGTACCTGGCAATGTTGGTCATGGTTACTCTGAA
**CoV-bait-420**	TACCTATGGGCACTTGCACAAATTTACCGCGCAACTTGCACAATCCATCTACATCTGGATGTTCAATACGCGCGCGGCAATATATGCAGACAGATGCACCACCATAAGAATCCTGATTAG
**CoV-bait-421**	TACAGGTTAGCTAACGAGTGTGCGCAAGTATTAAGTGAGATGGTCATGTGTGGCGGCTCACTATATGTTAAACCAGGTGGAACATCATCCGGTGATGCTACAACTGCTTATGCTAATAGT
**CoV-bait-422**	ATACAGTATAGGGACATATGCAAAGAGGGTTAAAAGCTTTGACACTAGTATAGCAACTCTACCACACCGCGACTTCGCTTTAAACAAAGCAAAGTAAACAGCAAAGAGGCTCATAAGTGT
**CoV-bait-423**	TGTAAGACCTCCTAAGGCAATGCCACCAATTAAGGAACCTGTGTACATGGCCATTCGTTCAGCATCAGCAACGCCAGGCAAAACCATAATGCCATTATAATATTGAGCACAAGCCAAATC
**CoV-bait-424**	GACCAATACAGCAATGGTACATAAACGAAGAGGATTAAAATTTTAAACACTATAAGAGCTACTCTTCCTCGATTAGATCTTGCTTTGAAAAAAAGCAAAGTAAACAACAAAGAGGCTCAT
**CoV-bait-425**	TCCACAGAAACCATAACGGTTAGACTGGGATTTGACACATTCATTCACTTTTTGTTGTGCAAGCTGTCTGGAAGCACGCACCTCAGTATACTTAGTAAGTGTCTGAGCAACGAATGCGTT
**CoV-bait-426**	TTAAGTCATCATAATCAATATCAAAATCAAGGTTAGGAAATGGACCGTCCTCAAAATCATCAGGGTGAAAGTTTACATAGTCACCTTCACCCCCCTGTTGATGGTGTTGGTCATGGGCAA
**CoV-bait-427**	CGTCGCAATTGAGCTATAAAATTCACCCTGTGTAATAACAGAGTCAATAGTATACCCAACTACATCCTCCTGGGGGAACCAACTAGGTGCATACCAAGGTTTTTGTTCTGCTTGTGCAGT
**CoV-bait-428**	TATAGAAGCCGCACTTTGATTATAAACATCCTTAATAACACCAACAGTGCACTCACTTGCAGAACCGGCATTATTTGTATAATTAGTAGAGTTCACTACTGCATAAGCACCCCCTTGTAA
**CoV-bait-429**	GCGTAAACAAGACTAAGCCAGGCTCACGTAGAGTCAAGGCAATTTCATCGTTAACGCATAAGCCAGCGATGGCAATAACATCTACAAAGTCACCCGGTACAAGTACTGTATGTAAAAACA
**CoV-bait-430**	GTCTGTGCCATCGTGGTTGAAGCGGGCGTGTTGAAGGACTGGGAGACCTTTTGCTAGCAATTGTTTGGCGACATAGATTTCATGGGCTGTTCCTGTGTCTTTGAGCATGATGGTACCGTT
**CoV-bait-431**	CCTTAGCAACCCAGAACACACCTTGAGTGCGTTTGCGGAAAGGTTCTTCAGCATGGGGACCAGTTCCAAGATAGTAGAAGTGCCACTTGGAAGGTAGTGTCTCCTTGTTGCCCTTTCTAG

**CoV-bait-432**	TCCTCATAGGAAAGTGCCTCATAGTACAAACCAGCTTTGCCAAACTTATTGAGAGGGTAACCAGCACTCTTATTAAGGTTGGTAACAACTACATCTCTAGCAGCAATACAACCACCTTCA
**CoV-bait-433**	TCATCAAGCAAAAGGTCTACAACTGTGCAAACCTGTTTGTAAGAACCATTGTCTGCCAAAACAAAATAGTTTTGCATGACATCAACATCTGAATTAGTTACGGATTTCGCGTCTAATTTA
**CoV-bait-434**	CAGCATAACTCTTAAGTTTTTCAGGTGAAATTGTATTTATTAGACGTTCATAAGACCGCATGTCAAGCACAAAAGTACCTGCCGCTGCGCTCTCAAAAGTACCAATAAATTTATCACCTT
**CoV-bait-435**	TTGGCAACAAAAATGTTGTCAAAAACACTACAGTATGCTTTATAGTAATGGTTACAATAAAAGCTGTACAACCAGCAATCATTGGTATCATTACATACATATTAACAGGTAAAATATGTA
**CoV-bait-436**	CCACTAGCTTGTAAAGTAGGACTCTGCTCATACATCTGAGCATAAAATGCTTCCTGCACAAAATTATTTACATAAGACATATCAGTCATAGTTTGAAAAGTATCTAAGATACCAGACTGC
**CoV-bait-437**	CTGTGAGTTCTTCTTCTTCTTTACGGTCAAGGAAGTCTTGTTTCTCTTGTTCTTTTAATTCTAACCATGTTGAGTAGTTGGAGCAGATTTCTGGGTCTGTGTTAAAAATTACTGGATAGG
**CoV-bait-438**	TATGTTGTTTACAAACAACAGAACCTTCATTAGTTATAATAGTAGATGGATACTTACGTCCCTGAACTATTGTTTCAACAGTTATAGTAGGTGAAGTACGTATCCTAGAACAAATAGTAC
**CoV-bait-439**	CAACATCACCTGTAAAAGTAGTGTAAGAAACACCATCCAACAAGGCACTCTTAGGCACAGGATCAGGTATAGAAACACTAACTACTATACCAATTCCATCAACAGCAGAAACCCTACTCT
**CoV-bait-440**	CATTAGGTTGTGGTTTAGTAGTAACAGCAAACCCAGAACCTATAGGCGCTATCATCTGAATAACACCTTGTATAGGTTTACCACCATCAGCTACATGCTTAAGATAGGCTTCTTTAGGAT
**CoV-bait-441**	TCACCGAAGTCATAAAAGTCTCCGTTAAGGTCTTGATTGTCAAGTGTTAGCACACCAACAACACCGGCCTCTACCATCGCATCACAGAGCTTGACGCACTTAAGCAATGCATTAGCGACA
**CoV-bait-442**	TTACCTCCTCTGTAATAACAAACTTCAAAGGGGTCAAATAATTCTGTGTTTAATACACTTGAAATATGCTGTAAATTAAAGCTGATTTGTTGTATTACTACTTCACCAGTTTCAATTATT
**CoV-bait-443**	ATGCATTAGCAATCATTTTCTGATTTTGGCTCAGTACATCCATAGTTACACCGAGTCCATTTATACGATACTGGACATTTAAATAAAAAGGAATACCTGCAGCCGCAGTCCATGGTGGAA
**CoV-bait-444**	TCCACATTGCAATTCCAAAACAGGCACAAGCCATCCAACTGGCCATGAGTAATATAATCATACTCTAACATTTTAACATTGGTATTTGTAGGATTTTTATCATAACAATACCAACTGGCG
**CoV-bait-445**	AAAAATTCAACTCCTACACTAAATTTCACCTCAATCCATTCAAAAATAAACCTAAATTTTTCAACAATAATACCCAAAAAATTAAACAACCACTAAAAAATCTATTATACAAAACCACCA
**CoV-bait-446**	GGCATTAACAAAAGTAGTGGCACAGCTATTAATTCGCACGAAATCTGCGCTAGTCGCGACACGCGGCTCATACATAGTACGTGGTGTCAAGTAGAATTTATCATCATGAGTTTTAAACAA
**CoV-bait-447**	CCACGCGCCAACATCTTCTTCCATGGCATAATATTTAGTAGTGAAATGCGTAGTTTCAATACACATCATACTACTACTGTCATTAAAACCAGCCAGAGTGACATTGACACTAGCAGGATA
**CoV-bait-448**	GGTTAGTAATGTTGTGTAACCGCATACATCCACTGTCAGTTTCTTGGGTGAGTTCCTATGGACCTCAAACTCTGACCGGTAACGGCCGGAGAAAAGAATGACTGAACAGGAGTCTGCCGA
**CoV-bait-449**	GCATTCAACAGAGTGTCCGGCCACGGCTCCAACACCACCAAGAAGACTTCCTTGCTGGATAAAAGCCCTCTGCGCCATTTGGGGGGCGATTGCAATCTGTGGGCCTATGTGTCGACCTGG
**CoV-bait-450**	AGGGTATCTAGGTTGATTGGGTGAAATTTGGTCTGTTCTGATTGTCTGATGAGTGGTGTATCAGCTTCAATACAGTTGTATACTTCTGTTCTTGTGTACATACTACAGCAGTCTGAGAAT
**CoV-bait-451**	GTAGTCGTTGGCAATGTTAAGTTATAAAATAGGCGTGGTACACCACCCTGTTTCATAGCAGAGACACGAATATATCCCTGTTGTATTAAACCAGTTAAAGGACAACTGCCTGCACCGCTT
**CoV-bait-452**	TCTTTACACATACAAGCAAAATCTTCATTTTCAAACTTATGATAGTTCTTTCCATCACCAACAGACGAATCTTCACCTGCCACAAACTTATTATAAGCAGCAGATATTATTATTGGCTTA
**CoV-bait-453**	ACATAGGCCATATAGTTTTGATGGTCAGATGCATCAACCATGTCTGGTTGGTATTTTAGGTATCCAAGACTGTCTGTGAGTTGTGGGATTTCATTTGCGAGTGGTGTTGTTGTTTTGGTG
**CoV-bait-454**	ATGCATCTATAGCTAGTGACACATATCGCTCCATCATAAGAGTACCATCAGTCTTCAGTAAATCATCCACAAAACAACCTGCTCCCAATATTCTAGAAGGGTCGGGGTATGGCAAGTAAA
**CoV-bait-455**	CGACCACTTTTAAAGAAGTACGTATAATCCTCATTAACATTCAAAAGCTGCAATTGGACTAAAGCTGATGCAACCCAACAATTGTTATCACATTGTCTTAGTACCTTAATACCATTAAGG

**CoV-bait-456**	CAGCAGTGTCAACAACTTTAAGCTGTGCAACACTACGTTCAACGCGCTTAACCTTAACAGGCTTGGTAGGTCCAAGTAGACACTGTGTGTAGGTCATAGTCTTAAGACCAGCTTCTTTAA
**CoV-bait-457**	AACAATGTGCAACATCGCCCAAAGAACCTAAAAATGCACCAGTGGCCTCGTAACATTGCTTAACAAGATTATGTGTGCCACCAACTCTATAAAGTTCCATAGCTGGGCTGTCAAAACCTA
**CoV-bait-458**	CAATTTGCTGCATTAACTACAAAGTCATGAGACAAACCAGCAAGATCTTTAAAATCACCCTGGTAGAACTTCACATTACAGTACTCGGTAAAAGGTGCCACAGTAGAAGGCTTAATATCA
**CoV-bait-459**	ACCAAACTCAAAGTGCAACCTAAGATTCTTACAGAAATTAACAGCGTCACCAATGCCATTGATAACAGTACGCCACAAACGACCACCATCAACAGCTCGTACAATCTCAGGACCTAAATA
**CoV-bait-460**	GTACGACAAAGTCAACACCATTAGAGAACCCTACTTGTAATGGAACATTTGTACCGATATTATCACCGCAAACATGAGCCCCCTCTACGTCCATACCAATCCAGCCTCTAACGTTACGCA
**CoV-bait-461**	CCTAGACCTAAGATAGGCTCAACACTATTGCAATCATAAAACTTATGAGAACCAATAGTGTAAAATGGCGCAACAACATCAGTTGTTATTTCAACCACAGGTGTTTCAAGCACTTCTTTA
**CoV-bait-462**	GCTCTAAAACCAAGACCTTGAACAACAACATATGGTAACCGAACATAATTATCACCACTCAAACGGTAATATGAATCATGTTGCAAATCACCATAAGTCATAGCACCCTCTATAAGACCA
**CoV-bait-463**	TGATAGGTGACTTCAACAAAATCACCAGCTTCTTTGGTGGTAATTTCACTACCAAAGGCTAATGCAGCCTGAGTAGGAAGCAGCTCCGCAATTGTTGGAAAATGCTTAGCTTCAACACCA
**CoV-bait-464**	TCTAGTATTGACTATAGACTTAAGAACCTTCTGGTGAAACTGTCTATTAGTCATAGTAGAAAGAATTGACACACCTGCCACGGTACGCGCTCTATTTTTCGCGGATATGGCATATTTAAG
**CoV-bait-465**	TCCCAAGATAGCCTCAAAGTCAGTAAGTGATATTCCGGGCTTTATAGCCTGCACACCGGGCGGAGCCTTAGTGGTGAACAGGTTATAATTAGCCAAGTAGGTGGTGTTATTGGGATGCCC
**CoV-bait-466**	CTGACCAAAAATAATCTTTGCATATGCCTCGTGGTCTGGACACACAACACACAATCTAGTAGCAGAAGTGGCAGGGATAATAGAAAGTGGTACAACTCCATTCTTAGCCAAATTTAGTAT
**CoV-bait-467**	CACAGCCAACACTAGTCTGCGCAAAAGGAATCGCAGCACTTCTCATTAAACTATTTGTACTACGTGAGAACAAAGTAGGCATAGTCTTTAACTGTTCACAAGGAACACCAGTAAAGATGG
**CoV-bait-468**	CCTTAAAAATAGGGTCAATCTCAGTAACTGTAACATTGTCATCAAAGGCAACATTCTTACCACCAGCCTTTTTCCTAAACCGCAATGGCAAAACTTTACCATCACTAGCATAGGGATATA
**CoV-bait-469**	TGGTTGATAGACATTGTGCACAGTTATCGGTTTCAATAGTGACGGAACACTATATCCACACACCACACTGACAGAACAGCGTGGGGCAACTAAGGACACAAGTACGTGACGGAACACGTC
**CoV-bait-470**	ACTCTGCAGCTTAACAATGGCATTGTCAAACAGCAAGTAATCAAAAGCAGCCCTAACAGCCTTACCTGCAATAGTGACAGTGTTAGCACAGGCATCAAAGATTCCTTCCAAAGCAGACTT
**CoV-bait-471**	TCAGGAGGTTGATACCAACGTAAAGCATGTTGAGGTCTGTTGTAAGGTAGTGATGAATGCTTGTGTATTGGTCAACTTGGAATGTGTATGCTGTGTATGTGTGTGCTGCGATTACTGTTA
**CoV-bait-472**	AACTCTAGATTACACGCCAGCGCACAATACGAATCCGTGCACTGCGGGCCCCCGATGGCAGTCGCGCGGTAGATGCCCGGCAGCGCACAAGGAAGTGTATGGGAAGTGGTACATGTATGG
**CoV-bait-473**	GTTTAAAATAGTATTAAAATCTTTGTCAAGCTCATAAAAAACTTTAATAGTCTTAGGTGTGCTTGCAATCTCATTTACTGTAGGCTGTTCCTTAAATGTAACACGCCTTCCAGCACAAGG
**CoV-bait-474**	CACAAGGAAGCTCCTTACTAAAGAACTTCATAGCACCATTCTCTATATAACGAAAATCATACTCCGGCACACTATCAAAGGTGGCAGGCTTAAAATTAATACTAGACACCCAAACAGCAC
**CoV-bait-475**	CATCTATTGCAAGACTTACAAATCGTTCTATTAAAAGAACACTATCAGTCTTTAACAAATCATCTACAAAACATCCAGCTCCTAATATACGACTAGGATCAGGGTATGGAAGATAGACAT
**CoV-bait-476**	TCGTCTCCAAAAGGTATTCTGAGTCTCCTGAATCACCCTGCTTCAAAGTTAGAGCTGCATAACAGAAGTTTACGAAAGGCATAACGTCATCTTTCTTAAAACCATCCCATAATTCATGGT
**CoV-bait-477**	CTCATATAAAAAGGGGTATTATGGGATGTGCAACACAACACACCAAACCACAAAAACAGCACATAGCATATCAATTTAAACTTTATATTATATGTTAAGCCCATAAGGTCAGTTTTAAAT

**CoV-bait-478**	TCCAACACAAATATGGTAAAGTGAGCTCCACCGCAAAAGGCAAAATGCGTTTTACACCAACGTTCTTCAAAAGTTGGCGGTGTTCGCCGAGGGGTGCTGTTTGCTCGAGTTAGACCACTC
**CoV-bait-479**	ACTGTAATATATGGAGTGATTTCCTTGACCTCGTTTTCACCGAAGGTCACGGTTTTAACCTTGTCAGGGTTGCCACCTTTGAGTTTAAAAACTCCTTTTGTGGCTTGACAGTCAGTAGTA
**CoV-bait-480**	TTGTTATTAATGTGGCTACAACATGCATCGAAAATAGTCTTGTCGTTGACAACAACATAGACTGTCTTCTTACATGTAGAAAGTAAGGCAGTAAGACTATCAATAGGCTTCATACCAAAT
**CoV-bait-481**	TTTAGTTTTACTCGAGGTAGCAGTCTGCGAAACACTGTCACTAATTACCAAGATATGTGAATACAACGACATTTACTGAGATCCTTGCGCGGCCCCCATTCCTGCGGCATAACCAATCGC
**CoV-bait-482**	TCCTGGCTACTATACTTCTTATGCTTTACATCATAGTCATATGCCGTGAATTCTTCACCCACATAGAACCTATAGAAACCATCAGAACATTCAACCTTTGTGACTTCCTGGTATGATTTG
**CoV-bait-483**	ATCTGTGGCTCACCTTTCATCGATTCATCAACATCTGCCGAATCGTCGACGGGGTCCTCACACACGTCTTCGACACTCTCATCATCACTAGAAGCAGCAGCAGGCCACTGAGAAATCATG
**CoV-bait-484**	TAAAGACATTATATGTACGCCATTTATATAAACAGCACCATAACGTGAAATAACAAACTCCTTTGTGACAGGTGGTAAAATACCCACAAACAAATTTTTTGTAGAATTAATTGCATCAAT
**CoV-bait-485**	TATGGCATCATCTGGTAACCATCTTCTTAATACAGTACTACCAGGAGCAACACCAGATGCACCAGCAGCTCCTAAATGCAATACACGCATTTTGTGTGGTACACACAATGTAGTAGTGTT
**CoV-bait-486**	AGATACATCGTCGATTGCCTTTGCTATGCCACCACCATGAGACAAATTCTCATTGGCGGCATTAACAATGAAATCAAACTTCAAATCTAGCAACTGACTAAGATCACCCTTATAGAACTT
**CoV-bait-487**	TGGATCTCAGCAGGGCCTGATGTCGTCCATTTCCTAGGAAAGCCTGACAGACAGACAGCCCCACCACTGTTCTAGGGCTCCCTGGCTACCAGAGGAGAGACGTCCCTCCATTGTCTGTAA
**CoV-bait-488**	CACGATAGGTAACCGTCTGACACTCTGGGATAGCCCCATACCGTAAAAACTTTGGCCGCTATCTACTTATCTAGTTTAACCGAGAAGCGCTTCGTAACGAGAATAGGTCAAGCACGATGC
**CoV-bait-489**	CAAGCTGTCGTTCACTCTCGTGGGTAGCCTGGCTAGTGCTATCGGCTTTTACGAGTTTTTCGACATGATCTGGGACCAATCTCTACATTCGCGTGCGACTAGAAAAGTTAATGCTCTGC
**CoV-bait-490**	CGCGAACGCGAACTAGGAACAGCATCTCCCCCATACACACAAAGTAGGCTCTTTCTTTTCGGGATTCCGGAAGCACATGATGGCGGATGCTGAAAGTGAGTCCATTCAAGTGGTCGGTGA
**CoV-bait-491**	CCTTACCTATAAAAGCAGCCGCACAAAGAGGTTTTACAACCAGAGAATCCAAGGAGAGAGCAGTAGTATCTCGACAAAAGATGCCGGTGCCAACAATACTTTTAAAAGTGTGCATCAAAA
**CoV-bait-492**	TCATCCAACAACAAGTCAACAACAGTGCAGACTTGCTTGTAGGAACCATTGTCCGCAAGAACAAAATAGTTTTGCATGACTTCACAATCTGAATCTGTTACAGATTTGGCAGACAGTTTA
**CoV-bait-493**	AGAATAATAGTAAAAACTAGGTGTGACAATATAATTATTAAGGCCTAGTAATGGACTATTAACTGTGGTCATAGCACCTACTATATTATTATTTATAATAGCAGCTTGAGCACTAAGTTC
**CoV-bait-494**	GTACAAGTCGTTAGTTGAGAGATAAGATAGAATTTTAAACACTATAAGAGCAGCTCTGCCACGGTGTGATCTTGCTTTAAATAAAGCAAAATACACAACAAAGAGGCTCATTAGTGTATA
**CoV-bait-495**	ACTGGTTACATACAATAAAGGTACAGTATTAAATCTAACAACAAATAAATTAGTCTTACACTCTATTAAACCACTCTGACTCAACTTAGTTAAACCTTTACCACTAATACAAAACTCTAA
**CoV-bait-496**	CGCGGACGCTCGTAGGAATAAAGGATCTATGCAAGGCTCATGGGTATATACTTCAACCAGAAATTGACGCGCATATCACTTCCGTATTCCATCTACTACAAGCGTGATGCTGCGTACTTC
**CoV-bait-497**	TGTTTTCACCACAAACGTGCGCACCCTCAACATCCATACCGAGCCAGCCACGTACATGACGCATGGCAAAAGCTCTGGTACAAAACATATTATGATAGTCAGGTACGTTGTAATCAAAAC
**CoV-bait-498**	CCAGCTCCTTTATCTCCAAGCACCTCCTTTATCGGAGCAGCAAGGACGCCATCAGCGTCAAATCCATATTGATCTGTTAGAAAGGTGTTTCCTCCACGCTTAGCCTTGGGCTTCGTGGGA
**CoV-bait-499**	ATAAAAGGGTAGTATAAGCTCTTTAGGACTTTGACATTCTTCCACACAATCCTCCTTCGCGAGCACAGGTGTTTAGACGCTCCTGTCGCATCTGTCGATTAACATTTTCCTCAGAGACTC
**CoV-bait-500**	TGGCATCTGCATGAGGTCCAGTACCTAAGTAGTAGAAGAACCACCTTTCAGGAAGCTCTTTACGTTGGCCCTTCAACATGCGATAGCGAGTTTGTCTATTCCAATAACCAATCTGTTGAT
**CoV-bait-501**	CAACAATGGGCTTCGTGACTAGTGCTACTGGCTTTCCATCTACAATACGTATGGTGGCACCTGGATAAAGCAAATAGTCAGAATAGATCATGGCTCCATTGCTAGGTTTAGCTTGGGCCA

**CoV-bait-502**	ATTTTGTCCCATTCAGATATCATAAACCGTGGAGGATGACCGAGTGACGTCGAGATCCAC AAACCAGATTTGAGGGTTCTAGTCGTTCACTTGGTGTCGGTTGATTAGTTTGGCCTGGCA
**CoV-bait-503**	CCATTTTTACAGGTGTTATCAGTTTGATTAGTTTGATTAAAGCAACCTGTAAAAAGTGGCT CATAATAACCAGATTGTGTATATACACCATCTGGCGGTCTCATAACATCTGGACTATAA
**CoV-bait-504**	CACGAATGATTGGCGGGAAAACTGAAAGAAACTTAACAGTGTGATTAGTGCCGCTACTA GTGTTAGTGAAGCAATAGTAAGGCAGGTCTTCCTGCAAGTTGTTTGACCTAGTGGGCTGT G
**CoV-bait-505**	ATGCTGTTTGATTCTGGGCTATTTCTTCATCGACATAAGGTCCATAAAATGTGCCCTCAAG ATCTGTACCACTATGAGTCTTGTTATTAAACTCAATGTGGTGCATATAGTGTAAGCAGA
**CoV-bait-506**	CACTATTGACGACACCTGGTAGCACCATAATGCCATTATGGTATTGAGCACACACCAAGT CTGCTATGTCCAAACCTTTAGTACAACGCTTATAATCCTCATCAACAGTACCAAGTCCTG
**CoV-bait-507**	CAGTAGAAAATATACTCGCTACAGCCGGTAACATCAAAAATGGAGCTAATGCCATGAAG ATTGTCTTTGTTGAATAAAACAATCCCAGGCATGTTTTCCGTAAAGTTGCTGCCGATGGCA
**CoV-bait-508**	CAGAGGAACCACAAATAAAGGTAAATAGTGCAAAAAGCCACTATCTGCACTGTAGGCTA AACGGGAGCTTAGCGAACTCCAGGCCTTTATGGCACCGGTGACTAAGTGGTCCAGTTCA AC
**CoV-bait-509**	TTTAGTGCTGTCCACGCAAATGCCAGCTGAAGCTGTGACACGCGCGAATTTAGTGGGCA CAAGAACTGCATGCATAAACAAAATGCCATTAGGCGCAGCTTGTGTTAACGAAAAAAGG TG
**CoV-bait-510**	CCTTATATATTCTATAAGAGTCCAGATTAATGTTAGCATCAGCAACTGCTTGCACAACTGT AGTGTTCTCAAGCAGGTTGTCCAAATAATTGTTAACGTGCTGAGCTTCCTCTTCATCTG
**CoV-bait-511**	TAGAAATGTTGATTAAAGTGTCCCGGTTTGACAGACTGTTTAGTGAGCCCACTAGACATA GTTGCCAAAGTCTGACACGGTGTGCGTAAATCTAAACACTTGTCAGAGACAGCTGCTATT
**CoV-bait-512**	ACTACGAGGTTGAAAATCTGAAATGACACGTCCTTGAGTGAAATAACCATCAGGGTATTC AACAAAGGCACCATCCTTTCGAGTGTAAATGTACCAAGTAAATGGTTTATCTTCATGTGT
**CoV-bait-513**	AAACAGAATAAGTGATACCACCAGGCCCTGGTGTTGTTACATTAAGATTGTAATAGATAG TACTGTTAATATATTGCATAGCACAACTATCCGCAGCACTACACCACGACACCACACGTG
**CoV-bait-514**	TGCACTTCCATTGTGCTGTCTGATTTGGTAAAAGACCAGCTAAAACAACTTGAGATGGTG GTTGTATATCAAACTTAGAAAATAACCTAGTAAGGTTGACAGTTTTGTTACAGCGCTCAT
**CoV-bait-515**	GGTACAAAACAAGGTCCAGTACTCAAACTTCTGAATGAGTTCATAAAGCTTTTTATTCCA GCTGAACTCAGTTATTTTAATAGCAACTGTTCCACCCAGCGCTAATTTTTCGGTGATAAC
**CoV-bait-516**	CCTCCTTCACAGTCTCTACGACTACGGGATATTCTTCGGGTACGCCTTCAATAGGTGCATT ATCTAGAGATAGCTTCTTCACAATAGTGTAGCGCACACCCTTATAAATAAGAGTGTTCC
**CoV-bait-517**	CAGGTGCCACATCAAAGTTATGATAGGTGGCCACTACTTTAGCTTTGGCGTTATAGCCGT TAATAGTTTTCTTGCCATTAGTATACCAGCAATGGTCTTGCGCATATTTAATGGCAGGAA
**CoV-bait-518**	CCTACCATAAATATTATAATTAACACACTTACCAACAACAATATTATTAGTAAAATTAGTA GTATAAACAGGTGGTTTTTCTGCAGTTATTATGCGTGAACCATCAGTCTTGTCTATAAA
**CoV-bait-519**	CCTTACGCATATCAGCAGAATAACCTACCATAAAGTCGTGTCTAAGGGTGAACTGATTAG AAGTGACACACACCACAGTGCCGTTATTAACTAAGGTGCCATTAGCAGTGAACTTTGGTG
**CoV-bait-520**	GTCACTGGTGTTTGTTTTTCTATCCTATCATGGACAAAAACACCAGATCCTTCTACACTTGC AAATCTACACATGTTGGTTTGTTTACAGTCACCACAGACACCATATTTAAACTCATTC
**CoV-bait-521**	AAATATTATCCACGTAACTAGCACATTGATCAGTGCAATTGGAGATTACAGTAGTGCTGT TTTTACTATTCACTCTATAATAAGTGATATCATAAACAGGGTTAAACCACCACAGGTCTA
**CoV-bait-522**	TCTACATTACAATTCCAAAACAGACAAAGACCTGGCATCTGTCCATGAGTAATGTAATCA TACTCAAGTGTCTTAACATTGGAATTTGTTGGATTCTTGTCAAAACAAAACCACTTAGCA
**CoV-bait-523**	AAAATGCATGAGCAACAGCCAACTTTGTAACCTGAACACACTTATCCAATTGATTATTCAA CTTACAATAATGAGTTGCTAAAGTTCCAGGTAGAACACACTTAGGTGCAACAAAACCTT
**CoV-bait-524**	ATTTTTGAAAGAAAACTGTCAAGACGAGTCGATTGTCTAGTGTTCATGTAGATTGTAACT ATGAATACCAAGACAATGACAACTGCTACACACACTATGAAGATTATGGCCCATGTTGGT
**CoV-bait-525**	TCTGATGAAATTGTCTTGTTGTCATAGTGGCCAATAAAGAAACACCTCCTACAGTACGAG CACGTTCCTTACCAGATATGGCGTATTTGAGATTTAGCTGAGTCATAGTTGGGAGAATAT

**CoV-bait-526**	ATCATCGCTCTCAACAGTGTAACCACGATTGAGAACGGCGTATGCATAATAGGGCAGTGGCTTATAATCAAAAGCAACATCATTATCATCATCAATCAGCAGAGGTCTAGCTCCAACATG
**CoV-bait-527**	ACCTAATTTTAGGCGATACATACCAAGATTGCCATTGGAATTACAACCAATGTTAGAGTTTACAAAGTTAATGCAAAGAGCAATGCACAAAATGAGAAACATTTTATTTAGTTGAAACTA
**CoV-bait-528**	TAGCAACCAGTAGCAAGACTACTGGGCGAAACTTGGTAACAGGAAAACTCACTCACCTGGAAAAGGTTAAGTAGTTTTGTTAGGTTATAATTGCAATTGGTAAAAACCATCCTATTGAAA
**CoV-bait-529**	AAATAGACGACTTAATGTGCCAGGACTGCTATGTCCATCAGAAAAGATATACTGCTTACCAAGGTCATTAGCCTTGGGATACAGTCCCGTATAAGTCAGCGTAATATTAGAGTACGACTT
**CoV-bait-530**	TACATCAGCCCAAGTATTCCCATCCAGAGAGTATACTACGACATTGCAGTCTGCACTATATCTCTGCTTAGGATAATAGACATCGGTCATCTTGCACTTCATATCGGAAGTCTTACTCAA
**CoV-bait-531**	GATTTTCTTAATGGGTTTAAATGTGGATTGAGATATGACAGTGGTTCCAGTTTTATTGGCAGCAGCACCAATACGGACCACAAAGCCATCATCAAAGGACTCCACTTGTGAGGAGTAATT
**CoV-bait-532**	TTGAAGTCAGGTAGGTCCGACACTTCTCAAGTGAGTCAAATGGTAAGGCAATTTGTCCTTTTACCACATAAAACTTATTATCTGCATAGCAAATGGTTTGATCTGACCAATTTAGCAGTT
**CoV-bait-533**	AACTTCCTGCTAGCCTGAACCTCAGTATACTTAGTGAGGGTTTGAGAAACAAAAGCATTAAGTGCTGATAATCTGCCGGTGATGAGACGGTCAACCTGAACATCGGCTGAAAGAATGTCC
**CoV-bait-534**	ACAATCCGACCCGTACCCCTGTTATTATAATAGCAGGGGGTAGGGACATGACACTTAATATCTGACCAGCTATTACAACATGAATCTCTTAGTTTCTGAGGCATCAATTCAATGTTATGC
**CoV-bait-535**	TATGGACTTAACTGTAACATTGTTACAGGTGTTTGAATCTACTCCTAAAAGCTTGTTAACATTAGCAGAAACAGCTTGAAAGATGTTAAAAGCCGAGTTAGCATACGCCGTAGTACCATC
**CoV-bait-536**	AGTATAGTGTTCCATCATAATAGAAAGCAAAGCCATCAACAATAGCAATACTACCATTGAGTGCTGGTGGTTTAAATGTCGTCTCTTCTAATTCCACAAAAGAAGTTTCAATGACGCGTG
**CoV-bait-537**	ACACTGTAAAATCAGTAAAATTACAGTGGGCCGTACAAAATTGATTAGTAGACCATGACATACCATTAGGTGGTGCAGTCATGGCTATAGAGGAAGCAGTAAGATTCTTACTAAAAACAA
**CoV-bait-538**	CGAAGTAATCACCAACACAAAGTTTGTAGGTTGTTGATGACTTGTACACAACCGAGTCGCCATAATCGGACTTCTCGAAAGTGTACTCGCCAATTTGAGTTTTACTGTTCTTAGTAATCC
**CoV-bait-539**	GGAGGAACCTATCCCGCAATAGCACTATTGCGCATAGAAGAGGGTCCTGATTGCGGAGTACCTACAGATAGACAGGACCACGGGAGTATGGGCAACCTTTACGTAGACATTCGGCTTGGG
**CoV-bait-540**	AGAAAGATTCATTGCAGGCGCCTAAAGTCACAAGTATTTCTTTAAGAACTTCACAGTTCTTTTCATCAAAGTGTAAGGGTACTACCCTACCGCTGACGGCCCATAAGGGAGTATCGGGGA
**CoV-bait-541**	ATTGTTGGATGTTATGGCACCAACAATTGTATTAGATACAACTACAAGTTGCGCAGGTGAGTCACAAGGAGTCACTTGAAAAGACCTACCTGATGTTGTATTTTTGAATGCAAATAATTC
**CoV-bait-542**	ATGAGGACTGATTCAGAATGTTGTGTGACACAAAGTGAATGGACATCAAAAGCGGAAGCGAGGGTGATTAGTCCTGCTGATGGTGTTTGAATCGTACAATGCGCAGCGTTTGCAACTTTG
**CoV-bait-543**	TTGCGCCGTCTTTAGCAACCCAGAAAACACCCTCAGTACGAGTCCTATAACGGAGGTCGGCGTGAGGTCCTGTTCCGAGGTAGTAGAAATGCCAATTGGAAGGTTGCTCAATTCGCTCAC
**CoV-bait-544**	GTGAGAAGCGCGTCAGTTTCAGGATTGAAAGACCACCAAGAATGTGTCCTGCGCCACAACCGAATGCTATTGACAAAATACATTATCCACAGCATAAGAGTGATGCAAGCCATAAGGATG
**CoV-bait-545**	GTTGACTAAACACTGGCAAATCCTGAGGCTCCACACTCTCTACCTCATGTTGTTTAATAATTGGGTCTAAACTTTTTGAAAAAACCTGTTGAGAGTCAGATGTGGTTACACCATCTTGTT
**CoV-bait-546**	AATTAAGTGAGAAAAAGGTAGAGTTGTGTAATGCAGTGGTATAGTTAAACTGCTCACTACAAACAAACACATAATTTTCACCACGTTGGCCAACGACTTCAACAGAGATGTGGTCAGTGA

**CoV-bait-547**	TATTATTTTCAGTTGAGTTACTGGATGTGATAGCACCGACAACGGTGTTATTTATCACAATCAGTTGAGAAGGGGTTTCACACGGTAAGACCTGAAGGGTTTTGCCAGTGGTTATATTTT
**CoV-bait-548**	GTCGTTACTTCAACAACTCCCACAACGACTGGACAGTCAGGTGAATTAGTGTAAGGTGCTTGAATGAAGCTATCAAATGCCAAAAACTTATTTGCAAAGCACTTGTCATTTGAGCGAACA
**CoV-bait-549**	AAAGTGGAGTTAGACAAACTAGAAATAACACCCACTATATCATCATCAACATATGCAGCCTGCTCTGAAAAAGAACATGGCGTAACAGAATAAACAGCACCACTAGTGACATTCTTAAAA
**CoV-bait-550**	CTGCCAACATAATATAATTGCGCCTCAAAGAAGACGCTTTAAACAGTGCAAAGAAGTAAACAAAAACACTGGTGAAAAGAAAACCCGTCACATTTGAAGCTTGTCTAATTGGAACTACAT
**CoV-bait-551**	CTAACGAGCAAAGAAATAACTTGCTGTGGACAACCCATCTCACCAACACGACGGTTAGTGGATGCGTAGATCCAAGCAACAAATCTGTTTGGATTACCATTGAGATACTCCCTATAGAGA
**CoV-bait-552**	AAGTCGAAATTGCTTGAAAATTGTTTGACAGCTGTGCAGTCAGGTGTGATAATGCCTCACCCTGCTGGTTAACAACTGTTTGAATCTTTTTAATAGCAATAGCTACGGTGTTAAGAGCCT
**CoV-bait-553**	TGACGCTGGGCATAATATTGGGATCTATCCACTTAAAAGTGACAGTCACCATACTTTTAAGCTTAGGTGGGTCAGGCACACCTTCCATCACTGCACGGTCAGACCCGAGCAGGGCGCACA
**CoV-bait-554**	TAGTTGTGATGCTATCATGACTAGATGTTTAGCAGTCCATGAGTGCTTTGTTAAGCGCGTTGATTGGTCTGTTGAATACCCTATTATAGGAGATGAACTGAGGGTTAATTCTGCTTGCAG
**CoV-bait-555**	CACCGCTGTCCGCCATAAAATAAAACAACAAAAACACAATAATCAACACAGCTATACCCAATAATACACCCAGCAATATGACCGGTAATGGGTCATACATGCAAACGGGTGTTATAACTC
**CoV-bait-556**	ATAACCTAAATCCGCATAATCTTTGTTGTAGCACACAACTCCATCATCAGATAAAATCATCATAGAAAAGTGTTTTCTCAAATAACTAAAGTACTCAACAACAAATTCTTCATCAATGCT
**CoV-bait-557**	ACAAAAGGTCCCAGGCAATGATCTATAATAATCATTATTAAGCACCCAAGAACCATTAAAATTAAAGCATATACCCTCATCAGCTTCCTCACATAATCCAACTCTGCAATACGACATAGA
**CoV-bait-558**	TGCTTCAAATTTTTAAGATACAAACAGTCAGTGATTTTACCACTGACTGTAGTAATCTTTTTAACATTAGAAGCATCAAAAAGTTGATAACTTGAATCACATCTCAAATGAGTATAATGA
**CoV-bait-559**	AGAGCCCTATCTTCTTCCATATAAAAATGATCCATAATGTCAACTAACTTAGGTAGCCGAAGGATAATAGAATCAGCAGCATAAACATGTACTGGCTCTTGTATAACAACAGAACCATCA
**CoV-bait-560**	TACCACCACTAATGGGACCAGTAAAACGCACAATCTCCCACTGTAATCCATCCAACTGCATCTCAAACACAGTAGGCGGAAATATGACCCTAACAAGTCCCTCATACCATGTTACACCAA
**CoV-bait-561**	AGACGACGATCTCGTCACCAAAGACATCAAAAGGCACAAGCTGCAAAAACCAAATGGACTGTTGTAGGCCCAAAAACACACCAAGTATGTTAAGATAATGGAAAATAAAATAGAGAGTAA
**CoV-bait-562**	CAGATGTAAACATACCAGGAATTGGTGTCGAACTGTTAGTTAGATAAAACCAATTATCAAACTGAAAGTTTGGTGGTATGGCACCATTCTCAGTCACAGCAAAAACATATTTAGGAAAGC
**CoV-bait-563**	TCGGTACGATTGTGTGCATTATACACACAACCAGATTCCGTATCAAATATTGTGTCATTCTCAGATGAACGTACACGCATCCAATAGTCAGAAGCAGGTATGGACACAGCAACAGCACGT
**CoV-bait-564**	GCAGTAATCCAACGACCGTACACATCTGGTGCTATAATAACTATAGCAATGCGGTCTTGCTCAAAAGTATTGCCAAAAACCACACCAAATGGTTGCTGACAATATAAACCTTCAATCTGA
**CoV-bait-565**	GTTATAAATATCACTGATAGAACTACTAATGGCTTGGAAATTATTTTGCAATTGCACAGTCAGGTGGCTTAAAGCTTGACCTTGTGTGTTAACGACATCTTGCACTTTTGCCAATGCTTT
**CoV-bait-566**	CCAACCTTATTAAATAATAAAGAAACACTACCAGCCAACTATTACCCAAGGGTATAGGCAAGTATAACACAACAAGGTGTAGTACTGCCAACACACCAACTATTGGCAAAGCCGGTGTGT
**CoV-bait-567**	ATCCTTCCTTAAATTCATTACAGGCGTACCTTTAGACCTTAAATTGAATTTCTGCATATCATACAAACTGTATGATGATAACTGCATAATAGTACTATTACGCCAAAATATATAGTTAGC
**CoV-bait-568**	TTATCAACAAGTATAGCGTCAGTTGGCAACCATTGTTTAAGAACTGACGTACCCGGACAAACACCTTTGTCACTACCGGCACCAAAGTGTATAACACGCATCTTGGCTGGTACAGCTAAA
**CoV-bait-569**	GAATCAGTTTGTCGGCCACCTGGTTGAATAACAACGGCCGGCCAGCTATCAGCGATAATATAGTCACTAGACATATTAACGTAAAAAGCATGCACACAACCATAGAAGTCTCTTGGCAGA

**CoV-bait-570**	TGAGTGACACTACCCTTGTAGTAACACTTATAACACTCACCTGTAGTAACCTTATTAGCCTTTAGTTTCTTATCATAAACTAAAGCGCTAACTGTTTCAACTATTTCCTTAGGACACCTG
**CoV-bait-571**	GCACAATACGACTACACCTATTAATAGGTAAATTCTTATGCGCCTTCTCACACAATGCGTCTACAGCAGCATGACTACATGCTGTGTAAACAATCTTTGCAGACGGATAATAGAGCGCAA
**CoV-bait-572**	AATTTATAAGTGGTACTAGAACGATAGTTAACTACGTCACCGTAGTCACCCTTTTCAAAAATGTATTCACCCAGTTGCACCTTACTATTCTTAGTAATATGAAACCCCGTGAATACATAA
**CoV-bait-573**	CATTACCAGTGCTATTTTCCATGGCTTCCATAACAAAATAAAAGGCATGTATATTATTAAAATACTGAAAGGCAGTAGTTCGAGCTGTTCTAGAGCAGTTGTACCACACCTCTGTAGGGT
**CoV-bait-574**	TAGTACCACTTGAAGATGCAACGTTCGCTGTAAAATTGAGGTTAAATCTAACAACATCAAAACTACCATTAACAGTAGCACCGTTACACTGACTAGGAACGGTGTCAAAGTTGATAATGG
**CoV-bait-575**	ATCCATGGTAAAATGATGGTGACTCATAATAATAACCAAATGGATGACACACACTACTCATTTCAAAACATCCACCGTCTATATAACAAAAAGGCACAAGATGTTTTTACAAGAAACAC
**CoV-bait-576**	GTATCCATTTGGCACGGCATGCTTTAGTAACATCATCGAATTGGGATTAGCTAGGTGAAAACTTTTTCGGCGTCAACGCAGTAAGGTGAGTGCAGTGGTCTATTTTCCCCAATGTAGCCG
**CoV-bait-577**	TTAAAGCTCGGACGTCATATATAAAAAAACTGAGGATGGAAGACTTTAATTTACGCTCTAGTGGAGAGGCCCTAATGATGGGCCTTCGTAGCTGTATCGAGGGAGCGTCTTGAACGCAAG
**CoV-bait-578**	CAGATTGTCGGTGTTATTTCAGCAACTCCTACACCTGATGTGGCTTTTAATTATACCATAGCTACTCCTATGTTTTATTATTCGTCGGATAGCACTGCCAATTGTACAGAGCCATCAATT
**CoV-bait-579**	TCAATCTTCAAATAACCTATTCTAGCAAGTCTAATTTGTGAAATGAGCAGGTGCAACCCGCCTATGGTGGATTTAGAAAAATCACCATAAACAACATGCTCAAAACCATAATCTTGTAGA
**CoV-bait-580**	TAGTGACATTCTTAAAGGCTAACAACTGTCCAGAATCAGATGTGTAATAAACACCTGCCAAAAAGCTAGAATTTGTAAGGGTAATGATACCCTCACCTTTAAAGCCATAGATAGTATACT
**CoV-bait-581**	AGCAAATCCCTAACACAAAGGGCTACCTCAGCATCGATATCCTCAGTGGGCACGCTCTCAGCTGCAACTTGAACTTGAGTTTGTTGTACCACAGATTCTTGCACCTCTACAGCCTCAGCC
**CoV-bait-582**	GCCAGTTCTTCAAATGGCTGTGTAGGCACTCGTTTTCTGGGTGAATTTAAAGGTTTTAGTGAGAAACGGGGCAACGTCTCCCACTGTTGACCATAACGCTGTCCGTTAGTAAAATAAACA
**CoV-bait-583**	CACTATCCGCACAATCGGCAGCGTTAGCAACGCTACCATTATTAAAATAAGACACTAAAAGGTCCCTATTAATTAAATGTGCAACATGCAAATTTACGTCAAAATTAGTACACATACCAT
**CoV-bait-584**	GCACCTTCATCTTTGGGCATTGGCTTGTTATCTGACCAATCCTTACCGTCACAAAAAACCTTACCAATTTGAGCTTTATAGCTCTGTGTACCAGAAACTAAAACAGTATTATAATTAATA
**CoV-bait-585**	GTAAGCTCCACAGGGGCGTCAGCCTTACCTTCTAAAACCTCCACCTCCATCTTCTGCTGGCGTGGTGTTAATAAAACGCCCAGTGCCTTAGCGCGAATCTGCAACTCAGAGACAACACCA
**CoV-bait-586**	CTCAGTAATGTACAAGTTACACTTATAATTACCATTGTAACGATGTCTGTACCAATAATGTCAACCCTACCCGGGTTAACATAATTGTACAAACTTAACAAATAACCCTGCACACCCTCA
**CoV-bait-587**	GTGGCTCTATTAAGAGCACCTGCCACACCACCGCCATGATGTAAATTAACATTGGCGGCATTAACAAGCACTGCATACGAATAATTACGTGCTTCCTGCACTATATCAGCGCACTTAATG
**CoV-bait-588**	GCATACATACCGTGTGTAGCGGTACGTGATGTGTCATACTGCATAATTGTACGCACCATAACAAGTAAGGTACGCAAATTACCAACGGTAACTTCCAATTCTAAAGCTGCACGCAGCAGC
**CoV-bait-589**	CCAGCTACCTCTATTGTCAGGTGCTATCACCACAATAGCTATATGATCCTGTTCAAATGTGGTCCCAAAGGCCACACCAAACGGGTCTTGACAGTACATATTAGACGGTTGATTCAAATA
**CoV-bait-590**	GGGGGACGCGTCACGGGCTGTAGGAGGATGCCATGTACTGACCTAGTAACCTCATTTGACCTTTCGGAAAGTCAATCTTCCTGAAGACTCACCCGGCGGAGTCCCCCTACCCTCAGACTC
**CoV-bait-591**	CAGGCACACCACTGATTCCAAGTAGTAGTCAACAGTCTTGCTTACCCCGCGTGGGTATGACCCCTCTTGTTCATTGGCGGAGGTCCTCAGCACTTCAGCAAGATGGGCATTTTCATTGCC
**CoV-bait-592**	TCATAATACAGACGGGCTTTACCAAACTTATTGAATGGAAAACCTGCTGACTTATCATAATTAGCTACAACAACCTGACTTGCCTGTAGGCAGCCGCCATCATAGCAATCAAAGTATTTA
**CoV-bait-593**	TTTGCTGGGCCAATCTTGACAAAATACCTCATGGTTGTAAGTTCAAGACCACCTGCCCAAAGCACAAACACCACGTTATCAGACAGACCAGCCAAATAATCAGCAATCATCTGCACTATA

**CoV-bait-594**	AGGTCGGTGGACCCCTAACTCGTATTCCCAAAGGGCGACACCTATACAGTACGAAGGGCAACTGTCTTAAAACAGGATAGCGTTTTCGACCAGCTGGGTGCTGGTGGTGACGGGCACCTA
**CoV-bait-595**	GAAAAATGTTTACGCAAGTAACCATAATACTCAACGACGAACTGGTCATCGACGGTAGTTGATCTATAACAGCACTCATAAAGCTTACGCTGCAATTGCTTAACTTCTAAATTATGACAG
**CoV-bait-596**	ACTACTAAGTGAACCAGTGTAACCCCACTGCTGTATATCAATACAGTAAGGGTTATACAGGTAATCACAACCAAATGCATGAGTAAAACAATAGAAAGAGTGTGACACACTGTTATAGCA
**CoV-bait-597**	ATCATTTTCTGGTTTTCACTAAGAACATTCATAGTGACACCAAGACCATTAATTCTATATTGAACACTTAAAGCAAATGGCACACCTGCAGCTGCAGACCATGGAGGGAACATAGCAGAT
**CoV-bait-598**	CAGCATTTGACGTAACGACATGAGTAGGAACATCATATTGTAGGCTGGTGGGGTGGTGGGGATTTACTATGTAACCTGGTTGAACCTTATAAACTGTACCCAAAATATTAGAACAAGTGA
**CoV-bait-599**	TGATTGTTAATAAATTCTTGATAGTTAGGCACCTCCTGTATAAAGGTTATAACACCATTGGCTACTGCAAATAAGCCATTTACCAGCTGGTAGCAAAGCCATCCAGCGGCACTAGCAGCA
**CoV-bait-600**	ACTATTAGTTAGAAGGAACCAATTATTAAAACTAAAATCTGATGGTATAAAGCCTCCTGGCTGTGTAGTAAAAACATTAGCCACATAACTAGCACATTGATCAGTGCAATTGGAAACTAC
**CoV-bait-601**	CACTTGTATGACACACATATGGTGTTATAGCTAAAATAAATTTGTGGTTGGTACCACAAACATGGTCATAAGCACACTTAGTACACAACAGAGGTCTACGTATACAATCACCACATCGCA
**CoV-bait-602**	CACCAGATGATTGGGCTTTAACTTCGCTAGAGTCATAAACTGTACAACACAATGGACAAGAAAAACCATCCATCATATTACCTGAAACCCAACCATAAAAATCACAATTATCATTAAAAC
**CoV-bait-603**	TGCATTACAAGTAACACAGAACGCAACCAACAGTTAGTATGTGTAATAGTAGGTGACCATAAACCATTAACTTTAAAATATGTTTCTGTTTCAACACTAGAACCATAACTAACAAACAAA
**CoV-bait-604**	CCAACACGTCACTGGCAGAGAGATGAAAAGTGTGGAAAGTAAAATACAAGATTATGATTAAACCACACTCACAAGCATGGATAACAGGGAATCTTGTTTAAAAAGTTTAGATTTAATGAG
**CoV-bait-605**	GTCTACAAAAAGTACGGGTTTAATACCACGATATTGTCTAAGTAAAGCATAAGCTTTTTGTGAATATTTACCTTTAGGCTCAGCATGAACCTCAGCATAAGCATCTTCTACACTAAAATC
**CoV-bait-606**	CAAAGACGGAATCTCCATAACAACAGGTTTCTCGTTAAAATTAACTTTTCTACCTGCACATGGAAAACGCCAAGCCTGATCTAAAAGACAAATATTTTTATCATTCATGACAATAGGGAA
**CoV-bait-607**	GGGGTGCAAAAAGCACAAAAATTGTCATCAAATAATGAAAAATGTAATGTACAAGGTAAGTCCGCTGCTATTAGAGTCATATTATGTCCACACTTACAAACATGAGACACAATATCTCCA
**CoV-bait-608**	TATAATGGCCTACACCTACACCCATAAACATGTTAGCTGCAACAACATCATCAGGTAAATCCTTACTCAGAGGAGTATTAGAACAAATCAAAAATGGTACATTCAATTTAGTACAATGGA
**CoV-bait-609**	GCCAGCCTGTAGCAGGTGCACTAGCATCTAACATGGTATTAAACCAATTAACCATTTTATTAACAAGTGTAGTACCCAATAACTTAAATTGAGTAACCAACCTATTAACATAATGCTCTA
**CoV-bait-610**	TATCAGGTGCTGTCATGGCTACAGAAGATGCACTAAAATTTTTACTCCAATAGATAGACCCTGCAGTACATTGACCTGAGCCTGCGTTGCTATATTCTACAGAAACATTAACTACTGCAT
**CoV-bait-611**	GAAGAGTACGTCTCGCGAGGATTTCGCCGCAGACTTCTTCGCTAAGCACCGTATCGAATACGAGTCCCACGTGCATCCGGGGGTCTGGTCCCTTGCTCCGCCAGAAAAATGGGCTCCCCT
**CoV-bait-612**	TTTGGTGGTATTGTCTATTTGTCATTGTAGAACATATAGAAACACCAGCAACTGTACGCGCTCTACTCTTAGCTGATATAGCATATTTTAAATTCATTTGAGTAATCGTAGGCAATATGT
**CoV-bait-613**	TAGCTATAGAAGAAGCATTAACAACACGACCACCATGAATAATACCAACAGTACACCCAGATGAAGAGCCTGCATTATTAGATTCACTAGAAATATTAACAACCGCATACGCACCCCCAT
**CoV-bait-614**	AACCCATCACTGTTCTCATTTCTGTGGATAAAGATTAAGTACAGACGGAACCCCGGAAGTGCGTCCCGGCAACCTAATGGTTTCCCAATAATTGCTTAAAACCAATAGACGCAGATTTAA
**CoV-bait-615**	TAAGCACGTGCTAACAAAACATAAGATTGTCTTCCATCTTGTCTAGCATCAGGGCCTACAATATTAAGAATTGTTTTACATAATTTACCACCGGTAGAAACATAACAATCTCCTACTTGG
**CoV-bait-616**	TTACCCTGACTACATATATAAATAGAATTAGGACACTCAAAATACTTAACAGTTTTAACACCGTTAATAACATCAAATTTATTGATAAGACGCCAATCTTTAGGAAGACTAGTCATCTTA

**CoV-bait-617**	GGCCATACTGAACTGAAGACAAGAAAGACTCTGCACGCAACTTAGCATACATGTCCTTATTAATCATGAAGGTAGATGTCGCGGCTTCAACAAAGCTCCCGAAGGATGTGTCACCAACAA
**CoV-bait-618**	CTAGAGTTGAACCTTCATTGTTTTGGATGCGAGGTTTTAAGTTCACATGAAAACGTTCTTCATTCTGATGATCTATGCAGATTACCATACCATTGCAACTAACAATTTCGCTGTCCATGT
**CoV-bait-619**	AGTCAAAAGTCTCATAAACTTTTTGACTAACATGCAAATGCTTATACAAATGCAGAGAATCCATACCATTAAAACAAACAAGACAGGTCAAATCACCTGCACAAAATGATTGATAATCAG
**CoV-bait-620**	AAAATGCGTAGCAAATTGGGCATAGCTCTATCGCACTTAGGGTAATCCCAACCCATAAGATGCGGTTTCTCTACACCATCACATAAAGTACGCAGCATACGGTTCCAGCCACCATAGAAT
**CoV-bait-621**	GGAGAGGAAGGAGAGCGTGGTTATGTTGAGTGACATATTCACTGTGCATGTTTTGAAGGAGTTGGTAAAGGGCGTTAAAAAGTGATGGATCCTTGTGTTGAAAAATGGCCAATTCACTGA
**CoV-bait-622**	CTGTGAATGTTCCTTCGAATGTTACTGTTGATGTGAAGAGGCCTGAGACTGTTGCTGCTTTGTCCAAATTATGGGCTACCAGTTTATCTTGAGGTTCACTGTTTTCGTTATATTCAATAA
**CoV-bait-623**	TATCGGTATGACAGGTAACAGTACGTAAATCATCTTTAGTTGATGTAACGGCCACTAAAATCTTTCTACCTGCTCTAGACACATAGAAGGATTTAGCAGTGCTTTCTTTACCGTTAGAAT
**CoV-bait-624**	GCCACGGCTATCACAGCAAGGCAAGTAATATGCTGACAGGGAGCTACTGTGAGGCGAATTGGTTAAGATCCCTACTTCCTTATGGATGCATTGCCCGCGTGTGCAGATTACTATAATGTA
**CoV-bait-625**	TTTCTGCAGCACATCAGTCTGAAGTGCAACATAATTAAGACGTGCTTGAACAGCTACGGAAAAAGGTATGGAAGAAGCAGCGGTAAGTCCTCCAAAGAACATACCACCTGTAAGTGAAGC
**CoV-bait-626**	GCAAAAAGTCTAGATTTTAGCCTTCTGACACGCCGTAATAAATGAATAATAAATGCTGATTGCAATAACAACAAAATACTAAGCAAAATCACCAAATAGGGTAAACCCTGCTGAAGTAGA
**CoV-bait-627**	TTACAGTTATCAACACTACAAATGTAGTTATTGATAGCAATTATACGTTTGTGTGTAGTTTGTGTTACATGTTGGTAAGCACAAACACAACACAACAATGGTCTTCTTATACAATCACCA
**CoV-bait-628**	GAACCCAAACATCCAGCAAATCCAAATATGTAGGATTATCATGGACTTCACATTTAGTGACACTCTTAACTGCTGCTTCAACAGCTGGAGGGTCACTATTAAGTGGTGAATCTTCAGGGA
**CoV-bait-629**	AGACTCCACTACAGTAGAATCTGTATGCTGCATAAGATCAATAAGCTTAGGCAGCTTTAAAACTATGGCATCTGGGGCAAGTGTGTGCACGGGTTCTTTAATAACAACCGTGCCATCAAC
**CoV-bait-630**	TGGCCGGTACAGGTACTCTTGAACATGGAGTTCGCGCTAACTTCAACACTTACAGTGGTACACTAGTCGGATGGAGGACCTATAGATCAGTCAACTTGACCGCTGCGCCTTATGTGACAG
**CoV-bait-631**	ATCAAGCTCAAAACAGACTTTAACAGTCTTCTTTGTGACGATCTCCTTCACTGAAGGTTCCTCTACAAAGGAAATTTTCTTTCCAGCACAAGGAAATCTCCAAGCTTGATCTAAAAGGCC
**CoV-bait-632**	CACGACAAGTATATCTGTAGTTGTCTCAGGTAAAGCGTTAATAGTAGAAAACACATACTGAGCACTAACCTCATTCACTTTGAACTTGCTAAAGCATTCAACACGTGCCTTAGCAGGTAC
**CoV-bait-633**	CTTGCAACATTCTCATGTTAATAGCAGAAAGAGATAGCTGAGCACCATTGACTATGTAACCAAATGGTATCATAATCAAAGGTAGACCCAACTCATTCAATGCTTTGCTAACGTCTTCAA
**CoV-bait-634**	TATCACTGCTAACCTGAATACTAAAATCAAGAAAGCTTAGTGCAAAGAAAACAGCAACTATGAACAGACACAAAAACCAAAAGAATTTCTTAACCTTTGAAAAACTAGGAAGACCTGCAC
**CoV-bait-635**	ATGCGCACCTTTCTCACCATGCTTTCGCAGGTACAAAACCCAGGGAAAGGTTTTATCCCGCACACGCTCAGGTAGCGCAAAGAGTGGAACAAACCATCCTATAAACGTACCTGCTCCTAG
**CoV-bait-636**	TCAAGGTCCACTCATCTCGACGAGAGTTCTTCTTTACCTGCACTCGGCCCATCCGTCATAGTCAACTTCATAGTGTCTAATTTAACATCAAGATTCCACACAATACCCAACTGCTTAAAA
**CoV-bait-637**	TGTGCATTGTAGTAGTCTATAATTGGTTGGATTTCTGAAACGTGAAAATTCGACGAGATGTTAAAGTGGTCGAATTGAGTTTGAAGCTGGATTATTTGTTGTTGGAGCGTAATAACTGTA
**CoV-bait-638**	ATTGGTTTTAAAACGGTCCGACAATGACAAAAATGTATGTGCATGTGTTGGTGGTAAGTCAACAGGGTTACGCGCACAATCTTTGAATAAGCCACAAGCCTGGTCAACGGACTGGAGATC

**CoV-bait-639**	GGCCGACAACACCGTTACCATTATCTAGGAATGTTGTGTAGGAAACACCCTTAAGAAAG AGGTGTGATGGTGCCACTACAGGTTTTCCAACATTGACAACAATTGTAGTACCTTTAACT A
**CoV-bait-640**	AAAGTATGTTGAGAACAAAATTCATGAGGTCCTTTTTCTAAATCTTCTTCTACCCAACATTT AGAATTACTCATAAAAACACCATTTTGATAATATAATAAAGCCTTAAATGCTCCTATG
**CoV-bait-641**	TTTGAAACAAGAAGAGCATCATAATTTGGATCAGACAACTGATCAGCGGGACACATAAC ATGGCGCGGACACCAGACATAATTGTCGAGCCACAAGCCATTAAGTGTCATTGAACCGC AA
**CoV-bait-642**	TCGTTCAATTCGCCTTCAAAATCAGTGGTGCAAACAGTCTCATTAACAGTGAAAGTTTTAA TCACTTTCCAAACAGAGACATTTTCTACATCACCAGCGTATTGGAGTACACTGTTAGCA
**CoV-bait-643**	CATAACTAGCTAAGTGTGAGAATTCAGATAAAGTAGCCTGCAGCACCGATGGGTGATCA AGCAGGTCACTTGCTAAAGCCTCTAAATCCACATTGGCTGAGAATGACATAAGAGTAGC AA
**CoV-bait-644**	CTGTTCTTTTACAACGACTTGTTCAGTCTTTGTATCTAAACCACCAGTAGTATTTTCAAATG GTTCTTTAAAGGCACTTTGAGAAGGTTCTTGTTGACACAATTGTTCTTGTGCATCAAC
**CoV-bait-645**	ATATCCACCACCATACTTAATATAGTAGTGGCCACCCTGATCGGGACCCGTGTAGACAAT TTCACCGACGCAACCTGGAAATAGCATCTTACTATCCTTAGTGGCTGTGTAACAAAAGAA
**CoV-bait-646**	ATTTTTCGTGACCTGGTTTTTCGCAACTTTTTAGGGTAAACTGCGCTGCAAATTTACCCTG GTTGCTACACCAAGCCTTTAAAGGAACTCTACAATAACCAACTCGGGGTTGGCTATAAT
**CoV-bait-647**	GCACGTTCTGCAAGATACATAGACTTCATAGCAGCATCAGCAAGCTTTTCAAGCTTACGC TGAGAAGCAGCCTCACGCTCCCATTCCGCCTTTGCAATGTTAACAGCTTTAAGCTTTTTC
**CoV-bait-648**	TACGAATCACACCTTTGCCAGATTTACCATAAATTGTGTAATCAGTGCAAACATCGGTGA CAATGTAAGAAAGATCTTTAACACCTGTACTAGCTTGCGGCACACCCAAAATATTGTTGC
**CoV-bait-649**	GTGTGTTGCCCACTTTACGCTTGGCAAAGAGTTCGAAAGCCACATTTGTGGGAAGTGTAG TCTTATTTACAAAAACTGTATTATCGACCACACCCTCACGAACAAAAACCTTGTCAGCTA
**CoV-bait-650**	ACTAATGTGAATGGCTGGAGAAGCCAGAGTAGTATAAGTTTGATAATGTAAATTATACG GTTCCTAGTGGCATATCCTCCTTGGAGTATTACCCCTAGGGCCCATATAAGGATGATAAA A
**CoV-bait-651**	TAATATCCTCAAAAATAATACTCTTCCGCGCTCTTAAAACAGAGGCACTGTCAAAGAATA ATTTTAAATATTCTCTACACACACACATTTATGTGTAAAGACTACCTCCTACTGCTGACA
**CoV-bait-652**	CGCTGTGTTATAACGTTATAGTCCTGCGGTTTTAACACCCCTTTATTAATGAGTGTACGTG GTGCTGGTAGTTGCTGGGGATCTCCAACATACACAATGTGTTTATAACTAACACGGCTG
**CoV-bait-653**	GAGAGACCGATGCCATTGGCTTGGTTAGATGTGATCTTGCACAGATTCTGCGATGCTGGT GTTACAACAACTGTGACTGCTTGTCTGATACGTTGGAGGGCTTGTCTGGTGATGGTGTTA
**CoV-bait-654**	AACTAACAAGCAATGGCTGGTTGGAAAGAACACGGCCATTGACAAGAGTGGAGGTGTTT GTAAGCAAAAACCAATTATTAAAACTAAAGCTCGAAGGTATATAACCACCATCTTCAACA G
**CoV-bait-655**	AGATGTTGTAATTGTGACACTGGTCCATATAAATAGTTTGCATAGGTACTTCTTTAACACT ATACATAGCTGTACCAAAATCAAAATCTATGCCAACATAATGGTACTTAATACCGTGAT
**CoV-bait-656**	TTAAGGAATTACTACTAACATTAGCCAACACAGTAGTGTGAACCAACTCAAAGAACAACT CCCAATTACCTTCCATATATGCAGTAAGAGTGTTTGAAGAAGTGTAAACAACAAAACCTA
**CoV-bait-657**	GATGAATTGTTTTGTGGTGAGACGGTTGGTGGATGTGTATCCTAGATCTAAGGGTGTTG ATTGACCATGTACGAAATGTGTAAGGTAGTATGCTGGTGTCTCATTTGGAATGTTGATCA T
**CoV-bait-658**	ACTGCAAGCCATTGCTTATGTTAGTGGTTTCAACAGCGACGGAACACTGTATCCACACAC CTCCACTGACGGAACAGTAGAGGGTCCAGGCACACTGTGATCTGTAGTGACGGAACACT A
**CoV-bait-659**	AGGAGAAATTCATTGGTACGGAAATATTGCCAGTGACTACAGGACTAATGGGTTCAGGT GCTGCATAACGACGAGTGGCATTGACAATTTTACCATCGGCACAAATACCAAGGCCACCA T

**CoV-bait-660**	AGGTTCAGCGTGGCTGTAATAGCCCATAAAGCTGACCAAGTACATGGTTAAGAACGGTGCGTCCAGAGGCCCGAAAGCTAGTTTCCACCCCATAGCTGGGGTGAGACCATCCATTCGAGT
**CoV-bait-661**	CTCATAACCACCATACATAACACCATCTAAGTCGCTACCAACATGACAGCCTGAACCAAGTTCAAGCTGGTGTAAATAGCAAAACTCAACGGTACCATTGTTAATGTTATAACCAGGTGA
**CoV-bait-662**	GGATAATGGCACCCGTTTGCGGCCACGATCCTGAAAGCTGACAGAAGCCATAAAGTTTCTGTTTAGACTAAATGAAGCACTTTCTCACTATCTGTGAGAACCGCACTCGGATTACTCACA
**CoV-bait-663**	CTGTAAAGTCATATTCCAAGAGATCAAATGTCTTGAAATCAGAACCAAATATATCACTTTTGATAAAGCACTCACGAGCCAAACAATTAGCCATACCCATAGCAGGCATCATATAAGAGT
**CoV-bait-664**	TGTACAAGTATCGAAAGTGGAGCCAACACAGTCCTCAATAGAACCACTAGCTACAAAACATTCGACACCCTGCACTTTAGTGACACGCCAGCATGCAACACCATCCACATTGCAAATGTG
**CoV-bait-665**	GTTACATGTTTGGTTCTTTGGTGGTGCATTGTAATTTGGAATTGCCCAGCAGTCTGTTGCTTCATTTGTGAAGAATGTTGGCCATGTGATGTATGATGGGTAGATTCTGTAGTTTGTGAT
**CoV-bait-666**	AAAATTGAGAGTGCAAGTACAAGAGGCCAAAGAAGCCAAAGCACAAGCATTTTCAAACCATACAAAATGGTCGAGTATTTGAAATGGCCATACTGCATTACTATCAAAAAGACAGTAAGC
**CoV-bait-667**	GACACAACCAGCCTCAGCAATAGTATTAGCCATTTTAGCAGCACATTCCTTGTAATCGTCATCTACAGTACCGAGACCACTGGTTACAATTTTACTGAACAACAGATCTTCAATAGCAGA
**CoV-bait-668**	GTTAGACTTAGAATTACCCACACCAACGGCAACTTCTCTTCCAAACTTATAATAGCTGATAAAAACAGCTTTAGCATTATAAGTTATATTAGCAACACCAACAGGGTTTTGTGTACAATT
**CoV-bait-669**	GTGTTTGTAGGTTCAACATACGACTGGCAACATAATTCTGACTATTATAAGGTGAGATAAAAACTGCTTTAGACCAAACAGGGTTTTTAGCTAAGAACATTTTAACAATTTCAAGCTGCC
**CoV-bait-670**	CATGGTGTTTGTTGCAAGGAAACAAACCGTATAGCTGCTTTTCCACACGGGCAGACTATACTTTCAGGTTTGTACAAATCATCCAATTGTACAGTGCCATAGTGTAGACAAGCCGGTACT
**CoV-bait-671**	AAATGCTATGACATTGACGGTCACCAAACAATCAGCACCTTCAATCTGAAGAGATGGCTGATCATCAAAACCACCGTACATAACACCTTCCATATTAGACCCTACATGGCAACCACTACC
**CoV-bait-672**	AAAGTTAACATCTTCGTGTAGTTGAACTCAACATGTCAACAACTTTCACATCATTAACAGGTTCAGTACCAAAGATGTAAAACAATGATTCATGAGGTAATTCTGCAACGCGAATCAACT
**CoV-bait-673**	AAAGAATCTCTCAACATACGTCTGTTTACGACGCAACACATGTTGAGCAGCCTTCAAGTTATATTTTAAGCTTCTACTAAGTATGACACCTGTGCGTTCTGGTGCAAGAGCAAATACCTT
**CoV-bait-674**	AGTAGTGTTAACACTACTTCCCCGATACACAATAAACCTTTCTTTACCCAAACTAGTATTAGTAAAAGGATATAACCAGTCTGAAAAATTACCAGTATTGTACTGGCATGCAAACAAACC
**CoV-bait-675**	TAAGTGGTGTATAGCTAAACATATGGTCATTTGCGCATCAGAAGATGCGACATGAGCATTTGAGTGGTACGTGCATACCGCATCATGGTTAGAACTAAGACTACCAGAATAACCCCAAGT
**CoV-bait-676**	CTCTTCCTATCGAGTTGACTTGGACGTCAGAAATCGGTCTGATTTACCCATAAAGTATCGTCGCCACGCAAAACCCCGCTATGTACGTTATGGCAGCAAGTGAAGGAAGAAGCGGTATCT
**CoV-bait-677**	GAGCTCGTGCGTCAGGACCAACAACATTAAGCACAGTTTTACATAATTTGCCTCCTGATGACACATAACAATCACCGGTAGCACAAACTCCTTTAGACTTAACCATATTAGCAGTCTCCT
**CoV-bait-678**	TCATCCAATTTTGGGTCTTCCTCTTTCTCTGGAGACTCCACCTTAGGTTTCAGACTGTCCAGATAATCTAGAACAACCTGATCATAGAGACTCTTGTCATTGACTGCGAGGTAGACTTGT
**CoV-bait-679**	CGACCCCCAAAACCTTTGTTGAGTCTAACAATACACTCAAGCAACTTTTCAACACTGTAACCAGTTTTTGCGGCCAACATGTTAAAAGCATCAGTTGACACGATTTCCGTAAAACTGTTG
**CoV-bait-680**	CATCTCCATCATGCACCATACCAGTGTCATGATCAAAAACAGTATAATGGCTGACAACACCGTTACCATTATCTAGGAATGTTGTGTAGGAAACACCCTTAAGAAAGAGGTGTGATGGTG
**CoV-bait-681**	CCACTTTGTTATGTAATAAAATTGCGGCATAGTGTAGGTGGTTACTGAGGTTAATGTTGACCTACGTGATCTTCTTGAATGTGTATGGTTGACAAACTCAAACAGATCAGTTTGATTGAC
**CoV-bait-682**	ATGAGGTGGTCCTCCTCTACATAATCAGCAGAAGCAACAAAACCATCTACACTTTGAACAGCAATCACTCGCCAGACAGAAACGCCCTCAATGCTAGCTACGAACTTCAGTGTCATGCCA

**CoV-bait-683**	ATCAGTGAGTGGTTTAAGCTGTTCTGGGAGGGTGGGGGCTGATAGTGTAGCAAAGTCGAAGAAGACAGTTGGGGTGGTGTAATTATGTCTGGATCGGGAGGAAGTTACAAAGATAATTGC
**CoV-bait-684**	GCAAAGAAGAAGTGTTTCAAAGTAAGCTCAGAACCCTCTTCAAAGAAACCCTGCGTAAGTAAAAAGTCATAAAAGTCTTTATTAAAATGACCAGGCTTAACAGTTTGGTTTGTAATACCA
**CoV-bait-685**	GCATCACCACTGCTAGTACCACCAGGCTTCACATATATACCACCTGTAGCTAATACAGTTTCAGACAATACTTGGGCGCATTCATTGTACAATCTATAAATGCGCTCAGACCATGTACAA
**CoV-bait-686**	CAAAGCCTTGCACAGCATTACCTGTGAGCCAACTACCCTTACCACAACTAGTGCAATCTGCATAGGCGGAATGGTAGATGTAAGAATGGTTATCAAGAGCATCCTTTCCATAAAAGGTGT
**CoV-bait-687**	GATCAAGTGCAGGATGGATGTTGTAAGTGATAGTAACATCACGGTTGACACGTATTTCCTTGACGGAAACACTTCCGAAAGACACTGCTTTGGAACCCGACTCAGTGGGGTTGCCTCCCT
**CoV-bait-688**	CAACGTCAGGTGAACCAGTGGCTGAATTTTTGTAAAGTCCAAAAATGTTGCCATTTGAACACAATGGAAAAGCTAAAGTTGGTTTGTGATCAATGCAATAGTAGCTAAGGCCACCTAAAT
**CoV-bait-689**	AACTTCTCTAAATTACTAAATCTAGCATTGCCAAATCAAATCTAAACTTCCTAATATGCAATAGGGTTGCTTGTACCTCCTATTACACGTGCTTACCATTCTGTACAAGAGTAGACAGCG
**CoV-bait-690**	TTAGAGGTTTATAGTACACCGAAATGGTGGCTTGCAGTAGTTTGTTGTTGGGAACGTTACTTTTGCAAGTTGGTTGGAATACTTGTAGTCTTCACCTGTAAGGCGTTACTTCCAAGTATA
**CoV-bait-691**	GCACTGAAATTTTTACTCCAAGAAATAGCTCCGGCTGTACACTGCTCTGCACCACCTGCATTATTTGGTTCTTCAGTAACATTTACTACTGCATAAGCACCTCCATATAAATGCCAACCA
**CoV-bait-692**	CACATGTCTTGGGCAGTAAACTGTGTCACCTAACCAAAGGCCATTGAGCACCATGTTACCGTAACTAACACGGACTATACAGCGCTCAACAACACCTGAAGGTTGTGCCATCTTTTTAAG
**CoV-bait-693**	AACAACTTGAACTCTGAGTACTTTGGTGGGATCGTACTTTGCGAGGTAATACACGCGCCAGTCTGGGCTTGTGTAAGTTGGGTGTGGTTGACCGTGGGCGGTTTCAATGTCACAGATGGT
**CoV-bait-694**	ACCCGACATAACCTACTTACGAACTGGACAGTCAATACATGAGACCGAAGTCACAAAGGACATTCACTACAGTCGCATAACCGCTGAGAGCGACTCCTGGCGGTCGAAGTCAGGAGCCGC
**CoV-bait-695**	ACGTAGCATCCACAGATGTAGCACGATAACAATTATCGTACAACTGTCTCTGCAGGCTCTTAACGGACTCATTATGAATCGCATAACTATCAACAGTAAGCAAACGGTTGATATTAGCGC
**CoV-bait-696**	AAGTCAACAGACAAGGTAGCAAGCAATTCTGAGTCAACAAGTTTAATGGGTTTGCACAACAATTGAGACAAATATACACACAAGTTCTTAACTTGCGTCACATTAGTACCACTGTTGTTA
**CoV-bait-697**	TTCCCACTCGAGGCATCCGAGGCTAGACAGTTACGCCCTTCAATCCTGATCATTGAACCGTCAATTCATTTAGCGAAACGGCATTCGGAGCGCCGATGAGCCGTTAAATACGTGATGGGA
**CoV-bait-698**	TTCAGGGACATAACTTTCATGTGAAGATCCAAGAGGGTATGTCAATTTCGTCCTCTTCTGAGGATTCATAATCACACCCAAAATCTGGTCCTTCAAATGATTGCCACTCAAGAAAATGTC
**CoV-bait-699**	GACAAAGTAAGACACATTGTTCACAACAAGTGTGATAATAATCATAACAATGAGAAGAGTGCAGTCACCAAAGATTTTCTTAAACTTAAGAACACCATAACACATTGCAATAGCCAGGCA
**CoV-bait-700**	CTGGTTGTTTAACAACATCACTATCACGGATGATAGCACCATTGAAGTAAGCATAATCAGGACCTACCATACAAGGGTATTTCTTGATTTTTCTATCAGAAATAAAGATGGCATTGGGAG
**CoV-bait-701**	CACGTAGGCCGTTAGCAACCATATACTTAAACTCAGCAGCACTAACAGTATAGTCATAGACACCTACACTAACCTTAAAAAACCTGTTGAACCAGTAGAGAATACCGTAGAAGCAACAGG
**CoV-bait-702**	AACAGCACTAAAAACACGAGGTAGAAAATGCAAGAAGTCACCATTGATTGCTCTCAGCACAGTACCCGGTAAGCCAGGCACTATGAAACCAATCTCTCTTGTAATGATAGCAGCTACTAC
**CoV-bait-703**	AACAAACTTATTAAAAACCCACCAAATATACAAATACACACAATCAACAACTCTTCAAAAATTACTAATCCAACAATAAATCCAATTTATAATAAACCAACAACAACTACTAACATACCT

**CoV-bait-704**	CCGATACATCTAACGCACCTACCGGTTGTACTAACGGCGTAAATATATTCGCTATTAAATTCATCGCATCTACACCACAAAATACACCCGATAACGCACGATAATGTTCATTATTTAATA
**CoV-bait-705**	AATAACAATTTAACCTTATTATTATTAACCTTTACCAAAAACTTTACTCTCAAACTAATTCAATCTATCTAACAACAATAACACAAAAACAATTTCACCACCTCCACTAACCATTCAAAC
**CoV-bait-706**	CCGGTTTACCTTTCGTTACTTTATAATTACCACACGATTCTACTATCGTTTTAAACGATTTATAATCTAACGATTTTATCGTATCTATAAACGCCGACGTCGACGCCGAAAACGACGCTA
**CoV-bait-707**	CTGACATTGTAGTCTTTGATGAAATCTCTATGGCTACTAATTATGACTTGAGTGTTGTCAATGCTAGACTTCGTGCAAAACACTACGTCTATATTGGCGATCCTGCTCAATTACCAGCCC
**CoV-bait-708**	AGCTATTAAAATCACATGGGGATAGCACTACTAAAATTAATTTTACACATTAGGGCTCTTCCATATAGGCAGCTCTCCCTAGCATTATTCACTGTACCCTCGATCGTACTCCGCGTGGCC
**CoV-bait-709**	CTACATGCCTGCTAGCTGGGTGATGCGTATCATGACATGGCTTGAATTGGCTGACACTAGCTTGTCTGGTTATAGGCTTAAGGATTGTGTTATGTATGCTTCAGCTTTAGTTTTGCTTAT
**CoV-bait-710**	AAAAGAACCCCTCATATAATCACTACTTAAATAAGTAAAAGAACGCAAAAAGGAAAAATTAAAATTATTATAACCAGGAACAGCACTAATCTGTTGATAATATACAAAATTTGAAACACC
**CoV-bait-711**	GGGCAATTTGTCACAGGGTTAAGGGCAATTTGTCACAGACAGGACTGTCATTTGAGGGTGATTTGTCACACTGAAAGGGCAATTTGTCACAACACCTTCTCTAGAACCAGCATGGATAAA
**CoV-bait-712**	CATTTTGTCATAACTGCATCACATAATGCCTCATGTGAATCACCATGAAACTGTTGGCAAATAGCATCATGTGCATCTTGTAATTTAACTCTCTCTTTCTGAACATCTAAATATTTACAA
**CoV-bait-713**	ACAAGTCACCAATATGGCAATAATACCACCAGCCACTACTGAAGCAGACACATCTAAAGCACCCACAGGTTGCACAAGAGGAGTAAAGATGTTAGCTATGAGATTCATCGCATCAACACC
**CoV-bait-714**	AACAACATCAAATGGTGTAAACTCATCTTCTAAAATAGTGCTACCAAGGATAGTACGACCATTCATACCATTCTGCAGCAGCTCTTTCAAAGCAGCACACATATCTAAGACGGCAATTCC
**CoV-bait-715**	ATATGCATAAGTATCATTAACACATATTCCTGACCATGCCGTCACTTCTTCCCATTCTGTAGGAAGCAAAACCGTGTGGAAGAAGAGTAAACCATCAGGTGCAGAATTGACAAGTGAGAG
**CoV-bait-716**	CTAAGACAAAGTAATTCTGCATAACATCAGAATCTGAATTTGTAACAGCTTTTGCTGACAACTTATTGTCATGCAAGAGCTTATACATACCAATAACTGTATGCAAACCTCCCAATTGAG
**CoV-bait-717**	TCTTGTACAAACCAAAAACGTTACCGTTAGCACATAATGGGAATGCCAACTGCGGTTTATGATCTATACAATAGTAGTTAAGTCCACCTAGGTACAGTTTTCTAACATCGTTAACATTGC
**CoV-bait-718**	ACTCGTTCACCTTGTCCTTAGCGAGCTGAGCGGACAGAGCAGCCGATTCTGATCTAACGAGTTGCTGAGCGACGAAGGCGTTCAGAGTGGTCAATCTGCCGTTGATCAGCCTGTCGATTT
**CoV-bait-719**	CCAATTAGCTGATAATATGAAACCAAAGTGGCGTACTTATCGCTAACATTAAAGGACGGATGTAATTTCAATGTACCATAACGTTCTTGATTAATTATGGTTGGAGCTGTTAATGGTTGG
**CoV-bait-720**	CGCCTGTTTAAAGGCGAAAAATTTGCCACTAATCACAATTGACCATCTGCAAACTCTCAAAAAGTTGTAGTAGGCAAGCAACTGCTGCTGATCAAAACCAAAAGCATCTTGCACAGCACG
**CoV-bait-721**	ATTCTACGACTCCAATTTTTAGATTTTAAACTTCTGAAAATGAAGGGCTATTACAATCAATACGCGCCCGTCTTCAATGCCAATACGCCAACTGTTGTTGGTCAACCCTTTCAAGACTTT
**CoV-bait-722**	CACGGACAACATTTGATTTCTCTGTGGCAGCAAAATAAATACCATCCTTAAAAGGTATGACAGGGTTGTCAAACGTATGATTAATAGTATGAAACCCTGTAACATTAGAATAAAATGGAA
**CoV-bait-723**	CTTCTTTTATTACATCTTTAGACGTGGATAAATGCCTCAAGGCATAAGCCAGGTCTAATAATGTATATTTTGTTAAATTCTGCCTGTTTACATTGGGTATTTTGTCGAACTTGAAAAATT
**CoV-bait-724**	GAGTACGGGCAGTTACAAAGAACGTGCCGTTAGCCCTAAATATAATACCACTACCAGTTTTAGGCATAACACCATAAGAACCAGTACGTGTGGTAACACACAACCCTGGTACCGCCTCAA
**CoV-bait-725**	AGTGGCAGTTGACTTATAACACACAGTATCTGAACCATAATCAAGCTTCTCGAAAGTAAACTCACCAACTTGAAACTTAGAATCCTTCGTCACCTGAAAGCAGGTGAAAACAGAATTCCT
**CoV-bait-726**	AGAAGTTTGCGCAAAGATAACATAATCATACTCACTGCCTTGTGAGGAATCCACAGTCTGAATCTGAAGGCCCAAAGACCGGCTAGCAACGTAATTCTGGCTGTTATAAGGCGAAATAAA

**CoV-bait-727**	CACACACCAGCTTTAGAATCAAGACACTCACCAACGCGACAATAAGTAGTCTCCATTGTTTTAATGGTGACAAACCCCAAGCCGCGAGCAAGTGTTTCCGGCAATTTAATAAAATTGCCA
**CoV-bait-728**	CCTGGTGGATATCCCGTACTAGGTTGCCGGGCGTCCTGAACTCACCATTCACAGCCGTGAATGTCTAGCAAATGGCTCGTATTCTATCTTACAAATCTCGACTACACTGTAGACAATGCT
**CoV-bait-729**	CATAGTGGGAAGTATTCAGACGATCTGCATACATGGCCGGAATGACTTTCACAGCACCAGAATCTTTATAAACTCTGGTACCGGTATACTCACTAGCATAGGGGACGTACAATAGGTTTT
**CoV-bait-730**	ATAGGCTCCGCCCCCCTGACGAGCATCACAAAAATCGACGCTCAAGTCAGAGGTGGCGAAACCCGACAGGACTATAAAGATACCAGGCGTTTCCCCCTGGAAGCTCCCTCGTGCGCTCTC
**CoV-bait-731**	TTCCACAAAATCCATAACGAGTGGATTGTGACTTAACACACTCATTAATCTTCTCCTGCGCCAACAACCTGCTAGCACGAACTTCTGTGTACTTAGTAAGAGTCTGAGTAACAAAAGCAT
**CoV-bait-732**	GTGTAAAACCACCCATAGTCATGCCACCAAGAAGGGAAGCAGTATAGATGGACATCTTCTCCTCATCAACAACACCTGGCAGTACCATAATACCATTATAATACTGCGCACATGGGACAT
**CoV-bait-733**	AAATAACGCATAGTAGCCAATTCTAACTGATGTGCCCACGTTATAAAACAAATGTGATCAGTTTGCGGAGCAAGCTCTGCCAAATGAGCCACAATTTCATAACGTACTACTTTCCAAGGA
**CoV-bait-734**	TGATAATTAGGAATGTCACAAGGTTCGAGTTCAGTAACTAATGATTTATAAACAGAAGGCATTTGATAACCATTAGTTAATGATTGTACTATAGGATAACAAGTTTGAACGTTAGCATCA
**CoV-bait-735**	GGTTTACCAAATCATCGCGCGTGCACAAAACGTGTCTAGGGCAAAGGACGTAGTTGTTAAGCCACACTCCATTCAGAGTCATAGTGCCATACGACACTTTAACAATGCATGGTTCAACAA
**CoV-bait-736**	TAACATCCAACTTCAAATCAAATGCTTTAAGGTCATGCACAGTAAAACGTTCTACTATAACAGTTTTCTGTACTAAAGTTGAATCCAATTCCTGGGCTTTTTCATCAGGCTTAACGTCAA
**CoV-bait-737**	CCATATATTTATATGTTCCTATAGGTAGTGCAAGAAATTTATTAATAAACCAAAAAATGCCAAAACGCATACATGATATATAACCCAAAAGGAAATACACACATATGCGAACTGCCAAAT
**CoV-bait-738**	TGAATTATTAGTAATATAGGACAATAGATACGAACCACCATATATATTGCCCTGATTAGTAACGTGAAAATTACGGGTTAAATTATTATCTAAAATAATCTGATCCGTAAGTTCTTGCAT
**CoV-bait-739**	GGTAAAGTGTTCTATCTGTGTAATTGCCATTAGTACAGGCAGTTAATATAAAACGCAGCTCAGTATGGCAGCGACACATTACATATACTTATATAATCTGGAATTACCAGAACCAGATGT
**CoV-bait-740**	TATAACACTTATATACTAACTTAATACAAGCTAAACAAGTGTTAATAAACAAAAGAAATACTGCAACACAAATAACAACAAGTGCTGCAATAACGTAATCGCCAGGAATAGTGATATCCC
**CoV-bait-741**	CAAAAACCTGAGCAAGCATTGCAGTCTGCTCGGGAACCTTGTTAAATTTATGATAAATAGCAACAACATCAGCTTGAATGTCATAGCCATTCACAGATTTACGGCCATCAGTATACCAAC
**CoV-bait-742**	AGCAGCCAATCGACCAGTAATAAGTCTATCAACTTGTTGATCTGCTTCAACTATACTAAGTCTATTATAAATATCTTGAATAGAACTCGATATAGCTTGAAAATTATTAGACAATTGAAT
**CoV-bait-743**	TCCACTGCCTTATATTCTACATGTGGAATTACATCTGAATAAAGTTTATGTGGTACATCAACTTTATCATAACAATAAAGTGTCCTATTATTATTATACAATAAAAAGGTACACTTAGCA
**CoV-bait-744**	CCATTTAAGTTACCATTAACATCATATAAGAGGTTCTGCCAACTATTGTAATAATCAGCCTTAACCTCCTTAAAAACACCTTGGCCTGTACTACCGTAGAGGTCATACTTGACACAAACG
**CoV-bait-745**	CGGTGCCGTCATAGCTATAGAAGAAGCATTAACAACACGATCACCACCAATAATACCAACAATACACTCTCTTAAATTATTAGATTCTATAGAAGTATTAACTACCGCATAAGCACCCCC
**CoV-bait-746**	GGAGGTGTAAAACCGACATTACGTCGAACATATAATTCAAAAGCCACATTTGTGGGTAATGAAGTTTTATTTTCAAAAACTACTATATCTTCCAAATCCTTCTTAACTATAACACGATCA
**CoV-bait-747**	ATTAAAACTAAAACCTTCTGGTATGTGGCCATTGGGCTCAGTAGCAAATACATTGGCAGCATAACCAATGCAATTAGCTGTCCTAAAATTAGTGTTAGCTGAGCACCTGGTGACATCTTG
**CoV-bait-748**	TAACGGAATTGGTCGAGTGACGATCTGACGCAATCCTATGGGGACACTACGAGCACCCGAACCGGTACACGACCTACCCGAACTGTTCGCATAGTGGCCTTATGTGGATTTTCTCACCCT
**CoV-bait-749**	CTAATGCCATCGCGTCCTCTGTGTAGCAGTCACATTAGATGCTAAAGGTAACGGCTAGAATTTGATCTAATTCGCACGAGTAACGGCTCGCGCGCGGACCTTGTGCGGTTATAGGTAGAG

**CoV-bait-750**	AGTTACAATAAACAGTGCAAAGTCCATTAGCAGACCATGTCATACCACCTAAAGGCGCAGTTATAGCTACTGCACTTTTATTAAAATGGTGGCCATAATTAATACTACCCAAAACACACT
**CoV-bait-751**	CATAATTTGTGCACATAGATACCTCATCAACCACACAAATATCAGCATTAACTTCAGGTAACGCATTGACAGTGGAAAATAGGTATTGTGCATTAGTATTATTTGCCTTAAAACCATCAT
**CoV-bait-752**	TTAAACGGGGAAATTTGTTAAACCAAAGTGATTTACCCTCTCTCAGAAAACATAACTTTCCTAAATAACTTTTATTAGTAAAAGGTAAAGCCTTCTGAAAATTGCCAGTATTATATGACA
**CoV-bait-753**	AGAAGACCCTGTTTTTCATAAGGGTCGTCGGCACGAACGGTATTCCACGCGTGGCGAAAAGTGACACCATTAACGGTGATCTCATCTGTATCATCATCAAAGTGCTCAACAAAAGACCAC
**CoV-bait-754**	ATAACATGGTTGTGTTCATCAAAGTTAGAGGATGCAACAAACTTATCAACTGTATAGGCCTTAACAACACGCCATGCGGAAACACCCTCAAGTGTGTCAACAAAGGTGAGAACAAGACCA
**CoV-bait-755**	AATACCGTAGAAGCAACAGGTAAAATAACCCAACACAAGGTAACAGAACATAACACTCTTAATATCACCAAAAATAGCCACAAAAGTAGGAAATCTCAAGGCCATATAAACAGCAGCTTT
**CoV-bait-756**	TCTACAGAGTGAGTATTAGATTTATTAACCTTAACAATGCCTGCGTTGTACTCAGTCGCCAAATCAATATAATGCTTAGATCGTGACGACTGATACGCGCCATTTTGAAAACAGAGATGT
**CoV-bait-757**	TATTTCAGCCATGTGCAGAATGTCAAGACTGCAAATATAACTGAAATAATAAAAACTGCATCAGTCTTGCTGCTGCCATGATTCACTGCTGCCCAAATAGTGACAACTAGTGTAAACGGC
**CoV-bait-758**	GACATAGAGTCGATCAAAACCTTGAAGAATACCTTGAACTTGTCAACAAAATTCTTGACCAGCTCAGGAACATCGCTTAAGAAGGTTATGCCACCATTAGCCACTGCAAATAAGCCATTT
**CoV-bait-759**	AAATTTCTTTTCTAAACCACATTGAATCACCAACCCCACCCTTGAACAAGGGGTACGACCACTCAGGTAATTGATTATAAGAAACAGCAGCATACTTTCTACCCCACATCAAAGACAACA
**CoV-bait-760**	TCGCAGCGCTTTGAAAAACAGCAAGAAATGCAACGCCAACAACAAGCCATCCGAAAGGTAGAGTGGCCTGTAAAGGGATGGACGCTGTAGAATGAAAAGAACTTGCAGGAGAAACATTTT
**CoV-bait-761**	AAAGCAGAACATCAAACTTATGAGCTAGTTTAACACCAGTCTCAAAAGTAGAATCTCTGCATTCAATGCCAGCAACATTCTTACAATTCTGTAAGTCGGTACACCGTGTAAAATCCTTAT
**CoV-bait-762**	AGTTGTAATCATCTGCTTTCCAATCATTTGGTAATATGGAACTAAGTTGGCATAATCATCACTAATATTAAAAACAGGACGCAACTTGTAAATAGATGCATAACGTTCCTGATTTGCAAT
**CoV-bait-763**	TCAACACCTTTTTCGACAAATTCCGCATCTCCAAAATTTTTGAAGCCCCCCCTGGGGCCGAAGCAAGCTCCTACGCTATTTTCGCCCTTGGGAATTCTCCTCCACTCTGGGATGTCTTTA
**CoV-bait-764**	CAGAAGGAAAGCGCGTAGCATTAAAAACCTTGTCAAATGGACAACGGTTTGTAATGTTTGGGAATCTAACAACTTCTTGCGTTGGTGAAACTCTGAAGTTAGATGTTTGATAGATTCCTT
**CoV-bait-765**	ACACAGATCACTCCAGTCGCCCTCAAAGCTAATGCGCTTACCAATAGCTTGTTTGCAGGCCTCAGCAATAACATCATTCTCAAACTCAAAAATAAGTTTGACGTTATTGCGTGCTGGAAT
**CoV-bait-766**	GCTTGTTAACAACATCTTGAATTTGTTGTAAAGCTAGAGAAGTACTTCTAAAGCCTTCCTGCATATGGACAATTGCCTTATTAAAGGAGGCTGCAATTTTTTCTTGATTTTTCAACAAAA
**CoV-bait-767**	TCTTCGATGCGGGGGGCTTGACTGCTCAAACAGACATAATAGTAATTAGATCCGCACTGGTCAATCACCCCCCCGCGGTTAGATGAATTACATGTGAGTGCATATAACCTATCAGAGTGA
**CoV-bait-768**	CAACGCGCACGTGACGGAGCTACGAAATTACGAATTGCGTTTGTCCAACGTTTTGCCACATATTACCAGCATCGACAGTAATAGATGCGTCACGCCAGAGGCGTGCGGCGGGGCTAGAAT
**CoV-bait-769**	CACCAGGGCCCACGGGCTCACAAACTTGAAGCGTATAAGATTCGCAGTCGTAACAGCTAGGGGGATCTGCCTTGGTCGTTGTCAGCCAACTTGATTCGCACCCCCTTTATGACCTATTTA
**CoV-bait-770**	TTGAGCAACAGTAGCCTTATTAAAACTACCCTGCTGTACATAACCAAAAACTTTATCCTGGCTGCATATGTCTATGCCCCCAGGACACTCAAAATACTTAACGGTTCTAACACCATTAAT
**CoV-bait-771**	CACAGCAACTGTTAAGTTATAAAACAAACTGCTATTTTTCATAGCAGAAATACGAATATGATTCTGTGGAATCAGACCTGTTAAAGGACATGAACCAGGACTACTTTTATAGCAATGTGT
**CoV-bait-772**	TAATTAGTTTCAAAGTAGTATTAACACTACTTTCACGGTACACAATGAACTTTTCCTTAACTAAACTAAAATTAGTAAAAGGATAGAAGCCGTCAGAGAAATTACCTGTATTATACTGAC

**CoV-bait-773**	CATATAAATTATGCACCTTATTACCATCTATAGATAGTAATGTATTAATGTTTGCACTAACGGCCTGACATATATTAAACACACTATTAGCGTATGCAGTTGTTGAATCACCACTACTAG
**CoV-bait-774**	CTACCACTTGAATAATAACAACACTCAAATGGGTCGAATATTTGTGTTTCTAGAACACTTGAAATATGTTGTAAATCGAAACTGATTTGTTGTAACACTAAATCACCAGTTTCAATTATT
**CoV-bait-775**	ACTAGCAAATCTAATACTACTATAATTCTCCTGCGGCACAAGCGTAGGAGCACTTAAATTAGCTACTGAATGAGAGGTTAAAACAAAAACATCTCCTACAGATAGCTTATAAGTGGTTGT
**CoV-bait-776**	TCAGAACTTAGCTGATTAAAAGCATCCACAGACCATATACAGGAAACACCGGCTATTTTAGCAACATTACCCTGCACATGCTTAGCAGAATTTTGAATCAGAACACCTAAATCAGCTGCC
**CoV-bait-777**	GGCAGTACCAGCCAACTTAAGCGGGAATTTAGCCATATCAAACAGGCTATAAGCACCCCCGTTCCAAACTGTGGAATTTCTCCAAAACAAATAATTGGCATGCATAACATTTCCATCTAT
**CoV-bait-778**	TTGGTCAGCAACACAACCTTTATCACGAATGTAATTAACTAAATCATCATGCAATTCTTCTACACCTTTAACATCTAAAACTATGCATCTTCCTAGGATATGGGGGTTTTCAAATTTAAT
**CoV-bait-779**	CTCCTACATAAACAACATATTGATAAGAAATCTTCTGATTCAAATAAGAAAGCTCATAATTGGTAAGCATGCTCACCTCATCAACAAGCAATATATCACAGCTAACTTCTGGAAGTGCAT
**CoV-bait-780**	CCCATCGTCAAGGACAGGAAAAACATGTTTAGGAAAACCGTCACACTCAGTACATACACTGTAAGACTGTATAAGACCTTGGCTATTTGTGGTGATGTTTAGTGTGGTGACATTTGACAC
**CoV-bait-781**	TTATCTCATTCACTTGCTTAACAACAACAACAAAAATATCACCACCAGGGCCTTCACTAACTCTGTAAAATTTTGTATCAAAAAAATCCAAAAATACCATTGCATCTTTAAACAAATTTA
**CoV-bait-782**	GCCCATGGATCATTTTTAGCAGAAACTTCTGCTTTTGTCATCAACAGCTGTGTTAAGATCATACTGTGAGATGCTAACACCATCTGGGTTGAATGTACAAATCCACCACATGTGTCGTAA
**CoV-bait-783**	CGTCTACTTTATTCAAACTAATATTGGCTGCCTCAGTCCTTTGTGGTGTCACATTAACAGTAGCACCAATCAAATTAGTGGCAAAGAATGCACTATCAAGGCCTTTCTGTCTCTTGCCAA
**CoV-bait-784**	AAACTGTTAAGTTATAAAACAAGTCGGCAGGTTTACCACCTCCTTGTTTCATAGCAGAAATGCGAATATAGCCACGTTGAATACGACCTGTTAAAGGACAAAAGCCCTGGCCACTCTTAA
**CoV-bait-785**	TTTAGTCACAGAAACTGTTAAATTATAAAACAAGGAAGTACTTCCCTTCCTCATAGCAGAAACACGAATAGCGCCACTTGGAAGAAACCCTGTTAGCGGGCATACACCAGTACCGTGTTT
**CoV-bait-786**	TCCAGAGCCTTAGCAATTCCACCACCATGTGAAAGTTGTTCATTAGCTGCATTAACAACAAAATCATGGTTCACAGACAACAAAGCACTAAGTTCACCTTGGTAAAACTCAATGTTCTTG
**CoV-bait-787**	CCACAGTCGATTGTACAAGTTATAGACATGCCATCACGGTCTTTTAACAATGACTCCAAACAAGCAGAGACATCACCCAAAGAACCAAAGATAGCACGCTGAGACTCGTAACAACGCTTG
**CoV-bait-788**	CCTACAGGTAGTGTCTTGAAAGCTTTTTCACATAATGCATCGATCGCTGCATGGGATGACGCCGTGTAGCAAATACGCGCATTGGGATAGTACTTAGCCAAACCTATAGCAAAGGTTGAT
**CoV-bait-789**	ACCGGTAAATTTGTTCTATTGTCAAATAACTTGACAAGATTATTATCAACCTTAGCATATACAGCATTACTATTAATAACTACATCAAGCGCACCATCAACATGTGCATTGCAGTTCTTA
**CoV-bait-790**	ATGCATGTTACATTTTGCTTGGCATAGTCGTTAGGTTTTCTATCTACATCCCAAGCCTTAACATACGTGATGCCATTGAGTGTAAATTGGTTACTGTCTTGGAAGTAATCCATGAAAGTC
**CoV-bait-791**	CGTTCTACAACAGGAGTAGCTATTTGTACAACAACCTCCTCAACAATTGATGTCTCTTCACATGAAACAACATTATCATGTGCAGCCTCTTGATTCAGAGATTTCTCCTGAGCATCAACA
**CoV-bait-792**	ATATGGTTGCTTGCCATAGCTAACAGTTATCGGTTTCAAGTCTGACGGAACAGACTTTCCACACAACCTGCAGACAAAACTGCAGGAAGGCTACTTTGAGGGTGTGAAGTGTGAGACGGA
**CoV-bait-793**	TCAACAAGGACATTACCAGAAGTGGCAACGTCATCTTTTGACTGCACTTTGGCTATACGCCAAACACCCCAACCCAAGTTAGTGCCTGGTACAACAAGGTCTATAATGTGTTTTAAAAAG
**CoV-bait-794**	GTAGGGTTAACAGGAAACACACAATGATTGAATGTGTTAAAATCGGCAACACCATCTTCTGTTGGAATTTGCAGGTCCTTTGCAAACAAGCGTGTCACTGTGTGATAACAACCGTTAATC
**CoV-bait-795**	TATAAATGTCACCAATAGAATTGGAAATAGCTTGAAAATTATTCTGCAATTGGACTGTTAGTTGACTGAGTGCATTTCCTTGACCATTCACAACATCCTGTACTTTTGAAAGTGCCTCTG

**CoV-bait-796**	CTAACCAAACGGACAAGTGCTGAAGGCAAGAGAACATAGTCTCCAATCTTGGCAAAAGACTTACCTGCAACAACAAGTGCATCACAACTACTGGTACACAAAGACTTCAAAGCAGAAGTC
**CoV-bait-797**	GCATTAGGTTCATCATCCACAGCCTTAAAGTCATCTTCACTGTCAGACGACAAAGGCCACTGAGATACCATAATAGTTAGATTCAAGTCTGTGCCACCCTCTTCGTCATAGACAAAGTAG
**CoV-bait-798**	ATGTCCTCTGCAGTCAACTCTCTACTCTTATTTTGCTGCACGCGGCTTTTGGGCACTGCAACACTTATATTATCATTTTCTCCTTGTCCATTCTTATGACCACGCTGACGCTGTGGTTTT
**CoV-bait-799**	ACTCTAAACGGGCGCTCATTTGCAACGCCTGCTCAATTGTGTCGCAAGCTGAAGTGTATTGTAACAACAACTTGTTACACAATGGGTTACCGTTGCAAACATAAGTGGCACAGTCTACAA
**CoV-bait-800**	CTAAACACAATATAACAATAATAGCCACCAGCATATATTAAAACTGCTACAACAGCTGCTATTATAAAAGTATTAGTACACAAAGGTATAACTGTAACTGCAATCAAAATGACTGCTACA
**CoV-bait-801**	TTAAGGCGAGTCGAAAACTCGTCATCAAAAGCTGACACACCCAACTTCAAAAAATCATCAGTTTGGACACAATTTAGCTTACGTATATAAGCAAAGGCATCACCCAGATTATATTTGTAA
**CoV-bait-802**	GCACAAGTGCTAGGTTAGGCTCTTTAAGAGCGTAGCCATAAGTGTTGTCTACACACAGCCCTGAATAGGCTACAACAGTAATGTAGCTTACAGGTTGTAGTACCGTGTGCAAAAACATAA
**CoV-bait-803**	CTGGTGATGCATAGGCATAACCGGCAAAGTATGTGGTGTTAGTTTCACGTATAACACCAGTACCGGTATACCCATAAATGGTGTAGTCTGTGCACAAATTAAACACGACATGATCCAACT
**CoV-bait-804**	GTACCATCTACACCACCCTTTTCACAATTTTCACAGAAAAAATCGTGCTTATCACAGAAATTTGTGCCACCATTGGTATTAACCATAGTATAATGTTTACGCCCCTGCAAAACCACCTCT
**CoV-bait-805**	TGATATCCCAGACGATGTTACCATACACAACATAATCATGTACAACAACCTTCTCCCAAAGAGACATATCTGGAATAACTATTGTTAGTTTATTAGAACAAGTAATCGGTACACTAGCCA
**CoV-bait-806**	TTGACAAAACACTCACCAGCAAGACAATTAGTCATACCCATAACCGGCATCATGTAAGAATAATATGATGTGCAAATAGGCACACCCATGTCTCTAACACTGCTAGTGAAATCGCCAAAA
**CoV-bait-807**	ATATAAAAGAAGCTTAAATTAACAAACAAGTCCTCAAACAAATAAAAGAAAACACGCCGAACCACTGAATTCGGTATAAACAAGATATAAGAATACCTCGCTGTTACCACCACAAGTGGA
**CoV-bait-808**	ATGTCACGCTCTATAGAAACAAGAGCAGCATTGAGAACATCATCAGTTACCAAGCCATCAGTTGCACGTGCCACATCTGATGCTAAATCACCTGTACTGATTATATCTTTAAAAACATAG
**CoV-bait-809**	CCTGAAACTTTGCTCTCAAGCTGGTTCAGCCTGTCTAACAGCAATAATGCAAGTGCCGTATCTCCTCCGGCAGCCATGCGTGCAGGTGATGAACCTCTGCTGCTGCTAGGTGTTGAATTT
**CoV-bait-810**	CCACCGTCATTGTTAGATGTACATAGTCCTTAACACCACGTACCACTTCAGCGGTGGGTTGTATGTCCACGGTTGATATAGCAGGTGTCGCGTAAAGCGCATCCGCTACCAAACGGTATA
**CoV-bait-811**	CATTGCTACCAGTACAAATATTCTTATACAGACCAAAAACCTGGCCATTCGCACACAGTGGGAATGATATCACCGGCTTGTGATCTTTGCAATAGTAGGACATGCCACCAAGGTATAATT
**CoV-bait-812**	GCAGCCTTACAGTCAATCATAGCACCAGCACCAACCTTGACAGGACCATTTTTCTTAATGTACCTGTCAGACAGAACCTGAAGTTCACCACCTGTAAAATCATTCAAGGCCTTGGCGATA
**CoV-bait-813**	TGGTGTTCATTCTTTTTCTGTGGGCTTTGATTTCTGGTGGTGTGAATGTTGAAATATCCATTTCTTGGTACATCTCTAGTGAATTTTGGAGGGACATTATGTCACTTTGTGTGGTGATGT
**CoV-bait-814**	AACCGAAACCGGGTGGTATAAAACCACCTTCTTCAACTGCAAATACATTGGCAGCAAGACCCTTACAATGCTCTGTACAATTATTCACAATGATTTCACCAATGCCGGTTTTGTTGACAG
**CoV-bait-815**	CATAGAAGCTGGCCATAAAAGCCATAATATGAACATCTTAAAGACGTAAACCATCATACTCCTAGATGGATAACCATATTGGAGTACGATAGTAATAAGTAGGAATATTATGGACCATGA
**CoV-bait-816**	AGCTGTCTCTAATTCAGCATAAGTAGCTAAATGAGAGAACTCAGTAAGAGTAGCTTGTAAGACTGATGAATTCTCAAAGAGATCATTGGCTAAAGCATCTAAATCAACATTAGCAGAAAA
**CoV-bait-817**	CAAGCCAGAAATAAACTTAGCTAAGTTAACGGTAGCAAAAACAGTAATTGTGTAATCACTAGTCAACGTAGTTATAAACATAAGATAGGTTATGGGACTACTGTGGTCCCCTATAACATA
**CoV-bait-818**	TGGCGTATTTTGTCAACATTAGTTGTGGTTCCAGAGAGAATTGCGAGGACAACATCGTTGACAACGAACTCTCCCACAAAGTTAAGGGAATGCTGAGTCTGTTCCAAGAACATGTTAGCA

**CoV-bait-819**	CAGTTGTCGTGTAGACATAGGCCTGCGTAAGAAAGATCTTAAAAAGGTTTGCAAGGTCTAAGAGAAAGTTGACAGTTGCAGTTGTAGCCTCGAAGCATCCATTAAGAAACTTCGAAACTG
**CoV-bait-820**	ATATATTATACTCAACACACGTATTTAAAGTAATATTATTATAATTGTGTTGAGTTATAACCGGTGATTCTGTGGCTGTTTGTATGCGAGAGCCATCACTCTTAGTAACATAAACCAACA
**CoV-bait-821**	TGTGGCGTAAATTAACTGTAAAGACACCTGTAGGCTTGCCATTATAACAAGCTAAAACGCTAAACGAGGCGCCAGGTTTAACAGTACTAAAAGTATATGCTGGTGTACTAGGATTAGCAA
**CoV-bait-822**	ATCAGCTTCAAGATCATCAATCCACTCAGAAAAAGAGGTGTTATTACGCTGGTAAGGCACTCTCTTGTAGTGGTAACCACCACGGCCATACTTACGGAGTAAGATGGTTTCTTCTCCTGT
**CoV-bait-823**	TCCTTACCTTCAACACGCGCCCATTTGAGATAGGCATTATCAGAAAGAAGAGCCATCAGCATTTTACGACCCTGCACAGGTTCGTAATAAGCTAAGGAACTAGTATTACAGTTAGTTTGC
**CoV-bait-824**	CTAATAGGCACCACTTACATAGTACAGAGCGGTTCGCACAATAATTAGAGATGTCAAATGAATTGGCTCTATAATCACGTGCAACACCATCACAGGCAGAAGTGATACCAAGATAGCTAC
**CoV-bait-825**	CTACACATGGTGTCGAGTTGTAGATGGCTGACGAAACAGCAACGGACTAGCAACTCAACCACAGCGGACGAAACCTACTGTGTGGATAATTGTGAGCGACACGGACGAAACCGTAGGCTC
**CoV-bait-826**	CATAAACCATCAAAACCCACCATGGTGCAATAAGTATATATGAGATCAAAAATCCCAAATGCCAAATCCACATATATCTAACACCTTTAGTGCACAAAAAGTACAAAGTTGCATAGCCCA
**CoV-bait-827**	GAATTGACCACGGGGTCTAAAACTGTGTAATTAACGGCTGTATAATTAACACTGCAGGTCGTAAGCACCTGCGTGTTACTATTACTAAGGTTGACAGGATTATAAAAATTCAACTTAGTA
**CoV-bait-828**	TCTGAATATCTGAACGCGTGGTCACATCGATAGATTAAGCCGATCCTGAGCCCCGTTTTGCTTCCTTGAGATCGTCTTTCAAAACATGGAGATAAAATGAAAAAGCAGACGTCTAATGAT
**CoV-bait-829**	GGAAACGAATGTTGTGAGTGGGCGGAGGAAGAGTGGTGTCAGTTTGGATGTAAGCAGGAGAAAGATTAGGAGGCAGAGGACATCTTGGTCAATGAGATAGAAAGAGTAGACTGTTGCTGC
**CoV-bait-830**	GGAGTGATTGGTAAGCACAAACCAATTGTGTGGTGAAAATCAGCTGGTATTTGTCCACCTTCAGTATTACTGAAAATGTTTTCAGCAAAACCAACACAATCATTACAAGCCTGATAGCGG
**CoV-bait-831**	GTCACCACACCTTAGTACTGTTTGTGAGCTACAAACAACACACATACCCGCAGCTTGTAAAACAGTAGACTTTTCGTACAGATTAGCATAAAACTCTTCACTCCAAAATTTATCCTGGCC
**CoV-bait-832**	ATCTGGATGCGAAGACAGCTGATTGGTACCCCGGTGGGTTGACTAGCCGGTATTTTGGGGTGCGCTGAATGTAAATGGATAGTGACTTTCCTGCTGAGCTGTCGATGCTCGCGGCCTTAA
**CoV-bait-833**	TGGCATTTTTCTTGCAGTTGTAGTAGTCACCATAAAAACCAGGTCCGGTTGTCTCTATCTTGCTAAAAAGAATATCTTCAATAGCAGATCTAGATGAGTAAGGACTAGGTGGAGATGGTG
**CoV-bait-834**	GGTTTCAAAAGCTGTAAGCAATTTACTCGCAGGCTATTGTCAGGCACACCTAAAACATTACTAACTGTTGTGAGATTAAGAAGATGACTGAGTAGCAACCTTTCCAGTAATTGGATTCTA
**CoV-bait-835**	TGCCTGGCTTAGCTCTGACAAACTTATACTTGGGTGTTTCTGGGTTGTACTGTTCAACGCCCAATTGCAGAATAGAGCCTGACATTTTGTGGCTAACAACTTTAAGGTGTGCAGTACCAC
**CoV-bait-836**	TTTACCACAACTGCGTCACCAGAACAGTCTGGGTAAACGGTCAGGTTACGTTTAGGTCCGTCACTACTTGCGTTTATAGCCTTGTTAAATGCCTCAGCTAGCTCACTAGCTTGGTCGGAA
**CoV-bait-837**	TTTAGGATTGTTGGGGTTGCGTTTGGCAACATGAGGTTTAATAGAAGTGTCAGCTCCCGAACCCTTAACCCAAGTAACACGAGTGGTTGCTGGGGTATCATTAGGAGGGAGTTCACCATA
**CoV-bait-838**	GCAGTAAAGTTATCTGCAAGGGTTTGCAGAAAACTTATAAATGCTGATAAAGCTTTGGACACGCGGATCTTCATACTGGAGAAGGCACGCTCAATGATAGCCCAAGATTGAGTTTGGAAA
**CoV-bait-839**	TTTATATTACGATAGAGCAGAGCCGGTTCGGGTGCATCTTTATGAAATGCAGCAGAAACACGGCCACTATAACAGCTCCTTATCGTATAAGTCTTGTTAGTGGTAAGATCACGAAAACCA
**CoV-bait-840**	GAAAGTTCGGTTTCCACCTCGATGGGCCATTAAGTAGTTCAGCGTGGTCAATGTAGAGGAATGGTCATTAGCTCTCAGACTCTCTTCTCTGACGTAAAAAAAACATCTAATCACATGTAC

**CoV-bait-841**	ACCTGGTTGCTGACTAATTGAGATGCATGCTTTGCATACTTCTGCCTGCTGGGGAGCCTGGGGACTTTCCACACCCTAACTGACACACATTCCACAGGATCGGAAGATCGATCTTCCCCA
**CoV-bait-842**	AAATCGTTTCACGCAACTCATCAGTAATAGCCTGAAGTTGTTGTGAACGATTTGTAAGGTCATTGATCTCGGCAGACAAGTTCAAATAAGTAGCATTGAAGATGTCAAGGCCGAAATCAG
**CoV-bait-843**	TCACAACTGGCAACATGCGCACCCTTGTGTACATCACAATGAGCATCATGGTTAGTCTGTAAATTACCTACATAGCCCCACTGCTGAACATCAACAAGAAAAGGGTTGTATACGTAATCT
**CoV-bait-844**	ACAGACCTTTATTTTTAATGAAGTAGAAATTTCTTTAACTACAAATTCATCTTTAAAAGAAACATGCCTACCTGCGCAAGGAATCCTCCATGCCTGATCCAAAAGCCCTGCCTGCTTATC
**CoV-bait-845**	TAATCGGGTATTATGCTAGGTAATTCCGACACAGTTGCGTTAACAAACGTGGTGGTGCAACTTTCTATTTGAACAAAATCAGCACTAGTAGCAACACGAGGTTCATACATAGTACGAGGT
**CoV-bait-846**	AGCTGCATAAACTAGCAGATCCTTAAGAGACAGACGTTGGGAGTGGACTTTAATGTCCTGATTAATGACCACACCCAATTCCCTATAATGGTAACCAGTTGAAACAACAAATGGCACACC
**CoV-bait-847**	CTGCCAAGGATTTTTGGATTTCCAAAATTAATGTGGAAACCATCGTCCCAGCATATACCATCTATAACTTGTTGCAAACTGGTATCCACACATTTGATTTGGTCTTGAACAATGTCAAGC
**CoV-bait-848**	TAGTCTATCGGGGGCTATAACAACTATAGCAATACGGTCCTGGATAAAAGTATCACCAAAAGCAACACCAAAAGGTTCCTGACAATACTTCGTAGAAGCAGAGTCTTGCCTGTACATCCA
**CoV-bait-849**	GATATGGACTGGGCTAGCATCAGTTGTCTCCCTACGTACTCGACCATGGAAATTCTGGAATTCTAGTAACGACCCACTGCCGGTGTCAGAGAGCAATTCTAGAACGCGCAATGGGGCACG
**CoV-bait-850**	CATCATCACCACCTGTATTATTATAAATATAATAAGTGGTTCTATTAACAAGCTGAAAATAATATCCCATAGTTACAGCACTCCAAAATGGTGACATTTTAACTTGCACTTTATCCCATG
**CoV-bait-851**	AAAACTAACAATTTCATGTTCGTTTAGTTATTAACTAAAATATCACTAGACACCACAACTTTGTTACTCTCTCTAATGATAAGTCTACCTTTCTCAAGTAGAGAATAAATCATGTCATTG
**CoV-bait-852**	CATTAGCTTGGAGTAGCAAGGTAGGGCCCGTGGTCATCGCAGCTCATTGCCCAAAAGGCTTACTCGGGTAACGTCTACACAGTCCATACGAATTCTATGATGGGAATCACTTAAATGAAG
**CoV-bait-853**	TACTTTTCCCGCTTTATAAAAGGGTAATATAAGATCTTTAGGACTTTGACATTCTTCTACACACTTATCCTTTACCCTCTGGGGGTCTTTCCATTGCTATAGAATTGAATACGTTACGGT
**CoV-bait-854**	TTCTCCTTGAGGTTAGAAGAGGGAGTAGTTATGCTAGCATCCTCACAAGGCGCTTCAGTCGTAGTCTTTGCATCCACTAATGCAGCGAACGGAACCGGTGCATCCCCTGAGGGGTACGGA
**CoV-bait-855**	ACAACAGTCCAGCTTGTTTTCAAGTTTAACATATCTGGTGAGTCAGCGATGTTAAGGAAGACTTTGTCATAGTTGAGTGTGTACGACCTATTTAGATGATTACCTAATGTGGGCAGTGGG
**CoV-bait-856**	AGTATTTTTCATAGCAGCAATACGAATATAACCCTGGTCAATACGGCCTGTTAGAGGACACGTATTAAGTCCGTCTTTAAAACAATGTGTAACAAACACTACAAAATTAGTAAAATTACA
**CoV-bait-857**	CGCTACGAGGGCTAAAATCAGCGGTTGTTCTACCTTGGGTAAAATAGCCATCAGGATGGTCCTCGAACTTGCCGTTCTTCCTAGTGTAAATATACCAAGTAAAAGGTTTATCTTCATGTG
**CoV-bait-858**	CTAATAACTACTTAAACAAAAACTAATTAAAATAAAAACCAAAATAATAACTAAAACTAGCAATACCCAGGTAGCCACAAAATATGCAGAGGAGGTTACATCAACATTCTCATCACTAGA
**CoV-bait-859**	GATGTTGTATCAGCTGTGCATAAAGCCCAGCCAATTCGGCAGTTGAGAAGTTATTCCTAACGTCTAATGCACGTAGTCCTGATTTTTCCTGCATGGAGATTGGGGGCATTGGAATGTGGG
**CoV-bait-860**	GAATTAACAAGTGTTTCATATGAACGCATATCTATAACAAAAGTACCCATGGCTGCTGATTCGAAACTACCCACAAATTTATCACCTTCAAAGAGGTTCGTTGTTACCTTAAGTTTGAAT
**CoV-bait-861**	GTTAGTTATAGCGCATTAGACGTCACCCTTGGTGCAGGCCGGGGCATAACTACAGCTACACACGAACTTGAAGCGTGGTTAGGGTCATACACGTAGCCTTGGGGTGTGGGGAGACCATGA
**CoV-bait-862**	ACCACAGTTTTCCACTGATTCGGTACTTACACCATGTTGCAAAGCAACTACATTAGTAAGGGCACCAAGCACCATACCTCTTACAATATTCTTAACATTTTTAACAAAATACAGATGCAT

**CoV-bait-863**	ATTACTAGATGAACTAGTATCACAATCAGTAAGGGGTAAAACACTCTCAGCCCCACTAAC AGACTCTTCATCATTTGAAGCACTCTCGAATCCTTTGGTCACTACTCCTTGGTTAGCAGT
**CoV-bait-864**	ACGAGTACACTCGCTTTTCCAATAATTCTTAACCTCCTCAGCTTTTTCAGGCTTAGGTTCTT CATTAATTTTCTCCTCAGGAGTAACAACTTGAGCCTCACTTCTAGGTGTGCTTATGTC
**CoV-bait-865**	AATCTGGACCTAATGCATCATCAACAAACTTACCAGTATAAACGGGTTTATAAGCACCAT CAACAATAACACTTGCAGTAGCTGGTCGGGCTAATTCTGCTGTGTAATATGTTTTTGGCA
**CoV-bait-866**	TTGGTAAAGGATCCACAGGTAACACAGTGTATTTATTCGTCGTCTCCACTACAAGCGAAG ACAACAACGGCACTAACTTCTTCCAAGTGTTAGGCTTATTAACAAACAATACAGGCTTAC
**CoV-bait-867**	AACACCGTATGGGGTTCTGGGCAGGGCTCATTAATGTTCTGGCAACCACCAACAACCTTA TTATCAAAACAAACACCTAAAACAGTGAACTGCAAAATGTGGTTACCAGGAAACACAAC A
**CoV-bait-868**	CATCCCAATTTATATTCTTAACAAAGCAATCATAAACTGCCAAACAACGAGTCATCTTAGC ATCTGCTGATGCAACATGAGCATTACCATGCATATTACAATACCGGTCATGATTGCTAC
**CoV-bait-869**	CGTCGTAATCTGTCGTGGGTTATACTGTTCTCTACTAGTTATGGCAAAGGGAAAAGTTAC GTTGCCATTTTTACTCTCATTAAAACTATTTTCAAATTCAGGGCAGTAAGTACCATTACT
**CoV-bait-870**	TACCGTATGTGGTACAGTAAGTTTACGCATACTGACGGCAGAAACAGCTAAGGATTGGC CGTTTACTATAAAACCAAAAGGAATCATGAGCAACGACCTACCCAAAGCATTTAAAGCCT T
**CoV-bait-871**	GTACCCTCGTCCGTGGGCATCGGATTCTTATTAGACCAATCGTGGCCATCACAAAACACC GTACCTATTTGTGCCCTGTAACTCTGTGCAGTAGACACTAAAACAGTACTATAATTAATA
**CoV-bait-872**	GTTCTTCTTTCAAAACCATAACAGGTGTACCTTTGGCGCGCAAACCAAATTTTTGCACGTC AAATAAACTATATGAAGACAATTGCATTATAGTACTATTACGCCAAAATATATAATTGG
**CoV-bait-873**	TACAGCAATACCGATAGGGTAGCATAACAAGACAGGGCAACAAAATTATTGTCCACTGT AGGTTCATCAACACTAGGAGAGACAGTTGCGTAACTAGGACGCAACGCATCTCTCACTA AG
**CoV-bait-874**	TTAACCAACACCGCATATGTATGTTGGCGAGCTTCCTGCACAATATCAGCACACTTTATAT ATACGTGCTCTGTAAGCTGTGTATAGCCATCAGGACCTGTAGGCTGTGCATTGGTAGTA
**CoV-bait-875**	TTGGTGGCATGATATACGCACGGCTTATCTTTGCACAGTTGTTGCGATAGAAAATCGTGG CGGAAACCTTCACACCATGATATTCCACATGCCCAGGATGTCTATAATACACATCTGAAA
**CoV-bait-876**	CCATCTGTAAACAAGCAGACACCGGAGTGAATGCCTTACCACAGAAAACGCGAAGTCTTT CTTCCTCCCTGCGAACATAGTCACGCATTGCAGCAAAGGTTTTGGAACTAAGGGCAGCAT
**CoV-bait-877**	CCACGCGATGATGCTCTGCTTGAGTTGCGTGAACTACTGCGGCTATTACCACGGGAGCCA CGCCCCTCAGCATAGAAGCCCTTAGGTATGCCACCAGCTAATTGCACTGGTATAGCACCA
**CoV-bait-878**	CTCAGACAATAATGAAATACTATCAGCAAACTCATCAACAGCACGACGTTGTCTACGTGC TGGTTCAGTAAGTTCAAGTGTGGTTTCAGGTAATATTAACTTTAAAACATGATGTAAATC
**CoV-bait-879**	CAATCTTCCCATTGTTACTATTATTATAGTAACATAGAGTAGGTGTATTACAAGTCTGATC TGGACCACTATTAACAACCTGAGTTTTCAATTTAGCAGGCATTAATTCATTATTTTGCA
**CoV-bait-880**	TCAGATGTCTGGGCAAATATGACATAGTCATACTCACTGCCCTGAGACGAATCGACAGTC TGGATTTGCAAACCAAGAGAACGGCTGGCAACATAGTTCTGACTATTGTAAGGGGAAAT A
**CoV-bait-881**	TAAACTCAAAAATGGCTGAAAAGGCATAAACCATCAAAACCCACCATGGTGCAATAAGA ATATATGAGATCAAAAATCCCAAGTGCCAAATCCACATATATCTAACACCCTTAGTGCACA
**CoV-bait-882**	CAAGCCAACCACGCACGTGCCTTATTGCAAAGTCACGTGTACAAAACAGTGTATGATTGT TAGGTATGTTAACATCGAATCTGAAGCCCATGAATGAAACAACATGCTCATACTTAATTG
**CoV-bait-883**	GATCTTCTTCTGCTTGGACTCGATCTCTTCGATCTTGTCCTCGATCTGCTTCATGCGCTTAA TTAATGGATCCGGTACCCCTCCGCCTGTGCCATTGGTAATCTTGATGTAGAACTGGTT
**CoV-bait-884**	TGCTAAAGATAATTTTCTTAACACCGAGTCGCCACGGGAGCGATGCAAGCAATCCGAAA CCCTTCTAGCACAACATAAACCACAGCTAGACGTACGTAGGGAACCTGCCGCTAAGGCCT A

**CoV-bait-885**	CCTGGAAGAAGTGTGTGCTGTTCCTTGGACAGATACAAGATCTCAGTAATAGACAGCAAATTTTGGTTAGCGTGCGGTAGTGGTTTACGTTCAACCTGCCAAGCGAGGTAGTAAACGACA
**CoV-bait-886**	AGGCATTGACATTCAGCGACATATTATAATAAATAGGCAGACTATTACCTAACAAACCGGGTATTGGCCACAAACAAGCCAACAAAAGTGGTTGTGCAGAAACAAACCGTCCCGAGACAG
**CoV-bait-887**	GCATTCTGGACAAGTGATAATATGTGATTACCATTGCCACAGAAATTAATGCGCGTAGTTTGGCTCTTAACGCACTCATTGACCTTTTCGATGGCCTGAGCAGCACTAAATTTAATAAGC
**CoV-bait-888**	TAGACACGCTCTTCTTCTTCTTTCAAATCAATTTCGTTTATAACCGCCTGAGAAAAGGCTGCATTGTAAACTCCTACGACGGCCTCCTGTGAAAATATATAGATAAAATCACCATTGAGA
**CoV-bait-889**	GTATGAACGGTCTGGTCTTTGCCGACCGCACGCCGCATCCAGCGCTGACGGAAGCAAAACACCAGCAGCAGTTTTTCCAGTTCCGTTTATCCGGGCAAACCATCGAAGTGACCAGCGAAT
**CoV-bait-890**	ATACTGCACAAGGTCGAACTCCCTATAAGTACCATTAACATACAACTCACTATCCAACGCATGACACATAGTCAGCATTGGCATCATATAAGAATAATAAGAATCTGCCACAGCAACACC
**CoV-bait-891**	GTGGAACCAAAGATGTATACAAAGAAGCATTCTTCATAACACCATCTGAATAACAATAAGGATGTGGTGTACCATCACCTCTTTTAAACATAGTACACAAAGATGATAAAACACAACCAC
**CoV-bait-892**	TTATACACAACCAAATAGTTGGTGTAAAACAATGTGCATAACACAGGCATAATATACATAGTTAAATACAAATGCTTATGCTTGATCAACAACATAGCAAAGCTTACAAAACAAAGTGCA
**CoV-bait-893**	AAATTAATACACCAAACAACAACATTTATAACTACAACACTAGTATAATCTCCAAAAGCACGCTTAAGTTTTATTAAATAATAAAAAACCAAGACAACAACTATAGCCAATATAGCTCCA
**CoV-bait-894**	TCTAGTCTAAAATTACAACTTGGGTGATAAGATCCATACATAAAATTAGACTCCTTATAAACAAAACTACTCAGAAAGGAAAAATTAAAATTATAATAACCACTCTGAGCTGTTTGTGTT
**CoV-bait-895**	TCCACAAACCAATATGTTTTGCAAGGCATGCAGGCATTTTGTACTTTTTAAGATAAGCTTCAACAAAAATGCCTTTGTCATCTTTAATTGCCCTGAAACAAAGATTTTGTTGTTTAAATT
**CoV-bait-896**	TCCATATTAGCCTGCACAAAAACACGTGCGTCATCATCCAACTGTATATCATGTCGCAGCAATTCAACTTCCTGCAACACATCATAGCTTGCCACGAAGCAGGACGCGCCAAAAAGCGTA
**CoV-bait-897**	GTCTGCCAGCACGAAATTCATACCATGCTTCCTGCCACTGCCATTTATTAAATTTAAGATTAATATGCTGCAACATAAGACAAGCTACATTCACATAACAATTATTATGAGACTGTTCAA
**CoV-bait-898**	GGTTTAATACCATATCGTATATATTCCCTAACTGTTGGTGATTTAACTAGGCGTGACAATTCATTAGCAACCCAAACAACATAATCACAACCTGTCAAATACATATCCCAAACATCAACT
**CoV-bait-899**	TATAATCGACACATTCTCCTAGTGTAATATTAGCACTAAAATGCGTACTATAAACAGGAGGAGAAGTAGCAGTATAAATTCGTGACCCATCTGTCTTACTAATAAAAACTAGAAGTCCGC
**CoV-bait-900**	ACCATAAATGTTGTAGTCAACACACTCATAAAGTGACAATTCAGAAACATCAGTAAATGATATAACTGGCTTTGTTACATAAGTGTATTGAGTACCTGTTTGATAAGTGTAATAAAAGAG
**CoV-bait-901**	TACAAGGCTCATGTGTTAGTGCAAAGGATATAGGTTCTTTGTCCTTATCAATTTGGACAAACCTTCCTTTATATAAACAGCGCCCATCCGCACCAGGGTGTGGTATATGAGCACGACAAT
**CoV-bait-902**	CAGCCTTTGCATCCAAGATTTTATTTTGAACAGTTGAAATGGCAATATTTCTATTACCACCTAAACCTAATAGTCTTAAATTAGCTACTAAAACCTCAAACACATTACGTGGAGGACCCA
**CoV-bait-903**	ACTACGCAAATGAATAGCGGTGTGACGTAGCCGGGATTAATCCAAAAGAACTTAGTATAACTCACCAGTTCGGACCAGAACAATGCTAAGAATGAGCTAACGAGAAAAACATTACGTGTA
**CoV-bait-904**	TGTGAACTGTGAGTTGCAAAATAGCACCCACCATCTTTACAGACTGCACTGTCAATTGTATTCCTTGTGTTGGACAAACAACACAGAAATCACGACAGTCAGCTATAGATGCTATATGCT
**CoV-bait-905**	ATATGTGTGTTTAGGCAGAGTAACAAAAATTGTTTTCCTCTGCTGATTTGTAGTATATTTAGCTGTTGAATAAAATCCATCAGTTAAACTAAATGATGACTGCTGGCAGCGAAGATGATC
**CoV-bait-906**	TGAAGCTAAAAATGCACCAATAAGTCTTATGTGTGAGACTACTTTGTTACCATTATAATTGTAACCATCATTACAGTAATTAGATTCACTAAAATTAAATTTTAGCACCCAATTACCACT
**CoV-bait-907**	TGGCCACGTATTATTGTGACCGTAAGGGTATGATAGTCCTCAATTATGGGCCTAACAAACATTCTCCCTTTCATATCTATACACATCAGGTTGTTTGTTTCGGGGTTGAAACTCCACCAA

**CoV-bait-908**	ATACAATAGATGTCCTGCAGCTTATCATAAGTCGATACTAGAGTTGTCTCCTTATCAAGAAGAGCTATAGTATCGATTTCATACTTAATTTGGCGGCCCAACTGTTGAGTTAGTCTAGCA
**CoV-bait-909**	TAATAATCTTGGAGTGTTATGTCGAGAGCTGTTGTTTCAACTTTACATCCGCGATCCTTACTGACATTTATTGCAATGATTGGGTTTTGTGGTGCTGAGACTATTTGGTAGTTGGTTAAG
**CoV-bait-910**	TTAAGAAAACACTAGTATAATTACTTAAACACTGCCATGCCTTAAAATTTGCATAAGGACCGGCAGCGCCAAGTGTTCTATTATAAATAAACTGGTCGTTATAGTTTTTGACACCAAAGA
**CoV-bait-911**	TTGTTATCATAATGATACGCGAGCTTATTCCAAGCATCATCATCTTGCTCGGGAAAACTTCCTTCAGGTAGCTCTTCTCGTATCATATCCAAACAACAATAGCCCATTATAGCACTAAGA
**CoV-bait-912**	AAGCAGCGCCAATAAGAGTTAGCATCCCATTTGCACTGTGTTAAAAACCAATACGCAAAATCAGCAACATAGCCTCCTTCCGGTACAATTATATTATCCGGAATCTTTTTCAACAGTGCA
**CoV-bait-913**	TGTGTTGAATTATTAACTATAACAGCTGACTGGGTGGTGTTATCAAAAGATGAACCAAAAATCCAGCCTCTTATTACGTTAGACTTTTCAGTGGCAGCGAAATAAACGCCATCACCAAAG
**CoV-bait-914**	AACCCATCAGAAAAAATTGCCTGTGTTATATTGACAAGCTAATATACAGCGAGGACTGTCATCACACAATATTATATCATGCGCAGTACCATTTTCAAAGTAGGCTAGGACCTTGTGCTCC
**CoV-bait-915**	TTCAATAACTCAAATAGAGTCAATATCCAAATTAGAATGTCTAGATGCAGGCGTGTTCATGTGCTGTAATAGCAGCTAAAAGTCTGCTGTACATAGATACAGACCATATCCGAGTCTATG
**CoV-bait-916**	CAGAATGACACAATTCACATATAAAACTACCATTACAAAAATGACTTGTAAAACCTACACCTAACTTAGAATAAAAATCACAAATTGTAACCAAACCAAAAGTAGCCTTTATCCACATAA
**CoV-bait-917**	GCAGGTGAGGATGCAACAAGAAGTGCTGGGTCAGTAACAAACCGCAGTAAGTCATTTATTGTCAATCTAGTAGAGTGTGTGTTGAAATCTTTGTTCCACACTATACCAAGCTGTTTAAAA
**CoV-bait-918**	CGTACCTTTGTTGGTGTTATGCCTGCTGACTTGCGTGAAATCGTTGTTTCTAAGTATGGTGCTATTTACCTTAATGGTTATAAGCTTTTGAACCTATTGCCACTTGAAGGTGTGCTTATT
**CoV-bait-919**	GATATCGCACCAAAATTCGACGATAATTGTTTTACTAACGTATTTAACGCTTGCGCATTTTGATTTACTACATCTTGTAATTTACCTAACGCCGTCGACGTCGTCGTTAACGATTCTTGT
**CoV-bait-920**	GCTACAGGCCAGACTGTTACTTTGTACTCAACAAACGGCAAATCTACATTAAAACCTAACTTATCATTCAATTGAGCACAAATATCATGTCCAACTAACTTAAAGTTAGTATAAACACCG
**CoV-bait-921**	AATTACAATGAGCCGTGCAAAATTTGTCAGCAGACCAAGACATACCAGGTAATGGTGCTGTCATAGACACAGAAGAAGCAGAAAAGTTCTTACTCCAACCAATTGTACCAATAGAGCACT
**CoV-bait-922**	GTGGTGAACCATTACTCATAGCATCCTCATAGTTCTGTCGTGCCGTCTCATAAATAATATAAGACGGCAAATTAACATATGTTGATGCCACGCTCTGTAACACAGCATTATCACTAAAAT
**CoV-bait-923**	CGACTAAATATTTCATTAATAGAAGCAGATATAGCACCAAATGAATTACCTAACTGGTTCACCAAAGTCTGCAATTGCTGAGCATGCTGGTTAATAACAAGCTGCATCTTACTAATCGCC
**CoV-bait-924**	TTCTGTAATTTACAGTCTAAACCCATTTCCACAAGTTGAGACTGAAGCTTTAAATACTTACCCATAAAGCCACTAAATTGGGCTAAAGGAAAGTTTATGAAATGATTAGGCTTGTCAGCA
**CoV-bait-925**	TAACAAATACAACAATATCAGTAAAATTACAATGGGCTGTACAGAATTGTGAAGTGGACCATTGCATACCCTGTAAAGGTGCTGTCATAGCTACTGAAGACGCGCTAAAATTTTTACTCC
**CoV-bait-926**	CCTTGACGGGGAGTTCGTAGAACCAGGGGCGTGGGCGGCCGTTGACGATTTTACTTGTCCAAGACAGATGCGCCTATCTCCTGACGCGGAGAAAGCAGGCGTTCAGCTCTAACGATAACC
**CoV-bait-927**	CAACTTTTAAGAACATCTTTACAACCATACTTACTTTCAGTAATGTCAAAGTTGTATCTCCAGAATACGTTATCAGGACCATATAGGTAATAAAAACCATTCTGAAATTCAACCTTGGTA
**CoV-bait-928**	CGTATTTTCTCAATGCAGCAATAGCCAAATATATCGCTAAATTTGGGTGTTGGAGGCTTATAACAAAGTCTCTCACCACACCAAACACTTAGCTCACTACAATAAAACCCATCTATTTTG
**CoV-bait-929**	TTTTTCTAAGTACCGCGCTCGCTCCATGGATCCGGTGGTCCGCAGTCGTTCTTCCACCTTTAAGGACTAGGCATAGCTAGCATTACAACGCCTCAGGTAATGCGCAGTGCGCCACATCCC
**CoV-bait-930**	TACGGCTTTAACATATCTGTCAGACAAACTTTGCAGTTCACCACCAGTATGTTCATCTATAGCACGTGCAACACCACCACCATGGGACAATTGCTCATTTGCTGCGTTTACAACAAAGTC
**CoV-bait-931**	CATTTGAAATATAAAAACTTTTGTCCTCCACCTTCGTAACCGTTAACATCAAAGCTTTTGCTGCTGGTGACAATGCTTGTTCCACATAATCTGACTTAACAACAGAAACAAGTATAAATT

**CoV-bait-932**	AAACCTTTCGGTTTTTCACACTCAAATTGTACACTCATTTTTTCTTGAAACACAGGTTCATCAAGTACAAAATTGTTATCACTCATAAGTCTGTAGACACCTCCGCTATAAAAGAACGAC
**CoV-bait-933**	ACCCATAAGACAGCCATTGTCAACATCTCGCATTAGGTTTTTCAACATATCGTCCCAGCCACCATAAAACTTAGTAGTGCCAATCACAACTGTAGCATTACGTGTGGCAGCAATAGATTT
**CoV-bait-934**	CACATGCATTCTTAACCTGATTTACATTGGAACCATTATTGTTAAAAACAATAAAGTCTGTGATTATGCTACAATTTTTAAGTGACTCTTTGCAAGTGTATTTGTTATCTGTTATGTCAA
**CoV-bait-935**	AACATAAGTCAGTATTACCAAAAACTGCATTGATAGCAAACACCAACGACCTGCCAACAAGAGTGACATACGCAGGTACATCAGGGACAGGTTTCATACTATCAACAATATCAAACACAG
**CoV-bait-936**	TTAGCATATGCTGTAGTACCATCACCGCTAGTTGTACCACCAGGTTTAAAATAAAAACCACCTGTGCAATGCACAACTTCTGTGAGTACTTGAGCTAACTCATTGGAGAGGCGGTAGAAC
**CoV-bait-937**	CACGAAGCAGTCATAAATTGCAAGACACCTGGTCATGATAGCATCACCAGAGGCAACATGCTCATTTCTATGAACATTACAATGCTCATGGTGATTAGAGCTAAGAGAGCCAGTGTAACC
**CoV-bait-938**	AATAGGTGAATGTCAGGGTGATGTCTCCATTAGCCTGGAAATTGCTAGAAACATGTCCAGCAAACATCAGTTCCTGAACTCCTGGTGCATGACGAGAAAGTGCCATCACTCTAGGATCAG
**CoV-bait-939**	CACCATTGGCATGTACGTAGAAGGATTTGCTAGTACCCTGAAAGATAGTCTGGACAGGAACGCGCTTAAGGCGAGCACTCTTAGAGCAAGCCACACAAGATACCTTATCGCAGCCAAGGA
**CoV-bait-940**	TCAGACCTCATTTCGAGGTCTGCAATTTCACTGGATAAGTTGAGATATGTGGCATTAAAAATGTCAATATCAAACTTAGGTCCAGTGTTGTTTTGCAGCTGTGAAAGAATATCTTCCAGA
**CoV-bait-941**	CAAGCATTGACTTGTAAGCGTCAAACATTGTTTAATTTCCTGTGTCGAAGATCTCGTTGATAATTTCAACGGCCGTGTCACCACCGTCTACAGCTGTGTCCCATTCCAAATTGGCATGAG
**CoV-bait-942**	TTACTAACAACACCACCAGCCTTCTTTTCCACTAACAACTTCTGAGTATAACAAGCAATAACTTGTTTAGCACCAGACCTCGTTATGAAAAACCTACCTCCTGACTTTACAGTTGCCGAT
**CoV-bait-943**	GCATGGGAGCACGCAGTGAAAACAATGCGTGCTCCAGGATAATAAAGACCTAAACCTATGACACAGTGAGACTTACCACTACCAGGGGGACCTTGGATGGTGGTAATCTTCTGGGTTGCG
**CoV-bait-944**	GCAGGTTCTAACAATATGGGATCTGACCAATCGTCAGTTGTGACGGTGCCACTATTGGTTACAGGCACACTCGCAGTCTTGAAAGAAAATGCTTCACCATATTCAACTACAGAATAAATG
**CoV-bait-945**	TGAGATGTTGAGTTGCTAGTGTCAATTCATTGCCTAAGATGCATCCGGTCATGATGGTGTCGACATCAGATGTGTGTGCTGCACCATGGTGGTGATCACAAATTTCAGCATGGCGTTTGG
**CoV-bait-946**	GGAAACGAATGGGATAATATGCACACCAAGTGTGACATAATGATCACCACCAGATAACACCAACCACGGTTGTTGGTGATAATACCACGGCTTTCCATTTATGAAAGCCAATACAGTGGT
**CoV-bait-947**	AGAAACAAATGGAGTCAAAAGACAGGTAATTATTAAGGTTGTCAAAGTCAAAAGGACAATTGACATTAATAACTACACCTTCATAAGTGTTCCCATCAGTAGCAGCATTAGTTGAGAAGA
**CoV-bait-948**	ATTTGAGTTTGCATACGGTTGTCATTAAATGTAAGTAGAAAGTTTACACCTTTAAGTTTGGCAGTTTTAACAACATATCTACGTCCTTCTTCAGACAACATGTTGAAATCTTTAGCATAC
**CoV-bait-949**	GTCGGCTGATATGGACATTTGTGAAGCGTGATGGGATGCTACGGTGCGGGTGTCATCGGAGAGTGAGATATCTTCGCTACTATCACTAATTGAAAGCGGTGTTGGGGCGCGTGGTAGAGT
**CoV-bait-950**	AATATGTACGTTGGTCTAGGCACAGCAATATCTATAAACTCTACTTATACTAGCTTATAAACAGGCTTACCATTCTGTATAAGAGTAGCACCTTGTCAATCCGAAGAAAGGGTGCGTATT
**CoV-bait-951**	TATTATACTGATCTAGGCCAAGGTCAGGAAGTGTGTGATTTGGTAACTGTTGTAACAAATCCTCTAGAGTTTTGTTAACATCTATGTAATCAGGTATTACAGTGTTAACATCAACATCAG
**CoV-bait-952**	GCAATGTCAGCAATAGGACAATCATAAAATGAAAACTTACCCTCAACTCTGCAAGGTTTAAGTTCCCTTCTATCCACATGTGAAAATGCTTGTTGTTCTGTGTTTTGTGACACAGATATA

**CoV-bait-953**	AGAAAAATGTTTGCGTAAATAGCAATAATATTTATCTATGAATTGTTCGTCAACACTTGATGACCTGTAGCAGTTGTCATACAACTCTCTTTGCAGAGTTTTGACACTAACATTATTACA
**CoV-bait-954**	AATTGCAGTGTGCAGTACAAAATTGTGATGTAGACCAAGTCATACCGTCACCCGGTGCAGTCATAGCTATACCAGAAGCATTAACTACACGGCTGCCTTGGAGACTACCACCTGTACACA
**CoV-bait-955**	GCAAAGAACACAGGAGTCACAAAACCAGGACTAACCCAAAAGAATGCTGTGTATTGTAAAAGTTCAGACCAAAACATTGTAACAAAGAGGCCCACAATGGCTAAGTTGCAAAACGCACCA
**CoV-bait-956**	CTTGAAACAATAATACTCATGAGTTGTACTATTGTAACAAGTAGACTCTTTAGAGCAGTGACAATGCTTGACTTGTCCTACTTTAACAAAGTATCTCATGGTAGTCAACTCCAAACCACC
**CoV-bait-957**	CTGTAGTGGCGTTTGTTAAGGGGAATGTGGATGCTGCGATTTGTTCAAAGCTACCTGAAAACCTAAGGGTACCACCGTCATTGTATACACAACCATGTCCTGATGTTGAGAATAGTCTGT
**CoV-bait-958**	TCTGAAACATTAAAATCAGCATGTTCACTTATGAAAAATGTCACAAGTGCAAGTAAATATTCCATAGCTTTCTCAGCATCGTCACACAGGTTAATCTTATTATGAAGATCAACACAATAC
**CoV-bait-959**	GCGCACCAAAACTCAACCTCCTACTTTCAGACAGCTTAGTATATATAATGGACAAATACCAAGCTGTATAAGTTTGCACATAAGCCTGAAGTTGAGTTGAGCTGGCAGATGTCATATTAA
**CoV-bait-960**	TAAGCTCAGAGTTGGCGTAACGCACAGGATAATAGCGGTCCCACCTGCAAATACGAATTTTAGCACGATGGTCACCATTCTTCTGATGAAAATACATACCCCAAGAGCTATTGCTCTTAG
**CoV-bait-961**	CCATAACAATTGCCAGCATCAGTAAAAACTGCCTTAATAGCAAAAACTAGTGTCTTGCCTACAAGTACAATACCAGATGGCACACCAGGAACGTTACGAACATCATCAAGTGTACCGACA
**CoV-bait-962**	TTAAAGTTAGCTGCAACACCAAGGTCTAAGGTTCTTTCTTGAACAACAATCATACTAGTAAGCTCTAATGCCTCTAGACGAGCATTTAATTGCAAAGGTTCCTCAACATTTCTGCAAACA
**CoV-bait-963**	TTATCAACTGCACTGACTTGCTTACTTTCAGCAACTACTGACTTTTCAGTAGCAAGCTTTTTATCAGCCTTTTTAGCATCCGCAGTAGGCTTTTTCTCAACCTTAGCTTCAGCAATAGGC
**CoV-bait-964**	TGATTTGCTGCATAATCAGTCATAGCCTTAGCCAAATGAGCAAAACAAGCACATCTGTAATCAGCTTCAGAAGCACTGCCACTATAATATTTATACTTGTTAAACATAGAGGCATGTTGC
**CoV-bait-965**	TTCAGCAATCCGAGGAAAGTTAGGTGTTTCAACACCCATTGCAAGCACAGAAGCTGATCCACAGTTTTTCTCAACAGATCGTTGACCAAAGCATGTGGTTGCGTTCTCAGTTCTATTAGG
**CoV-bait-966**	GTAAGATAAAATTGTCCCTATATTGGACTTCCCATTTCTGCGCGAGTGTTTGCAAATATATTACATACTTTTGCGCATCCAATCCATAGTATTCAAGAACAGCTTTGTCAGTTGCTGGAG
**CoV-bait-967**	CACATATGTAGGGGCATAAGCAGGTGGTAGTGGTTCTTCCACACGACTACAATCCTTAAAAAGACCACATAAATCACCAGATTGCAGATCAGTTAACTGAATCTCAAAGAATTTCAGTGA
**CoV-bait-968**	GATATTGGACATCTGCTTGCTGCACACGGAATTTATCCGGATCGGCCTGATAGCAATGTACTTTCAGCTAGCTTTCAAAAGGATTTCCCACCACGTCTAGCTAATCGCGTGCCGAGAGCG
**CoV-bait-969**	AACGTTATAGGACTTAACCCCTTAATAGCACTATTAGAGATCAACACGCCATCTTTACACATTGTAAAGCGCTCGTATGAACCAACTATGCTATTATCATAGCACACAACAACATCATCA
**CoV-bait-970**	GAGCATCTCCAGTATCGCCTACTTTAACCTTAGCGTAAGCGATCATTAAAGCCATAAAGCGGGCGGGTTTGCCTGCTTTAAGCTCATAATAAGCTTCTTTGAGAGCAGGTGTCACAAACT
**CoV-bait-971**	GAGAAGAACTCAATAGCCTCTTAACAGCGGGACACATTGAAATCCAGTTGACCCAAAAGGACCGCCCTACCAAACCGCCGAGCCACTGTCACAGCTACTAAGGCTATGGGCCTCGAGCCA
**CoV-bait-972**	ACATAGGCAATATACCAAATCCATGCGTACCTAAAAGTGCGTGTAACGGCAAAGTACAAGAGGGCATAGACAATCATAAATAGCACATTTTGGGTAACAACGTATGACAAATTGTTTACC
**CoV-bait-973**	GAGACAGACCAGTTGAAGTGTTAGAGCAAGAGCTGGGGATAGCATGAAGAGGAAGATAGTTGCAATTTGAATGGCAACCACCATCAAGACTGAGTTAGTGTAGAATTTGAAGATCTGGAG
**CoV-bait-974**	GGTTTTGTAATAACTTGGTGTGCACAAGTTTCTTTAAAAATACATCTAAAAGAAACAACGTCAAGTTCACCAATACCAGGAATGTGGTAAGAAGTAACACCATCAAGTGAATAAACAACC

**CoV-bait-975**	ATTAATAGGAAACGTGCTAAAGTACCTATAGCCATTGATATAAAAATGACCATATTTACTT ATAGCAATTTCTCTAACACTAGGTGGTAGTGAACCAATAAATTTAACTGCTGTGTTATT
**CoV-bait-976**	ATACCGTTACAATCACCCATTTTGACAGCTAACGTTGGGCCAACGTTAGTGCGACTTAGA CATCCGGTGTTTCCACAACAGCTGCCTCGCGCTTCTTACGCCTGCGCTGGGATTTGGGTT
**CoV-bait-977**	AACCATATTATAGAGTTACAGATGTTCTTGGAGACAACGTCACCAAAACTGAAAAACTTT TCTGAAGCATCATCTAGCTTTTTAACAATTTTAACTGGAGTGTTACTATTAGAAGTTTGC
**CoV-bait-978**	CAAGCATAACGGCACCCTGCTTAATCAGACCGGCAGACTCAGCATCAATGCAAGAAGAT GTAATCTGATGCACAGCTGCACAGGAGCAGGTAACACAGCCAAGGTCTTTAACAGGAAC CC
**CoV-bait-979**	ACGACAAACATTACCATAGACAGACCTACCCTCTTTCCAAGTGAAAAAGTCATGCTTTGC AACTGCACCACAATCCGAAATCAGATTATAAATGGACTGCTCGTGTTCCATAACTGACTT
**CoV-bait-980**	GAGATCTTAATGGTCTTCTCACCACCAATACCCATAAGCCTAAAACTAAGCAACAGAGAA TCAAACACACCACAAGGGGCTCTAAGGTCATTAGCAACCATATATTTAAATTCGGCTGCT
**CoV-bait-981**	TCGACATAAGCATCAAACATCTTAACGGAAACTTCAGTACTATCTCCAACGTCTGATACAA GAGCTTGGTCAAGCAACAGAATAGGTTGGCACATCAGCTGACTGTAGTACACAGAAGCA
**CoV-bait-982**	GCTTTGTTAAGAACATCAAGGAAAGCTTTTACACATTCCTTGAGATTATCTGAAGCCACTT GTATTTGACCCTTAACAATGTCAAACACTCCTGTAACCAAAAATTTTAGAATTTCCCAG
**CoV-bait-983**	TAATAGTTAGTTCGTTTAGACCAGAAGATCAGGAACTCCTTCAGAAGAGTTCAGATTTTT AACACGCGAGTAGACGTAAACCGTTGGTTTTACTAAACTCACGTTAACAATATTGCAGCA
**CoV-bait-984**	CCGGCATCCAAACATAATTTAGCAACACTCTTAACACTATTTTTAGCAATAGTTGTAGGTA GTGAAGCTCTAATTCTAGAATTGGTACTTTTAGTAAAAGTACACAATTGGAACAATAAT
**CoV-bait-985**	TTTCATGTTCGTTTAGACTTTGGTACAAGGTTCTTCTAGATCCTGGATTTCGAGTGAAAAC CAAAATATAATAAGCATTATTAAAACAAGGAATAGCAGAAAGGCTAAAAAGCACAAATA
**CoV-bait-986**	TGTGATGTCACTGATGTGACACAACTGTATCTAGGAGGTATGAGCTATTATTGCAAGTCA CATAAGCCTCCCATTAGTTTTCCATTATGTGCTAATGGTCAGGTTTTTGGTTTATACAAA
**CoV-bait-987**	CAAATAATACTTCTATTACCTAAAAACTAACCCAAAAACTTCACTTCCCTACAACACTAAC AAAAAAAACATCATATAAATTACAACTAAAAAAACCTTAAATACACCCAAAAACCACAT
**CoV-bait-988**	TACTCAACACACACAAACACTATAGCTCTAGCTAAAAACATGATAGTCGTAACGACACCA GAATAGTTAGAGGTTGCAGAAATAACTAAGGCCCACATGGAAATAGCTTGATCTAAAGC A
**CoV-bait-989**	TCCGTTACATCACAACCCGGCGCATTACATACATACGGATTTACCGATAATACTAATTTAT GCGACGTCGATATTACATGATCATAACAACATTTACAACATAAAAACGGACGACGTATA
**CoV-bait-990**	TTGCTAGGTAAACCTAACTTTAAACGTTGCATATCCAAATTACCATTGGATTCACAACCTA TGTTAGCCCTAACAGAACTTAAACACAAAGCAATGCATAAAATGAAAAACATTTTATTT
**CoV-bait-991**	GCCAACAGTTTTATTACTGTAAGCGAGTATGAGTGCACAAAAGTTAGCAGCATCACCAGC ACGGGCTCTATAATAAGCCTCTTGAAGTGCTGGTGCATTGAATTTGACTTCAAGCTGTTG
**CoV-bait-992**	ACCACTATATAATTTTCCCTCACAAACTACTAATATTATCAAATCAATTTTTACACACACAC TTAATACAACAAACCACTCAATTCCTAATTTACAAAAAACAACTATCACCATACTTAA
**CoV-bait-993**	AGGCCAGCACGCAGCCCATGAAGTCGTCGGGCAGCTTGTAGTTGTAGTCGGCGATCACG CCGGTCTGGCCGGGGGCGATCTGGCGCACGTCGTCGCCCTTCACCACGAAGCTGTCGGC GT
**CoV-bait-994**	TAATAAGAAAATAAACATGTTCGTTTAGTTGTTAACAAGAATATCACTTGAAACCACAACT CTGTTGTTTTCTCTAATGATAAGCCTACCTTTTTCCAGAAGAGAATAAATCATATCATT
**CoV-bait-995**	GGTGCCATTGCAACTATATATGTAGCACTCTGGTATCTCAAAGTCATACTCAGCGGCGGC AACAGCTTGACGCAGCTTTTCAATTTTCCCTTGCATATACTCTTCAAAATCATCAAAATT
**CoV-bait-996**	GTCATAGCCACGGAAGCAGCACTGAAATTCTTACTCCAATAAATAGCACCGGCAGTACAC CCAGTCGTGCCTGCATTACTAGATTCCGTAGAAACATTTACTACTTCATAAGCACCACCA
**CoV-bait-997**	CAGGATTACTTGTGTAAACAAACCATAATATAAGTAAAGCAGTAAAGACCACCTTACATA ACAAACTCTTATAGCTAGCAACTATACTTTTAGCGCTAGCTTTAATTAAGTAGAAAAAGA
**CoV-bait-998**	ACAACTCTGTCTGAGACCACTTCAAGCCATAAAGAAAATGACCACAATGATCAGTGTTAT TACAAATAGGAAAATAGTGGTTAAGTTTGCACTGACTACTCCAGCAATTCGCTTCGCCTG

**CoV-bait-999**	CAGCTATTATAATAGTCAGCCTTGACCTCTTTAAAAACACCTTGACCAGTAATACCATAGAGGTCATATCTGACGCAAACGCCAGTGGCCACTTCAGTATTAGGCAATTGTAAATCTGTG
**CoV-bait-1000**	ATATGTATAATAAGTAGTGCGGTTGACAAATTGCATATAATATCCATAGTTAATCTTATTCCAATAATAAGAGATGCTAGCTTCAACAACACTCCAATCATTTTCAACCCACACCTTGGT
**CoV-bait-1001**	TATCACCACTAGTGTAAGCCACCCAAACATTCCAAATAGGAATGTACTGAGACGAGGCTCGTTTATATATTCCAAGTGTGCAACCCTTACCAATGGTGCCATTCAAAGAAAAACAAATGG
**CoV-bait-1002**	AAACAGTGAAATTTGATGGAACAGAAATATTACCCATTGAAATAGCAGGATCTGGTTCACCACGAACCCTAGTACTATTAACAAAAGTAATACCGCCATCATCACAAATACCAATAGATG
**CoV-bait-1003**	AGCTATTAGTTGACATCAGTCCAGGTACAGCCCAAAGACACATAAGTAGCACAGGCTGTTCTGAAACAATGCGGCCATCAACAATAGTAGAACTATTAGTAAGCAGGAACCAGTTAGAAA
**CoV-bait-1004**	GAGACAATGGGTCATCACAATAAAGCAGAGCTTTAATGGCAGTACTTTGAACTTCAGCAAGGACTTCCGTAAAGTTAGAGAGTGCAACGGTCCAAAGATCAGTTGCACCTCTATTAGTTA
**CoV-bait-1005**	ATCTTTCAAAGCTATAACCACCGTTGCTTTATGCCTACACTCAAACTTAGAAAGATCTAAGACACTGTTGTAGGACATCGCCATAATAGTGGAATTACGCCAAAAGATATAATTGGCATG
**CoV-bait-1006**	TAACTACTGTTGTCATACCATTATGAACAGCCCACTCATTAAACCTGTCTACAGCAATCCTAGAAGAACTAAGCCACCAGGTAGAACCATTAATGAGTGCTGCATAAAGAAATGCCAACA
**CoV-bait-1007**	GCAACGTAGTGAACTATCACCAGTATGCACAAACTCCTCGACCTTAAGAATGCCCATCTTACTCAACCTAACCTGAGAAATAAGTAAGTGTAAACCACCTAATGTGGTTTTAGAGACATC
**CoV-bait-1008**	GCCACTTGCATATACGAAGCACAAGGTATTTGCCATTCTGCTGAGCCATGTAAGCACTATACTGCTCACCACCAGCAGAAGAACTAACACCAATCTCAAAGGCAAGGGCTTTGGTTCTAT
**CoV-bait-1009**	ACATTACCAGTAGTAATACTAGTAACGCCAAAACCATCAACGCAAACACGTGCATCATAGATATTGGTTTTGTAATGACCACCATCAGTACGACCACTGTAAACACTGGCACTAACAGGG
**CoV-bait-1010**	GATTGGTATCAGGGCAATCCACGCAACTCTCGGCCTAAATAACCTGGCGAAATAATTGGGCAGGGTGGATTACAGACTATTGCCTGCGAGGTGAGAGACAGGCTCGACGAGTCTACACCA
**CoV-bait-1011**	TGCAGTTGCATTGTAATAGGTTACTGAGCAATCTACAAGAGGTATAAAATCTGCAGCACGTGGAACTCTGGGTTCATACATGTTGCGAGGCGTTACTCTCCAGGAATTATTATACTGGTA
**CoV-bait-1012**	GATGAAAATTTGGGGCAAAGCTGCTGAGGAATATCCAGACTCTTTGGTGTGGAAGGGTTGGTGCAATCAGATCGACTATCACCAAATCCGCACCAATCAGATGTTATGTGGCCTGGACCA
**CoV-bait-1013**	AACAGGTAGTATAATGAGGGACAATGCCAACATATTGATGTTGGCTGCCATACAAATGTCTACCAGGCACATAATAGTCAGACACAGTACATAGCTTTTCTGTATTCCAATCATGTTCAT
**CoV-bait-1014**	GCAAACATAACTCATTATTTTGTATATTAGGAATACCAACCTTAATAGTTTCAACAACTACAGGATATTCTTCTGGTATACCCTCTATAGGAGAATTATCCAATGAAGTAACTTTAAGAA
**CoV-bait-1015**	AATCGTCGATATTAGGTCTGCGCAGACTTAGAACCGCAGTTAATTCATATAACGAGGACGGTCAGCATTGGTCTGAACACCAGCGGTTCTAAGCTCTAGGAGCTCTCAGACAAGGTGCTT
**CoV-bait-1016**	CAGAAGCGAAAGCAATGGTCTGACCACCATAAACTAAGTAGTGATCACCACCATAAACGGCAACGAACTCCTTTCCAAACTTCTTTTTATCAAAATACCAACATTTGCCCATGGCGAAGC
**CoV-bait-1017**	CAAAACCTATGCAAACAAGAAGTTTACCAGTCTTGAATTCTACTTTAAGAGTTACAGCAAAATAAGTGCGGTCTAATTCGTCAAGTACAGCGTCTACGGATGTATCAATAGATTTGACAA
**CoV-bait-1018**	CAGCAAAAAGTGAAAGTTCTTCAAGAGCTTGTATAATACAATTTACCACACCAACAAGTGTGGGTAATTTATCACACAAACATTGTAAAAATGTCAAAAATCCTGTCAAAGTGTCTGAAA
**CoV-bait-1019**	AGTGAGAACAGGATTAGAACAATTAGTATTGTTAGAATAATAATAAAAAGTTGGAGTAGATATCAAGCTTTCAAAACCAAACTTAGATGAATGGTTTTGAGAAGCAATAGTACCAATATA
**CoV-bait-1020**	TAATAGTGATAGAATAAGTCCAGGTTAAAGTTACTGCATCAGCCTGGAATTGGTGACTAAGATGCCCTGCAAAAAGCATCTCCTGAACACCAGGAACCTGACGAGCTAATGCCATAATAC
**CoV-bait-1021**	GAGCTACAACATTATCAGTAAAATACTGTGTTGGCATTTGTTGTTGAGCAATTTCTTCATCTATATATGGGCCATAAAACACACCATGGAAGTCAGTACCACCATGTGTTTGATTATTAA

**CoV-bait-1022**	TGAAATTGGAAGGAATTGAGATGTTACCACTGGAAACAGGATTAAAATGTGGTTGGACATCGGTGAGACCCAGTCTGGTAATAGAACCGTCAGGACACACACCATAAGAAGAATAAGTCA
**CoV-bait-1023**	AACCTGTTTTAGCATAATAATAAGTAAAAACTTGTACACCTGCACGCAACAATATTGTAACTGCAGGTAACAGCAACAATGGAGCCATTGCTAAAAGTATAGTTCTAAAAGAATAAAACA
**CoV-bait-1024**	AGCAAGTAAATACAGAATTACGGTTTAACGGTGGTTTGGCTTTTCCACTTTCCCATGAGAGCAAAAGTTCCTTAGGACCTACAATCTCCTTTAGGGTGGCATAAGCATATGATGACTTAA
**CoV-bait-1025**	CTGAGAAATTACAGTGAGCAGTACAAAATTGTGATTTTGACCAAGACATACCTGCAGCAGGTGCAGTCATGGCTACAGAAGCTGCAGAAAAGTTTTTGCTCCAGCCAATGGCACCCACAG
**CoV-bait-1026**	CAGAATCATCATTTGCAACTGCTACAGTTGCCTCTTCAACAGATGAAACATCTTCAACATCAACAGTTGAGACACTCTCTTCTTCAATTTGTTCAACATCTTCGTTGTCAGAAATCGAAG
**CoV-bait-1027**	CCATCCTAGGGCTGCGAACGGCAATACTATACAGCAACCCTTCATCCCAAGATCCGCAGCTTCAGCGCTCCTTAGCGCGATGGCGCTCCTGCATACCACCGACACTGTACTGTTGTAACG
**CoV-bait-1028**	AGCCATATCTGAAAGACTGAGACTTAACACACTCATTGATTTTCTCTTGGGCAAGCTGACGTGAAGCACGAACATCAGTGTACTTAGTCAACTGCTGAGAAACAAAAGCATTAAGAGCGG
**CoV-bait-1029**	GTGCAAACTATAACATCTAGCTCTTCTTCTACAACAGGAACAAATTCCTGTTTATGAATTGTTACCGGCTCTTCAATCGGCAACGAAACACTACTGGAATCAGCTACGTCATTCAAGGAC
**CoV-bait-1030**	CAGCATCACCCTTTTGTGTAAAAAAGAAGTGCTTCAATGTCAATTCTGAACCTTCATCGAAAAACCCTTGAGAGCGTAAGAAGTCATAAAACTCCTTATTAAAATGGCCAGGTTTTACTG
**CoV-bait-1031**	AGCTTTTGCAACATTCATAGCCTTTTTCAATTGTTTGATTATTTGTGGTGAAGAACCATTTGCAACAGCATTTTCATACTCTTGTCTTGCTGTTTCATATGCAACAAAAGATGGCATACC
**CoV-bait-1032**	TACTTAAATTCAGCTGGACTAACACAAAAGTCATAAACACCTAATGTGCATTTACAGAATCTGTTAATCCAGTACAACAAACCATAGTACATACAGCTAACAAAGCCTAACAACATATAA
**CoV-bait-1033**	CACAATAATTGATGGCAACAAAAACACTTGAAGGAACAACACCTTATGTTTAACAAAAGATGTAAGACAGAGTGACAAAGCAACAAGCAAAATCATAAACGGAGTGAGAAAACCAGGGTT
**CoV-bait-1034**	AAGAACTTTTGAAGCTGCTTCATCAAAAGTTTCTGCATAATAAGAAGGATTTAGACTACAAGGAGTCAGAGTGTGATCACACATTTAAGAAAATGAGGCTGCAAAGTCGGTGTCGTCTTC
**CoV-bait-1035**	GAATACTGAGGGGTGGTGTTAAACCAACCTTCCTCTTTGCGAAAAGTTCAAATGCTATGTTTGTGGGCAGTGATGTCTTATTAACAAAGACTAAATTGTCAGTGTTACCATCACGTACGA
**CoV-bait-1036**	ATACACAACGTGCTCAAAGTTAAAATCTTCGAGACCATACTTTTGAATAAATACACCTATATCCATATTCAAAAAGTCTTCTTCCATTGTGCTGCGAGGCAAAAAGTCTTGTAAATTACG
**CoV-bait-1037**	GCGAAGAAGAAGTGTTTGAGCGTTAAAGACGAACCCTCTTCAAAAAACCCTCTACTTTTAAGAAATTCATAAAATTCCTTGTTAAAATGACCTGGTTTTACAGTTTGATGCGTTAAACCC
**CoV-bait-1038**	CTTGTTGAATGTGGAGGTCGCTAGATGGTCATAATTGGTGACAACCACTTGGTTTGCATTCAAGCAACCACCATCGTAGCAGTCAAAGTACTTGTTGACAACCTCCATGCAGAACAGGAG
**CoV-bait-1039**	ACAATATCTTGATGACCACCATAATCATCACAGCAACTTCCACACTTCTTAAAACAACTAGATCCACATCCTGTACAACAGCATACAAAGAATAGGAGTACTAAGACAGCTACACCTGCA
**CoV-bait-1040**	TGTATTTTCACATGTAATACTTTTTCTAAACCACATTGAACTACCCACTTCACCCCTAAAAAGAGGTAATGACCATTCAGGTAACTGACTATAAGAAACAACAGCATACTTTCTACCCCA
**CoV-bait-1041**	AAGCGCATTTTACAACCTACTTGCTGCACACACTCTACAACATAGTGCGTAACATCAGTTGGATTTATAGTGCGTTTGAATTCTTTAGACAATTCAGCTGCAGCCTCTACGGTTATAAAA
**CoV-bait-1042**	TTAGCCTCCACATTGTTAGTGATAGCCTGTCCATTACCTGAACCATTAGACAACATTTTGACGCAGTTAGACAGTGGTTTAACACCACTCTTTACTGCATCAAGATATGCCTTGGCGGGG
**CoV-bait-1043**	GGTTAACACCAGTACCACCAGTATTATTATAGGTGTAATATGTGGTTTTATTAACAAATTGAACATAATAGCCATAGGTATCTTTGGCATTCCAATTAGATGAGATTTTAACATCGACCT

**CoV-bait-1044**	CTTAGAAGCTATCCACTCCGGGGATGCATTGTCTATTCATGCACCATTGTGGACCTTCACATTTACAAATATCGTCACACTACTGATATTAACCATGACTTCTTGAAGAAATTTAGAGTC
**CoV-bait-1045**	AAACTATTATTTCTCATAGCAGAAATACGAATATAATTCTGTGGAATCATACCTGTTAAAGGACATGAACCAGAACTACTTTTATAACAATGTGTAACAAATACTACAAAATCAGTAAAA
**CoV-bait-1046**	ACCATTTATGTTAGGTTGGCCATTAACATCATAGGGTTCTAGTAGTGAAGTATTAAAATCACCACCAAAGCCCCTAAAGATGGGTTGTGAACTAGCCACTGATATAGCTTGAAACAAACT
**CoV-bait-1047**	TTGCTGACAGTGTTGCATTCAACACTGCCATGACCTGGCCTACCAAACCGGTGATGTTCTTAACAGCAAGAACAACCTTACCCTTCACCACTTTGTACTTTTTGCGTACCAAAAGGTTAG
**CoV-bait-1048**	GCTACATTACTTAAAAATGTATTAAAAGAATCATTAACTGTACAACCAAATATTCCAGTTGAAAGCATTGATGTTAAAATAGTATGCTGGTCTTCACAGAGATCAAAAACAGATCTCCAA
**CoV-bait-1049**	GCACAAGAGAAAGGAGTTTTGTTAAATTATAATTACAATTAGTAAAAACTCGCCTACTGTAATTAGCTATTATAGGAGCATCGTTTTTAAACAATACATCTAAGTCACATTCTGTGAGTT
**CoV-bait-1050**	CTGTTGAGAATTCTTTATCATCTATCTTTTTTACGAGGGATGCTACGTTAAGCTTATCATCTTCATTGAGGGCGTGGAGGATGTTTTTCTGATTGGGGTGATAGATTTTAGGATTTTTTA
**CoV-bait-1051**	ATTGGACGAAGGTTTTATTATCGTCTTCTCCGTTACCGAAGTCTGTTGAGAATTTAACTGGGTGTACAGTACTGTTCTGTGTTGTGGTTAGCAGAATACAGACATCTCCTCTAACAGATT
**CoV-bait-1052**	AGAGTGTATAAAACAACACTTAGGGCAAATGCAAGTTTGCTCGAAGTTTTTAATAAAATAAGGCCGTGAGTGTGAAACTCCCACGACTGTATGTGATGTACATGTACTAACACGAATGAA
**CoV-bait-1053**	TAACGGCAGTCTGCTCATCAAAAGCGACTTTCTTACCTGCGCAAGGAAAACGCCACGCTTGATCCAAAACGCCAATTGTTTGATCATTCATAACCACAGGGAAAAATTGATCACCACACT
**CoV-bait-1054**	TACCAAGATTAACATAACCAAACTGTAGATTAACACCCATATCTAAGCCTCTAAGATAATAGTACCATTGGGATGCAAAATTGTAGAAAACAAAGCAGAGTGTAACAACAAACACGGGTA
**CoV-bait-1055**	GTGCGCGCATTTTAAGATTTAAAAATGAGAAGACACCAAAATACATAGTACAGATAAAACCTATACAAATATACATTAATAAAACTATCTTAACTTCAGGTACAACCACACTTAAAGTTG
**CoV-bait-1056**	ACCGAAGGCTTGAGTGGGATTATATGCCTTGGTGGCGACACGCTTATGGCGCATCTTTTTCTTAGCATCTGCGGCATCTTTTTTAGTGATAACCTTAGGTGCTGATTTTGATTTACCTGC
**CoV-bait-1057**	TTTCATTGTTGAGCGCTGCTCTATACGTGGCCCTCGCACCTTGAGAACCCTCACCAGTCGCGGAAGACATGATGAGCCCCGGATTGTCACGCACCTGACGGAACAGTATATTGTGACCGA
**CoV-bait-1058**	CACACATAGGGCGTTATGGCCAATATGAATTTATGAGGTGTGGCAATCACGTGGTCATAAGCACACTTTGTACACAACATGGGACGTCGTAAACAGTCACCACAACGAAGTACAGTTTGA
**CoV-bait-1059**	ATTCTGTACAAGAGTAGACAGCGCGATGACCAGTAATTCCTCGGAAAGACCAGTTTTAAACATCGGGTTGCCTTATAACTCAGTTTTAATCTCTTTTGTTTTGGCACAGCACCAACTAAT
**CoV-bait-1060**	ACACTTCATCTCTGTAAGTTTAGATTGCACAGTAGAAATTTTTATATTGCGTTCTCCACCAATGCCCATCAACTTGACACTAAGAATCATAGCATCATATGCAGTCTTAGGTGCAGACAA
**CoV-bait-1061**	TGCTTTGCCTGTTGCCCTACACGGGCCGGATGACGCTGAATTATCTCTTAGCCTAAAGTACAAGAATAACTCTCTTTTCGCGTCTAACGTTACCGCCGTAGACCACTCCTTCAGAAGAGT
**CoV-bait-1062**	ACGACAGACGTCGGCATAACCCGACATGTCAACAGCCTCCTGAACTGCAAGATAAAACTCACCGAATCTAGGTTCACCAATATGCTCAGCACACAAGCCAGCAAAGTCCACTTCATCAGA
**CoV-bait-1063**	ATTCAGATGTTTTAAGATAAGGCACTGTAACGGTCTTATTTAAGGCCTTACCACGACCAACACGTAATACAAAACCTGGCACACACGAATTAGTTACTATCTTATTCAAACCATTACATT
**CoV-bait-1064**	GTAATAAATGCACAAAACAACTTATCTTTAAGAGCAAAGTGGGCTGTAAATGGCACATCAACATCAATAAGTACCATAGACTCACCACACTTGCACACATGTGAGACAACATCACCATAA
**CoV-bait-1065**	TCTAGGTGGTAACAAGACTCTCAAACCATCAAAATAAAAACCACCCAGCTTCTCACCACCTAAAACAGTAGTATTAAACTTTTCAACAGTTGCGAGTTCCAGACTACGATCTGAAACTGT
**CoV-bait-1066**	TTAAACCATTGATCCAACTCTTCCTTAAAATCAGGGAGGTTGGGTGTTGAAATGTTCAGCATTACATCCGGCGCTTTAGTATAATTAACAGCACAGGTACTCATAACAACAACATTATTT
**CoV-bait-1067**	TCTTAACCTCTGGAAACACCAACATCGTCTGAGGCGAGTAGGAGTAGATGAGGTAAGTTAAAAATTTTGCAAAATTCACTGCTGTGAAAACAGTGACTGTATAATCACTGGTCAGTGTGG

**CoV-bait-1068**	CGAGTTGCTCGTACTTAGTATCTGGCGAAACATAACGAGCATTCGCCATCGTAATAGGCTCAGGGCTAAAGAAAGAACTACCAGTGTAATACCATTCCTGGTCAGCAGCACGAGTAGTTG
**CoV-bait-1069**	AAAGACATCCTTAAAGTTTAGATATTATTTCTCAACAATGCGGTGTCCAAGCCAGAACCCTTTTGGGTTGATGGCAGTCGGTAATTACCGACTTTGGACTTAACATAAACAGCAAAACCA
**CoV-bait-1070**	AACATGCATGAAATAAAGGCCATTAGGGGCATTAATAACAAAAGACACTATATGAGTGCCTTGACCACAAAAGCCAGACCTAGTGGACTGGGATTTAACGCACTCGTTGACTTTATCCTT
**CoV-bait-1071**	GAATGATAAAGTGTGTTATGGGAATATCTAGAGGAAACTCAAAAGCTGGTTTTGGATAAGGCACATGGTCTGTTCCATTGTGGAAACCATGGAAAACAGAAAATATGCCATTTTGATATT
**CoV-bait-1072**	CATAACATAAGTAACAACCGTAGGGTTGATTTCCAATCCACACGGCGCAACTTATCCATACTTAAATCAACAGCAGCAGTGCAACACTGTTTTACAACATTACCTGTCACAACGCCAGCT
**CoV-bait-1073**	CCATTAGAATTACGCAAGACAATTTGGTTAACTGAAGCCGCATTACAATCAATGAGACTACCTAAATCACTGGTAGACACACTATCAGGTTTAACATATGAGGGTACATAATTATTGTAA
**CoV-bait-1074**	TTACCACATGGACAGTAGGCTTCCTCAAAATTTGAGTGACACCACATTTCCATGCCACAATCCTCACAGTAATGGAGTGCACAGAGCGCCATTGGCTTGACTTCATCAAAGCCATTGATA
**CoV-bait-1075**	AACATAGGCGCTAGGATGTTCATCAGAAGATCCACAACATCATTACCGCAATAGACTCCGCTTTGCGATGTGTAATATGGGTTGTTCAAAACCCATGAGCCACGAAGAGACAGGCATAGT
**CoV-bait-1076**	TAGTTTGTGGTTGAAGGTCAATGGCAGCACGAGGAAGCGGGTCTCACCCAGGGGCTCCCAGAACTGAAGCAATCGAACAGTCCAGGCTCCTGGCCGCAGTGGCAGGCTCAGTGGGGGCTT
**CoV-bait-1077**	AATATTGTTTAGAAGTGAGAGGTGTAACCCAATATTCTAAAGTCATAGCACTATTACAAGTCAAAGGCAGGACATAATAATGTGAAAGCACCGTGCCTAAATAAACATTAAACAGAAACT
**CoV-bait-1078**	TTCATATAAAGCCTGATCAAGTATAAGAATTGGTTTGCAAAGACATTGCGCATAATAGATGGCTGCATTTTTAGCTTCCTCTAATGCATGCGCACTGTGTGTATTACAAACTATAAAACT
**CoV-bait-1079**	GTCCAGAGTGCTTCCAGTCCATAGCTAGGATTATTGCATCTATTTAATTATGCAAATCTCGTGTCTAAGGGTGGGCTAGTGACCACCGCCGTCACAGTGTGGTAAGGATGATGATGCATA
**CoV-bait-1080**	ATTAACATTGCCCTGAACGCTATCGCACTCACCATCATCATAATAGAAGAATGGTGCTTGTTTTAGCTTGGCAAAAGCACGCTTGTCAAAAGCAGGCGTGTGGAAAGCGTGATTGTTCAC
**CoV-bait-1081**	AAACCCTTACGTAACTCCGGTACCATGATCCTCTTTCAGTACGACACTCTATCGGTAGCCGACGGTGTCCCTCAACTGCGGAACCCGATGTCGAGAATGTAGCCATACGCAGACTTTGCA
**CoV-bait-1082**	GGTGAAGTAGGACAATTGCATAACTCTATTCCATAGTGGGTCCGACTTATAATCCCATGACACTTGCAGATGATCAAAATCTGCCAATTCATCATAAGATGCCAAACACGCCTTACACAG
**CoV-bait-1083**	AGTTGCCAGCAGGCACTGTTTTGTTACTCTTTTTAAATACATTTATGGCTTCAAGCAATGCACCGCCGGTCACATTGTTAAGACACTTACCAACGCTACAAAAATCCAAATTGGAATTTG
**CoV-bait-1084**	TGAGCCACTTTAAGTCGACTAATGTGCTATTTATGTTGTCAATCAGAGTTTGCAATTTTTGAACAGTGTAATTAAGCTCCGCAGATTTATTTTCAAGGGTGCTAATTTCACTGGTCAAAT
**CoV-bait-1085**	GTTAAGGACAATGCAAAAAGTTGTTCACCAGTATCAGCATTAACCAACCAGTGAGTTGGTTTGAGGTCAACTACCCACTGATTATCTTTCAGCTGAGTGTTGTGCATAAACCATTCAGGA
**CoV-bait-1086**	TAATACCTAGAAAAACGTTACCAACAGACACAGAAAAATTGTGCAGTCTCATCATAGTGTACTCATGCTCATAATTAATGGTACTATTAGTGGACGAGGCTATAACATGTCTAGGACATA
**CoV-bait-1087**	GTACTGCGTGGTGTAATGTGTAGTTTCAACACACATCATATTGGTACTGTCATTATAGCCGGCCAGTGTAACATTAGTAGATTGCGGGTAACAATTCCAACAAGAACCAGAACCTGATTG
**CoV-bait-1088**	TCGCGGAAAGGAGAGACCTTCTTTACCATGCTGAGTGAGAGCTGTGAACCAGGATGCTGTGTTGTTAGGAGGGCCTTGCGGTCTTCTTTGCTTTGGCCGCGCTCCAGCACGTTCTGCATT
**CoV-bait-1089**	GTCTAACGACAAAATCCACACCATTAGAGAATCCCAACTGCAATGGCACATTAGTACCAACATTAGAACCAGTTACATGAGCACCCTCAACATCAAAACCTAGCCAGCCTCTAACATTGC

**CoV-bait-1090**	GAGATAGTCACCAGTCTGTTGTTCAATTCTAAATGCACTATATGCAATGACTGGCACTTGCTTCACAACAACAGCAAAAATGTCACCACCAGGTCCATCATTCGTGCGGTAAAACTTGGT
**CoV-bait-1091**	TTGAGGGCATATGATAGTATGTGCAGATAGTATTGTGAGAATACGAGGGCGATGAGCAGTGTGGTGTATCTGTAGACTACGAATGTTGTGATTGTGTTTCTGAGCATTTCTGTGTTGTAG
**CoV-bait-1092**	AATTTAATTAATGTTATACATATAAGCAATACTACAAGCAACACTATCAGCCAAAGTAAAACATTAACTATGATAGCATGATCATCCACTAATTTCAGAAACATCTTCAAATTCGAGCCT
**CoV-bait-1093**	CCGCTATAGCAACTACGAATCATAAAAGTTCTATTTGTTAAGTAGTCTCTAAAACCATAGAGATTACCATTAGAATCATATAAAAGGTTCTGCCAACTATTATAATAAGTCGCATTAACC
**CoV-bait-1094**	GAGCTTTCAGCGTCGCTAACAACATCACAATCACTATCATCAAAAAAGAAGAATGGCAGTGCTTGGAGCTTGGCCAATGCTCTTCTATCAAAAGCAGGTGTGTGAAAAGCGTGCTTATTA
**CoV-bait-1095**	GTCTGCCAATCAACGTTGGGTGCTACCACAAGTACTTGACCTTCATCTTGCACAGAGGGTAAAGTGCCCGACAAAACATCTTTGCCGTCCGAAACAACACCAATCTGAGACTGGTAAGAT
**CoV-bait-1096**	AGCTAAACTACCCAGTACCATACCTCTATTTAGGTTGTTAAGGCCCTTGATAAAGTACAAGTACTTCACTTTAGGGCCTTTTGGTGTGTCTGTAACAAACCTACATGGTGGTTCCAGTTC
**CoV-bait-1097**	CGATAGGACTGTCCCGCGGGTACTCGCCCCCGAGCGCAAACGCCAATAGACACGACTTAGCTCTAGAGTCTCTGTACCGGGCGTCCAACTTTTTGTGGGCATACGCTAGCCCGTTAACAT
**CoV-bait-1098**	TGGTCCATCAGTTACGCCGAACGGAATTTCACCGTAACTGAAAAAGCTCGAGTCACTCACTGTATAACATGAAATTTCAAGAGTGACACCACCCGTTGTGTTCAATGAAAACACTGTGGC
**CoV-bait-1099**	GTAGGAGTTAGAATCAAAAGGGTAAGTTCCCAAACCAAAATAACTACGCAAACCACTATTATAATAGCCAACAAAAACACCATGTACATTAGTAAACTGACCAGTAAAAGCACCACATGT
**CoV-bait-1100**	TGCCAAATCCATGTGGCTGTTGGTCTCATATGCCTGGACAATGCCTTAAAAGTATATGGACGACCAGAATTAAGATGAGGTTTAGATTAGTAACATAATCTAGGGTCTTAGGCCCAAATG
**CoV-bait-1101**	TCTTGCACAGACTCAAGCACAACAGTGTCAAACTGGACACGTGAGCAAACAGTCTCTATAGCACCACCATCAAAGGTATACGCCTCACTAGAACCATCTTCAACAGGACGGTTAAAGGCA
**CoV-bait-1102**	TTGGCACAGCACCAACTAATCTGAGATTAATTCTTTGATTCCCCTCACCTATAACATAAAAATTATCTACAAAGGAATAGAAAGGGTTAGACAGATCAAAATGGAAATCAATATGATCAT
**CoV-bait-1103**	ACATATGTGGCTGCAACGCTCTGGAGGACAGAACTATCGTTGAAATAAGAATCCAACAACTCATCCACACCAAAAGCGCTGTTCTTACTTAAGAAGAAAGCCAACAATGCCAACAACATC
**CoV-bait-1104**	GGAATTTCCTCTGCTGTTGGACTGTCCACCACGACTACGACTCCTTGAGTTAGCTCTGGAGTTGTTTGGTGTGGTAGGTTCAACGATTTCCACATTATTGGGTAATTTGTTCTTAAATTT
**CoV-bait-1105**	ACAACTTTGAGTGGTTCAACATTTCTGACAAAAACAAAAATAATAAACAACAGACTATTGATGCATTTCTAAAAGCTCTGGAAACCTTACCACTCTGAAGTGTTACGCCATACATTTGTT
**CoV-bait-1106**	GTTACAACTTCTTTTGCATGCACAGGTTTGCCATCGTTATCTTTAATGTCAACGACAGACCAGATAACACCGGCATAATGCACACAACCTTCACGTTGTATTTTAGAATATGATTCAATA
**CoV-bait-1107**	CATAGAAATGCTTCTGAAGATGAAGCATTTACAGCAGTACAAAATAACTGATATTCATCAAAATAACCTGCTAACTCATAGAGAGCCGCAGAATATGAATGCTCAGTTACCTTTACAAAT
**CoV-bait-1108**	GATGGAGTAATTGGAATGAGTTTTGGGAGTAATGTTGATTAAGTTAGATGCAAAGAGATTCTCAAAAGAATGAGAATAAAAGAGGAAATAGGGGTAAGGGTTCCGGTCAAGGGGTTTCCG
**CoV-bait-1109**	GATGTCTGTGTATAAACAACATAGTCATACTCACTACCTTGTGATGAATCCACTGTCTGAATTTGCAGACCTAAGACTCTGGCAGCGACATAATTCTGACTATTGTATGGTGAAATAAAA
**CoV-bait-1110**	CAGGTGTTTCTGGTGGTATTGTCTAGTAGTCATGGTAGACAAGAGTGAAACACCACCAACAGTGCGTGCTCGATCTTTACCACTAATGGCATACTTCAAATTAAGCTGGGTCATAGTAGG
**CoV-bait-1111**	CTGGTGTATCATAACCCTTTGGTTTAGTAGAAGAATAGAAGCTCAAAAGTTCCATATCTTCTTTTTGACTAACACTATTTACAACAGACAATATATTATCACAAACCGGACCATATTGTT
**CoV-bait-1112**	TGATATCGCTGCTAATACTGTAATCTGGGACTACAAAAGAGAAGCCCCAGCACATGTATCTACAATAGGTGTCTGCACAATGACTGACATTGCCAAGAAACCTACTGAGAGTGCTTGTTC

**CoV-bait-1113**	GGCACAAAGTCAACCTCTGAAAGTTCCAGGGGTTCAACATCAGCAACCTTTGGGTTGTCAATAGCATAAGTGGCATCAATGAATGCCCAAAACTCGCCAACACGGCTGTCAAATGAGCAA
**CoV-bait-1114**	GTTGTTGACCAGCAGGGAAGTAACCTGTTGAGGACAACCCATATCTCCAATAGTTTGATCGATTGAAGCATAAATCCATGCAACAAACCGACAGGGATTGCCATTCAAATACTCCTGGTA
**CoV-bait-1115**	TTATCAACCAATATGGCATCATCTGGCAACCATCTTTTTAATACAGTGGTACCAGGAGCAACACCATTAGCGCCTGCTGCACCTAAATGCAAAACACGCATTTTATGTGGAACACACAGT
**CoV-bait-1116**	ATCCTTCCTGCAGATATTACCATAATGGGCACGACCTTCTTTCCACTGAAAGAAATCATGTTGTGCTACAGCTCCACACTTAGAAACCAGCTCATAAATGGATTGCTCGTGTTCCATAAC
**CoV-bait-1117**	CTTTAGCCAGAATATACTCGTATCTGTAACAAAACCAAAAACAACGGTTCCGGTGATGTGAGGGCCGCAGGGCTCTGCTATGAGTCTCGGAAGCTTAGAATTCCGAAAGACCCCGGTTAT
**CoV-bait-1118**	ACCCTTTATAACCACACTCCATTTAAATGTCTGTAAAGCGTTTTTAACAGCCAAACAGAACAATTTAAAAGTTAGCTTTGAAGCTATCTCTGTGGTATACACAACTTTATTATTGGCCTT
**CoV-bait-1119**	CTTGGCTTAGTCGCTGCCGTGCATGGGCTAACTATATCCGGTTGAGCACACGGGCAGTAGTTAGGCCGTACAGTAAAACATTGCTGGGCATAGGCAACATCATGCTGCTTCTTGCCAAAT
**CoV-bait-1120**	GTATTTTCACCGTCAATTGATTTTGTGGAGCCATCGTATAAATCAGAGACGAGCAAATCAAATTTGTCTTCAATGTAAAGACTAGTACAATCACCTGTAACACTGAAGTCTGCGTCGGAA
**CoV-bait-1121**	CATTGGAGACCAAAAGAGAAGTCACTTGTTGAGGACAACCCATCTCTCCAATATTTTGATTAATAGATGCATAGATCCATGCAACTAATCTGTTAGGGTTACCATTAACATACTCCTGAT
**CoV-bait-1122**	CTCAACTGTAAAAGTAGCTAACTTTGAGCTACTAAGTAATGCATCAAGAACTGGATGAATATCATATGTAATCGATACACTACGATAAGCAGTTACTTCAACGGTATTAACATCACCGAA
**CoV-bait-1123**	AGCCACAACTAGTGTAGACATATAGCTTATGCATGAGCACTTTAAACAATTTGAACATGTCATTAATAAAGCCTGCAGTAGCACGCGACGCTGCTACACAGGCATTAAGAAAACTACTCA
**CoV-bait-1124**	TAACTCGCATTATGACACAAGTCACGCAACTCTTCAAAAGTAGATGTCGTACCAGACAGTATTGCTAAAACAACCTCTGTAAGAGCAAACTGCTGACAGAAATTAAGAGAACGCTGGGCT
**CoV-bait-1125**	CAAGGGTTGTTTTGGAGATGTCACCATAGACCACATGCTCAAAAGCATAATCCTCAAGGCCATATTTAGATATAAAGAGTCCAGTGTCCATATTAAGGAAATCTTCCTCCATGGTACTAC
**CoV-bait-1126**	CTTATAACAGCGTTTAAGTAAAGCAGCATCTTCATTATTACGAGCATCAGGACCAACAACATGCAAAATTGCATCTGCTAAGCCATGGCCCTTAAGTAATACGGAATCACCTACATGAAG
**CoV-bait-1127**	GTTACCAAAGCATTCACCAGCCTCAGGAGCATAGATGAGAGCTAGGGTAACATGATAATCAACAGTCATAGACACATTAAGACGCGCTAAAGAATAGACGAGTCCATCAAAATGTATCCA
**CoV-bait-1128**	TCAATAACAAGTGGCCATGTCAAACTTTCATTAGTTTCTACAACATCTGTAGGTTTGACAACTTCATTATCCACATTGTTTATAACTGTAATATCCCACAAAGACCCAGCATAGGAAACA
**CoV-bait-1129**	TGCAAGCATCTTAACATAATTGGACAATGGTGTTCCACCAGAACCAACATAGTCCAAGTATGCTTTAGCTGGATCAACTGCAAATGCCACAAGAGTAAGAACAGTTGAATTACTAGCAAA
**CoV-bait-1130**	ATGTATGGACATTCCTATATAACCTAGGTAATAACCATTAACATAAATGTTGCCTGATCTAAAAATCGTAACATCCGCAATATTAGGTGGAAATGGACCTACATAAAAAGAAGATAATGT
**CoV-bait-1131**	CACCTATGATACCACCAGCCTTTTTCTCTACTAACAATTTCTGGGTGTGGCATGAAATAACTTGTTTAGCACCAGACTTTGTTATAAAGAAACGACATCCTGACTTGACAGTAGCTGAAA
**CoV-bait-1132**	CATACAAATGTCCAAAACAGTCGTACGGTTATAACGGTAGAAGTCAAAATCCTTAATAGAAGCATCATCCTTCTGTGCAAAAAAGAAATGCTTAAGTGATAAGTTAGAACCTTCCTGGAA
**CoV-bait-1133**	CTCAGAACCTTGTGACGAATCCACAGTCTGGGTCTGCAAACCCAACATACGGCGTGCAACTGAATTCTGACTATTATAAGGTGAAATAAAAACAGCAGTCTGCCACTTGGGGTTTGCAA
**CoV-bait-1134**	GTAAAATTAAGAGACTCAAATAACTGCATACTACTCATGACACAGAGGATACCCTTCTTAGCACGTGTAATAGCAACATTGAATCTGTTTACATTTACAGAATGCGCTGTTTCCGCAGTC

**CoV-bait-1135**	CAGAAAAATTACAGTGGGCTGTGCAAAATTGGTGAGTAGACCATGACATACCCGGTTCTGGTGCTGTCATAGCTACAGATGAAGCATTAACAACACGATCGTCCTTAATAGCACCAGCAG
**CoV-bait-1136**	TCACCAAAATCATAGAACTTACCATTAAGGTCTTGATTGTCAAGCGTCAAAACACCCACTATGCCAGCTTTGACCATAGCATCACAAAGCTTCACACAATTAAGCATAGCACGTGCCACT
**CoV-bait-1137**	AGGTTGACATCTTTAATTTCATAGAAATTAAGACCATCATACATGGCTTTGTCACACATTACACACAGTATACCAATTTTAGCACGCGTTATTGCAACATTAAACCTATTAATGTTAACA
**CoV-bait-1138**	CACCATTATCCATCATAAAATAAACCATAAGAAAAATTATAACCAAAACAGATATACCCAACAAAATGCCAAGCAATATTATAGGTAGAGGATCATAAACACACACAGGTGCTATATAAC
**CoV-bait-1139**	CCCAACAATTTAAAATTAGTCCAAATTACCTCAATTGCATTAGTAGGAGGTTTCATACGAGTAGCCATCATATATTTAAGCTCATCGCGAGAAACCATAAACTTATAAGTACCAACTGGT
**CoV-bait-1140**	GCATTAATCCAGCAGTTATTGTCAGTGGTTTTAAGAACTCTGAAACCATTGACAAAGTCATTAGGATAATTGTATGGCTTGGTGTCTAGTGATGCAAAAAGTGCAGCATTATCAAAACCA
**CoV-bait-1141**	TACTAAGATCCAACTTTCCCGTGATAATACAATCATGGACTGCATCATCAACTTCACCAGTAATAAAAGCTCTCTTAATAGAGCATGCGACAGGTGAAGTACTTTCTGGTTTGATGAAGT
**CoV-bait-1142**	TTTACAAACACCTATGTTACTATACACCAACACAGGCTCTGTACAATTAGAGCCATCATTAGAATGGTAGAAGAAACCAGGCAACTCCCTAGTACTGTTAAAAGTGGAGCTAGACAAACT
**CoV-bait-1143**	GAGATCAAGAGGTGACACTTCCACTGGTCAGTGGGTAGCTTTAGTTCTGATGATGTAATGACATCACGTAAATCATAGCCTGATATTATAGCTTGATTGAAGTATTGTTTGAGAACAATA
**CoV-bait-1144**	GCTCCCCGCATTCTGGTGGTCTGACTAGCAGGCGGCCAGATGCAACAAGATACTTAAGTATCGGTTTCTTCTCCAACTCCTTGGAGGAGATTATGGGCGTGGCTTTAAGCTTACAGGCAA
**CoV-bait-1145**	ATGCATCACCTGCTGCTATGGCTTCTTGGTAAGACTTTTGAGCAGTTTCCAACTCTGCATAACTCGCCAGATGTGAAAATTCTGTTAAAGTGGATTGCAAAACACTAGGATTGTCTAACA
**CoV-bait-1146**	ACCTCCAATCTGAGGCCTTAGTTAAGGTACCAGAATCATACTTATATAACAATCCTTTTTTTACACGAGCATGCACAAAATGTCCTACACCTGTCGCATGCCCTATAAAGACGTTAAAAG
**CoV-bait-1147**	TAATGCCCTGCAAAAGACTGCGTAACTCATCCAATGTAGATGTGGTGCCAGTTAAAATAGCTAACACCACATCACTGACAACAAATTCATTAACGAAGTTCAACTGCTGCTGTGCTTTCT
**CoV-bait-1148**	TCCAATCTATGCTTAAAACATAGTGGCTTAAATTGGTTTGAACTATTTGAATTGCGCTATAATGAGTGAGCGAGTCTTGTCCCATTAGACACCAATGACACACATACCCAAACTTTTCAC
**CoV-bait-1149**	TGCAGAGGTGATGAGTACGCACCAGCGCGTCTATTACACATACAACACTTCTGCTCTTTACCAATCTTGCAAAAATAAGATGCAGATGTTAATTCAAAGCCATGAGCCCAACAAACAAAC
**CoV-bait-1150**	TAGAAAACTTTCCTTTAGGATCCTCCTGTAAGTCTTCAACAATCTGAGAAAAACCTTCAATACCATGAAAATCATGGTTCTCAAACCGAGGAACTTCGCGGAACAATTGTCCACCAAGTC
**CoV-bait-1151**	AAACCCATATAAGAAATAACATGTTCATAGCGACAAGCACCAGTAACACCAATCTGCACAGCCAAGTCGCCATTAGTCTTAAAACTATCTGACAAGGCAACATAAGTAGCCGCATGCGAT
**CoV-bait-1152**	CTGTTGGCGTGCATTTTCATAAATGACATAAGAAGGCAAACCGACATAAGTAGATGCAACACTCTGCAACATACTATTGTCATTAAAATAGGATTCCAATAAGTCATCTAAACCAAAAGC
**CoV-bait-1153**	GGGTCATACCAAGTAAGATTATGGTCAAACCAATCCCATGGAGTTTGGTTTTCCCCACTACGATTCCAATACCAGTCTTCAATACAATCATTGAGGACAAGTATTTCTTTAAGAACCTCG
**CoV-bait-1154**	TGAGCAAAGAAGAAGTGTTTAAGCGTAAGTTCAGAACCTTCCTCAAAGAAACCTTGACCTCTTAAGAAGTCATAGAACTCTTTGTTAAAATGACCAGGTTCAACTGTTTGCTTAGTCATA
**CoV-bait-1155**	TGTCCAGGACACTACGAGGTGCACTAAGACCATTAGCTACCATGTACTTAAACTCAGCAGAACTAACTTTAAAGTCATAAACACCAAGTGTCAACTTACTAACACGGTTCAACCAATAAC
**CoV-bait-1156**	TACCACGATTAGTGGAAAGTATAGTTTTAAACAAATCAAAATTAACATCTGTACAACCACAAACTAACCTAATACAGGCTAGCAACGTTAATACAGTAAGCAATTTCATTTAGTTGTAAC
**CoV-bait-1157**	TTCTTGTAAAGAAGAACTAATTGCACCAAATTTATTAAATAATTGCTGTAACAAACTATTAAGTGCTTGAGCATTAGAATTAACAACACTTTGTATTTTAGCAAGTGCAGAGTTGGTAGC
**CoV-bait-1158**	TTTGTACCGATATTAACAGCTAGACCATCGCTTGTTTTAAAATTGTCAGACAAGCTCATATATGTTGTTGCATATGCAGGTGGTATGTATTGCTCATTCTTTGAACAATCTTTAAACAAA

**CoV-bait-1159**	GCATCTTGAGAGCTGGGTAATCAAGACCATCCATACCAGACATGCACATGTGGTGGAACCAGCCTTCGCTAGTCTCATTAACTGTTAGGTAATCACATGGGAACACAAAACCGAAGTATT
**CoV-bait-1160**	GTCCAGTTGTAATCCCAAGGAGCATCACGGAAGCGGTTGCCACCGAAGCCCTTCTTACGAAGCAGAAATTGAAACTCTCCAGTAACAAGATCCGCATCAAATGGGAAGAACATTCCAACA
**CoV-bait-1161**	CTGTACACGTTTTCAAGGTCCAGACTATGTGGGCCGGCTCTTTGCCCGGACTGAGATCACGGATAACATGACCTTCTCCGACGGGATGACGCCCATTAATGCTCCGCCAGTTAGGAACCA
**CoV-bait-1162**	GTTTGTGCTGTTCTAGAGCAGTTGTACCACACCTCTGTAGGGTAATAACCACCAACAACTACACTTCCTTCTTCTTTAAAGTTACTAAACAGAAAATCTCTGATAAGGTTTTCATTGCCA
**CoV-bait-1163**	AGCCTCTACAGCCCCACTTGGATGAGACATCTTAACCAAGCCACTCTGCAACACACCGGAGGTAATGCTACAGTTAGGTGGCTGATAAAGAAGGTCGCTACCAGTTTCACTATAGGTCTG
**CoV-bait-1164**	ATCATGAACAGGCTTAAACTTGTTTTCATAAACAAGCTCTGAAACTGTGTTCACGATTTCCGCAGGACAACGATAACATTTATGCAAGAACACATCAGGACCTACTGCACACATACGCTG
**CoV-bait-1165**	ATTTTCAATTGTTTGACAAGCTGAGGTGTATTGTGTTAACAAGTTAAGGCAATGTCTATTTCCATTGCAAACATACTTAGCACAATCCACAACAACAGGTTTAACTTGAATCTGGATGTA
**CoV-bait-1166**	GAACTTTCTTCTTCCTTCTTCTTATGCACTTTTTTGGCACGCTGTTGTTTTGGCGTTGCCGGTGCAGAATTTCTATCTTTAGATGCAGCGCGTGATCTTGGTGTCGGATCATCCTTAGGA
**CoV-bait-1167**	TTGAGATATTTCTTAGACTCTTGCCAATCACAATTACATTGGGAATACCCTAATGCAGTCGTCATAGCCTGAGTTGAAGCCTCCAAAGGTGTTCTAACTATTTGGGCCATTGATATCGAA
**CoV-bait-1168**	AGTGAGGATCTGAGGAATTACTGCAAACAAAAGAAAAATTTGTTCCTAAAGCAGTATGAAGTACAATTGAGCCTTCAGCAAGAATGACAGAGGTGTCATTAATATTAAACCTCAGAGCCT
**CoV-bait-1169**	TAAAAAACAAACAAGAAGCATTAGAACACCCACTGCGCATAGCAGAAATGCGAATATAGCCTGAAGGAATGAAACCTGTTAAAGGACACTCACCTGCCCCACTCTTATAACAATGTGTAA
**CoV-bait-1170**	GGCACCGTGGGTAGACCATGCCGAGTTTCCAGGTTAGGTGCCGACCGGAGAAGCTCACTCTGTGAGGCACAACCCGTATTGCGGCTCACCGGGACTAGTGACTTCTACGGTACACACCGG
**CoV-bait-1171**	AAAAGTCATCATCTCCAAAATTATGGTAGTTTGTACACTCACCAACATTAACATCACTATCAGCTACAAACTTGTTATAAGCAGCTGATATTATAACAGGCTTATAATAAATGGGTGGAT
**CoV-bait-1172**	CACTAAAGGTATCCAATGGTACATCATCAAAACCCAATGTGCTCTTGCAATCCTGCATGTCATTACAACTTGACAGATCTTTACCAAAACTATTCGACAACACACTAACAAAAGCACTAT
**CoV-bait-1173**	CATATTAAAATTACAATTACTAAATGTTTTGCGCTCCCATGAGATTGGAGAGGGTACTTGTGGATCATTTAACCACTGTTCGATCTCACAATCAGGGAGATTGGGTATACGCCTGTACAC
**CoV-bait-1174**	GTTGAGTAGATTAATTAATATGGACCTTCTCTATAGGCTCATAATAAGAGGGCTTTTTACAACTGTTTTTACAACAACCGGCCAAGCAGCTGGTTAAACAACCGCAACAGCCACAACAAC
**CoV-bait-1175**	AAGACCTTTTTTGTACCACCCTGCCACTTGCAGGATCATACACAGAAACACCCAGCACATTAGTAAAATTATATCCATCACCATTAAACGAACTGATGGTAGCTAACTGTAGAGCCTCTT
**CoV-bait-1176**	ATCATCTGATAATAAGAAACCAGATTAGAATACTGGTCACTAATATTAAAAGAAGGGTGCAATTTGTGTATAGTAGAATAACGCTCTTGGTTAATAATAGTAGGAGCAGTTAGTGGCTGC
**CoV-bait-1177**	GGCTGTAGACATAAAAACATTATTCTGGTAATACAAAGTAGCTTTAAAAGCACTAATGTCTGCTACATAACCAAGATCAGCATAGTCTTTGTTGTAACAAACCACGCCATCATCTGACAA
**CoV-bait-1178**	AATCCAATATCCATGGTTCTGATTCTTGGACACATTTTCATTAACAGGTACACCTGGTCCCTTAAAGGTAATTCCTTCTTCACCAGCCTTCCTTCCCTTTATTGCCTGAAACCAAGAAAC
**CoV-bait-1179**	GGCCATTAACACCAAATTTATTAAAGGTGTCCATACGACCCTCACAGTATGCACTACTGGCAGCATCATTAATGCCATTATCAGCACAACTATAAGTGTAATTTTGCAAATAAAAATAAC
**CoV-bait-1180**	AAGGGTTTATTTTGGGGATCCTTTTCGTCCGAAGACTTACGGTCGTCCCCACCCAAACAGTGTCCAGGTAGAAGGCCCAAGGCTGTCACACCGAATGGCGGTGGGAGAGGGGTCGCCTGA

**CoV-bait-1181**	GTGGTGTCGTACAAGTACACAAAGTTGGACGACTGAGTAAATTTCACACAATACATGTCCGTAGCTGCAAGGGCAGGAATAGTACCAGGAATCGCAGTATACGTTGCAGTAAGACAGGAA
**CoV-bait-1182**	AATTAGTTACTACAACTTGATTAGCAGGGATACAACCACCATCATACTGCTCAAAGTATTTATCAGTAACTTCAAAGCAAAACAAAAACTGCTGTATATCAACCATAGTAGGCTTATTAA
**CoV-bait-1183**	TTCCAAAACAAACATAATCTAAACATGATGTAAAATTAGCTAGTGATGATGGCAATTCTAGCTTAGGAGGTGCATAACGTATTATAACAGAAATATAAAATGAAACATCATATGCTTGAC
**CoV-bait-1184**	CCTCTTGAGAGGTTTCAGGCGCACTAGCACTTTCGTTTTCATCTTCTTCTTGAGCAGAGGTGTCTGCTGAGTCCTCATCAACCACCAGAGGATCCTCAGACCAACCCGCATCATCATTAC
**CoV-bait-1185**	CACATGTATAACATCATCATAATAACAATACTTAAATGCACCTGTAGTATTACAACTAGTAGACGCATTTACACCATCTATAGTAACCTGTTTAGGTAAATAATAACCAATGGCATTATT
**CoV-bait-1186**	CAAGGTGCTGATAGAGCTTAGTAAGCTCATTCCCTCCATACTTTTCTTCAAGTTCTGGAAAGGGTACATCACCTTCATTACAAACAATACCCACTACGGGGTTGTTGATAAATTTAAGGA
**CoV-bait-1187**	ATATTTATCTTTAGCATAAATCACAGTACCAAAACGCGCAACATTAGCATCACTGCCATCTGCTGCTGAAACTGTAATTTTTCTGTATCTAGCAATTTTCTCGGCACTACCTACTATACA
**CoV-bait-1188**	TTGTACTGAGACTCCACCATGACATCAACACCACAGCCTGCACCACTTTCACCAAACGAGAAACAAATCCTACCAAATGTAAGATAGTTGTTCAAAGTGTCTATGGTAAAAGGGCAACCA
**CoV-bait-1189**	CCCGCTAGGAATCGAAAATAGCTGGAATCCACGCTGGATAGTGCCGGATATAAGATATTGAGTTTGTGTGGAAGATAGTTAGTCACTCAAATACGCTGCTATGAGGGTGTTGGAGAAGGG
**CoV-bait-1190**	AAATCAGTGAAATTACAATAAGCCGTGCAAAGCTGCTTTTTAGACCAACTCATACCTGAAGGTGGTGCTGTCATAGCCACCGATGAAGCATTAAAATTACGACCACCACTAATAATACCC
**CoV-bait-1191**	CATATAAAGAATAACAACACACAAACAGCTACACCAGCTAATCCAATTAGCAACCAAATGTACCAAGGCCATTTCACATACATTTCATATGTGCCAACTTCCTTGAGGTTGATGTAGCTC
**CoV-bait-1192**	AGATGGTCTGACAGGTATTAAAGAACCATCAGCACAAACACCAAAATTAGAGTATGTCATAACAGGACTTGAACAACTGCCATTTCCATTACTGGCATAATAGAAATTAGGCAATTGAAC
**CoV-bait-1193**	GTATAGCCAAGCAGCGCGTCATAATGGCGTCGCAGCTTGCTACATGAGCACCCTTATGTACATCACAAATTGCATCGTGGTTGGTTTGCAGATTACCCACATAACCCCACTGTTGCACAT
**CoV-bait-1194**	AAATGTTGTATAATCATACTTATAAATATCCCAAACACGCTGTGGTTTAGACAAATCGCCATCTAAATGGCGTTCGGCTGCAAGCATGTCAGTCATGGTATAAACAGGCATAGCTAATGA
**CoV-bait-1195**	GCATGCCATGCGTTAGCAAGTCCTTTAGTGACGCACACATGGTACCAACAGAGATGCCAGTCTGGGCAACTAGGGGCTCCAGCGCAACGACACTATCTTCTGTTAAACTTTCATAGCCAT
**CoV-bait-1196**	CAAGAACAAAATAGTTTTGCATGACTTCACAATCTGAGTCTGTTACAGACTTAGCAGACAATTTATTGTCGCGCAAGAGCCTGTACATACCTATAACAGTGTGTAAACCACCCAATTGTG
**CoV-bait-1197**	CTAACAGCGGTTGATTTGTTACAAATCTGCCACTAACAAAAGTGGAGCTATTTGTAAGTAAAAACCAATTGTTAAAACTGAATCCGTCAGGTATGTAACCTCCCACAGTTGGGGCAAAGA
**CoV-bait-1198**	AAAGGATTGAAAATGCGAAAATTCGCCAATCGCTCTTAGTGTATAATTGTATGAAACTGAACCGTTAGCACCAACCATAATATAACCACTTTGAAGAGGTGGTGTTAACGTACAATTTTG
**CoV-bait-1199**	TTATATGATCCATCCAATACACCACACTTTTCTTCATCAACACCGAACCCTGCACAATGTTCACCAACACCAACCAGAGACTTTTTTTGAGAACAAGACCTAGGGTCATAAGCAGTTATA
**CoV-bait-1200**	AATGAAAGAGCCTTTAATTGTATGATTAGTTCTGAGGTTTACACTGTAAACACCAGCTGGAGTGCCATCATAACAGGCCAGAATATTAAACATCTCACCAGGTTTCACAGTTCTAAAACT
**CoV-bait-1201**	ACTATGATTTAATTGTGTACCACATTTTTCCTCATTAATACCAAGACCTGGACAATGTTCACCTATACCAACAACGACCTTCTTTTGAGGACATGTGTTAGGAGAATAAGTAGAAATGGG
**CoV-bait-1202**	AATCAGTTAAATTATCAGCATTAAAAACGCAACCAAGATAACTATCAAAATATGGCTGAGTAGCTAAAGAAATATTATTCAAAACATAGCTACATTTTAAATTACGATAAAGTAAAGCCA
**CoV-bait-1203**	ATCAGGTAATTTCACTTGTGCACCATAATTGTAGAGATTACACCGTTCGAGACACATACGCTGGATTTTGTACAGTGTAGGCATGCTATAACCGGGGTTCCATTCGGCAGATTGGAGTTG
**CoV-bait-1204**	TCTTTATCAACTTCAAACTCTGAACACGCCTTCGAGAGAACACTATCAAAGGTCGCATCCAGAGCGAAGATAATCTTAATCTTTCGCGTGGAGGGTACCTCCTTGACTTTAGGCTTGTCG

**CoV-bait-1205**	CCTTATAGCCCTCTACAAGCAATGTACCACTAAGGAGTGTTAGCGTTACACCAGTTGGTGCTCCAAGCACTGGAATGCAGACCTGTCGGCCCATCACAGAAGTAGTGAGAAGCGCGTCTG
**CoV-bait-1206**	TATATGTACAGACATTAACAGTGTTACGGTACAATGGCATATGGTTGTTGTAGTCCCAAATAACAAACTTATATGCAACATCCACACCAAGACCGTTTAAAATTCTGTTGTTAGGTAGCG
**CoV-bait-1207**	CTTGTTCAACAGAGTCCTCAACAGCGACAACTGACACGGTTTCTGAAACATCTTCAGCGTCAGCGGTTGAAGTGTTATCCTCTTCAATTTGTTCAACACCTTCATTGTCAGAAATCAAAG
**CoV-bait-1208**	TTGCTTAACTAAACTACTATTAGTAAAAGGATAAAAACCATCTGAGAAAATGGCACCTGAAGTATCTAAAATAGCCAATCCAGCATCTGCTAAATAACAGAACTATTAACTACTGCATAA
**CoV-bait-1209**	TGTCATCATAAACCATTCTGTTAGCTAGGAGCATCAAGATGAAAACGGCAACAGCAAGGTAAACGACATCTTTAACCGTGTAGCCGTAAAAAGCCCTGACAGTTTCAGCCATGTTGAAGT
**CoV-bait-1210**	GTTGAATAAAATTATCCTTAACTAGGATTTTCCACTTTTGTGAAAGCGTATGTAAATATATCACATACTTTTTCGCATCCAAACCATAATAATCAAGAACAGCCTTGTCAGTAGTTGGAA
**CoV-bait-1211**	GATTTTAAAGTACTATCAGAACTTGAAATGAAGTCTTCTACTTTCAATAGGCCTATGCGAGATAAGCGTATTTGAGAGATGAGTAAGTGCAATCCACCAAGTGTAGATTTAGCAACATCA
**CoV-bait-1212**	TCTGTCGCATAATCCCAGTACAATGCTTTAAGATTTTCCATTGTAACATAAAGATTATTATTATTATTGCGAATGCAATTAAACCAAGGTGTCACAGTAGGAAAGTAATCACCTAACACC
**CoV-bait-1213**	GAACCATAAATTGTAACAGCCAAACCAACTCTATCCCAAGTTTCCCACCTATTATTTTCATCATGCTCACGTACATCAAAATAAAAGCCATGTACATTAGTTATACCAAGACCTGCAGAA
**CoV-bait-1214**	TGAAAGCTCTTCTCGACTCTATCATATGTGCAATTAAATGCACTTTGATAGACCCACGAACTGAAATGGACGCCTCGAGAGACTGTAAACATGGGTTCTTTACAAAGGTTAAAATTACAC
**CoV-bait-1215**	AGACCAAGCCATACCTGATGACGGTGCCGTCATAGCTATAGAAGAAGCATTAACAACACGACCACCATGAATAATACCAACAGTACACCCAGATGAAGAGCCTGCATTATTAGATTCACT
**CoV-bait-1216**	GGTTCTAGTCGAGCTCCACTAGAACCCCGTACTCGTTTAAATAACTCTGATCTACGACAGCAGCTTGCATCGATGTTCTATCACAAACACAACCATTGTTAAGCCAACACGCACAAACAG
**CoV-bait-1217**	CATAACGTTCTAACATAATAACATTGTCAGTTTTGACAATGTCATCAACAAAAACACCCGCACTCAAAATTCTAGAAGGGTCTGGATACGGTAGATAATAATTACCTTCTGGAGTCGTTA
**CoV-bait-1218**	CCACCCGGGTAGTCTAGAAATGTCGTTGGGCTGGGCAGGGTCCGCCTGTCTATACATCGCCAACCTGAAAGTGTATCATCCTTGTTCTCCACTGAAGTGTCGAGCATTGGGCTTTGGATT
**CoV-bait-1219**	ACTGATCATCCAACTGTGCCGAACGATAAATATTGTTATAAAGTTTATATTGCAACTCCTTAACATAGGAATTAACAATCTTATGACCATTAGCAGACATAAGTGCCCCCACATTTGCAG
**CoV-bait-1220**	TTCTGACACCCAGGTTAGTGGGTTCAGTCTTTGCGCCTTCTTTAGCAACCCAGAAAACACCCTCAGTACGAGTCCTATAACGGAGGTCGGCGTGAGGTCCTGTTCCGAGGTAATAGAAAT
**CoV-bait-1221**	GTGCGGAGCCTCAATAGCATGACGAATAGGTGACCAGGTTATGCTTAGTTCAAGGTGGCTGACATAGGTCAGTCTGAAGGTCAAACATTGGCACTGTGCTTTCATCTTATAAGCGGCCTA
**CoV-bait-1222**	AGTGGCCTTGGCGACTGTGACGAAATTAGGTAATTGACTTACCTGTACGCCAGTAGCAACCTTATAGCCCTCTACAAGCAATGTACCACTAAGGAGTGTTAGCGTTACACCAGTTGGTGC
**CoV-bait-1223**	ACACCGTAGTAGTCGCTTATGGCCTGTACTTCTTCTGGAGAAAAGTTGTCTTGCTTAAACAATTGTATGTTGGCATCATCGGAGTTTGGAACTCTAGATGAAATGTCAACACCATTATGG
**CoV-bait-1224**	AGCCGCCGTCAAATTCCCATTTTACAACCTTTAAATCTGGTTTATCAGCCAAAAATGCATACATAAAGGTTTTACCACCCTCATTATTATAGAGCGCCTTGCCTTCCACAGAAAACCCGT
**CoV-bait-1225**	CTAAAGATAGTGACTAGCTTTTCTTAACCTTGCCATTCATCTCAAAGAACAATATAATATTTACACCACTTATTAATATACTAAACAAACTAAAACCTAAAGTAAGCCATGTAATCCAAG
**CoV-bait-1226**	TTCAACCGCTGTGGGCTTTAGAAGATGGACACCACCCTCAAAAACATTAAACTCAGCTGCACCCTCTTCACATGGAACATCTAAAGATTTTGCACTGCAACGCTTAACAGCATGCAAAAC
**CoV-bait-1227**	TCAGTGCTGAGGCCATAGTATTAAATCCATCTGAAATAGTTGTAATAGCACTAGAAACTTTTCCAAGTGCTAAAGTAATGTTACCAATGGCATTATTAAAAGCATTAGCAAGTATTTTCT

**CoV-bait-1228**	GTAAAATTATAATCACCACCAAATGCGAAGGACTGAATCTCATCACGGGTGTTAATAGAAAGCTCCTTAGACAAAGATAACAATGCACCATCTAAAATAGTGTTAACTTTAGCAAGGGCT
**CoV-bait-1229**	GCGTCTTTGTAAAAATAGTTCTTTTAGGACTGAGGCATCAGAACGGACGGCGCCTTGAATTTCTAGTACGGAACCTGCGATTGTCAGCCACGGATGTGGGCGCGCCTCACGGGATATGGT
**CoV-bait-1230**	GATGGCTTCCAGTATCGTAACGACGTAGCTACTACTAATGTTAATTTTGACCTGATCTTTCTATATGGTTAACAGCAGGCTAGCACTATTGGCTTACTATCTCTGCCTACCCACTGTAAC
**CoV-bait-1231**	AGTGTTTTGAGTGATGCAAACTGAGCGGATAGAGAAAGCGGAGCATAGTGTGATAACACCGTCTGTTGTTTCCATGTCGCAATGTGCAGCGTTTGCAACCTTAGAAAGGTGCATGCGTAA
**CoV-bait-1232**	CAGGAAAAAGAGTACGAAAAGCCAAAGTATAACATTAACTACTAGACCATTATCATTCACTAATTGTAGCATACTCGTCTAGTTGAATTGAGTCAAATGCAGCATTAGTAATGCCAACAA
**CoV-bait-1233**	AAGTAAAGGACTGCTATGAGCCATAGCAAAGTCTTGTGAATCACTACAAACAAAAACATATAAAATGTTTTTTGTAGTGGTAATATTAATAGATGCTCTTGCCTCCTCATTTAAATCGAG
**CoV-bait-1234**	GTACACCAATCACATTATCGCCATCTGAAAAAGAGACATATAAAGGCGCAATACTCTGTGGTTCACCATAAGAACCACGAGTGACAACACTCATGGCACAACCTCCATTTGGGACTAATG
**CoV-bait-1235**	TGGATGAACTGGCAAGAATTTGTTGTCATAGACCATTTCTGACACGGTCCTAACAATTTCAGCAGGGCATCTATAACACTTATGCAAAAACACATCAGGCCCAACAGCACACATCCTCTG
**CoV-bait-1236**	ACAGGTTTGTTACCAACATCCACTGCCAAGCCATTACTAGTTTTGAAAGAATCAGACAAACTTATGTATGTGGTTGCATATGCTGGTGGTATATAATGTTCCACTTTAGCACAATCTTTA
**CoV-bait-1237**	GCATGCTCTGCAGGTAAGTGGACAGGAGGCTGGCTCCGCCCCAAATGGTGGCCATCCTCCAGGAAGTGACTCGGACGTTGTCGTACTGCCTGGAGAACTGGAGCACTTGCCGATGCAGGT
**CoV-bait-1238**	ATCACCTTCCCGTGGACTGGCGTGCCAACCGCTCGGCGAGGCTCACGACCCTTGTTACCGGGCCGGAAGATAGATCGGTTTATATATCATTGGACTGTCCAGTGCGGGCTTACGTACGCA
**CoV-bait-1239**	CACAATAGATTTTTGGCTAGTACTACGTAATAAAGAAACTGTATGGTAACTAGCACAAATGCCAGCTCCAATAGGAATGTCGCACTCATAAGAAGTGTCGACATGCTCAGCTCCTATAAG
**CoV-bait-1240**	AATTGTATTAAGGTTAAGCACACGTTTGAATCTAGGACTTAAAAATTCCAGATTCAAAAATGCGCTTACCTTTATATTTAATAAAACCCTCCTCTTGGGTAAATTCTATATTATTTACAA
**CoV-bait-1241**	AAAACAAACAAGAAGGACCACTACACTCTTTTTTCATAGCAGAAATACGAATATAACCTGACTGAATGAAACCTGTTAACGGACAAGAACCTGATCCACTTTTATAACAATGTGTAACAA
**CoV-bait-1242**	CAAAAGTCTTCAACTGGCCACTCTCAATATACTTGAAGTCATAATCACTATCACTGCTAACCTGAGTACTAAAATCAAGAAAGCTTAGTGCAAAGAAAACAACAACTATGAACAGACACA
**CoV-bait-1243**	CTATAGTGCTAGTAGTACTAGGCGCTATAACATGGCGTGGGCAGATAACAGTATCACCAAGCCATAGGCCATTAAGAGCCATATTACCATAGCAAACACGAACTATGCACTTCTCAACAA
**CoV-bait-1244**	GCTACACCCTGAAGGGAAGTCGGCCTTTGACCTAGTCTTAGAGTTTTGCTCACATTATTCGAACCCGACCCACCTTCTCGGAAATCGTTTGCGACACTCCAAGCCTCGAGGCAGTTGGGT
**CoV-bait-1245**	TATAGCACTCCTGAGCCAAGAAAAATGGAGGACCATAGCACAAATCTGTCAAAACAAGAGATTGTTGATAAAGTGAATTTGAGAGTGCCGAAAACAATGCATAATTTGATGAAATTGTTG
**CoV-bait-1246**	ACGAATTACACCATCCACAAGTGACGACGAATTTGTCAAGAGAAACCAGTTGTTAAAAGCAAAGTCTGATGGTATAAAACCACCACTTTCTACAGCAAATATATTGTCAGCGTAACCATC
**CoV-bait-1247**	TGAATTGGAAAAGTGCGTAGAAGGGTAGGTATTGTACGGCTGCATGTGTTGTGAGTACTGATGTATCCATTTCTGTTGGTGTTGATGTTAATCCTAGGTTGGTGTGGGTTGTCTGTGTTG
**CoV-bait-1248**	TCCACTGTGTTGAAAGAGGAAAGGGAGATGTAGTCAAGACCTTCAAGATGTTCGTAGTGAAGGACCTTGTATGGTGTTGTGATTGTTGCTTCTTCAGTTGGCCATTGGAAGATGATTGGT

**CoV-bait-1249**	TCCTGAAGCTTTCAACGGAAGGGCTTGCTATAAGCAGGCGCCATCCGGCGTCGAAACCAGTCCTTCTTCGGTAGAAGGACGACCGATATAGCGTGCAGAGCTTTTTACTGAGCCACAGCA
**CoV-bait-1250**	AGATCTCTATAAAAGCTCTCTTCCCAGAATTTAGCTGAATTATCACCACATAGCATGACAGAATAACTGTCAAGCATGTGTCCTGTGAGGTCTTTGTACAGTTTTTCTATATACTGTAAA
**CoV-bait-1251**	TTGCCCACGTCATGTATTGAGCTTGGGTTTAAAACACTTATTGCTGCTCTAACAACATGCGACTGTACAACTCTACCGGCTTTATTTATAGACTCCTCATTATCAATAAAAGGGTACGTG
**CoV-bait-1252**	CATAGACATGCAATATACCACCATCCCGTGTTATTATACCAGCAGGTATGCCTGGCACTGACAATGTTTGAACATCTGCTATGCCAACCACCACTGGACAGTGGTGTGAATTAACATAAT
**CoV-bait-1253**	ACAGGCACACTGACACAAGGTCTGACACAATAGTAATTACCATTGTCAGAATGGTAACCAACTAAATTGTCGAAAGTATCATACACAAACCGCTGGTTTCTCAGTCCAACAGATGTGCAA
**CoV-bait-1254**	TAGAGCAAAACTGGCCATATTCTTTGAGCAAGTCTTCGCACTTCTTAAAACCATTACAAACGTACTGCTTGCAATCGACAGTAATCTTTTGAATGGTAGTCTCAATGAATTCTTGAGTAA
**CoV-bait-1255**	AAAGTGGTTTACCAATAACAGTCCATGCTTTTACAAGAACGTGTTTAGGTTTACGTATCCACAAATCAAGTAAATCTAAGGCATTGGGATTGTTCAATGTCTCATACTTGATGGGTTTAA
**CoV-bait-1256**	AGCAGACAACTCTCCATTGGCAGCTCGAAATGTGATGCCGTGGGGTAGTTGTAAGTCAGATAAGGTTATAACACCAGTGCCAGATACTCCATAAATGGTGTAAGAAGTGCACTGGTCTAG
**CoV-bait-1257**	TTACTAAGGAGCGGTGGAGGAAGCCTATTAGTGAGGTTTTCGAATTTTACATCCATGGACACATACTTAGAATTTGCTTCTGTGATGGGTTCAGGATGGAAGAAGGAACTACCAGTATAA
**CoV-bait-1258**	GCGAAGAAAGTCGCCTGTAGAATTTAAGTCAGCAAATGCAAAAAGTAGACGCATGGCATCATATGTTACATTTGTGACTTGATAGAATGGTGGCACACAGTATGCCAATGGGTATGTCAT
**CoV-bait-1259**	CTCTTTTCGAGATGAATAGAGGTGAACGCCTTGTGTGTCTTGGGTAATGCCGAACCACTCAGCGTTTTCATCCTCTGTTATAGTATAATTGTATCTGAACGTGCAATTAATCAAGTCAAA
**CoV-bait-1260**	CTAAATTAGAGAATAGCTGGTACCCAGAATCTGCACAATAAACACCAGGCTGGGCGTTTTCACTATACGGGAATTCATCCGTAAAGGATACACACAAACCAGGATTTGTTTTAAAACAAT
**CoV-bait-1261**	TTGTACTGGGATAGCAGAGTCATTATTTGGATTACGCGTGCCCATATCACCAATGCGGTTTATATTGGCACCATCTTCACCAACCCAAATCAAATCTGGGTTCTTCTGTCCCCAGCGGAG
**CoV-bait-1262**	TAAATGCACTATTACTCTGGCGGCCCCCAGGTTTAATAGGAAACCTATTCGCTACGGCATAAGGAGCTGTAGAATTCAAATAGAATGCATGCAAACAACCGTAAAAGTTGTCAGGCAAAG
**CoV-bait-1263**	AGGCATCATTTTCTTCCCGTGTAGACAAGTGCGACATGAAAATAAAGGATGCTACATTAGGTGCCATTTGCGCAACGGCAGGGAACCTTGGATCATCAACTCCAAGTTTCAACATAGTCT
**CoV-bait-1264**	TGACGTTGGGATTAGCAAAAGGATCGCACCACTTCAAAGTGGTAACCACCATACTTTTAAGCTTAGGTGGGTCAGGCACACCCTCCATCACTACACGGACGAAACCGCGCAGGGCTAACA
**CoV-bait-1265**	CGGGCACCATTACGGTTACCATTACGGCGCTCAGATTCGGAGTCAGAACCGTCAGATTCTTGTTTGAACGTGACCTTAGGGGAAGGTGTACCAGATCTAGATCTGTTCCTTCCAGACATA
**CoV-bait-1266**	AAAGACAAGGTTGTCAACAGTGCCTTCTCGCACAAAAACTTTGTCATTTACAATGGCGACTGGTAACTCACCAGACTCACCAACAAAAGAGCCCTTTTTAATCACATTATATGCGATATT
**CoV-bait-1267**	TTAACCTCAACAATTGACCAAATGGCACCTGCATAGTGTACATTGTTTTCTTGCCTAACCTTGGAAAGCACTTCAAGATTAGGTGTTACAACAATAAGTCTTGTAGCTGAGGCAGCTGGT
**CoV-bait-1268**	TAGCAGCTTGAGATGCCCGATAGGTAGTTGGGGCTGCAGAAGAAAAGCTAAGAATACTAAGAAGCAGTAACAATTTCATTGTGATTGATTAATTAAGAAACCTACAAACTAACAGACACT
**CoV-bait-1269**	CACTCCGACCTCTGGGTTTGTTAGCAAAGCTAACATTGTTGCCACCTGAGGCCATTTTGTTTAGTTTAGACTAAATGTAAAAGTTTCTCGTCTTCTGTAAGCGAATCAGCAGAACTCGTT
**CoV-bait-1270**	CCTTTAGTAAGATTGTTTATCGCTGCAGCAACACCACCACCATGACTCAAATTCTCATTAGCAGCATTGACCACAAAGTCATGTTTAAGTTGCACAAGGTCCGATATCTCACCCTTGTAG

**CoV-bait-1271**	AATTCATCTCCCTTGTAATTGCCGGACGCAACAACATCCATGCAACTCTGAACTTGTGTAATTCTCCACAAGCAAGCATTGTCAATTTTGTCCACAAACTTAAGCACCATACCATTATAG
**CoV-bait-1272**	ACTTCAACACGAGATGGTTGAATAGGTATTTCAAAGCTGTCAAAAACAGTCATTTCAACAAGGATATCTTCAGCAGCAATAGTGCAATCTTTAGCCTTAAGAACACGCACATTATGCAAA
**CoV-bait-1273**	GAGAAATAAACACAAGCATTCTTAACCTGCGCCAAATTTGAACCCACATTGTTGTAAACAATAAAATCTGCCAAAACATTACTATTCTTAAGCACTTCTTTACAGCTGTATTTACCTTCT
**CoV-bait-1274**	GGCTTATTCCGTCCAGACATAATAGGTTCGTTCAAACTATAAGAAGGAAATATTTACAACATGTGTTATTCTGAGTATTTCTTTAAGCAACAAACATAGCTAAACGCTCGCCATCTTCAT
**CoV-bait-1275**	ATGCATGTGTGAGGTCCACAACAATGTCCTGTGAACTTTCTTCTGATGTAGATGAAGACGATGGTTGCGTTCAAGATGATGGATGGTACGAGGAGCTGGGCAATTGACACTGTTGGTGTA
**CoV-bait-1276**	ATGCAGCACAGTCTATAGTCACCTTCGGAGCCCTGATTTGAATGAACTCTTCATGGTGTCCTATAGTAAAATTGGTTGGTATTTGCATATCATACATGCCATCAATGGCTTGAACACTAT
**CoV-bait-1277**	AATTAGTTGTGGTTTGGAAGGCTGGGAGTAGATGCATGCCACCGATTGTCCAGGATGTTGCTGTTGTTTCACCAAGGGAGACATGTTTACCAAAATCTTCGTACGCTTGTTGTTCGTTAG
**CoV-bait-1278**	CGACTCTTACCGCCGCTTCAGGTCTGGGAAGGTCCTTGAGGTATGTGTTCATCCGCCTGTTAAATTAACAAATTAGATTTTTTTAGCCTACGAAATAAGCCTATAAGCAAATGCAAACCT
**CoV-bait-1279**	GCCAACCTGTAGTCAGAAACATCAGTCCAATCGGATGTAGCAATGCGATTAAAGTCTTCAACATCGGGTGAGCCGGTAGCAGAATTTTTGTACAAGCCAAAAACATTACCAGCAGAGCAC
**CoV-bait-1280**	AAGTGACAAAAGTTTTGTCAAGTCTCTCATCTCGAACAAGACTGGCTGAATAAAAACCATCAGGGAGATCAAAAGACAATTGAGAACACTTAAGTTTGTGAATAACATCGTCACAATATA
**CoV-bait-1281**	CTTACTGGTATGCGTTTTTGCTTAGCGGTTTTAGAACAAGACAAACAACTAGCCTTGTCACAACCATACCAATAATGCTTTAAGAAGAAGAATCCTTTTACAACAACCATTAACACAACA
**CoV-bait-1282**	TCCTGCAAAGGCATTTCCCAGTTAGTGAGTCTGGGTACATTACGGGGGTCGATCCAATCAAGGGTGATCGACACCATAGATCTAAGCTTATTGGTGCCTAAAGCACCCTCCATCACTGGC
**CoV-bait-1283**	CAGGTGAAGAAGCAACCAGAAGTGCAGGATCTGTGACAAACTGTAACAACTCATTCACACTCAAACGAGTAGAGTGCGTGTTTATGTCTTTGTTCCAAACAAGACCCAACTGCTTGAAAT
**CoV-bait-1284**	CCAAATCAATAATAACTGTACCTTCTGGTAACATGTGTTTTATAACATCTGTACCAGGAGCTGTACCTTCAATTGAGGCAGCTCCAAGATGTAAAACAGTCATATTATGAGGAACAGCTA
**CoV-bait-1285**	CTCATGGATTTCAAGGGTAAACTTCAGTAGGAGGCAAGTGTATGCATAACATGCATCCGTGGCTTTATATCATCATCAGATCCTTCCGTGATTACCAAAGTGCCCTATTCACTGGAAGAG
**CoV-bait-1286**	AGATTTTAGAATAGTGCAAAAGTCTGGCAATGTCTGGTGCCCTAATAAGGAGCCTAGGGAAAACCACCACTGAATGTGGTGTTACCTAGGTAGTCCTCAAAGAACACAGTAAGACCCCGT
**CoV-bait-1287**	AGTGTTGCGCACTCACCTAGACCGCTAATCTCTTCACGTAAGCTCACTTGTTAAGGTACTATGAACATCTTCAGCCCGGCGCTGCGCAAGGTTGTTTAATCGTTTACAACCCATAGAACA
**CoV-bait-1288**	AGTGACAACACCAGTAATTGCTGAAAAGTTGTCTGCAAGATTTTGCAGAAAACTTATGAATGCAGAGAGCGCCCTGGACACACGGACCTTCAAACTGGAGAGGGAACGCTCAATGGTTGC
**CoV-bait-1289**	TTACCGCACCACCATTTAATTCACGCGTTAATTCACGTAACGCACCCGAACCATGTTTCGTATTCCAATTTTGTTCATAATCTTCTATCGGATCCGTACCTAATTCATCACCTAAATCAT
**CoV-bait-1290**	GGAAAATAGTACATTAAAGTTTGCACAATGAAGGATACACCTATCGTCCAAACAGTTAATACAATTGGGATGGTATGTCTGATCCCAATATTTAAAATAACGGTCGAAAAGACATAATCT
**CoV-bait-1291**	TTAATGATGCCAATAACGACATCACAATTTCCTGAGACAAATGTATTGTCTGTAGTAATTATTTGTGGAGAAAAGAAGTTCCTCTGTGTAATAAACCAAGAAGTGCCATTAAACACAAAA
**CoV-bait-1292**	ACGATACATTATGCGATTTCGCTACTTGCGCATTTATATGACGCGCATTACAATCTATACACGCACCTAAATCACGCGGCGTCATATTTTCTACTTTATTATACGTTAACATAAAATTAT

**CoV-bait-1293**	AGTAGATGCCGTCCTTGAAGGGGATCACGGGGTTGTCGAAGGTGTGGTTGATGGTGTGGAAGCCGGTCACGTTGGAGTAGAAGGGCAGGAACAGGTCCTGGGTCAGGTACAGGGTGTCGC
**CoV-bait-1294**	TTTTTATAATACCCATCCAATTTTGGTTCAATCTCTGTGTAAGTAACTCCATCGAGTTTATACGACACAGGCTTGATGGTTGTAGTGTAAGATGTTTCCTTGTAGAAAACATCAGTCACT
**CoV-bait-1295**	GGCTAGCTGCCATCTTTTATTGAGCGCAATTATTTTGGTAGCGCTCTGAAAAACAGCAAGAAATGCAACGCCAATAACAAGCCATCCGAAAGGGAGTGAGGCTTGTAGCGGTATCGTTGC
**CoV-bait-1296**	GACGGTTACGCCGCTCCATGAGCTTATCGCGAATAAATACCTGTGACGGAAGATCACTTCGCAGAATAAATAAATCCTGGTGTCCCTGTTGATACCGGGAAGCCCTGGGCCAACTTTTGG
**CoV-bait-1297**	GCTCATATTTTCCCAATTCTTGAAGGTCAATGAGTGATTCATTTAAATTTTTAGCGACCTCATTGAGGCGGTCAATTTCTTTTTGAATGTTGACGACAGAAGCGTTAATGCCTGAAATGT
**CoV-bait-1298**	ACACAAAATTACAATCACTCAACTTAAATAAAATCTTCATCACTCAAAACAAAAAACTTTACCATCAATATATACATAACAAAAAACAACTACAACTACTCATACCTCTTAAAACACCAA
**CoV-bait-1299**	CATCACAGGGTTCTGAATATGACTATGTCATATTCACACAAACTACTGAAACAGCACACTCTTGTAATGTCAACCGCTTCAATGTGGCTATCACAAGGGCAAAAATTGGCATTTTGTGCA
**CoV-bait-1300**	ACAACCAAAAACAACATGCTTAAAGAAACAAATAGCCTTATATGACAAAAACATAACAAGTATCTCATCGCCAAAGATGTCAAAAGGGACAAGTTGAACTAGCCAAAAGGAATCCTGCAA
**CoV-bait-1301**	ACAGCTGTCTTCCTCTGTTAAACACATAAACAGACGGGGACAGGACCAAAGTATTACACATACCGCAAAGTTGAATACAAAGTTTAATAGTTGCAAGAAATGCAATGGCAACAATAAGCA
**CoV-bait-1302**	TAATAGGTGCTCCGCCCTTAAGTCTGAATAAGGTAGGAACAGCTTGGCCATTAGTGCACATTGGATAGAAGACAGTCTGACCATCCTCTTCACAACGCACAAAAAGCTTATCACTAATAA
**CoV-bait-1303**	TTAACCTATAAAAGATACTCTGTGCAAATGGAATAGCAGCAAAGGAGGATAAGCCAGCAGTCCAGCCAACACCTGCTATGCTGCCAAGCAAAGATGAAGTATACGCGGCTTCCATATTAA
**CoV-bait-1304**	ATATACTGTAAATAGACCCAGAATACATTTTGGTATTCTAAATCTTCATGCTTTGTGAGAGGGTAAGCATCTATAGCTAACGACACAAACCGCTCAACCATGAGTGTACCGTCAGTCTTG
**CoV-bait-1305**	ATATGAGTCCCACTGACCAAAATGGCCGATAGGATAAAATTATAAAGCATAAGACCATAAAGAATTAGGGTCTTAACTATACTACGCATACAAGTGTCCTGATTAAGACAAAGTATGAGT
**CoV-bait-1306**	CCACGGCTGTTTGCAAAGGACCAGAATCATCAGAAATACGCATGCGCAGTCGGGACTGTGTCATACGTGAAAATTGTTGCATCATTGTGCCAATGTGCTCACAAGCAACACTGCCAAACA
**CoV-bait-1307**	TGTAAGATATCATCTAAAGTGTCCTTAGACGTGTAGCAATAATATTGCACACCGTCAACTGTAAGAAACTTCTTAGTATAATCAACACCCTTGTTCCTAAGAACTTTAGTGTTAGCAGAG
**CoV-bait-1308**	TTCCACAAACCAATATGTTTTACAAGGCATGCCGGCATTTTATACTTTTTAAAGTAGGCTTCAACAAAAATGGTTTTGTCGTCCTTAAGAGCTCTGAAGCACAAGCTTTGCTGCCTAAAG
**CoV-bait-1309**	CATCGTTGGATTCGTGGCCTCACTGCAGTTGTACCAGGCCTGAGAGCCCTAGGAGAACCTTCGGACTCGCGTCGCGGCAATCGCTTGCGGCCTACGCAGGGCAGTCTTATGGGTAGCAAA
**CoV-bait-1310**	GCATCATAAACAAAGCGCTGCTGTCGAACACCTACAGCTGTGCAATTCTGAAAAACACCACGGCCCGAAACACCATAGAGGGAATATTCCACGCAATTGCCTAATTGAGAGGCAATTTTT
**CoV-bait-1311**	GTTAAAGAGTTTCTAAGAGCTGCATAAGTGTCCTTGTTAATAACAAAGATATTAGAGGCAGCGTCCTGGAAACTACAATTAAGCTTACCATCAGTAAAAACTTCTACATGTTTCTTACTA
**CoV-bait-1312**	CCGTCTTCATCAACATCATCATCTGACGGGCAATAGTCAGAAACCTCTTCCACATATTCCAAGTTGCAGGCATCCTCAACACACTCTATATTACTATCAACCGACAGGTCATACTGTGAA
**CoV-bait-1313**	GATGACAAAACAATATCCCCTGGAGAACAACCATCAACACCAGCAGCACCCAAATGCCAAATTAAAGAGTTGCGTGCAACCTTTACATGATCTTGAATAAATGTGCATATTTGGGTGTAC

**CoV-bait-1314**	ACAGCATTTAACACACTAAGATGCTCAAGTACATTTTCAAAAAGCACAGCATTACCAGGAGCTATTGACTTATTGGATTTAAGATATTCTCTAGAGCGGCTTGTTAACTTGCCACCTGTG
**CoV-bait-1315**	AGGGAGTAACATACCGGGCCTCGTAATTGGTAGATCTCCTTATTATAGCTTATGAAATAACGGGTATGGGTGGGGGCAAGTCAAATACACGCATTGATCCGTTCGTCGAGAATATCCCGA
**CoV-bait-1316**	TTGTGGTAAACACAGCAATCAAAAGCAGCATCAGAGTAATCATCAAAAGATGGAGTGGCTGTCAAGCCATCAACACTCTGAGTTTTAAGCACACGCCAGCATGCAAGTCCATCAACAGTG
**CoV-bait-1317**	GCGCCGGCCTGACGAACTCCCTTGACTTTTGTAGTAACCAACTTAGCCACTGCATTACCAAGAAGAACGTAATCACCAACGCGCTTAAAAGTTTCACCCACAACCTTATAGGTGTCAACA
**CoV-bait-1318**	GAATCACTGGCAAAAGCCAGGGTCAAATGGTTAGAACCCATAGCGACCTGTTATCGGTTTCAAGCACGACGGAACGTACTATCCACACTGCACGCAGACAGAACTACGTGCAGGTCAGAC
**CoV-bait-1319**	AGCCACTCAATTGACTCGATGGCACCTAGAGACTGAAATTGATACGGAATGTCAGTGCGTGTAACATGCCAAACCTTGACATGCTTAACACCATTAACAATAAGTTCAGTCTCCTCACCA
**CoV-bait-1320**	CCGCAACCACTAAAATCACATTCCAAACCACAACTAACAACAAATGCTGCTAGTGGTGAAGGATCAAAATTTTGTGGTTCGGGTGACGACTTCAAAGGCCATTGTGACACCATCACAACC
**CoV-bait-1321**	AAGGTGGAAGCGACCCAACAATTGTTAACCTCCTGCTTAAGCACACGAACACCAGCGTGGTCGTAATACTCAAACTTAAAAGGATCAACAAAAGCAGATGGTTCATCAACAATAAACGAC
**CoV-bait-1322**	TTAACATAAAACTCTTCAAACTTGGCATCGACAACAAAGTCTTTGCAGAGCATAACAAACTGCTGCTTGTCTGTGATATAAGCAGGGGCCTTAAAATTATAACTATGGTCAACCTGGCAC
**CoV-bait-1323**	TCCTGCACAGGTTCAAGCGCAACAGTATCAAACTGAACGCGCGAACAGACAGTTTCAATATCACCGCCTTCAAAGACGAGAGCATCAACAGCACCAGCCTCAGTTGGTCTATTAAAGGCA
**CoV-bait-1324**	TCACCTCTATATATAGTTTCATATAACTCCCTATGCAGGTTAGCTACATCAACTATATCATGAGAAGTGGTAGAAGTGGATAAAAAGCTAGCTACATTTGCTGTAACTACTTGTAGAATG
**CoV-bait-1325**	AATCAGAACTAGTTGCAGCTCTAGGCTGGTACATAGTTCTGGGGGTCAAATAGAACTTGTCATCTAGATTACGAAACAACGTCAACTGGACATCTTTAACGACAAGTCCAAAAGTGCGCT
**CoV-bait-1326**	AGGTGACTATTATAAATGGTGTACCCATGAATTTTAAGCCACTCCTTAATATGGGGGACTGCCATGTTAACAGGCCTGGTCATGCCATAGCGCTGCAGCTCATGAGCATTCAGAACTAAT
**CoV-bait-1327**	GTGGACCAACAACATTAAAGACCTTAAGACCTAATGCCTCCAACATGACACCACGTCCAACTTTAATGGGACCGTGTGCTTTAATGTAATCATTCGAGCACTTCTGCAACATGCCCTTGG
**CoV-bait-1328**	AGAAAGATAGTTGTTTAAACCTTGTAGATCAAAGGGACAACCAGCTGATCTGCAAGATAAAGAACATCGAAAAGAACCACCACCACAGGCAAATTTTAAATAAAAATTAAAATAGGTGGT
**CoV-bait-1329**	TGTTTTGATAAAAATTCAAAGCACTACTAACAAAACAATTGTTACTCTGCTGTTGAAGGTGAACAAGACCTTTAACACGTTTAGTGGTCCAGCGAACCTGACAATTCTTCAAATACATAC
**CoV-bait-1330**	AACAGCAAGACCCTTAGAACATTTCTTATAATCTTGATCTACTGTACCCAATCCATTAGTTACTACTTTATTAAAAAGTAAATCTTCTATAGCTGAAAAAGTACGAGGTTGATCAGCTAA
**CoV-bait-1331**	GACTTTCGCTTGACTATAGTTCTAGCTCTAAGTTTAGCCTTCATAAACCGTAGTTTACGAACAAGGGCTATATTTAGTGCAAATGATATAATAAGTAAAATTGACAAAACAATAATGAGA
**CoV-bait-1332**	CATCCTTAACATGGACCCAGGTAATCCTAGAATTACCTTTGGATTCATTAGGAGGGAATTCACCATATTTAAGGTTGCCACGTGGTCCAGTGCCAGTATAATAAAAGGCAAAGGACGGTG
**CoV-bait-1333**	CAGTTGTTTTAATGTACTTATAGCCACGCACAGTGACAACAGTGCCCTCTACCACATCAACAGGTAATTGTTGGTAATCAGGCAAAAGGTTAACATCCTCATCATAAGGATTACCAACCT
**CoV-bait-1334**	CATTGACTTCATCAGAAAACACATCACAAACATAACTAACTGTTATGTCATCAGAAACACCAGCTAAGAATGCCTGTTCATGTGCTTGTGTACCGTCAAACATGTAAAGATGTTGACCAT
**CoV-bait-1335**	CTTAAACATTGAAAACCCAAACCCTCTTCTATAACGCACATCAGCTGTGAAAAACCCTGTGGCAGGATAATAAACAGCTTGCTGACTTGTAGTAGCCAATCTGCAAGTCCAAAACTGAGG

**CoV-bait-1336**	GCGCAACTGCAATGTAAGTTGGTTAAGCGCTCTGCCTTGGTGGTTGACAACCTCTTGCACTTTATTAAGAGCACTAGCAACAGTTTGCACAGCCTGGGATGTCTGTGACAAAGCGTCATT
**CoV-bait-1337**	CCCGGAACCAGACGGTAGGCTAGGTAGGACACTAGAAAGGTTGTATTCGCCGAAGCTAGAGATGTTGGCTAACATGAATGCATTGTCATCATACGTAAGCATACTGCTGACCTCAGCTGA
**CoV-bait-1338**	CAATAGCCTATAAGCTTGCTTTTATCTTTCTTGTTGGTGGGTACCAAATTACGAGGTATCACCTTCCAAGGTTGTTCACTATCAACAAGCAAAGGGCTATAAAGGGAATAAGGTATTCTG
**CoV-bait-1339**	CCACAGAACCCATAACGACTAGATTGAGACTTCACACATTCGTTAACTTTCTGTGTAGCCAACTGCCGCGATGCACGCACTTCTGTGTATTTGGTAAGGGTCTGCGCTACAAATGCATTC
**CoV-bait-1340**	AAGTACAGCAAATAGAATAGCTAGAGTAAATTGAGCAATAGTTTCAGAATTATCGAGACCTTTACGAGAGTAAAGTTTAGATGTATGATAATTCCTTTGATAATGTGCGTTAGTCTGGTT
**CoV-bait-1341**	TAAATCAGGATAAGTAACATTAGGAATACTATTAATAATGCTATCTACTGTAGAATTTACATCAACATAATCAGGGATTATGCTGGGTAAATCTTCCTGCGTAACATTAATATACTGAAC
**CoV-bait-1342**	AAGACCTCCACAGCATCAGAACCAACTGCTGTAGCTTTATAGAGACCAAACACATTGCCATTAGCACATAGCGGGAATGACAATTGCGGTTTGTGATCTCTACAATAGTAACTAAGGCCA
**CoV-bait-1343**	TTTCCTCCTCCTTGGCAACACCAATATTGGTGACAAGGCTAGATTCCACTTCCTCCTCTAAATCAACAGCAATCTGTACCTCAACCTGCTCCTTAACAACGCGTTCCTCCTGCACTGGAG
**CoV-bait-1344**	ATTGACCTTCGCAATATAAATTTCCAGCTTTAATAATAGGAGCAAGTACCATAGGCACTGTCTCCACGCTAAAATGGCAAACTTTGCCATTCTTCAAAGTAATAGTTCCCACACAATTAC
**CoV-bait-1345**	GTTTGCTGCAGGCAAACTATAATAGAGTTGACAACTGCTAACAGCAGTGTCAATCTTATAATTAAAAGATTGTAAATAACCAGATTTACCCACTTGCAGATCAACCTTTCTACTATTGGG
**CoV-bait-1346**	TATAAAATTACCATGTCGCTCCGGTACTTCTAGGAGCAAGCCGCATGCCTATCATGGGAGTGCTCCCTACCGCTTAGTGAGATTAGACCACTTCCAGGAACACCGAAAGCATCATCAGCA
**CoV-bait-1347**	AAAGTTTCAGGTAGCTCTCGGAGGCTTTCACCATTGCCAAGCTGTAAATAATACAACGGCTGTTCTCCTGTAGCCTCAACATAAATATGCACAGAACAATCATAAACTTGAGCACCAGCT
**CoV-bait-1348**	CCTGGGAGGCACGGGTATTAATGAACCATCTGCACAAATACCAAAGCTAGAATAAGTTAAGACAGGTTCAGTACAGTTATAGGTACCATTACTGGCGTAATAAAAATTAGGCATTTGTTT
**CoV-bait-1349**	AACAGCAGGTGCAACAACCAACATCTTAGTTTCATCTAAGACTGCGGGCTTCGTAGCTGAGATGTCACTGTCTGCTACAGTAACTGAACCCAACTGCTCACCATAAGTCTTAGCATTTGA
**CoV-bait-1350**	AAAGTGTATTCAACAAACACAAGTAGCACGCACTCAAATTTATGTGACAACTGCCCGCTGTAAACACCCATACATTCACGAGGGCCGTTATATGAGTACATAAAACAGCACACATTACTA
**CoV-bait-1351**	GGTGCATCATACTTACCATCAAGTACCTCAACCTCCATACGTTGTTGGCGAGGTGTGAGCATGGCACCAAGTGTCTTAGCACAGACTTGTACTTCTGTTAGTACGCCCTTTGCTGAAAAG
**CoV-bait-1352**	ACCCAATTGACCTGAAAGCTAGCCCATGCATCAAAAAGTGACAGTGCTAACACAAGAGGCCAAAGTATCCATAGAATAGCCATCTTGACACCATACAAGAACGCAGAGTACTTGTAATGG
**CoV-bait-1353**	TAGCATGTGGCTGTTCTACCACAATCACAAATCTTGACAGGCCCGATCTTAACGAAATAACGCATTGTCGTAAGCTCAAGACCGCCAGCCCAAAGCACGAACACAAGAGTATCTGACAAG
**CoV-bait-1354**	CCATAAGCTTAATATTAAGATATAGAGCTTCCATAGGATTTGTAGGACACTTAAGATTATTAGCAACCATATATTTAAACTCAGCCTGCGAAACACAATAGTCATAATATCCAAGACACA
**CoV-bait-1355**	TAACATATGTACCAGGTATGGTAATGGACCAATCATCCGCTACCATTCTTTGAGTTTAAATGATACTGGTGTTGGTTCATCGGGATATCTTTTCTTAGAAAAGAGTCCGAAGCAACCACC
**CoV-bait-1356**	GTATGAGTGCACAAAAGTTAGCAGCATCACCAGCACGGGGTCCGCTGGCTGACCACACAATCATTCGCTCCTCTACCGGACGAGCTAGTAGTCTTCCCTAAACATATGCCCGACCAGTCG
**CoV-bait-1357**	CGACGAGATTCACATTGGCATTTTCAACGCGCTTGGAAACCACCTTGGTAGTACTCCCAATAACAAGGCTTGTGTAACGAACTTCACAAACACCTGCCTGTCGTGGGCCGCGAAGTTTTG
**CoV-bait-1358**	CTTAGCACCGTTTATGACACTCTCGTACCAAGAGACAAGCAGAGTCTTGAAAAGCTTGAGGTTTGTACCAGTGACATCCTTGCAGCAAAAGTAACGGTCGCTAGCATCAATACAAAGAAT

**CoV-bait-1359**	AGCAAGGAGATGAGCCGGTTCACGAATTACTACATCTCCATCCAACAAATCCTCGATAGGTTGCGCCACATACTCAATGCTACGAATAGTAGCAATGGTTTGCAGACGCATCGCAACACG
**CoV-bait-1360**	TTAAAAATTTACCAAATGTAGTGGCATTGTAAACACCTGGTTTCATCTCCAAATAATCACAAATATACCTATGTAAACCTGCTGTTCCATTAGTGTAAAATCCATGGTAAAATGATGGTG
**CoV-bait-1361**	GAGGTGACAGAAAGGCGGCCAGGCTCTCACAAATTTGAGGCGGAAGAAGGGGGCCCCAACGCATTTTTATTCATACAACAGATCAACGTGCCTCATATGGGTTTCCACCTATCATGCGTG
**CoV-bait-1362**	ACAACAGAAGGCAGCAAGAACATCTGTAAGAATAACAACTTGTGCTTAATAGTAAGCATAAGCAAGGATGAAATAGCTGCTATAAATAAGAACATAGGTGTTACATAACCTGGATTTATC
**CoV-bait-1363**	AAGTACAACATGTAGTATGCTTGTTACCTAAGACCATTGCTGCGATCATGCGAAGCATATTAGGCATGGCTCTATCACATTTAGGATAGTCCCAACCCATTAAACTTGCATTCTCAACAC
**CoV-bait-1364**	CACGGCCACTATAACAGCTCCTTATCGTATAAGTCTTGTTAGTGGTAAGATCACGAAAACCATTCAAATTACCATTAACATCATACAGAAGGGTTTGCCAGCTATTATAATAGTCTGCCT
**CoV-bait-1365**	AGTTGCGTAGGGTGTAATTTGACGCACTAGAACCAGGACAAATGATATAATTATCATAAGAGTTGCGGACAGGACAACCAGATGGATGACATGGTGTAAAAACACTATCTTGTATAAAAC
**CoV-bait-1366**	ATTTAAACTCCGCGGGGCTAACAGTAAAATCATAAACGCCTAACGTGCACTTACAAAACCTGTTAACCCAATACAACAAGCCATAATACACACAGCTGCAGAACCCTAACACCATATAAA
**CoV-bait-1367**	GTAAGGTAAATATGTAATATTAGATGTCACAGGTACAAAAGTATCTATGTCATAGAATCCGGTAGAAAAATTAAATTGCTGATAAGAGCAACGTAACTTATTAATAATAGTGTTTCCTGT
**CoV-bait-1368**	AATAAATTGCTGATAATGGAAGATTAATACCATCATGGCTTTGTGCTAAAGCGTGTACAGCCTCAAGAGAACATGAATTAGGTTTCTGGGTAACGTATGTTACTTGTTGTGAATTTGGGT
**CoV-bait-1369**	ATAAATGATACCATCAACTTTGGAAGGATCAATAGGATAAGGCCACATTAATTTAGAAAATGCAGCTGCGTCAACTTGTGACTCCAAACACTCACTAGTATTACTAGCTGGACTCATGTC
**CoV-bait-1370**	CATCGTCGTTCTTCTGAACTGCGAATCGACTCATTCCGAAGAATGCTGACGGTGTAGGGGCAAGTTGAGCAATTTGGGGCCATTTTGGATAGTCTGTTCCCAATTTGTTGAATTCTTGTG
**CoV-bait-1371**	GGCATAGTTTTGATGTTAGCAAGTTTAAGGAAATCCACACTAGAGAACACAGACAATGGATATCTGTCGTAAACACGTGTGTCATTTTTTGAGTAGTAAAGGTGTACAAAGGTGTCTTTA
**CoV-bait-1372**	TACCATCAATGGTTGTAGCCATTGGCATTACAGGTTTACTAGTTATATAAGACACAGTACCCACTAAAAAGCAGGCATTGTGGTAGCTATTATAATATGCTCGACCTCAATGGGTCTTAT
**CoV-bait-1373**	AATGCTTGTGCAACAGCACTAGCATAACGAGGAATGTTTTTAACAAAATATATATCACTCTCTACTACTATATAAGCACCATGCAAAACATGAAATAAAACCCATGCAAATGCAAGAGCA
**CoV-bait-1374**	AATTTGCGCAGGTTTCCCTGGGCATGTTTCCCTTTAGGATCTTCAGTATACAGATCGATTGGTTCATGAACCAAATCATCTGTAACCGTATCACGGGTGTTAGTCCAGGCATAGACTCCA
**CoV-bait-1375**	TTCATAATAGAGCCTGGCTTTTCCAAATTTATTAAATGGATAGCCAGCACTCTTATCATAATTATTAACAATGACTTGTGATGCCGGTATACACCCACCATCATAAATCTCAAAATACTT
**CoV-bait-1376**	GAGGCAATAATAATATAAAACCTCATAAACACATGTGCTGGTAACATATTAGCTAAAGACACCAGCAACCTAACACTCCAATGTAATGTACTAAGCATAAAAAGCTCTGGCAGCCAAGTG
**CoV-bait-1377**	ATCATCACCAAAACAGACATCCACAGCTTCACAAACACTGGCGACTGCATCTTCTGCGTTGCAGGGCCATTGTGAAATCATAACTGGCAGGTCCAAATCATTACCACCCTCTTCATCATA
**CoV-bait-1378**	AACCACTGAGTATGCTAGTAAAACCAGCATCCCCATGATTGATATCATAAACACCAACATAATTGGTAGTAGAATTACGAAAATAACAGTACGGGGGTTGACAAGCAACTGCCGTCATGT
**CoV-bait-1379**	GATCTTGACCAGTCACTTGGTAGCGAAATCTTTTTTATGCGGACAAATTCCGCAGATAGGGGAATTCTTGGTAGTGTAGACTAGGATAGCGGGCCGGGGCAGTCGTATGACGTAAGGATA
**CoV-bait-1380**	GTCAGGGAAATGCATAAGGATGCCATATCGAGAAATACTAAATTTACCGTTCGAAGGCCCGAAAGACACCGAGCAATATGACCGGCAGAGGGTCATAAACACAGACAGGTGCCATATAAA

**CoV-bait-1381**	CATAAAATGTCACTGCCAAAGTAACCAAATAACTTGTATTCCAAAACAAAACATTCTTCAAAACAACCGTACTCAAAAGCTGTACAACAAAAAATAAAGGCAATACAACACTAAAGGGAT
**CoV-bait-1382**	TATGACCGCGTCCACCACAGTGTGTCACAAAAACATAAAAACTTGTATAATTACAGTATACAGTACAAACGCCTTCCTTTGACCAACGAAATCCAGTTGCTAATGTAAATGCGATTGCAC
**CoV-bait-1383**	TAATTAGGTTGTCCATCAATAAACTCACAATCACTCTCATCATAGTAAAAGAATGGCATAGGTTTAAGCTTAGCAAATGCTCTTCTATCATAAGCTGGTGTGTGAAATGCATGGTTGTTA
**CoV-bait-1384**	GGTGAAAGGCCATTTGAATTCATGTAGCGCAACTCCTGAGAGCTGACTACGTAATCATATGTGCCTAATGTGCACCTAAACAACCTGTTTAAAAGGTTAAACACACCAAAGTAACAGGTA
**CoV-bait-1385**	CAGAGGATCCTCTGTATACACCACATATTCCACAAAGCTGCGTGTCAGCACGAACCAGTCAGAGCCACCATCCACCACGATGCCTGCCGGGATCTGCCGTTCACCCAGGCGCCACATGTG
**CoV-bait-1386**	GTACCTTAGAAAGGGGTTTGTCATTAGTAACCCTAAGAGGGGCATAGAGGGATAATGGCACCCGTTTGCGGCCACGATCCTGAAAACTGACAGAAGCCATAAAGTTTCTGTTTAGACTAA
**CoV-bait-1387**	TAAGGTGAAATAAATACTGCTTTACTCCAATTTGGGTTAGCAGTTATAAAAGTTTTAACAAAATTCAATTGAGGTCTATTAATAGCAGAGCTTGCATCATGCGTAACGCTGCCTTTAAAT
**CoV-bait-1388**	TGAAGTTGCAAAGTCAGTATACTCTATTAATTTCGCTGGCGTGTAACAAATGTTACCAACAGCGCTAAAAACTCTAGGTAAGAAATGCAAAAAGTCACCATTGGAAGCACGCCTTACAGT
**CoV-bait-1389**	TGTCGTATATGTAGTGTTCAGAAAAGTTACTTCACAACTCGTAATTTGAACGAAATCACTCATTTGAGGTTTTCTAGGTTGAAACATATTACGAGGAGTTACCATATACGTATTGTTATA
**CoV-bait-1390**	AGGTATAAAAGCAGTAAGAAGTACTCTGAAATTAGTAATATTGAGGCCTAAAGGTAATTTTAATAAAGGCTTCAAAAGACTAAATCCACTAGGTAAGCCATTTGCTACATCTATATTCTG
**CoV-bait-1391**	CATGATACAAGTGGATGATGTACAGCCATTAATAACGTGTATATAAGCTTTCCACACATAGTAGAAAGAGGCAAAGAACACATACATTCTAACCATAGCAGAAATGGGAGCCATTTGCAC
**CoV-bait-1392**	TATCAACATCTGAAAGCTTATCAAGAATCAGCAATGAAGCCAACAATCTGCCCCTAGATGGTAAAAAGTGATACTCTCCATCTGTTTTAATTATATGTGCTGAACAAAATTCCTCAATAT
**CoV-bait-1393**	TTCATGTAGCTGTGTTATATTTTTTGATATCTCATAAACTTTATCTTTAAAACTATAATCTAACACCAAATCCGTCCAATTCACACCATTGGGTAAAGTTACATTAAAAGTAACATTAAT
**CoV-bait-1394**	GTTTTAATATGTCTGGGTCCGTTACGTCCATCAGCGTTATTTATGGGGTTGTCCGCCTCATTCAACTGTGCAGTAGCGAGGCGACACCCAGGAGTCACGTCCCACTAGTCGCCCCAAGTG
**CoV-bait-1395**	AATGACACAAACTTATTAGAGAAGCATGTGTCAGTACTACGAATTACGTCAAAAACGCCATTGCGTATTAACCTAAAGTCTGTAGCTGGTACGTCAACTAGAGTAGTTGGTGGTGATGGA
**CoV-bait-1396**	AAATTATTCACCACTGTGCACAGGCCAACACTAAGAACGCCAAATGACATATCGCCAAACAAGCGCTTAAACTTAGTGAAGGCAAAACAAGCCATAACAGCGGTAAATGCTACCACACAG
**CoV-bait-1397**	AACGCGCATAACAGACTTGCTAAGATCATAATCATACAAGTCATAACGTTGACCATTTGATGTATAAAAAATTCTTACCTTCAACTCTAGTAACAGTTAGCATTAAGGCCCTGGCTGCAGG
**CoV-bait-1398**	CCAGAACAAGTGGCCAAAGAAGCCAAAGAATGGCCATCTTTAAGCCATAGAGAACCCTGGAATATTTGTAATGTCCATACTGCAACACTACTAAGAGTATTGTAAGTATGATATTCCATG
**CoV-bait-1399**	GCTAATATACAAAAATTCCACATTGTCCTGATTACAACCTTCAACACTACAAATGTAGTTGTTGATGGCAATAACACGCTTATGTGACGTTCTAGTGACATGCTGATAACAACAGACACA
**CoV-bait-1400**	TCCTGAGTGCCTGGTACAAACCGCATGAGAGCCATGAGGCGCGGATCTCCCAAACCCGTCTTCTCAGTGTCTGGAGAACCAACATTAGCTTTAGCTGGCGAACGCTTGCCAAAGGTCCCA
**CoV-bait-1401**	AAAACTGCTTATAAAAAGCAGCGTTATTACCTGTTGTGACTGAGCTTAGATCAGCCTTGCTAATTTTCTCCACCAAATAAATGTAGGTGGCTGTGTCAGGCAGCTCCGGGTCTTGGAATG
**CoV-bait-1402**	ACCTGCGGTGTTACCTCATTTACGTTAGGCAACTCCACCTGTGGTACTACATCATTTACAAGTACCTGATATGGATTAGGTGTCACTGGTATCGTTACAGTTTGAACTGCAGCTTGCACA
**CoV-bait-1403**	TACGACAATCATTAAAGGTCAACATGAAGGTTATACCCTTAACTTTAGTCGTACGAATAATGTACTTCCTAGTTTCTTCAGAAAGGGCAATGAAATCACGTACAAGCCACACCACAGGTA

**CoV-bait-1404**	GTGTGATTATCAATACATTGAAATGAACGAAAGAAAGAATAATCACCTATAGCTCTAAAAGTATAATTGTAGCTAACTGAACCGTTAGCACCCACCATTATATAACCAGCTCGCAAAGGT
**CoV-bait-1405**	TCCTTCCTTGCTCGCATAGGAGTGTTATATTGCTGCTCCTCCGGAGACCCGCCTCTGCCTCCTTTGGCCGCTAACCTTCTGTAAAACGACGCCCAATCTAATCTTTTATACCACAGCCGG
**CoV-bait-1406**	GCTTTGTTTAAGACATCATTAACAGTGTCATCAAATGCCTCCGACACTACCGTGTAGGTTTTAATGACATACAAAACTTGATCTCCTGTAACACTAACATCGAGATTCTGAATAGGTGCA
**CoV-bait-1407**	TCAATGTCAAAACCTGTGTCTCTAAAATCCATTAATGTATCCCACGTGAATGTGACATGTTCATGCTCCAGAAGACCTAAAGCATTTGAGGCAAGAGCTGTAACTAACAGACTTAACACT
**CoV-bait-1408**	TTACCCATAGAATTAAAAGGCAATAAATGTAAGAGCCAAACGTTGTCCTGGTATCCGAGGGCCACACCAGCAGCATTAATGTACTGCATAATAAAATACGTGATACCAATGCGAACAAAG
**CoV-bait-1409**	TGATTAAGGCATCATGTAGGGGCCTGTAGAGGTTAACATACCACATCCAGGGGGCCCAATCATCTTTAACGATGTACACCACTAAACAGTAAGAAAAAGGCACCAAGAAAAGAAAGAATA
**CoV-bait-1410**	CACTTACAATATAAAAATTATCTACAAATGAATAGAAAGGATTAGAAAGATCAAAGTAGAAATCAATATGATCATGTGCAGGATCTTGAAAGCCAGGCCATGTGCAATTGAAACCTTCGA
**CoV-bait-1411**	CACTAGGTAGCCCAAGCAACGGATGTAATAAAGTGAGACACAACAAAACAAGCAGTGATACTAGCAACAATAAAATCTAATAACTAAAACCTGACAGGTGGCCAGGTGCCATCCAGGGCA
**CoV-bait-1412**	GTTGTGGCGGTCGTCCTGTAACTAACAGTGTCTGACCCATAATCGAGCTTCTCGAAAGTGAACTCACCAACCTGAAACTTAGAGTCTTTACTTATCTGGAAACAAGTAAAAACAGAGTTC
**CoV-bait-1413**	GTGTAATCACCACAGACAAAAGCCGCACAATCTATAGTTACCTTTGGAGCTCTAGTTTGAATGAACTCCTGGTGTTGTCCTATAGTAAAATTGATTGGTATTTGCATCTCATATAATCCA
**CoV-bait-1414**	GTTGTGCCTTGCTGGATGGTGGTGGTGAAGATGGTTGTGAGTTTACCTTCAACTGGTTGCATGAGTTCTGCATGGTTGTTTGCTGTTTGCAAATAGAAATGTCCGTCAGTGAGTGGTGAC
**CoV-bait-1415**	TCATAAATGGACTTGGTTAAAGAGTCATAAACAGTATAATGACCACTGTCAACAGGACCTGAAAACGCTGTATAAGCAATACCATTCAACAACCTAGATGGTTGGGCAACCACAGGCCTA
**CoV-bait-1416**	CGGTAAAACCACTGGAATTAATAGGAGTAACAACAATAGGAGAAGTATAATTAAGCACAGCAAGCTGAAATTGGCTAGCGCTATAACTTCTAAAAGTAGACACAGGAGGGACTGCACAAA
**CoV-bait-1417**	ATAATCTACACACTTACCTAAACGGTTAGTGATAGTTAAAGAATCACCTAAGTCCAACATGGGGCAAACACTATCGGTGCCGGTACCATATTGGACAGAGATTATAAAACTCATTTGTAA
**CoV-bait-1418**	TCCTGGTCGCCTATCTGCTAATCCCGAAAATAAAGACGGGTAATTAGGGTATTAATTCATTGTTGCTGTGCCAAAAGGCATCAATGTTTATGCTTCAGACCACTCAAGATCACCAGTTCC
**CoV-bait-1419**	ACTTGATGCACCTGATTTTCACTAGCTTCATCTTGAATAGGTAATGAAGAAACACCACATTGTTGAGCCTGTTCAACAGACACTATGTCATTTGTCACAATAGCCGTGACATCTGACACA
**CoV-bait-1420**	CCAGTAGGGTCAGCCTCAAGATCATCTAAAATTTCCACGTAAGGTGTTTCCTTACGATCCCATGGTCGTTCACGGAAAAGTTGTCCTCCAAGACCATTCTTACGTAGGAGAAAAGTGTAT
**CoV-bait-1421**	GTACACCACGTACCCCTACCACATGCATTACAATCAACATAAGCAGAGTGGTAAATGTAACCTGGTTGTTGAACAGCATCTTTTCCATAAAAAGTATAGAGCAAACGCTGTTTAAGATTA
**CoV-bait-1422**	GTCATTGTCGCGTATAAGCAACTTTCCTTGTGACAAGAGAGAAATTACAAGTTCGTTAATTTGACTCTCCTTTAATTGAAGTACTGGTGTACCTTTTAATTTAAGAGGAAAGCGAGATAA
**CoV-bait-1423**	AAGCTCTGTTTACCGGGGGCAAACTCGTTATATGACACTCGCCCGTTCCACCCATTTGAATTATGAGGCGCCCAAAGTTAGGATAGGTCCACCCGGGCTTCATCGGTTACGGTAATGAGA
**CoV-bait-1424**	TTATAAACCTGAGGAGCTACACCTGTAAGCATAGGTGAAAAATCACACTCAGTAGCATTAGGTTGTTCAATAAAGGTGCCAGTAGCTGAAGCTTCATATGAAGAAACAGAATAGACACCA
**CoV-bait-1425**	TAAAAAGCATGCAACACAGTCCCACAACCATCAGGAATGATGGTTAAACTGTAATTAGCATACAATGGCTGACCAGCCGATGTATTAGTAAGAGACGAACCTAAGATAAAAGCAGGATAA

**CoV-bait-1426**	TCTGTCGCCGCCCTCCCCATACAATCGAAACACCCCGGATATCGACTATCGTGGGGTCAGCGATACAACCCTGGAACAGGCACCCTTCCGTCTTTTGAAACCACTTTAACGAGTTGTATA
**CoV-bait-1427**	TCTTCGCATACTTTCTTTGGTTAAGCTTCAAAGATTGCATATCAGCTTGCGCGCGTATAAGGATCATGAAGACCCAGAGTTACACAGAGATAAGAATTCAACAATTCATCGTAGCTATCA
**CoV-bait-1428**	ATTCTAAAAACGGGGGAGTAGTGTCGGTCTGCAGTGTTAGTCCGTACAGAGATCCGGCGCGTTGGTGTGCTAGAGGTAGGCGATCACTCATGTCATTGCTTCTTGAACTGGCTTATTCCA
**CoV-bait-1429**	TAGCCACAAACAGCTTCTGAGGTGTGGTCCCTGTTGCATGGCCAGCAGAATACACGTACATATCGCCGTGGTCTCCCTGATATGGGAACAGGCCCTGGTAAGTAATTGTGATATTGGAAT
**CoV-bait-1430**	TTATAGCAATGTGTAACAAATACTACAAAATCAGTAAAATTGCAGTGAGCCGTACAAAATTCCTGAGTAGACCATGACATACCATTTTGAGGTGCTGTCATAGCCACGGAAGCAGCACTG
**CoV-bait-1431**	TTGTAATATTGTTATAAACAAGATGAGAAGGACAGACCAACTGAAATTCCAGTTCATCAAATGCCATATAAGATTTCCTTCATTAATACAAGTATCACAAATGCCATTTGTATTTTGACA
**CoV-bait-1432**	ACAGCTCCTGTTTTATTTGAACCATTGTCCACTGCAGCCCATATGGTAACTACTAGTGTAAATGGCTGCAAAAGCCACAGGAGAATTAGTTTAATAATATATATTATACGGGTTCGAGTG
**CoV-bait-1433**	CTACAAAACTAACATGACTACCCTCTAAATCAAAACTCTCAACACTCTCAAGACAAGTGTCTACCAAGACTGTACCGGAAGCAAACCGTTTTTCCTCAGTAAAGACTATAGCATCCTGAG
**CoV-bait-1434**	GATCCGAATGTTCTGAATTTGAGGCTTTTAACTGTTAGTTTAGATCCTGTTGGTATATAGACACTGTAAGGAGAGAAATCAGGGCTTTCGAGTAGAGTACTCAAAGGTTTGGATGATTTA
**CoV-bait-1435**	CAAGGATGCGATCATTATTAAAATCTACTTTAGCCATATTAAATGTAGGCAAAATGCAATACATCAATATCGCAAGGATTACTAAGCAAATAGTAAAACCAGCATACATTTTAAACAAAA
**CoV-bait-1436**	TGAGTGTGCCTTCAACAATCACTTCAGGAAACTTAATAAACATGTTTGAATCATAAAGATCATATCTCACATGTGGTAACATAGTGTCATATGGTACAGCTCCAGGTAAGACATTAGCAT
**CoV-bait-1437**	TTGCACGGCTTATTGTAATTTCACTGATTTTACAGTGTTTGTTACACATTGCTTCGTATCTGGGCATGGTAAATGCCCTTTAACAGGCCTAATCAGCCAGGGTTACATTCGCATTTCTGC
**CoV-bait-1438**	CAGATATGGCTATGCCAGTACCTGTTTTAGGAGTTAACATCTTTACACAATTTGTTAACGGTGTACCACCAGCGTTAACATAATCTGTATAAGCTCTAGCAGGATCCACAGCAAAGTTCA
**CoV-bait-1439**	CACTGGTTTAAAAAGAGAAAGTGTGTAAGCCATAGTTCGTCCTTAATATATAGGCACTGACATGACCACCTTGGGTGTGTCGCCGTTAAATGCACGTGTTGTTACAAGCTCTACAGATGT
**CoV-bait-1440**	CTAGTGGCAGCCATAGACTTGAGCATCTTTTGATGGTATTGGCGATTAGTCATAGTACTAAGAATAGACACTCCAGCAACAGTGCGAGCTCTATTCTTAGCACTTATAGCATACTTTAAA
**CoV-bait-1441**	CTGCATCATCAACTAGAGATTGTTCTTCTACAGCTACATCACCCTCTGCTTCATCACTCAAGGGGAATATAGCATCAACCATTTCAGCCCAAGAATCTGATTCCTCTTCCTCATTGAAAG
**CoV-bait-1442**	ACACAACAGTTTGATCAGACCATTGTAACAACTTACTGTATGAAACACCATTAACTATTATTTCACCTTGCACTAATTGCCAGTGTGCATAACCATTATCCTTGATGCCACTGACAAATT
**CoV-bait-1443**	GCATCATAATAAGTAACACCATCATTATCAAACACAGGGTATGACAATTTAGTGTTAGTGTATGTGCCAGATGCTGCGCTAATAGCATTTTGAACACATGCCTCGAACATGGCACCTTCA
**CoV-bait-1444**	CACCATAATCAGTCTTTTCAAACACATACTCACCTAATTGAACCTTACTATTTTTAGTAGTATGGAATCCTGTGAATACGTAATTACGATTAAGCGGGGGGCGGGTTTTACCTGCTTCCC
**CoV-bait-1445**	TCTAAAAATAATACCACTGCCAGTTTTAGGCATTACACCATACTGTCCAGTTGAAGTACTTATGCACAAACCAGGAACTGCTTCTACAGTAACAAAAGTAACAGGCTGGTATGTGTAATG
**CoV-bait-1446**	TGCTTCAAAGTTAGAGCTGCATAACAGAAGTTTACGAAAGGCATAACGTCATCTTTCTTAAAACCATCCCATAATTCATGGTTGAAAAAGTCAAAAGCTTGCAACTGGTAACAGCAAGCG
**CoV-bait-1447**	GCATAGAGCTGATAATTTTAGACTTGCGATTTTCAGCACGCGCCTCCTTATACATCTGTGCAGCAGCATTCTCAGCCATACGCTCAATTTTCTTTTGAACGGATGCTTCACGATCATATT
**CoV-bait-1448**	ATTTTGGCGTCTATTGGAACGCTGTTGATTTCGTGAAGATGTAAGACCACGTCGTTGATTTAGAATACGTGATCTATTCTGACGGCTGCGGTTCTGATTAGAATTATTACGCCTCTGGAA
**CoV-bait-1449**	GAAGTGGCCTGGCTTAACTGTCTGGTGTGGTATACCAGAGGTTAAAGCACAGACACTAAAACAAGAAGTTCTAAGATCAACTAAATTGTTAGAAGTTCCTACTAAAAGGGCAGGGTCTCC

**CoV-bait-1450**	TAAGTGAAATTAGAGAAGGGCCTATCCGACTCATAATCCCACAAAACAAATCTGTAAGCCGCTGTCACTTCTAAATTACGTAACAGCGTTAGTGGTGGTGTTAGACCTAGCTTCTTCTTA
**CoV-bait-1451**	TCAAGATCTTCACCATGCTTATCAAGTTTGAGTGTTGGACTCGTAAACAAGACAAATGGTTCAGGTCCGGTAAGATCGGTGATGTTACAGTTGTAAAACTCAAAGTTGCCTTCAACCCTA
**CoV-bait-1452**	AAACCTGGTGTATTAGGTACATTCTTTGTGAAGTACGGTGAAAGTTGAGGTGCGAATGGTTCACAGGGTGTGCCATAAACGCTACCATAGCCTTTGTATGATGCCACACATTGTGTGTCA
**CoV-bait-1453**	TCATAATGATCCTGGAACTGGCCATTACGCCTAACGTAGATGAACCAACTCACAGGCTTAACAACACCATCTGCATCTTTTGTAGTGCCAACGGGCAAGCCGTTAAGCATACCAAAGTTA
**CoV-bait-1454**	TTACATCTTGACAGGGGCTTACTTTATAATAATTAACGCTACTAGCGCCTTTAACCATAAATGTATCTATTGCACCTGACGGATCTAAAATTGCTAAACCTGAGTCAGCTAAATAATTAC
**CoV-bait-1455**	TATGGCCAACATACGCACATCTCATGCGGAAAGGGTCGTTACCTAGACATATTGAGGTACACCGACAACGCCGCCCGATTATAGCCTGAAGTTCCGTGAGCTCAGCACCGCATCTATTCG
**CoV-bait-1456**	AAATGCCCCCTTCGAAGACACTGCTGCTTCCTCCGGGAGCTTCGTGCCATGCATTAGATGAGACGTGACAAGAGATTCTTCACCCCGCCCTTCCTCGCGGCGGGGTTAACACGTAACTCG
**CoV-bait-1457**	AAAACACTTTTTCAACACCACAATTAGAATAACCAAATGCATCGAAACCAGAGCCATTGTTCCAATGCATAGATATAAACATTGAGGAGCCCGTCCTTGATGAAAATTTAGGACATAGCT
**CoV-bait-1458**	ACATTTGCATTGACATGTCTAGCACCAACCTCAATACACACACCTAAGTCGTATGGAGTCATAGAATCAACTTTGTTATAAGTAAGTACAAAGTCATTGTAAGAATCTTTTGTGATGTCA
**CoV-bait-1459**	CTGAGATTGCGATTTGACGCACTCATTAACCTTTTGCTGTGCTAGCTTCCTGCTAGCCTGAACCTCAGTATACTTAGTGAGGGTTTGAGCAACAAAAGCATTAAGTGCTGATAATCTGCC
**CoV-bait-1460**	AGCCCCATATCCATGTTAAGAAAATCCTCTTCCATAACACTACGAGGTTTAAAAGTAACACTAGTACGGCCCTGTGTAAAATAACCTTCAACATGGTCTTCAAAAACACCATTCTTCCTA
**CoV-bait-1461**	TAGTTTGCAGCACGCATGGACAAACGTTGATAATCTGGATTTACCAAATCATCGCGTGTGCACAAAACGTGTCTAGGGCAAAGTACGTAGTTGTTAAGCCACACTCCATTCAACGTCATA
**CoV-bait-1462**	TACTCGACTCGATGTAAATTCAATTATTAAATACCTGATGCAGAGCCGCCTACACCAACCCTGAAGGAGCAAACTCAAGTCTAATAGTCGCTCGCATTGAGGAGCGATAGTTCCGTCGAC
**CoV-bait-1463**	GCGTCAACAACACCAGGTAGTACCATGACACCAGAGTAATACTGTGCACAGACTAGATCTGCCACAGCGCGACCATTAGAACAGCGCTTATAGTCTTCATCAACAGTACCAAGGCCATTA
**CoV-bait-1464**	GGTGCAAAAGCACACAATGTCAGCAAACCACTATTAGATGGGTGTTCAGTAGGTTTACCAGCTTGCAGACGAACCGTTGCACCGATATAACCAAGAACAGCACCACGTCTAAGAGTGTTC
**CoV-bait-1465**	CACCTGGTAACACCATAATACCATTGTAATACTGAGCACAAAAGACATCTGCCAGTGCAGCACCAAATCCTTCACCTGTGCAAGCTTTGTAATCCTCATCCACAGTTCCAAGACCACTTG
**CoV-bait-1466**	ATGCTGCTTAGAGCACCTTTCCTATCAAGGCACTGGCAGGAAGAGTCTGGAGGACTGCGATCTAGGTTTATGTTGGGCCACGATAACCCATGAAGTCAAGAGCATCCCGCATCCTGCAGT
**CoV-bait-1467**	TACAACATTGTAATCGCCAGTTATCCAAGCTTGCTTAATCACATCTAACGTTAAATACTCCTCTAATTGAGGCCCTTTACCAGGTTTAACAGTAGTGTTTCCTACTTTAAAGGGTTTAAA
**CoV-bait-1468**	CATACCAGTTGGGCTGATCAAAATAATCCCACTCACAGCAACCCTTAACAACAAGGATCTCTTTGAGCACATCACAATCATTAGGATCGAAATGCCTAAAAGCATAACACAAATCCATCA
**CoV-bait-1469**	TATCTAGGTTCTAGAGTTCCCTTATTCAGTAGCACACGAGGTGCAGGCAACTGAGCAGGATCTCCAATATACACATAATGCTTAGCCCTAACACGACTGTTAATAACAGACAGCTCATAG
**CoV-bait-1470**	GTGCAAGATGTTATCACATTACGGTCGAGATATCATTCTATGCAACGAGCCTAACCAACGTGGCTGCCTTCGGATGTCACCGTGTGTCTTAGAACTTGGTCAAATATATATTAGTTTCAT
**CoV-bait-1471**	TCTTCTTAACATCAATAATAACATCCCTTATCATAGATAAAGGCACAGACATAACACGTTTTAACTCATATGAAAACCTAACACAAGACATATTATAACTACGCAAATCTTGCCAACCAT
**CoV-bait-1472**	AACAGACTACGTGCACTAAGCGGTAAATCTGTCACATTCCGCCAGAACAAATAATTTGCATGCATCACCGATCCATCAATAATCGGTTTTTCTACATCTCCTAAATAATTAACACCTACC
**CoV-bait-1473**	CAATGCCTTCACGTCCAATGTTGAATCAATAGACAACAAAGTCATGAGCATACCAGTTAATTTTTCAATGCAAATAGCGGGATCACTCTCTGCTAAAATATCATTATGTGTTTGTACCAA

**CoV-bait-1474**	AACAGGAATGACCTCCTGTTCATGAATTACATTAGTACTAACTACCGGATCTTCCTCCGGCACTGGTACACTACTGGAATCAGCTACGTCATTCAAGGACTTCTCCTGTGCATCGACGGC
**CoV-bait-1475**	TGCTACAATGCTAAACCTAAATTCAAAATCTGCTATCAGAGCCCCAACTGGAAAGCCATCGATAGCCAGCTGTCTCTGCTTAAAATCTATTATAAATGCCTTTACCATTGGCAAAGGTAC
**CoV-bait-1476**	TATATTAGAAAGAAAATTACCCATGACTAAAACCAAAAGTGGTCAAAGGTTCTGGGTTTCCAGTATCAATAAAAGTCGTTATTGGTTGAGTAACACTTATCATATTAACACCCAACAACT
**CoV-bait-1477**	AGGATCACTACCATCAAAGTCAAAGGATTTTGGCAAGAATTTCTGAAAGCCATAGCCTTCAAATTGACCAAAACTTGCATTAAGGTTCTGCAAACTCACAGAAATATTATCAAAACTATT
**CoV-bait-1478**	CTCATTAATAGAAGACGAAATAGCACCAAATGAATTTCCCAACTGCGAAACTAATGTCTGCAGCTGCTGTGCATGTTGATTGATAACAGACTGCATCTTACTAAGTGCCTCATTCGTAGC
**CoV-bait-1479**	TATTAACAGGTAAAGGATCTACAGCCAATACGTCATAAGTATTAGGCGTATTAACAACAACTGTAGATAATAACGGCACTAATTTCTTCCAAGTATTAGGGCGCGTAACAAACAATACCG
**CoV-bait-1480**	ATGCCAACTGAACACAACTATTAATAGGTGCACACACACTGCCACAAAAATGTGCCAGGCGCTCCTCCTCAGCTCGGACAAAGGTCCTAAGTGCTTCAAAGGTGGTACTGGCCAGTGTTC
**CoV-bait-1481**	TTGATTAAAGCACCCCCTTGACAAATATCAGGAATGTCAACAGGTGTAACACCGAAGCCTTTAGACAGAACAGTTAGGACAACATAACGAGCGCCCCAACCTATCTGTGGTAAAGAGCCA
**CoV-bait-1482**	GCCATATGCAAACCAGCACCTACAGGTATAAAAGCCCTGCCGCAAAAAGCACGCAGCCTTTCCTCTTCCCTGCACACATAATCCCGCATGGCAGCAAAGGTCTTGGAGTTTAGGGCAGCA
**CoV-bait-1483**	GTCTTTAACATACTCTAATCAACTAGCACTAAACTACATCTTAAACCTAAACATAGCGAATGCACAGCTTACTTATACCACTGTACACTAGGATTTCATTCTGTATAAGAGTGGACTGAA
**CoV-bait-1484**	AGGACTACACCATTGCTGCACACTTCTGGTACAGTCAACTTGTTTTGTCCAAAACCATAGCCTAGTATGGACAGTGTCACTTGACGTGAGCCCCAGCCACCTAGGTTAAAACTGCCATTG
**CoV-bait-1485**	TTCTTCGTCACGAGTGCTAAAGTGTGACATGAACATGAATGCAGCTACATTAGGGGCCATTTGCGCAACGGCAGGAAACCTAGGGTCGTCTGCACCTAGTTTCAGCATTTCTAGATCCCC
**CoV-bait-1486**	CCATTGGGTAGTTCCGCAAGGAACTTAGCAATGGCACCATTAATACTTGCAAGACAAGTTCCAGAAAATTCGCGAACAACAATAGCTTTTTCCACAACAACAACAGTAATTGTTTTAGCG
**CoV-bait-1487**	GATTGGTAGACAACTTAAGCTTGGTAATAGATGGCATGCAATCACATAGCAGTACTAATACATAGACCACAATAACATACCAGGGTGCAACAAAAACATAAGCTACCACATACATAAAGT
**CoV-bait-1488**	ACAAGTGGACTTATAACAACTCCAATCTCCAACAGTCCAGGAAACTTTTCCACAATGGCATTTCACCAGTCCATGATTGAACACAGGTTTGGGTATAATTTCAGTTAATACTTTACCATT
**CoV-bait-1489**	CAAATCCCTGGCCACCGGTCCCATGAATCGCTACGGCTATCGGACAGGACTGGAGCGGGTGGGCGCCGGGAAGAGCTAGGTCGGACCGCAAAACCCGGATGGGATGGCTTTCGTTCCATC
**CoV-bait-1490**	TACGCTGTTGGCGAGGTGTAAGCATGGCACCAAGTGTCTTAGCACAAACTTGTACTTCTGTTAGTACGCCCTTTGCTGAGAAGAAGTGGTAATCACCATTGTAAAAACCATCTATCTTAC
**CoV-bait-1491**	ACCTGCATCGGCCGCACAATCAGCCACCTTAAGTGGCAAGCCACTATCACTGTAAGACACTAAAACTTCACGTAAACTCAAAGGTGCTATATACAAATTATTCTGAAAAGTATGACATCG
**CoV-bait-1492**	TTCAACGCCCAGTCGTCAGGCATATACTTCTTCAGGTACGGTACACCCTAAGCGGAATCTCCAAGCCGTTATGCTTGACAAGTTTCAGAACCTCGTGAAGCAAAATCCAACCATTTCTTG
**CoV-bait-1493**	GAGGTCATATCTGACGCAAACGCCAGTGGCCACTTCAGTATTAGGCAATTGTAAATCTGTGGAACACGTAGTCCCACTATTAATGCCATTTAACAATATGTTAGCAAAAATTGGGCAGCG
**CoV-bait-1494**	TTGGTAACCACAGCACGACCTTTGACAGACTTGACACGAGCACGTACTTTACACCTGTGTGGGCAATGACCGGCGTCACAACCGTCACGCAACACGCTAGCGCAAACAACAGCACCACTA
**CoV-bait-1495**	CTCCCATGGCTCAAGTCAGAGCTGCAAGATTCAACTTCTGCAACAACATCATCAACTTTCAAAACATCAAACTTATTAACATCAACCAAACTAGGTCGATTAAAATATGTAAGAGAATTA
**CoV-bait-1496**	GATTAATGGGTCGTCTCATGACCTGGCGTAAAAAGGTATATTCTCAGCGCGTACCCTCAAATCTGGTAGATGACTGCATTTGCACTCCTACAAAGTCACACACGTAGTCATTACGCTGTA

**CoV-bait-1497**	CATATAGGCTGGAATAATTACAATATGTAGTGCAAACGCCTTGAGCTGACCAAGCAATAGGTGTTTTAGTAACAAAAGATATAGCACTCTCATTAATGACAATGCCGCCTTTTATTGTAC
**CoV-bait-1498**	TCCTACTCGGCGTGGCCTCGGTGTGTATTCTTTGTAATTACTCCAATCAACTATACACTAAATGTCAAAGACAGTGATAGTAAACGTGTATAGTGTCTTCTTGTGCTACACTATACATAG
**CoV-bait-1499**	ACATGAAAGTTTTACCACTTTCTGTATTATAAAGAGCTTTACCCTCAGCAGACAAACCATTATCGCCCTCAGCACGCGCAACTTTTTGCTTAAGCTTACCTGGCATAATCTCATTATTTT
**CoV-bait-1500**	CAAACCAAACTTCACCATTGTCTTCCTCCACTTTGTGAGCTCCACCAGCCCAAAAAAGTAGATCACCGGCCCAAATTCTACCTATGGCACACTTTTCCAAGGTTTTATAAAAAGCATGGA
**CoV-bait-1501**	ACACATCAGGACCTACTGCACACATGCGCTGTGTGACTACATTGTAGTCCTTAGGCTCAAGTACACCTCGTGTTATCATAGTTCTAGGAGCAGGCAGTTGCTGTGGATCACCAACATATA
**CoV-bait-1502**	AATGTTTGTCTGCATTACCATCCTTTACTAAAGCTGAGTTGCCAAAATTTGCCTCGGGTCTACGTTTTCCAAACATAGACTCAACCTCCCCCTTACCAGGTGTTTTCTTCCATGAGTGCT
**CoV-bait-1503**	TACCGGCATATGGCTATTCGACATCGAGAACATTACCCACATGATAAGAGATTGTATCAGTTTCGTAGTCTTGAGTATTGGTATTACTATATAGTATATGTCTAGAAGCGATGCTACGCT
**CoV-bait-1504**	ATACTCGAACCTGACAAGGATTTACCACAGCCGCCATTAATACCTGCGGTGATTATAAACTGATATACAATTAGTGAAGAGTAAAAAATCTCATAAACAAATCCATAAGTTCGTTTATGT
**CoV-bait-1505**	CAACACTTCAACACCATTAGCACGGTATTGATCCAAAGCATAAGCCAACCAGGCACGACAAGCATGCAAATAGTCTTGCTCACCGCCAGTGCCTGAATAATACTTTAGTCGTGCATAGGC
**CoV-bait-1506**	TCACTATATGGCTGGCGCCCGCCAGGAGTTATTGGGGTGGCCGGCCAGCGCTGAGCTATAGCATAATCTTTATTAGTATCATTAATATAATACGCATGTACACAACCATAAAAATTAGAA
**CoV-bait-1507**	GCCAAGTTGTTCTGATTCTTGTATGGCAATGGTTTACGTTTAGTAAGCCATGCACAAACATAAGTGAGACCATTAATAACAATTTTTTCGTTGTCACCAAAATGGTCAATAAATTGCCAC
**CoV-bait-1508**	AGGTGGAGAAACTCGATGGTAATTCCTTAAAGTATAGTTAGATTCATTGTCACCCTTACAAACAATATAATTATCATAGGAACTACGCACAGGGCACCCAGAAGGATGGCAAGGTGTAAA
**CoV-bait-1509**	TTGCATTATTGAAAGCATTAGCAAGTATTTTTTGGTTTTCTTGTAATACATCAGTTTGTAGTGCAACATAATTCAACCTTGCTTGCACTTGCATTGCAAATGGGATTGCAACTGCTGATG
**CoV-bait-1510**	ACTTTGTCTTTAGCCAATTGCACACCACGACGTGCAGTTTCGGCTCTGACAAGTTGTTGAGACACAAAGGCATTAAGCGTGGTTAGACGACCATTGATCAGGCGATCTATCTGTGAATCC
**CoV-bait-1511**	GGAATTAAACCTGTTAAAGGACAAAAATCAACTCCTTTCTTAAAACAATGTGTAACAAAGACTACAATGTCTGAAAAATTGCAGTGAGCCGTACAAAATTGCGAACTTGACCATTCCATA
**CoV-bait-1512**	GATTTAGAATTGCCAACACCAACAGCAACACCTGTTCCAAACTTATAATAACTAATGAAAACCGCTCTAGCATTATAAGTGATGTTGGCTGTACCTGCAGAATTTTGTGTGCAATTCCAT
**CoV-bait-1513**	TTGCATCTTCTGGAACTTCTGTGTATGGGCAATCTGGTTTGATGGTACCTGGTTTGAGTTTATAGGCTGTTTCAATGCTTGTGGCGAGTTCATATCCTAATGTGTGGGTGTGACTATCTG
**CoV-bait-1514**	CAGAAAAATTACAGTGCGCCGTACAAAATTGACTGGTAGACCATGACATGCCTTGACCAGGTGCTGTCATAGCTACAGATGAAGCTGTAAAATTACGACTATAACTAATAATGCCAGCTG
**CoV-bait-1515**	GGACATTCTCTTAAATCCGGACATCCTGGACAACTCGATCATGGGGTCTCGACAGATTAAGCCACTGCCACAAAGTCAGCAGGCCGGAACTGGCCGCACTCGGTCGTGTCCTGGTGGTAT
**CoV-bait-1516**	CAGATTGAAGATGTTGGATGGACAACGGTAGCAGATGGAAAGCACCCGACTGTCATTACAGAATTGGAAGAGTGGGAAATTTGTGTATGAGTATGACAAACTGCGCATGTGTGTGACTGG
**CoV-bait-1517**	AATTGCAGTGAGCAGTACAAAATTCGCTTGTCGACCACGACATGCCAATACCGGGTGCTGTCATGGCTATAGAAGCTGCACTAAAATTCCGACTCCAAACAATAGCGCCTGCTTTACATT
**CoV-bait-1518**	CACACCAGTCCACAATATGCTTGTACTGGCACTTGCAGCCCAGCTTGCGGTTCCAATTGGTGATGCGCAGGTTGTTATCCACCATGGTGTCACAGTGGGGGCTGTTCTCCAGGACCGTGT

**CoV-bait-1519**	AGGTAAAGTATTCTGCAGGGAGAGGGCCGCGGGGTAGCACGAGATAGAGCAATATATGCCAGTCTATAATACGGTTTGGCAAATTGATGCGAGGCCGACGTTGACGTCACGCTTTTGCAG
**CoV-bait-1520**	GTAGAAGTGGACTTATAACTAACAGCATCAGAACCATAATCAACTTTTTCAAAAACAAATTCACCCACTTGAAATTTGGAGTCTTTTGTAATATGAAAACATGTAAAAACACTGTTTCTG
**CoV-bait-1521**	GAGAACTCTGGGTACATGTCTACATTGCAGTTCCAAAATAACATAAGACCATTCATTTGACCATGTACCATATAGTCATACTCCAGACATCTAACATTGTTATTAATAGGATCTCTGTCA
**CoV-bait-1522**	TAGCAGCTTGTTCAGCCATTCTATTAATTTTCTTCTGAACAGATATCTCATGATCAAATTCAGACTTTGCGATATTCATGGCACGCTTCAATTGTTTAATAAGTTGAGAAGAAGATCCAT
**CoV-bait-1523**	TCCTCAACTGATGGCAAGTATCCTAACCACACTCCAGAATATTTACTAGGATAGTGACCATCTGCCACAATACATGGTGCTTCAGGAAAAAATTGTATGGAAACATGTGAACCAAACTGA
**CoV-bait-1524**	TAACATCACGACATATTGGGAGCGACAGCGCGGCGGGTTGAAGCTGCCGCAGCGGTGTACCTCCTCGAGGAGACGAACCTCTTGTGCGCGATCGCGCAGTTTAACTGTATCGAAAGATGC
**CoV-bait-1525**	GCATAAACATATCACGTGCTGTAATATAATACGTGCCGTTAACTTGAATAAAAATACCTCTACCATTAGCAGGTACTATTGCATACTGACTAGCATTAGCAGGATTTATGCAAAAACCTA
**CoV-bait-1526**	AGTTGTAAGTTCCAAGCCACCAGCCCAAAGTACAAAGATGATTACGTCTGAAGAAGATGAGATGTAATCACAACACATCTGTACAATACGCTTACGGACAACATTCCATGGTTGTCCTTT
**CoV-bait-1527**	TAGATATAAATATAATAGTATAAGGTCCGAACTTTACATGATTTCTACCACCATGTAAAACGAACGGTTTTTGTTTGTAGTAATACCATGACTTGCCATCTATGGTAGCCAAAGTGCAGG
**CoV-bait-1528**	TCACCAAACATGCGTTTAAATTTAACAACTAAAAAGCAAATAAATATTGCCATAGCTGCAACAACACAATTAAAGATGACCTGTCCCGAAAGTGCCATAACAGACAAAGACATTGTAAAA
**CoV-bait-1529**	AACAACATTATAAGCGACATTTTCAAGACTCTGTAATTTTGGGGTGACCAATGTTTGCCACAAATTATAAGTATCGAAACTTTTTGGTACCCAAATAGTAAATCCAGCCTGGACATATGT
**CoV-bait-1530**	AGATCAGAAGAGACGTCAATAACCTCTTCAGGTTTTACTTCTTCTTCTGCTTGTGGTTCATTAGCATCATTCACTTCTTCTTCAGATGTAATTACAGTAGGTTCAGCATCAGTAACTTCT
**CoV-bait-1531**	ATCACCAAAATCATAGAAATTGCCATTTAGATCTTGGTTGTCCAGCGTGATAACCCCCACAATGCCTTTCTCAGTCATAGCATCACAAAGTGACACACACTTGAGCATAGCATTAGCTAC
**CoV-bait-1532**	AAAGCCTTCTGATGATGACGTGTTAACACTTGTACAAAACACAGTCCAATACTCAAATCTTTGAATCAATTCATATAAATCTTTATTCCAACTAAATTCCGTGATTTTAATGGCAACAGA
**CoV-bait-1533**	TGTAAATCTTGCCCCATACTCAATCCGAGTGAATGAGCTCAAATCTTCAATACGCCATGCACGCTGCGTTAGCTTCGGGAGTCGTTTGAAGGCACACATGGAAGGAAGACAGCTGCTGAG
**CoV-bait-1534**	AACTGAGACACGGGCCATAGGAACGCGGATATTAAGTGGGCGTTTATCTAGTGGTGGGGCCGGATTAAATTGAGTTGGTTTGAGTGCGGTGGTGGTCACTCTAAATTTGGTGGCTTCTTC
**CoV-bait-1535**	AACGGGAATAGAACCGTTAGACATATTTCGTTTATACGTCAATAACAGTACTGGGGAGGGGGTCTATTTGCATGTAAGACTTATAAACTCTATACAAACGCCCTATAGGTGTGTAAACTG
**CoV-bait-1536**	CTAGATGGTACAATGGTATGTTATTAACGAAACACTCTGGTACCATCACATTGGGATGTAAGTTAACAAACCTAGAAAAGGTTTGCTGAGGACACAATGTGGGCGCAGTAAGCGAGACCA
**CoV-bait-1537**	CTAACGAATGACCCGCCATACGTAAATGACCACGTATTATTACCGCACCTATTACTAATTCCGATTCCATTAACGGACGCGTTACTATCGTACCACGTAACGGTACATTTAATAATATAT
**CoV-bait-1538**	AACTATGCACTTCTCAACAACACCAGATGGTTGTGCCATCTTACGCAAGCCAGCCTGTAGAGTTGAATTGTAACTCACAGTGGGTGGTGTGTATAACGTGTCGTTGTGATTAGAAGCATA
**CoV-bait-1539**	CAAAGGGGGTGGTCTCAATAAGTCGTCCATCCTGGTCAGTGTCCTGACGTATATGCGGGTGGCGTACCAGTATACGAATCTATAACGTTGAATGAGATTGTGCGTATATCCTGACGGACG
**CoV-bait-1540**	ATTATCCGCCCATTGTTACTATTATTATAATAACACTGGGTAGGCACATTACAATTCATGTCTGGTCCACTACTAACAACCTGTGTTTTTAATTTTGCAGGCATAAGTTCGTTGTTCTGC

**CoV-bait-1541**	CCTGGCATGCGTAATTTTACTACACCGGGTAAAAGCCGCCCTGACAGCCACCATTCATGTTGGACACGGCATACCAACTCCAGGTTGAGCCTCTGGCACTCTACGGAATTGGCGGGACCG
**CoV-bait-1542**	AACTGGAGGAGAGCTCCTACCATCTTAACTGATTGGACATTTAGCTGAATACCCTGTATTGGACACTTGACTATAAAGTCGCGGCAATCAGCAATTGAGACCATGTGAGTCCACTGGTCA
**CoV-bait-1543**	GTATAACAAGTACTATGTTAACAAAACATAATATTATAGCCAAAGCATAAGGCATATAATTTGTAGTGTTAACAACCACTTGATTAATTTTCAAGTGGTCATACAAGTGTAAAGAATCTG
**CoV-bait-1544**	GAGTTTGTAGGTAGATGTTCCTCTGTAAGTATAAGTGTCACTGCCATCTGTTTTAGCAAGAATGTAATCACCAACTTGGGTTTTTCCATTCTTGTTAAAATGGTAGCCAGTGAAAATGTG
**CoV-bait-1545**	CAGGTCACAGTCTTAAGATTGTCTTTAGTTGATGTAATGGCAACCAAAATCTTCTTACCGGTTCTAGTAACATAAAAAGATTTTACAGTGGATTCATTGCCATTGAAGTCCTGAGCTGTG
**CoV-bait-1546**	CGGATTGTGTACCAATGAACTCTGTGAATTGGTTACTCATAGCCCATTCATTAAAAGTAGCGATTCCAGTCTTATTTGGCTTAACAAACCAATTACAACCGTTAAGAATCGCCGCATAAA
**CoV-bait-1547**	CTGGTTAACGACCGTATTGAGCGCATCCTTAAGTCTCTCATAGTCAGGAGAACCCTCCTTGACTGTGATTGAGTAAGAGAAGGTAAGGGTAATTGCCCCTGCCTGAAAGTTGCTCTCAAG
**CoV-bait-1548**	AAGGTACTTATCTGCCACCTCAAGGCAAAATAAGAACATCTTGATATCTACCATCGTGGGGGTATTATACCTGTAGTAGCTGTAGTCTGTGATAGCCGCTTCACCATCCTGCATATAGTA
**CoV-bait-1549**	GAAACACGGTTACGGTGTAATCACTAGTCAATGTGGTAAGAAACATAAGGTAGGTAAGGGGACTAGAGCAATCACCCGTAGTATAAGTGTAGAACCAAACCATGGCACTACCTAAGGCAA
**CoV-bait-1550**	AGTTAACATCTTAACACAATTAGTCAATGGAGCGCCACCTGCGTTCACAAAGTCTAAGTATGCTTTACCAGGATCAACGCTGAAGGTGACAGCCGACAGCACTGACGAGTTGATAGCAAA
**CoV-bait-1551**	GCCATGTCAAGGATTCATTTGTCTCTGTGACATCTGAAGACTTAACAACTTCACCGTCCACATTGTTTATAAGAGAAATGTCCCAAAGTGCACCGGCATAAGACAAGCTTGGCCATGTAA
**CoV-bait-1552**	AATAAGAATAGTAGCTATCAACAATAGCTACTCCAGCACCAGGTTGAGTGATGACAAAATCACCAAAATCATACCACTTGCCATTCAGATCCTGATTGTCAAGGGTTAAGACACCAACAA
**CoV-bait-1553**	CATAGTGATTTCACGTGAGTCAAGCTGTGCTGACGAGTGAAGTGCACTCTCAATGTTTGAGCATGCTGAGGTATACTGCGCTAGCAATTGTAAGCAGCGAGGGTTACCATTACAGACATA
**CoV-bait-1554**	GGCACTTCCACTTCTTCAGTAGAGGCTTGTTCAGCGACATCATCAGCTGGCTCTGAAACATCAATTTCAGCTACAAGGGTATCTAATTGGACGTCTTCCATTGGAATGACGGCTTCAACA
**CoV-bait-1555**	GATCATTGCTGTCGGTACTTGATAGGGACTATGTGGTGAAATTGTGCTCATGGCGGAGGGGCATTTGCCTAATGAGAAGGAACACAGAATGCAGCTTACGTGTGCGTGCTTTGGACGCGG
**CoV-bait-1556**	ATCACTTAGCAAACTAAGTCTTAATCTATCACTACCTACACAATTTTTACACTGATATATACAAATACTATCATTCAAAACTATAAACAAATCCCTTATATCATCATTTCCACATGGACA
**CoV-bait-1557**	GCATAGTTAGGGTAAAGAGTTGATGCTGGGTCGATAAAGATGCATCCGAGATACCAGATTTTCCCCTAAGTATAAAAGCGGCACGCCATAGACGGGTAGTGTCCAGCACCTGTCGGACTG
**CoV-bait-1558**	ACCAAATCAACACCAACATCACAATATGGCTTTGAAAATGCACTTAAAATTTGGGGTGGACAGCGATAACATGCAGTAAGATAACGTTTTTGAGTTGCTAATTGTAACAAATAAAAAGTA
**CoV-bait-1559**	ATTTCTCAACGCGCTTAACTTTAACACGCTTACTAGGTCCATAAACTGTGCTAACATAAGTAGCAGTTTTCAAACCATCTTGCTTGAAGCCCTTAAGTTTCACACTCGCAAGTTGGCACA
**CoV-bait-1560**	GCGACGAGGACCGATAAAGTGTAAGTGATTGAGAGACGCTTTAGCCCTCCTTCGTCGCAAGAAGACGATAACTGAAGTGACATCAGATACTCGGCGGGCCTTCGCTGTCAGGATGGATGT
**CoV-bait-1561**	AACTATAAAAATTATTTGACCCACATACCACACAGTGTCTGCAAGATAAGCATCAGCCATAAACATTTAACTCCTGTCTCCGCCAGGTAATAAAGTCAACAGCTAAAAAAACCAGTTTGT

**CoV-bait-1562**	TTTCATGATAGTCTCAAAACCAGGTAGAGTACTATCAAATCTAACTGCACCTGAATATTGCAGCTCATACACATCTTTGGTGGGTTCATCAGCACCACCAGAATTCTTTTTGACCAATTC
**CoV-bait-1563**	TTTGATGATATTGGCGAGTGGTCATCGTACTGAGAAGTGAAACGCCACCCACAGTTCGAGCGCGTGCTTTACCACTAATAGCATACTTAAGATTAAGCTGCGTCATAGTGGGCAACACGT
**CoV-bait-1564**	AAAGGGTCGAGTCCCGCCCCGCACGTTCCGACTGCCTAACCATTGCAGCGCGCGTCTTTAGTCATCTGGCTGTCACTGAGGGAAAGAGAGCTGCGACGCTGACCGTCTTCCTTGTAGGCG
**CoV-bait-1565**	CATTTCATAATGTATTGGAACTCTGTGTCCATATTCACCAGCACAAAGTTGTACTAGGCGAGCAGACTTACGAGGTCCAATTTTTAAAAACCAGTCCGAATAGTAATGTATTGGACATGG
**CoV-bait-1566**	GCCGTTTTAGAGCAACACTGATAATGCCAATTATCCAAAGTCTTAGATCGACCTGTTGATGTTAGCTCTCTGGCACGCATCACTAGAGATGTAAAAGATGCTCTACACCATGATGGTACA
**CoV-bait-1567**	TTCAGTGTACTGTCAGAATTAGTCATAAATTCTTGAACAGAAAACAAACCCATTTTTGCAAGGCGCACTTGCGATATAAGAAGATGCATACCACCAATGGTAGTTTTGGAGACATCTCCA
**CoV-bait-1568**	TTTGGCTTCCTTAAAAATTACAGTTCAAAAAGTATGAGAAAATCCATGCAGGCTGAAGGAAACAGCAAAACTGTGACAAATTACCCTCAGTAGGTCAGAACAAATGTGACGAACCACCCT
**CoV-bait-1569**	ACAGTCCTTAAAAAGTCCAGTTACATTTTCTGCTTGTAATGTAGCCACATTGCGACGTGGTATTTCTAGACTTGTAAATTGCAGTTTGTCATAAAGATCCCTATCAGACATTATGCACAA
**CoV-bait-1570**	ATCTACCACTACATTAGTAACACCATCTATGTATTTAATGATCGCGCTTTTTTCGGCTGAAGTATAGCAAAAACACTTTACTACTCGTTCGCCCGCAGACGCAAAAAGAGCAGACAGCGA
**CoV-bait-1571**	CAACTCTTTTTGATTGTCCAAGAACACACTCAGACATTTTAGTAGCAGCAAGATTAGCAGAAGCCCTGATTCCAGCAGCCCTGATTAGTTGTTGTGTTACATAGGTTTGAAGGCTTTGAA
**CoV-bait-1572**	TGGGCTTAAAATCCTCTAAGTCTCTGCTCTGAGTAAAGTAGGTTTCAGGCAACTGTTGAATAATGCCGTCTACTTTCTTAAAGTAGTTAAACTGTGTTTTTACTGATTCTCCAATTAATG
**CoV-bait-1573**	TATGGTAGGATTTTCCACTACTTCTTCAGAGGTGGGTTGTTGACTTTCACAAGCAAGATTGTCCATTCCTTGTGTGTCTTCTACTGCCAGAACTTCAAATGAATTTGAAGTATCTACTGG
**CoV-bait-1574**	AACTACAGCCATAACCTTTCCACATTCCGCAGACGGTACAGACTGTGTTTCTGAGTGTAAAACCCACTGGGTCATTAGCACAAGTGGTAGGTATTTGGACGTACTTACCTTTCAAGTCAC
**CoV-bait-1575**	ATCCTCTAGGTAAACTGTAGCGCAATCACCAGTAATGCTGAAGTCAGCGTCGCTAACATACTCATTGATGTCATTATCAATGATAATGGCATCTGGAGGAAGCCAACGCTTAAGAACAGC
**CoV-bait-1576**	TCATTACCAGTGTAGTATGACCACCAAATACCTTTAAGATTACTAAAGTGATCAACACCGTATTGATTTAAAGGTTTAACAGGACATATCCAATTATTACTACTAACTGGTTCAGGCAAA
**CoV-bait-1577**	GGGTCTTGACCATAATTCTTAAGGTCATAATGGGATTCCAGTTCTGGTAAAATGGTTGGCCATTCATAATACGACCTTATAAACTCTTTTTGAGGATAAAAGGTCTGTATATCGTCCTCG
**CoV-bait-1578**	TTTTGAGAACAATCAACAGCATCTGTGATTGTACCATTTTCATCATACTTGAGCATAAATGTAGTTGGCTTTAAATAGCCAACAAAATAGGCTGCAGCTGACGTGCCCCAAATGTCTTGA
**CoV-bait-1579**	CGGTAACACTAATGCTACTGCGCGACTGCGCATTTGCCAGTTTCCTAGCATTAAAACATTGGGCCCCACTGCTAATGATGTTACAACAGGTCGTAATTGCCTATTTAACAAAGCCATCCC
**CoV-bait-1580**	CATTCATCAACTAAAGTGCGGCCCATTTAATAGGTCGTTGGATATTTCGTATAGCTCGGATGTCACTCGTCCTTTAGTGCTGGGCAGCAACAGAGGACCTGGCCAGCCTACTCTTTACCG
**CoV-bait-1581**	TCACATGTATAGTGCGTCTCTCATCGGTGGTATGGTGCTAGGAGGTTTTACTTCTGCAGCGGCATTGCCTTTTAGCTATGCTGTTCAAGCTAGACTCAATTATCTTGCTCTACAGACGGA
**CoV-bait-1582**	AGAATGCAGCGACAATCTATCTTCACATGATTATCATAAACAAATCCAACACTTTCCATCGCTTGACCAGTAGAGGGGCATAACCCACCCTCATCTGACTTCATAGGACTAACCAACTCC
**CoV-bait-1583**	TCATAACTTTCAAATTGTCTTCTACTACACATAGAGTGACAACAACTTCCAATGCATCCAAAGCATCCACAGCAACCAGTACTACAACAACAAAATAGCAAAAGTGGTATGCAAAATATT
**CoV-bait-1584**	TTATCGCACGTAATACCGTACCCGGTAAACCCGGTACTATAAAACCTATTTCACGCGTTATTATCGCCGCTACTACCGGACACGATTTATCATTTTTATACGAACCACCACGTTGCGAAA
**CoV-bait-1585**	ACCCAAAGCCTTTGCATTATAGCGCACACTTTTACGCAATATAATACCAGTACGTTGGGGTACACCAGCTAGAACTTTAACATCCCTCTTACGAAAGGCCCTAAAACAGAGACCCAAAAT

**CoV-bait-1586**	ACACACTTAATGGCAACATTATCAGTAAGTTTTAAGTAGCCAGTAAACTGATTAACTGGTTCTTCAGGCAGAGGTTCTGGTTCAGGTTTTGCTGCAATAGCTTCCTCAAGCCAGTCTTCC
**CoV-bait-1587**	AAAATTACAACAAATGCGACGATGGCGAGGGTGATTAGTAACCAGACCCACCACGGCCACTTAATATAAGTTTCCACTCTGTTGAGCCAATCGAGGTCAACTAGTGTGGCATTTAATTTA
**CoV-bait-1588**	CAGCTGTTCAGATAAAGACATGTAGTCTTTTACATTCCAGATGAGTGAAACATTGTGACTTTTTGCTACTTGGGCATTGATATGCCTTGCATTACAGTCAATACATGCGCCAAGATCTCT
**CoV-bait-1589**	CAGGCCTTAATTTTTGGCTTCACATCGACAGGCTTCTGTACGACAGATTTCTCCTCAGTTTTGGAATCTTCTGTGTTTGGTGGCTCCTCTTGTTTAGGTGCTTCCACTCTAGGCTTCAGG
**CoV-bait-1590**	CTTGACCACTTTTGAAATCACTGACAAATCTTGTGACTTTATTATCTCGACAAAGTCATCAAGTAAAAGATCAATCACAGAACGCACACATTTTGATGAACCTGTTTGCGCATCTGTTAT
**CoV-bait-1591**	TAATCAATGTCAACTTAGGCGCCTGATGCGGTATTTTCTCCTTACGCATCTGTGCGGTATTTCACACCGCATATGGTGCACTCTCAGTACAATCTGCTCTGATGCCGCATAGTTAAGCCA
**CoV-bait-1592**	CATAGCCAATAGATTTCGCAGGCCAGTCGGTCCGGGAGTGTATTGGTAGTCTCCGAACGGCTAGCAGCGTTATTACATCACGTCGACTGATTATCTCGAATAAGGTTTCCCTGGGGCCAC
**CoV-bait-1593**	TAACACCTTACTTCGAATTCCGGTGACAGCAAGGTTCCCAATGACTTCGTCGCAGAGGGCGCCTTAGACAATCCTTATCCTGTTCCTAAGAGTAATAGACGCCTCGATTTGTCATTGCGG
**CoV-bait-1594**	AACTCTCTGGATGACACTTTATAATCATATTTACCAAAAGTAAACCCTGTAAATTTATTAATCCAATATAATACACCAAAACGCATAGTTACAATAAAACCAACACCTACATAAGATAAT
**CoV-bait-1595**	TGCTGTAATGCAACACGGCCTCACCGATGTCACCAAAACCAATAGCAGAAATTTCACAAACAGCGGCTTTAGCCACAGTAATAGGGTTGCGAGCCATGAGTTGCAGTTATAGGTTTCAAC
**CoV-bait-1596**	ACCACAGACATCCTTAGTGTATGAAGTAAAAGGTCGTTCAGCCTCATAGTCCCACAAAACAAATTTGTAAGTACGTGTAACACCCAAATTCCTCAAAATAGTGAGGGATGGGGTGTTACC
**CoV-bait-1597**	CCCACTCTAACTGGTCTGAGTCAGCAGGGGCCTCCTTGTCAGATGGCTTGCTATTCTTCTGCTTCCTCGGCTGCTTGGGCTTGGAGGATTGGTCAGATTCAGGCTTCTTGGCCTTAACAG
**CoV-bait-1598**	AAATCACGACCCTGGTTATCAACCAAGGCAACATTATCATAATGCGGAGGTTTTGGAGCATCTGGAAATAGTTTTAAACTGTCACACATCATCTCATAGACAACTGCTCGCAACTCCTCT
**CoV-bait-1599**	TGCCATCTGTTGCCATTTGTAACACTGTAGAGCCTACAACAACGTCAACAGCCTCTAGAGCCCTAGTAGGAGTGGCATCTGTTATTGCGAGCGAATCATGTGTTACTACTTTGTTGGTTG
**CoV-bait-1600**	GTCTACGAGTGGGAGGGCCATACCTTCAACATGATTGTCACATGCGACTTTAACTGTACCCATTACAAAAGTGTGGTTCAAGTGTCTAGGATCTGTTTGATCGCATACGTGGCATGGAAG
**CoV-bait-1601**	CAGTGTAGAAGACACATATCATCTACACAATCAATACAATTGGGATGGTAGACCTGATCCCAATACTTAAAATATTTCTTAAAAAGAGCATCCTTATGTTCTGTAAAATCATACATAAGA
**CoV-bait-1602**	GATTACTAAAATCTCTTTAAGAGTTTCGCAATTCTTCTCATCGAAGTTGCGAAGTGCATGAACAAGGTCCATCATAGTGTACTTAGTCAAATCGTGACGTGCAATATTGCCATAAACAGA
**CoV-bait-1603**	GACTTCCTATTAACAGCACGAGCCTCTTTGTACATCTGTGATGCAGCCTGTTCAGCCATGCGCTGAATTTTACGCTGCACAGCTGCTTCATGATCAAACTCGGCCTTCGCAATATTCATT
**CoV-bait-1604**	AACACACCCTGTATGTCCATATTAAAGATGGACATATGATAATCAAGGTACTTAGCCAGCAGGTCACGCACATAGCAATCCCACGCCAAATTGGAGGTGGCAGTGATAACTATACCAGGC
**CoV-bait-1605**	AACACGCCGGTACACTAGTCCTACAGGTTGGACGGTGTAACCGGATAAATCATAGACTCCCGTATTGGGATTCATACTTTGAGTCTTACACTTTATTTCACTGGTATAACTACTAGCACA
**CoV-bait-1606**	TTAACACCCAAAAACACATTACCAACAGAAACAGAAAAGTTGTGCAAACGCATCATGCTATACTCATACTCATAATCTATGGTATTAGTAGTAGAGGATGCCATCACATGTCTAGGACAA
**CoV-bait-1607**	GCACCACTCATATCTGTAAAGCCATTGTTGTGCGCCCACTCATTGAACGCCTCAACACTAAGGCGCTCAGGTGACACAAACCAATTAACACCGTTAAGAAGAGCACCATACAAAAATGCT
**CoV-bait-1608**	GACGACTGAGCAGGCGCATAAAACACCCGGGCAGGAGCTTTTACACGCCTGCATTAAACCAGCATCTAGCACTAGCTAGCCGCGAAGGCGCTTAGTTGAGATGTCTCGGATTTTAGGCGG
**CoV-bait-1609**	GAAATTGTTAGACAGTTGTGCTGTTAGGTGAGACAGTGCCTCACCTTGACTATTAACAACTGTTTGAATTTTATTAATGGCAGTAGCCACTGTAAGTAAAGACTCAGAAGTTTGCTTAAT

**CoV-bait-1610**	CCGTATTATCTCTCAAATCAGGGTAAGGCAAATACGTAAGGTTGCTTGACACAGGCACAAAAGTGTCAATGTCATAGAAACCTGTAGAAAAATCGAATTGTTGATAAGAACAATGCAATT
**CoV-bait-1611**	TATAGGCATACTACAACTATCTGGCATTTGTGTAGTAGAAAAGCATAAGCGATCAAATTTTTGAAAATTATTAAGCTTATCAAATGAAAATGGACAATTGCCAGTCATCAAATAAATATG
**CoV-bait-1612**	AGGTCCAACAATCTGCAATGTATGCTGTGAACAAAACTCATGTGGTCCAACACTAAGATCTGGTTCTACCCAACACTTAGATGTGGACATAAAAACATTATTCTGGTAATAAAGTGTTGC
**CoV-bait-1613**	ACCACCAGCTTTAGAAGTAATAATGTTAGTGAAGTTGTAATCACTCTGAAAATTGCTAATGTTAGCAAGCGTGAGAGATTGAGACGATGTTTGGAACATATTAGTAATCTCTCTGCTATC
**CoV-bait-1614**	GCTTCAAGGACTGTTGATTTGATGAGGGCCAAGATGTAAGCGTGGCACAATTCTGTGTCGTCACTGGATTGTTCTGAGATTTCTTGGTACAGGGCGTGGAGTTTACGGACCTTTTTGACA
**CoV-bait-1615**	CAGAGAAGCTAAAATCTCATCAAGTGTTTTGTTAACATCGATGTAATCTGGGATTACATCTGGTAGTTGGTCTCTAGTCAAATTGACATAGGTGACCACACAACTCTCAATTTGAACAAA
**CoV-bait-1616**	TTATAATCTGCAGGACTTATATCTTCAAGCATAACACCTTGATGATCAAAAACAAAAGTGTTTGACTGAAAGTCCAGCGGGTGGTAAACACGTCTCATCTCTGAATAGTAGTGTGTTACT
**CoV-bait-1617**	TTTGGTTGAATTGGGAAAGCTTCCTTTTTGGAAACTGCCATTTCAACAAAGTGACCTACGATGGGGTAGGTGTGGCCATTGATAGTAAGACTATCAGCCACGTAGACACGGCGTGTTTTA
**CoV-bait-1618**	CATCAAAGGACAACTGGTCACATCTCAGTCTGTTAATAACAGAGTTGCAATAAATTATATTAGCAATAGAGGTTTGTGACACATTAACCAAAACGTCAGCATAAGAAGCTAACGCAACAG
**CoV-bait-1619**	ATACGAGCTATCGTGCACATGCGCGGTGCTTTGCAATTGAGACAAACACCATAAGCAAAAGCATTCTTAGTAGGTGTACAAAACAACAGCGCGCCAGATTCCTCAATTACGCCACTAGTG
**CoV-bait-1620**	ATCCAAGCGGGGCAAGATTGACTAGTCCAATAGCTAGAACGTGACGGTATCGTGTACACGGGTTATGAGAGGTAAGGGTATAATCGTCCAGGAGGTCCAATATACCAATCTTAGGCAGTG
**CoV-bait-1621**	ATTAAGCGGCAATCCCAACTGCAAGGACGAGATGGACGTTGCATTATACCAACCATCGACACAATACCTACCAACAGTAGGGTGAGTTACATTGGACAGCGGTATGTAAGAAGTAGTGTT
**CoV-bait-1622**	AATAAAACTAGTAGTTGTTGTGTTTCCTATGGTGGAATTGATGAAGCAATAATATGTTTCTTCAACCAGACCAAAGGGAACAAAAGGCTTTGAGGCTTGAAGAGTGGTGTTACTGCAATA
**CoV-bait-1623**	CACTGGCACCTGGCACGTACACGGTGTGAACAGCGTCAGACCAATGTATGCGCACTGTGTCACCTGAAGCACTAAAGCCTAAAACTCTGCCCTTTTTGTCTGCAGTTTTAAAATCAATAA
**CoV-bait-1624**	CTGTTGTGCCAGTAGGTACTTGAACATACTTACCTCTTAGCTTGCAAATACCATTCATACCCGGATGTTCCACGCTGGCACGACAGTATAAACAACATGAAGCGCCACCATAGGAATCCT
**CoV-bait-1625**	GGTTTGAACTGTTAAAACTAGAAAAGTTAAGAGAATAACCAGTATTAATAGCCTCCACAAAAGAAAGCATGCCGTTATCCTCATTATCGTTAACACCAGCTAATATATTATTAACGCTGG
**CoV-bait-1626**	TGTTTAATTGTGCACATACGAGGCGCCTTACAATTAAAGCAGACACCATAAGCAAATGCTGCCTTAGTAGGTACACAAAACAGCACTGCACCGGACTCCTCAAGTTCACCACTATTACAG
**CoV-bait-1627**	TGCAACAAACATACAATACAGCAAAATAGCCAGAACAAATTACTTGAGTGTCCAGTCTTGCCAGCACCTTGTTTAGCAACAATGCTGGTTGCAGGTATTTGATTAACAGCTTGATTTTCA
**CoV-bait-1628**	GCGTTATCTAAAACACGACCATAGTCATCTAAGGTAAGCAACGAGTCTGTAAATAAAACAATAGGTTTATTAGCAGCAACGCACAACAAATCACCTGTATAATAACTAAAATTACCGTCC
**CoV-bait-1629**	CACACATAAGGCGTAATTGCTAAAATGAACTTGTGGTTAGTGCCTATAACATGGTCATAAGCGCACTTAGTGCACAACATAGGCCTGCGAAGACAATCACCACATCTCAGAACAGTCTGA
**CoV-bait-1630**	CTGTCCATCTGCAAACTTAAACTCCTTTCCCTTCTGGAATTGGGTAATGCCAGAAAACCAGGAGTAATGGGGAACCACACTCCCGGAATTGGGTTGAGTAGTTGCAGTCTGCTTTGGCTG
**CoV-bait-1631**	TTGTAACAGCTGCTACAAACTCATCATCAGCTACGTCAAGTCCGAGTTTTTCCTTACATTCAGCCATAGTATTACAATCTTGCAATTCAATATTAAAACTGTTCTGCAAGGTTTCAACAA

**CoV-bait-1632**	ATACGCCGTCTGACAATGCTCCAGGCTTGACCTCTACGCATTAATGGTATGAGGTGTGCAAACTGTTCACCAGGAGGGGCCCTAGACTTTACGGGGACAATACCGTTGCCATTCTCTGTA
**CoV-bait-1633**	GTTTACCTCAGCTCGGCGTACAAGCCAGGCCCTTAAGATCACCTTATCACTCCAAGTATATCTCGTACATTCGATGTCTGGTCCGTAGATGCAAATCGTAAGACCTTCTCTTTGAGCACG
**CoV-bait-1634**	GAGTGACTTCCTGGCAACACCCACTAATAAGGCTAAATTTCAAAGCATTAGCGGTGGCACTAACATTCTCTACACCACAAGACTCCATGGTTGAGCAAAAATCATCCTTAAAACGGTTGC
**CoV-bait-1635**	CTCCTAGCACCATACCACCGATGAGAGACGCACTATACATGTGAAGCTTCTCAGCGTCAACAACACCAGGTAGTACCATGACACCAGAGTAATACTGTGCACAGACTAGGTCTGCCACAG
**CoV-bait-1636**	CTCAAATTATCAGTTCTTGCCTCTGTTGAGTAATCACCAGCTTTAGATTTTACATAGTAAGCCCATCCAGTCGCACTACTTGCTTTCAACTTCTTGCCAACAAGTGTGTAGACAATAGTC
**CoV-bait-1637**	TGTAAAGTCTGAGAAGTTACAATGAGCTGTACAAAATTGTGAAACTGACCAAGACATACCATCAAGTGGTGCTGTCATGGCTACAGAAGCAGCAGAAATATTATAGCTTTCATAAAAAGT
**CoV-bait-1638**	ATCTGTTGGTTTTTATTCAATACATCAGTTTGTAGAGCAACATAATTAAGTCTAGCCTGTACTGCTACTGCAAAAGGTATAGCCACGGCGCCACCACCAAGTGCACCTAATGTTATACCA
**CoV-bait-1639**	ACTGACTGTTAGCTTACCTGGTCTCTTCTGATATGCGGCCGCCGAACTTGCACAGATTAAACAAAGACTAACTTTTGGTCCACCCACGGTCGCCGGGCTCAGGAAAAGCACACTTATGAG
**CoV-bait-1640**	GTCTAACGGTTTGAAATGTCAAACCAGTTGTAAGAGCTGCAACACTAAAACATGGTGTCCTCAAATCCCAAAGGGCTGATGCAGATGCTATATGCATAGCAGGATCTGCTGCATACATCA
**CoV-bait-1641**	CATAGTATGACCAACCAGTCGCACTGGATGCTTTAAGAGACTTGCCAACTTGGTGATATATAATAGTATTACCAGGTGTGGCTACCATAATGTACTTGGGTAGGTTATCGACATTTACAC
**CoV-bait-1642**	CAAGACGCAAATTAACAGTGGCAAGTTCAATAACAGAAAACTTAACTTCAGTTGTAGGACCTACAACTACTTTAGTAAACATGGCTTTCTTTATGCGAGCATCACGAACACCACGAACAA
**CoV-bait-1643**	CATTGTCAAATTCAAATTCTAGCCTGACACGTGTAATAGGTTCTATCTCCTTAATGTTAACATTATCTGAAAACGAGACTGACTTGTCACCACCACCCATCTTGTTATACATCTTTTGCA
**CoV-bait-1644**	GCACTAGTTGCTTTAAATTGCAAATAGCCTAAACGCTGACGTGTAGCGTAGAAGAAGACTATTTTCAAATCACCTGTTTCATTGAACACTCTAATTCCGAGTGGAATGGAATTAGATCCT
**CoV-bait-1645**	TTGGGCGACCATGCATATCTTGTCATACAGCCTAGGCCCGACTGGGACACTGTTTAACTACTGGGCTCATCAGCCGCCTCGTTATGTGATCTGTGGACGATCACAGTTAGAAATCACGAC
**CoV-bait-1646**	GGTGGGAACATAGCTGCCGCAGTAGCACCCGCTGTGTAGCCAGAGATTTGACTCTCAGACAACACGGGAGGTAATACTTTGATGCCATTAAAAGACTGTACGCAAAGGAGGTCGCGAACT
**CoV-bait-1647**	GGAAGAACTATCGACGACGAATTGGAGTGTGATCGACCTACGGGCGTGGGCGTTTTGTGGTCCGCTCCTGCCACTGTTGGGCGCCACGCAACATCTCTCTCTGACTCGGAGATGCCATGC
**CoV-bait-1648**	GTCGGCGTTTCATGACCACGCCGCTTGCCCAATCTGAATACACGCCAAAGCGCTTGAGGATGGTGTCTGACGCAAGCAGGTATGCTTAGTCACTCTTACGAGCTATGAGAGGGTAAAGAC
**CoV-bait-1649**	AAAATAAGTCACTAGGGCCAGTACCATTACTTTTCATAACAGCAATACGAATATAGTTTTGTGGAATAAGACCTGTTAAAGGACAAAATTGCTGTCCGCTTTTAAAGCAGTGTGTAACAA
**CoV-bait-1650**	TTTCAAAATCTTCATCCTGTTGAATATGATAGCAACCAGTACGTGAGAGCCTAAAGCATGTGGGAACATATACCCTATAATTGCGTTGTGCAATAACACACTCTTCAACCGAGGGTAGGT
**CoV-bait-1651**	AGATGTCAGAGGGATTAGGATGGAGTTGGGGTAAGTAACTGGGTGGATGAATGGTTTACCTGTGGTGGATCCGCGCATGGTAGGGTTCAAGCCGGCATGTAGGGATGTGGTGGAGGTTAT
**CoV-bait-1652**	TACCATCAGTAGCCATTTGTAAAACAGTATCACCCACAACAACATCAACAACTTCTAGAGCCCTAGAAGGTTTATCATCAACAGGGTGCAAACTGTCTTCATAAATGACTTTATTTGTAG
**CoV-bait-1653**	AAAAGGGTTATCTTTACTATTGTTCATACCAGCGAATTTGTTGGTGTAAATACCAATCTATACAAAATATTAACTTGAAACAATAGGAACCTCTTGCAACTAATAAGGAAGCAAGTGGGT
**CoV-bait-1654**	GAGTAGAAAAAGAATGGCAATTGCTGAAGATCAGTAAATGCAGACTTTTGAAATGCAGGCGTGTGGAAAGCATGTTTATTTACGTATAAACTACCTCCATCACAACCTGGTAAATTTAAT

**CoV-bait-1655**	TAAAAGGATAAAAGCCATCTGAAAAATTACCAGTATTATACTGGCAAGCTAACAAGCCTCTAGGTGAATCATCACACAAGATAACATCTTGTGCGGTACCATTAACAAAATAAGCTAGGA
**CoV-bait-1656**	AACTCCTATGTAAGATGTTAAAATCAAGACAGTCTGGTTTCAAAAGCTGCAAGCAATTTACTTTCAGACCAGTGTCAGTCACACCTAGAACATTATTGACAGTTTTAAGATTGAGAGAAT
**CoV-bait-1657**	ATGTACTAAACACCTTTTATATGTCTGCCGGTGCCCCCGTACTCACCATACACGTGCTCACGATACGCTCTGGCTAATGCAACCATGCACGAGCTAACTTCCATCTCTCCGTGGCGAAGC
**CoV-bait-1658**	CTTCCGCTATATTAAAGACAGGATAGAGCTTAGATATGGTATTGTACTTTTCTTGGTTGACTAAAATTGAGGCTTTGAGGGGACTCACATTGTGAGAAGTCAACACAAAAATCATACCTG
**CoV-bait-1659**	TTTCAGGCTAGAAGACTACCGGGGTCCCCAAGGTCAGTGTTCTCTCCAGTCGCGCGGGACCGTCAGGAGCCTGCTAGGAGCTGGATTAGCGACCGGTACAGATTAAGCGTGTCCATGCAT
**CoV-bait-1660**	CCAACGGATAAACCACCCCATGCACATTGACGACCTGCTGAATGTTGGAAATTACAGATGTCAGTCATGAAGGAGGTTATGAGAGCTTCATCATAGGGGAATACAGCTGCATTACCATTG
**CoV-bait-1661**	CAGTTAGTGGTTGTTCATCTGGTTCAACATTGTCATTAAAACTATAAAATGGAGCCATTAACAATTGAACTGGTTGAAATAATCCTTCTTCTATTTGAAAATAACGTTGTGTCATTTTTA
**CoV-bait-1662**	TTAGCAAACCCATAGTATGACGACCAATCTACGTCATCCACTTTGGTCAAGACTTCATGATCAAAGTTCGCATCAGGGTAAACACCGTCACATAACACATTCTTATTAACAACCTTAGAA
**CoV-bait-1663**	CATTGTCATTCACTGGAACACCACTACCTGGGAAGTGTGGTTCAGGTCCATCTGGTTTCCTAGACTTAAGAGCCTGGAACCAGGACGCTGATCCACTACCTCCTACTTTTGGTGGTCCAC
**CoV-bait-1664**	CAAAATGTGCAGTAAACGGCACATCAACTTTAAGAAGAACCATAGACTCGCCACACTTACAAACATGTGACACTATGTCTCCATAAAAGAATATAGCATCCAAACCTTGAAGGTTTAGTG
**CoV-bait-1665**	CATAATAATACAATGCGGTGTAGGGTACAACGCACAACGGGCATGCAACCACAAAGCAAAGACATAAGCTATTCATAATAGTTGCACAGACAGCTATAACTGCACACTGAGTATAGTCGG
**CoV-bait-1666**	GATACAAATCTAGCAGGGCGGCCAGAACGAAATTCAAGCCACGCCTCCTGCCATTGAACAATTTTAAATTTCAGATTCAAACTCTGGAGCATTAAGCAAGAAACATTAACAAAACAATTG
**CoV-bait-1667**	GCACATAGTATACTGGCAAACAGAGATCTCTAAGAAACCTTGTAATTTATTATCCAAATTGGTAGTATGTGGTTGTACTACCACACTATAGGATGTATTTACAAAAGTACTACCTATAGT
**CoV-bait-1668**	AAAACAGTCATAGAAAGTATAATGACCATTACTCTCATCACCATTGTAAAGAATCCTACCAACGTTAGTAATATACTGATCTGAAACACAGGGTTTTGAACTAGTAAGAATGCAGTCACC
**CoV-bait-1669**	TTCTTTATAATGGTATCCAATTGAAACAACAAAAGGAACACCATCCACAAAAATTTGCCTAACAAGAGGCCCAAAACATGTATTAGGTAAAACCATACTAAAAAGTATGTTAAAATTAGC
**CoV-bait-1670**	TGAAACAAACTCCTGAATAACCAGATTGGAAGTTTGCTGTCTTCGGTACAAGCCTATTAACAAATGCAAACCACCAATAATCTTCTGGTTGAAATTACCATAAACAATGTGTTCAAAGGC
**CoV-bait-1671**	TAACCCAAATTACCCAATTGTAGGTCAACCTTCCTACCATTGGGTATTGCAAACTTATCTATAGTTATGCTGGAAAAACACATACCATATATCTTAGCAGCATCAATATTATTACAAGTA
**CoV-bait-1672**	CATATGCATGAGCACCTGGTAATACAGTAGGGTCATAGCAGTAAGGCACCATTTTGCCCTCTGCATCCCTAAACATAGTACATTCCGAAGGTAGAATGCAACCACTGTCCGAGAAACTCT
**CoV-bait-1673**	GTCGGTACAGCATGACCATTAGTGCAAGCAGGGTAGAAGGCAAATCCGTCTTCTTCCTTACTACGCACAAACAATTTTTCACTAATAATGACATAGTCACCTACTATAACATCAGGACTA
**CoV-bait-1674**	GAATCATTACCACAACTTCCACACTCAATGAATGCGGAGTGGTAAATGTAGGTTGGGTTCTCAAGTGACTCCTTACCATAGAAGGTGTAAAGGAGTTTTTGTTTGAGGGACAAGTCAGAA
**CoV-bait-1675**	AACAACAGGAATACCTAAATCATCAACAACAAAACTGACATTATCCTTAACAAAAGGCTTGTTCAAGCCCTTGCATTTGACAATAGTCATTTCTGATTGAGGTGGTGACACCTGTACAGT
**CoV-bait-1676**	ATCAACAGAAAAATCCAACAGGAAAGTTAACGGTTGAAGTTTATATACGTAGAAGGCAGCCCAAGCTTTTCTATCACTTTGGATAGAACGAATACTGTGAGGAATGATAGAGTAATACTT

**CoV-bait-1677**	TCAAGACCTTGAACCTTAGTAAAGGTAGACCAAAGATTATAAATATCAAAGGTCTTATAACACCATAAGCGGAAACCAGACGCAGATACAAGGTTATATGCTTCCATATAATCCCTATAT
**CoV-bait-1678**	ATAAAGAAGCTGTTCAATAGCAACACCTGTCTTAACAGCTAACATATCAATGGACTGAGTGCCAACAAATTCAGTGAATTGATTGGCAATAGCCCACTCATTAAAAGAGACAACACTAGT
**CoV-bait-1679**	GCATTAGAGGAGGCAATGTGCATAGCAGGATCTGCTGCATACATCATTAACTCCTTAAGAGACAGTCTATGCCTATGGAGACTAACATCCATATTCATGACCAAACCTAATTCCTTGTAG
**CoV-bait-1680**	TTGGGATGGTAAGTGTTTGGTCCAAGGGCCGGTGGAGCTAGCACGGTGGAGGGCTAGCAAATTTTGAGCGTGGGTGTTAGTCACTCTCTGTTTAGGCCTGAAAAGGCCAAAGGGTCTAGT
**CoV-bait-1681**	TCATTCATTCTGTCTTTCTCTTAATGGTGAAGCAAAGTATTAAAAATACTAGAGCAGCAACAATGAGAAAAAGTTATTTAAAACAACAAGTACGTCTCTAAATGCAGCAGTTTGGTGACC
**CoV-bait-1682**	ATAGTAGAGCCGATGTCTTCGTCTATAACCGCAACAACAATGGGACAAGCCATAGAATTGCGATAATATATAAGACCAAATGTAGACTCATACCACTGATCAAAATATAAAAATTTATTA
**CoV-bait-1683**	GAAATCAATTGGTGCATCAGTAACAACTTCCACATCAAGCAGATCCTGTGAGATCGTGTCATCATTCAACGGCCACTGAGAAACCATTACGGGAAGATTAGGATCGGTGCCACCTTCCTC
**CoV-bait-1684**	TACGGTTTATTTTACGCTGCACAGATGCTTCCTTATCAAACTCTGCTTTTGCAACATTCATAGCTTTGCGTAGCTGTTTGACAACAGATTGACTAGCATCGGTATTAACCGCATTTTCAT
**CoV-bait-1685**	GTGGGATTATAGGCATAAAACACTCAAAACCTGACATCGTGCAACAAGTGGTTACGAACCCTTGCAGATCCTGTTGTTCATGGGCCGTAATGTAAAACTCATGAAAACCAGCAGTACCAA
**CoV-bait-1686**	AACGCAATGTAATGATTTAAATTTACTATAACTAGAAGTGGTAACTGTTTTATTAAAAATAGAATTACCACTACTATCTAAAACACCAATACGAATATCACCCTCCCTTAATTTACCTGT
**CoV-bait-1687**	TGGGTATCAGGTATTAGTTCAGCATAAGTCTTATGAGCCCCTAGCATGACTCCGTCCTTGAAACAATACACTGCAGTACCACCCATACCAACAAGCTGGGTGCAAGCTGCATTGAAAACA
**CoV-bait-1688**	ATGTATCCAGTTGTTGTTTCTCTGGTTACACTGCGTGCAGTTTCATTGATTGTAGTTTGAAAGATGTGGTCGAATGTGACTTCCTTAATGAATGATGGTGAGAATTTAGGTTGGATGGCG
**CoV-bait-1689**	GTTACGTTGGTTAGGTTTAAACAAGTTATAGCCGGTGTTGTTGGTGTTGGGGCGTAGGGAGATTTCTTCTATTAGTGAAGTTACTGTGGTTTCCTGTTCAGTTATTGCACTGTTCCAGTC
**CoV-bait-1690**	ACAAGTAACATAAACTTCATAAACTTAGCAAGCAACCACCACTTAGACTTAAGAATGGCTGCCGAAGCTTTTAAGTTGTACTTGAAGCTACGTTTCAACACAACACCTGTGCGTTGCGGC
**CoV-bait-1691**	CCTATAATCTTTAACAATACATTGATTTGCATCTGAAACAAATTCCCGTAGGTCGACGTCAATTATACGTGTGCCTTCTGGAAAATATTGTCGTAAAACAGAAGTGCCAGGGGCAACACC
**CoV-bait-1692**	GACCTTGTGCATTAAAACCCATGAGAGAAACATGGTAATCAAAATGCTCTGCCAAATAGTTGTAGACTTCAACATCAAATGCCAAATTAATGCAAGATGTAACAATCAGAGCAGGTATTA
**CoV-bait-1693**	AGGCACTATTCTTACTAAGGAAAAATGCCAACAAAGCAAGTAGCATTTCCTGCGCTTTTTCTGGGTCATTACACAAGTTGATCTTGTTATGCAAGTCAACACAATAAGCCCATTCTGTTG
**CoV-bait-1694**	TTCTGCACATTGGCTTGTTGTCAAGACAACAGCAAAGCACGAGACCATCAGCCTTAGCACGTTGGAGCATCTGCTTTATGTTATAGACGACCGCTGGACAAAGCTGTTCCAAGTCATTGT
**CoV-bait-1695**	ACCTGCCGCGTAATTTGCACAACCCATCAACATCTGGGTGTTCAACTCGTGCGCGGCAATATATACAAACAGACGCACCACCATATGAATCCTGACTAGTGGTAGCATCGGGTTTAGCAG
**CoV-bait-1696**	GCTCTTTCTTTACCGCTGATGGCGTACTTTAAGTTGAGCTGAGTCATAGTAGGGAGTATATTACGCTTTGTGAGCGCATACAACTCATCCTGTTCCTCATATGACAAAGACTCATAATAA
**CoV-bait-1697**	GGATTTAACAGTAAAAACTAAAGTTTGGCCTACAAGGTGAACATCTGCTGGAACACCAGGCAGTGTACGACCGTTATCACCAACACCTACAATTACTGGACAAGCCTTACCAGTTGAAGG
**CoV-bait-1698**	CTATCGCCAGCAATGCATAGTCCGGGACTAACACGCGCAGTGGTAAAGGATGTAGGCACATAACTGAAGTGTATAAAATATAAACCATAAGGCGCATTCTGGACTAGTGATAATATATGA

**CoV-bait-1699**	CCAGTAACCGTGGGTGCAATCTTGACTTGGCCAGACTGAGATGGGACGTAGCCAATACTGCCAGATTTACAAACACCTATGTTACTATACACCAACACAGGCTCTGTACAATTAGAGCCA
**CoV-bait-1700**	GATGTCAGTTCCGGCGCGAGAAACGATAGGGCAGAAAGCAAGGGTGAAGTGGCTTATTTGATCTGATGTTTCTGTAAGCGTTTGAGTAGTTTCACCGAGGAGTTTGATGATACTATTGTG
**CoV-bait-1701**	TTTTTATCTTCTACCACACAATCACCTAACTGCTGACCATAAGTCTGGCTAGTCGACAATTCCACAGTATGAAAACTACGCTGGTCTTGAGTAACAACAACCTGAACAGGTTTAGCCTCA
**CoV-bait-1702**	TCTAACCCCACCTTAAGAACACTTGCATTAACCACAGGCGTTGAAACTACACGCTTGGTTGAGCAAGTACCGTCACAAGTGGTAGATGTAGCTCTTAACATTTCTACAGAACCCTGAGGT
**CoV-bait-1703**	CTTTCAGGTACACCGGCCATGACTTTCATATCCCTCTTTTTAAGGGCCCTAAAACAAATAGCAAGCAAACTAAACAGGTAAACAATCAACACAATAATGTTTCTAGAAACAAAATCACCA
**CoV-bait-1704**	CACGGGCTTTCTTTGTGCGAGCGTATTTTGCAATTACCTTTGTTGTAAATGGTAGTAGGGTTTCTTTTGCAACTTCAAGCATGCGTGGGCCAAAGTCAGGTGGTAGAGACTGCCAAACAG
**CoV-bait-1705**	CTTGTGTCACATTAGAACCACTATTGTTAAACACAACAAAATCAGTTAAAATATTACAGTTTTTAAGCACATCTTTACAGGCATACTTAGCCTCCGTAATATCAAAATTGTACTTCCAAA
**CoV-bait-1706**	AACGTAAGCATAAAATTGATACCCTTAGTCTTAGTGGTTCGAACAATATATTTGCGAGCCTCTTCAGATAGTGCAATAAAGTCCTTAGCAAGCCAAACAACAGGAACATTGTCTTTTGTA
**CoV-bait-1707**	GTACAAGCAGAGTTGAAAATGCATGAGTCTGGGTCTGTAAGGCCTCTATCATCAAAACAAAGTCCTGACGTGCCAAAAATGGTACTCATAGCAAAGATGAGTGACTTGCCATACAAAAAT
**CoV-bait-1708**	ATAGAAGGTGTCAAATCATAAAACTTAGCATCACCTTCAATTCTAAAAGGTTCACAAGGATTTTCAACCTTGACATCAGGAACAGGTTTTTCTAATTGGACACCTTTCACCTCCTGAATA
**CoV-bait-1709**	TGTTTGACTAGTGTGACATTTTCTGGTTTGTTCTGGTCAGCGATGGCGAGCATACGGGCTGCTGTGTCAATACCTGTTACTGTTAGTGGACGAACGGCATAACGGGGACCGTCATTACCT
**CoV-bait-1710**	CCACCGCCATTAGCCATAGTTCTCCAGTATCGCGAGGAAGCAGGCGTTGGAAGCGTACGGTGATGGAACAGCCAAGACGATCGCCCTAGGACAAAACTTTTCGAGTCTCTACTCTCCCCT
**CoV-bait-1711**	TGCAATGCGCGCTGCAAAATTCGCTTTTGCTCCACGCCATGCCCTGCAGCGGCGCGGTCATCGCAATGCTCGCCACGCTCTGGTTATACACATCTTTAATCACGCCCACGGTGCAGCCCT
**CoV-bait-1712**	CTGGGATGGCAAAAATATGAAACGCCCGGAGACAGCTGATTGATATAATCGAGGCGCTCGAGCCCCCGCCGCAGGTGATCGTTTCTTCTGCAGGATGTCGAGCGATCAGAGCTCGTCCGT
**CoV-bait-1713**	AATCAGACCGACGATACGAGTGGGACCGTGGTTCCAGACTAATAATCAGACCGACGATACGAGTGGGACCGTGGTCCCAGACTAATAATCAGACCGACGATACGAGTGGGACCATGGTCC
**CoV-bait-1714**	TGTAACCACAAACATCTTTTGTGTAACTGGTAAAAGGTCTATTGGCCTCATAATCCCACAACACAAATTTGTAAGTGGCTACAACGTCGAGATTGCGTAGGACAGAAAGTGGTGGTGTCA
**CoV-bait-1715**	CAGTCATGGTTATAATAGGCATAAGATAAGAATAGTAACTATCAAAAATAGGAACACCTGCACCAGGCGTTGTCTTTTGAAAATCACCAAAGTCATAAAACTTGCCATTAAGATCCTGGT
**CoV-bait-1716**	TCATAAGACAAAGATTCATAGTACAAACCCGCCTTGCCGAACTTATTCAAAGGCCAGCCAGCACTCTTGTTTAGATTAGTCACAACCACATCACGAGCAGTAATGCAGCCACCCTCATAA
**CoV-bait-1717**	AGGTAAAAGATATGTAGTTGTAAATACTACAGAGTGTTTCACACTCAGTGTGATTAAAAATGCAAGACTCACTACAACTGGCAAACCAATTGGATATAAGTCTACTGGTAAAACATGCAA
**CoV-bait-1718**	AAAGGTGATATAAGATCTTTAGGACTTTGACATTCTTCTACACGTTCTTCCTTAGCTGGTGTGGGTGGTTTGATTGTAGTTTTAGACTCCTCTTTCATTACATTGGCATTAAGAAATAAA
**CoV-bait-1719**	CACGCCTACCGTTAACAACTTCACTAGGAGAGGCAAACCCAGTATGGTCTAGCATGTGGAACTCACCAGCCTTATCAAAACCATAATGTGAATCCCAATTAGCACTTGGTACAACCTTAG
**CoV-bait-1720**	ATAGTACCTAAGGTAACCCCACAAGGCCCACGTGATGGTAAGGGGGAGGTTGCAACCCGTCCACTTGGCCCCTTCATCCGCATTCGTTCCTCTCTGAGAAATGGAACGTGATCCTACAAT

**CoV-bait-1721**	TATTGCCAACCTCCTTGACAAGAATTGCAAAAGGTGTGCAATGAAGCTTAATAGTAACATCAAGTGCAACAAGCACAATTGGTCCCCGTATAGCAATTGCAAAACTTCCAGTCCTATAAA
**CoV-bait-1722**	GCCATCTATATGACCGTTAAGAATAACAGAAGCTTCAACTATAGCACCGGTCATAGTTTTAACTGTACTATCACAATGTTCACATAACGTAGAACGTTTAATAGTAACAGAACCACTAGA
**CoV-bait-1723**	AAATTGCATATAATATCCATAGTTGAGTCGGCTCCAGTGGTAGGAAATACTGGATTCAACAACACTCCAATCATTTTCAACCCACACTTTAGTAATGGTGCCACCTAGATAAATGTTGAC
**CoV-bait-1724**	AAATTAGTCGATGCTATGGTCCAAAAACCAGAAACATCATCGTCAGTGCCATGACCAGTGAAATTAATTGTGACAGCATCCAACAAACCGAGATGCAAGTATCCAAACCCATTGACATAA
**CoV-bait-1725**	TCTGAGGTTTGTGTGTAAATGACATAGTCATACTCACTACCTTGGGACGAATCGACTGTTTGGATTTGAAGTCCCAACAGACGTGACGCAACATAGTTCTGGCTATTGTAGGGAGAGATG
**CoV-bait-1726**	CGTTTAACTAAGTTATGAACACCACCATGTCTAAAAAGTTCCATTGCTGTATCATCATAACCTAGCATTTGCAATTGATAGCAGGTTGCATTAACCCAACAATTATTCTGGCTCTGTACT
**CoV-bait-1727**	TTTTTAGCCGCTGCCAACAATAAAAGAAGAGAGCTATTATTCGATAGCTCAGGGATTACCAAGATCCCTTTAGGACGTGCTTTATTCGCCATACTAGTAGAGAACAACCAGAGTGCCCTA
**CoV-bait-1728**	GGGTGAATGGTTGAAGTACCCATAGTAGTATAAGTTTAAGAATGTAAGTGAAACGGAACCTGGTGGCATACCCGCCCTGGAGTATGATACCAAGAGCCCAAATAATAACAATAAAAACAA
**CoV-bait-1729**	ATTAATAACATCAGTATATGTTGAAACATTCACTGTTTGAGAGAATAGTGTATCACCATTCAACCAGACTGTAAAATTTACAGTTGTATTAACACCTAAACCTGTGAGTAGAGATGCTGT
**CoV-bait-1730**	TATTCAAATAACTAATAGCAAAATTAAGCGTAGTAATGTTAATAGCCATCGGTAACCCTACAAATGTGCGCTGGCGCTGTAGGACTTCAATGGCAGATGTGGAATAGAACCCATCAGAAA
**CoV-bait-1731**	AAGCAATGACCCATGCGACAATAATTAACTTGTGAGAACCTAACAATGTATGGCGTATACATTATCTGATCAGGTACAACAAGTGGCAATAACTTACCATCAGAACTCTCTACAGTATAA
**CoV-bait-1732**	AGCTCGAGTAAGAGCAACATTTAACCTTGACATATTAAGTGCGTGACCGGAGTCAGTTGTTACACAAAATATAACGTAGTTATATTCGGATCCCTGAGAGGAATCAACTGTTTGAGTAGA
**CoV-bait-1733**	CAATACCTGACAACACTCATTAGCCAAACGGTAAAACCTCTGTCGATTCGTACAACAGGTGTGTTTACGGGCTAATAATAATGAAGAAGCTATGCGCAATATATTCGGCATAGAACGATC
**CoV-bait-1734**	CAGCTGTTATAACTCCCACTATCTCATCGTTAATCACAGCTGCTTGTGCTGTTAGATCACATGGCACAACAGTAAAGATCTCCCCAGTAGTGCTATTTTTAAAGGCAAGAAGGTCACCAC
**CoV-bait-1735**	TGACCAACATTAGTATAGCAAACCTGTACATGGTGGTCTAACTAGGAGACGCCCAGTTGCCACAAGTGTATAAAGAATTGGTTTTGAGTTAATTTCATTAAAACCAATTACAGGTGTTGC
**CoV-bait-1736**	GGGTCTAAATTATGTTGTTTCTCCATCTGAGACGCAATTTCGTCATCAGTATCAACTGAGCATTGATTTTCATTATCAGACTCATCACCAGGAACCGGTTGCCACTCGTCAGAGTCAGTG
**CoV-bait-1737**	GGCTGGCCCTCACACTTAAAGGTAACAATATTACCAGGTGGTACTACAGCCGTATTTTGCTTATAAGCATCAATAGAAGCTGACAATGCACCACAAGCCTGCTCATCTAGCAACTTACCA
**CoV-bait-1738**	CAGCAATTATTATCACTGCCAGCCAGTGCGCGCTTACCATCGACAAAGTCGCTATCATAAGCATACGCACTATGGTCTAACACATGGAAGTTGCCAGCGTCTTTAAAGCCGTAATGACTA
**CoV-bait-1739**	ACCTTTGATGTTGCAGAACAATACACGCGGTAGTGACCAGCATCTATAGGACCAGAAAAAGCGGTATAAGCCGTGCCACTAATAAGTAAATTTGGCGCCTTAACAGAAGGATCACCAACA
**CoV-bait-1740**	TACCAAATGAGTCACAATTGACACAGAAAAAGTTGTGTTTACTACAAAACTTGGAACCACCATTAGCTGTTACATAAAATGACTTATTAGAACCATTGACGACAGTTGTCAACGGCACAC
**CoV-bait-1741**	CAGAAGAAATTATGCCTAGAACAAAAGTTCTTACCACCATTGGCAGTGACGTAAAACGACTTGTTAGCTCCACAAACAATAGTTTGCATTGGAACACGTGTAAGGCGTGCACTCTTACTA
**CoV-bait-1742**	TCATCAGAAACTTCAAAGCCTAAAGCTTGCTTACACTCATTCATAGTAGAACAATTGCTAAGGTCCCTAGAGAAACTATCACTAAGAACATTAACAAAAGCAGAGTGTAATGCACCATTA
**CoV-bait-1743**	CCATCAAAATCTTTAAGCACACCATGTTCAATGTACTTAAAGCCAAAGTCCTCAAAACTAGTAGCAACATAACCGAACTCGGAAGCATTAAGGAGTAAGAAGAATGCAACAATAGCCAAA

**CoV-bait-1744**	GAGTCAGACAAAGGCACACCAAATATACCAACACTAACTAACGGTGTTAAAGGTACACCTGGATTTGAAAGAACACTCTTATAGCACTTAACAAGCAAAGACCTGGCGTGTTTGCCTTTA
**CoV-bait-1745**	TTTCAACAGTTGCAAGCTCCAAACTACGACTTGATACTGTAATCATATCATTAAGCATTAACGATTCAAGACGTGCACCAAGATTAAGGGCATTTTCGATTGTCTGACAAGCTGAGGTGT
**CoV-bait-1746**	AAGGTTGAATTTGCATAGCCGTCAACATGGTCTTTACACAAGGTTTCATTAGGTGGTCCAAAGCGTATGAACATAAACAACACAGCATAAGCAGTATACAGAAGTAGACAGAATTTTAAA
**CoV-bait-1747**	TAGATAGCACGACCATCTTTCCACGTAAAGAAATCATGTGATGCAACAGCACCACTATCTTTAAGTATGTCATATATGGACTGCTCATGTTCCATGACAGACTTAGGACATTTCTTTATA
**CoV-bait-1748**	GTAAAATCATACTGCAACAAGTCAAAAGTTTTAAAATCACTAGAAAACATATCACTAGACATAAAACACTCACTTGCTAAGCAATTTGTCATAGTCATAATAGGCATCATATAAGAATAA
**CoV-bait-1749**	AAGAACATAGACAACAACCACCACTTTTGTTTTACAACAGTAATGGAAGCCCTAACGTTGTACTTCAAACTCTTACGCAAAACAACACCAGTACGCTCAGGCGCCATAGCCATAATTTTT
**CoV-bait-1750**	TTACAATATTATGCCACCTGAGCCTACTATCAAGCTGAAAACCCGTAAGCTATTTCCGACAGTATATATTCGGACCAGGGACGGCCCTGGAGCGGGATCGGATTAGTAGGGATAAATTAT
**CoV-bait-1751**	AACATCAGCAACAACAGGTTTGTTGTCTGTGACAACGGTGTCATTAACAAGACAAGGTCCAATTTGTTGTCCATAAGTAACAGTGGATTCTACTTTAACAGTCTTAACACTACGTGTATC
**CoV-bait-1752**	AAATGCTGGTAGCATATTGGCCAAGAACACACAAAGCCTCAAGGACCAATGCATAGTTTCCAGCATAAAGAATTCAGGCACTGATATGGTAAGCAACTGTAATCCTATAAGGCAAAACAG
**CoV-bait-1753**	CTGCGACATCGCAGTTGGGTGCATTACAAACATATGGAGAAACAGACAGAACCAACTTGTGCGGTGTGGATACAACATGGTCGTAACAACACTTGCAACAAAGGAATGGCCGTCTAATAC
**CoV-bait-1754**	AATGATCGCTTTGGCGGGTGGTCATTTCCCGCCTATATTACCCATCGCGGTGAAAAGACGAGTCTAATACGAAAGACTTTCACAAATATCGGAACTAAGTGGGCGATGGTGAGCTCCTGT
**CoV-bait-1755**	CTTATTACCATCTATAGATAAGAAGGTATTGAGATTAGCACTAACAGCTTGACAAATATTAAAAACACTATTAGCATATGCAGTTGTGGAATCTCCACTACTGGTACCGCCAGGTTTCAC
**CoV-bait-1756**	AGCGTTCGTCCATGAGTACTGTGTACTTGTCTGGTAAAACATCAGTGTAAGCAGAAACATTTATAGTGTTAGTGAAAACAGTATCATTTTCACGCCAAACTGTAAAGTTTCTTGTACATG
**CoV-bait-1757**	GGTCGGCCATGTTCTACGCACGACTAGAAACAATTAGGACGGGGGTCCGTACTCGGACGCTCTTTGGCTCTCACTGACTGATGAAATCGACCAAACCCGTTATGCATCTGATTCCGTAAT
**CoV-bait-1758**	ACATGCCTATCTGCCTTAACCTTTAATAAACTTCTACGATATAACAAAGAATAAAGCAGAATAAAAACAATAAGCACAGAAAGAAAGCAAACTGCAAGCAACAAGGGAAACCAACTAAGG
**CoV-bait-1759**	TGAAAACTCTTAAAAAGGGAATTACCACTTTTAGCAGAGATCTTACCTGAATGGCACAACTCTTGGGGTAGGTCTAACCAATCATATGCTGGATGACCGTTATTTTCCACATAGCTGCAG
**CoV-bait-1760**	CACCTTCAGCTTTCATTGCACGTTGTTTAAGCTTGCCAGGAATAATTTCATTATTCTGCAACTTAACAATGCGCTCACAATTAAGAACTAAAGGCCAAGACAAACTTTCTGCATTTTGCT
**CoV-bait-1761**	TTGTTGTGTCTTACGATGCTATCTAGTAATTTCTCAACACTTTGACCAGTTTTTGCAGCCAACATGCTAAAAGCATCAGTTGAAGAAAGTTCTGTGAAACTGTTAGTTTTGGCCCATGTA
**CoV-bait-1762**	TCATAATTAGTACACATAGAAACTTCATCTATGACAACAATGTCTGCAGTACACTCGGGTAATGCATTGACAGTTGAAAAGATGTATTGCGCACTATTATTATTAGCCTTAAAACCACTA
**CoV-bait-1763**	GTATAGCAGGAAAACCCTAGAAGAACTAGAAAGATCACTCGTAGTACTACTACTAGTAATCTGGTTTCTGCTAAAACTTTATGTAGGATGTTAAAATCAAGACAGTCTGGTTTCAAAAGT
**CoV-bait-1764**	AACATCTTTATTCCACACCAAACCAAGCTGTTTAAAGTGATAACCAGCTGTGACCACAATAGGAACACCATCAATAAAAACTTTCCTAACTAAAGGTCCAAAGGCTGTAATGGGAATCGT
**CoV-bait-1765**	GAACTGTTAAGTTATAAAAAGGCGAATTACCACCTCGCATAGCAGCAATGCGTATAAAGCCTTGTGGTATAAAACCTGTTAAAGGACATGTACCCGCTCCGCTTTTAAAACAATGTGTGA

**CoV-bait-1766**	CCATCTATAACTACCACAACACCTCCATTCTTTGGTGGCTGATACTCAGTCTCTTCCAAAATAGTTGTAGACCAGACATCATCTTCAACAATCTTTTCAACACTAACAGGAATGCTAGCA
**CoV-bait-1767**	TAGACATTGTACTCAGTACAGACATTTAAATGCAATACAGAGAGATCTTTCAAACCAGCATCAGCTCTTGGAAGACCAACTATGTCTAAACCAAATTTATAACTGACATACACGTGAGCA
**CoV-bait-1768**	GTTGCCAACATCATGTATGGCACTAGGATTAAGAACTTTGATAGCGGCACGGACCACGTGTGATTGAACAACTCTACCAGCCTTATTTATTGTAGCTTCGTTATCAATAAAAGGGTAGGT
**CoV-bait-1769**	AGGACCCCATATGGTAAAACCAGCCTGTACAAATGTATTGTAATCCTCTACATACTTACGGTACAGAGCAGCATGTTTCTTACATACAGCACCACCAATATTACAACGTGTAATACAAAC
**CoV-bait-1770**	CATCAATTTAAAATTAGCAATTAACTAGATCCAAAAGTTGAGCCCTTCCATTGTTGGCTCGTCTAAGCGGATCTTTAAATTACTCTCAAATTTTCAATCTAGTATTGATTAATTCAATCT
**CoV-bait-1771**	GATTTAACTTTTGTACTTTGTAAATTTACGCCAAACATCTGTCTCACAACTTCTGTTGGCGTAAACTCATCACTCAAAGAACCATATCCTAAGATCGTACGACCTCCAAGCCCTGTTGAC
**CoV-bait-1772**	AAGAAGTAACACACGGCTATGGCACACAAGGCTATGATCAAGTTAGTAATCATATAGCCTGATGTTGCAACAACTGATAAATTTTGGTTAAACAACCTAAACACATTAGTTGCAAAACCA
**CoV-bait-1773**	TAACACCAATTGAGTAGTAACATTATTTTCGCGATACACAAAAAACTTTTCTCTAGAAGAAGTTGAATTAGTAAATGGGTAAAAACCATCTGAAAAATTTCCTGTGTTGTACTGACATGC
**CoV-bait-1774**	ATTTGCTAGTACCCTGAAAAATAGTCTGGACAGGAACGCGCTTAAGGCGAGCACTCTTAGAGCAAGCCACACAAGATGCCTTATCGCAGCCAAGGAAAACATGCTTAAGGAACATCAATA
**CoV-bait-1775**	GATCAGGACAGATCTACCGTGCAGGAGAGTCTAACACACTGCGAGGGGGTATGCTGTATTGTTTATGTGCCTCGGCAGGTCACTATCAATAACCGTCTCCGCCCAGGACGAGAAGAGTTT
**CoV-bait-1776**	TAAAGAGTATTTTTATGGCGGGAAAATCGAATCGGTAAGTGTAGCGGTATCAGGAGTTGCAAGAAGGCGGCGTGATGGGCCGTCCCGCATCACTCAACCCACGGTGGATCATTTCAACGA
**CoV-bait-1777**	AGCCTTATGTAAATAAAGCTGGTAACCACTAGGGTCAAAAGGCTCTTGCGAAATGCCAATCTCAAAGCCATGACCACCTCTAATATGGCTAACAAAGATACCATGAACGCCACTAGCAGT
**CoV-bait-1778**	AAAGCAACTAAATCTTGAGCAACATCATTTACTAACTTGACGTCGCATGGTGAATCATCAAAAAAGAAGAATGGCATACTCTTCAGATTTCTAAAGGCACTGCGGTCGTACTTTTCGGTG
**CoV-bait-1779**	CTCTCTCTACGACTTTAAGCTTAGATTGCTGAACACAAGTGTTAAGCAACTCACGCCTATAGTCGAGATGAGACCAACACAAACAACAAATTTTATCATCCTTGTTGTGTAGGTGTTTCT
**CoV-bait-1780**	TCGGTCATAACAAGACCCTCATTAGCTACAAGATAATCAGTACCAATACTAAATCCTACCATCAATGGAAGATTAGTACCTATATGTTCATTGGCTGCGTGAGATGCTTCCACATCAAAG
**CoV-bait-1781**	ACAAATTTTATCAAATGTAAGGCCACTATTTACTTTACTACCAGCAAAGGGGCACTTACCTTTAAAATCCATACCTCTAACACCATAATTATTAATTTGGTCCAATACGCCCCTACAATA
**CoV-bait-1782**	CTTTACTCCATACATACTACAATTATAAGGACCAGTTTGAACCATACGTGTATCCATTGGCACAGGTCCCTTTGTGCGAATTATACCTTCACACTTCTTAATAAACAGTTGGCCTGCCAA
**CoV-bait-1783**	CCAAGGATCTTAACACTAACAAGTTTGACAAGTGCATTTTTGAACAACAGGTATTTAGCACCAAGAACAAAGCATTTGGCCTCAATACCAGCTATTATAATATTCTGCGTAAAAACATCA
**CoV-bait-1784**	ACATCAAAACTTGAAGGCGAAATCTCATGCACATCAAATTTTTCCTGAGTATATAAACGAGCAAGACCAATAGTTAGGTGTGCACCTCCTATCACTTTCTTGCTGAAATCACCATATAGT
**CoV-bait-1785**	TTTTATTTCCTCTAGGTTGCCTTCTTCATCCATTCGCAATCTCTCATAATGGGCCTTTCGCTTACAATAGCCAAAGAGTGCTGGTATGCCACAACAATTGCAGCATCCACCACAACACCC
**CoV-bait-1786**	TATCAATAACCTTACTAGTGTCTACAACACCATTAAAGACAGGCTGACTAAGCTCTCTGTCCCACAATGTAAATCCTGCCGTAACGTCAACACCTAAACCATACAATATGGCACTCTTAG
**CoV-bait-1787**	TATGGCATCATTACTACCACCGTAACAATAAAGTCTTTCAGACACCATCATCAATTTAACACACCTAGCGCTAATCAAAGCAAGAGACTTAATAAAGTCTTCTCCATAGACAACGATATC
**CoV-bait-1788**	CGTCACCGTCCGTCTGTACATGTGAACTACCCCTACCAATATGGGTAGGAACTTATAAACGGCTCCACTCCTTTTTTAGGCAATGTGACCTAATCAGCTAAGTGGACTGGACCTAAGAGA
**CoV-bait-1789**	AACTGCTGTTAGAAAACTACCATTCTCCTCTAGCGACTTACTCAATATATTCATCGTCACCCTCTTCGGCTGACTCTACTTCCCAAAAATTACCTCTTCTGTAATAACAAAGTTCAAAGG

**CoV-bait-1790**	AGTTGCTGCTTTTGCCAATGTGAAGGAGACAGCAGCTTCCTTCGCATACACAAATTTATGGCCAAGAAACTTGAATGGTTCACCTTTCTTAAGTGCTGCCACAAGTTCTGTTATACTAAA
**CoV-bait-1791**	TATAATTGTAGAAATCGGTGAAATGAAAACTTCTAAAGAGGGAATTACCCGCCTTAGAAGAAATCTTCCCCGACTTACACAATTCTGGGTTAATATCCATATACTTATAATTCTGCTGAG
**CoV-bait-1792**	GTAACAACACATCCTTCAGTTTGTACTACGAAATCAACACCATTTGAAAAACCCAGCTGTAGTGGTACATTGGTTCCAACATTATCACCACAGACATGTGCACCTTCGACATCAAAACCT
**CoV-bait-1793**	GGTATACTGGCAAATAGATGCCATAATAATACCATTGTATGGTTCCAATACTACAGTATAGGAAGTATTAACAAAATTACTACCTATAATTATAGTAGGAAAGTATGAAGCCGAGCCTAT
**CoV-bait-1794**	TCACTTTGCACAAACCAATTGCACCTGTTAAGAATAGCAGCGTAAAGCCAAGCTACAACATTAACAGTCTGCGTATAATCTTGAACTGGCAATTGTACAACCTGAGCATCTCTATAGGGA
**CoV-bait-1795**	GTACTAGTATCAAAACCTCTTGGACTACCAAGGTGGACTCTTGTATAAGAGCTAGTGGGTCTAATTAAAGTATACGTAGTACCGTCGAGCGTCGTCGCGCTCGGTGTTACTGGTGTACTT
**CoV-bait-1796**	TCAAGAAGTTTGTCTGTCATGTAGATGAAGTCGTCTGGGTCGTCACTATCGATCAAATTTGTGTAGAATTCTTTTGTGAGTAATTTTTCTGGTAGAAGATTAAGACCGCTGTTGGCAACT
**CoV-bait-1797**	TGCAACTCTTCCATAGAAAAATTGTCCTGTTTGAACAAATGTACGCCTTCGTCAGCAGTAGTGGGATGTCTATCCGAATAATCTCTACCGTCACAAAACACTGTACCGATCTGTGCTCGG
**CoV-bait-1798**	TTTAATAAGGCGGCGTAACATGTCATCCCAACCGCCATAAAACTTCGTGGTGCCTATAACTACAGGCACACCACGAGTAGCTGCTATACTCTTTAAACACTTTTGATGAAACATTCTGCC
**CoV-bait-1799**	AAGATTGACCTTATGCTCTGTGAAGTCATACGCCAAGAGATCAAAGGTTCTAAAATCTTCTCCAAAAATATCACTCTTAATAAAACATTCACTAGCTAAGCAGTTGGTCATACCCATAAC
**CoV-bait-1800**	TATCCATACTTAGGAAGTCCTTTTCCATGTCGCTACGAGGGCTAAAATCAGCGGTTGTTCTACCTTGGGTAAAATAGCCGTCAGGATGGTCCTCGAACTTGCCGTTCTTCCTAGTGTAAA
**CoV-bait-1801**	ACTTTTGGCGCTACGAACTCTTGCCTCTCATCTCTTAATAACAATAACTTAGTGGGTATTACAATTTTACCCCACTTCACATACTCTCTAACAGTCGGTGAATTTACTAGTCGAGACAAC
**CoV-bait-1802**	AGATACATACAAGTGAGATCAAGACCTGGATTCTGAGCAGTGTTGCCTACATCCAAGGTCGAATTGAAGCCATATAGGACACCAGTATCCATATTATAGTAACTCTGAGAGTCAGTATAA
**CoV-bait-1803**	GACAGAGAAGGAAGAAGGTTAGGTGTTCATACTACAGTCGATAGGCGGAAGGTACCGACTAACAGGGTAAGTACCGGAGCCCACTTGGGTTAGCGTTTATAGGTCGTGTTACCCCTTTCC
**CoV-bait-1804**	TTTGGATTCTGACATAAACACGTTATTTTGATAATACAGCACCTGTTGAAAGGCACTAATATTAGCAATATAACCTTTGGACGCATACTCAGAATTATAACATACGACACCATCATCACT
**CoV-bait-1805**	GGGGGCACCGACGTATCCTGAGAGGTAAGATTGGAGAGCTGGTTTCTTACAATGTCTTTTAAGATGGCCGAGAACTTTTGTGTTGATGACACCTGTAGCTGGAGAAGATGTTGTTTTGGC
**CoV-bait-1806**	ATCACTAGGAGTGATATCCCTAATGATACCATTCTCTACCACTTTATAGGTGAGAATGCGGTCTTCATGGAAGGAGACTTGACTCATGTTGAATGCCGGTAAACATAGGTAAGACAACAC
**CoV-bait-1807**	CCTTTTACAAACATCTTGAAGCACTGCTTTGATTCAGGGTTGACAGGTACAAATTTATTTTCATAAACAAGTGCAGAGACTGTCTTAACAATCTCAGCTGGGCATCTATAACATTTGTGT
**CoV-bait-1808**	CGGTCAGGCTACGTTTAGGTCCGTCACTACTTGCGTTTATAGCCTTGTTAAATGCCTCAGCTAGCTCACTAGCCTGGTCGGAAACAAGTCTAAAGCCATCATATGGTGTAACTACTTTAG
**CoV-bait-1809**	TACAAGTTTGTTTGGTAATGGCCAGAACCTCTACTACCCAAATACGCAGACGCACAAATGGGCATGGTTGGCAGTGCATCAAAATCAACAGGCGCAGGGTCCTGAACAAAAACACCAGTA
**CoV-bait-1810**	CAGCCTTCTTGACATTGTCATTCTCAGGAACACCACTACCAGTAAAAGTTGGTGGTGGTGATGTCAATTTTCTAGCTTTGAGAGGCTGAAACCAAGATGCTTGGCTTGAATTGCCTATCT
**CoV-bait-1811**	TCCACCCCATCTTATACTGTTACGGATATAGGTTTTGAAGATGATTCTTAGCCCTGATGTTAAAGCTGGCACTTCCGTAGAGGCGTCGTCCCAGCCGCCAAGGTCTTAGACCCCTTGAAG
**CoV-bait-1812**	AAGTAAAATTATGCAAAACACCATAGCTAGTAGAAGAAGTATCAAGTGGTACAAAAGTAAAATTACTAGCTACAGGTTCTTCATAAACAGGGTATAAACCATCTGAAAAATTACCAGTAT

**CoV-bait-1813**	CATAAAAACTGGTGAAATTGCAATAAGCTGTGCAAACACCGTTTGCTGACCAACTAATACCTTGTTTAGATGTGAACGCAACTGCACTTTCATTAAGAGAAATTCCACCTTGTATAGTGC
**CoV-bait-1814**	AATCCTGCATGTCATTACAACTTGACAGGTCTTTGCCAAAACTATTCGACAACACACTAACAAAAGCACTATGTAAGCTTGCGCCAAAATCAACAGACAAACTGGCCAACAACGCTGAGT
**CoV-bait-1815**	GTGTAAGTCACTCCATCGAGTTTGTATGACACAGGTTTTATATCTGTACTGTAGGATGTTTCCTTATAGAAAACATCAGCAACAGGACCCTTATATTCAGTAATTTTAGTCAAGAGAGCA
**CoV-bait-1816**	CTCTGATTACAACAAGTATGTTTCCTTGCTAATAAACAAGAGGCAGCTATACGTAAAATGTTGGGCATTGACCTATCACACTTAGGATAATCCCAACCAGCAAGAGTAGGATTTTTAATC
**CoV-bait-1817**	TCAAAACACTCTACTCGTGCCCTAGCGGGTATTATTCTACTACAATCTACAATATTATAATTCTTAAAAGCTTTATTACACAAAGAATCCACAGCTGCGTGAGAACAGGCTGTAAATACT
**CoV-bait-1818**	TAAAACCATAAGCAGACTGTAGTGTGAATTACCATGTATGGCTATCAATAAGCCAACTCTATCACGTGTGGCCCACACTCCATCAGAATTATGTTCACGTACGTCAAAATAGACGCCATG
**CoV-bait-1819**	TTATCTTGTGGCGGGTGCTCATGCGACTGGCCGATATAAACTTGAACAAGCGATTCCTGCGAAACGGGCCTCGCACCTGCGAAGTACACCACACAACCGATGTCCTCACGTGCTAGTCCA
**CoV-bait-1820**	TCATTATCAATAACAAATGTAGATTTAGATGCTTCCAAAAAGCTGGAAAATTTACCAGTCGTTTTGGAAACTACGCGTATATAACTGTAGCAGAGGTATAAGACACATACAACAGCAATT
**CoV-bait-1821**	CATCCTGCTCTTCATAGGAAAGTGTTTCATAATACAGTCTAGCCTTGCCAAACTTATTAAGAGGGTAACCTGCACTTTTGTCATAATTAGTAACTACAACCTCACGGGCTGTGATGCAAC
**CoV-bait-1822**	CGCACTGTTAAAGTACCGTCCTTTGCGACAAACTCACAAACATCTTGTTGATCAGGTACTGCGCAATCAAAAGACCACACTGCACATAAACAAACAGGTAATAGAAACACAAAACTAAGA
**CoV-bait-1823**	ACAACCTCTCGAGCATTGATGCTCCCTGCTCCTATTTCGAGGTTCGATTAATAGAAGTCTGCTAAGGAAGTGTATATCATGCGGGCCCGTAATTAGTAGAAGCGCGGCTGTCGCGTGGTG
**CoV-bait-1824**	ACGGCAACAGAACCGACAATCGGCCGCATTACGTTCTTCCCGATTCATAATCCGAACTTTGCGGGGTTGACGAGGTATGCTGGTCCCAGATCTTGGACCTGTAGTTATTGAGTATATCCA
**CoV-bait-1825**	CTACAACAGAGGGGTATTTTCTACCTTGGACTATTGTCTCCACTGTTATAGTTGGTGAAGTGCGCATCTTTGAACACAATGTGCATGTTGGCTGCTTGCAGCCTTTAGCCAAGTGTCTTA
**CoV-bait-1826**	TCGAAGTGGCCCTGGATTTGTTCTTCTTAGGTGTATTTTTGCCGCTGCTGTCAGACCTTTCCTGTTTAGGCTTCTGCTGTTGCTTAAGCCTGTCAGGGTTTTCTCCAATACCCAAAGATT
**CoV-bait-1827**	AAGCATCAATAGCCAATGAAACATAACGTTCCATCATTAACGTACCATCAGTCTTGAGGAGATCATCTACAAAACAACCAGCACCTAAAATTCGAGATGGATCAGGATAAGGCAAATAAA
**CoV-bait-1828**	TACGTAAAGCGCACCGCCGCACAGAACCATTTCGCTCAAAACTTGAGCGCACTCATTAGCAAGTCGGTAAAACCGTTCACTCAAATTACAGCATGTAGAGTGTTTTCTTGCTAGAATAAG
**CoV-bait-1829**	TATAAGGGCCAGTAGTATGTATAGTGCGATTATAAACCCACTTATTACTATAGTCTTTAACACCTAAAACAATCACACCAATTCTATTTTCATTAACAATGCAACTTTTATGTCCTGTGC
**CoV-bait-1830**	TACTCATCACAACAATTTCCACATTTCTTAAAACAACAAGAGCCACAACCTGTGCAGCAACATATAAAAAATAACAAAACACAAACAGCTACACCAGCTAATCCAATTAGCAGCCAAACA
**CoV-bait-1831**	AGTGGCACTACGTGGACGCTCTCATTCCAGCATTTGTGAATGCGCCGGGCAAGTCGCACAAATCGTGTGAGGTTCATCGGGCAGGGTAACACGAATCCACCGTACCTTTATCCATTGCGC
**CoV-bait-1832**	GCTGTCATGATGGCCACGCATCACTTAAGGCCCGACTACCAATAACTGTCTTTGCCGTAGGTTAATTCTCCACGTCTCCTGGTTGCTTCATGCTAGGCACTCGGAGCGTCAGGGGCACGC
**CoV-bait-1833**	AAAACAGCGTTACGCCATTCTTTATTATGTTCTAAAAAAGTTTTGACAAAATCCAACTGTTTCCTATTAACACTAGAACTATTATCAACTTGTACATGACCCTTTAAAAACACTTTAAAA
**CoV-bait-1834**	TAAGTTGATTCAAGGCTTTGCCATGACTATTGACAACATCTTGAACTTTTGTCAGGGCTTGTGCTACAGTTGATAGTCCCTTAGCAGTTTGCTGAATAGCACTATTAACTTTACCAAAAG
**CoV-bait-1835**	CACCTGATTGCTTAAGGCCCTTAACCTTGGTGGACACAAGCCTAACAACAGCATTCTCAAGGAGAACGCAGTCACCAACACGGCTAAATGTCTCACCAGCAACCTTAAAGGTCTCAACGG

**CoV-bait-1836**	GTGTCCTGAGATGAATGACGGATCGAGGAGAAATGCGAACATTTCATTCAAAAACCAGTGGTTGTTGGGTTCAAGATGTCCAAGTTCGTAGAACAGGGCAGCTGAGAGGGCACGGCACAC
**CoV-bait-1837**	ACAATCTCAACTACGCTGGGAAGCTGTTGAGAGAAATTTGGAATGATAGGCTTTTCAGACGCCTTTCTGACACCCAGGTTAGTGGGTTCAGTCTTTGCGCCTTCTTTAGCAACCCAGAAA
**CoV-bait-1838**	CATCCTCGACGCCAAGCTTCAACACGCTAGCATTCACAACTGGTGCAGTGACAACACGCTTACTACAACAACAACCGTCATGCGTGACACGTTCAATAGTGACAGAACCAGCAGGAACAA
**CoV-bait-1839**	CATCTCAACCTGTGTGTCATCAAACACATCTGTGTATGCCTCTACAAGAGTTACAGACAATTCCTCAGGCTTCTTATCAGCAGACTTAGAACGAGATTTTCTTTGCCTCTGATCCTTAGC
**CoV-bait-1840**	ATCTATCACGACCAATGCACAAACCAGCTTTAGAGGCTGTGCACTCACCAACGCGACAATATGTAGTTTCTTGCGTCCTTACGGTACGCATACCAAAACCCCTTGTGAGAACTTCCGGTA
**CoV-bait-1841**	TGGTAGGCACAGAGATATAACCGGTGATAACAGGACTAACAGGCTCAGTAGCAGCAACGGTTCTAGTAGCATTGACAAGCTTACCATCAGCACAGATTCCAAGACCACCATAGGTTAAAG
**CoV-bait-1842**	CCAATTGTTTGGACTGGTACTTGGGAGATGGCTATGTGGTCATTTGACACTCTGGAACCACCAATTCGTACAACTGCTTGTTGTGAGATGTTGTTTCTGAGAAGTTGGATAATTGCATGT
**CoV-bait-1843**	ATTAGGTGACTGGACCTTTTATGGTATGTACCCTAGATGATCCTAGGATGTGAAAATCTCAGCTCTCGCATAGCCGCCCTTCCACCGCGTGCTTTACTCGATGGGAACGCACGATCCTTC
**CoV-bait-1844**	AACATATCTGTTGCTCTTTTGGAAAAATCTTCACCATAAAACTCAGCATCATTACCATCAAAGATGTCAACCCACTCACCAGTACATGTAAAAAATGGAGCAGGCCAAACAAATGGGTGC
**CoV-bait-1845**	TTACTTGGGTACTTATCACCCGTATGCGTGGAGCCGATAAGGAGCCTCCCTTATTGCTGCATTGCGGAATTGGTCAATAGCTATATCAGGGAGATCTTGGAGGGGGTCTAACCCGCACCA
**CoV-bait-1846**	TATAATCTATACGCTCGAAGACATACTCACCGAGCTGTACTTTACTATTCTTGGTTAAATGATAACCAGTAAAAACATAATTACGGTTGAGTGGTGGTTTGGATTTACCAACCTCCCACA
**CoV-bait-1847**	CTGCTTAAATGATAAAAGTGCTTGTACAAAGGGTGTCAACTGTGCCCCCAATGTCAATCTTCTAGACTCGGGGAGCCGGGTATATAGAATGGACAAAAACCAAGCAGTATAAACCTGAGC
**CoV-bait-1848**	ACAATGACATCCACAACTTTGGACACAACATTAAGATCTAAACTCTTAATGATAGTCACAAAATCGTCCAAGAGTATGTCCATATAAGTGCACACATTCTTAGAAGATGGATCATCAGCA
**CoV-bait-1849**	GAGGTGCAGCAGGAGTTGCCATAACAATGAGTTATTAAGATTCGTTAAAATCTCACTTACTATGCCCTAAGCAATGCAAGTTCTTCATCTTGTATAATAGTAGGTCTTCTATAATTACCA
**CoV-bait-1850**	CTCTTGAAGATGACTGACTATTACCCCCAGTCCCTTCAATGTGGAAGTTTTTAGGAATGAGAGTACCTGGCGCAAACTGGCAAACGATAGCAGCATCATTTGCTGGGTTGCGAGTCCCAA
**CoV-bait-1851**	TGCATTAATGCACTAGCAACTTGTAGTTGCATATTATCCAAGAGGTTATTAACCTCATTAAGAATGGCATTAACATTAACACAGAAAGAGCCATACTCAACCAACTGCTGCCTGCATGCA
**CoV-bait-1852**	GCTTGTCCTCGAGAGATGTTAAAGTACCTCGAGGTATAAGAATGGGAAGGATACATGCTCCACCTTCGTCCTGTGCACTTCCGTTTGTAGACTCCAATCTCCGGGTCATCAGGGCTCCGT
**CoV-bait-1853**	GTAGTAAAGTCCAAGACCAATAACGCAATGTGATTTTCCACTGCCAGGAGGGCCTTGAATAGTTGTAATTCTTTGTTTACCAATCATTTGGTAGTATGGTACCAGACTAGAATATGCTTC
**CoV-bait-1854**	GCGCAGCACAACGCATATCAGTTCGCGGGTGAAAAACACAATAAAAAACATGTAATATAGTACCACAGCCTGAAGGGATGATAGCTAAGGTGTGGTTAGTATATATAATAGAATTATTAT
**CoV-bait-1855**	GTGGCAACGTCTTTGTCCAACACACTCTTGGCTACATTCATAGCCTTCTTCAGAGCCTTAATGGTAGACGCAGGTGCGCCTGTAGTAACTGCAGTGTCATAGGCTTTTTGAGCATTTTCA
**CoV-bait-1856**	GCTCAAACTCAGTAAGTGATATTTTGGGGTTTAAAACCTGGACACCAGGAATTACTTTCGAGGTGACCAGATTATAATTACCCAAATACTTAAGGTCTTTTGGATGACCTTCTTCAGCTG
**CoV-bait-1857**	TGCGGGTTGTTGTAACTGTGATGGAGTATGGGTATCCATTAACGACTACGTTTAACCGTTGTTTGTGATTCGAAGTGATATTTTCTGTAGCATAATCCCAATACAATGCTTTAAGATTTT

**CoV-bait-1858**	ATGGATTGCCAGTAGAAATACTGAGGCCCACATCAGGTTAAACTGAAGTAGACTGGACAGAATGTCGACATTTCCTCCGTAGAAGGAATTGGATGAGGTGAGTTGGGAGAGGGCTCCGGA
**CoV-bait-1859**	GCAAGTCGCAAGACTATTAAAGTCTGTGACGTCATTGCTACCAGTACAAATATTCTTATACAGACCAAAAACCTGGCCATTCGCACACAGGGGGAATGATATCACCGGCTTGTGATCTTT
**CoV-bait-1860**	GAAAAGAAGGCTATGCATGGCAGAAACAATCTTTGACTTTCTGTCCACAGCTCGTGCTTCTTTATACATGCTAGCTGCAGCCTGTTCAGCCATTTTATCAAGCTTCTTTTGCACTGATGC
**CoV-bait-1861**	ACCACGAAGGATGTACTGAGGAAGCAATGGTCCAGGGAATCCATCCGAGACTTCCTGATGATACTGTACCACATCTGAGTTCCTGCCGCAACGATACGATCTGACCTCAAATTTGGCAGA
**CoV-bait-1862**	CAACTTCAAAAACAACGTAACTAATTTGGTGGTACACATTGCCATCCTCATAAGCGATGGCGTAAATACAAGAACCAAAATTAGGATAGGTAGGCACTAAGGTAACACAAAACATCTTAA
**CoV-bait-1863**	TGTTGAAGCATGCCAGTTATAGGACACCCAACATGTTTGTAACAATGTGTAACAAACACTGTAGTATCTGAAAAGTTACAGTATGCAGTACAAAACTGACTGCTAGACCAAGCCATACCT
**CoV-bait-1864**	CCAAGCCAGCACACAGCCCATGAAATCATCTGGCAGCTTATAATTGTAATCAGCAATCACGCCAGTCTGTCCTGGGGCGATCTGTCTCACATCATCTCCCTTCACCACAAAAGAATCGGC
**CoV-bait-1865**	CGCCTCGACTTTATCAAGTCGCGAAAGGATATCATTTAGCACACTTGAAATTGCACCAAAATTAGAGCTAAGTTGTTTAACAAGTGTGTTTAATGCTTGAGCATTCTGGTTAACAACGTC
**CoV-bait-1866**	ACATAGCAACAATAGCTCTGTTCATAACTTTAGTGAAAGCGGTGCTTCCCAATAGTACAACAATGACATTTTCAAAGGTTTTACCCTGAGATGAATCAATAGTTAATGCATTTGGTACAC
**CoV-bait-1867**	CTCTGCAGAACAGCAGAAGTGATTGATGTCTGTGGTGGTTGGTAGAGAACATCAGCACCTGAGTTGCTAAAGTCATTTAGAGCCTTTGCTAAGTGGCAGCAAGCTGCTTCACGATAGCTG
**CoV-bait-1868**	ACACCAGCAGCCTGTGAGGGAAAACCACACAGTGGTGTTAAAACTGATCTCTGTTGTCCAATGTTCCAAGCACCTTTTACGGGCTTTCCCTTGGTAACTTTATAGTTACCGCAGGACTCA
**CoV-bait-1869**	TGATGGCAACCTCTTCATTCAAATGAAAATCGCCAACAATGTTAATGTTAACACGTTCACGACTCAGTATCTCAAGGAGATCCTCATTCAAGGTCTCCACATTGTCACCAGTAATGCCAG
**CoV-bait-1870**	GCAGTACGTTTTTGGCGAGGCTTTTTAGATGCCTCAGCAGCAGATTTCTTAGTGACAGTTTGGCCTTGTTGTTGTTGGCCTTTACCAGAAACTTTGCTCTCAAGCTGGTTCAATCTGTCT
**CoV-bait-1871**	CACCCTTGTTACACCGTTTTCCATGAGCAAACTGAAACGTTTTCATCGCTCTGGAGTGAATACCACGACGATTTCCGGCAGTTTCTACACATATATTCGCAAGATGTGGCGTGTTACGGT
**CoV-bait-1872**	AATAAGTGCGTTGTTACAACGACACTTGAATTTAGGAGTGTCTAGGACTGAGTTATGTGATAGAGCCATAATTGTAGAGTTTCTCCAAAATATATAATTGGCATGCATTATATTTCCATC
**CoV-bait-1873**	ACACAGGACTCAGACGGCAAAACAACCGTATGCTCTACTTGCAACATTCTCATGTTAATAGCAGAAAGGGATAGTTGAGCACCATTGACTATGTAACCAAGTGGTATCATAATCAAAGGT
**CoV-bait-1874**	TGAGCTAACTCATTAGAGAGTCGATAGAACCTATCACTATGTGTACAACACCCAATGTGCTTAGAACCCAATACCATAGCTGAAGCCATTCGTATCATATTAGGCAAAGCACGGTCGCAC
**CoV-bait-1875**	TGCACCAAAAATGCGTGAGGGGTCTGGATATGGCAAATATCTTAGCTCTCCGTCAACCTCTACTAGCATTGTGTGCTGTGAACAAAATTCATGTGGGCCTTTTTCTAAATCTGGTTCCAC
**CoV-bait-1876**	ACACCCAAGGACCAGGGGCCATGACAGGGACTCAGCATTCTGTGCGGTTACCTCCTTAACGTGTACCACCTTGCCATCATTGTCCTTGATATCAATTATATTCCAAATGGTACCAGCGTA
**CoV-bait-1877**	GCAATTTTATAGTCTCTAACATCTGTCCAATCAGAGGTTGCCAGTGTGTTGAAAATTTCAACATCTGGAGAACCAGTGGCTGAAGCTTTGTAAAGACCAAAAACATTGCCAGCAGAACAA
**CoV-bait-1878**	TTGTCAGTGACCAAAGTACTGGCACTCTCAACTTGTAAAGTCGGCTGATCCTCAAAACCACCATACACGGCACCCTCAAAACTAGAACCAACATGACAACCACTACCCAACTCTATTTGG
**CoV-bait-1879**	AGGAACCACTTTAACAACCTTAGGAACCTCAATAGGCAAACGGTCAGTAACAACTTTGTCGTCAATGACGCAAGTACCTAATTGCTGCCCGTAGGTCTTTGTGGAATCAAGTTCCTTAAC
**CoV-bait-1880**	TGCATCACCAGATGATGTACCTCCCGGTTTGTTCAACAACATACCATCACAAATCATTGTATCAAGCATGTATGAATTAACTTCATTCAAATACAACCAAGACTCTTCCTCCCAACCACC

**CoV-bait-1881**	CCTTGACAAGATGCCAATGATGAAAAGAGCCCTCCTCTTGAACATGCTTTATAAACTCATCGACAGGAGAGAGACGCTCACCAGTGGATGTAAGAACTAGAGCTTGCTCTTTATTAGTAA
**CoV-bait-1882**	GCAAAAAGGCGTAGTACTCATCTACGAACTTATAATCAACATGGACACTACGATAAACATTCGCATACAGTTTACGCTGTAAGTCTCGAATGTAAACATCGACTATCTTGTTTCCATTAG
**CoV-bait-1883**	GCAAAGTCAAACACACATAGCTCTTTAACGTAAAGCTATACAAACTCGTCAGCCATGATGGGGCGCCACCAACAATCTTAGTGGCACTAAAACGAACTGACAATAAATTGTCATGCGCAC
**CoV-bait-1884**	GCTTGTGGTCTTTGTCCTGTAAGTACATTATAGACTTTATGTTGAGAAAAGTCTGTTTGTCATGGGTTACATCAAGATCGCGTTCGACGTCCCATGCATGCGAATATGTAATGCCATCAA
**CoV-bait-1885**	GTTTATTTTGGAACAAAACTGGCCATACTCGCGCAGTAATTGCTCACACTTCTGGAAACCATTGCAAACGTACTGTTTACAATCAACAGTAACTTTCTGAATGGTTGTCTGAATGTACTC
**CoV-bait-1886**	TCCACTTCTGGTAGTTGGGGTTCTTGGGATCAAGTTTTATGGCTCCACTGTATCTAAGAAAGTACACGGGGGAACCATCAGCATCATTGCTTTGGTGGGTTAGTTTAAACTGAGACATAC
**CoV-bait-1887**	CAGTTTGCCGTGGCGATGCAATTGCCAGGCAACCTGTTGTGCTGCATCCTGCTTTGCTAATGCCTTAGTGCTATTCACAGCCAGTCCAGTAAACCCGTCAAAGGTTAGCTGGCACTGCCA
**CoV-bait-1888**	TGCAGCCCAACCATCATCATCAGAATCTTCATGCTGCAAGCCAGCCTGTACAGCTGACTCAACAGTAGTATCAACGGTGACTTCTTCCTCGACACCATCAGCTTCTTCTTCAACATAATC
**CoV-bait-1889**	AAACTTGGATAAATCAAAAAGTGAGTAAGTACTCAGATTCATAGGAGTGGAATTTCTCCAAAATATATAGTTGGCGTGCATAGCACCACCATCTATATTTTCTTTAATAGTACCCAAGTA
**CoV-bait-1890**	TCACAATTAAGCATAAGTGGCCATGAAAGGGATTCGGCATTGTTGGCAACAACCTCCTTGACATGCACTACCTTACCATCATTGTCTTTGATATCGATTACATTCCAAATGGTACCAGCG
**CoV-bait-1891**	TAGTGGACCCAATAAAAACTACATTACCGGCAACATTCTCATCACAATCAACTGAGACTGGGCCATCAGTAGCAAGTAATAACAAATAAGGCAGCGAAGCTTCAACAACCTCGTCTACAC
**CoV-bait-1892**	TACTCTTTTTAAATACATTTATGGCTTCAAGCAATGCACCGCCAGTCACATTGTTAAGACACTTACCAACGCTACAAAAATCCAAATTGGAATTTGTAAACAACACCAGCTTGTCAGCAC
**CoV-bait-1893**	TGGAGTGTGGAATGCATGGTTGTTGACGTATAACGAACCCCCATTTACGCCTTCAAGGTTCAATGTAGAGCGCGTACGTGTATCAAACCTGCAAACAATTGAGAATTCAGGGTACATATC
**CoV-bait-1894**	ACTTCAAGTCTTCGTAGCATGCACGCTCATGTTCATAATTAGAGGGCGTTGTTTGCTTGACTACAAAATAGGAGTCAGCATACAATAGACCTCTTTTCCAAGATTTATCATCTTCAATCA
**CoV-bait-1895**	TGTGTCATCCTTACACCCCATTAGGGCCAAGCCTCGGGAAGCGTTGGAGGTGGCAACTTTAACTTGTGTGGATCGCAAGAACGTCCTCGTTAGACCTACAACGGGTACCTAGGGTCATGC
**CoV-bait-1896**	TTTCCTCACGAGAAGGATCCAAATAAGCATCAACCTGATCAAGGAAGACTTGGAGGTTCTTGTTTTCCTTAGGAACATTCATCTTATAGTGGAACTTAATTTCGACCTCGTCCTTAAAGT
**CoV-bait-1897**	GTACCTACCATCAGGAGTCGTTGTGACAACATAAACATCAGGACTCATGAGGTAACCAAAGTCATACTTCGGCCAAGCACTGCAAACAGTGCGAAAAGAAACACAAGCACGAGGAATCTG
**CoV-bait-1898**	TGGCATTGAGCTGCTCAATGGTTAGCTGTAATTTGTCAGCAGTAGCCTGAAGCTCCAATGACTTATTTTCAAGAGCATCAATCTCAGCACTGATATTAAGATATGTCTGGTTAAAAATAT
**CoV-bait-1899**	TCCAAATAAGCGCCACAATAAGCAATAAGTGGACAATACACCAAAATGAGCAAAATGCGTGCAATAAAGCATGCATAATTGCCCCTAATTGTAACAGCCTTAAATAAGGCAAAATACAAT
**CoV-bait-1900**	TGACAGTTCCACAGCACAGGGTGCAATGAAGTGCCAACTAAAGGTGTATACTTAGCTGTGGGTTTAGGGATATACAAACAAGTAGTATACGGTGAATTAAGGTACTTCTGCCAAATTTGA
**CoV-bait-1901**	ATTGACATCTTCGGTAAAGCCTGAAGCTGTTGACAAAATACATTATCCACAGCATCAAAGTAGTGCAGGCCATAACGATGCTGAATGCGAAAAAGGTCCAATTAACCCCGAAGCTAGCCC
**CoV-bait-1902**	GCAGATCCTGTGAGATCGTGTCATCATTCAACGGCCACTGAGAAACCATTACGGGAAGATTAGGATCGGTGCCACCTTCCTCATCATAGATGTAGTACTTAGGCACATCGATATGATCTT

**CoV-bait-1903**	GCAAAAGCGTCTGTTGAGAATGTTACCTTAGCGGAGTTACCATGTTGAGTTGCTGGAACGTAAAATGAACCGTCAGGTCTACTGGCGAGCATCCAACTTCCTGCATTGTCAATACCGTAA
**CoV-bait-1904**	GTAATATAACAGTAGTGTGTTTGAGAAACGTCACCAAATGGTATAACAGCTTCACCACCTAAAACACTACTATTAGTACAGCTAAAATTATACACATTATTACTGGTAAAAACAGAAATA
**CoV-bait-1905**	TTTAAAAGCACTTATGTCAGCAACATAACCTAAATCTGCATACTCTTTATTATAACAAACAACGCCATCATCAGATAATATCATCATAGAGAAGTGTTTACGCAAATAAGCATAATACTC
**CoV-bait-1906**	TGACTCCAATCCAGCGCATTAAGAGGTGTAACGTGCGTCTAGGCTCTTGGCCGGATTCAGAGTATTAGAATGGCAGTCTATGACATATACTGGAGCATCACAACACACGGGGCGTAGCCT
**CoV-bait-1907**	ACTAATGGAAAAGTTGACACTCTTAATAGGTGGTAGACTAAAATATTTGTAACCATTAACAAAGATGGAACCATCCCTACCAAAAGCTAACTCTCTTACAGTTGGTGGTAATATGCCTAA
**CoV-bait-1908**	TCTCACAATATGACAGCCCCACCAACTGGTCTAATGTTACAAATCTATAAACACTACTAAGAAGATTTGTCCAATTATATACCTTACCATGATTACAATCACCACTACAAGCACAGGGTG
**CoV-bait-1909**	CACACGGTTGCAGGCCATTAGGAACAGTTACTGGTTTCCACAAAGACAGAACTTTGCTTCCACACAAACACGAGACAGAACCTCGAGTAAGGCGTTTATAGAATGGAATCCCGTGACAGA
**CoV-bait-1910**	GTTAGCATGTTCCTCCTCTACAAAAGTAGCAGAAGCAACAAATCCATCCACACTCTGAACAGCGATTACTCGCCAAACAGAGACACCTTCAATGTTTGCAACAAACTTAAGAGTCATGCC
**CoV-bait-1911**	GGCAAGGTTGTTCTGACGTTTGTAATCTAAAGGCTTGCGCTTAGTAAGCCAAGCACAAACGTAAGTATGACCATTGATGATAATTTCTTCGTTTTCACCGAAATGGTCAACAAACTGCCA
**CoV-bait-1912**	CTCGTTACTATATTGTCTGCAAAAATCATCCCATTTGTCAACATAGTCTCTCACATAAAGTGGAACACACAGGCTGATGTTAGGTTTGACAATTATTTCATTGTCAATAACATCAACACT
**CoV-bait-1913**	CTTTATGGTGACAAGTTTTACAAGTGCATTGTCAAGCAAAACATAGTCAAAAACCTTGTCAAATGATTTACCTGCAATAGTACTGGTCTCAACAGCACAGTCAAAAACAGTTTGCACAGC
**CoV-bait-1914**	CAAGGTCTCTGGGGCCAAAACATTGTGTGACATTAGATGTCACATCATCATTAGGCTGTCTTTTCCACCGTGGCTTTTGCATTTCATGCTTTTGTTCTTTTGTTTCAGAACTCTGAGAAC
**CoV-bait-1915**	TAAGAAGCTCATCCGAACCTGCTTTGAATCCTGGGAGGCAGGCATGCGTACCATCCGAAGGATAGAAGGCATTGCCATCAGTAGCCATTTGCAGCACAGTGTTACCAACGACGACATCCA
**CoV-bait-1916**	GGAGGAGTAGTAAAGTCCTCCAATGTGGGTTTCATTTGTTTTAGTAATGACGCCTGTACCAGTTTTACCATAGATATTATACTTGGTACACACGTTAAGATAAACAGTGGATATGTCAGT
**CoV-bait-1917**	CAACTGCCTAATTGTACACATACGAGGTGCATTACAATTTAGACAAGTACCATAGGGAAACGCCTTCTTAGTAGGTGTACAAAACAAGACGGAACCCGATTCTTCAAGTCTACCACTGGT
**CoV-bait-1918**	TTTGAGCTTACGAGTTTGACAAGCGCATTATCAAGCAAAACATAATCGCCAACTTTTGCAAATGCCTTGCCAGCGATAGTGCACTTGTCAGCCGCACACTCAAACATCGTCCTAACGGCA
**CoV-bait-1919**	TCTACACCACAGACAAACAGACGCACCACCATATGAGTCTTGACTAGTATTAGCCTCAACACCATTAGTGATAGCCTGTCCGTTACCAGCACCATTAGCCAACATTTTAACACAGTTACC
**CoV-bait-1920**	GAATATGGTGACAAAGAACGTCTAAGAGTGGAAGAAATCCCAAACTGGGCCACTTGGGCACTGGCATAGTACATTATAAATAGGCCAATAGGACGAGGCGTTGGTCCATATATAAAGTTA
**CoV-bait-1921**	ACCACTAATAGAAGTAATGGTATGCAATAAAACCTGTTTGCAATTCTGACAACAACCATATGGAAACGGTTCCGTCGTAGGCTCAAACCTAAAAGCACAGCCAACAAAGGTCTTAGCGCC
**CoV-bait-1922**	ACAGTAAAAGCTATGGTCCAAAAAGTTGAACCAAAAACTTGTTGTGAAGGAAGCTCAACATCAACACTACTAAGATCACCAGCAGCAAAGTAGCGATACCCATTCATATAAACATCACCA
**CoV-bait-1923**	TGTAGCATAACATGTATACAATATGAAACAGGCCTTCAAAAACATACAAACCCACTTGGCTTTCAGCCTCATAAAGTAGCGTAATGCTCTAACATTATATTTAATGCTACGACTAATTAT
**CoV-bait-1924**	CACCCACCATATCAGTAAAGCCATTGCCATGAGCCCATTCATTGTAGGCCTCGACAGTAAGTCGCTCATTAACCAGGAACCAATTACAACCATTAAGCAGCGCACCATACAAAAATGCGA

**CoV-bait-1925**	TATAGTCAACAGTAGCTCATAAGAAATGGCTTTGTAGGAGCCTGATTTGAGATTAAAATACTTACATTCTAAATCAAGACCAACACTAGCACTCGATGTAGAATTAAAGCCATAAATAAC
**CoV-bait-1926**	TACAAAGACATCCAAAAGCTGTTTGAGGAAACAGTGTAGCAAATAAAATATTAAAATTAGCGCAATGAATCAAACACCTATCATCAATGCACTCTACACAATTAGGATGATAAGTACGGT
**CoV-bait-1927**	GCCGGCTAGCTGCATGCCCATTCTGCCGGATACCCAGCAGAGTTGGTGGCATGGGCCCACGGCCAAAGCCAGCGATCCAGTTGGCAGCAGCCTGGCCGGTAGCCCCAGGGCCATGGAATG
**CoV-bait-1928**	ATGTCGAACGTTCCTTAATTCATGCTCTTTATCGTAAGGTCCGGTGTAGTTTACATCTACCGAGCTGGGCGTATGCTCTCATGCCGGTGGGCCTGCCACATTGTTGGTGCGGGATGATTA
**CoV-bait-1929**	CCATAATAGACACAGCACAAATAACCAAGGCCCATATAACAAAGCATAATAGCTTTTATAGAACCCAAAATTTGTGCAAATGATGTATAATATTGAAGGTAATATGCCAATTTGTAAACA
**CoV-bait-1930**	CAGGGAAGACGTGTTGCGGAAAACCATTACAAGAACCACAAATAGTGTAATCTTTAACACGTCCATCAGCTGTGGTATTAACAACCCATGTAACACTGTCTTTAACAGGGTAGACCTGAC
**CoV-bait-1931**	AAATCCCATAAAGGAAATTACGTGTTCATACTTACAAACACCATTCACACCAACTTGCACTGCGAGATCACCACTCGTCTTAAAATTATCAGATAAAGACATAAACGTGGTGGCATGCGA
**CoV-bait-1932**	TCCACGCACTCTTCACAAAGCTGCACAAACAGCGCACTGTTTGCAATATAAGATGGTGCTCTAAATGTGGTCTCTTGTGTAACAATACACATGCCATCTTTAACCTTAACGTCGTAGAAA
**CoV-bait-1933**	CTAGCCATTGGGCACAGTTACAGGTTTCAGACAAGACGGAACTTGACTTCCACACACTTCACCAGACGGAACTGGCAAAGGGCGTTTGTAATGGATGAGAGGGGACGGAACCCTCACTCA
**CoV-bait-1934**	TTTAGTTTAGTTTGTTAATCAGATTGAACTTCAACTTCTTCACAGAGTCTAAGTTCAGAAAAGCACATGTTCACCACACCTACAACAGGCTCTTGTGCAAAGATGTAAAAGAATTGTCCA
**CoV-bait-1935**	TTTATAACTAAAAACAACACAACAATTTCGTCACCAAAAATTGAAAATGGGACAAGCTGAAGGGCCCAGACACTATCTTGGAAACCCAAAACCACACCAAATTGGTTGACATATTGCGCA
**CoV-bait-1936**	GAGCTGGCTTTGTACATAGCAAAGTAAATTACAAACACACTAGTCATGAGAAAATTGGCTAATGGCAATGCATGTTGAAAATGTACATCAGGTAATGGTAATTTTTCAACTAAATGCTGC
**CoV-bait-1937**	TATTTTAACAAATCATAAGTTTTAAAATCACTACCATAAATATCACTTTTCACATAACGTTCACACGCCAAACAATCAGTCATACTCATAATAGGCATCATATAACTAAGATAAGAAGTT
**CoV-bait-1938**	AGCAAGAAGTCGTAAAAATCCTTGTTAAAATGACCAGGCTTTACAGTCTGATTCGTAATACCAGTGCCCAGAGCGGCAACAGAAAAGCAGACTGTGCGCTGGTCAACAAGTGCAGGAGAT
**CoV-bait-1939**	CAAAGCCGCGTGAGATAATTTCAGGTAAAGACACAGCATTACCATCTACCATTTTATAGTAGCTATGAGGTGCCAACTCACTATAAAGTTTAGCACCTTCAACTAGACCATTCTTATAAC
**CoV-bait-1940**	GCCTCGTATCGTTGAAGCCACCTCAAATAGAGACCGAGAGCCAACATAGATTCAAAATCCATTTGGAACGACGGGTATCAAGACCTTCACTCTCAAAAGTACCTATAAACTTATCACCTT
**CoV-bait-1941**	ACAGGTCTTTGCCAAAACTATTCGACAACACACTAACAAAAGCACTATGTAAGCTTGCACCAAAATCAACAGACAAACTGGCCAACAACGCTGAGTCCACTAACTTAACAGGTTTACAAA
**CoV-bait-1942**	GTGAAACAACAAATAAGCCATTCAGCAGCTGGTATAAAAGCCAACCTGCTGCACATGCAGTTGTCTCAACAAAAGCATCAAACCACTCAATAAGCTTTTTGACAAGACCGGCACCCAATA
**CoV-bait-1943**	ACTGTAGCACCACGTGTGTTTACAATGGACTTCAAATGCTTCTGATGATACTGCCTAGTAGTCATAGTTGACAATAATGAAACACCACCAACAGTCCTAGCTCTTTCCTTGCCACTAATG
**CoV-bait-1944**	TAATTCTGTGGAATCATACCTGTTAAAGGACATGAACCAGAACTACTTTTATAACAATGTGTAACAAATACTACAAAATCAGTAAAATTGCAGTGAGCCGTACAAAATTCTGAAGTTGAC
**CoV-bait-1945**	TGTAGTGGTTGTCGGCAGCCCAGCTGTTAAAATTAGTAACGCAGACACTGTCCTTAGAACACCACCAGCGCTCACCATTAAGAAGTGCAGCATAAAAGAAAGCAATAACATTTTCAGTAA
**CoV-bait-1946**	ACTGCTGCTTTGCGGATTTATATAGGTCAGTTGTGTAGCAGGCGGTACAATAACTATCTGTCCATCCACGGTAGTGTTATTACCATATATCTTATCGACGAAAGGCGGGCTCGGTTTTTA

**CoV-bait-1947**	TTAAATTATAAAAAAGCTGGCCATTTTTCATAGCAGAAACACGTATAGAATGCTGTTGAAGCATGCCAGTTATAGGACACCCACCATGTTTATAACAATGTGTAACAAACACTGTAGTAT
**CoV-bait-1948**	AAGCAGCGGTCTTGAATTCTCTACAACGGTGCCGTCCCATCAGCCAGCGTGAGCTGGTTACTATCTAGGAACCCGTCAGCGGCTTGATCAGACCAATCTTCGCTTAGTTATATTTAACCG
**CoV-bait-1949**	CTGGACTATCAGCAAGTAGAGCATAGATAAAACCTTTACCTTGCTCATTACAATAAAGAGCTTTTCCTGTACCACAATCAGTGTTAACCACACGTTGTGAAAGCTTACCGGGTGTAATTT
**CoV-bait-1950**	TCCTCGAGTTCCGCATCATTCTTGCTTCGGCTTCAAGGTGTCACAGGAGGATTGCACTAAATTTATAGAGGTCCAGTTCCAGCTAGGGCTCTTGTGTGACACTGCCTGGCGCGACTCCGT
**CoV-bait-1951**	GAAAAGTGAAATTGTGTAACTTAAGAGTAGTATTAATACTATTTTCACGATAGACAATAAACTTCTGCTTAACTAAACTACTATTAGTAAAAGGGTAAAAGCCATCTGAAAAATTGCCAG
**CoV-bait-1952**	GCCACCACTGCTATCAGTGACAATAGTCTCAACCTCTTCAAAAGTAGGTTGATCAATTGACTGTACAAGCTCCTCAATTTGAGGAACCTGCGGTTTATCATCAGCCCCCTCTATAGTTGC
**CoV-bait-1953**	CATTAGGGGCATTTTGTGGTATGGTTAGAACATGTCTTCCATTACCACAAAAGGAGTACCTAGTAGACTGTGATTTAACACACTCATTAATCTTTTGCGTGGCTAACTCACGCTGTTGTG
**CoV-bait-1954**	CACGGCTCTTGTACGCCAAAGGGCAGAGAACAGCTATCTATGACGACAGGATAGTAGTCTTTAGCCCCACTGGAGGGGGTGACTACGCGGGGACCCGCAGCGCATATCGATTTGTCCTGG
**CoV-bait-1955**	CAAAATTGCCAATAAAAGTACCATCCTCAAACAAATGTGTGGAAACTTTCAACTTAAGTATATTAGGAATAAGTTCCAATACTAGAACACTAAAATACAAAAGCATAAGCCACCACGGTG
**CoV-bait-1956**	TCACGTAATTGATCACACGAACAACCATAACCTTTCCACATACCACATACCGTACATACCGTATTACGTAACGTAAAACCTACCGGATCATTCGCACACGTCGTCGGTATTTGTACATAT
**CoV-bait-1957**	GGCCTTAGCATTTACCCGCGAGCACCTGTCACGATGGGACATCAAATATCCCCGACGGGCCTAGGCGTGCTGAAGACCTGATAGATGTATGTCCCCTCCCATACCGTGCTTATACAGGGA
**CoV-bait-1958**	ATAGTCCCAAAGTGTGAATCCGTTACATATGTCAACACCTATGTTACGGAGTAATTTCACTTCAGGAACTGGATTTACATTACGCTTCGCGTAAAGCTCAAATGCAACGTTAGTAGGCAA
**CoV-bait-1959**	CAAACATGGGGCAAGAGTTGCGCCTGGTATCACGATCGACTCACAGACCGATACGACCGACAACCCACACTGCGATTCGGTCTTTAACGTGGCATTTGTGCACTAACTATGGCGCAGCAT
**CoV-bait-1960**	AGCTTAAATTTTGCTCGCGCACTACTCAGCGACCTCCAACACACAAGCAGGGAGCAGCTTTCTCAACGTAACACTTTACAGCGGCGCGTCATTTGATATGATGCGCCCCGCTTCCCGATA
**CoV-bait-1961**	AGGGTACTTAGCTACACTAACTGTTAAATTATAAAAAAACTGGCCATTTTTCATAGCAGAAACACGTATAGAATGCTGTTGAAGCATGCCAGTTAAAGGACACCCACCATGTTTATAACA
**CoV-bait-1962**	TCCTCGCTAACTGACGAAACAGTAGACTTAGGTTCCTCTATAACCACAACCAACGCTGGTGCACTATCAGCAGCGCTTTCAGCTTCAGTGACTGCGTCTTCCTCTTCACAATCGTCTTCA
**CoV-bait-1963**	CTTTATAACAAACAGCACCGACGAACCAGTTGCCAGTCACACTAGCAAATAGTGTCATAGCACAACTAAGAATGTCACTAGCATAAAAATAGTTATATGCCGCAGTCAGCAAAGCTACCA
**CoV-bait-1964**	TATGGGCGCTGACCAGTATAAGCTGTAACTGTGTAGCTAAACTTTGAAATTGATGACTTCATATACGCATCATCTAACAAAACAGATGTATTTAACATACCATTAGAATAACTAAGAATA
**CoV-bait-1965**	GAGGGTTTGGAGGTGTACTTGGAAGTAGAAGATGCCGTATTGTATCCAGAGACCGTTCATGAGTTGTGTGTTTGGTTCCGTTGAGCTGAAGCCGAAGCCAATTCCTTTAACTGTGAAAAA
**CoV-bait-1966**	AACATTTCCACATCAGGGGAACCCGTGGCAGAATTTTTGTATAAACCAAAAACATTGCCACCGGAACACAGAGGGAAAGCCAAGGTAGGTTTATGATCAACACAGTAATAACTAAGACCG
**CoV-bait-1967**	AGGCTCGGTATAACATCACCAACTTTCTTAGCCACTACAGCTACAGTAATGGGACAATGTCTTGAAGTGGTTGTCATTTTGAAACCATTACGAGACAGCCATGACACAAAACTACTATGC
**CoV-bait-1968**	GCACCAGTTTATCTATAACTCGGAGTATTGAGTTACCCTGCTTCATTGTCGCTCTGCCTTGGTCGCATGTAAAAATACATCAGTGGGCTAAACGAGAACCCCTCTTCCGATTCGTCTCTG
**CoV-bait-1969**	ATAGTTTGCATAGCACTGACAATTTTTGCTTTCTTGTCTTCAGCACGTGCTTGCTTATACATAGAAGTCATAGCCTGATCAGCCATACGTTCTAACTTACGGGCCACTGCCTTATCCTTC

**CoV-bait-1970**	AGTGGAGATGGCAATATTACGCACACCACCGAGGCCCAGCAAGCGTAAGTTAGCCAAAAGAACCTCAAACGCATTGCGCGGAGGGGACATCTTAACGGCCATCATATATTTGAGCTGCTC
**CoV-bait-1971**	CCACACATCTAGCAAATCCAAACAATTTGGATTCTCTAAGACTTCATGGTTCTGTGTTTTCTTACTGCTAACCACTACAACACTAGGGTCATCATTAAGAATTGATTCCTTGGGAATATC
**CoV-bait-1972**	TCATATTAGATTGTACAGTGGACACCTTATAAATAGGTATACCACCTATACCAGCTAATTTAACATTAAGCATAAGCGCCTGCCATGACGTAGTTGGCGGCAATAGGCCATGTGAATTCA
**CoV-bait-1973**	ATCACGCATTAAATTTTTAAGCATATTGTCCCAACCACCATAAAACTTGGTTGAACCAATGACCACAGTAGCATTGCGTGTTGCAGCAATTGACTTCAAATGCTTCTGATGATATTGTCT
**CoV-bait-1974**	AATTTCTTTTAGCACTTCACAACTATTTTCATCGAAGTGTCTAAAAGCGTAAACAAGATCAGCCATAGTGTACTTTGTAAGTCGCTGACGCGTGATGTGAGGCTCCATTCGTCCCTCCAC
**CoV-bait-1975**	GGGCACCAGATGTTAAATCCTGCCTGTACAAACATATTGTATTCTTCAACATACTTACGGTACAATGCAGCATGTTTTTTGCAGACTGCACCTCCTATGTTACACTTAGTGATACAAACG
**CoV-bait-1976**	ATAAGCATTAAGTGCTGCCAATCTACCAGTAATGAGGCGGTCAACATGAGCATCAGCTTCCACTTTTTCCAGTCTATTGTAGATTTCAGCTATAGAACTACTAATAGCCTGGAAGTTTTT
**CoV-bait-1977**	ATTATTGGCCCGCGAGTAAAGAATGCTGTCATTCCTCGTAGGTTTGTGTAGGAAGCGATGTGCACCCGGGAAGGTGAGCAAATCGAGTAGATCATCAGCAAACTTAGCTCGAGCGAGACA
**CoV-bait-1978**	AGGCTCCTAATCTGTAATAAGATAGAGTTCGTGATGTAGCAACAGTGATTTCTTTAGGGAGGTCCTTAATGTCACAGCGTCCAAGGGAGTGTCCAGCCATACGGAGGTGACCACGAATGA
**CoV-bait-1979**	GTTGTCTCAAAACTGAAACTACCTCGTCATGCAAACTCTGTAGACCTTCCACCTCACACACATAATGTCTACCCATCATGTGTGGATTCGTAATTGTAATATAAGTTGTTGGTAACTCTA
**CoV-bait-1980**	AGTTGTGTTCAACTCCTCAATGCGGGTTTTAACAGGCTCTTCCGAAGACTTAAGCTTCTGATTGGTCTCTTCATGCAACTCCTTAGTTTCTGAAAACTCAACAACAGGCTTCTGGTTGAC
**CoV-bait-1981**	AGGCTGGGTCTACAGTACCAGTGCGATAACAATTGTCATACAGTGAGCGTTGCAAACCTTTAACAATTAGGTTATTGCAAGTATTACTATTAACGCCCAAGATGCGATTAATGTTAGCAC
**CoV-bait-1982**	TGTCATTAACAAGACAAGGTCCAATTTGTTGTCCATAAGTAACAGTGGATTCTACTTTAACAGTCTTAACACTACGTGTATCCTCAGTAACTTTAATAACCACACTCTTATTTGCGTAGA
**CoV-bait-1983**	ACCGCCAGTTGCTTTATCTAGTGCTCGTGCAACACCACCACCATGTTTCAAGTGTATGTTAGCAGCATTCACTACAACTGTTGGGTTGACTTTCAGAGACTCTTCAACAATATCAGCACA
**CoV-bait-1984**	TAAATGGACTATATTGATCCAGAAGTGACTGTGATGTACTGTTGGTTAACTGCGTCTTGGCGTTCAACAGCGCGCCTGAAGTGTTAACTCTAACTGCTATAGTGTGAAACCCTATAATGT
**CoV-bait-1985**	ATAATTAGCATTAACTACAAGACAGTTGGTAACAGGTGCTTTATAATTCAAAGGTGTTATACCAGAACCATCAACAGCCTTACCATGAACAAAGTCATTGGTTCTATAATGGCCGCCATT
**CoV-bait-1986**	GGAAGTTATTTTGCAGTTGCTCAGTGAGTTGAGTGAGTGCCATACCTTGTGTATTGACAACGTCTTGCACCTTATTCAGCGCATTAGCAACAGTCATTAGGCCTTGTGACGTTTGTTCAA
**CoV-bait-1987**	CCTTGGATGGGTGCATGATTCATGACAAATAGTTTGCCAGAAATATATGTTACTTCATCTTGAAGGGTGAGGAATGTTTTTCTTTCTGTGCGAATGAGGAGTTGAGGTGTTTGGCCTGTG
**CoV-bait-1988**	AGATGTAAGCCACCTAACGTAGTATGTGAAAAATCACCATAAACTACGTGTTCAAAAGCATAGTCTTCCAAGCCATACTTCTTAATAAAAACATCGCTATCCAGCACAAGAAAGTCTTCC
**CoV-bait-1989**	TGCATTACCATTGCAGACATATGTTTGACAGTCCACAACAGTATGCTGTGCTTGTATTTGCAAGTATTCAGCCTGAATTGAAATTGTAAAATTCAGTGGTATTGTAATATTACCACTGGT
**CoV-bait-1990**	AACATTCTGAAAAGTACGCACAACAACGCGCGCATAATCTGGAATGCTCATAATAAAATATATATCACTTTCAACCACTATATATGCGCCTCGCAAAACATGAAGCAAAACCCATGCAAA
**CoV-bait-1991**	CAGCAGAAGTCTTAACCCGCAATTTGCGAAATGTCTGCTGAACTTTTGCCCAAGACTCAGTTTGGAAAACTTTGTATAAACCTGAAACATCAGGATCATCAAAGCATTTCAAGTAGTCTT
**CoV-bait-1992**	CCACTCTTAGGCGCTATACCTCTATCACCAGCAATGCACAGACCGGGACTAACCTTCGCTGTGACATATTTAGTAGGGACATAGCTAAAGTGGATAAAATACAAACCATATGGAGCATTC

**CoV-bait-1993**	CATTTTCACTGTAAGGAAATTCATCAGTGAAAGAAACACAAACACCAGGGTTAGTTTTAAAACAGTGTCCCATACGACAGTAATAATTACTAGTGTATCGAACAATGTGAGGGTATAAAA
**CoV-bait-1994**	GCGTTGTTGAAGGCATTAGCAATCAGTTTCTGGTTCTGGTTCAAAACATCCATAGTGACACCAAGACCATTAATTCTATATTGAACACTCATAGTGAATGGGACTCCTGCTGCCGCTGAA
**CoV-bait-1995**	AGAGCTCTATCACACTTCGGATAGTCCCACCCCATTAGATGAGGATTTTCAACACCATGTATAAGAGTCTTAAGCATGGCATCCCAACCACCATAAAACTTAGTTGTGCCAATGACAACA
**CoV-bait-1996**	TAACATCCTTTGTAACCAAAGTAACACGTCCTATATCAGGAGGACATGCCTTTTTAAAAGCATCCAAAGAATGAACAGGGTTACAACCATAAATACCTGCACCTAGTAATGGTATTACAT
**CoV-bait-1997**	AAAAGGTCCACTGTCAGTCAAAGCAAAAGATGCCTCACCTGGTTTAGTATCTCTAATAAAAAATCCATTACCAGTAAATGATACATTGTCATGACTTACAGCAGAAGTACAATGCCATTT
**CoV-bait-1998**	GTTATTAAATACCAATGCACCAGCATTTGATGTAACAACATGTGTTGGTAAATCATATTGTAAGCTTGTTTGATGGAATGGATTGACTATATAACCAGGTTGTACCTTAAAAACAGAACC
**CoV-bait-1999**	GCCCTACATATGAAATAACATCATATGTAAAAATTGACTGGGATGCTCTCATCATGGCATCAGAACCCAGAACAGTCCCAAACAGTTTACCGTTTGGGTAACTCCAACCTAAAGGAGATG
**CoV-bait-2000**	AGGCGGTATTATCCATGCGTACGGAATTTCCAGCCCTTTGGAAGGTTCACGGTTACGTAAGACTTAGCTAGGCAATTCCCAGGCTCAGCAAGATATACTGGTCCCCGTTGATGACCGTCG
**CoV-bait-2001**	TTACCCCTACAGTGTATTTTAAAACACTGTTTGCTGTCAGGATGTACTGGGACAAATTGGTTTTCATACACCATATCAGAAACTGTTTTCACAATTTCGGCTGGACAACGATAACACTTG
**CoV-bait-2002**	CGGTACCACCAATACCTGTAAGAGTTGTACAAGCTGCATTGAAAATACATGCTCCACCACTAGCTAAACCTCTTTCGTCAAAGCACAATCCTGATGATCCAAAGATAGTGTTCATGGCAA
**CoV-bait-2003**	GAGTATTGACATTAGGTTGGTAATTGGGTGTATTAAATGAAGTCCACTTACAAATTCTTACAATGGCACGACCCTCAGAGTTTTTCTGGAAAAAGTACATGCCCCAAGAAGAATTTGAAA
**CoV-bait-2004**	ACGTACCAGGTGCTACTCCTTTATCTGACCCAGCTCCAAAATGCATAACCCTCATATTATGGGGTACACACATAGTGGTTTTACTAAGATACTGGCACAACTGAGTGTATTTTGCTACAT
**CoV-bait-2005**	CATGATTGGATCTTCAACGGCACTAGTACCCCTCGGATTGTTACCGGATATATCTGACTTGCGGTTAAGTTCCCGACATGTGATTTACTTGTCTCACGCAAGGCAGAATAGAAGCTGTTA
**CoV-bait-2006**	AAATCCAACCTCTGACAACATTAGATTTGTCAATGGTTGAAAAGTAAACACCATGCCCAAAAGGCAAGACAGGTGAAGAATAAGCCTGGTTCGATGAATTGTACCACGTAAGAGTGGTGT
**CoV-bait-2007**	TTTTGCCTCTTTACCTCAGCCCACGATCCATATTGACCAGGCACTATCATAACTATGGCAATAAGGTCATTTTGAAAAGTAGTGCCAAATGCCACGCCAAAGGGCTCTTGGCAATACATG
**CoV-bait-2008**	AGGCAACATAAAGAGAATAAATAACATGTTTAGATTAAATTAAGCTTCATGAAGCCTAACACCATTATCAACTAAAAAATAAAACATCAAAAAGACTATAATAAGCACTGCTATACCTAA
**CoV-bait-2009**	ACTAAAATCTTCAGGTATATAACCTTGTGATGAGGGCTTAAAAACATTTTTATTATAGCCTGCACAATAACTATTGCTATCACGATTACACTCAATAAGCTGAAGTGTGTTAACTGCATC
**CoV-bait-2010**	TACTTGGTGACAATTGTGCAAGGTTGAAAAGACGAAGAGCGGTAATCTCAGCGCGTCGTGGTGACCCGGGGATGTGTTGTTAGGCGACAGCCGGAAGCCGATAACCTTTACGTATTCTTG
**CoV-bait-2011**	GCAATGGAGCGGTTGTACTTTTCAAATGCATCTTTGAGGGCTGCCAGAATAGGTTGTGTTGTCTGACAGTAGCTTGAGAATTGTGTACGGCATGTTGGATCTTTGTCACAGATGAATTCT
**CoV-bait-2012**	ATTAACATATCCCCTTAAGTCATCGGACACATAGAGGTGCCTCGCACACGAGATAAATAGTAGGATTAGTCTACGTAGACATGGGTTCCAATCCTACCCATCGCGCTCTGCAAGAGCGAG
**CoV-bait-2013**	GGTTCTGTCTTATCCACTTCATCTACAAATACACATGCACCCAAAATGCGTGATGGGTCTGGATATGGCAAGTATCTTGGCTCGCCATCAACCTCTACCAGCATTGTGTGCTGTGAACAA
**CoV-bait-2014**	GCACTACACAGTGAAAGGATACGCAAGGAAATTCTAGTAATCAAGAGCCCGCTTTGATCAGCAACACAGTTTCGCGCCTATACATTAGCTCCCCGTCTTACGACACTTCTCTAAGTAGTT
**CoV-bait-2015**	AAGGATTCCTGAGTGACAACAATGTCCCCTGGTCGAGAGACACCTATTGTTACTGTTGTATAATCATCAACAACACAACCATTGTCCCAATCTACCACAGCATCATTCAAGAGTGTCTGT
**CoV-bait-2016**	CTGAAGGAAGTCATAGCACAACTCACAAAAAGAAAGTGTGCAAGGCACTCCTTCAACCCAGTGTAGCCGGAGATTACAATCATAGATAGGGGTGTGAAAAATGAGCGACAGTTGTCTAAG

**CoV-bait-2017**	GGGTGTAACCACATGAGCCATAAGAACTAAAACATCATTAGGCTGCAATTTATAAGTAGATGTACCCCGATAAATATAACTGTCACCATCACAGCGTTGTAGGACATAGTCACCTACCTG
**CoV-bait-2018**	ACTGTCTGCTAGCACGAACCTGAGTCATTCTCGTCAGTGTTTGAGTGACAAAGGCATTAAGTGCTGCCAACCTGCCTGTTATAAGACGATCAACTTGAGCGTCAGCATGAAGCTCATCCA
**CoV-bait-2019**	CAGTGGCAACAGTGCGAATAGCATCACTAGTCTGCTGTAAAGCATTATTGACTTCACTAAAAGCATTGGTAATATTACCCATGGCTGCGTTAAAAGAGGCGGCAAGCTGTTGTTGATTCT
**CoV-bait-2020**	CGATTTACATTACACGAATGCGCCGTTTCCGTCGTTTGCGTAAATATTACATAATCATATTCCGAACCTTGCGACGAATCTACCGTTTGCGTCGGTAAACCTAATATTTTCGACGCTACC
**CoV-bait-2021**	TTCCTTCTTCGGATAGCATGTTGAAATCTTTTGCGAGCCAAACAATTGGTACATTTTCTTTAACTAGTACATTATGATTTACTACTTTAGAACCACCACGCATACACGCAGCGATATCAT
**CoV-bait-2022**	ATCGTAAACTTTACGTTGTATGGATTTTACTGTAACGTTATTACAAGCGTTTGAATCAACTCCCAAAAGCTTGTTAACATTAGCAGAAACAGCCTGAAAAATGTTAAAAACAGAGTTAGC
**CoV-bait-2023**	CTATTCCCGGGCTTTTATCGAGTGGGCCTAGGCTGCTGACTGAGACGGTAGGGTGTGTAGGGCCGCTGAGATCGGACATCTACAGCAGCCGACGGCCACTCTTGCCCCGAATAGTCAAAC
**CoV-bait-2024**	AACAACAACGACACTGCCGCTGGGTTGTGGTTCAGCATACTCAGTAGGCTCAAGTTCGATATAAGAAACCTCAACTTGCTTAGGTGTGATTGGAACTGTGCAGCCAAGGAACGCTTTGAA
**CoV-bait-2025**	TTGTAAACATAAGCAAAATAGAAAGACCTACTATAGAAGCAAATAGTGGTGTAACATAACCTGGGTTAATCCAGAAAAACTTTGTATAACTGACCAATTCAGACCAGAACAAAGTAAGAA
**CoV-bait-2026**	TTGGATTTAGAATTACCAACACCAACAGCAACTTCTCGACCATTTTTATAATAACTAATAAATACAGCCTTAGCATTGAAGGTGATATTAGCAGTTCCCGGTGAATTAATGGTGCAATTC
**CoV-bait-2027**	TTGAAGCCTCCCCTGGGTCCGAAGCAAGCTGCTACGCTATTTTCGCCCTTGGGAATTCTCCTCCACTCTGGGATGTCTTTGAGGTCACGTTCTTTCGAAGTGGCTCTGGATTTGTTCTTC
**CoV-bait-2028**	GTCGTAGTAGACTTGTAAACGACAGCATCACTATCATAGTCAAGTTTCTCAAATGTAAACTCACCCAACTGCATCTTAGAATCCTTAGTAATCTGAAAACCAGTGAAAACAGAGTTGCGA
**CoV-bait-2029**	TAGGATTTCCATCAAGAGGAAGAATATAACTTCTACCCAATGCATTAACACAAAGAATTGCATTAGTTTCAGGATTAAAAGACCACCATGATTTGGTCCTTCTATATAGCTGAATAGATC
**CoV-bait-2030**	GCGTTGGTGTCCTAAGTAAGGGAACTATATGACAGGTAGGTAATTATGCTATGAACAGTCGCCCAGAGGAGGGTGTCCTGCTGGATCGACCAACCAATGCATGGGATGACCTTTTCTTGT
**CoV-bait-2031**	TTAGGATAATGCTGTACAAATCCTGCGTAGCCATTGGCATTCAGGGTGTGTCTATCAGCACCAAAAGAATAGTCACAAACTTCATTAGCTACCAAATCCGGGTTAATCAAACCAGAATCT
**CoV-bait-2032**	CAAAAACAACAATTGTACTAAAATTGCAATGTGTTGAACACACTCCATTACTAGATGACCAATGCATGCCATTAACAGGTGTAGTAATCGCAACTGCACTTGCAGTAACATTTAAACTAT
**CoV-bait-2033**	CTTAACGAACTTGCCAATACTAGCAACCTCTTTATTGTAAATGTCAAAGGCGCGTACAACATGCTCTGGTTCAGTACCATTACAGGGCTCTAGTCGAGATGCACTAGAGCCCCTTGCTCG
**CoV-bait-2034**	CACAGAGGAAAGCTCATAATTGGTCAACATAGAAACCTCATCTACAACTACAATGTCACACTTAATGTCAGGTAAGGCATTGATAGTCGAGAAGATATACTGTGCAGTTGTGTTATTTAC
**CoV-bait-2035**	AGTCCAGCATAGATCAGCATATAAAGAGCAACAGTGTAACCATACAGTCGCTGAGCAATTGGGGAACCGAATATAGTTTCGTAGCAGGTGCGCAGAAATGTGTTCGGTATCCAAAAAGTG
**CoV-bait-2036**	ATACTCATTATATAACATACTATACTCTATCATCTTAAAAAATGGTCCAATTTCCTCAAAAACCTCACATTTTTCTACAAAAGAAAAGAACTCTACCACATTTTCAAAAATGGTAAAACC
**CoV-bait-2037**	AACGGATCATATACCGTATTATTTATTATACCTATTACTACATCACAATTACCCGATACAAACGTATTATCCGTCGTTATTATTTGCGGCGAAAAAAAATTACGTTGCGTTATAAACCAC
**CoV-bait-2038**	ACTAGTATAGCAATAATATATTGTCCAGGGATGGTAACGGCCCAGTCGTCGACTACCATTCCTTAAACTTAAAGGACGGTGTTGGTTGGTCGTCGGTATACCTTTTCTTTTTAGAGAAAA

**CoV-bait-2039**	ACAAAAGTAGCTTTAGCTGCGTCTAAGAAGGAAGAGAATTTCCCAGCTGTCTTAGCTACAGTATGAACATAGGTGTAGAGAAAGTAGATAATGCTACACACAATGAGCACAAGCAATTGC
**CoV-bait-2040**	CTGAAACGATTTACCCACGTCCGAGAAGAGGACGTGCGTAAACGTCACACAGGCTATAACTGTAAATGGCCTTTCAGCTTGCGTGATGTTAACGACATCACCCTGTGGGCATGAAATAAG
**CoV-bait-2041**	AAGAACAGTGCAAACGTTCTTGGAGCTTGCGTTAGGTGAGGTAACAACACAGTTCTGGACTGGGTTGTAAACATGGTTGACAAGCTGATGTTCAAACTTGTTTTTAACTAGTGAGATAAG
**CoV-bait-2042**	TACGGAAATTCGTCCGTAAATGAAATGCAAATACCAGGGTTAGTATTAAAACAATGCCCCATACGGCAATAGGAATTGCTAGTATAGCGCACAATATGTGGATAGAACATTATCTGCTCC
**CoV-bait-2043**	CTCTCGGGTAAGAACCCTTATTAAAATCTGTGTCTACATAACTAAAATCACTCAAAAATGAGAAATTAAAGTTATTGTAGCCAGCAACAGCAGAGTATTGTTGATAATATACATAATTCG
**CoV-bait-2044**	TTAAGAGTTTAACATATACAACATCACCACTATTATCTCTAGTTATGGTAAACTCATTAACAAACATGTAGAACGGTGATTGGATATCAAACCTGTAATCCACATGGTCCTTAGCCTGAT
**CoV-bait-2045**	CTATTAGCAACAAGACTGTTACTATCATGATCAAGCAGTGCTAATTTACACTTGTCGAAGGTTGTTGTAGCAACAACGTCATTGGCATACGTAGCCATGACACGCCACACAGAAACACCT
**CoV-bait-2046**	TGAGATAGAATAGCACAATAAAGAATAAAACCCTAATAAACAGTCTAAGAAGGCGCACTTCAACAATGGCACGCTGCAAACTATAAAGATCTAGACAGTCAGGTTTTAACAATTGAAAAT
**CoV-bait-2047**	GACCACCACATCAACAACTTTGGAAACAACACTCAAATCCAAACCCTTGACTATTGTCACAAAGTCATCTAACAGTATGTCCATGTATGTACACAAACTCTTAGAACTAGGATCATCAGC
**CoV-bait-2048**	CCACTAAATTCATCCAAAGTGACTGACCCACTAGCACACAGTAAAGGTTCTAATTTATGAAGTGCAAGCTCCACATCATTAGGTTGACCTTTCGTAGTGCCCGTGATAAAATACAAGAAG
**CoV-bait-2049**	CCAATTGCTTTAGGTGAAACTGGGTAAAATCCATCTGGTAAGTTGGGTGTAAGCTGATTACACCTAATATCATCAATGTGACTATTACAATAAACTACTCTTTTTATGGCTGTATTTTCA
**CoV-bait-2050**	TAACCTTCGTGCAGTGTTAGTTAGCCATAGGGTGATTCTCTGCACGAGTAGACATACGCGATCTACTAGTATTTCGGTACCGTATGCCTGCTCGTCTGTGTCAAATGCTGTAGTATGATG
**CoV-bait-2051**	GACTTTTAAGCCATCTGTTGAAGAGCCAGTTATAGCATGTAAAGGTATTACATCACTCTTTGCTAATTCAAACAAAGCTTTTACTTTATCTGAGTCAACACGACGTAACATGTGATAAAG
**CoV-bait-2052**	TTCTGCTGTCTTAGATGCTAATACTTTATTGTGTAACTGTGTAATTTTCTTACACATTTCAGTTTTATTAGTTACGCCTACTTTTTCCAACAGTTGCGCAATAATTACAGCTGTTGCCTT
**CoV-bait-2053**	ACTAACCGTGTTGCAGATGCAGCTGGAATTATACTAAGTGGTAAACAACCATTACGTGCTTGGTCGATCAACGTGTTAACAGATGACATGTCCAGTCGCTTAAGCATACTAAAAAGTAGA
**CoV-bait-2054**	TGTAACAGCCTTTTTAGGTTTGTTACTAGTTTGCTTTATAAGTATTGGTTTGAGACCACCTACCTTAAAAGCATGTGATATGATGGAATTATCTGAACCCTGTGGATAGCAGTAATCACC
**CoV-bait-2055**	TGCTAGTACCACCTGGTTTAACATACATCACATTGCCAGATACGACAATTTCAGACAAGACTTGTGCACATTCATTAGCCAATCTGTAAAAGCGTTGACTATGTGTGCAACAAGCATGTT
**CoV-bait-2056**	CGGGCCGCTGAATTGTTAGCGAACGTCGATTGCCACGTGGCTACACATGGGGTCCGGTATACGGAACCCGTGTAAGGGTCAAAATAAGACAAGCTACATTCACATAACAATTATTATGAG
**CoV-bait-2057**	GTTGAGCATGCAGATTGGTATTGTGATAATAGCATCAGACACCTGGGGTTGCCATTACAGACATACCGCTGGCAGTCTATTACTATATTATCTGTTGAGATTTGAATATACTCAGTTTGA
**CoV-bait-2058**	CCATCTAACAACTCGACAGTTCTCAACAGTTGGATATCTGACTCAGCACTACCACGAATGGCAAGATAGAGATTACTTCGGCTGACGAATGGTACTATATCAGTACCCAATGTGACGTAA
**CoV-bait-2059**	CGCGTAACTGCAACATTAAATCTATTAATATTCATAGCATGGGGTGAATCTGCTGTTACAGCAAAAATGACATAGTCATACTCACTCCCCTGAGATGAATCCACGGTTTGGACTTCTAAG
**CoV-bait-2060**	TATGTGGTGAAAAATTCACACCCTCATAAAAAACTTTCTCAACACCACAATTTGAATAATTATAAGCAACAAAATCTACACTATTATTCCAATGCAAAGATATAAACATTGAGGAAGAGG
**CoV-bait-2061**	CCCGTACAATTACTTAATGTAATATTACGTAAATAACCATAACTTGTAACAGAATTAGTTCTGTAAACATTAAAAACACTAGATACCTTAGTAGTGACAAAAAATGGATACAAACCATCT

**CoV-bait-2062**	CAAATGCAGGATCAGGATTAACCCGCCTATACACTTGCTGATACAACTCATACTGCATAGACCGAACATCATCATAGACAATGTCAGGCGTACAGGTAGAGATTAAACGAGCAACATTAG
**CoV-bait-2063**	ATTTATTACCTCCTACTGTTACAACCATATCTCCACTATCTTTTGTTGCTGAGAATGTTGATGTTTTTACACCATCCAGATCATGATGACTTGGAATGATGATGTCGATAGTTGCTGTTG
**CoV-bait-2064**	ATGTTTATAACCACCCCTATCGTCAATGGCCGTCACTGGAAACTTAGTGTGAACCTTTGCGACATGAGCATTAAAATCATCTGCATCGTAGTCAGCGAGAACAAAAGAAAGCTTCTTACT
**CoV-bait-2065**	TAGGGTAATAACCACCAACAACTACACTTCCTTCTTCTTTAAAGTTACTAAACAGAAAATCTCTGATAAGGTTTTCATTGCCAGCCAATTGTGTTACGTTAACTTGTATGCATTCATTAT
**CoV-bait-2066**	CCCTTACACAAAATCTTAAAACATTGCTTACTCTCTGGATGAACAGGTTTAAATTGATTCGCATAAACAAGTTCAGAAACAGTATTAACAATTTCAGCAGGGCACCTATAACACTTATGT
**CoV-bait-2067**	GTCAACTGTAATCTTCTGTATGCTAGTCTCAATATATTCTTGTGTAACACCAAATGTAAAATTAAGCGGCAAGGACACCTTAAACTCCGAGGAGTTAAGGGCATCAACACGCAAAGGACT
**CoV-bait-2068**	CCACCAGCAGATTTAGAAAAAGCTACAGGCAAAACACCATCCTCAGAGGCAGGGTAACAGATGTCATCCATAATGTACCAGAAATAGTCATCAGAAACGACAAACTGACCACCCTCTTTT
**CoV-bait-2069**	TCTCCAGATGGTCTTGTTCCAACACCATTCACACACTCATTACAAATTTTCACATAATTATCAAACTGCGGATCATTGCGATCGACAGTAGTAGTAAAGGTAAAAGTGACAGCCAGTCCC
**CoV-bait-2070**	ACACTGCTATCCGCCATAAAATACAAAATAAGAAAAACAATAATTAACACAGCTATACCCAATAAGACACCTAGTAATATGACCGGTAGGGGGTCATAAAGACAAACGGGTGCCATATAA
**CoV-bait-2071**	GCAGAGGGGCAAAACCCATCCTCCTCTGACCTCTCAGGGTTACCCAACTTCTCCGATGCGTTCGGAAGCATCCATGGAAATTCTGGGGCCCATTTGAAGCCGAGACCGTATTTGCCCATC
**CoV-bait-2072**	TCTCCCTCACCCGTATAATTGTAAAAATCAGTAAAGTGAAAACTCCGGAAGAGAGAATTACCACTCTTCGCAGCTATCTTTCCTGAATTACACAATTTTGGGTCGAGGTCTAGCCAATCT
**CoV-bait-2073**	TGGGCCAAACGTACTAGTATCAAACCCTCTGGTTTTACCAAGATAAACTCTTGTATAAGAGCTCGTGGGTCTAATTAAAGTATACGTAGTACCGTCGAGCGTTGTTGCAACTGGCGTTAC
**CoV-bait-2074**	CATTAAACTATATAACTCAGCATTCCAAGAAAACTCTGTTATTTTTATGGCAACACTGCCACCCAGAGCCAACTTGTCACAAATTAAATGACAGAGGTAAGTAAAGAATCCATCTTTACT
**CoV-bait-2075**	TGGGACAAGTTAGCATGTTCTACAGTGTGCTCAACTGCAGTATCAATAGTATACTGGAACAGACCAAGAAACATTATTGCACATGAACCTTTTCTATTGGTTCGTAATGTTGAAGTTTAG
**CoV-bait-2076**	AAGATTCTTGGTAAAAGCCTTACAATCATTAACTTTCTCGCCATCTTCGGTAATAGGGGAATCAATGGATAGGTCTTCACAACCATCCCTAGACTCTTCCCATTCTACCCAAGAATTGGC
**CoV-bait-2077**	TGGTTGTAAAGTCTTCACAACAGTCTCTGCTATAACACATGCAAATTCAGTAACTTCGGTACCGGATTCAACAGTGTAGACAGAGCACTTTTCATTAAGCACTTTGTCAACACGCTCATC
**CoV-bait-2078**	CTGGAGAATGCATTAGCAAAGAGTGAAAGTGCGATTAACAACAACAAAGTTGGAGGTCTCTGCACTCTCATAACTAAGTTCGTTAATGGACATGAATCTTATGTGGCTCTACATCATAAT
**CoV-bait-2079**	AACACAAGCAAGTTCTTCAGCTGTAGTGCCGGTCTCTACTGTATAGGATGCACACTTCTCATTAAGAACTTTATCAACACGTTCATCCAACTCAAAAGTGATTTTAACACTTTTATAACC
**CoV-bait-2080**	TTTTATAGTAGCATTTTCATATAACACCTGGTGTGTTTCGTTATCATGAAATACACCAGTAAAAATGGGCTTATAAGCACCATCTGCAATTAATGCTGCTGAAGCTGGTCGAACTAAACC
**CoV-bait-2081**	ATTTCAGAAAAGTTACAGTGTGCACTACAAAATTGTGACTTAGACCAAGCCATACCCTGAAGAGGTGCTGTCATAGCTATAGAAGCCGCACTTTGATTATAAACATCCTTAATAACACCA
**CoV-bait-2082**	GAGTCTGAAAGAGCAATCGGCTCTGGGAGCGCTTCTTGATTATACTGCGGCTTAACGACCGGCCCATACGATTTGTCCAATTATCATGTACAGCAACATAGCCTAGGATGGTAGCCCTCT
**CoV-bait-2083**	GAGCAACACCTGAATTAGCAAAAACGGACTTATAAGCCTTAACAAGAAGCTCAGGTGCATGCTTACCCTTACGTGGACCAACAACATTAAAGACCTTAAGACCTAACGCATCCAACATGA
**CoV-bait-2084**	GATCTTCATAACTCATAGAATCATAATAAAGTCTAGCCTTTCCCCATTTATTAAAGGGGAATCCAGCTGATTTATCCAAATTGTTAACAATGACTTGGTTAGCATTTATGCAACCGCCAT

**CoV-bait-2085**	ATCAACTGATAATGGGGGACAAGTGAACTATAAGCTGGGTCAATGTAAAATGATGGATACAACTTACAGATCGAGGTGTAACGTTCCTGGTTGACAATAACAGGTGCTCGGAGAGGTGCT
**CoV-bait-2086**	ATCGCGATCATCTGCCATGCTGAGACATGAACGGAGATCTGACATTACGCGTCTGTCAGGGGTGTACTCGATAAGACGGTAGGAGAAGGCGTCCTTTTTCACCGTGTAAGTTAAACTAAA
**CoV-bait-2087**	ACATCTTCTGCACAACCATGCCAGAACCGTATGGATAAAAATGATCGTCTTCATCCTTGTAAAAGGTGTATCCAGCAATGACAATTACATCGCCATTGTCTTTAGGTTCAACATAATCTG
**CoV-bait-2088**	CAACTGGAGTGAAAATTGGTGTCAGCATGTTAAAAAGCAGGTCGACAACATCAGCACCACAGTCACACCACCTAGGCTTGTGTAACTGGGATTGTTCAAAACCCATGTACCACGAAGAGA
**CoV-bait-2089**	CTATAGGAATATCACACTCATAAGATGAGTTGTCATAAGCAGCACCAATCAAACAACCAGCCTGTGTTTGGAATATGTTGCCATCATTACGGAAGGCATACACACGCCAATCATGAGAAA
**CoV-bait-2090**	TCATATCTGAAGCTGTGACAAAGTCTTCGGCTTTAAGAATGCCCATTTTACTAAGGCGCACTTGAGAAATGAGCAGATGGAGACCTCCCAAAGTGGTTTTGGAAACATCACCATAGACAA
**CoV-bait-2091**	AGTTGCAATACGCGGTGCAAAACTGGCTGCTGCTCCACGCCATGCCGCTGCTCGGCGCGGTCATCGCAATGCTGCTCGCGTTCACCACACGGCCGCCATGAATAATGCCCACGGTGCAGC
**CoV-bait-2092**	ATTGAAGGCGCTAGTCCCCACTAGCGCGACAGACTTGCACCGTCCAGATGTGCAGTTGGAGACGGTAGTCATCACTAGTCCTCCCCCTCTATCTTTATGAGTCGGCGTCGTCGCCGACAT
**CoV-bait-2093**	AACCTTCTTACTGCTCTGCAAGTTAACACCAAACATTTGTTTAACAACCTCACTCAAAGTGAAGTCGTCCGTAAGTGCAGAATAACCCAAAACATTACGACCACCAAAACCATTAGCGTG
**CoV-bait-2094**	TCATGGCTACTGAGGCCGCACTGAAATTCTTACTGTAGCCAATAGCACCAGCAATACAACTTTGGGAGGTACCTGCATTATTAAGCTCCTCCGACACATTAACTACTGCATAAGCACCTC
**CoV-bait-2095**	TTTGATTCTCCTGCAGCACATCTGTTTGCAGTGCAACATAGTTTAACCTAGATTGAACAGCAAGTGAAAAAGGTATAGAGGCAGCAGCACTTAAGCCACCCATAACCATAGCTCCAGTGA
**CoV-bait-2096**	AAACTCACGAACAGTCTTTGGCATAGCACCAATGAAGCTAGTGACAGTCGTGTTAGCTATGGTAGAGTTAATGAAGCAAAAGTAAGTATCCGCCACTAAACCAAGAGGCACATAGGGTCG
**CoV-bait-2097**	TTTTCGGATGCGGAAGATACCGAGGGTAACGATGCCGTGATTCTACTCGGCCGTCGGTCATCATTTGATTTGGGGCAGAGCCATATTGCTCCGAATTACCAAATTAGAGAGCATTTGGGG
**CoV-bait-2098**	TACTAACGTTACCATAGATGGTTCTGCCTTCATGCCAAGTGAAAAAATCATGCTTAGCAACAGCATTACAGCCTTTAAGTAAGTTATACATGGACTGCTCGTGGTCCATAACTGACTTAA
**CoV-bait-2099**	GCAATAAATACAAACAGAAGCGCCACCATATGTATCCTGTGTAGTATTAGAGTCAATAGTACTAGTAATAGCCTGACCACTACCAGAACCATTAGTCAACATCTTTACACAGTTGCCAAC
**CoV-bait-2100**	ATCACCTTCAGCTTTGGTGGCTTTAACCTTCATCTTGCCCGGCATTATTTCATTGTTCTGCAATTTAACGACGCGTTCACAAGTCAAAATCAGAGGCCAAACAAGTGTTTCCTGGTTTTC
**CoV-bait-2101**	ATGCTCTTTTAGTTATCAATAGTTGCCAATGGCCTTCTGGCCAATTTTATAATGTTTAAGGACCCGCTATTCATACGTACGACGCAACTGCAGTTAAGAGCGCTAATAAACACCATGAAC
**CoV-bait-2102**	AGCATAAAGAAATGCGACCACATTAACTGTTAGCATCTGGTTGGCAGATTCAACTTGAAGGTTAGGTTGGTCTTCAAAACCACCATACATAACACCATCAAAGCTAGAACCTACATGGCT
**CoV-bait-2103**	ATGTTAAAGACATTGTTGGTGTTGTGTATGACACACCATCGTAAAGAACCATTACACGGAGTGAACCGAGGAATAGGGTTGATGGATAGTGAAGTCTTGCTGAGGAACAAAGTTCTGTGT
**CoV-bait-2104**	CATTAAAAATGACACTTTCCACATAAGGAACCTCAAAAAGCTTAACACCGTTCATGTAAAAGGAACCATAGCGGCTAATGACAATTTCTTTAACAACAGGGGGCAATATGCCAACAAATC
**CoV-bait-2105**	GTCACGTGAGTTGTCCCGTGATTGGTAACGCGAGCCATTGTTACTCGGTCTAGCTGGGGTGCTGCTGCGCGACCTTGAGTAGTTGTTATTGTTCCTGCTCTGCGAGCGGGAGTTAGGTCG

**CoV-bait-2106**	TAGAAGCCTCACGGTCAAATTCGCTCTTTGCTACATTCATGGCATGGCGCAATTGCTTAACCAACTGAGGTGGAGAACCATTATTAACAGCATCTTCATACTGTTGGCGTGCATTTTCAT
**CoV-bait-2107**	TCATAGTTCGTGCACATCGAAACCTCATCCACCACAACTATGTCAGCATTACATTCAGGTAGTGCATTGACAGTGGAGAAGAGGTACTGGGCACTATTGTTGTTAGACTTGAAACCGCTG
**CoV-bait-2108**	ACTTCAAGAATGCATAACAGACTGCTATGGCAAACAGAGCTATAAGCATATTAGTTAACATATACCCAGACGTTGCAACAACCGACAAGTTTTGATTAAATAACTTAAACACATTAGTTA
**CoV-bait-2109**	GAACCGCCTAGCCTGTACTCTCTAGACGGTCGATAAGCTCATCGCAACGACCCTCTTACCAAAGAGGCTAGGATACTTAGATACAGTCCGAGCCGATAATAGAAACGGCCCGTCTGAAAT
**CoV-bait-2110**	TATCACAACCAAAAAGAACATGTCGCAAGAATAAAAATGCACGTGCAACAACAAAACAGACAACTATCTCATCTCCAAAGGTATCAAAAGGTATAATGTGCAAAAGCCAAAAACTCTGTT
**CoV-bait-2111**	TGGGTCCAAATAGAATTGTATGTGAAAAGTACCTGTAAAAGAATTTATGATATTCGTTGTCATAAGCTTTAATGGTGTAAGTTTCATCATCATTAATACATGCAACTTTAGTATGTGGAA
**CoV-bait-2112**	AAGTTATACAACAACAATAAACCTAACAAAAATTTTAGATAAAACCAAAATTTAGTGTTTGTAAGCAATTTTACAGAATTAAGAGCTCGCAGTTTTACGTCCAATGGCTTTAAAGCAAAA
**CoV-bait-2113**	CAAGCCACACATACCAAGGCCATTTAATATAAGTCTTGAGTATTGAAAGTGCTTCAAGATCAATAAGAGAGTCATTAAGCCCCTGTATTACTTCTTGAATTCTGTCAATCTCATTACTAA
**CoV-bait-2114**	TTTTCAATTGTTTTAACAATACTGCATTTGCCTCCTCTTTCAAAGCTTTTTCATAAGCTTCTCTAGCAGTTTCATAAGCTACAAAACTCGGCAAATTAGCAAAAGTGCTAGCTACAGATT
**CoV-bait-2115**	TGGCCCCAGTATTTAAAATACTTCTCAAACAGAGACTGTTTATGGGCAGTAAAATCATATTCCAACAAATCATAGGATTTAAAATCCTCACCAAAAATGTCACTTTTCATAAAACACTCT
**CoV-bait-2116**	GTCTCTCAGTGTAAGTGCTCTATTCTGGTGGCAAATACCTGAATAGGCTTGCACTGTGGCGTATTGTGTGGGAAGCAGAACAGTGTGGAAAAACATCAGTCCATACGGTGCTGCATTTGA
**CoV-bait-2117**	GAAATGCAAGGATATGGTGACTCAAAAATGTATCGGCAAAGCCAAGCTTAAGTAGAGGCCACGTAAAGCACCTTAACTGTGGCATTTGAAAAACAACAACCAGAGTCTTAACAAAAAAGT
**CoV-bait-2118**	ATACTCCGCATCGTGGTGCTTAGTGAGTGGATAAGCGTCTATTGCCAATGAGACATAGCGCTCCATCATAAGCGTACCATCAGTTTTAAGCAGGTCATCAACAAAGCAACCAGCACCCAA
**CoV-bait-2119**	TATAACTCCAGTACAAACCAAACTTATTGGCATCAAACTCGGAACTACCAACACCAATGGCCACATCATAATGACCTAGACCATTATAGCCATAATAAACACCACCACCTTTACCTATGT
**CoV-bait-2120**	CCACTCTGAAGATTAACACCATACATTTGCCTTATGACTTCTGTAGGCGTGAATTCATCACACAAGGAGCCATATGACAGTATAGTGCGTCCTCCAAAACCCTTATTGAGTCTAACAATA
**CoV-bait-2121**	GAAATTACTTGCAGGATCTGATTTTGGTAAAAGGCACCTACGGTAAAGACTTGTTGGAGTTGGTCTACGTATGCGCTGAGTTCAGTTGTTACGGTGTTAATCATTGTGAACTGATCTCTT
**CoV-bait-2122**	TTGGCATGTACACATCACGAGACGTAATGAAATACATGCCATTTTCACTTATAAAAATACCCCTACCACTAAATGGTACAAGTGCATATTCAGTACCATTTTCTGGTCTTACACAAAAGC
**CoV-bait-2123**	TATAATCAACACACTTATTTAAAGTAATGTTATTATAATTAAAATGTGTAAATGTAGGCACTGTGTTTGATGTTTGAATACGAGAACCATCAGTTTTACTAATAAAAACTAGCAAACCGC
**CoV-bait-2124**	AAAACAGTACCATTAGAAACTAGTGGTATTGAAAGCCTTGGTTTATGGTTGTGACAAAAATAAGACATACCACCAAGGTACAATTTTGTAACATCCGCTTCACCACAACCTGGTTGAGTA
**CoV-bait-2125**	AACTAAATCAGCTACGTCTGTACCAGCAGAACACTTTTTATAATCATCATCTACAGTGCCAAGACCGCTGGTTACCACCTTATTGAACAATAGATCCTCAACAGCAGACCTCATACCTAT
**CoV-bait-2126**	CACCAACGACATTATCGCCATTAGTGTAGGAGACGTACAAAACAGCAAACTTTTGAGGTTCTCCATAAGAGCCCTGGGTAATAATGCTCATGCCACAACCACCATTAGGAACAAGCGAAA
**CoV-bait-2127**	TGACCCCATAAAACCATTAGGTAGTTCTTCAAAGAACTTAGCAACGGCACCATTAATGCTCGCAAGGCAAGTTCCAGAAAATTCACGAATGACAAGAGCTCTTTCCGCAACAACAATTGT
**CoV-bait-2128**	ACCTGCCATCTAAATTACGAAACAACGTCAACTGGACATCTTTAACGACAAGTCCAAAAGTGCGCTCGCCATCTGAAGCACAAATACCTGACCAAGCTGTTACAGTTTCATAAGCTGTTG

**CoV-bait-2129**	ACACATTGAAAAGATTTAAAATTAGGGTATTTAGATACGCTAACTGTTAAATTATAAAATAAAGAACCATTTTTCATTGCAGAAATACGAATATAATCACGTGGAATCATGCCTGTTATA
**CoV-bait-2130**	CGATCATCACTCGGCTTATGAGAATCGCGAATGGGCAGTCGAAACGGTCCCTATGCATATACGCGATCTCATTAACGAATTATCCCACCCGTCTTCCCTCAGCATATGCTTTACTTGCGC
**CoV-bait-2131**	ACAATCTACACAGTTAGGATGGTAATCTTGACCCCAGTACTTAAAATATTTGTTATACAGCCTAAGCTTATGGTCTGTAAAGTCATAGGCTAACAGATCATAAGTTCTAAAATCTGAACC
**CoV-bait-2132**	AGGAGCGACCATCTTCTTTATACCTGCTTGTAGCCTCGTAACCAAATTAACACCAAATCTAGGTGGCGTATACAGCACCTCTACTTGTGTGTTCTTAAAATTCTCAAGAGCATTAGCTAA
**CoV-bait-2133**	GCAGAGATGAAAGCTATTATGCCAGAGATGATCTGGGTTGACAAGTCTATAGGGTAGACGGCGCAAAAAATGGAGAGCGCCATTGAAGCTGGCCAAAGTAGCCATAGTACTAGCATTTTG
**CoV-bait-2134**	AATTCTTCAAATATAAATTCTAATTCTAACTCAATCTCTAATTACTCAATAATATCATCCAACCAATCTTCCTCTTCTTCTTCCTCAACTCAAACTATTTCAACTAAAACACCAAATTCC
**CoV-bait-2135**	CGTGGCACTCTTGTAAATCCGCTGGCCAGTACCGCAGTCACAAACGACGTCACACGTAACTTGCATAGATGAAACACCCTTAAGAATTGCCTCAACGCAAGTACCTACGTCACCCAATGA
**CoV-bait-2136**	TAAAGCGTATTGTCATGAATAGTAATGCATACAATGCATAAATACCCAAACTAAACTTACCAAGAACTTTGAACCAATAAAGTTTTAGCTTGAAGAAGACACCCAAAGCCTTTGCATTAT
**CoV-bait-2137**	CCTATTGGGTTGCCGCACGGCGCTAATTACGACATCCCGCTGGCAGGCGATGCACTGGCTGGCCCATGCATTGCCCGTTCCAAACTTAACCGATTTCACTCTGTACCATCCACAAGAATT
**CoV-bait-2138**	AGCCTAGGCTGCAAAAGAGTGCAGAGGATGCTGAGCAGGCTTCTGAGAGAGATGCATATGGGAAGTAGTACTGTACACTTTCTGTTAAGCCGAATGCGACAAGAACTTTTTGAAATGTTA
**CoV-bait-2139**	GCTGACCAGAAGAACGGCTCAACCGTTAGGTTAGTGGAGTTAGGCTTACAGGATAGATGATAAGGTTGTTTGAATATATACCGGAAGATACACTGCCTCAACATAGGTAAGTATCTCTAA
**CoV-bait-2140**	TGTCTGGGCGTAGGCAACATCATGTTTACTTTTGCCAAACACACCAGCATCATTAAAACCATACCTCCTATTCCAAGACGAGGGGTTATAGTTATTAATGGTAACATTATTCTTAGGAAG
**CoV-bait-2141**	CTAGCTACTTCAACAAATCCCTGGGACACAGTGTCTAGTGCTGACTGTATACCTTTAAGGACTTTATTAAAAGAATTTGCTATCAAATTTTGGTTATCTAAAAGCACCGATTGTGTCAAC
**CoV-bait-2142**	AACGTCTTTGTTGTAAACATCAAAAGCACGAACACATCTATCGATGTCAGTGCCATTTAGGGGCTCTAGTCGAGCATCACTAGAGCCCCGCACTCGTTTAAATAACCATTGTCAAAGCTC
**CoV-bait-2143**	CCTCGGGTTCGTCGTCACCTTCTGTACTGTCTGTAATGATGGTTTCGTCATCATATACAGCAGGTTCTTGTTGGTCATCAGACTCAGCATCCACAAATGACTCGTCGTCCGACTTGTCAG
**CoV-bait-2144**	CTTAACTACATCTTTATGTGAGTAAATTCAGATACGCGCGTCCCTTTAACCAAAAAACACTTGCACCTAGAACACACATGCAAAATGTGCTCTGGCAGTAAAAGCCCTAGGCCATGTCTT
**CoV-bait-2145**	GTGAACGAGTGGTTCCTTTCTCGAGTAATTCGTCTATAACCAGGCTAATGCCGTGGCTAATGTGTGAAGAGTAGCACCCTGGCTTAGGTAAGCAACAGGGTTCTAATTTATTTCTACACC
**CoV-bait-2146**	TCATCAAAATTGCGGAGAGCGAAGACAAGGTCCATCATAGTATACTTAGTCAGATTTTGCCTGCTAACATTACCATAAACAGACCTACCCTCACGCCATGTAAAGAAATCATGTGAAGCC
**CoV-bait-2147**	GACGGCGCCTTGGTCTGGGTGTTTCAAAATCAGTTGCTTTGAAAAGACCACGCTCTCTCAAAACCAAAACAACCTTAGACCAGTCAGGATCCTTCTTAACAACCACTTTATCATCAAAAG
**CoV-bait-2148**	TCTAATGAGTATTTTACCCTTCCTTAGCAATCCAACAACCATATCATTTAATTCTCTTTCTTTTAAATTAACAACAAGTGCGTTATTACAACGGCACTTAAACTTCGGAGTATCTAGAAC
**CoV-bait-2149**	AGCGCACATTACCATTGCAAACATAAGTGGAGCAATCAACTACGATAGGTGTACTTGTAATTTGTAAATACTCAACCTGGACCGAAGTGGTCCAATTGGAAGGTATAGACAAATTAGCTG
**CoV-bait-2150**	ATACACTTACCAATGTCCATGGCTGACACACCAGATGAACCTGGTTTGACTTTTGATGGCCAAAAGTTGTTAAAAGAGCGGTCAGTAATCAGAATGCCAAATCTATGAGCATTGTTGACA
**CoV-bait-2151**	TGCCCTATTGTACATCGACCACGGAAAGGATGGACAGGCAGTTAACAGGGTTGCATCGCACTGGCAACGATCTTCTTCGCTGACGCTCCTCTTTCTTGGCATATGGTTGGATTCGATACA

**CoV-bait-2152**	TAAATTAATAAGCTTAATACAGCGAGATGTTGATGCCATAGAATAAAGCTTACGATAAAAGCCAACTTTATTATTAACCCAACCAGAGGAGCCTTCTTGGTAACAGGTATAATTTCTATG
**CoV-bait-2153**	GCCAGCATTGTTGGACTCGCTGGAGATATTCACCACAGCGTAAGCTCCTCCATGCAGGTGCCATCCGTTTGGTGGGCGAAAGGCGCTCTGGTAATAGTACACATAGCTGGAGCTGTCGTA
**CoV-bait-2154**	CGCTAGGAGGAGCCTACTTGTTGATCCCTGGGCTCAAATAATAGAGCGAGGATCATGCTGGCTGTGTGAAATGAGCAGACAGGGCGAGTGCCGCCGGATACATCCTAAACTCAATTCAGT
**CoV-bait-2155**	TATGATTAAGGTCAGTCGACCGTCAGCGACATGAGACGTACCGCGTCTAAGTGCCAGCAGTCATCATCTGGCCACGTACCACCAAGGGCCAATGTGCTTATATCCATGCTAGATCGAGGG
**CoV-bait-2156**	TGTTAAGTGCATCATTATCAAGCTTCCTAAGCATAGTGAAGAGCATTGTTTGCATAGCACTAGTTACTTTTGCCCTCTTGTCCTCAGATCTTGCCTGTTTGTACATTTGGGTCATAGCCT
**CoV-bait-2157**	TGACTTGCAATAATAGCTCATACCTCCTAGATACAGTTGTGTCACATCAGTGACATCACAACCTGGGGCATTGCAAACATAGGGATTAACAGACAACACTAATTTGTGTGATGTTGAAAT
**CoV-bait-2158**	ACAATGGACCAATCTACACGCTTGACAAAGCAATCATATATAGCAAGGCACCTAGTCATGATAGCATCACCACTAGCTACATGCTCATTTCTATGAATGTTACAAACTTCATGATGATTC
**CoV-bait-2159**	CAACACGTTCATCAAGCTCAAATGTGATTCTCACATTCTTGTAACCTTGAACTTCCCAAACAGTATCTTCTCCAAAGGTTACACCTTTAATTGGTGCACCCCCTTTTAAGCGAAAGACAT
**CoV-bait-2160**	CTAATATCGTACGACCATTCATACCATTTTGTAATAATTCTTTTAACGCCGCACACATATCTAATACCGCTATACCCGTTTGCGCCGATAACGGACCTAATATATCTACATGATCTTGCG
**CoV-bait-2161**	GCGGTTCTTCTTGTTTCGGCGCTTCTACACGCGGTTTTAAATTATCTAAATAATCCATTACTACTTGTTCATATAACGCTTTATCATTTACCGCTATATATACTTGCGTACGTACCGTTT
**CoV-bait-2162**	TAAACACAAACTCTCGTAAGTGTTTAAAATTACCTGACTTTTCTGAAACATCAAGCGAAAAGGCATCAGATATGTACTCGAAAGTGCAATTAAATGCATTATCGAATATCATAGTATGTG
**CoV-bait-2163**	CCTATCGTCGATACATGCGCCGGCGCTTCACGTTTATAATCCCATATTACCGTATTCGCCGCTATATCTACACCTAAATTATTTAATATTTTTATTTCCGGTACCGGTTTTATATTACGT
**CoV-bait-2164**	AGACTACTCTTATTAGAGTGAACATGTCAGAGATGAATTTTGCAGTAGCTTTGTAAGTAGACATACATGCCTGTACAAAAGCTGTAACTGCGCTCTGGCATGAGGAGAAGACAAGGTTAA
**CoV-bait-2165**	CGTAATTTTAACGGAAATTTCGACATATCAAATAACGAATACGACGATAATTGTATCGGATTCGTATTACGCCAAAATATATAATTCGCATGCATCGTATAACCATCTATTTGTTCTTTC
**CoV-bait-2166**	TTATAAAACAAGGAATTACTTCCCTTCCTCATAGCAGAAACACGAATAAATCCACTCGGAATAAACCCTGTTAGAGGGCATAGACCGGTACCGTGTTTAAAACAATGCGTAACGAACACT
**CoV-bait-2167**	ATCGTGCAATCTCGTTGTGGGATTTTGATTTACGGGTCAGTCTGAATCAGAGTCCGTGAGCGCGGTGTCTATCGCTGTTACGACCGCCAAAGCCATTTGTGGCCCTCTCTGATTCCTTAG
**CoV-bait-2168**	TTACATTCTACTGTCTTATAACCTATATTGGCTTGTTGTTTAAAAATACCCTCAAGGTTAAAAATAAAGGGGTAGGCTGACAAAGGTTGTGTTGTAGAGTATGCTAAACCTGCAAGATTA
**CoV-bait-2169**	CTGCGAGAGGATTTATGATTGTGCCATCTTCAACGGCCTGAAGCATGTTGGTAAAACCTGGTGGTAAAACGATGGTGAAGATGTTAAAGGTTTCAGCATCTGTTTTCTTACAGAGTGTTG
**CoV-bait-2170**	ACCCGTTCACTGACCCCTCAACTACTAATTAAAAACAAGGATAGAAGAAAGCGAACGCGAACCGAGCCCCTATCTGTAAGGCCGTGTAGCCATGGAAGCACGGCCGGCTTGCCGAGCCGA
**CoV-bait-2171**	TGCGTAAGCGTATCAGGAGAAGCGACAAGCAACGGACAGATCAAGGGGATAGGGGTAGGGACAGACTCCGTTGACGAGGATTCCCATAATCATCGCCAGCCCTAATTACCCCGTGATTTA
**CoV-bait-2172**	CGATGAGCGAACGCGTAACGCGAATGGTGCAGCGCGATCGTAATCACCCGAGTGTGATCATCTGGTCGCTGGGGAATGAATCAGGCCACGGCGCTAATCACGACGCGCTGTATCGCTGGA

**CoV-bait-2173**	TGAGCGACCAGCACCTTTCTTGCTAGCTATTGCTACAGTTGGTACGCTTGTATGCATACGACAGTCGTTAAATGTCAACATAAATGTCAATCCTTTAACCTTGGTCGTGCGAATTAGGTA
**CoV-bait-2174**	CGGATGTTAAATTATTAACACACTGAAATGATTTAAAAGTAGGGTACTTAGCTACACTAACTGTTAAATTATAGAAAAGCTGGCCATTTTTCATAGCAGAAACACGTATAAAATGCTGTT
**CoV-bait-2175**	TGGCCTGTTCAGTACCAGACACCACCCTCCGTCGTTTCCGCTCTGTGAACATGGGTAAAGCTCCGCGGCAAGTGTTCGCGGACATATGGCGATTGTGTGACACAACATGCCAGGATAGCG
**CoV-bait-2176**	TTAAAAAACCAAATAAACCTATTATCCAACAAAACTACAACAAACAATGGCCACATTAACGGATTAAAAAAACCAAAATTAGAAACAGTCACACCATTCTCTACATTCATAACAGGTTGT
**CoV-bait-2177**	TTGCTATGCCGCCACCGTGAGACAAATTCTCATTTGCAGCATTAACAACAAAGTCACATGGAAGTTTAACAAGGTCACTAAAATCTCCTTGGTAAAAATCAACATTCTTATAAGAATAGA
**CoV-bait-2178**	AACCATGTGCACTTTGAAAACTCTCCACAACACCCAAAGCCTCAATGTACTGATCAGAAACATTAGTAACCACAAAAACTACAACATCTTTATCTAAATCTACCAAATTACTAAGCAATG
**CoV-bait-2179**	CAGGTTTTACCAGGTTACCATTGTTGTCAAAACCATACTGGTCCGACATATAAGCATTACCACCACGTTTAGCCTTAGGCTTTGTGGGGCGGCAGTTGTCTGGATAATCCAGATACTGCC
**CoV-bait-2180**	TCTAGGAATTGGCAGGTTTTGGCATCATCTTTTGGCAGGTAGTAGGTATGAGTGAGCGTGACTTTCACTTGATCTCCGGCTTCTTCAGCGGACCATTGACTACCGAAGAGCATGCTAGAC
**CoV-bait-2181**	AACTATAGGTATACAGAGTACAGGCTTATGTGCTCTGCAGTAAATGGCTGTTCCTGAAATGAACAGTTCTTCTACATTATCTTCATTGCAATTTTCAACACTACAGATGTAGTTGTTGAT
**CoV-bait-2182**	TTCTCTTACAGTAGCGACGTTCAGCCATTTCATTAGCCTTAGCTTTAGTGATACGGCTACCTCGCTTCTGCTGCTCTTCAATAATCTTGGCTGCGCGTGCAATTAGATCTTCATTAGGAC
**CoV-bait-2183**	TTAACTTCACTCAAAATGGTGTTAATGTTATCACAAAAAGTACCATACTCTACTAATTGCTGCCGACATGCAGCATAATCTGAGCAGACAAAAGCTGCACAGTCTATAGTAACCTTAGGG
**CoV-bait-2184**	TAACAATGCAGGACTTGATGCTACAAGTAACGTAGGGTCAGTGACAAACCTAAGAAGATCTGTCATAGTCAACTTCATAGTGTCTAATTTAACATCAAGATTCCACACAATACCTAACTG
**CoV-bait-2185**	GTTCAACATCATCGTTGTCAGAAATCGAATCACTTTTATCATCAACAGCTGTGTTAAGATCATACTGTGAAATCATAACACCATCTGGGTTGTTAATGTCAAATCCACCACATGTGTCGT
**CoV-bait-2186**	AAGTTCGACCAACTGAGCACTTGTAAATACAAGGCTTTTCAGACTAGTCACAAACGTGTCCCACGCTTCTCCGAGAAGTTCCTCGAGCTTTCGAACGAACCACGGTTTAGTTAGAACAAC
**CoV-bait-2187**	AAGCCTGTGTGTCTACTGTGAGGTTTATAAGCACTGCAACACGTTTAATGTTGCAAGTAGAAGTATTACTTGGGTAAGTATACTTAATACAACAAGTCATGACAGGCCGGTAGAAACCCG
**CoV-bait-2188**	CTCTTAAGTGTACTATCAGTAGTTGACATGAACTCTTCGATTGTAAAAACACCCATATTTGCCAACCTAACTTGTGAGATTAACAAATGTAAACCACCAAGTGTCCATTTAGAAACATCG
**CoV-bait-2189**	TAAAGCCGTTTGGAAGTTCTTCAAAGAATTTTGCAACAGCACCATTGATACTTGCAAGGCAAGTTCCTGCGAACTCTCTAACAACTAGAGTCCTTTCCACAACTACAACAGTAATTGACT
**CoV-bait-2190**	TTGCTGCGTGCAACTCTCTTGCACACTTCACATGTTACATCCTTACAATACAGTATATGATGCACCTGTATGTATATCTTATAAAAGAGCCAGAAATAAAACCCTGCGCCCATAAAATTA
**CoV-bait-2191**	AAACTTCATCGCCATCTTGAACAATTTTGTGTACACCCCCTTTCCAGAAGAACAAGTCTCCATGAATTCTACCAAGTGCACACTTCTTAAAACTCTTATACAGAGAATGTATTGCATCCA
**CoV-bait-2192**	TTCACCTGGAGGTGCTTTAGAATTCACAGGCTCAAAATTATTGCCTATTGAAGTGTCAACAAGACCCTCAGGTGTAACTACAAAGTCTGCACCAGTAGAGAAGCCTATTTGAAAAGGGAG
**CoV-bait-2193**	TAATTAGTTTAATAATTGTAATACAAACTAGCAAAAGTACTATAAGTACTACGCACCACAGTATTACATTTACAACTAAAGCATGATCATCCACTAGCTTCAGAAACATCTTCAAATTCC
**CoV-bait-2194**	CGGGTCCCACATTCCTTACTTACATTTACCTAAACCATACCTCTTCAATGAACTTGGGACACCACCGCCAAGAACGTGCTTATATACACGCTCTATAACAGACGTCCAAGTTAAATCGGC
**CoV-bait-2195**	ATCTTCTGGATGCATGTTTAGTTGACTTCATCAATTATGTCAGTTTCAATAGAAACTGCATCGTAGACTGGTTCAACAGTTCCAGGTGAAGTTTGTGATGGATTGAATTCTTGTGCACTA

**CoV-bait-2196**	TTGTGTATCCATATCGAAACCGTTCCATTAGCAGTTACTAACTAGATAGGTCTCAAGACAAATGACTGGCTCTTCCATTGTTGGCTCGTCAAAGCGATCTTTAATTACTCCAGCACATTT
**CoV-bait-2197**	AATCCAATGTGTTCTTTAGATAAACGTTGAAGTTTACCTTTAGTGTACACATCTAAAGCTTTAGCAAGTCCACCACCATGGGCAAGGTTTTCGTTGGCAGCATTTACAATAAAGTCAAAG
**CoV-bait-2198**	AATGTCCAAACTGCAAAATTAGAATGAATATGGTTAGGATAACATTCCAACCAAAATTCCAATTTTTCAAGTGTGTAACGATATTGTCGTTACACGAGGCATTGTTGTTGTACAACACAC
**CoV-bait-2199**	AGGGCACACATCATACTGTACTATACGGCCATTTAAAGTGGTGACATTAACTGTTGTGGCATTATCTACAATGCTAAAATAGCAAGATTGTATATTAGCACACTTCACGTTAACAACATT
**CoV-bait-2200**	CAAGGTGCACCAGCATATTGACTGCAAAAATCATACCACTTGTCAACATAATCCTTAACATAACGCGGTGCACACAAGCTGGATTTGACAATAATTTCATTGTCCAAAACAGCAGCGACA
**CoV-bait-2201**	AACCTTAAGGTTTCTGACAAAATCTGCTACGGCGCTCACACGGCGAACAACACTTCGCCATATGGCACCGCCATCAATTTGCAAAAACAAAGGATGTGGCTTGGGTACAGCAGGAGCCAT
**CoV-bait-2202**	TTACTAACAACCGTGCCGGAAATAATGGAAGAGGAATTAGAAAGCACAAACCAATTGTTAAAGCCAAAGGATGGTGGTATAGAACCATCATCGCCAACGGTAAACAAATTGTCTGAAAAT
**CoV-bait-2203**	GCTTCAGTAGCTGGGACTCCTGGTGCAATAGGCACACCTTGTCCTTCTGCAAACTCAAACTCCTTTCCCTTTTGAAACTGAGTAATTCCAGAGAACCAAGAATAGTAGGGTACAACATTC
**CoV-bait-2204**	CCTTGCCAACTGGCACAGCATTGTTAGGAAGAACTTTCCAAATGGGTTGGTCAGAATTAATAATAAGTGGGGCAAAATAGGAGTATTCCATCCTTCCTCTGCGGTTGTTCTTGGCGAACT
**CoV-bait-2205**	CCCCTTAGCCCTGTTCATAGCATGCTTAAGTTGCTTAATTAACTGGGGTGAGGAACGGTTTGAAATTGCTTCCTCATATGCCTGTCTTGCATTCTCATACATAATGTATGATGGCATGTT
**CoV-bait-2206**	TTCCACTGGCAACCGGCGCACTAGGAGCTGGTGTTGGTGGACGTGAACTTCTGTCCTTCTTCTTCTTGGGTTCCCTCTGAGGTTTTGCCTCTTTATAAGCATTAACATTAGAGAGGAAGA
**CoV-bait-2207**	TGGTAAGAGGTGGAGTCAAACCAACCTTTCGCTTTGCATACAATTCAAATGCCACATTGGTAGGTAGAGATGTCTTATTTGTGAACACGAGGTTATCAACATCGTTCTCACGAACAAGAA
**CoV-bait-2208**	GTGGGTTCGTAGATGGGTAGGTTTGGGAAGTTCCTTGGAGATGGACCGACTCAAGCACTGAAACCGCCGCGTCCGAGAACTAACTGTGTCTCAGATAATATCCACTTTGTGATTCTTGCC
**CoV-bait-2209**	TTACAAACGTATGGTGTTATAGCCAGTATAAACCTGTGATCGGTACCAAAAACGTGATCGTAAGCACACTTAGTACACAACATTGGTCTACGGAGGCAATCACCACAACGCAAAACAGTT
**CoV-bait-2210**	CCTTAGAAAGTGCTACAGAAACAGTCTCAAAGCCTTTTGCAACCTCTTGAAAACCAGCTGACACCACATCAAGTGCTGACTGTATTTTTTCAAGAGCATTATTAAATGAATTAGCAATAA
**CoV-bait-2211**	AGGCAAAGCGCGGGTCTCCTGATTCGAATACGCGATGATTGCGTCGATTTGTTTGAGAACCTACTCTACCGCTAGGGGTAGCCTTCTTCCACCTGCGCAACTTAGTCTATGCCCATCACC
**CoV-bait-2212**	CCTGTATGGGCCACTGTGAAACCATAACAGGCTTAGTGTAATCAAAACCTCCCTCCTCATCGTACACAAAGTACTCAGGTATAGGGAGTTTAGTTGAGACAACAGACAAGACACCACGAA
**CoV-bait-2213**	ACAGCATTGGCATACTTGGCAGTTCGAAGACTGTGCTCACGCACACCCTTCACTTTTTCTGTGACAATCTTAACAATGGCGTTGTTAAGCAGTATGTAACTGCCAATGGCTCTGAACTGT
**CoV-bait-2214**	TTTACACTTAATTTCCTTGTGGAAATTAATCGTAAGATTACCTGTATTATTGGTCCACAAACCAAAGACTGTCCATTTTCTTTGGGTCAGGTCAATAATTATTTAAAGTTTGGTAGTATC
**CoV-bait-2215**	AAAACTGAAGCCTCCCAAAGTCACTGGGGGGACTTGCGACCTTGAAGACTTTCTTTGGCATCATCCATGTCTCCAGTGTCTCCAGTTTGCTCACAGCAAGGTTGGTCACATGGTGCTTGA
**CoV-bait-2216**	ATGTTTTCAGCTAGAATGGGGCAGTATGATCCAATACAGATGAGGCGGTATGGGGGAAGCTCAGACATGAGTTGTGCGATGTCTGGTGTGGCAGTCTGAACGATCTGCAGAAGTTGTTGG
**CoV-bait-2217**	CATTTTACAACTTTCAAATCTGGCTTGTCAGAGATAAAGGCGTACATAAAAGTATTGCCACCCTCTGTGTTATAAAGTGCCTTACCATCAGCCACAACACCACCGTCACCTTCACCCTTA
**CoV-bait-2218**	TCAAATTGCAAACGGGCTGTTCCAATGTGGAGGGAAGGGAGGATAGTACCGATGTAACCGCACCCTAGCAAAAACAACTTAGGTCCTTGTTAGGATTCTGCTGCAGAATTAGGTACTATA

**CoV-bait-2219**	ACAAACACCTTGTAACATTCTAGGTTGACCAATAGAACACCCAGGCTTACCTCCAGGATGACATGCATAATTACCACAGTTATCAACTCTAACACTATCAGACCAACCAGTATAACCGCC
**CoV-bait-2220**	GATTGCAAATAGCCTTTGGCCCTGAGGAAGTAACTTCCAGGGCGTGATCAATCCTCAAGACCCTTGGTTTTTTTTCGTAGTATACTTTCATTGACGGCACTCAATAACAAAAACTGTCGA
**CoV-bait-2221**	TGCACACCTGATACCTTTAGGATTTCCAATGTCATGAATTGCTTTAGGATTATACAACTTTATAGCTGATTTAACAACCATAGATTGCACAACTCTACCACTCTTATTGATGGCATGCTC
**CoV-bait-2222**	GTCCCTTTACTTTCAATTCACAAAGGGTGAGTTGATTACTGGCACGCCTAAACCACTTGAAGGTGTCACGGACGTTTCTTTTATGACTCTGGATGTGTGTACCACGTATACTATCTATGG
**CoV-bait-2223**	GCTCTGTAAACGTCCTGTTATAAGCCTGTCAATTTGAACTTCGGCCTCTACTTTGTCAAGTCGAGACAGAATATCATTCAGTACACTAGAAATAGCACCAAAATTGGAGCTAAGCTGTTT
**CoV-bait-2224**	CCTCACTTGCGGGTTGTTGCGCGCCGCTCCGCTTACAGCCCTCATATCCCTTGACTTGGCCATTACTTGTGTTGGATGGTCAGTCCTGACACTAGAAACACTTCAACTCAACTGCAGGCT
**CoV-bait-2225**	CAGACATAAGGTGTCACAGACAAGATGAACTTATGTGTAGTGCCAACAACATGATCATATGCACACTTAGTACACAACATAGGACGACGTATGCAATCACCACAACGAAGCACAGTTTGA
**CoV-bait-2226**	GAGAGCTGACTACGTAGTCATATGTGCCTAATGTGCACCTAAACAACCTGTTTAAGAGGTTAAACACACCAAAGTAACAGGTACAGCAATAGCCTATGATCACATAGCAGACAAGGACGC
**CoV-bait-2227**	GTTTAGTCGGACTCCTCTCAGATGGCCGGCAGTCCGTTAACTTTGCGTATCATTCCATAACGACCCCTACGATCTATACAACTCTGAGCATAGATCGCTCGCCGGCGCCGCCGACCGACC
**CoV-bait-2228**	GCCACAACCGAATGCTATTGACAAAATACATTATCCACAGCATAAGAGTGATGCAGGCCATAAGGATGCTGAAAGCGAAAAAGACCCAGTTGACCTGGAAGCTAGCCCATGCGTCGAAAA
**CoV-bait-2229**	CACTCACTAGCCTCACAGCCGAGTCTAGCTTCCATAGTTTTCACAGATGCCTCCAACGGAGTCTTCACAACCTGGGCCATGTCCAGCTTGTTAAATCTCAGTACTTCCTAGAGCTGAATC
**CoV-bait-2230**	GGTCAAAGTAAAGTGTTAACATCAAAGCAAAAAGGGTCTAATGAATTTCTATGCAAAAGTGCATCATAAGAAGATGTTGCCACAAACATTCCATCAGAATGAACAGCTGTAACACGCCAT
**CoV-bait-2231**	GTTGATCATTTTCAACAATCTTACGGCCTCCCGCGTATAAATTTACGTCGTTACACAACACTTCGCGACAGCCACGCCATCCACCTTTGCGTCTCAGTGGGGAGCACCAATTCGCCAAAT
**CoV-bait-2232**	GGGTAACCTGCGCTCTTATCTAGATTATTAACTACAACTTGGCTTGCTGGTATACAGCCGCCTTCATAGCATTCAAAATATTTAGAAGTCACTTCTAAACAAAATAGAAGTTGACGTATG
**CoV-bait-2233**	TAATCACCATTACGGTCATCATACAAAACAACAAGCCCTTTAGGTTCTATGTCTGTATAAGGACACACATTAACAGTGTTACGATACAATGGTGTTTGATTGGTATAGTCCCATATAACA
**CoV-bait-2234**	ATCTCCTTGGTAAAATGAGACTTTGCCCGCTGTATAAAAGGGTAGTATAAAATCTCACCCACAACGCTTTGCGTGGATACGCGCATCGGGATCGTATGGGTGTGGGCACGGCGATTCCTA
**CoV-bait-2235**	TGAAATTCAGGGCGCCTTGGACGTATTTTCCCATGGTGACTTGGAAACGGCCAGTACTCTCGAGGAGTATTTTATACTTGTCAAATGTCAAATCCTTGACATTCTGGATGAGTGATTCAA
**CoV-bait-2236**	AGCTTGACCACTTCAGACCTAAAATACGTTCATGACAAATACTCTCATCTGCACATAGCCTGAATGAACGTACTGTACATGAAGTAGAACAATTAGCATTAAAGGCCGGTATGGTGCTGT
**CoV-bait-2237**	ACAATAGGACAGGACTTATCAAATGTAGGTACAAAGCCGTATTTAGCTTTAAACCACAATGCAAAGCCTTTGTATGTATTATGTACACAGTTAATAGAATCATCAAATGGTTGAACAACA
**CoV-bait-2238**	GAGGCGGAAATAGCACCAAAAGTATTAGATAGCTCGCTAGCTAATTTGGATAGAGCCTGTGCATTGTTGTTCACAGCATCCTGAACCTTGTGAAAAGCTTCATTAGTTGTAGTGAAGCCT
**CoV-bait-2239**	ATGTGGCCCTTTCTTCAGATCGGTTTCCACCCAGCATTTAGCTTCAGACATAAAGACATTGTTCTGATAATACAGCGTTTCCTTAAAATTCTGTATTCCAGCAATGTAACCCTTAGCTGC
**CoV-bait-2240**	GTTGCTCTGTAGCAGTACAGAGCTGAATAGGCTTAGGCTCATCTACAATGGACTCCTCATCGTCATCCATAGGAAATGCCTCATCAACAAGACTAGCCCAGGCTTCGTCACCTGAACACT
**CoV-bait-2241**	TGACTCTTACCAGTGCCAGGTGGTCCCTGCACGGTAACATACTTACTATAACCTGCTTTTTGGAAGTTAGCAACGTGATTGGCAAACTCCTCAGGCACAGTAATAGTTGGATACAATCCA

**CoV-bait-2242**	CTAATAATAACAGTGCCAGTGGAATTGGCAGCTGCTCCTATACGGACGACAAACCCATTAGCAAACTGTTTGACGTCCTGAGAATAGTTAGCTACAAACAACTTTTGTGGAGTTGTGCCT
**CoV-bait-2243**	CCTTTAGCCAGAATATACTCATCTGACTCTTTTTGGACAGCCCCTTTTGAAGCCGCATTAATAGCACCAGCGATACCACCGCCATGCTTAAGATGTGTGTTAGCAGCATTAACTAACACA
**CoV-bait-2244**	TGATCGTGAGTGGGTTGTTGATGAAATTTTGCAGATGTTGGAGTAGTTATCCTTGTAAACTGTTGTAACTAGGGATTTAAGTCTAACTAGGGATTGTTGCATGATGCCAGTTGTTACTGA
**CoV-bait-2245**	TCTTAAGCAGGCCCTTGGCATACTTCCCCTTAGGGTTGCCCTCGAGGTCATCCAAGAGTTCAAAAGGAATTTCCTCATCCGGGTTAGGACGAGGTTTGCGGAAGTAATTACCTCCGAATC
**CoV-bait-2246**	GAAGATAACGTACTGGTATTCTGAACCCTGTGAGGAATCAACAGTCTGAGTAGTAAGGCCCAGCATTGAACGAGCCACAGCATTCTGTGAATTATAAGGCGAAATAAAGACTGCCTTACT
**CoV-bait-2247**	GTACTTAAGAGGCTTAGTAATAGTAGTAAGGTTATGAGGAACAGTCGCTAAAATCAAACATGTGGGATTAGAAAAGGACTGTTTATAATTAAACTGGGATATTGGACCAGCAGAACTAAC
**CoV-bait-2248**	AACTTTAACAGAGGTGCCACAATCACATGCAACATTACATGTCACCTTAAGTGTTTTGACATCCTTACACAAACGTTCCAAAAGCTGTGTTGTATCACCCAAACTGCCCTTAACAGCTCC
**CoV-bait-2249**	ATTTCGCTGCACGGCACTTTTGGGCTGTGCAACGCTTATACTATCTAATTCTTCTTTCTGAGCCGTTTGTTTCGTCCCTCTCTTTCTTTGGGGCTTTGGGCTCACTACATCTACACCACC
**CoV-bait-2250**	TCAACTTAGACTGCACAGTAGAAATTTTTATAGTACGTTGTCCACCAACACCCACTAGCCGCAAAGACAAAACTAATGAGTCAAGGGGTCCACGTGGTGCACTAAGACCATTAGCGACCA
**CoV-bait-2251**	GTACTTCACAAAGTAAACTCTATGAGTACACTCTAGATGTCTACCATTAACAGGGTGTTGAAAATCCCTGTCGTGGTATATCTGAGTCCCAAATGGAAGGTCAACAGCAAATGGTGTGTA
**CoV-bait-2252**	CCAGAATAGTTAGAGGTTACAGAAATAACCTCATGGTACACAGTAGAGCTCTCAAACTGTTGTTATTAGAAAGCCCTCCGAATTCTATATTTCACATCTCTCGTATAAATCAGTGCCAGG
**CoV-bait-2253**	CAGCGGCTTGTTCAGCCATCCTACCAATTTTCTTCTGAACAGACAATTCATGGTCAAACTCAGACTTTGCAATGTTCATAGCCCTCTTTAGCTGTTTGACAAGTTGTGGGTTGGAACCAT
**CoV-bait-2254**	ATGCCACAACTCCTGACGTTGATTGCCAAGATTGGAATGGCAAATGGTATTAGGATATTCACACATGGTATACTGGCAAACCGTAGCTTCCAGTATACCATTATGTGGTTGTACAACTAT
**CoV-bait-2255**	AGATATAGTATGTGGCTGAATTGTAGGTGTAACCCATAGTATAATTTTCATGTCCTGTTGTTGTTGTGGCTACATGAAAGTATGGGAAATTACCATCAACAGTAAAGACTACTGCTGTGT
**CoV-bait-2256**	CCCAGGATCACTATTATAACAATGTGTGACAAAAACTGTAATTTCAGAAAAATTACAGTGTGCACTACAGAATTGTGACTTAGACCAATCCATACCCTGAGGAGGTGCTGTCATAGCTAT
**CoV-bait-2257**	TCATCAAGCAACTGTAAGGACTTTAAGACTTCTTTACAACTATACTTTTTATCTGTAACATCAAAATTGTAACGAGTGTACTCATCACCGGTGTAAAAACGGTAAAAGCCATCAGAGCAT
**CoV-bait-2258**	GGATTAATCCACGTAAAGGGTGTGTACATGAAGAATTCTAACCAAAATGCAAAAAGGATAGCCATAGCAGTCATCATGGGGTAAAATAGAGACTTCACCTTACCACACTGAAGATTAACA
**CoV-bait-2259**	AACTGGCAGACCAAGTAATAAATACTACACTATCAGAAAGATCCAAAAGATAATCAGACAACATTTGAACAATTCTGATTCTAACAACATCCCACTTTTGACCTTTCGACATAAGGGGTA
**CoV-bait-2260**	CCAAACCAAACCTAATTGCTTAAAATGATAACCAGCAGTTGTAACAAGTGGAACACCATCTATAAAAACTTTACGACATAGTGGACCAAAAGCAGTACCTGGTATAGTTGTGGCAAATAG
**CoV-bait-2261**	GCATTTAAGCATAGCTCTAGCTACAATTTTGCCAAGAGATGCATAAACTCTATGTATATCTTCATTTTCAACTGGGTCATACCAACCCTTACTATCAAAATAAGAATTGTCACAACAACC
**CoV-bait-2262**	TTCTAAACAAAACCTAATAGGATCCAAACAACCAATAGGAACCTGAACACATTTACCCTTAAACTTACAGTAACCATCCATACTAGGGTGAGGAACGTGGGCCCGACAATACAAACAAAT
**CoV-bait-2263**	TACATAAAAGTTTTACCACCCTCAGTATTATACAAGGCATTACCATCACCTAAAACACCACCATCACCCTCAGCTTTCATAGGTTTTTGCTTAAGTTTACCAGGCATAATTTCATTATTT

**CoV-bait-2264**	CCGTAAACCTGGAGAGAGATGATTGCATTAGTTTCAGGATTAAAAGCCCAAAAAGTTTTAACACGGCGCCAAAGTCTGAAACTATTAACAAAATACATAACCCATAAACAAAGTGTAATA
**CoV-bait-2265**	TACTTCAGCTGGAACAGGTGCTATTTGCATATAATCTTGGTAAGCAAGAAAAATTTTATAAACTGGTTGATATAATGTCCTACTAAAAAAATAATGACAAGTAAAACACAATTGAATCAG
**CoV-bait-2266**	ATGTTAGAACTCACGGCTTGAAAAATGTTAAAAATAGAATTAGCATAAGCTGTACTAGCGTCACCAGAAGTCGTACCACCTGGCTTAAAATAAAAACCACCATTAGAATAAACAACTTCT
**CoV-bait-2267**	CCTTCTCTTCAACAACAACTTGTTCAGTCAACACACTAAAATCACCAGTAGAATTTTCCAATGGTCCTTCAGAATTATTTTGAGCGTGTTCCTGTTGACACAGTTGTTCTTGTGCATCAA
**CoV-bait-2268**	GGTGGTAAATCAACAACATTAGGATTTACTGTAAATCTCACATTATGTAACAATGCATACCAAGCCATCAACATAGAATCAGCATGTGAAGAAGGTGCACACAAAACAGTTTCCTTTGCT
**CoV-bait-2269**	CCTAAAACGACATAGTGGTCACCACCATAAATAGCAGCAAAACGATTTTCATAAAATGATTGTTCAAGATACCAACACTTGCCTGAAACGAAGCATAGTTTAGTAACATTGAACAAAACA
**CoV-bait-2270**	GGCATTATTGAACGTGGACCTTTTCAAATTCGTAATAAGGAAGTTTAGTTGAACCACAATCACAACAGCCTCGCATTGATGAAGTTAAACAATTGCAACAACCACAACAACCTGTAGAAA
**CoV-bait-2271**	ATAGAACCCTTTAAAGCCAAGTCTCTAAAGTTAGCACCAGATTTAGGAAAATAACCCGTAAATAACAAGGTAGTATTTAAATAAACACGGTTAAGAACATAATATGTGCCCAAACCAAGA
**CoV-bait-2272**	GCCCACCCAGTTCCCCTGAGCCTGAACGGGCGACCTATTCTCCGATACATCGATCTTTGTCTGACTTGTTATGCGGAGCTAATCTTCCCAGTCCTGGTGCCCGTCAATACGCGAGGCCCT
**CoV-bait-2273**	CTACAGATGTGCATTTGACATCTGACATTTTGGACTGCACAGTTGAAACTTTAATAGTAGGAACACCACCGATACCGGCCAGTCTGATGTTAGTTTTAAGCGCCTCCCAACTTGTTCTTG
**CoV-bait-2274**	TCCTTAAGAACGTCGCAATTCTTTTCATCAAAATTGCGAAGTGCAAAGCACAGATCCATCATGGTGTATTTCGTCAAATCATGACGAGCAATATTACCAAAGATACTGCGCCCCTCTTTA
**CoV-bait-2275**	ACAATCAGCCGCATTAGCGACGCTGCCATTATCAAAATAAGAAACTAACAAATCCCTATTAACTAAATGTGCTATATGCAAATTAATATTAAAATCAGTACACTGATCGTCTACACCATT
**CoV-bait-2276**	AATGGTTGGTTGGAAACCACCTTACCATGCACCAAAGTGGAATCATTGGACAAAAGAAACCAATTATTAAAACTAAAACCTTCTGGTATGTGGCCATTGGGCTCAGTAGCAAATACATTG
**CoV-bait-2277**	ATCAATTCCTGCTCTACAACAACGACTTGTTCAGTCGCTTCAACACCAACATCAGCGGGTTTTTCAACTGAGTATTTAAGAGGTTCAGGCTCTTGCCCACACAATTGTTCTTGTGCATCA
**CoV-bait-2278**	AAATCCTAGCACAGAATTACCACAAATGTATCCATTACCAAAGTCATTGTCGTAGACAAACCAGTTATCACCACCAAAGCAAAAACCAGCTTTACTATCAATGCACTCTCCCACTCTACA
**CoV-bait-2279**	TTCTTAGGGTCTAGTTTAATTGCTCCTGAATATGACAAAAAGTAAATAGGATCACCTTTGTCATCATTGCTTTGATGGGTAAGCTTAAATTGAGACATACTCATGAAAGCTGAAGCTGAA
**CoV-bait-2280**	CATTAGAACACCCACTGCGCATAGCAGAAATGCGAATATAGTCTCTAGGAATCAAACCTGTTAAAGGACACTCACCTGCTCCAGTCTTAAAACAATGTGTAACAAACACTGTAAAATGTG
**CoV-bait-2281**	TCAAGGAAGGCTTCAAGTGAATCAAGAGGTTTATGTCCATACGCACCTGCACCGAAGAGTGGAAGGTAGATGTGGGTGAGCTTGTTTAGTTGGGCATAGTCAACAATTGCTTTGTATGTT
**CoV-bait-2282**	CAAGCTGACCATGTGTCATATAATCGTACTCCATGGTCTTAACATTACTGTTAACAGGCTGTTTGTCATAACAATACCACTTGGCAGTAGTCACAGCACAACGAATGCCCTTGGGGTTGC
**CoV-bait-2283**	GAGCAGCTGCAATAGACTTAAGCATCTTCTGATGGAATTGCCTATTAGTCATAGTAGACGCAATAGACACTCCAGCCACTGTTCGAGCACGATTCTTCGCGCTAATAGCATACTTCAAAT
**CoV-bait-2284**	ACGTGCAGTATTAAAAGGTTTTGGGTCCTGGCAGAAGATACCAGTGCCAACCAGTGTCTTGATCTTGAGTTTAAGTACTTTGCCACAAACTGTGCAAGCACCATAATCAAAACTATCTCT
**CoV-bait-2285**	ACCGGCCTTCTTATCTAACAACAACTTCTGTGTTAGACAAGTAATGACTTGTTTAACATCAGAACGTGTTACATAAAATTTAGCTCCTGATTTTATAACACCAGACACTAAAAACTTAAG
**CoV-bait-2286**	ATAGAAGCAGAAGCTTCCACATGTTGTCCAAGTAGTATAAAGTAATCAGATTTTTACACATTGTAAAATCTAAATTAACAAATACAACCCTTCATTTTGCAAGCCATGCGAAGAAATTCA
**CoV-bait-2287**	CAACTGTCAAAACTTTAGGGATGTCTGGCCTATGCCTAAAAACTGGAGTATAATTATTTACCAATGGAACAAGCTGGACCCTACGCTTGAAAAAAAACACCTCTGCCAAATTCTCAAAAG

**CoV-bait-2288**	GTTAAGTTAACACCCTGTAAAGGACTAAATATTACAAAGTCTCTACACTCGGCCAAATTAACCACATCCTGCCACTGAGAGGCCATATACTTACCAAGAATATGTCTAGGTACATAAATA
**CoV-bait-2289**	GTTAATGCCATTAGAGAAGCCCAATTGTAGCGGTAGGTTAGTGCCAACGTTAGGTTTAACTGCATGTGCGGCCTCAACGTCAAAACCTACCCATGATTGCACATACTGCTTGGCTTCATC
**CoV-bait-2290**	AAATGTACAAACTTCCTTAGATTTACCATCTGAGGCTATTATTATGGCATTCTGGACTGGCTGTGCATGTGCTACAACACTGTCAACATTCAGCTTACGGTAATAAAGTGAAATAAGTGC
**CoV-bait-2291**	GGTAATAATCCTGGCCTACACCAAGTGTCGTGTTAAGACAATGTAATGTATACAGCTCTCCGTCCGGTGTAGTAAAGTTAATACTGCCTGCAGTGAGCTGTAATATGTCGGTTGTGACAT
**CoV-bait-2292**	TACATAGCCTTCATTGCTTGATCCGCTAGCTTCTCCAATTTACGATTGGCAGCAGCATCACGATCCCATTCAGATTTAGCTATGTTCGCAATTTTAAGTTTCCTCTTTTGTTCTGCCAGA
**CoV-bait-2293**	CTCGTATATGAGTTATTTTGAGCCCACTCATTAAACTCCGCTACTGATATTTCAGTAGAAGCTAACCAATTAGGTTTAGTTGTAAGTGTCAACAAATGTGTATATATGTGAGCCAACACA
**CoV-bait-2294**	GTATAATAGAAGGAATAAGAGGGTGGCAGTGGAGCACCACCTGGTTTAACACGCTTGTAGCGCAGCCAGTAACCATGGCACATCTCCTTAGGGAGGGTTGTATTAATAGGCACACCATCA
**CoV-bait-2295**	GTGGCCATATTATATGGCGGTTCTACTACATCACAAGGTAGCCCAGTTTTAGCCAATAAAAAGACACCTACAAAGAACAGTGCTTTTAATATAAGTGTTGCTGGATATATCCAATAAAAT
**CoV-bait-2296**	GACACTTTAAGTGTTGTAAGAGTTTTATGGTATAATTTACCAACTACCTCTACTCCATAGGCACATGTAGTTAGTCCAACATCAACAGCCTGCAAAACCACAAACGTACCATCCACGACA
**CoV-bait-2297**	TCAAAAGGGTCAAATACAGCTCGCTCTTGAGTGATTGAAATAGTGGATAATATGTTTGTGACCCGAATAATTACAGAATCACCTAATTGTAAAAGCTGATCAATAGTGATTGTGCTAGGG
**CoV-bait-2298**	ATTTACATTTTCGTAAACCATATTACCGGTTGTACATTTCCAGCCGTCCGCCACGGGTAAATAACCACTAACAAACACCGTGGAATTTGCAGGCAAACCCAAATTGAGATTGGGCAATAA
**CoV-bait-2299**	TTCTTTGAGCCAGAACGAGACTTGCTACCACCATCGTCTCGGCTTTGGCTACGAGAGCTAGCACGGGAGTTTGGTTGGCTAGGGGCATTGACGATTTCAATGTTTGAAGGCAGATCGAAA
**CoV-bait-2300**	ATTGACGTATTGAGCAAGGAAGTAGAGCATACAAGAACGTGTAATAGTTCCACCAAAAATCAAGAGGAAAGCCATGTAAAACATAGGCATTACCTTCTTAAATAGTGGATCTTTAACATG
**CoV-bait-2301**	AAGCCCATAAAAGATATAACGTGTTCATATTTAACAACGTTAGTACCATTAATTTGCACAGCTAAATCTTGGTCAGTCTTAAAATTATCAGAGAGTGCGACATATGTAGACGCATAAGAA
**CoV-bait-2302**	GACTAATAAGTTGACTCAATGCTTCACCCTGTTGATTAACTACACTCTGGATTTTAGTCAATGCTGAAGTCATAGTATTAAAACCATCTGAAATGGTTGTAATAGCATTAGAAACCTTTC
**CoV-bait-2303**	TGAAATGCGTGCTTATTTACATACAAAGAACCACCATTTACACCTTCTAGATTATACTTATTACACACACGTGTATCAAACCTACAGACTAAAGAAAATCCTGGATAACAACTTACATTA
**CoV-bait-2304**	CAGTATCTGAAGTCTGACAATAAATAACGTAGTCAAACTCTGAACCCTGAGAAGAGTCCACAGTTTGAGTCTGCAAACCCAGCATGCGTCTAGCAACTGCATTCTGGCTATTATATGGAG
**CoV-bait-2305**	TTTTTTTGACTGCTTTTTACTACTTCTGGGGTAGGTCTAATATTAACTAACATAACATCTTACATGGAGACACTCAACCATAAAATCCAGCAAAACCATTAAAATCTGGATGGTTTACAC
**CoV-bait-2306**	CATCAACATTCTCACTACCAACACAGTTGGCTCTGTAAATACCAAACACAGTACCATTTGAGACCAAAGGTATAGAAAGCCTAGGTTTGTGGTTAACACAAAAATAGGACATACCACCAA
**CoV-bait-2307**	ATCATCACTAAGATTGATTTCATTGTGCAAATTCACACAATAATTCCACTCCTTAGAGTTAGCCTCAATATGCATTTTAGACAACAAACCTAATAAAACAACATTAGTACATTTCATATC
**CoV-bait-2308**	GTTGCATCAAACCCATCCTGGATAGCACCCAGCGCATTGTTAAAAGCACTAGCAATCATCTTTTGGTTCTCACTAAGCACATTCATAGTGACACCTAAACCATTAATTCTATATTGAACA
**CoV-bait-2309**	TTGCCTGCACTGGCTGTGTAATCATTAGACAAACTCTGAAGTTTACCCTTAGTATACACATCCAATGCCTTTGCAAGACCACCACCATGAGCGAGGTTCTCATTAGCTGCATTCACAACA
**CoV-bait-2310**	GAAGCAACATCTTCTACGCTCACATCAGATTCGACTGAGTAATCAACAGCAGTGTTAACATCATACTGTGAGATCATGATACCATCAGTATTGTTGATGTCGAAACCACCACAGGTGTCA
**CoV-bait-2311**	GAACCATTGTACTTAATTTTAGAACCTATGGCTTTCTTACAGACTGCAGCAATATTTTCATCTTCAAATTCATAATCTAATTTTACTTTGTAAACTGGATCAATTTCTTTGACTTCAACA

**CoV-bait-2312**	GACACCTTCTTTTAACAAGGTCCCTCCAACAATGTGGTACATTCTCAAAATTATAATCAAATTCTCCAACTTTACCATCAAAGCCAATTAAATCATCAACCTCATCACTACATTTCAATA
**CoV-bait-2313**	TTTACAAGACACAACAAGATGTGGTCGTCAGTCCATGGCTCACCGATCATACCTAATAACTTAACAATATTAAACATCACAAAGATGGTTGTTACAAATAGTGAAAGCCATGACTGCTTG
**CoV-bait-2314**	ATAGTACACGACGACACTATGCTGGGATTAGCACATGGACAGTAAGACTTCCCCACTATAAAGCACTGCTCTGCATAGGCGACGTCATGTTTACCACTACCAAATGTAGCTACATCGTTA
**CoV-bait-2315**	GTCTGAAGGGGCTGCTGATTAACACGTAAATGCGGATACAAATGTAGGGAGTCTTGACCGTCAAAACAGGCTTGACAAACCAAGTCACCTGCACAGTAAGAATTATAATCAAAGTGTTGC
**CoV-bait-2316**	ACTAGGGTCGATGTCAGTATACGTAGATACATTAATAGTGTATTGGAAAATTGTATCATTGTCGAACCACACAGTGAAATTTCTGGTGGCGGTTACGCCTAAACCAGATAACAGCTGTGT
**CoV-bait-2317**	CTTCAATAGCAGATCTGCCACCTAGTCTGGTGGTTAGTATGCTGCTAAAATTGTAGTCACCCTTGAAGTTGGTAATATTAGCTAACTGCAAGGACTGTGTTGATGTTTGAAACATATTTA
**CoV-bait-2318**	GTTCGCACAGTGCAATACGCAACGGTCATCTGGACATTCAACAGTGTTAGGATGGTAAGGACGATCCCAGTACTTAAAATACTTCTCGAACAAGCCAAGCTTGAAGTCCGTGAAGTCATA
**CoV-bait-2319**	TCCAAAATGGTAGACTGGTCTTCTGCATTGAGGGTTGAAAAGTCAAAGGCGTAATGTGGTAATATATTGTTAGGGTTGTCAATTGTAAAATCAAGACCGCGCTGGACAGCCAGAACAGCT
**CoV-bait-2320**	TGCCTTTTGCATCACCGGTACTATTTACCATGGCTTCCATATCAAAATAGAATGCATGTATATTATTGAAATTGTGGAAGCTAGTGGTGCTAGAACTTATTATATTAGAGCAATTGTACC
**CoV-bait-2321**	TTACCAGCAGTAGTACCGGTGGCAGCAGCAGTGGTAGGTGCTGTAGTACTGGTGTTATTGCCTAAAACGCCGCTAAGGGCCAAGAAAAATGCTAGAATAAATCTCTTCATTTTCGTTTAG
**CoV-bait-2322**	AAGCCAAACATACCAAGGCCATTTGATATATGTTTTAAGTATTGACAATGTTTCGAGATCTATCAAGGAGTCATTAAGACCCTTGATGACCTGTTCTATCTTGTCAATATCATAAGTAAT
**CoV-bait-2323**	TAATATGTGCTTTGTCTTCGGATCGTACATATCACTAATAATAAGATCCCATTTATTAGCTGTATAAACAGTAGAGCAGTCTCCTATTAATGTAGAATCAGCATCAGACACAAAATCATT
**CoV-bait-2324**	AAATCAACGTGCCAATCAAGAGATCCTGCAGGTCTATTTTGAAACACTGTAATACAGTGCCCAGGACCATCATCACCTCCTCTTTGCCAGTGAATTTTCCAAACCTTACTGCCTGACTCC
**CoV-bait-2325**	CCCATAATGTAGGACATATTAGTTTGTTCACATGGGAAGTTAGCAAAGGAATTGTTAGCAGCCCATGAGTTGAAATCCTCGATACTTATCTGAGATTGTGCCAGCCATTTTGGTCTAGCA
**CoV-bait-2326**	GTTTGGCACTCAACATCTCTAGCAAGTAAGAATGCTGGTGCTGTTGGTTTGACACTAAGCCTGGTAGCTCGTGAGAGCGACTCAATAGCTTCTGTCGGGATGAAAGTGCTGGGTGTTTGT
**CoV-bait-2327**	TTAACAGTAACAATAACATCATCACCAGACTCAGTAGCTTCCCACTTTCCACCAAACACTAGTGCACCTGTGGAAGGAACCATTTCAGCCAACTGAGGGTAGTTTTTATGCGAACTACCG
**CoV-bait-2328**	AGCGTCATAATTATACAACCAGGTCTCAACATCTTCCATTGTAAGTCCTCTTCTAACAGTCCATTGTCGTGTGGGAGGAAAAGTTATACGCAGGTAATGTGTAGGTTGTTTAGCAACCTG
**CoV-bait-2329**	AAAACAGCACCACGCCTCAAAGTGTTCAAATTCTTAACAAAGTACAAATGTTTAATTTCAGGTCCATTAGCACCCTCCACATAAAACTTAAGTGGCTGTTCGAGTTCGATGGTAATAACA
**CoV-bait-2330**	GGATCATCACTGAGATTAATCTCGTTATGAAGTGTAACACAATAATTCCATTCTTTAGAGTTAGCTTCTACATGCATTTTTGAAAGCAAGCCTAGTAGCACCACATTGGTACATTTCATA
**CoV-bait-2331**	CACGTTGTTGCCATCAATGCTTTTATTTGTACTATCATACATGTCAGATATTAGAAGATCAAACTTGTCTTCTAAATACATACTAGTACAATCTCCTGTAATACTAAAATCTGCATCTGA
**CoV-bait-2332**	GAAATGCCACCTGACAAAGAGGCTGTATACATGCTCATTTTACCATCATTAGCAACACCCGGCAAGACCATGATACCATTATAATACTGTGCACATGCTAAATCAGCAATATCAGAACCC
**CoV-bait-2333**	GTAATAGAAATGTTGTCAAAAATGCATGTTTATGCTTAACGCATAACATAGCAAGTGCCAACACAACAAACATAATTGGAGTCAATTGTAATGGTATAACATTAAACAAATAGTTCCAAA
**CoV-bait-2334**	CAGCTACACCAGCTAATCCAATTAGCAACCAAATGTACCAAGGCCATTTCACATACATTTCATATGTGCCAACTTCCTTGAGGTTGATGTAGCTCTCATTTAACTTCTTAATTGCATCCT

**CoV-bait-2335**	TAATAGCTACAGTACCACCCAAAGCTAGTTTCTCAGTTATTACACCATTGATGTAAACAAAGAAACCATCCTTAGAAACATTTTCACCATCCATTTGTTTAATACGACCGTCATACATAT
**CoV-bait-2336**	TTTATTAACAAATGTTTAGCTTCACCACTAAGCTTTACGAAGTCTGTTGCAGACCACACAATAGGTACATTCTCTTTACTTAAGACACTATGATTAACAGCCTTAGCATTAGCCCTAATA
**CoV-bait-2337**	GAGGACTGTGAACGCACACACTCATTAACTTTCTGTTTAGCAAGTTGTCTGCTGGCTCTAACCTCAGCTTGTCTAGTGAGAGTTTGTGTCACAAAAGCGTTAAGTGCCGCCAGCCTACCA
**CoV-bait-2338**	AGTCTGTAGTAGCACTGACAGTGCCATTGAACAAAACAGCATCAAGACGTGGAAATTTATGAACCCTGTAGCCATTCAAGTAAACGTCACCATAAACGGTGACGACAATCTCTCGAAGAA
**CoV-bait-2339**	ACGACGAACTGGTCATCGACGGTGGTTGATCTATAACAGCACTCATAAAGCTTACGCTGCAATTGCTTAACTTCTAAATTATGACAGACATTGCTGTCAACACTAAGAAGTTTGTTAACA
**CoV-bait-2340**	AAGGCTAAAAGATGGTTGTGTGGAGATGTTGTGTTGGATCTCTGGGATGTAGTAACTGCCATCAGGTTTAGAGGCGAGCATAAATTGATCACAGGTGTTAACTCCGTAGTGAATTCCTAC
**CoV-bait-2341**	TCATAAGACAGTGATTCATAATACAAGCTAGCCTTACCGAACTTGTTGAGTGGATAACCAGCGCTCTTATTAAGATTAGTGACGACAACGTCACGAGCAGCTATGCAACCACCTTCATAT
**CoV-bait-2342**	TAGGCGAGACGTAAGGAGTGCTATATTGGTTAGTTCTGTCCACTGACGGGTGGTGCGACCGGTAATACTTAACCCTACAGTAGTCCAACGCTGTTTAACAAACGACTACACAACAAACAA
**CoV-bait-2343**	ACGGGCATGGTCACCTGAATAAAGAGTTACCGCCGGTTCGGCGGTTTCCGAGCATAGACCCGTATCTGCATATAGCGAGTTTGTGGTAAAAGATAAAAGCCCACGTTTACAAGAACCTCT
**CoV-bait-2344**	ATGACTTAGTTGGAAACACCAAAAACTTACCTGCAGTTGATGCATTATACACACCTGGTTCCATTAGCAAATACTTACACACAAAATTATGAAACTCACTACCAGAATACTTATTATTAT
**CoV-bait-2345**	GTGAATGTTTTGGATTTTGTGGTGGAATGTTCGTCTAGCATGTAGGAATCGTGGAGAAGTTTGATGAAATTACCTTCCATTGTGTCTGAGTGATTGTACAAGAAAAAGTTGTTGTCAACT
**CoV-bait-2346**	ACTATTGTAAGGTTGGCGGCCACCTGGTGCTATAACGGTAGCAGGCCACTGCTGCGCTATGGCATACATGGGGCTGGTATCATTAATATAATACGAATGCACACACCCATAAAAATCGGG
**CoV-bait-2347**	ATGGTAGGATTATCCACTACTTCTTCAGCGACAGTTTGTTGACTTTCACAAGCAAGATTGTCCATTCCTTGTGCGTCATCTGAACTTAACACCTCAAACGAATTTGAAGTGGGAACAGGC
**CoV-bait-2348**	TGATGTTTCAGGTAATACCATGGAAAATAATGTATTAAAATTGGCACAATGTATTATACAATTATCATCAAAACACTCTACACAATTAGGATGATAAACACGATCCCAATATTTAAAATA
**CoV-bait-2349**	AAGGCCTTGAGTATTCACGACATTCTCAATTTTACTAATCGCCTCAGCGACAGTCGAGAGTGCCATGGATGTCTGTTGAAGAGCATCATTGACTTGTGAAAAGCCAATGGTGATCTGTTC
**CoV-bait-2350**	TTTTCATAGCAGAAACACGTATAGAATGCTGTGGAATGGAGCCAGTTATAGGACACCCATTATGTTTATAACAATGTGTAACAAACACTGTAATATCTGAAAAGTTACAGTGTGCAGTAC
**CoV-bait-2351**	TATCGACTTTGAAAGCAGAGCCACACGAACAGTTGGCTTCATTAAAGTTGCTAGTGCACCAGACAGTCATGTCGCACGTAGCACAGTTGCATAATGCACTGAGTGCCATGGGCTGAAGTT
**CoV-bait-2352**	ACAGCACCAGATGCAGTGGAATTAATATTATAGGCTCCATTGTATATTGGTGTTTTGCCGTAGGGCACGACTAAATCAGTAGCATTTGGTGTAAACGTGTTGACTTGGCGCAGGACAGCA
**CoV-bait-2353**	TCCAAGCATGCAGTTAGTGGGAAAGAGCGTAGCATAGATTGGGTCACTAGCAGTAATCAGATGTGCGCCCACAGTACAGACCCCATCAACGGAGAATGTTTTTGTAGTACACCAAATCTC
**CoV-bait-2354**	TCTTGTTGATAACCACTAATGTTAACACCCATAGATGATTTTCTGGGCTTTGCTTGCTCATAGGTAAACCTTTCATTTGTTTGATAACACTCCGTTACTCTCTTCATAAAATCATAAACA
**CoV-bait-2355**	ATTTCCGGGTTTGGAAATTAGCAGCCCAGAGTTGTTGCTCAAATGATGCCATGTTCGGGATACTTGAAGTTGACCCAAACAATGGAGAAAATATGAGCAATGTGTGCACTCGTAAACGAG

**CoV-bait-2356**	AATTTCTGCACTCCAGAGAATTGCCACAAATATACTGCAGGCAATTAATTTGGACCTTATCCATACGCGTTTGTATGTACTCATCGGTAACAGTTAAATTAAAACTGTTAGGTATGAGCA
**CoV-bait-2357**	ACTTGGAATAAGGAATGTGTACAAGAACAACACTTTATGCTTAATAGTAAGTGTGAGCATGACAGCAATTCCAAACAACAAGACAAACACAGCAGTGATTAGGTCTGGTTTTATCCAATA
**CoV-bait-2358**	ATACTTAAGATTTCCTCTGGGACCTGTGCCTGTATAATAGAAGGCATAGGATGGAGGAATTGCAGCACCACCTGGCTTTTGTCTATTGTAACGTAGCCAATAGCCATGGTTTTCCTGGGC
**CoV-bait-2359**	CAGGACAAAGAATGGTTTTAATGGCCTGAATCTTTGTATCCTTGATATCCTCCAACTCATTAAGATGATTGAAGGTGTCAAGAAAACCCTTCTTGTCAGAATCTCGTTTTATAATGTCCC
**CoV-bait-2360**	GCATAACAAGCCATGCGATAGTCAGCCTCTCCCATTGAACCAGTATAGTACTTATACTTGTTAAAGCTGTTAGCATATGACTTAATTCTATCTAAAGATGTGGAATTAACAAGTGTTTCA
**CoV-bait-2361**	AAAAGATGCTTGCACGTAGGGTTTAGCAACAAAGTGCCGTTGGTTGAGAGCTTGTAGGACGTTGTGGCGGTTACCGGTGAAGGTTTGCGCTAGCGCGATTCTAGAAGAGATTTAAGTTAT
**CoV-bait-2362**	ATACTACCATAGTAATAGAAATCATGTCTGCACACTCAAAAGCAACACAGTTGCCAGCAGGCACTGTTTTGTTACTCTTTTTAAATACATTTATGACTTCAAGCAATGCACCGCCGGTCA
**CoV-bait-2363**	GGTACCATGAATGGCTATCAATAAGCCAACTTTATCACTAGTATCCCGAAGGCCATCTTCATAAGAGCTTTCTCGTAGGTCGAAGTAGACACCGTGAACATTCGGTATATTCAGATTTTG
**CoV-bait-2364**	AATCTTCAGAAAGCACGACCTTAGAATTCTTCTTGACTGGTGGTGCTACTTCTCTGATAGCACAATTTGGCAACTTGTGTGGGATGTCCAGATTGTATTGGATTTCCTTAATTGTGAGGA
**CoV-bait-2365**	ACCAGCTTCTAAGAGAACAGACTGGTCTTCTGCTGAAAGGGTGTTAAAGTCAAAAGCATAATGCGGTAATATATGGTTTGGATTATCAATAGTAAAATCCAATCCTTTTTGAACAGCAAG
**CoV-bait-2366**	GGTAATATGGAACTAAGTTGGCATAATCATCACTAATATTAAAAACAGGACGCAACTTATAAATAGATGCATAACGTTCCTGGTTTGCAATGGTAGGCGCTCTTAATGGCGGAACATTGT
**CoV-bait-2367**	GGCTAAGTGTGCATAACACGCAAGTCTGTAATCAACTTCACCTGGTGAACCGCTGTAATACTTGTATTTCATATAGCTAGCAGCATACTGACGAAGTCTTTCTGCTGGAATGCTATTAAC
**CoV-bait-2368**	GGCGGCACAAGAAATTTATCCTCCTTAAAAGTCAAGACATGGTCGGCAAAAAGCAGGTCAAGAGATTGACGACAACGATAGCAAAGTGTGGTGCAATCATCCATTTCACCGGGAATCAAT
**CoV-bait-2369**	TATCCAATTTACAGAGCATGCCCGAGTCGATTACTGGGATGAAGAAGTATTTGCTTGGGAGGTTTGTTGGTCCGTTGATTTGTGATGGGATGAATGATGATGTTGTGGGGTCAGCTGATT
**CoV-bait-2370**	TATAATACTAGGCAAGTCAATTACAGTAGCATTGACAAACAACACATCACACCCCTCAATTTGAACAAAATCTGAACTAGTTGCAGCTCTAGGCTGATACATAGTTCTGGGAGTCAAATA
**CoV-bait-2371**	TAGAGATATCAAGCGAACCAAACTGTGTCAAGGTGTAAATTGGGCAATAAATAGTAGTGGAGTTAGCTGCAGCAATAGACCACATAGACATGTGTCTAGCAAGCAGAACAACACGAGATG
**CoV-bait-2372**	ATTTAGCATCAGCATGAGGTCCTGTACCTAAGAAATAGAAGAACCATCTCTCAGAGAGGTCCCTACGCTGGCCTTTGACAATACGGTAACGAGCCTGTCTGTTCCAATAACCAATTTGTT
**CoV-bait-2373**	TGGAATTGGGTAATGCCCGAAAACCAAGAGTAATGGGGAACCACACTCCCGGAATTGGGTTGAGTAGTTGCAGTCTGCTTGGGCTGATTCTTTCTGCCTCTATTTTGATTATTTAACCCG
**CoV-bait-2374**	TTGATACATGAACACTTAAGATGGTCAGTTGTGGTACTGTTACGAATTGTGTTGTAACTGTTTGTGGTTCCATGATGGTGAAAACTGTATCGCAACCTGTTGTGTTGTCCTGTACTCTGT
**CoV-bait-2375**	AATCCACCTACGGATTGAACGCTATCATTGACTAACATCGGCATGTATGGCTCGAATGTGGTTAATCGATAGCCAGTAGAAACTGATCGGCGGGCTCTGCGTGACTTTGAATAATCTACG
**CoV-bait-2376**	GGTGTGAAAAGCGGCCACATATATACAATGAGTCTGTAGATATAGGTCGCAATCTTAGTTTTAATTCCCGAGTCGTCGAGAAAGTGTAGTGTTTTTCTTGTCCACAAACCGCCATTTATC
**CoV-bait-2377**	TATGCAGCTTCAAAACCTGGGAACTTGCACAAAACACCATCAACCAAACCACAATGTAATTTGGCGTCAAGTGGTAATGATAAAATCACAGATTTATCAATTTCGATGTAGGAACCTTCA
**CoV-bait-2378**	AAAGAAACAGACACACGTTCATGGTTAACATTGTCCTCAGTAACATTGACAGGAATAGAACAAGCAAAGAAATTTTCAATAGTTTGCCTTTCCTGGTCAGTATATACGAAGACATTCAAT

**CoV-bait-2379**	TATTGTGTGGAGTTTAGATCATCAATTTGAGAGAAGTAGAGGTCATATGTGTCAGGTTCACCTAGTGTGTAGGCTCTATGTGCTAATGCTTGGATTGGTTTGAGTGAGTCTGGTAGATCT
**CoV-bait-2380**	GAAAACATTCTGGTACTCGGGATCGACATGCTTCGTAAGTGGGTATGCATCAATAGCAAGTGACACATACCGCTCCATCATAAGAGTACCATCAGTTTTCAATAAGTCATCCACAAAGCA
**CoV-bait-2381**	AGAACACCACTGCTTTGATAGCATCAAAACTATCAAGTAAAGATGTATGTGGCAAAACATAGGTAGCTAACTTATAAGTAGCAGTGCCAATAAACCAGTAATTAGTAAAACCAGACATAG
**CoV-bait-2382**	CACCATACTTGGTGATGACAATTTCCCTGACGGTAGGAGGTAAAACAGCCAAGAATTTATAAACAGTGGAGTTATCCACTTTAAGAAAACAATAATAGGGTACTTGGGTAGCACCCAGAG
**CoV-bait-2383**	TTAGTCACAGAAATTGTTGAGTTATAAAACAAGGGATTAGTTCCTTGTTTCATAGCAGAAATACGAATATGACCTTGTGGAATAAGACCTGTTAAGGGACAAGTACCAGGTCCACTTGTA
**CoV-bait-2384**	TTTGTAAACTATGGCACCTACGTACCAGTTGGAAGTAAAATTAAAGAGTACCATCATCACAAGACTAAGAGCATCTGATCCCATATAAAATCCATAGGCCACAAACAACAATGAACAGCA
**CoV-bait-2385**	TGTTACCAGCTGGCAATGGACCAGACTTATTTAAAGCCTCTGTGACACAGCCATTCAAATAAACGTCTAATGCACTAGCTAAGACGCAAAGTTGCAGATCCTCACTAACAAAGAAAACAA
**CoV-bait-2386**	AATTACTGTCCTGTGATTTAGACACATAACCATAACTGTTTGTAACATTATAAAACAGTGAAATGGTAAATTGTCTAGTGTCAACACAGAAAGAACTAAACCCATTGATAGTAGTGTCAG
**CoV-bait-2387**	GTACGGCTCTAAAACCAAAACCACGTGCCAAAACTTCAGGAAATATGACATGGCGACCGTCTTCCATTTTGTAATAAGTGTTAGGTACCAATTCACTGTAGGTACGAGCACCTTCAACTA
**CoV-bait-2388**	GGAGAGGCGATAGAACCTATCATTATGCGTACAACAACCAACATGCTTAGAACCTAATATCATGGCAGAAGCCATTCTAATCATATTAGGTAAAGCACGGTCACACTTAGGATAGTCCCA
**CoV-bait-2389**	ACCACAGCCGTCGCCACTGGCCTGCAAATCAGAAAGCTTAATTTCAAAGAACTTCAGAGCATCAAACAGCTCACGATCGCACATGACACAAAGAATGCCCTTCTTGGCTCTTGTGATTGC
**CoV-bait-2390**	GCAACACATTTAAGCATAGCATTGGCTACAATGGGTCCAAGTTTTGCATAAACTTCATGTATGGCTTCATTTTCAACTGGATCAAACCAATCTTTATTTTCAAAGAATTCTTCAGTGCAA
**CoV-bait-2391**	GACTTTCTGGGTGGGTCCTAAGACGACTGTTGCATAATTGGCTGAAACGACACCACTTCTGCGCGGTCCCTTAATCTTGAAGGAAACGAGCTTAACAAGCGCATTATCGAGCAATACATA
**CoV-bait-2392**	GTCACCTTGGGAGCCCTTATCTGGATGAATTCCTCATGATGACCAATAGTAAAATTGGTTGGTATTTGCATCTCATATAATCCACCTACGGATTGAACGCTATCATTGACTAACATCGGC
**CoV-bait-2393**	ATAAAGTACCCACTTTTAGGTGCTACTCCAACATCACCAGCCAAACATAAGCCAGGGCTCACGTAAGCAGTGGTGTATTTAGTTGGTTGGTAGCTGAAGTGAATAAAATATAATCCATAT
**CoV-bait-2394**	CCAGTATACCATACGTGAGATAAAGTTTTAACGCAGGGTAGATTAATTCCCACGTGACGGGAGAAGCAACTACGCAAGGCACTGCCTAGGCGGAAGTGGGTTTTGGGGTGGGTAGCGCGG
**CoV-bait-2395**	TCTGTGGCTAATTCATGCTCGCTATGTTTTGTCTCTGGAAGAGGTTCAACAGAAAGAGCTTGAAACGAGTTTTGTGTAGTCACAGGTTTAGAGGTGGTTATACAGCGCATAGCTGCCATG
**CoV-bait-2396**	ATCCGAGCCATATGTACCATGGCCATTTGATATATTGAGAGTTAACTCCAAGCTCTTGGAGGTTGATCAATGATGCGTTAAGCTCTTTGACTACGTCATTTAAGATTTTAACTTCCTCAC
**CoV-bait-2397**	ACCATAGTCCACATCAACCAACTAGATTGTGTGAGGTAGAGGCCAAAATAGACAGCAAACCATTGGAAGATTGCAGTCGCCAACAAAACCACAAACATCTTAGTGTAAAGCAAATACGCC
**CoV-bait-2398**	CATTTGATGTCAGACATCTTAGATTGCACATTAGAAACCTTAATGCATGGCACACCACCAACACCTGACAATTTGATGTTCATTTTCAGAGCATCCCAGCTGTTGGTAGGGGGAGTTAAG
**CoV-bait-2399**	ACATTACGCATTCACCAACTCTGCAGTATGTCTCTTCCAAAAAGCGTGTAATTTTAAAACCAAAACCACGCATAATGACCTCGGGCAACATAACATAATTATTAACATCAGAATAGTACC
**CoV-bait-2400**	TTACCATCAGATTTATCAACACGAGTCCAACGTAATCCGTCATGATCACTGACAACTGCCAAAATGACATGTTTGCCTTGGACTGTGTTATAATATGCTAACGCTGGTTGGTCGCATATG

**CoV-bait-2401**	GGCGAGACATCTGGTCATAATAGCATCGCTACTAGCCACATGTGCTCCTGAATGGACAGAACAATGATTGTCATGATTGCTCTGCAAATTTCCTGAATAACCCCATTGCTGTACATCTAC
**CoV-bait-2402**	GCGACAGACGAACCTCTTCACTCTGTAAGAGTTCTTTGAAAGCACCAAATGATTTTGCGGTTAGCACTCCGTACCAGCACGCACGGTAGAAATGATAGGACAAGCCCACAATTCCAGGAT
**CoV-bait-2403**	AACTGTCGCCGTACTCACTAAACTAAAGGATTACAGCTTCCCTCAGCTGGGACGTTAGATTGCGGCATTTGAATCCCGCCCGGGCTGCCGCCTGCCGGGAGATCGTTCGAGCGAAAATAA
**CoV-bait-2404**	TAGGAATACTTATAACACCTTCAGACATAGGCTGAATCTTAGGCTGTGTATTTCTTATGCCAACAAGACCAATAGCGCCATTTTTACAAACGCCAATGTTAGAATAAACGAGTGCTGGTT
**CoV-bait-2405**	CCATCCAGTCTGTATGTGACGAATTGGTCTTCAGTGTCATATTTTATGTCTTTATACACAGCGTCAGCAACAGCTGTTGTTGTTTCTGACATTTTTGTTACTCGTGTACCGTTTACACTG
**CoV-bait-2406**	ACAGTGTTTTTAAGCAAAAACCCTACTGGATCATTAGTGCATACATTTGGTATTTGCACATATTTGCCTTTATACTTACACAACCCGTTATGACTTGGGTGGGAAATGTGACATCTACAG
**CoV-bait-2407**	TAACATAATCATCACCACCAACATGCTGAAATTCTTGATCATAAGCAAAATAGTAGTAGTCCTGAGGCAGCTGATGACGATCCATTTCAGAATAATAATGAACTTCAACAACCCTTTTCT
**CoV-bait-2408**	CCAAGGAGATAACAGTAGTATCTCGGCAAAAGATGCCGGTGCCAACAATACTTTTAAAAGTGTGCATCAAAACTTGAGCACACTGAGCACAAGCACCATATGGGAACGGTTCCAAAGTTG
**CoV-bait-2409**	TGACAATGCCAGCAGTTCTTAAAGCACCTACTTCAACTTTAACGGTGCTTTGGTTTTTAGACTTCCAGAATGTGAAAGGTAATATTAATAACATTTTGAGCAGAACAAAGCTCTGTCCAA
**CoV-bait-2410**	GTGGCTTCTACATCTTCCTCAAATACTGCACAAAGCAGCTCTTTATTTGCACTTAATGCTTGACTTTGTTCAGATTCAGAGTTAACCACAGCTTCAGTTTCCACAGCATCCACAGCTTTC
**CoV-bait-2411**	GCTATTGCTTTTGCTACACCACCACCATGCGCCATATGGCTGTTGGCGGGGTTCACAACAACATCAGCAAAAACCTTCTTAGAAACGTCTATAATATCACCTTTTACAAAACAAACATCT
**CoV-bait-2412**	AAAAGTACTGAGATGACACCACCAATAACAACACGTTAAAGGTTGAAGTGTACAAGACGTAAGCAAGTGCGAATTCCATTACAAACCACACCCAGTCTATGTTTATGACATAGCTGGTAA
**CoV-bait-2413**	CCTTTAACTCCTGTCTTGCGCACCTTAAGAACATAAGGTTCCAAGCGCGCAAGAGCTTTGGGTGGCAGCACACTCTTTGGTACTACCCAACCCATAAATCGACCTGCACCATAACCAACA
**CoV-bait-2414**	GCAGCAGCACAACCATTAGGCAAAGCTCCAACAGCTGGAGCCTGACTACCAATAAAGAATGGCACATTCATGCTAAGCACGTGAACAGTTTTAGGTCCACAACTACAATCTGTAGTAGTA
**CoV-bait-2415**	TTGGTAAGGGGCACACCTTGTCCTTGTTCAAATCCAAATTCTTGCCCTTTTTGAAACTGAGTAATTCCAGAGAACCAGGAGTAAAATGGTACAACATTTCCTCCTGAAGCCTGGGTATTA
**CoV-bait-2416**	ATACCTAACATGGGGCTTCATTTGATCATACGCAGAAGCGCCAGGCAAAACAGTAGGGTCATAGCAATAGGGGTTAATCTTACCCTCTGCATCCCTGAACAATGTGCATTCAGATGCTAA
**CoV-bait-2417**	ATAACTGGTTTACCAAATGTTATACAACCCTTTTTATAGCGCTTGACATACAAGTCATCATCTGCTAACACAACATCACCCGTTGCATCAGGCCAGACGGTGACCTTATATTGAACTGAA
**CoV-bait-2418**	CACCATAGTAGGTAGGTTATATTTATAATAATTATAATCAGTGATAGCAGCATTACCATCTTGCGTAAAGAAGAAGTGCCTCAAATCGACAGAACTACCTTCCTTAAGCAAGCCTTTACT
**CoV-bait-2419**	GCTTTTGCCAAGGTGAAAGAAACTGCCGCCTCCTTCGCATAAACAAATTTGTGCCCCAAGAACTTGAATGGTTCACCTTTTTTAAGTGCTGCCACAAGTTCCGAAACATTAAAAGACGAA
**CoV-bait-2420**	TCTACTTTTGCGCACTTGTCACATGCTGTATTGTGTGTGACAAGGATTTCACAATCATTGTCCATCAAATCACCAAGTTTATGCAACATTAATTCAGCATCACCTGGCTCACCTTTCTTT
**CoV-bait-2421**	CAGACAAGACAACAGTCTTCTTAATCTTTGGAGTACGTGCAACCTTCATGGCAGTACCATCAGGAAAAGTATGCTCATAGGTTGAACAGTAAGTAATAGACTTAATAGATGTTAAATTCT
**CoV-bait-2422**	TCAGGGGGGGCAGCACCTTGTAGCCGGCCACGTACTGGGCGCAGATCAGGTCCCGGGCGCTGGCGGGGCCCTGCTGCATGCAGTCGTCGTAGCCCTGCATGTAGCCGGGGTCGGCGATGG

**CoV-bait-2423**	GGATCTGTGTGTTGAAACGGTACCTGATGTCTGCTTTGCTGCCTTTTCTATATCAGAACTGAACTACGTCTGGACAATCGCTGCATGTAGAAGGGTGCTCTGCAGAGTGGGTCCAACATT
**CoV-bait-2424**	TGGGCGCCATACGCTTCCAAGCTATTGTTGACTCTGTCCACCGGCCGTACCAAGGGCACCACAGTTACTTGCCAGCCCCAGTGGCTGGTAGACGGCGTGCATGGTAATTTTACGGAACGG
**CoV-bait-2425**	ACTTAACACCACTATACTTATAACTAAAACTAGTACTAAACACTTTAAAATTAGGCACTATACATAACTTATTCCTATAAGCAGCAATACGACTTAAAATATTGGGAGAACACAATTCCT
**CoV-bait-2426**	CCCTTAACAAACATCTTAAAACACTGTTTGCTGTGGTCTTTAACTGGTTTAAACTTGTTTTCATAAACAAGTTCAGATACAGTGTTAACAACCTCAGCAGGGCACCGATAACACTTATGC
**CoV-bait-2427**	CCACCTGCTGCAGGCTCAGCATCTCGTAGGTCAGGTCCAGCAGGGTGGTGTTGATCTGGGTCAGGCTGCCGAAGTTGGGGATGCTGGTGCTCACGTTCTTGAAGAACTCGTCCAGCTCGT
**CoV-bait-2428**	ATCCTCTAATAGTGTAACAGAGAAAGAATCCAAAACTCCTTGGTTAAGTGTCTTGTACAAGTGCTTAACCCAATCCAACATAACCGTGAAAACACGCCTGTACTCAGGATTATCGTGTTT
**CoV-bait-2429**	CATTACTTTGTCATTAACTACGGCAACAGGTAATTCACCTTCTACACCAACGAATGACCCCTTCTTGACAACGTTGTAGGCTATGTTCTCCAAACCTTGCACGTTTGAATTCTTAAATGT
**CoV-bait-2430**	CCATCTTTCCATGTGAAAAAGTCGTGGGTTGCAACGGCATTGCTGTCCTTCAGCAATTCATATATGGATTGCTCGTGTTCCATAACTGAAGTAGTACAGCGTTTTATAACAAAGAATGCG
**CoV-bait-2431**	GGGCTTTAAGAGATCTTTAGGACTTTGATGTTCTTCTTTTACACACTTATCCCTAGCGGGCACAGTTGGTTCAATTGTGGTTTTAAATTCAGCTGTCATTACATTTGCATTAAGAAACAA
**CoV-bait-2432**	TGGAGGCTTTCACACAAAGTGAATCGACCGCTGCATGAGAACAAGCTGTAAACACTATACGTGCACCTGGATAGTACAAACCTAGCCCTATAACACAGTGAGATTTACCACTACCTGGAG
**CoV-bait-2433**	CAATATGTATACGGTCTAACTCAACATCAAGATCTTCTGTAAGATCCCAAGTTGCCTTAGCACGGGTAAGACACTTACCTTCAACAAGTTCTTTCCAGCAATGTGGCACAGTCTCAAGGT
**CoV-bait-2434**	GTAACAAGTAGCACGTTTACCACAGTCACAAGTTTGCACTGGCCCAAGTTTAACAAAGTACCTCATTGTAGTGAGTTCCAAACCACCAGACCAAAGAACAAATATGAGTGTATCTGACAA
**CoV-bait-2435**	ATGTGAACAAGCTGTAAAAACAATTCTGGCACCAGGAAAATAGAGCCCCAAACCAATAACGCAATGAGACTTACCACTACCAGGAGGACCTTGGATTGTGGTAAGCTTCTGCTTACCTAT
**CoV-bait-2436**	TCAACAAAACTTTGATCCACACTTGATGATCGATAACAACAATCATACAACTTACGCTGCAATTGCTTAACTTCTATGTTGTTACATGTATTACTATCAACACTCAACAAGCGGTTAACA
**CoV-bait-2437**	GACAGGACCATTGACGCCTATTTGAACAGCAAGATCTTTATCTGTTTTAAAATTGTCAGACAGAGACATAAACGTAGGTGCATGTGATGGTGGAAGGTTATCTTCGCCTTTATAACAATC
**CoV-bait-2438**	ATGTAGATCTAATTCAGGTGTTGTGTAATTAAGGTTATATTGTTCAAGCATATCAGCGACAGTTTTGTTAATGTCAATATAATCAATCACAATCTCCTGAAATGTCGTATATGTAGTGTT
**CoV-bait-2439**	GCTGTGTTTACCATTTTGTTGGTGACGGGCTTGTGGAGCATGCGAAAGATTGATGATGTTATGAAAGATGAACCTCCAATTGTACGAACTCTTGGTTTCTTTGTCCGTTGATGTTTAGTG
**CoV-bait-2440**	TTCAAGAACACATCTGGGCCGATGGTGCACATTAGGCGTGTGACAACATTATAATCAGCGGGTTCCAAAGCACCTTTAGTAATTAGGGTACGTGGGGCTGGTAATTGCTGAGGATCACCG
**CoV-bait-2441**	CCTCAGTTGCAACCCATACGATGCCTTCTTTGTTAGCGCCGTAGGGAAGTGAAGCTTCTGGGCCAGTTCCTAGGTAATAGAAGTACCATCTGGGGCTGAGCTCTTTCATTTTGCCGTCAC
**CoV-bait-2442**	GTAGCATTTGGCAGCGTTACCGTTGGCAACGAGATCACTATCACCAAAGTTAGCTGAAGCACTTCTAGAACCGTAGAAATTTGTCACATCTCCCTTACCTGCAGTTTTCTTCCAGGTGTG
**CoV-bait-2443**	GACCAATCTACACGTTTTACAAAACATTCATGTACCGCTAAACAACGCGTCATTATCGCATCACACGACGCTACATGCGCATTACCATGTACTTGACAATGTTGATCATGATTCGATTGT
**CoV-bait-2444**	TGCGGCTGGTACCACACAAACAGCTTGTACTGATGACAATGCACTTGCCTACTATAACAATTCGAAGGGAGGTAGGTTTGTGCTGGCATTACTATCAGACCACCAAGATCTCAAATGGGC

**CoV-bait-2445**	ACTACATCTATAAAACATTTTACACAATCTTTTATATTATCCGACGCTACTTGTATTTGACCTTTTACTATATCAAATACACCCGTTATTAAAAATTTTAATATTTCCCACGCATCTTTT
**CoV-bait-2446**	GGCTGTATTAATGCCAACCAAGTAATCGTTAACAATCTGGATAAATCAGCTGGTTTCCCATTTAATAAATGGGGTAAGGCTAGACTTTATTATGACTCAATGAGTTATGAGGATCAAGAT
**CoV-bait-2447**	CTAAAAAATATCCAACCATTCACAAATAACCACAAATAATCACAACACCAATAACAAATTCACTTTCCATAAACAATCTAATCACAATTATCCCCCAAAAAAACACATTAAAAAAAATAT
**CoV-bait-2448**	TATTTAACGCATCATTATCTAATTTACGTAACATCGTAAATAACATCGTTTGCATCGCCGACGTTACTTTCGCACGTTTATCTTCCGAACGCGCTTGTTTATACATTTGCGTCATCGCTT
**CoV-bait-2449**	GTCTGACCTGGTTGAATACGGACAAATTTATACTTTGGTGTCTTAGGATTAGAAGTATCAACTTTAAGCCTAAGTAAACAGTTTTGCATGGAATGGCCAATAACACGGAGTTGAACATTA
**CoV-bait-2450**	TGAGAATTGGTCGACGGCCCGGGCGGCCGCAAGGGGTTCGCGTTGGCCGATTCATTAATGCAGCTGGCACGACAGGTTTCCCGACTGGAAAGCGGGCAGTGAGCGCAACGCAATTAATGT
**CoV-bait-2451**	TAACTTGGTTTAAAGGATTATCACAATAAAGCAAACGCTGGATGTTGGTAGAATTAATATCAACCATAACATCAACAGTGCTAGCAAATGCGACAGTCCAAAAATCTCTGCCATCAACAG
**CoV-bait-2452**	TTTCGGTAAGTAGCAAAAGACTGGACTTCCTCTACTTTATTGTACTCAACATCCTTAAAACACACTGTGTTAACTGTATTCCATTTAATTTGACCACGGCCATTACCATCAACACAAACA
**CoV-bait-2453**	AATATCCCTTTTGCTCTGAAGCTGGGATTCCGTTGGCAATAGGCACTCCCTGTCCTTGTGCAAACTGAAACTCTTTTCCCTTCTGAAATTGGGTAATGCCCGAAAACCAAGAGTAATGGG
**CoV-bait-2454**	TTAAATTTATGTTCAGGTGTGTTAGGGTTCACTTGATTAACCTTAAGCACAAGATTTACACCCTTATATTTGGCAGACACAACACCCAAAAACACATTATTTTTTGCTATAGAGAAGTTA
**CoV-bait-2455**	AAAACGACAGACCACAGAAAATTCTGGATACATGTCTACATTGCAATTCCAAAACAAGCACAACCCATCAAATTGGCCATGTGTTATATAGTCATACTCCAATGTCTTGACATTAGAATT
**CoV-bait-2456**	ACTAATACACCCACCGTCATAACATTCAAAATATTTGCCAACTATTTTGTAGACAAATTGGGCTTGGCAAATATCAAGTACTGTGACTCTATTGTAGCGGTAATAATTGAAGTCTGTCAT
**CoV-bait-2457**	CCCTACGCATTCAGTGCCGCGAATTGCAGTTAAACTTATAGATTGTTGCATACATTTTTCTCTTTATCGTAAGATTGCCCGTCGGCGAGACGATCACGCATGATATCGAGACATAACTGG
**CoV-bait-2458**	ACAGATACTCAACAACGGGAGTACCTGAGTCATCAGCAACGAAAATGACATTGTCCTTCACGAAAGGTTTATTTAAACCCTTACATTTGACAATTGTCATTTCCTGTTGAAGTGCTACCA
**CoV-bait-2459**	CAAATCGCAAACTACTAGACTTCCAAGGTGGTTTGGGAGTTCTGATACCGGTAGGAGTGTGATGTTTGATGTTACTGAGTATTCATGTAGTTGTTTTGTGATCTGGTTTTCTTTTTCCAC
**CoV-bait-2460**	ATATTTATCCAACAAATTAAGCAAGTTTTCAGTATCACCTAAATCACCCTTACGCGAACGCGTAATATGGTAAAGAAAGTGAACAAACTCACCACAATCACCTGTAACATACTTATTCCA
**CoV-bait-2461**	CGCCGCCATTAAGGTAGCGTCTAATGTTTCCCAGCGCATGTTTCCCGTACGGGTCGTCCTGGTAGAGGTCACGTGAATCAAACATGACATAATCAGGCACAAGTTCCCGCTTGGGGTAAA
**CoV-bait-2462**	CCACGTTCAAAAAGCTTAAAACACTGTTTGCTCTCAGCATTAACAGGCTTGAACTTATTTTCATAAACAAGCTCTGAAACAGTGTTAACAATCTCAGCAGGACATCTAAAGCATCTATGC
**CoV-bait-2463**	TAAAGATTATTCGGCCCGCTTGCGCCGCTTTATTACCCCACCCTCGCCGTTACGAAGACCTTGAACTATAATGGACGTAAGTTGCATCTGCAACCAATTGAAATATCCTCTAGGCAGATC
**CoV-bait-2464**	ATCCTGAGCATAGAAAAAATGCTTAAAAGATATAGAACTACCCTCCTTGAAAAACCCACATTTAATGGCGAATTTATAAAAATCCTCATTAAACTGTCCTGGTTTAACCGTCTGGAAAGT
**CoV-bait-2465**	CCATCACTAACATTTTTAAAGCCTAACAAATCACCTGATAGTGATGTGTAATATAAGCCACTAAGTAGCGTACTGTTAGTTCGTCTAATAATACCAACACCAGTTCTACCATATATATTG
**CoV-bait-2466**	AGCTGACAGAAGCCATAAAGTTTCTGTTTAGACTAAATGAAGCACTTTCTCACTATCTGTGAGAACCGCACTCGGATTACTCACAGCTGAGTAGTCGCCGTGTTTTGACCGTACATAGAA
**CoV-bait-2467**	TTAACAAGATGGTTACCAAAGTTTAGTAATTTACCGTTATTTCTAACTAGTAACTTACCACCTTTAATCAAACCTTTAACCATAACATTTATATCACTGTCTTTCAAACTAACAACCAAC
**CoV-bait-2468**	AATGGCCGGTGGAGCATGGGTGAGAGGATAGTATGTCTGATTGATGGACAGAACATGAGTACCTTTTGTGGCAAGAAGCATGTCTTTGTGATAGTAAAAGGACATGCATATCTGTGCAAC

**CoV-bait-2469**	CAGGGTTGCACTCATAGAAAGCATGAGCACCTTCAATCCTGAAAGGTTCAAAATTAGGCAAAACTGGTTTTTGTGAAACTTGTTGAACATCTTCCTGGACAGGAGTAGTATCAACAAGTG
**CoV-bait-2470**	TTAAGTGTGTAACCCGTATTAATAGCTTCCGCATATTGCAGTAAACCATTATCTTCATTCTCATTAACACCACTTAATATAGCATTAACACTCTGGCAAAAAGAACCATACTGCTGCAGC
**CoV-bait-2471**	CGAGCACAATGTTGGCGCTACAAGAGAAACAACATTGTGTGAGGTTAAAACAAAAATGTCTCCTACAGACAATTTAGCAGTAGACGTTGCCCTATAATAGACAACATCCTTACCTTCACC
**CoV-bait-2472**	CATACCACCTGTGAGTGCAGCAGTATACTGGCCTAAAAGTGAAGGATCAACAACACCTGGCAAGACCATGATTCCATTATAATACTGCACGCAACCAGCCTCAGCAATAGTATTCAGCAT
**CoV-bait-2473**	CTGAAGTGAGAAGTAGTTTTCGCTGTAGGTGTTTTGGCCGATTGTAAATGTAAATTTATTATTAGCAAGTTCAATTTTGTAATTTGTGATGGTTGTGTTACTATCGTGAAGAGCTTTAAG
**CoV-bait-2474**	TATTTTCTTCTACAAGAGAAACTTGTTCAACACAGTCTTCAACATCGACAACTGACACAGTTTCTCCCTCTACAAATGGGGCATTTTCGGCATCCACGTCTTCGTTGTCAGAAATCAAAG
**CoV-bait-2475**	CCCTCCACAGCAACCAGTGCAAAGAAAGATTGTTATGAGGATTGCGACAAATGCAGTAAATGCCAGGGCTATGGCAAGCCATATATACCATGGCCATTTGATGTAGGTTTCAACACGGTT
**CoV-bait-2476**	ATGATTACCACAGAAGTATGACATACCTCCGAGGTACAATTTAGTAACATCTGCCTCGCCACAACCGGGCTGTGAGCAAATGTATGGGTTTATAGACAAAACATTTTTGTGGTCTGTATG
**CoV-bait-2477**	TTAGTAGACCCTATGAAAACAACAGTCCCTACAGCATTTTCATCACAATCAACTGCAGCAGGGCCATCAGTAGCAAAGAGCAATAAGTATGGTAGTGAAGCTTCTATTACTTCATCTATA
**CoV-bait-2478**	CATCCTTGGAAGTAGACCACTCAGCCGAGCGGGCTTCACAAACCAGATTACGGATGGACCGCAAAGTGGGTTGAGCTCCAAAGACATTCTCAAAGTCAGTAAGAGAGGTTTTAGCACTAA
**CoV-bait-2479**	ATCATCAAGCAGTAAATCCACAACAGTGCACACTTGCTTGTAAGAACCATTGTCTGCCACTACAAAATAATTTTGCATGACATCAGAATCTGAATTAGTAACAGACTTTGCATTCAACTT
**CoV-bait-2480**	AGAAACAAGCCTACAAAACTCCCGTTAACAATTACAGCTTGAGTTGAAACTGTCTGGCACGGTTGTACACTATAAAGACTTCCATTATTACGAAAGGCCAATAAACCACTGCCACTAGTT
**CoV-bait-2481**	CAGTAAGTATTATCGCTGCGACAAGACCTCCTGTATTTGGTGGATATACACATGAAATTACACCTACTGCAATGTTAATGGGCCAAAAGCACCATAACACTATCATTTTCAGTATGTAAA
**CoV-bait-2482**	GTTCTTCAACAACGGCTCGTTCAACCATTGCCTCACAACTACCAGTAGAATCCGCAACTAATTTCTCAAGTATGACTTGAGTAGATTCCTGTTGAAACAATTGTTCTTGTGCATCAACAG
**CoV-bait-2483**	CCAGTGAGATTAAGATAAGTGGCATTAAAAACATCTAAAGGAAGACTTGGACCAGTTCTATTGGGCAGAGAAGCTAAAATCTCATCAAGTGTTTTGTTAACATCGATGTAATCTGGGATT
**CoV-bait-2484**	CGATTTTAAGCTTATAATTAGTAACAAACAGTTGAAGAACATTCATACGACCATTAATGAGTCTGTCCATCTGAACGTCTGCTTCAAGTTTGTCAAGACGCTGTGATATGACCTTAAAAT
**CoV-bait-2485**	ATGTTGATTTCTTTGGTTTCTTTGGCACCACCCTCTGAGCTGTCACCACTATCATCAACATCATCCACTTTTAAAACATCAAATTTATTATCATCAACCAATGAAGGTCTATTAAAATAT
**CoV-bait-2486**	GTGAGAACCATGATTGTTTGCAAGTGAAGAATCTAACACTATATGTAACAAACACAATAAAACAGAACACTGTGAGCATGACACCTTGCATGTCAACAGGCAAAAACATAGTGTTAGTAT
**CoV-bait-2487**	TGTTGTTCATTGTAGAACCAAAAACCCATCCTCTAATAACATTAGACTTTTCAGTCGCAGCAAAATAAATACCATCCTTGAAGGGTATTATGGGATTATCAAAATTTAGGCCAAACGTTA
**CoV-bait-2488**	TTTAAACAGTGCAAAGAAGTAGATAAAAACACTGGTGAAAAGAAAACCCGTCACATTTGAAGCTTGTCTAATTGGAACTACATTGAGCTCCAACTCTTGGACAGCATCCAAAGACAAGTT
**CoV-bait-2489**	TCTTCATCGTCATTGTCACCAGTAACAACATCTTCATCGTCATTGTCACCAGTAACAACATCTTCATCGTCATTGTCACCAGTAACAACATCTTCATCGTCATTGTCACCAGTAACAACA
**CoV-bait-2490**	TCATGGACAGCTAAGCATCGAGTCATGATAGCATCGGATGATGCGACATGAGCACCCTTATGCACGCTGCAAATAGGATCATGATTGCTAGTTAAAGATCCTGTATATCCCCACTGTTGA

**CoV-bait-2491**	TTGCGATAATTGGCACCAGAAGTGGGATAATAGCCATTGAGCAACAATGTGGTATTTAAATAAACACGATCAAGCACATAATAAGTGCCAAGACCGTTAGTAACATCAACAGTCTCTGTA
**CoV-bait-2492**	TAATGCCAACAATTTGATGTTGCGAAAAGACATAAAGCTTCTTGCCATCAAGAAGCTCAACAGTTCGCAACAGCTGTAGGTCAGCTTCTTGCCGCCCACGTATAGCTAGATACAAGTCGA
**CoV-bait-2493**	CCTCTTCAGTCAATTTAAGCATGGTCAAACTAATATGGGGGGCCACCTGTTTCTCCCCAGGAAAGCCCTCTTGTAACTCATAAAAAGCCTCAAGAAAATTTTTATTTACAACAGGTAAAG
**CoV-bait-2494**	GCAACAAATTCTTCAGTTTTGAACACACCCATCTTAGACAGCCTAATTTGCGATATAAGTAGATGTAAACCACCAAGTGTGGTTTTTGAAACATCACCATACACCACATGTTCAAAATTG
**CoV-bait-2495**	ATTGACATTACAACCATTAAAACTACACACATATGGTGTGATAGACATAATGAATTTGTGCTTTGTTCCCATAACATGGTCATAAGCACATTTCGTGCATAAAAGTGGTCTCCTAAGACA
**CoV-bait-2496**	GACTAAAACGCACTGTCAAGAAAAGCAACATGTACAATGTATAAAACATGACAAGCAACTTCATGACAAACTTTACAGTGCCCCACTTCGAATTTAAATGTTTATAGGCGGCTTTACTAT
**CoV-bait-2497**	CTGGCCTTCCGCTCTTGACTTATGGTACGTGAGAGATTACTACTTAGGCGATGCGGAACTCGTATAGCCTTTACAGCCCCAGTCACGGATCTATGCTCGTCTAGGGTCGTATGTTGGTCG
**CoV-bait-2498**	AATCAGCTGGCAAGGTAACAGATGGTGTCTTGGATTGAAAACCAACCACCCAATCAGTTGTTAAATTATACTCACAATTTAAACCTCTATCACAGCCAAAAGAATGAAGGCGGTAAGGTG
**CoV-bait-2499**	TGAAAACCACGATTGTTTACATGTAAAAAATCTTATAGTGTATGTAACAAACACAACGACACAAAACAGTGCAAGCATAATACCCTGCATGTCAACTGGTAAGAACGTGGTGTTAACATT
**CoV-bait-2500**	CAATCCTCATGAGAGGAAATATTATGTATCACAATACTGTCCTGGTGTCCTCCATACTCATCACAACAATTTCCACACTTCTTAAAACAACATGAGCCACAACCTGTGCAGCAACATATA
**CoV-bait-2501**	ATCTGCAATGGCAAATTAGTGCCAACATTAGGGCCACAAGCATGGGCTCCTTCTACATCGAAGCCTATCCATCCTCTAACTTCCTTTATAGCCTGCTCCTTAGTTATAAAGAGTTTGCTA
**CoV-bait-2502**	GAGAAAAGTTGTCTTGCTTAAACAATTGTATGTTGGCATCATCGGAGTTTGGAACTCTAGATGAAATATCAACACCATTATGGAACACCACACCAATCTGGTCTTGGAAGGTGCGTTCAT
**CoV-bait-2503**	GTACTCGCACCAATACCTGTACTCCCTTGGCTCAAATTAGCGCTGCAGGATTCAACTTCTGCAACAACATCATCAACTTTCAAAACATCAAACTTATTAACATCAACCAAACTAGGTCGA
**CoV-bait-2504**	ATTATATTTCCGTCTACAATAGCCCTGTCACAGTATGGTCCTAAGTAGTTAACACCAATCAGAAATCCTTCTGATGAAGAGGTATTAACACTTGTACAAAACACAGTCCAATACTCAAAT
**CoV-bait-2505**	TCATGGCACGCGTGCGAACTATGCGCACCACGCCCTCACTAACCACTTCAGGAAATCGTATATAACCATTTGAATTAGCCAGGTTATAACGGACATGAGGAGCCAAAGAACCATACAGAG
**CoV-bait-2506**	ACCTTACCTATCGTGTAGAAAGGTGTTAAGAGCTCTTTAGGACTTTGACGTTCTTCGACAAACTGCTCCTTCTTATTGGTTTTGGGTTCAGCTGTCATTACATTTGCATTAAGGAACAGC
**CoV-bait-2507**	AAGTGTGAAGGTATTATTTGTTACCTTCATATTAGGTGCAAGAGCACAGTACTTTTCTGTGTCTTTAATTTCGAGCAACATAAGCCCGTTAATACAAACTGGTGTACCAATCAATGGGGC
**CoV-bait-2508**	ACACAATCCCATTGGGTGCTGATTGTGGAATTGAAAGAACATGTTTTCCCAAACCACAAAAAGAATTCCTATTTGATTGTGAACGCACACATTCATTAACTTTCTGTTGAGCAAGTTGGC
**CoV-bait-2509**	ATAGTACAATCCAAGTCCAATGGAACAATGTGACTTACCACTACCAGGAGGACCCTGTATTGTAGTTATCTTTTGTTTACCAATAAGTTGGTAATATGGAACCAAATTAGCATATGCATC
**CoV-bait-2510**	CCAGGAGTAAAAGCAGACTAAACAAAGCCTGCCAATAAGTGTGCAACAAATAATAGTTGCATCTAAAAATGCACCACAATAATATAAAAGTGGGCAATAAAGAACAATGACAGCAAAACG
**CoV-bait-2511**	CAGCAATATTCTTACAAACATTGCTGTCAACAGCTAACAACCTATTAACATTTGCGCTAACAGCCTGAAATATATTAAACACAGAATTGGCATATGCTGTACTAGCATCACCAGAAGTAG
**CoV-bait-2512**	CCACTTTAGTAGAACCATTAACAACAGTTTGAATGGGAATACGAGTGAGGCGTGCACTTTTAGAGCATGCAACACAACTAGGTTTTTCACAACCAAAACAAACATGCTTAAGAAAAGCCA
**CoV-bait-2513**	TTTAACTCCTCAGCAGAGAAGTTGTCTTGCTTAAATAAATGTATGCCCTCATCTGCATTAGCAGGGCGTCTATCAGAATAATCTCTACCGTCACAAAACACTTTACCTATTTGTGCCTTA

**CoV-bait-2514**	CACATCAGCATATTTACCGAGTGTTGTGATTGTGTAATCTACACCACTCTCAATAGCTAACAATTGTGGCTCATGCACTATAGTTTTAGGAGTTGCTCCAACCTCACTAAAAACACGAGT
**CoV-bait-2515**	CCTAGACAAGAATGTGTTGAGATTGGCGCTCACAGCTTGACATATGTTAAACACACTATTAGCGTACGCTGTAGTAGAATCGCCACTGCTAGTTCCACCAGGCTTAACATAAAACCCGCC
**CoV-bait-2516**	ACTCAACCTGCACTGAAATTGTAAAATTACTAGGGATTGTAATGTTACCAGTGCTTATAGGTTGTGCAACACTATTACTTTGTGTAATATTGACAAATACCAAAGCACCATTTTCACACA
**CoV-bait-2517**	ATTCCAAAAGGACATGACTCTTAGCGGTCGCGTTCGATAAGCGGCGAGGACTGAGTTGACCACAGGGCTGGCGCACATTACTCACATATATACCGAAGGTCTCAGTTTTATCCATCCACA
**CoV-bait-2518**	CTTGGTTAGAACCTCTCGTACCTAGTCCTGTAGGTACAGTTTTAGAACCATTCTTGCCAACCCAATAAACGCCGTCAATGCGTTCTTGGAATTTGGCATTCTGATGAGGTCCAGTTCCTA
**CoV-bait-2519**	GGTGGTGGCGGGATTGCGACGGAAGTGACTACTGTTGAGCCATGCTTGATCTCGAAGGTTTTGTTTGATGTTATTGTAAGTAAATCAAGACCTGTTGGTGTTGGATAAACAGCGAGAATA
**CoV-bait-2520**	CTAGATATATGATCTTTAGACAAACGTTGAAGTTTGCCTTTAGTATGAACATCTAATGCCTTGGCAAGACCACCACCATGTGCAAGATTCTCATTAGCAGCATTAACAATGAAGTCAAAT
**CoV-bait-2521**	AGACCATGTTAAATCCATGGTCTTTCTCCACAGAGACTCAGTAGTCTCTGCCCAGTCAGATGACTTAAGAAGCTCTCGAAGAGTTCTTAAGACTGGTTGAATACCAACCAGATTTTCAAA
**CoV-bait-2522**	AAAGCTTGCAAAAACAACATCTTATGTTTGACAAAACCCGTCAAAATAAGAGAAAATAAAACGAGCAATCCCAACACTGGTGTAATAACAACAGGGTTAAACCAAAATGATGTCGTGTAC
**CoV-bait-2523**	ACACTACTATTAAAGGCATAACAACAATTGGATTCACACTATAAGACAAAAGCATAATAGCATTAAATCCCCAGCTCAATAAAATAGCACATGCAAGACCTGCATAAGTCTTGAAAAATG
**CoV-bait-2524**	CTGAACAGTAGTAACATTAACTGTGATGGGTTGATCTACTATGCTGAAGTAACAACTAGCTATGTCAGCACACTTGACATTAACCAGGTTCCACTCCTCGGCACCAGGTACATAAAGTGA
**CoV-bait-2525**	ACCAAAGCTATTATTGGCATACATTGAAAACCCAAAGCCTCGCCCCCAACCAACATCGTCAGTGAAGAACCCACGTGCCTCATGATAAGTTGGACCCCAAGGACCAGTAGAGTCACATCG
**CoV-bait-2526**	CGCTTCAATCTTGCACTCTTTGAGCAAGCAATGCAGTCTGGATTATCACAACCAAACAGCACATGTCTAACAAAAGCAATAACCTTTATAACAATAACAGTGACTAACAACTCATCACAA
**CoV-bait-2527**	TTCAGACATCATAGCACCCACAATGTTTTGCTGGTATACAACCACTTGATCTGGTGGGTGACAAGGCGTGATAGAATATATGGTGCCGTTAACAACATCTTTAAAGCCAAGCAAATCGCC
**CoV-bait-2528**	AGTGTCTTCTTGTTGCTCTTGTTGAACCTCCTCTTCCTTGTCCGATTCGGAAAGAGGCCATTCAGAAATCATAACAGGCAAACTCAAATCAGTGCCGCCTTGCTCATCGTAGATGTAATA
**CoV-bait-2529**	TTCTCTATTAGCTTGTTCAAATTTTGAACCGTATCATTGAGAGCCGCAGACTTACTCTCTAAGTCAGTAATCTCAGCTGTAAGGTTCAGAATAGTCTGATTATAAATGTCAAGAGGCAAA
**CoV-bait-2530**	CTCTAATAGCGACGTACAAATCGTTAGTTGAGACATAGGAAACAAGTTTGTCACCTACGTAAAGAAATTGGTCCCCTCCATAAAAGCACACAAATGGTTTCAAGGACTTGTTTTCAAGGA
**CoV-bait-2531**	TTTCTGTCTCATTGACAAAGATACATGCACCTAATATTCTTGAGACATCAGGATAGGGATAGTAACAGGGCTCCCCATCATATTCTGCCAACACAGTGTGTTGTGAGCAAAACTCATGAG
**CoV-bait-2532**	GTCTGTAGTACCAGAGTAATAACGATAGCGTGCGTAAGATGCCAAGTATGTGTCATATTCTGAACCTGCTAAATTTAGCATTTCAACATACTTGTCATTAGAAATAACAAAAGTGGCTTT
**CoV-bait-2533**	CGACCCCCTATCTGTCCACAATGTCTCCTTCAGCGGTTGAACCTAACGAATTCAGTTGCTCCCATGTACAATAGTATTGTTAATTTGGAACAACACTCTGATTTCCTGGCCCCAGTCTCA
**CoV-bait-2534**	GACACTGTGTGACGTCGGCATAGAGCATAGCCTTAGCTGAAAAAGCGTAAACAGAAGCTGTTAAACTTTCGTTAACTTGTTCTGTTAGTTCTTGATTTTTGACACTTGTAGGTTTCTTAG
**CoV-bait-2535**	GTACTCCAATAACATTGTCACCCTCAGAAAAAGACACATAAAGTGCGGCAAATTTCTGTGGTTCACCATAGGAACCACGCGTAACAATGCTCATAGCACAGCCTCCATTAGGTACCAGTG
**CoV-bait-2536**	CTAAATCATTAATCTCACCCGTGAGATTTAGATATGTTAGATTGAATAAGTCAAGGGATAAATTTGGTACTGTCCAATTGGGCTTGTACTGCTCTAGCATGTCTTGAATTGTCTTATTAA
**CoV-bait-2537**	CGTTGAGCTCTTCATTATATCAGTAGCTGATACCACTCCAAAGAACATATTTGTTAGCAATTGAACTAAGGTATCACCACAGTAAACACCGGGTTGTTGTATACCATCATTAACCACAAA

**CoV-bait-2538**	CTTTACCGTCACACATTTTCTCGTAGATCACAGCGTTCTCAAATGGTGGAGGTTCTGGGGCCTCAGAAAACAGTTTGAGGTCGTCACACATTTTCTCGTAGATCACAGAGAGCAATTGTT
**CoV-bait-2539**	GAGCTTTTCCTAACTTATTGAACGGATAGCCAGCTGACTTATCCAAATTGTTAACAACAACAGTTTCTGCTGGTATACAACCACCATCATAAAATGATAGATACTTATCAGCCACCTCCA
**CoV-bait-2540**	GCTTCCTACCAGGCTCGGTGTAGGTCAGGGTTTGAAACTAGCAGAGTGCTACTAGGGAGTGGCAACCCACTGAGAGGTCCAAGCAGATCAAGACAATGGTTTGTGTATAGACTAATGCAC
**CoV-bait-2541**	GGAATTCATCATAAAAAGTAGGGTTTTCGATGGGATCGTACCATCCATCTACAAACCACTCTTCTGATGTACCACACATTGTTGTTAGGATCTCTTGTATTATCTCTTGAGAGGTAGAAA
**CoV-bait-2542**	GAAATTAGTAAAACCATCAAAAGCATCAAGAAACTGCGCCAATGATGTTGCAATTGGGACATTAAATATACCAACACTAAGAAGTGGTGTCACAACAAGCCCATGACCCTGTTTTCTAAC
**CoV-bait-2543**	ATAAGCTCCTATATCATCACCATTGTCAAAATAAGTAGGATCATCTTCTTCATACTCTACTTCAGAACCACTACTGTAGTCTTCAGAATCAGAGCAATTTTCATCCTCTAGAGGTAATAT
**CoV-bait-2544**	AGACTGAACCATCCAATTTGCATGGACAGAAAGTGGTGGGAGCTTTAAAACAATGTTGTGCATAGACAACATCTTTAGCAGTGAAAACACCTGCAGGCTGAGGCTTAAAAGCGAAATTCT
**CoV-bait-2545**	CTGTGGTCTCCTTAACAAACTTCTTGCCAGCTGCTACCGCTATAGCCTTTGCAACACCACCACCATGAGCCATATGACCATTAGCAGGGTTAACAACGACCTCAGCATTCACAAGCCTAG
**CoV-bait-2546**	AATTGTCAGCGGCGGTGTCAAACCTGTGGCACGTTTAGCATAAAGCTCAAATGCAACATTAGTGGGTAACGAAGTTTTGTTAACAAAAACAACATTATCCGATAGGTCCTCACGAACAAG
**CoV-bait-2547**	GCCTTTGGCGAAGAATGGTGGAGATACGCGTTGGTCAAGGCCAGTTACATCCAGATGAGTTGTGGCCTGGTGTGTATGGCTCCGCACACATTTAAAGATGTCCAAGCGACTCGCACTGTA
**CoV-bait-2548**	TTGCGACAGATGATCATGTACGACGATATGACCTTTCGAACCTAAGCTAAGATTAGGGAATTTTCGCACTGGCTACTACATAAGCTATATGAGCAAGGCTAAAATAACAGCAACTAGGAT
**CoV-bait-2549**	AAAAGCGGTCCAGGCAGCATCTGCTAGAACACCAAGTTTCTTCAAGAACCATGGTTTTCCAAGCACATCGTCAGTAACGTCACAAATGAAATTACCAACGCTGAAAACACCGCTAGCAAT
**CoV-bait-2550**	ACATTAATAGCATGCTGTGTATCAGATGTCTGTGTGTAAATGACATAATCATACTCACTACCTTGCGCAGAGTCTACGGTTTGCGTTTGCAGACCTAGAAGACGTCGTGCTACATAGTTC
**CoV-bait-2551**	AAGAGGTGTCCGCTAAACTGTAGTGGCAACCCGGATCTCGCTCGCTACGCAGTGAAATAGCGCGCAGTCATCTGTACTCTTGTAAGTCCACACTCGATACATTTAAAGCCAAAGAAGCAT
**CoV-bait-2552**	GTGCATACAATGGTATTGCATCAGTGGGAACAAACCAACCAATGAACACACCATTTCCTAGGCAAACGCCATCAGGTGTGATGTTAAACAACTGGTATGCAACATGGTAATTCGTCAGAC
**CoV-bait-2553**	AACTGCATTCCATCAAGAACCTTACGCAAATTCTCAATCTGTGATTCAAGATGTTTAATATCCGCCACATCTTCTGCCTGCCACATTAGTGTAGGCACTTCTCCCAATTCACTTTCTAAG
**CoV-bait-2554**	GCAAGTCTGTAATCTTTGGCATCAGACCATTCAGACGTAGCAAGAGTATTAAAAACTTCCACATCAGGTGAACCAGTGGCAGAGTTCTTGTAAAGACCAAAAACATTGCCATTTGAACAC
**CoV-bait-2555**	TTCTCTCATTTGTTGTGAGAGCATTAAAAGCCTTCCACGGTGTAACAGTAGTTCCTATTACTGGAAACAAATCTGTGAATAATGCAGTAGTGTTTGACCTAAATATCCTGTCAGGATAGT
**CoV-bait-2556**	TGTAGTACACTTTAACTCTGCAAGTGGATCTTGAGAACAATCCACAGCATCTACAATAGTTCCATTCTGATTAAAACTGAGCATAAAGGTGGACTGTTTAAGATTACCCACATAATAAGC
**CoV-bait-2557**	ATAATCTGCACCACTCTCAATAGCCAATAATTTTTCTTTACGAACTATATTTTTAGGAGTTTCTTCAACCTCACTAAAAACACGAGCCGTGTTTGGTTCTTTAACTAGATTCTTAGGCAC
**CoV-bait-2558**	TAACCTTATTTACAGAAATCAACCATGCTAACACTTTTACAACTCTACACTGATCAGGCTTATTGCCAATCCATTTAACACTCTTAAATTTAGTTAAAATGTAGTCACAACAAACTTTTT
**CoV-bait-2559**	GTCAGTACCTAACACAGTTTCCATTACTAAATTTGCAGTACGTTTACGACGCACTGCACCAATTAACAAATGTAAACCGCCTATGGTACCATCAAATTGACCATATGCAATATGCTCAAG

**CoV-bait-2560**	CAATGCAATTAGCTGTACAGGGTTGATAAGAAATACCATCCTCACCAGCACTAGTAACATTAAGCATGTAATAGGTGGGTTCGTAAACATATTGCATAGCACAACCTCCACTGTTGTAAC
**CoV-bait-2561**	ATAATTAGTACACATGCTGACTTCATCCACAACGAGTACATCAGTTGAGGTTTCTGGTAAGGCATTGATGGTGCTAAACACATATTGTGCTGTACCATCATTGACTTTGAATTTATCAAA
**CoV-bait-2562**	CCACTTTATTAAAAAGCAGGTCTTCAATAAAAGACCTTTTTTGTACCACCCTGCCACTTGCAGGATCATACACAGAAACACCCAGCACATTAGTAAAATTATATCCATCACCATTAAACG
**CoV-bait-2563**	CTATGGAGCTGAGTCGCGAGTTGGGGCCTCTCATCGTACAGCTGAGGAACTCGGCGGTAGACTAGGCTTTTAAAACATGAGGTTATTGTCTAATGGGCGTGATCTGACTTAAAAGAATAA
**CoV-bait-2564**	GAGCTCTGAGAATCAGCTTGAAAAGGTTCTCTGATCGAGACCAGTCTTATGTAAGTACACTTCGCCTCATATCATAAGGAATAAATGAAGTGCATGACTGAGGGGGAGTGGCTTGGTCGT
**CoV-bait-2565**	GGCCACGAGGCCAGGGTCGTGCCAATCATGCTCGTACTTCTATATACGTTTGGCCATGTCCAGAGCGCGCATCCCACTAGATTGAAAGGTTATAGCGCGGGAAATACGCTGATAGTCGAG
**CoV-bait-2566**	ACTGGCTGCTTAGCATTACCATCACGAATGATGGCTCCATTGAAGTAAGCATAATCAGGACCAACCATACATGGGTAGTTCTTGATTTTTCTATCAGATATTAATATAGCATTAGGTGTG
**CoV-bait-2567**	CATTGTTGTCACCAAAGGTCACAGCACGGGGCGCTGCAGGGGTGGCCATAACAATGAAAATTAAGATTCGTTAATGATTTTAAATCATACTCTTACTAATGAGCTTACGCTCGCAGCAGT
**CoV-bait-2568**	GAGTTCGTTAAACCCACTCGTCAGGTGGTAGAGGGGGTTTACTATCCTGGAATTTTACATAGACTGAACGTCCAGTATTATACAAGTATAATGCGGGCTGAACTAACAGGGTATTGAAGC
**CoV-bait-2569**	AACAGACAAAACCATCTTATGTGGAGTTGCTATAACATGATCATAGCAGCATTTACAGCAGAGAAATGGTCTACGGATGCATGTCCCACAGCGTAAGGAAGTCTGTGAATGGCATACAAC
**CoV-bait-2570**	ATGTGGCGTATGTAACATAATCACCATTATTGGGGCACTTAGTACCGTTGCGCGGCTCAAGCACACAATAGAAGACCCATAGGTTGGTACCACAACCATCGGGCAAAATCACTAAAGTAT
**CoV-bait-2571**	ACGCATCCAAGCTGTGATCTATAAGTGTTCTTATTATTTAGAACGACTGTTCTAAAATTTACACCATCGACAGTCACTAAGACTTCGATTGTTAACTGCTGTGTCGTGCGTGAATCCAAA
**CoV-bait-2572**	GTATTAGTCTTGTAGTATTTCCCTATACCAGCAACTTTAGCCTTGTAGTTGCAGATGTCAAATGCCCTAAAAACGACATCAGTGGAAAGTCCACTAGCACAGGGTTCTATTCGGGCATTT
**CoV-bait-2573**	GTCTGGGCGAAAATCACATAATCATACTCTGAACCTTGTGATGAGTCCACAGTTTGGGTCTGTAAACCCAGAATCTTACTAGCCACTGCATTTTGGCTATTATATGGTGAAATAAACACA
**CoV-bait-2574**	GTAACTTGTCAACATCTAAATGCTTAACAAGATTACGGAGCTTGTCCACATTAGTGGTGGTGCCAGAGAGAATAGCAAGAACGACATCGTTAAGCACAAACTCTCCAACAAAATTCAAGG
**CoV-bait-2575**	TCCAGCAATAAAACCAAGCCAAATGTACCACGGCCATTTGTTGTAATAAGTATAGTTGCCAAGCTCTTTAAGGTCTATGTAAGACTCATTAAGAGCTTTAACAACCTCCTGAAGAGCTAA
**CoV-bait-2576**	TTTGATAAGAGCGAGTAGGTAGGCATGTGTTAAATCGTCTACTTCAATTTCTTTAACTTCTGATTGCTTGGTTGTAGTTGTGCAGTCAATAAGTGTGGCAACTTCTAGATCAGTTGTTTC
**CoV-bait-2577**	TTTTGCTGCACTTGTACATAAGAGGTATTATACCAAAACAACCACAGCAACATCCGCAACAACCAGTCATAAAGAAAATCCAGCATAGGATTAACACAAAAATAAGGCATGCAAAACCTA
**CoV-bait-2578**	CAACGGTACATAGCCAAAAAGAGAACAAACAAAACAGCAATGCTAAACTCAGGAATAGCACGATAATGTTTGCTATTAACAACAAAGTGTTGCTCACTAGCAACAACAGATTTAGAACTG
**CoV-bait-2579**	TTTTCTTACGTAGAACCACACCTATCAAGAGGTGTAGTCCTCCAATAACACTTTCAAACTGTCCATAGGCTATATGCTCAAGGCCATAACCCTCTAAGTCATACTTCACAAGGAAGTCCT
**CoV-bait-2580**	ATACTTAAAAAATGAATCATCATCTGCGAAGAACATCGTTAAGAGATACTGGTTGTGATCCATTTATTGATCGCAAAAGCTTGGCGTAATCATGGTCATAGCTGTTTCCTGTGTGAAATT

**CoV-bait-2581**	ACAAGGACTGATTTTGGAGCTACCCTTTCGGTGATCATAGAAGTATGTGCCTACACAGTCCACTGGAAGATAATTTAACGTCGTATGAAAATTTAGGAGGCAGCGTAAAGCCTTGATTTC
**CoV-bait-2582**	TTGATGCCATTAAAAGACTGTACGCAAAGGAGGTCGCGAACTTCTTGACCACCAGTGCAATTGTTATAAGCCTCGACAAAGCCAACGTCAGATAGTTTGACCTTGTCAAATAATAAATCC
**CoV-bait-2583**	ACACTGTAAAATCTGTGAAATTACAGTGTGCTGTGCAAAATTGGCTAGGAGACCATGACATACCACTTGGTGGAGGCGTCATGCCACAGAAACAAGCACTAAAATTCTTACTCCCAAGAA
**CoV-bait-2584**	CTTGAATTTGTGTTCTGGTGTGTTAGGGTTTACCTGGTTAACTTTAAGTACTAGGTTCACACCCTTATATTTGGCAGACACAACACCCAAAAACACATTATTCTTAGAGACTGAGAAATT
**CoV-bait-2585**	GTAGTTTGAATTAGAGAACAGCAACCAACCCCTAAGATTTGAAGGTTGGTCACCGAATTGGCCTATACGGGCGACTAGTTGATGCACCCCTGTAATAACTAACACGTAGTCATCTTCAAG
**CoV-bait-2586**	TTTTAGAACGCTGTTGTTTTGGCGTAGATGGTGCAGATTTTCTATCTCTAGATGCTGAGCGTAGTTCATTCTTTGGACGTCTCCCCACACCATCTATACAATCATCACATATCTTACTAT
**CoV-bait-2587**	AAATAATTCTTATCAAAACCAAGCGCACCAGCGTCCGATTGCACATTAGATTTAGGTTGTCTAAGTGAGTCGCATTGACACCCATAGCCAACCCAACATTGACAAACAGTGCACACCTTA
**CoV-bait-2588**	GCATGGTCCATTATCTAATCTACGTTGATGTAACTAACGGCGGTACTGACTAGTTTGAAAGTGTCCATAAGGCGGGTTCTATGTTCCTTATGTCGACCCCGCCGCGCGGATAAGGCATCA
**CoV-bait-2589**	CAGCAAAGATAGCTTGTTTGACTTCATCAGAAAACTTGCCACTAAGGATGTCAAACTTGAGCTTAGTAGCAATGCTCGAGTCATTCAAAAAGTAGCAAGGATCTGTGAAACCTTCCTCAT
**CoV-bait-2590**	GCGTGTGACGCCCATACCAATGATGCGTGCCCTGGACTGCGAACGATATCACCGGCCATTAGGAAGCGATTTAATCAGTAAAGGCGGACAACCGGGTGGATTTGTGCGGAGCGTTACTGT
**CoV-bait-2591**	CGAAGCACACTGCGGCGTCATCCTTCGGGCTTAAAAGGACTGCGGGTGGATTCTACACCCTGAGCCCCATGCACCTTCAACCCTTCGAAGGGTGATATCAGGGTAGGATGCCCTGAGAGG
**CoV-bait-2592**	GTGAAAACAAATGTGTACCATTACCACAGAATCCGAATCTCTGAGACTGAGACCTAACACATTCATTAACCTTGTCTTTGGCAAGTTGTCTACTAGCCCTAACCTCTGCTTGTCTGGTTA
**CoV-bait-2593**	CCAATTCCGAGTTAACCAGAGACCTAAAGAATATTGGCACAGGCACTCTCTCTATGATCGTGACTAGCGCGCATTATTTGCTACCCTGATGAATTCCGAATATTTCGCCTCAATTTTTCC
**CoV-bait-2594**	GAACAGACTTTAAATTGCCGTCACCACCGCCCCATGATGCCATATCTAGACACTGGGGTATGATGCGACCGCCAGGTGATATTATCTGTTCATCTCCATTAATGCCACACCAGGCAACAT
**CoV-bait-2595**	ATTACATACACCTCTCTCAGGAGCTTTGCTCGGGGTCGGTACTCATTTATATCCATTCTGGGTCCTCTATTAGACGTCAATTAAATACCTCCGCACAAATACGTTTCTCGCTGCAGACCT
**CoV-bait-2596**	ATTGAAGTGCACGCCTACAAGCTCCTGAAATGACAACCAATGGCAATTCTGTGGTCCTCGGGTCAAACCGAGGATAACGTCAAGTGGTTCCTTAACGCTTAAAGAACTCTTATTAGATTA
**CoV-bait-2597**	TTCCTGTGTCGAAGATCTCGTTGATAATTTCAACGGCCGTGTCACCACCATCAACAGCTGTGTCCCATTCCAAATTGGCATCAGATGGCACACCCACATCATCGTAGATGGCCTCTTCAT
**CoV-bait-2598**	TTCATAGTGACAAGGGTGACACGACCTATGTCACTTGGACATGCTTTCCTGAACGCATCCAGAGAGTGGACTGGGTTGCATCCAAAGATACCAGCACCTAGTATAGGTATAACATAGTGT
**CoV-bait-2599**	CGACCACTGCTTCTCAAAGTACCCGGCGAGCCAATAGGTTTACCCCGACTTCGTGTTCGTTCTGTTGACCGCTCCAACGAACCACACATTGAAGGGAGAATACATGCCTTGTGGTCTGGC
**CoV-bait-2600**	ATGTTGTGAGCAAAATTCATGAGGGCCAACATTCATGTCAGTTTCTGTCCAACACTTTGAGTCTGCCATGTAAACATTGTTTTGGTAAAACAAAATGTCTCGGAAACCATCAAGATCAGC
**CoV-bait-2601**	GTTATGGCCATGGGTAAGTTTGCTTTAAGTATGATGTTAACATTAGGTATACATTTTGTCAATACCTTTCTGATTTCTGATTTGAAGAAATGGTACTCAGAATAGAAGCCAGGATCTAGA
**CoV-bait-2602**	CAAGCCTGAAGTAAGGCTTAGTGACACAATAGGATGTGTTACCATTGATGGTAACGCTGTCAAGTCTGGCTTCGCCAAAATCCATGTGTGCAGAAATATTTAACTGCACTACTTCAAGCT
**CoV-bait-2603**	ACCGGCTCTGGTTTAGGATAGACGTCGACGCTAAGTACTTCGTCGCTGCTGGTCTTCCGTTCCTGGATCTTGAGTAAAAATTTATCAACATCAGGAAGTTCCCACTGTTGGGGAATTACT

**CoV-bait-2604**	AGTTCTGTAGTCTGTAACTATTATTTGGTTAGCATCTGAAGTGAACTCTCTTATATCAAGGTCAATAAGAACAGTTCCTTCAGGAAACATTTGTTTTATGACTGAAGAACCTGGTGCTGT
**CoV-bait-2605**	TATTGGGAAGATTAGCAGCGGTGGTGTTGTAAAAAGTAACGCTGCAATCAGTTAATTGTATGAAATCAGCTTGCCGGGGCAGTCTGGGTTCATACATATTTCTAGGTGTAACTCTCCAGG
**CoV-bait-2606**	ACTTCAAACTTGATAAACATGCTAAAATATCATTTGGCAGTGATATATTTTCCAACTGTCCCCTCACCAATAACTGTAAGAGTTTATCACTTGCTTCCACCAAATCTTGAAGACAAACTT
**CoV-bait-2607**	CAATTATTTGAATATTCCAAACCGAACTCTACAGTAGCCACCTCAGCCTTAGTGATGCGGTCATTGCGCACCCCCGATCTCTATTATCTGTACCTTATGGTTACCTACTGTTTCAAACTT
**CoV-bait-2608**	TAACACAAGTCAGTATTACCAAAGACGGCGTTTATGGCAAAAATCAGAGAGCGTCCAACAAGTGTTACATAAGCCGGCACATCTGGTACAGGTCTCATATTGTCTACAAGGTCAAACACA
**CoV-bait-2609**	TTGTTACAATCTTTTACTACAACAGTCACAGGTGTTTTGAAATAATCCAAAACCTTACGTTCTTCAGCTTCTGTGTACACAAAAACACAAATAGGTTTTTGTTTAAACGCAGCAAGAAGT
**CoV-bait-2610**	AGTAAGAAAAAATAACAGCAAAGTAAGTATTTAAACACCGCTAAAATTGTCTTAGCCACAGTGTCACCAAACTTAAAAAACTTATTTGCATACTTGTCCAATTTTTGTATAGCATCAAGA
**CoV-bait-2611**	AACCTCCATAACAAACACGTACAACACACTTTTCTACAGCACCAGATGGTTGGGCCATCTTTCTAAGACCTGATTGAAGTGTTGAGTTGTAAGAAATAGTTGGTGGTGTGTACAACTGGT
**CoV-bait-2612**	CGTGCTAAATGAGCATAACATGCCATGCGATAATCAGACTCACCCATACTGCCAGTATAATATTTGTACTTATTAAAAGTTGCTGCATAACCTTTCAAGCGCTCCATAGGTATAGAATTA
**CoV-bait-2613**	AGCCAGACATACCATGGCCATTTAAGGTAAGTTTCAACACGATTTAGCCATTCTAGGTCTACAAGTGTGTTGTTTATATTTTTAATATACTCCTCTAGCTGATAAGCTATCATAGAGAGG
**CoV-bait-2614**	TCCTCAACAACAAGGATTTTAAAGTGCTTGGATCCCATAATGAGGGGAGTAAGGTTTCACCTAGACGGAACTAGGATCCACGCAACAGCTAGACGGAACTAGCTGAAGCACCTACTAAGC
**CoV-bait-2615**	TAATATTGGTTGTCTACAGCATAAAGGCGCAGCAGGTTGCTAACGGATGTGGTAATATTAGAGTGAAACCTAATATATTCCGTAGAATTACCTGCTGCATTATGCTGCGCACCGCCAGGT
**CoV-bait-2616**	CGCAATTCCTCCACCATTGGTAAGGCGCTCGTTTGCTGCATTAACAATGATTGCATTGCGTAAGCCTTTCAGCGATCTAATATCTCCAATGTGAAATTCTACCTGTGTTGGCTGTTCATC
**CoV-bait-2617**	CTGCATCACAATGCTTACTTTAATATTAAGAAGTTGTTCTGTCACGCTAGCCACAAGTGCAGAGAGTGCACCTCCACAGAGGTCTGAGACCTTGTTAAGCCATGACAGAAGTTTGTGAAA
**CoV-bait-2618**	CCTGACTGCTCGCGTATATCAAGAGAGAAAGCTCTAGAAACATACTCAAAGGTACAATTAAAACTATCACTAAACAAGTGTGATAACTGGGAATTATTAGATTTAAAAACCACAAATAGT
**CoV-bait-2619**	ATAAATAGTCTTTATATTGGCTACAAAATAGAGGTATGTGTTTAAGTGTTTACCATTAACCATGTGAGCAAAGCGCATAGGTTGTTCTAAATTTAAGACTACTTTAGAACCATCATCTTT
**CoV-bait-2620**	TGCTCCTCTACGACTTCTGACACAGTGTCATTAGTAGATGGGTCATCGACAACTTCAACATCACTAACTTCTTCAACATTTTCTAGGGGTGCATCAACACAAATATCATACTGTGAGATC
**CoV-bait-2621**	AGTTGACCAGAAGCTGAAGTATAGGTTCTGCCAGCTAGCAAAGTGACATTTGTGGACTGGATAATTCCTGTGCCAGTTTTTCCATAGATGTTGTAATCAACACACTCATTCTCGTAGATA
**CoV-bait-2622**	ACTGGGCCCTGCTAATTCAAACAGGTTGGTATATGTCCTGGCTGAACGGGTAGGCTCGGACCCGGGATTACTAGGCCCCGCAGTGGCGTATTCTGAACACGCGTAGTATGAACAACCTAA
**CoV-bait-2623**	CAACAAGTAGCTCTGCCACTAAAAACAGACTGCTTACACCCACCTTGAAGGGGACCATAAGCTAATGAGACTGACAGTGAGTTAAACCACAAGCCATTATTAATAGTTTCTGGTCTAAAA
**CoV-bait-2624**	GGCTGGTAGAAACACAAAATTTGCTAAAAGACAGGTAATCATTAACAGATTGCAAGGTAAAAGGGCAATTACTGTCCTGTGATTTAGACACATAACCATAACTGTTTGTAACGTTATAAA
**CoV-bait-2625**	CTATGGTACAAGCACCAGCCAAAAGGCTGGTTGAAACACAAAATTTGCTAAAAGACAGGTAATCATTAACAGATTGCAAGGTGAAAGGGCAATTACTATCCTGTGACTTAGACACATAAC

**CoV-bait-2626**	TCCGGCGGGTCATGCCTCAGTACGGCTTATGGAAAACCTAATAGTACTGGGGACGCGGCTGGGAACAAGGTGCCCAAGCCTGGTTTGGTCTCACACCTGGGCGGCCCCCTGATGATCTGA
**CoV-bait-2627**	CAAATTAACTCACAAACATCAAAGCTAAAATCAAACCTAGGAAATGGATCATCCTCAATATCATCAGGATGGTAGTTTACATACTCACCATCATCTTCTTGGTGTTGCTGGTCATGAGCA
**CoV-bait-2628**	TACCTGTAATAAGTGTTGGGCTGTAAGTCACTGTACAGTTTGGCACCATCGACCAAATTGGCTTCATGACAATAAACACCAACACCACCTAAACCTTCCAGTTTAGTACAAGCAGAGTTA
**CoV-bait-2629**	GTAACATGTAAAAACTGAATTCCTATTAAGTGGTGGCTTTGTCTTAGAAGCTTCCCATTGTAAAACAACTTCCTTAGGACCTACAACTTCTTTAAGCACAGCACAAGCATACTGGGACTT
**CoV-bait-2630**	ACCATCAAACACACTGCGACTATAAAAATAATCTGCCCCACATACCATACTGTGTCTGTAAGGAATAAATTAAACATTTCTTTCGAGCAACAAGGCCCTAAAAAGAATAAAATCGACAGC
**CoV-bait-2631**	CACTGCTCCCATTGGCAACGAGGTCAGTGTCACCAAAATTGGCTGAACTGCTTCTAGCTCCATAAAATCTTGTCACATCACCTTTACCTGCAGTTCTCTTCCAGGTGTGTTTGTTTTCAT
**CoV-bait-2632**	TAAACCGCAAAGTTGAATACAAAGTTTAATAGACGCAAGGAAGGCAACCACTACAATAGTGACCATCAAACACACTGCGACTATAAAAATAATCTGCCCCACATACCATACTGTGTCTGT
**CoV-bait-2633**	CCAACTCAAGGTGGTGCATGTAACAAAACTCTACAACACCATTATTGATATTGTAGCCTGGTGAACCACAAGCTCCACTAATAAAAGAACCTCTGATAGTGTAGTTTGTACGCATATTAA
**CoV-bait-2634**	TTTTAATTTTTGTAAGAATAGTGTCTAAATTGGGAACATCCCAACACTGTGGTATGACTCCTTCATCGGCACCATCATCCTCACAAGAATATTCATCCTCTTCTATAATGATGTCATCAC
**CoV-bait-2635**	ACATGATTATCATCAAATGTATACCACTTATCCACAATCTTCCTAGCAGCTGTCCAATGTCCACATTGATCTTTCCCAGAAAACATTAACACTGCTTGTGCTGGCACTTCAACACAGAAT
**CoV-bait-2636**	GACCCCTCTTCATGCGCCAGCGGACCTGCTCATTCCAGTATCCGACTTGTTGGTCTTTGTTACCAAAACCTTTGGGCACACCATTCTGAGGCATAGCGCGGAAGAATAGTGATCCATCGG
**CoV-bait-2637**	CATTGGCACAGGCTGTGAAAGTATGCGCTATGCAGCCTGGACCACAAGAGTTGGTGTTTGTTAGACACTTGTAATTATACGGTTCGTGCTTCCCTTCACAAATGCACATAACATTATGAT
**CoV-bait-2638**	CGCAGTACTTAGGCTGCTATACCCTCGCGATGCCAGACTCATTTCACTCATAAGTACCACCGTAAGCAGATCCCTGCTTGTTCTATGGTCAGTGTACGTTCTTGTACGAGTCCGATCGGG
**CoV-bait-2639**	TTTTAGCACCATTCTTACCAACCCAGAAAACTCCATCAATGCGGTCCTTAAATTTAGCATTGCTGTGTGGTCCAGTTCCTAAGTAGTAGAAAAACCACCTGTCTGGTAATTCCACTTTTT
**CoV-bait-2640**	GGCGCGGCCCCGCCACCGTCCGTGCCTTCTCCCAGCACTGCCCCTACGCCCTGCAGAGCTGTCCGTGTCCTTGGAACCCTCTCCAGGGTCTAGCGGCCGTGGCTTTCTCTGCCTTCCTTT
**CoV-bait-2641**	GTGGTGTTCATGAAATAAATTAGTCCTTTTTGAGCACTTGGGAGAGGTCGTTCCATTATGTAAAATCCTGGTTGGTCAGGATCTCTTCTGATGGTATCTGTTACTGGGTTATACGTTGCT
**CoV-bait-2642**	AATCTTGAGATCTGATGAAGTCAACTTCTGTTGGTGAGAGTTTAGAGCGGATCAAATCATTGTTGACTTCATGGTAGCCAAAGAGTGGTGAGTGTAGGCCAACCATGTCAGTTGTGGCTG
**CoV-bait-2643**	TATTACGGCTACTACTGCGTGAATTACGACCTTCTGCATAGAAGCCCTTGGGTATGCCTTCAGCCAACTGAACAGAGATGGCACCATCATTATTAGGGTTACGTGTGCCCATGTCACCAA
**CoV-bait-2644**	GACTTACCACTGCCTGGAGGACCCTGTATGGTTGTAATGCGTTGCCTGCCAATCATTTGATAATAGGGCACAAGGTTGGAATAAGCTTCATTAATATTAAATGAAGGGTGTAACTTGTAA
**CoV-bait-2645**	GTTACTGTGGCATTGTTGTCGAGCCAGTCGTTGATAATTTCAACTTTGGGTGGTAGAGGTGTCAGGTTGAATGTGATGTAGCCTGAGAGAATACGTTGCATTTCTTGGTCGTTGTTGACA
**CoV-bait-2646**	CATGGCTACAGCAGAAGCAGAGAAATTTTTACTCCAAAAAATAGCCCCTGCAATGCATTGTGAAGCTGTGCCTGCATTATTAGTTTCTAAAGAAACGTTTACTACTGCATAAGCACCACC
**CoV-bait-2647**	GTACAGACTCAGTAAAATCAAAAAGACCAACACTCATATAAAGTGCAAAAATGAATGCAAAGACTATACACAACCAACCAAAAGGTTTAATAGCACCGCCCTTCTTACAAACGAAGTTAA
**CoV-bait-2648**	GATCCGGCAAACAAACCACCGCTGGTAGCGGTGGTTTTTTTGTTTGCAAGCAGCAGATTACGCGCAGAAAAAAAGGATCTCAAGAAGATCCTTTGATCTTTTCTACGGGGTCTGACGCTC
**CoV-bait-2649**	TTATAATGTTTTAAGTAATTAGTAGATTCCAAACTGAGCGCACCAGCCGTAAAGTCGTCAATAGCTTTTGCTACACCACCCTTATGTTGCAGATGTGGGTTAGCAGCATTTACTAAGACT

**CoV-bait-2650**	AAATCAAGTCCATGCGTAACGTAGCCAAAAGGCATGGCACAAATACCTGAGCAGCCATTAGCAGCTGCAATAACATCAGTCAAGGCATCTCTAGCACTATATAAATAATAGCTAACACCA
**CoV-bait-2651**	TGCGAAGAAGGAACCAGGTTGAGCATGGCAGTAGTACGCCCATCCCTAATGCCTTCCTCTACCATTTTATCATCACCGAAGTTGCGCTCACGGCCCTTCTGACGTGAACCAAAAACCTGG
**CoV-bait-2652**	TCGTAGACCCAGGCTGGTTGTTGAAAAACGTCACCGTAGCCAAGGTTGACAGTAGATAGGAGTGATCGTCTAGTACGTGTTCTTGTGTCGTAGAGGTAGTTTGCAGCGGCTTGAATTGTG
**CoV-bait-2653**	GAGCTTCGAAGATGGCTTGATCCATAGCTTGTACGAGTGCGTGTGTTGGGGCAATGTAGGCTATTTTGTTAGTTGGTTCTGCTCTGTCAATGTAGTTCTTGATGACGAAAGTAGTTTTTC
**CoV-bait-2654**	TGTGAAGAGATAGACGATGTTGAAGTATTTGCAGAGTTGGAAGCATGTGGTTACTGTGTAAAGGACATGGGTGTTGTAGAAGCATGTTTTATTAAGGAACCAGGCAGCTAGGTAGGCAGT
**CoV-bait-2655**	TGAGTGCACCTCCATTGAGCTCACGAGTGAGTTCACGGAGTACACCACTGCCATGCTTAGTGTTCCAGTTTTGTTCATAATCTTCAATGGGATCAGTGCCAAGCTCGTCACCTAAGTCAT
**CoV-bait-2656**	GGGTAGTCGTCGTAGCCTGCTGTCGCGATCCCATGTCGTCGTACCCACGCCGTAACCATTCCCATGAGAAAATCTGCTCTATGCCCTCATGTCACGTCGTCGAGGTGCGTTTCTACAGTG
**CoV-bait-2657**	CGCACTCTTCAAGGGTTTCCCAGTCAAGTGGTGTTGTTGATGGTGATGGTGGTGGTGTTATATGGTCAGATTGTTGTTCTGTAGGTGTGTCTTGTGAATACAAATTTAATGTGGGTGAAT
**CoV-bait-2658**	ATGACATTAGAATCTGGAAGACAAAGTGTGTGGCTAAACCATAGAGTTGCAATTCGACATTATAAGATGTAATAAATCAGATTAGCAATCGTAATGGCACTCGACTCGTCCCATACACAC
**CoV-bait-2659**	ACTTATGATCAACATGAACACTACGATACACATTCTCATACAATTGACGCTGTAAGTTGCGCACGTAGTTGTCCACAATCTTGTTTCCATTAGCTGACAGTAGAGCGCTAACATTAGCGG
**CoV-bait-2660**	ACCGAAGCGCCCCTAGTATTAACTATGGACTTAAGGTGTTTTTGATGATACTGCCGAGTAGTCATGGTTGACAAAAGCGAAACACCACCCACTGTGCGTGCACGTTCTTTGCCACTTATA
**CoV-bait-2661**	AAAACAACGTTCGTACATTTCATCTCTGTGAGTTTAGACTGCACAGTGGAGATTTTTATGTTTCGCTCTCCACCAATGCCCATCAGCTTAACACTAAGAATCATAGCATCATATGCTGTC
**CoV-bait-2662**	CCTTTGTACGAATAGTACGGATGCCAAAGCCACGTGAAATAATTTCAGGTAAAGACACAGCATTACCATCTACCATTTTATAGTAGCTATGAGGTGCCAACTCACTATAAAGTTTGGCAC
**CoV-bait-2663**	CTTCCCGAATTTGCTGAAAGCATACTGTAATGAAAGATGGTTGAGCTTAATAGTGGCTAAGCTCTCCCAACCTGGATGCGGCCTTATAAAGCGGCACGCTCAATGCCTACGTTGCAACGA
**CoV-bait-2664**	GACAAATTTTTGCTGGCGTATTCCAGTTGCATTGCATAGCTGAAAAACACCCGTGCCTGTTATGCCATAAATGTCATAGCTAATACATGAACCTAACAAATGAGTAATGTTGGTACTATA
**CoV-bait-2665**	AGTCGAGCTGCACTAGAACCCCGCACTCGTTTAAATAACTTTGATCAACAGTAAAACTCTGCATAGAAGTACGATCGCACATGCAACCATTGTTAAGCCAACAACCACAGACAACACAAA
**CoV-bait-2666**	GCGGCAGAAACACGACCAGAATAACAAGGACGTATAGTATACGTTTTGTTAGTTTGATAGTCTTTAAAACCATAAAGATTACCATTAACATCATATAAAAGGTTCTGCCAACTATTATAA
**CoV-bait-2667**	AGTGCTATAGGGACCAAACCCTTAATAGCACTGTTAGAAATCAGAACACCATCTTTACTGGTTGTAAAACGTTCATAAGAACCAACTATGCTATTATCATAGCATACAACAACATCATCT
**CoV-bait-2668**	TGTCCAGGTTAAGCACCATTTTATCGCCCTTATACAATACTGTCGCTCCAGGAGCAAACTGATGTCGTGAGCTTAAACTAGTTCTTGATGCAGATGACGTTTTAAGCACAGAAGTTAAAA
**CoV-bait-2669**	CTGCCTGTGAACTTCCATTCTCCATCATCTTGAACAAAATATCCAGCTTTAGGTGCTAATCCTCTATCACCAGAAATGCAAAGTCCAGGACTCACATTTGCGGTTGTAAAGGATATTGGC
**CoV-bait-2670**	AAGGATACGTACCATTGCTGCGCTGCTCTCGAAAACATCGTGCTTATGTAGCTTCCGGTCAACCTCGCCACGTAGCGTTTTCCGTCGCTCGCGGTTACAATAGTACGTCTCACCATTCGC
**CoV-bait-2671**	AACCTAAAATTTACATCCCCACGTCTCACACACTTAATACCTTTAGGGTTGCCTATGTCATAGACAACATTAACTTTGAGAGCATCAACACATGCATTAAGATACATGCGTTGTAAATAT

**CoV-bait-2672**	CCAGCGGCACCGCGCCTTTCGGCGGTGAAATTATCGATGAGCGTGGTGGTTATGCCGATCGCGTCACACTACGTCTCAAGGTCGAAAACCCGAAACTGTGGAGCGCCGAAATCCCGAATC
**CoV-bait-2673**	GCCAAAAGATGTAGTTTGCATGCATAGTATTGCCATCAATGACAGGTGTTGTTGCGTAATCACCAAGGTAATGTACGCCTATAAGAAATGACTCTGACGATGATGTATTAACGCTGGTAC
**CoV-bait-2674**	TCAGCATATGTTATACAACAACTTTTCAGTGTACTGTCAGAATTATTCATAAATTCTTGAACAGAAAACGTAACAATAAAGACGAGTTTGCTTAATGGGCGATGGGCACGCTCACGTGAC
**CoV-bait-2675**	CACTCATATTATTGAAAGGCGGGAGTGACGGTCTTAAAAAAGGCTTGACACAAGGGAGTTTGGAGGCAACGGGCGCATAGCGCAGTAATTGCAACTTAATTTTGCTGTTGAAGACGCTAA
**CoV-bait-2676**	ACTCGTTCACCTTGTCCTTGGCCAGCTGGGCGCTCAGGGCGGCGCTCTCGCTCCGCACCAGCTGCTGGGCCACGAAGGCGTTCAGGGTGGTCAGCCGGCCGTTGATCAGCCGGTCGATCT
**CoV-bait-2677**	ATCGAAGGCGTCCATGACACTGAGCACTTCGACTAGTTACAGGCGGAAACCATATCTAGAAATGGAGATCATGGTCGTTCAATTTGTGAGTGGAGGCAATGAATCGATCCACGTCCTTCC
**CoV-bait-2678**	GCCATGTTTACGAGCTAATATGAGTGAAGCGAAGATTCTACACATATTAGGCATAGCTCTATCACACTTAGGGTAATCCCAACCCATAAGATGCGGATTATCAACATCTTTGTACAATGT
**CoV-bait-2679**	AAAAATGGTGTTGTCGTTAAATTTGCAGGATCTTGTAAAAATGGTCTAACATTATTAAGACACATAGCCTTGTCAAAATACTTACCCGAGAGTAATGTATTCTGAAAATGAAGTTGAAAA
**CoV-bait-2680**	CGTTCTCTCAACACTCGACAGACGGGTGATCGCGACTTTATCTATGATGTCCACGCGACCGGGAGGCCGTTACTGCTCATAAAGAAATGCAGCTTCCATAGAAGAGCTCTCTATGCCATC
**CoV-bait-2681**	AATCTGGAATAATGTCAGGCAACTGGTTATTGGTGATGTTAACATAACTAATCGTGCATGTTTTTACTTGCACAAAATCAGCAACTTCAGGTCTCCGAGGTTCAAACATTTTCCTCGGTG
**CoV-bait-2682**	ATTAAACATATATTTTCTCATTAACACCAAAAACAAAACTCTCCATCTTACCTTTCAATCACACCCAAACAAAACCTAAATATACTAATAATCAACTACAACACAAACAAAACCATAAAC
**CoV-bait-2683**	GACACGGCGGTTCTAATTCCGTATATATCGTACCCGTACCATCCGATTTCGGAAAACGCGCCCATTTTAAATCTTGATGATCCGATAATAACGCTAATACAAAACGACCACCTTTCGAAT
**CoV-bait-2684**	TGTAGAGGATTCTGCTATCTTTGGTAGTGTTACAAGTGTTTTTGGTAGGTTGTTAACAGCAGGTGCTGGGTCAGAATAAAAACCATCCTGCAATTGATGAGTAGATTGCTGGCAAGCTAA
**CoV-bait-2685**	TCATTATCAACCCAGATGGTAAAATTTCTGGTAGCAGTTATGCCTAAACCGGCTAATAACTGCGTGGTTGGTAATGTTTTAACATGCCTGTTAGTATAGTGCTCAAAGGCAGTAGAAACA
**CoV-bait-2686**	CGCCGCGCACGCGCCTGGTGGCGCGGCGATAGTAGCCGATCTGATCGTCGGGGCCGCTGTTGGTGTTGATGGGCACGCCCTGGCCGCGGGGGAAGCGCAGCTCCTCCTTGCCGTGCTGGG
**CoV-bait-2687**	CGCACACTTGTAAAGACTGAAGTGGTTTAGCACCAAATATGCCTGCTGACAACAATGGTGCAAGTAAGATGTCCTGTGAATTGAAATTTTCATATGCTGCCTTAAGAAGCTGGATGTCCT
**CoV-bait-2688**	AGTTCTCCACGTTAAAAAACGTGTTCGCGATCAGACAACATCTTTATTGTGCTAAAACATCCCGTCGGCACACCTGGACTGGTGTTGGACCAGCCTAGTGAGTAGGAGTATTAGTTCTCA
**CoV-bait-2689**	CACCTAGGAAAACATTACCAGATGAAATGGAGAAGTTGTGGAGGCGTAAAACAGAAAGGGCATAGTCATAATCTATAGTGCTAGTAGTACTAGACGCTATAACATGGCGTGGGCACATAA
**CoV-bait-2690**	TTTAAAAAACATAATCCAAAACAAACCCAAAAAAATTTCAAAAACCAAAACCTAATCAAACAAAAAACTAATTACAAACATTAACCACAAATTACACAATTTACTCCAAATACCTCTACA
**CoV-bait-2691**	AGCATACTTCAAATTCATCTGAGTAATTGTGGGTAAAACATTGCGTTTGGTGTATGCAAAAAGCTCATCCTGGTCAGCATAACTGAGGGATTCATAATACAGACGGGCTTTACCAAACTT
**CoV-bait-2692**	TGTGAAAACAAGAGTATCAACAGTACCATCTCTAACGAGCACTTTGTCATTAACCACAGCTACAGGAAGCTCACCTTCAGCACCAACAAAAGATCCTTTCTTTACGACATTGAAAGCAAT

**CoV-bait-2693**	ATTACGAGGAGACTCTGAACGCTGCTCTAAATCTGCAATTTCACCAGTGAGATTAAGATAAGTGGCATTAAAAACATCTAAAGGAAGACTTGGACCAGTTCTATTGGGCAGAGAAGCTAA
**CoV-bait-2694**	AAGTATGAATCAATAAGATCACTAAGATCACATGTGTTATGCTTGGACAAAAAGAATGCAATGAGAGCAAGTAATTTTTCAAGGACGACTTCAGGGTCATCACACAAATTAATCTCATTG
**CoV-bait-2695**	ATTCCTCACTAATAGAGGAAGTGCGGTAGCAATTGTCATAAATTGCACGTTGCAATTCCTTGACAGCAATGTTCCTACAAACATTACTATCAACAGCAAGCAATTTATTTACATTAGCAC
**CoV-bait-2696**	GCATAAGCCGTCGTTGCATCACCCGATGTCGTACCACCTGGTTTTAAGTAAAAACCACCATTAGAATAGACTACCTCAGTTAGCACTTGAGCTAACTCATTGCATAGTCTATAATAACGA
**CoV-bait-2697**	TAAATGAAGGATACTTAGTCACAGGAACTGTAAAGTTATAAAATAGGTCTCTAGGGCCAGTACCAGTATTTTTCATAGCGGCAATACGAATATAACCCTGGTCAATACGGCCTGTTAAAG
**CoV-bait-2698**	TCATAGCCTTCTTCAGAGCCTTAATGGTAGACACAGGTGCGCCTGTAGTCACTGCAGTGTCATAGGCTTTTTGAGCATTTTCGTAATCCACATACGAGGACAAGTTGCTAAACTCACTGG
**CoV-bait-2699**	CATCGTGAATTGCAGTAGGGTTAAAAATCTTCAGAGCAGCTTTCATGACATGTGACTGCACTATGCGACCAGCTTTATTAATCTTGTCTTCATTGTCAATAAAGGGTAAACAATTGACC
**CoV-bait-2700**	GAACTGCTTCACGATTACCCGATGGGCGTTTCTTTGATTTCTTTTCGGCAGACTCGGCAGTGTCATCAGTGGCAGCCTTATCGTCGTCCTCAGTTGCGCCAGATACAATTTTGGTGTGCA
**CoV-bait-2701**	GTGATGCAAGCCATAAGGATGCTGAAAGCAAAAAAGACCCAGTTGACCTGAAAGCTAGCCCATGCATCAAAAAGTGAAAGTGCCAACACAAGAGGCCAAAGTATCCATAGAATAGCCATC
**CoV-bait-2702**	GTCTAACAATGTGCCAAGGTTGACCCTTTCTCATAAGCGGAATCAAGTGTGCAAATTGCTCACCTGGTGGTGCACGAGCTTTTACAACCTCAATGCTATTACCTTTTTCAGTAATAACAC
**CoV-bait-2703**	GTGAGCACACTCAAGATGTCTTCCCTCGACAAGATATTGCATATCTTTGTCATGGTAAATCTGAGTTCCAATTGGAAGGTTAACTGAGAAAGATGTTCGACAACCAACATTCAATCTTTT
**CoV-bait-2704**	CATGAACATCAGTTATACCCATGGACACAGAATCAAAATACTGTATCTTTTTATTATTAGCATTAGGTTTATCCGAACCAAGTAGATTAATCCAGCCACATCGCTGTGATGATCCACAGT
**CoV-bait-2705**	AACGTTATAGAACATGCTAGAAACCTTACCACTCTGAAGAGAGACTCCATACATCTGCTTAATAACTTCGGTAACTGTAAACTCATCTGTGAGTGTAGTAAAACCAAGTATGTTCTTACC
**CoV-bait-2706**	TGACAGATAGAATGGTAGGCTTGGTTACGAGAAATGAAACCAGAGTCATAATAACAGACAGATTGGAGGAATCTGTGGAAGTCAAATTTCTGTTGTAAAGTGAAAGAAGAGTAAAATGTT
**CoV-bait-2707**	CAAAGTGCAACGCACTTAAGCATGGCGTTAGATACTACACGGCCTAATTTTGCATAGACCACGTGTATGGCCTCATTCTCAACAGGGTCATACCAGTCAGGATTATCAAAGTAACTACTG
**CoV-bait-2708**	TGTAAGTTTTAGGTTCATGTAAAAAGACACCAGTACCTTCAACTTTCACAATAGTAGTCTTCTTAACATTGTTACAAGTGCCACACATACCATAGTCAAACGTATCACTTGTACAACACA
**CoV-bait-2709**	TCATGTTTGATGAGTAAAGGCACAAACCGTAACCGTGTTTAGATTTGGTTAGTGTGGTTTGAAATTCTGTGGATGTCATACAGTCTTTGGTTGTTACTTTGAGGTTTTCAATTGTACAGA
**CoV-bait-2710**	GGTATTACATCATTGTTGCTCTCCCATTTAACATACTTAAGGTTGCTGTCTGATGCTATAAAAGCATATATAAAACTCTTACCACACTCAGAAGCCATAAGCGCCTTACCACTACCATAA
**CoV-bait-2711**	CCTTGCCCTTCTTGGATTTCTGGGAACGTTCCTGTGAATTACTTCCTGAGTTATTAGTTGAGCCTTTAGGCGTCTTTTGCTGGTCTGAATTATCCCTGCTTTGAGAGTTAGCACGAGACT
**CoV-bait-2712**	AATTAGCATTATTGTTAGACATATTAAAATACACAGGAAGCGTATTACCCAACAATGCAGGAATAGGCCAAAGGCAGGCAAGCTGTAAAGGTTGCACAGAAACAAACTTTCCAGAAACGG
**CoV-bait-2713**	TTAGAAAGTGGGTAGGCATCAATAGCTAGTGAGACGTACCTTTCGAGCAGCACAACAGCGTCGGTCTTCACGACATCATCAACAAAAACGCCGGCAGACAAAATGCGTGATGGGTCTGGA
**CoV-bait-2714**	ACCTGCTGGATTGTGATCGTACACACTCGTTAATTTTCTCTTTTGATAATTGTCTGCTAGCACGAATTTGAGCTTGATTGGTTAACGTTTGCGTAACAAATGCATTAAGCGACGCAAGTC

**CoV-bait-2715**	GAACGATCGGTAATCAAGACGGAAGCTTCAACCCTAGCCTAATTCCGCAAAAATCTACCCAAACTGTGACCCGCCACATTAGCGTAGTGACGGAATATCGAAAGGGTAGAATGTGGGTAC
**CoV-bait-2716**	TCTGTATTTGTAAATTAGCAGCAACATTATTCTTTTCATCCGCAGTTAGGTCTTGACAATTATATGTTGTATCTAACACAGCTGCAGTCTGCACATTAGTAGGTATGGGAACTTGGCCAA
**CoV-bait-2717**	TAACAGAGGAAAATGTGCAGACAAAGTAGAGGTAAATAACATAGAATGGTACTGCAAGTGCAAGGCACTTATACATAAGTGCGATGCCAATTAAGTTAATAACAGCCAACCCACAAGTGT
**CoV-bait-2718**	CACAAGGAGTATCGGAATAGAAAAAGAACGGTAGTGGCTTCAAATTTACGAAGGCACTCTTATTATATGCAGATGTATGGAATGCATGCTGATTCACATAAAGTGAACCACCATTACATC
**CoV-bait-2719**	CCACGCAGTTTCCCAGCTGACTTGCGATCTTAGTATCGTTAGCGAATTCCAGCTTGGGACAGACAGAGTTAGTGTCTGTGCCGTACTGCACTGTGATTCCGAACCCCATCTGCAGCTGCT
**CoV-bait-2720**	GCTAATCCTCTATCACCAGAAATGCATAGTCCAGGACTCACATTTACCGTTGTAAAGGATGTAGGCACATAGCTGAAATGAATAAAATATAAACCGTAAGGCGCATTCTGGACTAATGAC
**CoV-bait-2721**	CTGGATCATATTTAGAGAATGTTGCGGTGTTAAGTGGTTCATCATTGTCATCAAATAAGGCCGTGTGTGTTATTGCATGGACTAGGGATCGTTGACGTTCGCAGTTTTGGAAGAGGGGTA
**CoV-bait-2722**	TAAATCTTGCTGTGCAGAATAAAAGCCATCAGCAAGAGTATGTGTGGAGCGTTGACAAGCTATATTGTCAATGGTGGATGATTCACAATAAGTAACTTGTGTAATAGTTGTATTTGCAAG
**CoV-bait-2723**	TATATTACTAAAATCCTTGTAAGCGGTGGTTGTTGCGCTTCTGGAGCAGTTATACCACACCTCTGTAGGGTAATAACCACCAACAACTACACTTCCTTCTTCTTTGAAGGTGTGAAATAG
**CoV-bait-2724**	ATTTTTCGCAATCCAGACTGTAGTGTCGAGTTAACACTGACAGTCGGTGGCGTGTAAAGCATATCATTCCTTGAAACAGAATAATCCATTAATGCTTTACCCAAATGAGCATAACAAGCC
**CoV-bait-2725**	AAGCAGCAACCATAACCACAGGTAGCAAGTACACAGTCAAAAAGGTGTGCTTATGCTTTATAAACATAGCCATGAAACCCACACAACAGCTAATAAAACCCAGCAATGGCAATAACAGCT
**CoV-bait-2726**	TGTGACAAGAAAACAACAGCTGCACAAACTGAATAATAGAACTGCAAATAATAATGACGCATATACACACTATTAACCAAAGTAAAATGTTGACAACCAGCCCGTGGTCATCAATCAATG
**CoV-bait-2727**	AACCTCGCCTTCTTCTTCAAAGAATTGAACGAGGGGGTTAGTGCAAGAACCGTCAGGTGTGTGCAGTTCATAAGTCATGTCAGGTCCTGCGTGTTGTGAGAGGACACATGCTCTGGAAAT
**CoV-bait-2728**	GTAGGTCTATGTTCTTTCGGGGCGCACCGGTCCATCTCGCATTGTAACTCTATATCTAAGAATGCGAGACGTCATCGTTACCCGACGAGCCCTAGTGCGTCAATCGTAGTCTATGTGACC
**CoV-bait-2729**	CCATCAAGATCAGTATAAGCACACACTTGCTTAGTAAAGTTTGAGAAGGGACATTCAGCTTCATAATCCCACAACACAAATTTATGTGTTGCGACAACACCTAGATTTCTAAGTATTGTT
**CoV-bait-2730**	TCAGGTCCACAATCATGCCAAGTAAGTGCATGAGCTGGGTGAACACCTTTATAATCAAAGCCACATCGTTTAAAAAGAGAAGTCAGTGAGCGAGTACTCGTCTCACCCCCAGGAAGTACT
**CoV-bait-2731**	GACCACCAAAAACAGCAGAGACAGTAATATTAACAAAAGAATGATCATTAAATGCTGGCAAAGTAACAAAAGAAGTTGGCTGTTCATGACTCAGAAGGTTTGTAGAGGAAATAGGGTAAA
**CoV-bait-2732**	GTTGAACTTCTAGGTTGATTGCTTTTTGTGAGTAACCTACATTAGCCTGTTGTGTAACGTCAGTGTCATCCACTTGTTCCTCCTTTTGAAGTGAGAAAACAACTGGATACATTGGAAACT
**CoV-bait-2733**	AGTAGCCCCGAAGATCGTCAAGCGTACCCCCGAGGACATGGCTTCTTGAACAATTTCCCTCGATGTGTAGCAACACTCAGCTGGAGCGGGAATAACCGTTCACTCAAGAAGCGGTGTTAG
**CoV-bait-2734**	TCTGATGAAATTGCCTATTAGTCATAGTGCTGCAGATAGAAACACCAGCAACTGTGCGTGCCCTATTCTTTGCGCTAATTGCGTACTTCAAATTCATCTGTGTTATGGTAGGCAACACAT
**CoV-bait-2735**	AACCCCCTTGCGACTTACCCGCTGCAATTCCACGGTCGGGACGGACCCCCGCCGGAGGGTTCCCCGATTCGTCCGGGTGGTGTGCCTGGACCACCAGCATCCTCCGGTGTGGAACTGTTG

**CoV-bait-2736**	TCCACCATTGGCTTTTACATAAAAACTACGTTTCACTCCATTAACAATAGTGTCACAAGAGATACGGTCGTATTTTGCACTGCGAGAACAAGACAAACATTTAACGTCATTGCATCCAAA
**CoV-bait-2737**	TGATAATCTGCCGGTGATGAGACGGTCAACCTGAACATCGGCTGAAAGAATGTCCAGTCGAGAGTAAATGTCATCAATAGAACTAGAAATGGCTTGGAAGTTGTGTTGCAGCTGTACGGT
**CoV-bait-2738**	CAGACTAAGTGATCCTGTGTAGCCCCACTGCTGTATGTCTATAGCAAAGGGGTTATACAGGTAATCACAGCCTGTTGCATGGTTGAAACAATAGAACGAGTGTGTCGCACTATTGTAACA
**CoV-bait-2739**	GCACCATAGTACCATCAACATTAAACTTGAAAAAGTCATGCTTAGCTACAAAGGAATAATCCTTAAGCTTATCATAACATTGTTGCTCAAGCACATAATTATCCTCAGTGTGGCGCTTGA
**CoV-bait-2740**	GCAGTCACCTTTTCGCTGCCCTGCACAGTTGGCCGGTTCGTAAAAAGGCTATCAACCTTACGTGCCCTGAGCCTACGAGCGTGCGATCTGCCCCACCATCGCAATGTTTCGAAGCCAGTA
**CoV-bait-2741**	TTGACCTTTTCGATGGCCTGAGCAGCACTAAATTTAATAAGCGTACTATCACTGAGTTGCTTGGATATATACGCATTAAGTGCAGTTAACCTGCCATTAATAAGACGATCTATCTGGGCC
**CoV-bait-2742**	AGCCTTCAATATTAACCTGCTTAGGCAAATAACAACCCAGAGCATTATTGGAGGCTACGGCTAATTGCGCTACTTCCCTAGTGCACTGCGTAACCTCATACAAAGCAAAGTTATGTCTGA
**CoV-bait-2743**	AGCCCAAACAGTCACCACAAGGGTGAATGGCTGGAGAAGCCACAGTAGGATAAGTTTAAGCACGTAAATCATACGGTTGCGCGTAGCATAGCCACCCTGAAGGATGACGCCAAGCGCCCA
**CoV-bait-2744**	AAAACATCTACGTCTTCTGAGCCAGTGGCAGATGCTTTATATAAACCAAAAACATTACCATTGGCACACAATGGGAATGACAATTGAGGCTTGTGGTCTTTACAATAATAACTAAGACCA
**CoV-bait-2745**	CATATAATTATATGTTCCCATAGGCACAGTTGTGAATTTATTTAAAATCCACAGTAGACCAAACCTCATACACATAACATAACCTATACAAACATAGATAGTTATGCGAATAGCAAGGTC
**CoV-bait-2746**	AGGCTTTATCATAATCAATCAAAGTCGTTGTGTTTTCAGCTATAACATGGCGCGGACAGTAGACTTCATCTCCCAACCACACACCATTCAAAACCATATTACCGTAGGCAACACGAACTA
**CoV-bait-2747**	CCTGCGCAAGGACTACCGGGGCCTCTTTAGGTCTCGGATTTACGTCAATGCTTAGTACTTCGTCGCTGCTGGCTTCCTTTTTCTGGAGTTTGAGTAAAAATTTATCGACATCAGGAAGGT
**CoV-bait-2748**	CAGTTCCAGTCACTTGGTCAACAAGCAGCTGCTGATAGTCAGGGAGCAGGTTCACATCCTCCGTGACAGGCTTACCCTTAGTGGTAGCAGTCAGGAGTTTGGTTGAAATGGCTTCAGCCT
**CoV-bait-2749**	ACTTTAAACTGGTCATAGCACTCAACACGTGCTTTCGCAGGTATAATGCGTGAACATTTTGCAATGTTCAGGTACTTAAAAGCTTTCTCACACAATGCATCTACGGCTGCGTGTGAGCAA
**CoV-bait-2750**	CATGGCGATACTATGCTGGGGTTAGCACATGGGCAATAATCATTACCAACAGTAAAACACTCTTCAGCATATGCAACGTCATGTTTACCACTACCAAATGTTGCCACATCGTTAAATCCA
**CoV-bait-2751**	GCTGTTTTATATAACCATTTGACCAATTTGTTAATTTAGGACCACACATACAGGATATAATTTTAGCAATGCCTCCTCCATGTTTAAGCTGGCTATTAGCTGGGTTTACAACATAATCTA
**CoV-bait-2752**	TACAACAGTCGTATGATTAGCATACACAGGTGTAGTAACGAATGTTTCAGGTAAAGCCTGCTGTTGAGCGGGCGTGTATGGATAGACTCCATCAGGCAATTGGAATGTGTTCATTTCACA
**CoV-bait-2753**	CTTTATCGAACAAAATGTCTTCAATAACTGACCTAGCACCAGGATTTGCTGGAAGAATATTGGTAAAATTATAACCACCACCAGGAAATTGGCTTATAGTGGCAAGCTTAAGTGCCTCAT
**CoV-bait-2754**	CAAACGTTTCCCGAGATCTACGCCTCAAAAGAGCAGCTTTCGATTATTCTGGGCTCCGTTTACTGTCGGCAATTGCGGCTGGTATGCAATGGAACAAGCTGGGTAACCAACAGGAACGGC
**CoV-bait-2755**	AACCGCGTGGTGGCACCACATGTAAAATACATGAGACACCATACTTAGCGGCATCAAAAGGTTCTGTAGTAACATTACCTGTAATAGGCGCTATGCTATCACAGTAAGCCTGATATTTAG
**CoV-bait-2756**	CACCGCTTTTAAAACAATGTGTAACGAACACTGTAAAATCCGTAAAGTTACAGTGAGCCGTGCAGAATTGATTAGTTGACCACTGCATCCCTGAACCAGGTGCAGTCATGGCTACAGAAG
**CoV-bait-2757**	TTGTGGAATTAAAGAACCCAGTATTATTAGCACTACTAATGACGCCAACAATCCTATCCTCAACAAAAGCAACCTGCTGAACCAGTTGGCAGGGTGTAACAGAATAAATCTGCCCTGTAG
**CoV-bait-2758**	AGGAACTAACACCCTCAACAGGTTGTGGGACGCCAGTAATTCCATCACCATCACTCCAAGAAACATACAATGAGCCTATAGTATAGTACTTACTATAAGCCCAATTAGCCACTATAGGCA

**CoV-bait-2759**	GTTGTCAAGTGTTAACACACCAACTAGTCCTGCTTTTACCATAGCATCTGCCATTTTGTTGGCGGATAGCACTGCATTACGCACTGTTTCTCCCAACTTATGGTAGACTGAGATGAGTTC
**CoV-bait-2760**	ATCTAGCAGCGCCAAAAGGTGGTGTACACTCATTTTCATCAACAATAAGCTTTGTAAAATAAAGATGACCACTCAACAACCTATAAACAGAATAAGAAATGGATGGAACTTTCAACGGTG
**CoV-bait-2761**	ATTGTTTGTGGTTGACATGGTTTAGAGTCATTGTAAATTGTAAAAAATGAGGATCCTTGTGTGTAGGTTTCATAACCGAGACAGTTAGGTGGAATACCTGTAACAACTGCAATACCTTCT
**CoV-bait-2762**	TTACCTAAACGTCATCCACATCAAGGACTGGTGGTTTTACATCATTGTAAAACTCATAAAACTGCCTACCTCTATTAAACACATAAATAGAAGGGGACAGTACTAAGGTATTACACATAC
**CoV-bait-2763**	ACTTATCGAAGTTCAAGGAACTGCCATTCAGCGTATTCTTTATTGTGATGATCCTGTTAGCCAACTCAAGTGTTCTCAGGTTGCTTTTGACCTTGACGATGGTTTCTACCCTATTTCTTC
**CoV-bait-2764**	TTTGTCTGAGATGTTCTTTTGTTTGTTGATATTGGGTGTAATTACCAGGGTCGAGCAATTGGGCAGGATTTGGTGAAAGTCCGGCTGCGAGTAGAAGGTGTTCAAAATCGCCGAGTTGGT
**CoV-bait-2765**	GTGTGCAGTAGTCTGGAGGCATCGTCAGGTTCACCATAGGTACAATCACCATATGCCATTATTAGGGCAATGAATTCAGTGGAATCACCACCCTTATGTTTGAGGTACGCAGCTTGTAAA
**CoV-bait-2766**	ACTACAATGTTAGTAAAATTACAGTGAGCCGTACAAAAGGCTGTGGTAGACCATGCCATACCACTTGGTGGTGCAGTCATGGCTATTGAGGCCGCACTAAAATTCTTACTGTAGCCAATA
**CoV-bait-2767**	TTAGCGGCTCCTGCCTATCCAACGTACTCATCCACGGTTAGCTCTAACGTTGAGGTGGTGGGGGTTCCCTTCTATATTACAAGCTCCCGTTTGAGCATTAGGCAGACAGTTAGCGCATGC
**CoV-bait-2768**	ATACATTAAGTTTGTATAAATCCTGTTGTGAATTATCATACACACCAGGATAGGGCACAAAAGTTCTATTTGGAACAACAGGAACTACACTGTCGTAGCCATACAAACCATCACTTAAGG
**CoV-bait-2769**	CGTAGCGACTAGAAAACAGGTGCACGCCCTGTGCGGTCTGAGTGATTCCGAACCACTCCAGAATTTCGTCCTCGGTGATGTTATAAGTGTACATGAATGTGCAATTCCGCAGGTTGAAGT
**CoV-bait-2770**	AGGACAGGTGCCACTTGTGATACTAGCTTCTGCATACAAAGCATTATCACAACCGTTATCAAAGTTGTCAGACCAACCACCACTCTTACACTCAACCTGCAAAGAAACGGAGAATTGGTT
**CoV-bait-2771**	ACGCGGTGCCGGTAATTGAGCAGGGTCACCTACATACACAACATATTGGTAATTTATCTTACCATTAATAAAAGACAATTCATAATTAGTCAACATACTAACCTCGTCAACCAAAAGAAT
**CoV-bait-2772**	CGTTCTTATCAACAAGCGCAACGTTCTCAAATGGTGGGGGCTCTGGTGCCTCTGGAAACAACTTGAGGTCGTCACACATCTTCTCATAGAGCACAGAGAGCAATTGTTCAACTGTGAGGT
**CoV-bait-2773**	AAGCATCCACAACAACAACCACAACAACCAGTCATGAAAAAAACCCATCCTAATATTAAGATGAATATTAAACAAGCAGAACCAATTGCTAACCAAACATACCAAGGCCACTTAATGTAA
**CoV-bait-2774**	TATAGGGACACACGTTAACAGTGTTACGATACAATGGTGTTTGATTGGTATAGTCCCATATAACAAACTTATAGGCTATATCAACACCAAGACCACTCAAAATGCGATTATTGGGTAGTG
**CoV-bait-2775**	CCGTTATACCACCAAATGCCATACCTCCCAAGAGTGAAGTAGTGTAGAGACCCATTAAAGTAGGGTCTACCACGCCTGGTAATACAAGAACACCATTATAGTATTGCGCACAGGTGAGGT
**CoV-bait-2776**	ATCTCTAGTGTCATGCCAGCTAATGGCAAGGGCAGTTTTATAGAGGATCTTTTGTTTGACAAGGTAGTTACTAATGGTCTGGGCACAGTTGATGCAGATTATAAGGCTTGCATCAATGAC
**CoV-bait-2777**	GTACTCCAAATTTGGTGGATCTCGTAGTGGAACCCGACAACCATTATTGGTAATAAAATGCGACCGCATAGACTTTCCATAGTACCCACTCCCAGGAGAAACGTACATTTGTGACTGTTT
**CoV-bait-2778**	CTGAGTTAGAGCAACCACACCATCATCAATGACACAACCAAAATTGTAGTCATTATGACTGACAGCACAGAAGCTGGCAGCATCAAAACCGTAGTAACTTTCATAATCAATCGCAGGTGG
**CoV-bait-2779**	ATGATCTTCTGATTCTCAGAAAGAACCTGCTGAGTGATGCCAACACCATTCAAGCGATAAAAGATACTTTGTGCAAATGGTATTGCTGCGAAAGACGACAGACCTGCTGTCCAACTAGCA
**CoV-bait-2780**	CTTGAACAAATTTATACTTAGGAGTGTTAACATTTACAGTTGCTGTTTGTAAAACAAGAACTGCACCCTGTAACTTCCTACTGACGACATGTAAAGTTGCACCATCTGAGCTAACAATTT

**CoV-bait-2781**	CTGAGGATGAAATCCTGGGAAGTACCAGAAGCGTCGAGGTATGTTAATTCTTCCATGTTTCCGTAGAGTGATGTCACTACAGATGTGTTTGAGTCTGATGATGTGAGTGGTGCTGTTGGG
**CoV-bait-2782**	GGGTAGATTGTGATTTTACACATTCGTTGACTTTCTGCTGTGCAAGACGCCTACTGGCTTGAACTTCAACATACCTAGTCATAGTCTGAGACACAAATGCATTAAGTGCAGACAAACGTC
**CoV-bait-2783**	GAGAGTTTGCTGATTAAGCGGTTTCTCATCACGCACAGTGGTCCAAGCGCAATGGTATTCCTCTCCTTCAAATTCAATAATGTCCTCATCACCGAAGTAGTCCTTAAAATCACCTTCAAT
**CoV-bait-2784**	TCTTGATCTTCTACAACGACTTGTTCAGTCATTGTCTTAGAACTACCAGTAGAATTTTCGACTGGTTCTTCAAAGGTATGTTGCAGAGGCTCTTGTTGACACAGTTGTTCTTGCGCATCA
**CoV-bait-2785**	TACCGTCAAATGAAGAGCCGGTATGTGTACCATTTGCCAATTCCATTTGATGCATATAACAAAAGTTGATCACATTACCCTCCTTTGTGTAACCAACACTACCACACGATCCACACAAGA
**CoV-bait-2786**	ACCTCCTTCTAATTCAAGTCCAACAGACCCAGCAGAAGTCCTATAATCCGACTGGGATATGTTGACATTAATTCTGATATTGTCAGTAAGACCTTTCGTGTTGTTCCATCCCTTAAACAA
**CoV-bait-2787**	GCCAATTCTTGTTGAAAAACTCAGGACGCATGTCAACTTCCATACAAGAAGAGGAATTGGAAGGGAGACCAGGATCAACATCTCCTTTTATAGGTGTTAACAAGCACATCAGTAGAAACA
**CoV-bait-2788**	CACACCAGTTGAAAATCCTAATTGTAGAGGCACATTGGTGCCACATGCATTACGGGAAGCATGAGCACCCTCAACATCGAAGCCTATCCAGCTTCGAACTTGCCTTACAGCCTCTTCACG
**CoV-bait-2789**	ACCATTACCGCAGCCTCCACAATCAGTGAACGCAGAATGGTAGATGTAAGAAGGATCTTGAAGAGACTCTTTACCATAGAATGTGTAAAGAATTCTTTGTTTGAGAGGCATTTTAGCTAT
**CoV-bait-2790**	AATAGCTAAACAACGAGTCATTATTGCATCATTAGAAGCCACATGAGCTCCTTGATGGACAGAGCAATAACGATCGTGATTAGTAGCAAGATTGCCTACATAACCCCACTGTTGAACATC
**CoV-bait-2791**	TTTAACATAGTTACCATCAGGCAAAAAGTAATGACTGTCATGCAGCAAATCTCTATAGAGACCACTATCACTGACCAGACCACTACGGTAACAATAAGTAGAAGTACCACCTAGACCTTT
**CoV-bait-2792**	CATGCTAACAAAACTGCAACAATGAGCATTATAACTTGTTTAAACTCACGGTACACATCGAAAAAACCAGAACCACTTTTACGACCAATAGCAACAGTTGGTAAAGTGTGGCTCATGGGT
**CoV-bait-2793**	CTAACTGCTTCTTGGAAAACTCACCAGGCAAAATAACGTCAAGATCTTGTTGAACGGACTTAGCCTTAGTGGTGACACAGTAATAGGTTCCCGATGGGAATATTAGAGACTCCCTGCCGC
**CoV-bait-2794**	ATGAACCACGCGGCTGAGCTTCGAATGAAGAAACTGAGTAGACTCCAGTATCTACATCAAAAGACTCATAAGAGCAACGAAGCTGTGCCAAGTCATCGTGGCCACAGTCTATTGCCCTAC
**CoV-bait-2795**	ACATTACCATCTATAAAAGGCCCTTGCGCTAGGTCACCAAGATAGTTTATGCCTATGACAAAGGCCTCTGACGACGATGTATTAACAGAAGTGCAAAACATAGTCCAAAAAGCGAATTTT
**CoV-bait-2796**	CTGCGGGCAGCCAAAATCACCAATCTGATGGTTGGTGGAGGCATAAATCCACGCAACGAGGCGGCTGGGGTTACCATTGAGGTACTCTTTGTAGAGTTGGTCAATAGCAGGACGTAACTG
**CoV-bait-2797**	TCGCGAGACAGACGGTCATTATCGCATCTGCTGAAGCGACATGAGCGTTAGAGTGATATGTGCAAACCGCGTCATGGTTAGTGGAGAGACGTCCCGAGAAACCCCACGTCGCCACGTCGA
**CoV-bait-2798**	ATTCTCAAAGACTTCAACTTTGGACTGCACTGGAACCTGTGGCGTTGCAGCAGCGGGCGGATCCTGCAAGAGGATGCTCGTCTGGTCAACTTCCTGAACAGGTAGCACAGAGAACTTGTT
**CoV-bait-2799**	CCCGAGAAGGCCTGCACATAAGCATATTGGGTTGGTAACAACACAGTGTGGAAGAATAACAACCCTTGTGGCGCTGCATTGACAATTGAGAAGAGATGGGTGCCGTTACCACAGAATCCA
**CoV-bait-2800**	AGCCGTTAGGGTAGTAAAATCGAAGGCATAGTGGGGTAGTATGTTGTTAGGGTTATCAACAGTAAAGTCAAGACCACGCTGGACGGCCAGGATTGCAGCATTAATTGCCGAATCGGTAGC

**CoV-bait-2801**	TGTTGGGTCAGCAATAGGTAAGTATGCATTTTCAAAAGTACTAAAAGACTTAATTCTGTCATTACGTGGCATAAGCTCAACCCAGTCAGCATCTGTTTTATGCTCAACCTTACACACGAT
**CoV-bait-2802**	AGCACCATCTTTATAAGAAACAGTGATAGTGGCGAACTGCGTACGCCACTTGACATAATGAACTAGTGCCCACTTACAACCTGCAACAGCTGGGTTCACATCAAAACACAGAGTTTCAAA
**CoV-bait-2803**	GTAGGCACTATATCCCTGAGAATTGCATTGTCTATTACCTTAAAGTCAATAACATCATAAGGCACTGCAGTCATTGTGTACCATGGAGTAAACCATTGAGGGTTTAGCACCACTAACAAC
**CoV-bait-2804**	GTCTCGCGTATGACGCCCGTACCCGTCTTACCATAAATGGAGTATTTGGTACAGACATCAAGATGGACAGTGGAGATGTCAGCAACACCAGTGGAGGGCTGCGGCAGAGCTGTGATGTAG
**CoV-bait-2805**	CTGAAACTAAAGTGTTGTTAGTGCAATAAAACACAACATCACCATAAGATGTTTTAAACAAAATGGTTCCACGTCTAAGGTTTTCTTCCGAGTTGGATGAAAAGTTGAGGTTGTAGCGTA
**CoV-bait-2806**	CACTAATAATACCACCAGCTTTTTTCTCTACTAACAACTTCTGAGTACTACAAACAAAAATTTGTTTAGCGCCAGATCTTGTTATAAAGAAACGAGCCCCCGACTTGACAGTTGCTGAGA
**CoV-bait-2807**	GTAAATGAGTAACAGTAGTTTCATAAACTTAGCAAGCAGCCACCACTTAGATTTAAGAACAGCTGTTGACGCTTTTAAGTTATATTTAAGACTACGTTTTAAAACAACACCCGTCCTTTG
**CoV-bait-2808**	AGTGTCAATGTAGGCTCGCAATTGGGTGACACCTGTCACAACCATAACAAAGTCAGAGGGTTCAACACCCTCGACAGTGTTAGCGAGACCTGCAGCAACAAAACGGCATTGGGTAAAACC
**CoV-bait-2809**	GTCTCCGAAACCATAAAATTCAACCCAATCAACATCTATTGCCTTAGTAAGCAACTCAGATGTATTAAGATCGCTTGGTACTGAACCAGAAAGATCTTTGTCTTTAACAGCAATAACACC
**CoV-bait-2810**	CTCTGATTGGCAACAGGGCTCTGACTCCTAGAAGGCTTCGGCGTTGATTTTCTGCCTTTCTTTTGATCTTTTTCCTGTGGTTTTTCAAAACCTAAGGACTTAAGAGCAGCAGCAACAGCC
**CoV-bait-2811**	AATCAAACACACAAGACCACAAAGTAGCCACAACCATGTTGATGAATGGCCAGCATCACCAGCACCTTGCTTAGCAACAATGCTAGTTGCAGGTATTTGTGTAACAGCTTGGTTTTCATT
**CoV-bait-2812**	TTAATAAAATTACCATTGTCATATTTATAATATGCGTTAGCTTGTATTGAACTGTAAGGCAATGAACCTTCCATAAGATCTGTGTTATAACAATAAACATTGTTACCACCCAAACCTTCC
**CoV-bait-2813**	ATGTATGCCTTATGTAAGACACCATTAAAATCAACTGACAAAGTAGACAAAAGTTCACTGTCAACTAATTTAATAGGCCTGCACAACAACTGTGAAAAATAAACACTAGCATTTTTAACC
**CoV-bait-2814**	TTCGACACTGACTATAATAGCTCTACCGCTAACACTTCTAACACGTGAATAGTACTTAATACCATGGGCACAATAACCAACTTGCACACCATCACGTTTGACACTAGCACAAACTATAGC
**CoV-bait-2815**	AGAGACTGGTCCGAACGGAAGGCACTGGTCAACCCGGGCAGGCTCCACGTACAATCAGTGCACGATATTTGCGCTCAGTAATTGCGGCCTGACAAGACGCCTGGTTACATCTCTAACCCT
**CoV-bait-2816**	AAAAGATATGTATGCAATTAAATATGCAGCACACCAAATCCATGCGTAGCGCAAACTTCTAGTAGCAAAGAAGTACAACACAGCATATGCAATCATTGTTACTAAGTTCTGAGTTACAAT
**CoV-bait-2817**	AACATGACGTGGACAATAAACAATGTCACCAAGCCATAAACCATTTAACACAGTGTTGCCATAACAGACGCGAACAACGCATTTTTCCACAATGCCAGATGGCTGTGCCATTTTACGCAA
**CoV-bait-2818**	CGGTTGCAGGAGCCAGAGTAGGATAAGTTTAATAATGTAGATTATACGGTTCCGCGTGGCATACCCTCCCTGAAGGATAACCCCAAGGGCCCATATAAGAATAATAAAAACAATAATCTG
**CoV-bait-2819**	AGAGCTGGATCCTGTTTGGTTAGCTGGGGTGAAATCACTAGTTGTAAAATCAGTGTAGGTGGTAAGGGCTGTTTCAGTGGTGTTATCTGAGAAGTCACGGGCGATGATTTTCCATGTTAG
**CoV-bait-2820**	ACACCACTACCATACTTAATATTCCAATTTTATTCATAATCTTCAATAAAATCAATACCAAACTCATCACCTAAATCATAAAACTTTAAATCAATACCATAACTATAACCACCAACTCCC
**CoV-bait-2821**	AAATAACCACAAACTTAATGCATATAATACAACTTGAAATTTACTAACATTTGCTAAACTCACAAAATCAGATTTAAAACCTATGTATTGTTGAAAAAATACATCTTTCCAATTCATCGC
**CoV-bait-2822**	TGACAAAGTACATTATCCACAGCATAAGAGTGATGCAAGCCATAAGGATGCTGAAAGCAAAAAAGACCCAATTGACCTGAAAGCTAGCCCATGCATCAAAAAGTGACAGTGCTAACACAA

**CoV-bait-2823**	CATTCCAGATCGCCATGTGGCCGTTGGTCTCATAAATTTAGGCAGACGCCGAAAATTATAAGGACGGCCAGAATTAAATATAGGTTTAGAACTGGTAGTATAATCAAGGGTTTTAGGCCC
**CoV-bait-2824**	TATACGCCACGCATTTATCCAGGGTAATGTTGTTATAGTTATACTGGGTCAGCACCAGCGGTTCGGTACGGGTCTGAATACGGCTGCCATCGCTTTTGGTCACATACACCAGCAGGCCGC
**CoV-bait-2825**	ACATTGAGGGTTATTACTCTGCAAGATAGCATTATAATCAGTCAAAAACCATGGCTCACCACAATTATCAATGCAGAATTGCTCCCAAATATTATAACAGTCCTTAAAACACACATGTGT
**CoV-bait-2826**	CAAAACGACCATTATTGCGTAACAACAACCTACCACTCTTAATCAAACTCAAAACCATATCATTTACATCACTATCTTTAAGTGTAACAACAACAGTGGCTTTATGTTTACATTCAAACT
**CoV-bait-2827**	GTTACATTATATAAATGCAGACGCACAGTTTCACCAGATATAGTTATGCCCAAAGGCGCACCTAACTGTTCTGTAAATAACAAATTAACTATACAGTCAAAAGAACTAGAAGCATTACTT
**CoV-bait-2828**	AGGTTTATTGTTTGTCAATATGTCATCGTAAAGACCAAACATACCAGCACTAATGTCAATAACACCAGTGCTAGCAGCAAATTTAACATCTTCTGTAACAGAAGCACCAAGAAAAGCTAG
**CoV-bait-2829**	AAACCAAAACCTGAAATTTCCGAATCACTTGCAACAGCAAGTGTCACTTGATTGTAAAACATGGTTAGCAGTTATTGGTTTCCACAACGACAGAACGTTGCTTCCACAATAAGCAGTGAC
**CoV-bait-2830**	CCATCCTTAGGTTCAACAAAAACAGTTTCTTTAAGCTCTAAATGTTCAACATCAATGGCATTAGGTTTACTAACACCAAAACAAGTGTCTAAAAATTCATAGAAAAAGGTGACACAACTA
**CoV-bait-2831**	GTTCTATCATAAGTGTACCATCGGTTTTGACGATATCATCAACAAAACAACCAGCGCCTAAAATTCTAGATGGGTCTGGGTAAGGCAGGTACACGTAATCATCTCCTTGTTTAACTAGCA
**CoV-bait-2832**	GGAAAAATCATCCAAATCTGCTGCAGGTAACAGAGTCACAGTTGCCTGTTTCTTCTGACGCTGCGGCAGTGACTGTACTTCATCAGCCTTTTTCTTCTTGTCCTTTTTAGGTTCTGTAGG
**CoV-bait-2833**	TAGGAAACCTAACAACTTCAGTGGTTGGTGTAACACGGAAGTTAGAGGTTTGATAAATTCCTTTTTCAACTGTAAAACTCTTAGTGGTACACTTAAGCTCTGAGAGTGGATCTTGAGAGC
**CoV-bait-2834**	CAGAGGACTGTGTAAATCCATAGGAGGCAAGTGGTTTGTAACAATTAAGACCTTCAGCTGAACATGTACCACCTGAAGGGTTAAAAAGAACATTGGAAAGGTCACGCCCATAAGGTTTAA
**CoV-bait-2835**	AACTTAGCAGCATCAACAACGCCTGGCAAAACCATAACACCCTGATAATACTGAGCACAAACAAGATCAGCAATGGAAAGGCCATTAGAACATGCCTTATAATCTGCATCAACAGTTCCG
**CoV-bait-2836**	GAACCAACATGAGCACCACTACCTAACTCAATTTGGTGTAAATAACAAAACTCAACAGTACCATCATTTCTAACATTATAACCAGGAGAACCACAAGCTCCATTTATAAAAGAACCTTTA
**CoV-bait-2837**	TGTACAAGTGATAGCAAACTTGTTGTCAGCCAAAGGGTGATAAGGTGAGTTGCCTTCGTAAGTATTAGGTTGGCAAGGCTCCTTTAAGAGTACGGTTGTACCTCTAGCACACTCTTGGTA
**CoV-bait-2838**	CTGACAGGCAGGGCAAACCATCTTTACTACAGCATTACTAGGTACATACCCACATGTGGTAGGTCCCTCTTCAACAGTATTCTGGTTGCCACACATTTCACAAGTGGCTGTTAAGAAATC
**CoV-bait-2839**	AAAATGGTGTTGTTGTCAAATTTGCAGGGTCCTTTAAAAATGGCCTAACATTATTAAGACACATAGCCTTATCAAAATACCTGCCCGAAAGCAAAGTATGTTGAAAATACGATTGAAAGT
**CoV-bait-2840**	AACACCTGTACCAACAGGCCTACCAACATTAGTAGGACAAAACCATTTGGCTTTGTTAGTAGATGGATATGCACCAGTAATAACAGTAGTTGTATTACTTAAGCCACGACTGGTAGAAAA
**CoV-bait-2841**	CTCTGTATGGCTGAAAGGAGTTGACACACATCAACACCAGTCTTAGCAGCCAACATAGAGAACCCGTCACCAGACACTAAATTAGTAAAACCATTACCAAGAGCCCACTCATTAAAACTA
**CoV-bait-2842**	ATGATGGAGTGTTAACATTAGCCTGTGACACAGTAATAACAAGATTAGCACCTTGCATAACAGCACCTACAACACCAAGAAAAACATTACCCTTAGAAATAGAAAAATTATGCAGTCTCA
**CoV-bait-2843**	GTGAACAAAACTCATGTGGACCTTTAGAGACGTCACTCTCAGTCCAACACTTGGCATCACTCATAAAAACATTATTTTGATAATACAACACAGCTTTAAATGCACTAATATCTGCTATGT
**CoV-bait-2844**	TGACATTAGCAAATGTCAAATTTTCCGGCTTATAAAGATTACGCGGTGACCACTGCCAGTAACTTAAATTAGCAGAGACTAGAACACCATCCTTAGCAACAATGCACTTAGAACCTAATT

**CoV-bait-2845**	ACCACACATACCACAACAGCCTGTAGCAAGACAGCACCAAAGCATAAGACCAGCAAGCAAAGTCAAAGCTATAAAAATAATAAGCCACTGCCACCAAGCCCATTTGACATAATTAACATA
**CoV-bait-2846**	TCTTAACAGCCATAGCGCCACCAAGCGCAAGTTTTTCACGTATAAATCCATTAATATAAGTGAAAAATCCATCTTTAGACACATTGTCACCATCAATGCTCTTAATTTTTCCATCATACA
**CoV-bait-2847**	GGTCTATAGGACTCAATATAATTATACTCTACTCATACTAGCATAATTACGTGCTTACCATTGTGTATGAGACTAAATCCACAAGCAAAGCTAGGGATTGTATGTCAAACAAATCTGCAA
**CoV-bait-2848**	CACCCACCATGTTTATAACAATGTGTAACAAACACTGTAGTATCTGAAAAGTTACAGTGTGCAGTACAAAACTGACTGCTAGACCAAGCCATACCTGATGACGGTGCCGTCATAGCTATA
**CoV-bait-2849**	CCACACAATAGAAAGCTTTTCTTTGACTATAACATTATGATTAACTATCTTAGCACCTGCACGCACACACTGGGCGCAGTCAAATGCTGACAATTTTTCTTCAGGCTTAGCATAACTAGT
**CoV-bait-2850**	GTCTAATTGGAACTACATTGAGCTCCAACTCTTGGACAGCATCCAAAGACAAGTTAGCAGACTTTGAGACATCTTTGACAACTGTGTCAATCGTGTATTGAAAAAGTCCAAGAAACATCA
**CoV-bait-2851**	GCAGACTGCAACTGTGGGTAGAAAGTAGCCACCTTGGAGTCTTTGCACCAAAGCATCCAGCGCCATGCCTTACAGTCAACAATGACATCATGCACTTTAGATGTCACAGAAAGATCAAGA
**CoV-bait-2852**	AACTCCTGGTGTCCAGTATAATCATCACAACAACCACCACATTTCTTAAAACAACTAGTCCCACATCCTGTGCAACAGCATATGAAGAATAGTAAAACAAGCACAGCTACACCAGCAAGG
**CoV-bait-2853**	GGGTGGGGTGAGTCCTACCTTACGCTTGGCATACAACTCAAAAGCTACGTTAGTGGGTAGTGATGTCTTGTTTGTGAAAACAAGAGTATCAACAGTACCATCTCTAACGAGCACTTTGTC
**CoV-bait-2854**	TCTAGCCACTTCATCAAAGCCCTTAGACACAGTATCCAAAGCAGATTGTATGCCTTTAAGGGCCTTGTTGAAGGAGTTCGCTATTAAATTCTGGTTATCCAAAAGCACTGACTGTGTCAG
**CoV-bait-2855**	CTCTGGGCTGATACATAGTTCGAGGAGTCAAGTAGAATTTGTCATCTAAATTGCGAAATAGCGTCAGCTGAACATCCTTAACAACAAGTCCAAAAGTGCTATTGCCATCTGACGCACAAA
**CoV-bait-2856**	GTGTTGCCTTAATTGGTGGGTAAACAACATCATGCTGTTGAATAACAAAACATGTAGCAAGACGCCATGGTTGGTTAAAATCTGGACATGTAACCTTAAACCAGGTCAAATTCTTATTAT
**CoV-bait-2857**	TACCAAATAAAACTATAAGTCTTCTTAAAACCAGCACCTTGTTTACTAACAATGCTGGTAGCAGGGACTTGTGTGATAGCAGCATTGTCGTTAAATGTCAATAAAAAGGTTAAACCCTTG
**CoV-bait-2858**	GGTCCACCTGGATTGGGTGGTTGAAAGCGATGGAAGCCAGTCTCATCTCTCCGGGCACCGAGCGAACTGAACGCGGGGTCAATGTAGAGGGGGTGTCCGGGAGAGCACACAGGGACTGGC
**CoV-bait-2859**	GCTTGCATTGAATAGAACTTCCACACACATATTGAATGCAATTAATTTGAATCTTTTGAAATTGAGTCTGTATAAACTCATCTGTAACAGTTAAATTAAAACTGTTGGGTATAAGTACAT
**CoV-bait-2860**	AACAAGGGTAGAACAGTGCTCAGGCATAGGAGTGCCAATGGGCTTTGAGAAAGCATTAGCAACTAATAAAATGAGCAAGAGTGTGGGTGGTCTTTGCACACGCATCATAAGTTCGTTAAA
**CoV-bait-2861**	AAAGACCACCAAGAATGTGTCCTGCGCCACAACCGAATGCTATTGACAAAGTACATTATCCACAGCATAAGAGTGATGCAAGCCATAAGGATGCTGAAAGCAAAAAAGACCCAGTTGACC
**CoV-bait-2862**	AAACGGAAGTAGCATTGTTAACACAATGAAGTGACATAAATTTAGGGTAAGAGGTAACGGAAAGTATTTTATTAAAAATAGACTCACCATTACGCTTAGCACCAATATATATGTTACCTT
**CoV-bait-2863**	TAATTTCCTGGTCCTCATTAACGAGGATCCTAAAATGCTTGAACACCATATCGAGGGGAGTGAGGTTTCACCCAGACGGAACTGAGATCCACGTGAAGACTAGACGGAACTAGCCAGCAC
**CoV-bait-2864**	AGCCACAAGCGGGTCATTAAGCTCCAAAACATCACTAGGATGTATATTGGTTGCCTTGCAATGTTGTACTAATGCTGCAACTTGCTTAATGCAGTTACACATTAGACGTACTTGTATAAA
**CoV-bait-2865**	TGCAATGGCGGTTACAGGTACGGATAGGAGCCCCCGCTCGGTACTAACTGTGTGCGGGAATAAAAGCGGCCTCCTGGGTCACCGGATTAGAACGACTCTTTGGTGTTTACGTAGTGTCAA

**CoV-bait-2866**	ACCAGTTTCTATTAAAATAGATTGGTCTTCAACAGCCAAATTTGAAAACTCAAAAGCATAATGAGGTAGTATATGGTTTGGATCATCAATAGTAAAGTCCAAACCTTTCTGAACAGCTAG
**CoV-bait-2867**	TCCTCAAAACTTAATGGTAAGTCACGTAGTGTATCCACATATGTACAATTACGTAAGTTGATATAGATAGCAGTGTTTGTTTGTGCACCGCCACTAATTATAAAGGCAGGGTTGTCACAA
**CoV-bait-2868**	ATCACCGCAGCGTAAGACTGTTTGTGATTGACAGACAACACAAAGGCCAGCAGATTGAAGTACGCTAGACTTTTCATAGAGACTAGCATAGAATTCTTCACTCCAAAACTTGGCTTGAGT
**CoV-bait-2869**	GCTTGACCTGCAATAACAGCAATTCTACCCTTAGGATCAACACTACCAGCGTAGTCCAAGACACGCAGGTTGACCATAGCAGGTTCAATCACAGAAGGTTGCACATCAGTTGTGGCACCA
**CoV-bait-2870**	GCAAGTAAGTATAATATTCTGTAACTAAATCAGCATCCACATGTGTACTACGGTAAATACCCATATAGAGGCGATGTTGCAATTCACGTACATATAAATTATGCACCTTATTACCATCTA
**CoV-bait-2871**	ATAGCCGTGAGCAAACCCGTTATAGAGAGACCTGATCGCGGCTAGTTATGAATACTTTGAAGGTAGTAAATCAAGCGCACACTGCGCTACTGTTTTCATCTCAGGGACGATTACATCGAA
**CoV-bait-2872**	TCTTTGAATCTTTGAGTTCATAGAAATCAAGATTCTCATAAATGGTTTTATCACACATGATACAAAGTATACCAACCTTTGCTCTCGTAATGGCAACATTAAATCTGTTAACATTAGTAG
**CoV-bait-2873**	ACCCTGGATCTTTGACGTCCTCTTGAACTGTCTCTTGATCCAGGTCTACTATTCTCCCTAGATGTCACCGCTGAAGAATTCCTACTCCTACCACGATTCAGTGCAATGAAATCCCAACGG
**CoV-bait-2874**	ACAGTCCAATACTCAAATCTTTGAATCAATTCGTATAAATCTTTATTCCAACTAAATTCTGTGATTTTGATGGCAGCAGATCCACCAAGTGATAGTTTCTCCTTAATGAAACCATTGATA
**CoV-bait-2875**	GACAACAACCGGACAGGCTGGTGAGTCAGTGTACGGTGCTTGATGAAAAGAGTCAAAAGACAAGAACTTATTAGCAAAGCAATTGTCTGTTGAACGGATAGTGTCCAGCACCCCGTCCCT
**CoV-bait-2876**	GCACGATCAAGGCAACGCCTGATGTTAGCACAGAAGTGCGACAGACGAACCTCTTCACTCTGTAAGAGTTCTTTGAAAGCACCAAATGATTTTGCGGTTAGCACTCCGTACCAGCACGCA
**CoV-bait-2877**	GATGAATGTTGCTTTCTTGAGTGTGTTAAGAATGTGTCTGTTAGCTAACCGTGGTTTGTGGATTGTGCGTCTGATCTTTGAATACTTTCTGTAAAGTACACCTTTCGTTGTTGTGATGTA
**CoV-bait-2878**	TCAGAAAAGTTGCAGTGTGCAGTACAAAATTGTAACTTAGACCACTGCATACCTTGACCTACAGGTGCTGTCATAGCTATAGAAGAGGCATTAACAACTGTATCACCACTAATAATACCA
**CoV-bait-2879**	AAAACAGTGAAATGGTAAATTGTCTAGTGTCAACACAGAAAGAACTAAACCCATTGATAGTAGTGTCAGATGCAATAAGGTTGGCACCACTATGACCACCAAAAGCAGCAGAGACAGTAA
**CoV-bait-2880**	TGTTCACTTGCAATCTCGCAGAGGTGACAGTCTTTAAACTTCTCTCTATAGCTACACACTTTACTAAAATGACAGCTATCAATTAAATGGGTTCACTTACCTTCTTGCACTGTAAGTAAA
**CoV-bait-2881**	CAACATTATAATGCACCAGCTGCGAGTCTGCCAAAACTTAAATGCAGCTTTACAATTATACTTTAGACTACGAGTCAGTATAATACCTGTACGTTGCGGTGCTTGTGCCAATACTTTAAC
**CoV-bait-2882**	GTATTGAATAACCATCAGCACTCTGCAATGGTGTGAAACCAGATGTAGTGGCCCATTCATTAAAGGCTTCAACACTAACACGAGATGTGTTAAGCCACCATGTAATACCGTTAAGCAAGG
**CoV-bait-2883**	TAGAATGGTAGAAGAAACCAGGCAACTCCCTAGTACTGTTAAAAGTGGAGCTAGACAAACTAGAAATAACACCCACTATATCATCATCAACATATGCAGCCTGCTCTGAAAAAGAACATG
**CoV-bait-2884**	TTATGGCGCCCGAGTAACTTAGGAAGTACACCGGTTCACCATTTTCATCATTGCTCTGATGAGTAACCTTAAATTGGGAGGTACTCATGAAAGCAGATGCTGAGGGAGCAAGTTCAGCAA
**CoV-bait-2885**	TAATCCTCCTCTCAGATAAATTCGCGCTTTTCGTGCTATTGCTCCGCGAAAAGGATTATCTTTACTATTATTCATACCAGCGATTGGTTGGGAGTATATGCCAACCTATAAACTAAATCT
**CoV-bait-2886**	AGTTTCAATCCCCTCAGGTACAATTTCAACCATTTCTCCATCTGGCTTAAAACAAAATTTTCCATAACTTACGTATGGACAAGTAGTTACAGTATTAACAGACACAGAGCGTTTTCTCCT
**CoV-bait-2887**	TCATAACTAAAGGTCTCGTAAAACAAACGAGCTTTACCAAACCTATTAAGAGGATAACCAGCACTCTTATCCAAATTAGTGACAACAACTTCTCTAGCAGTGATACAACCACCATCATAA
**CoV-bait-2888**	CACAACATCCATCTCCATGCCTTACAATCTACAATAACGTCCACAACTTTGGACACAACATTAAGATCTAAGCTCTTAATGATAGTCACAAAATCGTCCAAGAGTATGTCCATATAAGTG

**CoV-bait-2889**	AGACACATTGACAACAGTTGGTGACGGATGCCTTTAACCTGGAGTTCTGACCACCTACGGTGAGCCAGACCCCCTTATGCAGCCTCTGACTAAGAGCTCGATTAGTTATCGTCAATTCCG
**CoV-bait-2890**	CTTACCGAATGTTGTGATTGTGTAATCCACACCACTCTCAATGGCTAGCAATTTTTCTTTACGAACTATATTTTTAGGAGTTTCTTCAACCTCACTAAAAACACGAGCCGTGTTTGGTTC
**CoV-bait-2891**	CACAGAAGCCAAATCTAGCAGACTGGGACTTGACACACTCATTTATTTTCTGCATGGCGAGGGATCTCATAGATCTGACCTCGGCATACTTTACAAGCGTCTGAGCGACAAAAGAGTTAA
**CoV-bait-2892**	GGTTGACGCCCGTTCGCGGAACCAGGTAAGATCGACTTCAAAGGGTACTCAAGGAACATGGGGCAAAATGTCGTAGACTCACAACCGACAACTGCGAAGCAATGCAGGTTTTAACTCGCA
**CoV-bait-2893**	AATGCGACGCCTAACAATACTCCATGCTTGACCACGTCGCATTAGCGGTATAAGATGAGCAAATTGTTCACCGGGTGGTGCTCTAGACTTAACAGGACATATGGAACTTCCTTTATCTGT
**CoV-bait-2894**	TTGAAAAAGCCAGGCTCACCTGCATTAGACCGAATGATGTCCCAAAGACCAGTGTGATCTCTAAGACACTTTGGCATTTTATATTTACGACAAAATGGTTCTATAACGATGATGTCTTTG
**CoV-bait-2895**	CTGGACTACCAGTACATATATTTTTGTAGAGACCAAATACTTGACCATTCGCACACAGAGGGAATGATATAGGTGGTCTATGCTCTCTACAATAATAAGACATGCCGCCCAAATATAATT
**CoV-bait-2896**	CAGTCTACTATTTATAACACTGAGATCGTAATTTGTGCACATTGACACTTCATCCACAACCACAATGTCACAGTTAGCTTCAGGCAAAGCATTAACTGTGCAAAATAAATATTGGGCATT
**CoV-bait-2897**	GCACAGCAATGCTGAACTCTAAATGGAAACTTTTAATACTAGCACTAACAACATGATGTTGTTGTACTATAGAATTGACATGTGGTGTGTTGTTAACAATCACATGTTGATTAGTAACTA
**CoV-bait-2898**	CAATTTTGGTACAGAATGATTCAAATAAACAAGAGGCGCTTTAGTAAAATTAACAGAACAAGTATTCATAAAAACAACATTTTTATCTGAAATTGGTTCAGGATAATAGTAAGAACTACC
**CoV-bait-2899**	ATCGTCTAATTTAATTTCGTTTACAATGTCTTGAGAAAACGCTGCATTATAAACACCAACAACAGGCTCTGGTGAAAATATGTAAATAAAATCACCGTTAAGAAGTTCTATAGACCTTAC
**CoV-bait-2900**	CCAGTGCCTTCAATTCCAACTATAGTTGTCTTCTTAACAGTTTTACAAACACCACAGACACCATAGTCAAAGGTAGCACTAGTACAGCGCATTCTAAAAACACAGCCAGAAATCAATTCT
**CoV-bait-2901**	CACGAACATAATATGACCATCCTGTAGAATTTGCAGCCTTAAGTGATTTTCCCACTTGGTGGTAGACAATGGTATTACCAGGTGTGGCCACCATTACATACTTAGGCAAATTTTCCACAT
**CoV-bait-2902**	ACCACCGATAGAACAGCGTCGTTGTGTGGTTCCAGTCGGACAAAGACCTGCTTGCGGTTTATCTACAGTGCAACTATTAACAACAGATGGGCTTGCACAAGGACAATAATCATTAGTGGT
**CoV-bait-2903**	AAATCAGTAAAATTGCAGTGAGCCGTACAAAATTCTGAAGTTGACCATGACATACCATTTTGAGGTGCTGTCATAGCCACAGAAGCAGCACTGAAATTCTTACTCCAATAAATAGCACCG
**CoV-bait-2904**	CCCCCTGGGCTCAAACCTGCAGACTGCAGTGGCCAGTGGGGAAACAGTGGCAGCATGACGCAGACTGATACGGGCAAAGGCTGTGTAAGCAGTGAGTAGGACATCGCTGAGTCCACTAGG
**CoV-bait-2905**	CTAGCTGCTTGTAAAGTTCGTCATTATTGCGAAACACAACGAGTATCCCAATTTTTGCTCTTGTGAGTGCAACATTCAGACGCGCCATGTTTAGTGCATGTCCTGAATCCGTAGTGACAC
**CoV-bait-2906**	TATAACATGATAGATTGCAGAAACAATACCATCATAAGTGCAAGCTATTGTCATCGATGAACCTGGTTTAGCAGTGACAAATTCATAATCTGGTGTATGAGGATTGTTCTGCTCAGTTGG
**CoV-bait-2907**	TAACCCATAAACCCTCTGGTATCACAGAAAAATTATGTCCAGTGCTAAACCCCAATTGTAATGGTAGATTGGTACCTGTATGAGGTTTTATAGCATGGGCTGCTTCAACATCAAATCCTA
**CoV-bait-2908**	ATTAAGATGATAAATACGATGAGTTGCCACTCTGCAGCGTCAGACATGGTGCCAGTCGTTTCGAGGGAACTTATGAAATTTGATATAGTCTTCCTTACTCACTACAGGAGCAGGTTTGAG
**CoV-bait-2909**	TTTATGGAGTAATAACTGTGATTCTGAACAGTCGGCATGGTGACAAACGTAAACTTCCTGGATATTTTTTCTATAGCAGAGGTTGAGTAAAAACCATTAGCAGGCTCAAACTGGTTGACT
**CoV-bait-2910**	ATTAGCACCTCCTGGGGTTCTCTTACCAAAGACTTGACTAATAGTCTTTCCCTTAGTGAGAGTTCTCTTAGGTATTTGGTGCGTTTTGGGGTTTGTTCTGCTCGGTGCTGATTTGGATCG
**CoV-bait-2911**	TACAGCGTGTGATACAGTCTCTTGCTGTCATTACTATAGGCTGTTCTGTTGAACAGTCAGTGTGTGAGTAATAGACAATGGGTGCGAGTCTTAGCTTGTGTGTAGCATAGACAGGCATAG
**CoV-bait-2912**	AAGTCTGGATCTTGAAAAGTTTTCTTCAAAGTAAAATGATATGTCTGACTCGGTGTCATGACATACAAAGCTCTAGGAGCTGTCTGCACAGTTTGTTGTGCCATAAGGAGCTGCCCGTTT

**CoV-bait-2913**	ATTTACGGGAACACCATTCTTCTTGAAAGAAGGTGCTGTGATTTTCTTATTCTGAGCCTTAATAGGCTGGAACCAGGATGCGTCAGCGCGTGGCACACGCGGGACAGCCATTCTGTAAGA
**CoV-bait-2914**	ATCCAACAGTTTGTACTATTGGATGTCCATCAACAGTAGCTTGTGAGCATAGGTTACCAAAACATGTGTTAGGTAAACACATAGCAAACAAAATGTTAAAATTCGCACAGTGCACAATAC
**CoV-bait-2915**	AATATGTACGACCAGAATTATACAAAGGTTTAGAATAAGTAATATAATCTAGGGTCTTGGGTCCAAGAGTTGTAGTATCAAAACCCCTCACAGTGCCAAGATAAACTCTTGTGTAAGAGC
**CoV-bait-2916**	AATAATACCAACAGTACACCCTGATGAAGAGCCTGCATTATTAAATTCGCTAGAAATGTTAACTACCGCATAAGCACCCCCTTGTAAATGCCAACCACTAGGTGGTCTGAAGGCACTTTG
**CoV-bait-2917**	TAATATTCCGCTCACCACCAATACCAATCAATTTGGCAGACAGAAGTAGTGAATCAAGTGTTCCAGTTGGTGCACGTAGGCCGTTAGCAACCATATACTTAAACTCAGCAGCACTAACAG
**CoV-bait-2918**	ATTTGACTGAATTACTCTGTAAATTAACACCAAACATTTGGCGCACAACTTCTGTTGTTGTGAATTCATCAGTCAATGAGCCATATCCAAGTATGGTTTTACCTCCCAGACTTGTTGACA
**CoV-bait-2919**	TACAGTGTGCAGTACAGAATTGCCTGGTAGACCAATTCATACCTGCTCCTACAGGTGCTGTCATAGCAACTGCAGCAGCACTAAAATTTAAACTACCAACAATGGCACCAGCAGTGCACG
**CoV-bait-2920**	TTGTAAACCACCAAATAGTTAGTGTACAACAGTGTCAAAAATACAGGAAGTATAAACATGGTCAAATACAAGTGCTTGTGTTTAATAAACAACATAGCACAGGCCACAAGACAAAGTGTT
**CoV-bait-2921**	CCATATACACACAAGGCGTATCTGAATAATAAAAAAATGGCATAGGCTTTAAATATTCAAAGGCTGCCCTAGAAAAGGGATTGGTGTGGAATGCATGTTTATTAACATACAAACTTCCAC
**CoV-bait-2922**	GCTAGAGTTGTCTCAGGCTCATCGCCTACATCAGTGCTGTCCGTTATAATTGTTGTATCATCATATACAGCAGGATCTTGTGGATCATCAGACTCAGCATCAACAAATGACTCGTCGAAT
**CoV-bait-2923**	CCTTTAATAGTACCCTGACTTCTCATGTTCACACCGTAAACACTACCAGGACAGCCTTCATAACAGGCAAGAATGTTAAAACTCTCACCTGGCCTCACGGATTTAAATTTGTGTTCCGGT
**CoV-bait-2924**	TGCAATCATTGCGATCAAAATGTCGCAACGCATAGCAAAGATCCAACATAGTATACTTTGTTAAATCCTTGCGTACAATGTGTGGCACACGACTACCTTCTACATCAAATGTAAAGAAAT
**CoV-bait-2925**	CAAAAGGTATAGCCACGGCGCCACCACCAAGTGCACCTAATGTTATACCACCTGCAAGTGATGCTGTGTACATAGTCATCTTGTCAGCATTAGCTACACCTGGTAGAACCATGATGCCAT
**CoV-bait-2926**	TTATTATAAGCACCCTTTTCTCCACGTTTACGCAACCACATGGTCCAGGGCACAGTTTTGGCTTGTGCCTCTTTTGATAACTTAGCTAGAGGCACCACCCACCCTACAAAGTTGCCAGCA
**CoV-bait-2927**	CTGCATACAGAGCGTCTTGCTCCTCATAAGAAAGTGCCTCATAGTATAAACCAGCTTTGCCAAATTTATTGAGAGGGTAACCAGCACTCTTATTGAGATTAGTGACAACTACGTCTCTTG
**CoV-bait-2928**	GAAAGCACTTTTTCATAGTCCATGTTGTAAATATAACACTCGTCATCAGCGAATGCGTCTATGTCAATAGGCCGCACGTCAGCTGGACAATCCTCAAACAGTTCGCGCAGTTTTTCAGCA
**CoV-bait-2929**	TGCAGCACTTGAAGTCTCGCGTGGGGCCAATGGTCGGTATCGTTACAGTCCTATTAATGCGCAGTTTCGGATCGACAATCCGAGATAAGCCCATCGTAAGACCTCGTTGAAGGAGTAACG
**CoV-bait-2930**	TATATGCGTAGCCTTGTTCATGGTCTAAATAAAGAGCCCTAAGGTTATCGAGTTGGACACCACCGTCGGTACCATTCGACACACAATCATACCAGGGTTCTACTGTGGGAAAGTAATCCC
**CoV-bait-2931**	AACGTCCTTAGATGTTGACAAATGACGTAGAGCATAAGCCAAGTCCAGGAGTGTATATTTAGTCAGATACTGGCGATTGACATTAGGTATCTTGTCAAACTTGAAGAACTCAGTTTTGGC
**CoV-bait-2932**	GAATTCCACGTCCGATAACGCACAACGTCACCAGTATTTCTTTAAGAACTTCACAGTTCTTTTCATCAAAATTTCTGATAGCATAACACAGATCCATCATTGTGTACTTTGTAAGATCCT
**CoV-bait-2933**	GAACATTAGTAATTCCCATAGACACAGAATCAAAATATTGAATTTTCTTAATACCACCACCATTAGACTTATCAGTACCTAACAAATTGATCCAACCACATTGTGCTGGACCCCCACAGT
**CoV-bait-2934**	TAACAACCTTACCACCCTTGAGTGAGATTTTAGTAGTTATGACATTGACAACTTGCCTAGTAGTAGCACAAGTGAGCCTAAAGGGTATGTTGTTCTTTTTAGCAGCACTACGAATTTGCT
**CoV-bait-2935**	GTGGACCTAAACATCCAACTAGGGATTTAAAATTAATATTATCAACATCAAAATGCAAATTAGTATTAAGATTGGAGCTAAGTGTGACACCTTGCATAAGAGTATCAGCTACATGCAATT
**CoV-bait-2936**	TAAGAAGCCATACATCATTACTACCATTAGGAAAACACTTAAATCTATGATAAGGATTAAAATTAACACCCTCATAAAAAATAATTTGATCACCTTCGCCAGTGTAATTATAAAATCGAG

**CoV-bait-2937**	TACCAAGATACAAACTAAATAAAAAAGTAGTAGGCATGCCAGAATCAGCATAATAAGCATAAAAAGTGCCACGTTCTTGATAAAAATGAAAATACAACCAATCTGTAGAAACATTATAAG
**CoV-bait-2938**	CTTCTTTATCAGACCCTGCACCTAAATGTAAAACACGCATATTAACAGGAACAGCTAATGTTGTAGTATTCAAATACTGACATAATTGAGTGTACTTAGCAACATTCATCATACAGCCTG
**CoV-bait-2939**	TGCGAAAATAACAATATGGCAATTGACATGCAACAGATAAAGAAATATCTGAGGCACGCTCGTTGTTCCATCTAGAATCAACAACCTGTACAGGTACAAATTGTTTAGATTTATCAAAAC
**CoV-bait-2940**	GCGATTAGGTATAGCAAACTTGTCAACAGTAATACTATTAAAGCAACTACCAAAAATTTTAGATTTATCAAGATTATTACAAGAAAAAGAATCAACATGAACTAGACGAAGTAAAGTGCT
**CoV-bait-2941**	GTACTCTCCTCGCTGCTGATACAAGGTGTACTTCCTTGAAGGTGCACGGCGTGTGTATACTCGACATGATTGAATCGTGGGCATAGGGGAACTGATAAACTATCTACGCTCCGCTAGACT
**CoV-bait-2942**	AGAGTTTAGGTCCCTGGACTGTGTTCTACTCCAAAACCATTATCCCTAGAGTGTGAAAAAGAATTGTGTCGACTACAAGTCTGCGACTTATTGTATTGGGCGCGAGCAACAAGGGGGGCA
**CoV-bait-2943**	CACTGGTGTGCATACGCCGGTCATTAAAAGTAAGCATAAACGTATTACCTTTTACCTTAGTAGTACGCACAAGATACTTTCTAGTCTCTTCTGACAAGGCAAGAAAATCCTTAGCTAGCC
**CoV-bait-2944**	CTTCCCAGAGATAATCACTCGCTTTCACCGGCTACGGAGTGCAAGTCTTTGGGCTGCCTGCAGTTTGCTGATGCGTTACCTGTCGCAGGAATACTTCCTTCTTGAACTCTCGCACCACCT
**CoV-bait-2945**	TGCCGTATACACTATGCGAGCAGATGGGTAATAGAGAGCAAGTCCGATGGCAAAATGACTCTTACCAGTACCAGGTGGTCCTTGGAGTGTAGAGTACTTTTGCATGCCGACCTTTTGATA
**CoV-bait-2946**	ACAGACCAACATAGTTGAAGGCCTTTTTCCATATGTCTAAGCACTAATTGAGTAAGACACCAGCCTTGCGCTCTAGTCTTACCTGTGCCCTGGATAGAAAATTTGTACCTACTCCCTGTA
**CoV-bait-2947**	AACACGCTAAAAGCGAAATTGCATGCGACTATAGGTAGCATAAGGGAAAAACACAAAATGTGACAAAACCTCATGTTCGTTCAAGAGTGATTCGTAAGGGAAAAATTAAAAACCTTAAGA
**CoV-bait-2948**	TAAATCTGAGTTTGACCGTGATGCTGCCATGCAACGCAAGTTGGAAAAGATGGCAGATCAGGCTATGACCCAAATGTACAAACAGGCAAGATCTGAGGACAAGAGGGCAAAAGTAACTAG
**CoV-bait-2949**	AGAAAAGCTTCTGAAGAAGAGGTGTTAACACTAGTACAAAACAATGTCCAAAACTCAAAACGTTGGATTAATTCATAAAGTCTTTTGTTCCAAGAATACTCAGTGATTTTGATGGCTACC
**CoV-bait-2950**	ACATTGGACTTGAGTGGTACATAATTAGGTTGTCCATCAATAAACTCACAGTCACTTTCATCATAATAGAAGAATGGCATAGGTTTAAGCTTAGCAAACGCTCTTCTATCATAAGCTGGT
**CoV-bait-2951**	CACCTTCTGCAGATTGCACACACTGGCCAACGCGACAGTAGGTCATAGCCTTTGTACGGATAGTACGGATGCCACAGCCGTGTGAGATAATTTCAGGTAAAGACACAGCATTACCATCTA
**CoV-bait-2952**	AAACCGCTAGGACCAGCATTTCCTTTTTTCATAGCAGAAATACGAATATGGTGCTGTTGAATTAAACCTGTTAAAGGGCATACCCCTATACCACTTTTATAACAATGAGTAACAAATACG
**CoV-bait-2953**	TACAATATCACTAAAATTACAATGAGCAGTGCAAAACTGTGAGACTGACCATTGCATACCATCTGCAGGTGCTGTCATAGCCACTGAAGACGCACTAAAATTCTTACTCCAGTAAATAGC
**CoV-bait-2954**	TGCATACCATCTAATACAACACGCAATTTTTCAATTTGTGCCTCAAGCTGTTTTACATCTGCCACATCATCAGACTGCCACATAACTGTTGGGACTTCACCAAGTTCTTGTTCAAGGAAC
**CoV-bait-2955**	CACTAAAATTACAATGCGCTGTACAAAATTGATGTCCTGACCAGGCCATACCTGAAGGTGGTGCGGTAATAGCTACAGAAGAAGCATTAAAATCAAGACCACCTTTAATAACACCAACTG
**CoV-bait-2956**	TTAGACAGGGGATATGCATCAATACACAATGACACATAACGTTCAAGCATGACAACAGAATCAGTTTTGGCTATGTCATCAACAAACACACCAGCAGACAGAATACGACTTGGATCAGGA
**CoV-bait-2957**	CTGAAAAGTTACAGTATGCAGTACAAAACTGACTGCTAGACCAAGCCATACCTGATGACGGTGCCGTCATAGCTATAGAAGAAGCATTAACAACACGACCACCATGAATAATACCAACAG

**CoV-bait-2958**	TCAAAGTTCGTCTACAATCCTAGCAACGCGACCCTCAGGTTTGGAAACATAACGTGTCTCACTAACCAGGGGTTGCACAACCAGGCTGCAATCTGGTGTTGTAGACAGAATAAACAATCT
**CoV-bait-2959**	GTCTTCTGTCACATACACCTGGTATATGGTTTGAGGCGGCACGCACCCCTAGAGAGCCAAGACTTCTGTGATGCGCATGATCAGTGCACACAACACTGCGCCTTTAGATGGTATACCTAC
**CoV-bait-2960**	CAGCTCAAGCCCAAACTCAAGGGTCGGGCGGAGGAGGTTCAGAGGACTTTAGCCCCGCTACAGGACACAAAATCTTGACGAAGAAAGCCCAGGCGTAGCTATATAGGGGGACAGACTGAG
**CoV-bait-2961**	ACCTGGTAATAACCGGAGAACGGGATCGTGAGGTCGTGGCCCATAGGCCCAATGCATGTGGACGCGCACGCCAATGCGTTTCTATGTCTCATTAGTCTATTGATAATATAGCCAAAGGGT
**CoV-bait-2962**	AAACGACAGGCAAGCAAAAATGGTCAGGACTATTGCTGCAGCAAAACCGCCCGTGTCTACTGGATATATGCAGGATATGAGACCACCAGCGATGATAAGTGGCCAAAACTGCCACAACAC
**CoV-bait-2963**	CGAAAAAGTTTGCTTTAAATCTCTAAGGTACAAACAATCGGTCAAAAAACCCTTAATGTCTGTGACCTTTTTAACATTACATGCATCATAAAGTTGATGGGGGGAACCACACTTAACATG
**CoV-bait-2964**	CAGAGACTTCCTCCACATGTTCCAAGTTACAATCATTGACAATAGCAGCTACCTGTTCATCTGACAGACTGTGGTCAGCAATACAGTCTGAATTACAGGCATTGACACCGTCAGCAACCA
**CoV-bait-2965**	AGGCTTTAAATGACCTGATCTCAGATTTATGATCTGCGCCCTCAACTCCTTCCATGGGTACTATTAAAACGGCTTGTGGTGGGTTAATAATAAACTGACAAGGATTTCGTGAGCAAGGCC
**CoV-bait-2966**	GAGACTACTTCCGACCGCGCGGGCATGAACGGACTAGGAGGCACAATAGGTAATGCTTCCGCGCAAGCAGCTGACACACCTGAACCGAACGTGTACCTATCCCGAAGGTACCCGACTATG
**CoV-bait-2967**	GACGCTCATAAGAACGTGCATCAATAACAAAGGTACCAACAGATGCAGCATCAAATGTGCCAACAAATTTAGTACCCTCAAACAAAGTTGTAGAGACCTTAAGTTTAAACAAATTAGGGA
**CoV-bait-2968**	CTCCGCCATTGATCTGGTCGAACGAATGCTGTGTCAGCGACTGGAGGGGTACCCTCCGCAGGGCGGGCATAGCTATAACGAGTCGGCAATGGATTCTAAGCTCAAAAAGCATCAAGAAGA
**CoV-bait-2969**	AAAGACTTGTTGTCAATATTGACACACATAAGCAATTTTTCTTGCTTAGCGCGTGCCAACATTTGTTTAACATTGTATGTCACTGTCGGACACAACAATTGAACATCATTGTCAACACTA
**CoV-bait-2970**	TGTTATTGTTAACACAGTGTAGTGACTTAAAAAATCTATATTCAGTTGAATTAAATGTAATGTTATGAATAGAAACACCACTTTTAAATACACCAATGCGAATAGCACCTTGGCCTATAT
**CoV-bait-2971**	GGTAACAAATGTTTTGGGTAAATTCTTTTTAACAAGCGAGCTAGCAGAATAGAAACCATCTGGTAATTCATGCTTCAACTGTTGGCACTTGATTCTATTGATGGGTGAGTCACAATAGAA
**CoV-bait-2972**	TCGTTTTCACCTACTACTGCCGAAGTGCTAGGGGTATAGTCTATTGACTCTACACTTGGTTCTTGTGCTTCAGCTGTTTCAACTACACACACCTCATCTGAGACGCTTTCCCCTACTTCA
**CoV-bait-2973**	GTTATTGTCAGTTGAATCTGTGGGTCCACCAAATGTAATGCGGGGGGCACTACGTTGGTTTGATTGGGGTCCATTATCAGACATTTTAATTTGTTCGTTTATTTAAAACAACAAGTACGT
**CoV-bait-2974**	TGAGAGCATGCAGTAAACACAATACGTGCGTTTGGAAAATACAAGCCCAAACCTATTACACAATGAGATTTACCACTACCAGGTGGGCCCTGTATAGTCGTAATTTTTTGCTTACCTATT
**CoV-bait-2975**	TAGTGGCATATTACACCCACCAGGAATTTCTCTCAATGAAAAACACAGAGTTCCAAACTTTTGAAAGTTATTCAACTTATCAAATGAAATGGAGCATGTGCCTGATTCTAGAAAAATTCG
**CoV-bait-2976**	CTGTAACAAGTGCTTCTTTAGAAACATCTGAAGGTGGCTCCACTAACTCTACAGGACATAATGTGCCATTGACAGCAACCATAGGTTTGTTAAAAGCATAGTATGTAAAATTCATACAAG
**CoV-bait-2977**	CATTGAAGCGATTAACATTAACAGAATGCGCTGTTTCCGCAGTCTGTGAATAAATGACATAATCATATTCAGACCCTTGCGCTGAATCAACGGTTTGAGTCTGAAGACCCAAAACGCGTT
**CoV-bait-2978**	CTGCAAGCAATTAAGCAAAACAACATTAGCACATTTAACATCAGTCAATTTTGATTGGATTTGAGATACTTCTATGATTGGCACACCACCAATACCCAAAAGCTTAAAATTGAGCATAAG
**CoV-bait-2979**	CACACTATACCATCTTGGCTATCACCCCAATTCAGGTCAGCGGCTGGTCCTGTTCCAGTGTAATAAAAATACCACGCATCTGGGACTGGTTTTCTTCCACTTTTGCCCGGCTTATACCTG

**CoV-bait-2980**	ATCATCATCATTCTCGGGAAGATGGCCTTCTTCTAGCTCATTACGCACCACTTCTAAACAACAATAACCCATAATCATAGCTAGATTATCAGTGGGTGGATTATGTTATTCCCACCCAGT
**CoV-bait-2981**	TGTTAGACCTTTGACTGAACCTTCTGTTATTAAAACACCATTACGGGCGTTTCTAAAAAGGTCTACCTGTCCTTCCACTCTACCATCAAACAAGACAGTAAGTGAAGAACAAGCACTCTC
**CoV-bait-2982**	TGTTGCTATCGCATTGAAGTCAGTGACATTGTCACTGCCTACACATGTGTTTTTGTATAAACCAAAAACCTGACCATTAGCACATAATGGAAAACTAATGGGAGGCTTATGTGACTTGCA
**CoV-bait-2983**	CTACTTGTTTGCCATGAGACTCACAAGGACTATCAGAATAGTAAAAGAAAGGCAATTGCTTTAAATTAGTAAATGCACTTTTATCGAAAGCTGGAGTGTGGAATGCATGCTTATTCACAT
**CoV-bait-2984**	AGAAGGCACAGTCGAGGCGGATCGATCCGAGCTCTCCCTTAGCCATCCGAGTGGACGACGTCCTCCTTCGGATGCCCAGGTCGGACCGCGAGGAGGTGGAGATGCCATGCCGACCCTTTT
**CoV-bait-2985**	TCTTAATAAGGTCAGTGGATAATTTTGGTCCACAAACCGTGGCCGGTGCATTTAAAAGTTCAAAAGAAAGTACTACAACTCTGTAAGGTTGGTAGCCAATGCCAGTAGTGGTGTAAAAAC
**CoV-bait-2986**	TTTAACAGCCAAAGCGACAAGCTTACACGTAAGCTTTGAAGCAATCTCTGTGGTGTATACAACTCTATTATAACTGGATATTTTTATCCACCCAAACAAAAGAACAAAAAGCCATTTAAT
**CoV-bait-2987**	AGCCTTCGTCATTTCATGACCAGCGTTTATGCACTGGTTAAGTGTTTCCATGAGTTTCATTCTGAACATCCTTTAATCATTGCTTTGCGTTTTTTTATTAAATCTTGCAATTTACTGCAA
**CoV-bait-2988**	ACACTAACACACACTTTACTTTTACTTATACTAACAATACTCAACATACCTATCAACTACATACAAAATCAATTTCACCAAAACTTTTCATCAAACAAAAAAAAATTCAACAAAAACTCT
**CoV-bait-2989**	AAGTTTGCTGCACTTTCATCTCTGAGATTCTTTACGTCACCTCGGAGTTCACGAATATCATTTGCGTTAGCAACTGCCAATCCAATAGCAACTGTAGCTTCAACAAGTGCAACTGACGCT
**CoV-bait-2990**	AAAAACATACTCACCTAACACGGTCTTACCATTCTTAGTAAAATGGTAGCCGGTGAAAACATAATTTTTATTAAGTGGTGGCCGCACTTTGCCAACCTCCCAAGAGAGAATTAATTCACG
**CoV-bait-2991**	GGAGGTTTTTACTTTAAACCAGGCGGTACGACATCTGGTGACGCTACAACAGCTTATGCTAATTCTGTTTTTAACATCTTCCAAGCTGTTAGTGCTAACATTAACAGACTGTTGAGTGTT
**CoV-bait-2992**	AGCTGCTTGTGCCAAAGATCATCAACACCCAAGATGGCATGTTGTTTTATATCTGCACACTTATATTGAACATAGTCCAGATACAAAGCATGACAAATAGCAGCAGTCTCTTGAACAATG
**CoV-bait-2993**	TAAGACATAACAGTAAGACTCATACGATCAAACATAACCACAAAATCTGTCGAGGTAACCCTACAAAGTAAAGCCTCATAATCAGGATCAACCATATCATTGGCTGCACAGATGACATGG
**CoV-bait-2994**	AATCCAACTTTGAGTGCACTTAAAATCGCCAATAATACCAAAAACAGGCTCGGCGAATAAGAGCAGAAAAATTATAACCATGTTTAGATTAATGGCCTACGCATCATGAAGCCTAGTACC
**CoV-bait-2995**	CTTTGGGAGGCGCACCACCTTTGAGTCTGAACAAAGTAGGCACGATCTTTCCATCTGTGCAGAGTGGGTAGAAGACTGTTTCGCTGTCTTCGACACTGCGCACAAAGATTTTATTACTAA
**CoV-bait-2996**	ATAACTTCCTGAATGGCAATATCAACCGAATTAATATCACTAGGCTTAATATCAAGCATAGTGACACTAATGTGCGGACAATTTTGTAGCTTGCATTCTACACCTCTTTCAAGTATTGTA
**CoV-bait-2997**	TACTAGAAGTAGTACCAGAAAGTAGTGCCACTACAGTCTCTGTGAGCACAAACTCATTAACAAAATTGATGGCATGCTGAGCCCTTTCGAGGAAAAGGTTTGCTGTTTCGCATACCTTAC
**CoV-bait-2998**	TAACTGGTTTATTCTTTTGCAATTTAACAAGCAATGTATCAATGTTAGGAATATCCCAACACTGTGGTATAACACCACCATCATCATCGCCTGTATCACATTGATAATCATCATCTTCAA
**CoV-bait-2999**	TGGTTGCAGGAAGCTGTTCTTCCTCTCCATTCTCAGTGCTATCTGTAATGATGGTGTCATCATCACATACAGCTTCTTCATAAATTACATCAGAATCTACAGACTTTAATTCTGAGTCTG
**CoV-bait-3000**	CGTTTGCGGATTGATCAGTGCAGATAAAGCACGTGAATCCAAAGGATTTTGCATTACGAATGATGCCACGCCAACCCTCGTGTGAATAAGTTAATCCAGTCGGAGGTTTTGGTGCAGCAA
**CoV-bait-3001**	GAATGCAGTCTTCAGTCGCTCTTGCCACAGCAATAGCGTCACCAAAGACTAGTGTAACATCATTGTTCAAGACAATGTGTTTGAAATTTTTAAGAGGATTCTCTGCCTTGGGCTTTTTAA
**CoV-bait-3002**	ACCTCACTAGTCGGAATAGGAGTGCCACACAAGGGGAAGGTCGGAACAACCCACAAGACTGTTAGTGCAATACCTATCGGGCGGAACCGATACAAGCGTAAACACTAAAAGTACACACCG
**CoV-bait-3003**	TTTCAGATCACGGAATGCACTAGCATCATATGCAGGTGTGTGGAAGGCATGCTTGTTAACGTACAAGCTGCCACCATCACAACCGGGCAAATTGAATTCTGAGTGCACACGTGTGTCAAA

**CoV-bait-3004**	AATTACAGTAAGCCGTACACAACTGTTGTGCGCTCCAAGTCATGCCAACAGAAGGTGCTGTCATAGATATAGCGCTCTTATTAAAACTATGACCGTAATTAATAGAACCTAAAACACATT
**CoV-bait-3005**	CATATTGTTGTGGTTTTGTGTAGTTAGTCTGTGCTAGCATTGGTGGTGCTGACAGAGGTGTTACTACATGAGACATTAATACAAGAGCATCACCAACCTGTAGTTTGTAGGTTGAAGTGG
**CoV-bait-3006**	GGACTGGGTTGTATATATGATTGACAAGTTGGTAATCAAACTTGTTACGTACTAAAGAAATTAGAGTATGTGTTCCTCCAATAATAGGAGTGGCATCATCTCCATAAATGATATGTTCAA
**CoV-bait-3007**	ATTCCGTGTTGTACTAGAGATCTGTAACCTTGATTCTTTCTCATCTCGAGCAGGCCGAACACAAGGTAATCAGATGTCAGAAGGGTCCCAATAATAATCGGGACGTGCAATAGCTTTGGC
**CoV-bait-3008**	TRGTAGTGTTATACAAAACAACAGATCGATTTTCCAGATCATTAATCTCATTAGTAATATTAAGATAGGTTTGATTGAATATGTCTAATGATAACTCTGGTACAGTCCAGTTAGGTTTAT
**CoV-bait-3009**	GTTCTGGGAGTCAAATAGAATTTGTCATCTAGATTGCGAAACAGCGTCAACTGGACATCCTTAACAACAAGTCCAAAAGTACGATCGCCATCTGATGCACAAATACCTGACCAGGCTGTC
**CoV-bait-3010**	CATCACTAGAACCCTTTACTCGTTTAAATAACTATTGTTCTGGTTTGAACTATAACCCAATTGAAGGTTTGTACACGTACAATCATGATTTACCCACCTGTGACAAGCAGTGCAAGGTTC
**CoV-bait-3011**	ATTCTGCTGCTCATATGGAACATCAGCACGCTGAACGTTCCAAGCATGAACATAAGTAATGCCACAAAGAGTGATCTCATCACAATCCTCACCGAAGTGATCAGTGTAAGACCACATACT
**CoV-bait-3012**	CGATCGGTGTTGATAGTTTCAGTGATACCCTTATGGGGTTTCATGTAATCCCAATTGTGTTTTTCATTTGCTTTTGGTACGCCGAATTCATATTTACTTCTAACTTTTGTGGTGAGTGGT
**CoV-bait-3013**	TTATTAATGTCAAATCCGCCACAAGTGTCATAAATGAAGTAACCTTCAAGTTCTACACCCTGTTCAGCAAGCGTGTCGAAAACCTTGTCAAGTTTCTCAGAAATAGAATCTTCAAATTCT
**CoV-bait-3014**	TGCTGAGAACAGAATTCATGAGGGCCAACAGTCATGTCAGTTTCAGTCCAACATTTTGAATCTGCCATATAAACATTGTTTTGGTAGAACAAAATGTCACGAAACCCGTTTAAGTCTGAA
**CoV-bait-3015**	AAACAGGCCAAAACAGCCTCCACAACACCCAAAACAGCCTCCGCAACATCCTGTGCACAAAAAGATAGTGACAAGTATTGTTGCAAATGCTGCTATTGCTAAGAAAATGAGAAGCCATAT
**CoV-bait-3016**	ATGCACCAGGAGTTAAACGTACAAACTCATGTGCAGGTGTTTTTGTATTCGATGTGTGAACGGTCAATTGTAAGAGAGCGCCCACCATCTTAATAGAGGTTACATTAAGCTGAACGCCTT
**CoV-bait-3017**	GCAACTAAGTAACGTAGAATAGGTTTCTTCTCTAATTCTTTAAGAGATATTATCGGGGTTGCCTTCAGCTTACAAACTGTTGAAGGATTGTCGGCTAAAGGTGAATAAGTTGGTGTCAAT
**CoV-bait-3018**	ATAGAACCTCAGATTGATCGACATCTTGAACAATTTTAGGTTGTTCAACACTTGGCAATTCTTCAGTAGGTTTAACATCAATGGTCAAATCCTGCTGAGTCTTAAATTTGTTAAGGACTT
**CoV-bait-3019**	TCTTGTCAATGAGTGTGTTGACTAGTGTTGTTAAGGGCTTAAAGAGGTCCATAAGACGCTCTAAAAAGCTCAAAACATGGTAGATTGAATCTGAGACCCGTATGCGGAGTTTTCGAAACT
**CoV-bait-3020**	GCCACCAGGTAGAACCATTAATGAGTGCTGCATAAAGAAATGCCAACACATTCTCTGTAAACAGACTACTAGCGCCTTCAACTTGCAAAGTAGGTTGGTCCTCATAACCACCATACATAA
**CoV-bait-3021**	TAACTGTTAAGTTATAAAGAAAAGAACCATTTCTCATAGCAGAAATGCGAATATGATTCTGTGGTACAAAACCTGTTATAGGGCAAGCACCCGTAGACGCATTATAACAGTGCGTAACAA
**CoV-bait-3022**	GAAAGTAATGTTGTTAAGTTTACCATAAGTTGTAGTACTAATAACATCATAAACAACAACACTATCATTAACTACATTATTACTTACGAAAGGGTATAACCCATCAGTAAAATTTCCTGT
**CoV-bait-3023**	CAGGTTTAAAAATGTAGAGTTGCCACTTAGTAGGATCAAAAACCTTCTGTGACACTCCAATAACAAAACCACCATTTCCAGAACCAATATTAACAAAGAAGCCATGAGTATTATTCTGCG
**CoV-bait-3024**	CCTTTGCCAAAGTGAAAGACACAGTTGCGTCCTTGACATAGACAAACGAGTGTCCCAAAAACTTAAATGGCTCACCCTTCTTCAGCGCAGCCAACACATCAGCCACAGTAGATGACGTAT
**CoV-bait-3025**	GACTTTACGGGATACGCCACAGTTGATCAGATCAACTAGCTAACCAGATGAACGCATTGACCCCATTATACCCTGAGGGGCCGGAAAAGGTTTATCGTGCTTTTGCATAAGAGACCACAT
**CoV-bait-3026**	CCAGAATTCTCGTCTTAGCCTCACGCTGAAGATACTCAAATGACACCTTAGGATCAACTCCAAAAATGCCTGCAGATACAAGTGGTGTCACAATTAAAGGGTATGAATTCATAGCCTTGT
**CoV-bait-3027**	CTGGGTTAACTAGGACTGAATTGTCAAACTTTATAGTTGACAAATCACCAACAACCAGCTCTACCATAGTAGGGCTTGGTTGTTTAATAGCATGTGTTGGGATGTTGTTGTCAATAGTAA

**CoV-bait-3028**	TAGGCTGGAGTGCTAGGGTTAGCAACATCGACAGTCAACTTCAAAAGAGTGCCTTGCATGGCATGACCAACAACACGCAAGTTTGCTGGAGCGCCAATGTGTTTTTGCACACTGAAACTA
**CoV-bait-3029**	AAATAAAGACGCCGAGAAAGCCATAGTTCGTTAAAATCCTGGATGATGTAAAATGGGGTAAGAATGAACAATTTGTTTATTACCCTGATTGGAAGACCGTATGGAGACAACATCTACGGC
**CoV-bait-3030**	CAGAATACTTTGAAATGCAAACTTGAGCTTGACCACTAATGGTAGCTCGAGCAACAACAACATCAGTTTGACACTCTACATCACGAGCCAAGACATAGGCAGGTGCAGTAGGTTTTACAT
**CoV-bait-3031**	GAGAGAAGCATTAGTAAAGAAAACCAATTTATCAACACCAGCAGAGCTATAATGCCGCGGGTCACTCGAGAAAAAGGCGAGATCACCCTCAGTACGAAACGGTTTTGGGGTTTCGCTAGA
**CoV-bait-3032**	ATGCTTAAGGAACATCAATACGCGTGTAACGATGAAAAAGACGACGATCTCGTCACCAAAGACATCAAAAGGCACAAGCTGCAAAAACCAAATGGACTGTTGTAGGCCCAAAAACACACC
**CoV-bait-3033**	TAATCTCGTCAGCATCAAAATCATAGTATGGGAATCTGAGATTTATTTTCAGACCTGTTGTAAGTGGGGGGGTCGTGCACCGCTTACATTTTCAAGGCTACAGGGAACGGCGTTCAGACG
**CoV-bait-3034**	ACAAGTAATTTTCCAGAATTAAATAAAACTTTGAGTGTTACAGCAAATACAATGGAATTTAATTCGTCGAGTACAGCGTCAACGGAGATGCCAATGGACTTGACGGTGTCCATAAATCGT
**CoV-bait-3035**	ACATCCAAAGCACACTGATAAAAGGAAAGTTTGCCAACTTTGTAGAATGGTAGTATAAGATCATCGTTTTTAGGACTTTGAATCTCTTTTTCAATAGAAGATTTGTTATCTTCCACTATA
**CoV-bait-3036**	TCAGTAAAATTACAATGGGCTGTACAAAATTGTTTAGTAGACCATTGCATACCTTGTGAGGGTGCTGTCATAGCTACTGAAGATGCAGTAAAATTCTTACTCCAATAGATAGCGCCAGCT
**CoV-bait-3037**	ATGTTGTCCATTTGTCACAACGCATACAAAAGACTTCTGGTTCCAAAGGTCCAGATTGATTGGTATTGGCAATAACACCATCATGCGTAAAATAGGTATTGCAACAATTAGCAGTACCAG
**CoV-bait-3038**	CCCAGAGTAAATGACGAAGACAGACTCCCACAATGTAGGCAAACACCATCCATATAGGGACGACTGTTCCGAACATGACAAACCATGAGACGTGCATAACGAATGCAGGCTCACCAGTAA
**CoV-bait-3039**	ACGGATGATAAGGGTGAGATTGTCTCGTATAAGTTATGTGAGTCTTGTGCTGGTTTTCCCCAACACGTTTTTGCTGTGTCGGAAGGTGGTAAAATACCTCCAAACTTTGATTTTACTAAT
**CoV-bait-3040**	AAGACTATCACCAACAAAAATTTCTTTACGCGTATCACGCACTAATAACTTACCTCTTTCAATAAGAGAATAAACTAAATCATTTAATTGATCTGGTCTTAAATTTACTACAGCAGTGCC
**CoV-bait-3041**	TTAGAGTCAACTGAATAATAGTATTAAGGACTCTACCAACAGAAACACGTGTGGTTTGAGCAAGTCCTGTAATATATGCCTGATTGGCAGATTGACATGGATAACTAGCAAAAGAATTGT
**CoV-bait-3042**	TTGTCTCTAAATTTAAACTCTTTCTTATTTAAACACCATGCAAAAACTTTAACAATTGCACCTTGTTCAGGTTTTGCACCTAACCAACGAACACGACCAAATTTTTCATGAATATAATTA
**CoV-bait-3043**	TCCGGCGTAAACACGCGGAGCTAGTCCACGTGTGCATTCATGCGACCTCTGACCGTACAGGTGTTCGTTGTCGCCTCGGACAGCTAAACGATCAATCGCTCTATAGTACGGCGGATAATC
**CoV-bait-3044**	ACTTGTTGTCAATGGCAGTAGACCTGTAGATGTTATGGTACAGGCGTCGCTGCAATTTCTGCATGTATATGTTAGAGATCTTATTGCCATCTGCTGACATAAGGGCACTAACATTAGCAG
**CoV-bait-3045**	ACTGGGTTGCTTGTGTAGACAAACCATATTATAAGTAAAGCAGTAAATACCACTTTACGTAGCACACTCTTGTAGCTAGACATTAAACTCTTAGCACTAGCCTTCAGAAGACAGAAGAAG
**CoV-bait-3046**	CACTACTAAACCGATTAGTAGCCACACATACCAAGGCCATTTTACATAAGTTTCAATTCTGTTGAGCCATTCAAGGTCAACAAGAGTCTTATTAAGATTGTCAATGTACTGCTGTAGTTC
**CoV-bait-3047**	CACCTCTCCCAGTACCATTAAAATAGACAAAACCAGTAGTAAACCGCAAGCCAGAAACAGACCATAAGCAATTAAGCAACAAAGGCTGAAAACTCCTCACAACACCATCTACAACAGATG
**CoV-bait-3048**	CAACATCAAAACTGTTGTTGACAGTAGCACCATTACATTGGCTAGGGATTTCGTTGAAACCCACGGTTAGAGCCTCCTCACCAAAACTAGGCACAGGTGTTAAACAATTAACAAGTAGAG
**CoV-bait-3049**	TCGCATAAGTCATAGCACTATAATTATAAATAGAGCGATAGTAAAACGACGGCGTTTCGATGTAATGTGGTAGGTTTAACATGTCGGATTCAACAGACGTAATAGCACCTACTATCTTAC

**CoV-bait-3050**	GTGTCTGAATATATTCATCTGTAACAGTAAGATTAAAACTATCAGGTATTAAAACAGATTCAGTAAAATTAAGTAGGGGAGTAACTACACCAGCAAATATTCCAGGAACTATTAAATGTA
**CoV-bait-3051**	CACTGGAAAGTAGGTACGCATAAGGTGACTTAGTTGTTCCTGTTGCTACAATCTCATCCAATATTAACTGAAAAGATTCCTGGATGTGGCTTAGGGTTCAACAGTATTAGTACAAGGCAC
**CoV-bait-3052**	TGTTAGCAAAATTGTCCTTGGAGGCAAACCAATCAGCTTTGCGGTCAACAATCAGATTGCGGACAGCGCGTAAGATCGGTTGAGCACCAAAGAACTGCTCAAAAGCCGTGAGTGATGTCA
**CoV-bait-3053**	TAACACCTTCTAAGTTTGAACCTACATGACAACCACTACCAAGTTCTAATTGGTGCAAGTAACAAAATTCAACTTTGCCATTAGTTACATTGTAACCAGGCGAACCACAAGCACCAGTAA
**CoV-bait-3054**	TACGACAGCTCATGATAAGGCGAATACGGGAACGTAATCAACCCGCGCGCGAGCGGCGCCAATTGTTAAATATCGGATTCTTTGCATTCCTCGAAGTACTCAGTTCCTTAAGATGGTAAA
**CoV-bait-3055**	TTAATATAAGTAAAGAAACCATCTTTCGACGTGTTTTCACCGTCAATTGATTTTGTGGAGCCATCATATAAATCAGAAATAAGCAAATCAAATTTATCTTCAATGTAAAGACTAGTACAA
**CoV-bait-3056**	CTAGGTAAGTGTCATAATCGGCACCAGCCAAGTCTCTTAAAAGAACATATTTGTCATTATCTATAACAAAAGTGGATTTTGCAGCCTCTAAAAATGATGAAAATTTACCACCAGTTTTAG
**CoV-bait-3057**	ACTGACGGCCGGTGCAGAGCGGTAGCAATTAACCCTACGGTGGTCACTGGTGTTTACAGAGTTGGTGTATAGGCAGCGGAACTAAGAGTAGAAGTTTTGTGCAAGTCCAGAGACTGACCG
**CoV-bait-3058**	TAGAGAATTATACAGGGCCGTGCTAGCCGTTCGGGTGATAGGAGTGTGGACGCCACGGCCCTACGGCTGTTAAGTTGTGCTAGATACCTTCTCGTTCTATGAAGCTGTTAAATCCTTCCG
**CoV-bait-3059**	CAACATGACCGGCAAACAACATTTCCTGCACTCCTGGCACATAGCGAGCTAGTGCCATAATCCTGGAATCAGCCATACCAGTTTTCTCAGTGTCAGCAGAACCGACATTAGCACCCGTCC
**CoV-bait-3060**	CGCGCAGCTGGTAGGTGTGGCGGGTGCCGTCGGCGCAGGCGAAGGCGAAGTGGGTGCTGGTGCAGGTCAGGGCGAACTTGTTGTCGGCCAGGGGGTGGAAGGGGCTGTTGCCCTCGTAGG
**CoV-bait-3061**	CCATTAACAATAGTTGTACACTCAACGCGTGTGGCATGATTGCACTTATAGCACATCATGCAAGTCGAAGAGGTGCAACCATCCATGATATGAACATAGCTCTTCCATATGTAGTAGAAA
**CoV-bait-3062**	TCGCCGCATCACCCGCACGCGCACGATAATACGCTTCTTGTAACGCCGGCGCATTAAATTTTACTTCTAATTGTTGTAACGCTAATAATACCGACGATAAATAACAATTATTATCCGCCC
**CoV-bait-3063**	CATTGGACCTCTCAGCTGCACCAAAATAAACACCATCACCAAAAGTAATGTTAGGATTGTCAAAATAAATCATCCATTCGCCAGGGGCAGAATGTTGAGTAGTGTAACGGGTGACATTAC
**CoV-bait-3064**	CCGGCGCCATTTGTACTATCGATATTATAAACCACATTAACCACGAATTCGATATAAAATGCGACGCAAAATAACCAAAAAATACTTGCATTATCGCCGATAAACCTAATAAATAAAAAA
**CoV-bait-3065**	TATGGGTTGATATGTACACTGTAAAGCCCTCTGGATGGACTGTGCGCACGTTTGATAAACCAAGGAAAAGATTCATAGCCTTTTTCATCGCCGGCATCCTCTTCAGGGCGATAAAAAACC
**CoV-bait-3066**	TGCTTTTCGAACTACCAACACCAACAGCAACATGCCTGCCATTTTTATAATAACTGATAAAAACAGCTTTGGCATTAAAAGTTATGTTAGCAGTACCAGCGGGGTTTTGTGTACAGTTCC
**CoV-bait-3067**	GTAATTGTTGTAATACCGATAATAATACTACCGACGTACATTTTACATCCGACATTTTCGATTGTACCGTCGCTACTTTTATACACGGTTTACCACCTATACCTAATAATTTTATATTTA
**CoV-bait-3068**	AACCTTAACAGTAACGACACCAGGTTTGAAACAATCATCTATGCTAGAGTCAGCAGCCAACACAAACTTAAAGCTTGTGGGCAAATGGTCCTTTAGCTCGTCAAAACAAGAAGAGACCTC
**CoV-bait-3069**	TCGGAGTGTCCCCGCCATTCGCAAGTGACCACGAATGATCACAGCACCAATGACAAGTTCACTTTCCATGAGCGGTCTGGTCACAATTGTCCCCCGGAGAGGCACATTGAGAAGAATGTT
**CoV-bait-3070**	GCATTGGCTGGGTAACGATCAACGTTACAATTCCAAAACAAACAAACACCATCAGTGAATTTATCGTGATGTGTAGCATAAGAATAGAAGAGTTCCTCTATTTTGTAAGCTTTGTCACTA
**CoV-bait-3071**	CGCCTACCGCTAGATGAACCAGAGGTTCTTCGCACTCGAACCGACCACAACGCCCGCAGGACGCTGGATACTAGGTAGCCCAAGCAACGGATGTATAAAGTGAGACACGGCAAAACAAGC

**CoV-bait-3072**	CCAAGTACCATACCCCTAACAATAGACATTGTATTCTTTATAAAATACAAGTAAACAACGTCAACCTTGTCACCCACCTTAATACCAAAGTTACATGGTGGGTCTAAGTCTACATAAATC
**CoV-bait-3073**	ACAGGATTACTTGTGTACATAAACCACACTATAAGTAAAGTAGCAAATACCACCTTACATAACACTCTCTTATAGCTAGAAACTAAACTCTTAGCACTAGCCTTTACGAAATAAAAGAAA
**CoV-bait-3074**	CCTTCGCTAAAGTAAAAGACACTGCTGCATCCTTCGCATACACAAATTTGTGGCCTAAGAATTTGAGTGGCTCACCCTTTTTAAGGGCTGCCACAAGTTCTAAGACACTAGAAGATGAGT
**CoV-bait-3075**	CTCTGTTTTATATTTATTGCAATCTGAAAGCACAGAGACATGTGCATCAGATACATAATCTACAATATCATTATCAACAAGGAGTGTACCTTCAGGGAGCCACTGCTTAAGGACAGTACT
**CoV-bait-3076**	TGCTCTGCGAAAAGGATTATCTTTATTCATACCAGCGGTTGGGTGGGCGTAAACGCCAACCTATAAACTAAATCTAACTGAAACAAAAGCACTCGCCTACTACACGTTAAAACGCGTTTT
**CoV-bait-3077**	GGCCCTACTACATTGTTCACACATTGAATGCCCTTGACAAACGAAGGTGTAACAAGTCTTTGTTGTGGCCCATGTTTCTTAACATAGTCCTCACAATATTCCACAAAATCGGGTCCACAA
**CoV-bait-3078**	TACACCATCAACAAACACCTTTCTACACAAATTACCAAAAGAAGTCTGCGGTATCAGTGTAGAAAACAAGATGTTGAAGTTTGCACGATGTATCAAACACCTGTCGTCAATACAATCACG
**CoV-bait-3079**	CTTCCGAGAAATTGCAGTGTGCAGTACAAAATTGTGTTGTAGACCAAGTCATACCATTATTCGGAGCGGTCATAGCTACAGAAGAAGCATTAAATACATAGCCGCCTTGAATACTACCAC
**CoV-bait-3080**	GGACAGCAATGACAAACACGCAAACATAGGTGTGACATAACCAGGATTTACCCAAAAGAACTTAGTGTAGGAAACTAATTCTGACCAAAAGAAAGTCAGAAAAGAACCAACCAGCAAGAC
**CoV-bait-3081**	CGACAGGGGCGGTCGCATCTCGCGGCTCATGCCGAGGGGGCCTCATATTAACGTCAGTTCTGACCATCTCATAGTTAGAATTATAACTTAGACGCCTCTAGGCATTATGGGCGACACACC
**CoV-bait-3082**	GTCTGCGCTGCGGGACGCGCGAATTGAATTATGGCCCACACCAGTGGCGCGGCGACTTCCAGTTCAACATCAGCCGCTACAGTCAACAGCAACTGATGGAAACCAGCCATCGCCATCTGC
**CoV-bait-3083**	GGGATTTGTCGGTACAAACAGTAGATGAAACCCATGTTGTATAACCGACAGATGAAATTAACTCATTTGAAAATGCTTGATGTTCTCCAAGATTAACACCACCAAGTCTAACTCCTTGTT
**CoV-bait-3084**	GACTGCACCACGTCTCAATGTGTTCAAATTCTTAACGAAATACAAATGTTTAACCTCAGGACCGTTAGGACCTTCTACATAAAACCTTAAAGGTTGCTCTAATTCAATAGTAATAACATC
**CoV-bait-3085**	GTAAACATGTAACATTAACCCAACAGTTATTATCTGTGGTTTTAAGCACACGCCTACCGTTAACAACTTCACTAGGAAAGGCAAACCCAGTATGGTATAGCATGTGGAACTCACCAGCCT
**CoV-bait-3086**	TGCCGGGGGGGTCAGGGTTCAATGCATCTTGGGCTCAGGAGTCAGGTCGCGTGAGCTACTCCGTAAGGAGGCTAATCGTCTAAAATTCACCTCGGCGGTGTGGTAAGCGCTATTGCTGAA
**CoV-bait-3087**	AGTTGCTACCCACGAAGGGCCGGACGTCGTTTCTAGTAATACGACGCACATATATGTCGCCAACACGAACATATTCGGGGCCTGCCCATATGGATACTGCCGACCGTGCCCAAGCTTCAT
**CoV-bait-3088**	AGTGTCATAGATACTATCTTAAACATTAAGACACCGACACACACTTTAAAGTTTGTACTACGGTACAAAGCTAAAAATAAAACAAAGAGCACAGCAATGCTGAACTCTAAATGAAAGCTT
**CoV-bait-3089**	GGTAGTAGTCTCTTGAGAAGGGTCCCGGTCAGCAAGTAATTCAGAATTGAAATCTTTAGCAATCGTCGGGTTATATTGGATCTAGAGAATGCCCTTTCGTGGGTTGGTTGAATAACTATT
**CoV-bait-3090**	TAACGTCTGAGTGCGTCATCCTGATAGTTACTAGTCGACGTTCGGTCGGTGCCACTTAGTAAATACTATTCAACGTTAACGCTTAGCGTTAGAGGTGATAGATCCCCTTGTGCGGGAGGG
**CoV-bait-3091**	CCAAAATATATAATTGGCATGCATTATATTTCCATCCACTATAGTCTTGTCACAGTATGGTCCTAAGTAGTTAATACCTATCAGAAACCCTTCTGATGACGAGGTGTTAACACTTGTACA
**CoV-bait-3092**	TGTCCTCGCGAGAAGGGTCCAAATAAGCATCAACCTGTTGAAGGAAGACAGGGAGGTTCTTATTGTCCTTAGGAACATTCATTTTGTAGTGGTACTTGATTTCAAACTCATTCTGGAACT
**CoV-bait-3093**	ATCTTGTGTAAAGAATGTAGTCGGTAACTTGCACGATCTCTTTGGGAGACCGATGAATCTACTTATAGGGTTCAGGATAAGAGCCAGTCTGATAGACGCGATACCCATTGTGGGACGACT

**CoV-bait-3094**	GCCGATCACTAGTTAGCCACCAATTACAACCATTAAGCAAGGCGCCATACAAGAATGACACAACATTAGCCGTGTACAGTCGCGACGCACCCTCAATCTGTAAAGTGGGTTGGTCGTCAA
**CoV-bait-3095**	TCACCCTCTGCACCTACAAACGAGCCCTTCTTGACAACATTGAAAGCAATGTTTTCAAGACTCTGCAGGTTGGTTTCAGCAAAGGTTTGCCACAGGTTATAAATATCAAAACTCTGTGGT
**CoV-bait-3096**	TCTTCTTGTGTCTCCTCTGAGTCTTAGGTCTGTCATCTTCTTCTTCGTAGACGGTGTCATCGCCGCCTAGCCATTCATCATCAGTCTGCGCACCTTCATCCTTAACCCAGACTTTAACAA
**CoV-bait-3097**	AGCCTTTTAGAACTATATCACCAGGGCTGTAAACTTGTCCGGCATGACCAAGATGGTGGACGTGGCCATTTGTTGGGATGGTTAGGTGTTTGTCAAAGAATGCACACATTTGGTCGTATT
**CoV-bait-3098**	GTAGTCGCAACACATCTGAACAATGCGCTTACGAACAACAGACCACGGTTGTCCCTTACGAAGTAGAGGAACCAAGTGTGAGAACTGTTCGCCTGGTGGAGCACGAGCTCTAACGGGTAC
**CoV-bait-3099**	CTGTAAAAAAGTATGATTCTGTGCGACAATACCACTTTGTGGTGTCCTTTGTAGGCAACAAACCTGGTACATAAACATTAGTCAAATTAGGTGACAAACCAAGATTCAAGTTCTTAGCTA
**CoV-bait-3100**	ACCACTTCCTGCTCCACGACAACAACTTCTTCAATTGCTGAACCACCAATACAAGTAGGTTTCTCAACTGGTTCTTTAATAGATTCAGACTCTTGTTCACACAATTGTTCTTGTGCATCA
**CoV-bait-3101**	CCATCAGGTAATTTAATACTGGCACCATAATTGTAGAGGTTACAAGGCTCGAGGCACATCCTTTGTATTTTATAGATTGATGGCATGGAATAACCACATTTCCATTCAGCAGACTGTAAT
**CoV-bait-3102**	CCAACGTAACCTATCCCATTACGGTCAATCCGCCGTTTGTTCCCACGGAGAATCCGACGGGTTGTTACTCGCTCACATTTAATGTTGATGAAAGCTGGCTACAGGAAGGCCAGACGCGAA
**CoV-bait-3103**	TCTGGGCGGAGTCTCTTGGCTCCGACTAGCTATTCGAAAACAACTTAATATCTCTGTATCCACTAGGTCTCTAAAGACAGTGGGCTCCGTAAACGGCGCACTGTCACGGATTTCTCGCGC
**CoV-bait-3104**	ACCATCTCAGAAACAGTATTAACTATTTCCGCCGGGCAGCGGTAACATTTATGCAAAAACACATCGGGCCCAACAGCGCACATGCGCTGCGTAACAACATTATAGTCTTTAGGCTCTAAA
**CoV-bait-3105**	AGAAAAAGTCCACCAATCATTTTCACAAGTTTAGTTCGAGTTGGTGCTACAATCTCTCTTTAATTGTTTTGTAAAACTTAATACTTTGGATTTTGTGTGTTTAATTCTTCAAAACATCCA
**CoV-bait-3106**	TCTTTACAAAGTACCGATTGATACAAGACCTCCCTTAAAAGAGTGCGAAGCTTTGGGGCGGGACTACTCGGCTGAAGTCGTGCGCGGCTCTATGGACAGCCGTGTACTTAGTCGTAGCGT
**CoV-bait-3107**	AAGTAGTGACATGTAACCGTTTTGAACGATTGATGTGTTGTATTCAACACCGGTATATCCTGCGCAGAAGAGGAAGGTTTCGGAGGAGGAATAGTTGACAGCAAATGTTACTGCTTTCCA
**CoV-bait-3108**	GGGGCGGTCTAATTTAGCGTGTTTAAGGCATGTGTGAGAATGGCAGCATGATTTTTTGAAATAGTAAAGGTAGATTACATAGATAGCTAAGAAATGTACGGCGAGTGCAGGGAGCAAAAG
**CoV-bait-3109**	GTCCAACAACAAATCCATGTATGTGCAAACAACCTTAGAACTTGGGTCATTAAGGTAAGTAACAGTACAGCACCGCAAGGTATTATCAGAAGTGGAGACAAAGTCGTCAGCCTTCAATAT
**CoV-bait-3110**	CCGTCCGGTCCGAATATCGATGCGATAACTGCTTTAGAAGTCCTCAAATCTGTCTAGCTCGTATAGCAGCATAGCCCGTGTAATCTAAGACTAGAGAGGAACAGGTGCGGCATTTTTATG
**CoV-bait-3111**	TGCGAATATGATTCTGTGGTACAAAACCTGTTATAGGGCAAGCACCTGTAGTCGAATTATAACAGTGCGTAACAAACACAGTAATGTCTGAAAATTTACAATGAGCAGTACAAAACTGTG
**CoV-bait-3112**	AATTGTGGAATTACGCCAGAAGATATAATTGGCATGCATGGTGTTGCCGTCAATCACAGCGCCACTTGCAAAATCACCTAAATAGTGAACACCAATTAAAAATGCCTCTGACGATGACGT
**CoV-bait-3113**	AACTTCCTTATGCTCTGTGAAATCATACTTCAACAAATCATAAGTTTTGAAGTCTTGACCAAAAATGTCACTTTTCATAAAACACTCGCTTGCTAAACAATTGGTCATACCCATAACAGG
**CoV-bait-3114**	CAACACCAGCTTGTCAGCACCTAATGACATCAAACCTTCAGGGTTGCACTCATAGAAAGCATGAGCACCTTCAATCCTGAAAGGTTCAAAATTAGGCAAAACTGGTTTTTGTGAAACTTG

**CoV-bait-3115**	GGCAAGGAAAGCTGCTTTGCTTGTAAAGCTGAGTAAGTCAATTGTTGTGGATCCGAGGGTGAGATGAAGGGAACGGTTGTTTACTTGGTTGGTAGCGTACAAGACGTGACGGGTGGTTGC
**CoV-bait-3116**	TCACTTGTTGTGAAAAATCCTGAACCTGTTGTCCCAAAGTATTCAAGGAAGATTGTATTTCCTTAAAATTTTTTTCTGTATAATCAGTAAGAATAGTAATCTGATTCTCAAGAGCCATAA
**CoV-bait-3117**	CAATAAATACAACAGGATGCACCACCAAAAGACTCTTGGTCAATACTAGCATCTGGCTTCGCAGTCAATGCAAGACCAGTTCCAGTATGAGTGGTTAACATTTTAACACAGTTACCAATT
**CoV-bait-3118**	ATGTAAGTAGGTGCGTGAGAAGGTGGCAAATTGTCAGAGCCACGATGACAATCCTTAAAAAGCCCACAGCCATCACTAGATTGCAAATCAGTCATTTTAAGCTCATAAAATTTGAGCGCA
**CoV-bait-3119**	TAGGCAGTAGTGGCATCACCAGAAGTAGTACCACCAGGCTTCATATAGAAACCCCCGTTAGAATAGACCACCTCAGTAAGAACTTGTGCAAGTTCATTACAAAGTCTATAATAACGATCT
**CoV-bait-3120**	CAAAGTATGACCTGTAGTACCAAGGTAATGAATGCTCTCAATGGCTAAAACATTTTGCTGTTCATAAGAAAGGTCTTTACGTATAACATCCCATGCTAGTTGATAAGTGACACCACCAAT
**CoV-bait-3121**	CTTTGGTTACAGCAAGTGTGTTTTCGTGCTAGCAAGCACGAAGCGGCAATGCGCAGCATGTTTGGCATAGAACGATCACACTTAGGGTAATCCCAACCCACTAAAATGGGATTGTTGATA
**CoV-bait-3122**	TTGTAAGAGGGTAAGCGTCTATTGCCAACGAAACATAGCGCTCAAGCAATATAACGGCATCTGTTTTTACCACATCATCAACAAACACTCCTGCTGATAAAATTCTAGATGGGTCAGGAT
**CoV-bait-3123**	GAATACCAGAGACAGGATAAGGTACACCAGTAATAGAATTACCTTCAGACCAAGTAACATACAAAGCACCAACAATAGTATAAGAAGTGTAATGCCAGGTGGCTTCAAGCGGAAAATTAC
**CoV-bait-3124**	ACTAACATCATTAGTTTCCAAATGAGCACTAAGTCGTAAGGCATCTTCAATAGTTTTACAAGCAGAAGTATACTGCTTAAGTAGATTCTTACAGCGAGGGTTACCATTACACACATAAGT
**CoV-bait-3125**	AAGAGCACTACCCTGTTGATTAACAACATCCTGAATCTTATTAAGTGCAATAGTAACAGTATGTATAGCCTCTGCAGTTTGTGTAATAGCATCATTAACGCTACTAAAAGAAGCAACAAT
**CoV-bait-3126**	TTTGCACAAAATCAGACAAAACAGGTAAGCGAGGTTGAAACATGACCCTGGAAGTTACACGAAAGACACCATTATCAGAATAAAGAACCAAGTTAGGTTGACGCAGAACATAGCCATAAA
**CoV-bait-3127**	GCTGGATCCTGTACAAACACACCAGTACCTTTCATACTAACAAGCTTATAAGTCTGCATTTTATGACAAATACAACACTCACCTTTTTGAAAAAGTTTTGTGTAAGGCATAATCCAAAAC
**CoV-bait-3128**	ACATCGTACTGTAAAGTTTTAAAAACTATAACACCACTCTTGAGATTGTCCAAAGAATTAGCACTGAAGTTAAGATTGTAACGCATAACATCAACAACAGAATTATGTTGATAGCCGTTA
**CoV-bait-3129**	TCGCGCAAGCAAGTCTTTTATGCATTCCAGAGGATAACCATCTGGTCCGCAGAAGTTGTTATCAACATAGCGTGTGTAGACACCACCATTGAGCTCCCTAAAGAGATCGCGGCGAAGCCC
**CoV-bait-3130**	TCAGTCTTCAGTAAGTCATCCACAAAACAACCTGCTCCCAATATTCTAGAAGGATCCGGATATGGCAGGTAAACTTGTTCACCCTTCATGTCCACCAACATAGTATGTTGAGAGCAAAAC
**CoV-bait-3131**	CTACTTCATAGTGCCATACACCATCAAGACGGTAAGTAACGAACTCACTCTTGTTAGTGTATTTAAGGGCTTTGTAACCTGTGTCAGAGACAGGAACAATGGTCTCCGAGAACTTTGTAA
**CoV-bait-3132**	TGAACCACAAGCTCCATTGATGAAAGAACCTCTGATTGTGTTATTTGTGCGTAATGTTACGCCATAAACACCAGAAGGAACACCATCATAACAAGCAAGAATGTTAAAGGAATCACCCTG
**CoV-bait-3133**	AACCTCACCAAAATTTATAGCACGCACTCTAGCACCAGCAACAGTAATGTAATCAGTACAGAGCTCAAAAACCTTGTTAACGACGTCAACAAATGATGTAACGCACTCTATGAGACTAGT
**CoV-bait-3134**	AAATAATCTGCCCCACATACCATACTGTGTCTGTAAGGAATAAATTAAACATTTCTTTCGAGCAACAAGGCCCTAAAAAGAATAAAATCAACAGCTTCTTCTACTAACTTACTAACGGCC
**CoV-bait-3135**	TGTTAAGACCATCAAGACTAACATTCTGTGAAGTATGGTTTTTAAAATACTTGTCAAGTTCTTCTTTAAATGAGTCTAGTTCAGGCTGTAGTGGGTCGTAGACAGTGTTATTAACAATGC
**CoV-bait-3136**	TTTTATAAGGCTGTCCACTATTGACAGCAAACATTGGATCAGCACAAAAATTAAAGTTACACACATCTATAACAATGTGTGTACCATTATTAAAGAGAACAGCAGACTGAGTTGTGTTGT

**CoV-bait-3137**	AGCGACAGCCTCTTCTACGGTGTCACCGAATCCACGTACGAGCACGTCACGAACCTGCAAAACAGGTAAACTGAGTTGGACGTGTGTTTTCTCGTTGAAACCAGGGACAAGGCTCTCCAT
**CoV-bait-3138**	TAGTAGAGCCAATGAAAACAACATTCCCTACAGCATTTTCATCACAATCCACTGTAGTAGGACCATCAGTAGCAAGGAGCAACAGATATGGTAGTGAGGCTTCTACCGCCTCATCCACAC
**CoV-bait-3139**	GACACACAGAGTTAAAATATTCAGGTTCTAGTGTGCCCTTTGTAAGCAATGTGCGTGGTGCAGGTAATTGAGCAGGATCACCAATGTAGACATAATGCTTAGCACGTAATCTAGCATTAA
**CoV-bait-3140**	TTTTTCCACATAGAGGATAGCTTACCAGCTCCCTGTTTTTGCAACACACTAACACAAGGTAAGGTAGTTTCCATAACATTGTTATTAACAGTAAGGAGAAAAGTTACACCTTTAACCTTA
**CoV-bait-3141**	TAGAACGCAAAGCTCTAAAATTATATTTAAAACTCCTGCGTAACACTATACCACTACGTTCAGGTATACTAGACAATAATTTAAAATTACCACCACGCCAGCACCTATAAAGAGTGCTAA
**CoV-bait-3142**	TCAACATCAGAATCTATAGCACACTTACGTCGAGCGCAACCAACAAAGGTGTCCATAACCTGTTCAAGCTGCACACCCTCCTTTAAAGAATTGTGCGCAGCATTTACAAAACTTGTTAGA
**CoV-bait-3143**	ACAAGTGATTCAACACCATTAGCAGAAGGTCCCAACTGTGCATCAAAGGTTTCCGCAGTTGTAACTACAACATCATTGACATTGCGACCATCCGCTGTGACTTTAACTTTAACATCTTTA
**CoV-bait-3144**	TCCATCAGCGTGACCATTGAGAATAACAGAGGCCTCAGCTATAGCACCTGTAACAGTTTTAACTGTACTATTACAACGGTCGCACAATGTAGAACGTTCAACAGTAACAGAACCACTAGA
**CoV-bait-3145**	TTTTACAAAACAATCATGAATTGCAAGACATCTAGTCATAATAGCGTCACCTGAAGCAACATGCTCATTACGGTGCACATTACAAACTTCATGATGGTTACTGCTAAGAGACCCTGTATA
**CoV-bait-3146**	GTATACTTGCAAACAGATTTAGTAAAATTAGAAAAGGGTCTGTCAGCATCATAGTCCCATAACACGAAACCATGTGTAGCAACAACACCCATATTTTTCAGAATGGTAAGTGGCGGTGTA
**CoV-bait-3147**	CTAGGTCTATTAAAATATGTGAGCGATTTCAGCGATGCTTCCTCATGGCCAAGCCAGACAACCGGTTTACCAAAAGTAATGCACCCGCTTAAGTACCGACTCACATACAAATCATCACTA
**CoV-bait-3148**	TACTCCCTTGTCTATGTACACGATATGAGCAGCCTGCGATACTGGTTACTTGCCTTTCTGATCGCGGGGACGCGAGTATGTACGAGGCGCGATAAAACCCAGCCCCGGGACTCAGTATTA
**CoV-bait-3149**	AGCACACCATTTTCTATAACTTTAAAACTGGCATATAAAGGCAACTGCATATCTGATTTGTGCACCGCATAAGTCGGAATAAGAGCCCACAATACAATGAAACAAATCAAATTAGCAACA
**CoV-bait-3150**	TTGGGTTTACATTTCACATGCGTGTAATGGCCCAAACTACCACCAGTAAAAATATTAGCTGCAACAACATCGTCAGGCAGTTTCCTGCCCTCTGGAGTGTAGGAGCAAATTAAGAATGGT
**CoV-bait-3151**	TCACACAGCTTGGTTTTATAACAAAATTCTGTAACGGACGAATCCGTATACAGCATAGTCTCCACCAAACGGGGGAGTCTTTTAACAAGGGCATCAGCTGCTAGAAGATGAGCAGGCTCA
**CoV-bait-3152**	CATTAAGTGAAGACAGTAGTGTAGAATCAATAATCTTAATAGGCTTACACAGAAGTTGTGAGAGATAAACACACGCATTCTGAACCTGAGACAAGTTTGAACCATTATTGTCAAAGACAA
**CoV-bait-3153**	CTTTATAATGCCAAAACTCTATATCACAAAACACAAGAGATGACACTTCTGGTATTATATTAAAATCAGACTGCTTTTCCATGCCAAAATAACGATACTTTGGTTCTGGCCAATTTAAAA
**CoV-bait-3154**	TTACACACATCAATAGTGTAGGGTGTTAAAGGCCTTTTGGCCTCATAATCCCAAAGCACATGTTTATAAGTAGCAGCAACACCAAGGTTGCGTAAAATTGTGAGAGGTGGTGTCAACCCA
**CoV-bait-3155**	ATTGTACATGCGTTTTTTCATTTACACCTAATACTAACGATTCCATCTTACCTTTCGGTCACACCCGGACGAAACCTAGGTATGCTGATGATCGACTGCAACACGGACGAAACCGTAAGC
**CoV-bait-3156**	AGGAATTGCATGCTAGTTCAGTCTGTTTACCGGCCTGCAACCTAACAGTAGCACCTATATAGCCTAAAGTAGCACCTCTACGCAAATTGTTAAGATTTTTAACGTAGTAAAGGTACTTGA
**CoV-bait-3157**	CAACAATGGCAGAAGCAAAAACAGCATTCTTCAAACCAGATTCACGCTTACCTGACAGCTTGAAAGTTTCTAACTTGAAGAGTGCATTCTCAAATAAGATGTATTTACAACCAAGCACAA
**CoV-bait-3158**	GCATCCATGACTCCTGGGTAAGGCAAACGTCTAGACAAGAAATAATACGCTACAGCATAAACAACCACTAACACCAAATTATGTGTGAAAAAATATGATAAATTGTTAATAAAGAGTGTA
**CoV-bait-3159**	AAGTGATGTTTGTGAATTACAAACAACACAATTACCAACACTCTGCAAAGACGCAGATTCAGTGTACATACTCTCATAAAAATCGACTTCCCAATACTTGGAAACATTGTCATTGGCTAA

**CoV-bait-3160**	ACTTCAAGCTGGAATTGTGGTGGTATTTTACCATCAAATCTCAGTGGTTTGGATTCATTGTTCGTTCCACGAGTGCCAAGTGATGTTGGCTTGTTCATGGCACCATCCTTTGCAACCCAG
**CoV-bait-3161**	CCAGCCTTTACATAAACTACTAAAGCGCAATGTAGTAAAGAGCAAACTATAACCTATATAGAATGCGCTAGCTACTATGCCAAATATGTAAAACCATCTACGTTTTGCATAGATGCCACT
**CoV-bait-3162**	TAAGATTGAAAAACCTATTTACCCAATAAAGGGCACCATAAAAACAGCAAAGAAAGTAGCCAATAGTCATATACAAAAACAAAACACCTTTTAACTGACCTACGAATTGCACAAGACCAG
**CoV-bait-3163**	CTACATCAGGAGAACCTGTAGCAGAGTTTTTGTAAAGTCCAAAAACATTACCAGCAGAGCAAAGAGGGAACGCAAGGCGAGGCTTGTGATCAACGCACCAATAACTCAAACCACCAAGAT
**CoV-bait-3164**	CAAGGGCTAACTATGTCCGGTTGAGCACACGGGCAATAACTAGATCTTACAGTAAAACATTGCTGAGCGTAAACAACGTCATGTTGGTTTTTGCCAAAGACGCCAGCATCATTAAAGCCA
**CoV-bait-3165**	CCGCGGCAATGTATCTTAAAACATTGCTTGCTTTCAGGATGGACTGGGACAAACTGATTCTCATAAACCATTTCAGAGACCGTCTTCACAATTTCGGCGGGACAGCGGTAACACTTATGG
**CoV-bait-3166**	TAATACCAGTTGTCCAAGGTTCTTGAATAGCCTGTTGCTGTTAACTGCTCGGAACGTTGCAAAAGAGTTCTAAAAGACTCCTTACACCAATTGGGAACGTAAATATACTTTCGGTCATCA
**CoV-bait-3167**	TAATGGTACGTAGAATCCTACCAATATCAACTGAAGTCAAAGCAGCCAATTTATTAATAGCCACATTGTCAACAAAAGAAGTGAAACCACTATCAGCAGCCCACTTATTATAATCTTGAA
**CoV-bait-3168**	TGTAATCTGCACCACTCTCAATAGCCAGTGAAGAGCACCATGTTGATGTTCAAGCCCTGCGTCTTCAACCTCACTAAAAACACGGGCCTTGCTTAGTTCTTTAACTTGATTCTCAGGCAC
**CoV-bait-3169**	GAGTGTAGCAAACACTGCCTGTATTAGCAAAGACTCGGGAAACAAAGAAAATTATCTGATTGTTCACCCAAGCCAATGTAGTAGGTACGTCTGGAATGCGAGCACCAGCAACTCCAGCAA
**CoV-bait-3170**	ACGAAGATATAGCACCGAAGGTATTAGATAACTCCGATGCTAACTTAGATAGAGCTTGTGCATTGGCATTCACGGCATCTTGCACCTTTGAAAAGGCAAGATTTGTGGTGGTGAAGCCAG
**CoV-bait-3171**	ATTAGCATTTTGACCAATACGTATGACAATTCCATCACCAAATGGATTGCCATAGTATGAATAATTACTAATAAAAGCATCATTAGCAGTGCTTTTATGATTGGACGCTGAATAGACATA
**CoV-bait-3172**	TGTAGCAATTAGCATCACTCAACCTCAAAGAGCGGGCCTTATCACATACAAGCATCTTCCACCCAGCAACCTTAGCCTTTAGGGAATAATACCTGTATAGGAACGTATCATCAGGAGTAC
**CoV-bait-3173**	CACTGGTAACAACTTTGGGTACTTGCAAAAGTGTCAAATTAAAGTCGCCATTCAAACCAGCATCAAGTGGTTGGGTGGCCTCTGTCTTTATATTTGCAAATAAGTTGGCAATGGATTCAT
**CoV-bait-3174**	ACTTATTTAAAGTAATATTATTATAATTGTGTTGTGTTAATACCAGTGGTTCACTTCTGGTTTGTAAACGAGAGCCATCACTCTTAGTTACATAAACCAGAAGTCCGCATTCAAAATTTT
**CoV-bait-3175**	GGATCCGCCTCAAAATCGTCCATCCACTCAGGGCAAGAGGTGTTGTCTCGCTCATAGTGGAATGGGGTGTAGTGATAACCACCACGGCCATACTTGCGCAGGAGAATATTTTGCTTTCCT
**CoV-bait-3176**	AAAGCCCATCCTGGAAATAACACGAGAGTATGGGACATTTGCGGGTAAATTAAGATTCACGCAAAGCTCATCACTCGTCTTATACTTGTCATCAACACTAACGTATGTTGGTGCATAAGC
**CoV-bait-3177**	TAGAAACCCACAACACTGGTTTACCATTAAGCATGGCACCATTCTTGTAAATGGGGTCATATGTGCTATACTCAGCCATAAGGATATCACCATTTTCATCTGGTAGGACTGAGTAAGTAT
**CoV-bait-3178**	TAATAAGCTGCTTTACCATTGAGGAAAGAAAGTGTCGTAGTATTAAAAATAATAAAGAGCGCATTTTTATAGCGCCAGGAGTAAAAGCAGACTAAACAAAGCCTGCCAATAAGTGTGCAA
**CoV-bait-3179**	AAAAAACCAAGAAGATGAAATGTCAAGTTTTCCAAAACGAGTAAGTGTATAAATTGGACAATAAATTGTTGTTGAATTGGCTGCTGCAACAGACCACATTGACATATGCCTAGCTAGCAA
**CoV-bait-3180**	ATTGTTATAAACAGTATCACCTGTCTGACAACGCCAATTGTTTTCAATCGGCAAATAACCACTAACAAAAACATTAGAGTTGGCAGGCAAACCAAGGTTTAAGTTAGGTAATGCAGCAGG
**CoV-bait-3181**	GTCTGCTATGGCCTGTGCCACACCACCGGCATGACGCATGTGCTCATTAGCAGCATTAACCAAAACAAAATCCTCATAGGAGTCCATTGCCTTAGCAACGACTACAGACAAATCACCCAC

**CoV-bait-3182**	TCATTATTGGTTTTAAAACGGTCTGAGAGTGACAAAAATGTATGAGCATACGCTGGTGGGAGGTCTACGGGGTCACGAGCACAGTCCTTAAAGAGACCACAAGTTTGCTCAGACTGCAAA
**CoV-bait-3183**	GCCGGTTGAGAAACCTAACTGAAGTGGTACATTAGTACCAACTGCATCTTTTGTAGCATGAGCACCTTCAACATCGAAACCTACCCACGCTCTAACTTGTTTAATCGCCTCATCCCGGGT
**CoV-bait-3184**	TACCACAACCACAGACTACAGAACAGGTAATCTTAAGTGTGTGTAGGTCCTTAGTCAGAGACTCTAGGCAAGCCGACACATCACCCAAAGATCCCAAGATAGCCTTTTGTGACTCATAGC
**CoV-bait-3185**	ACAGACCTACCCTCCTTCCACTGGAAGAAATCATGCTCAGCAACTGCACCACAATCTTTAAGCTTTAGATATATGGACTGCTCGTGGTCCATAACAGATTTAGGACACCTCTTGATAACA
**CoV-bait-3186**	TTTTCAATAAAACCTGTTAAAGGACAGGCACCACGTTTAAAACAATGTGTAACAAACACTGTAAAATCAGTAAAATTACAGTGAGCCGTGCAAAATTCAGAGGTAGACCACCGCATACCG
**CoV-bait-3187**	CTTAAAGACATTCGCGGCTGTAGGTGGGTTGTGATACCGCAAAAGGCTACAATACGGGATGCCAACAATTGAGCGAGGGTGCGATCTGCAAGAGATCCACCAGTAACATGGGGCGGTTCT
**CoV-bait-3188**	TTAGCACTACATGTTTATGGTCTTTCAGTTTGTCTGATCCTCCGTATGTTGGTAATTTTTCAATTTCGTTTGATAACAGAAGAGATGCGTCATGTTTGTTAAAGATTGCGCATGTGACTC
**CoV-bait-3189**	AGCCTATATCGTGCCATAGGCGTCCCCGGTTCATGTCGGTGTCTGTCTTGTGCTTGGAATCAGGACAAAAGCCCCATTCCCGGTCGACAGACGACTATAGACGTGCGTTCTGGTATCCCA
**CoV-bait-3190**	TTTAAATTAACAACAAGTGCGTTATTACAACGACACTTAAATTTCGGAGTATCTAGGACCGAATTGTGTGACAATGCCATGATCGTGGAGTTTCTCCAAAATATATAATTGGCATGCATT
**CoV-bait-3191**	TTGTGCCATCTTACGTAAACCTGCTTGTAAAGTTGAATTGTAACTAACAGTAGGTGGTGTATAGAGTGCATCCTGATGAGAATTAGCAAAATCCAACATAGCCTTTGCTAAATGAGCAAA
**CoV-bait-3192**	TCTGTCAAGAGTAATAAGTTCTTCGCCAGTTAAGGTGGAACTTATACCGATGTGTGGAGGGTGAGGATCTGGATCGACTAGGTGTTTTCGTACAACTAGAACGGTGGTGTTAGGATGAGT
**CoV-bait-3193**	TTTTGGTTACAACATTGTAATCCTGTGGTTGAAGTACACCCTTATTAATCAAAGTTCTAGGAGCTGGTAGCTGCTGTGGGTCTCCAACATACACAATGTGTTTGTAACTCAGTCGGCTAT
**CoV-bait-3194**	TACTTAAATTCAGCAGAACTAACTTTAAATTCATATACACCCATGCTAAGCTTTAGAAATCTATTCATCCAATACAGTATGCCATAAAACATACACACTAGATAACCTAGAAACAAGTAG
**CoV-bait-3195**	CAACGCTTTCCATGGTGTTGTCCACAAGTGGCTGACGAACAAGCACGGATTGTGAAACAACCACAGCGGACGAAACCTACTGTGTGGTTAATCGGACGCGACACGGACGAAACCGTAGGC
**CoV-bait-3196**	TCTACCGTTCGTTTCCAGGCTACTAAGTAGACAGATTAGACATTTCCCTGGCGATAGACATGTAGGTAATCAAACTACATGCCTATCTTCCTTGATTCTTATTGATACTCTAAAATAAAG
**CoV-bait-3197**	CAGCACTCCAAGAACCTAAGTTATCTGGGGCCATAATAAGAATAGCAATCCTGTCATTTTCAAATGTATCACCAAAAGCTACACCAAAAGGCTCTTGACAATAAGCACGCGAAGACGCAG
**CoV-bait-3198**	GACTAAACATCCTAAGCAGACACTTAATAATAATAACCACATAACAACAGTGAGTATTCCAGAAAACTTAGGTGATTTAACATACATAGTCAAAGCATCTAGACAAAATCTAACACCACC
**CoV-bait-3199**	CTTGTAAACACCCACCCTCATAACACTCAAAATATTTATCAACTACCTCCATAGTAAAAAGCAGTTGTTTAATATCACACATAGTAGGTAAATTATAACGATAATAGTTATAATCCCTTA
**CoV-bait-3200**	AAGCTGTACAAAATTGAGCATTAGTCCACCGCATGCCGCTTGTAGGTGCTGTCATGACAACACCTGCGGACGAAAAATTATGCCCTTGCCACCCTTGTAAAGTATTAAGCGTGCAAGTGG
**CoV-bait-3201**	AAGTTTGACTAATAGCCTCTTTAACACTCTCAAAGGCTGAAGTTATATTACCAATAGCAGAGTTAAAAGACTCAGCAAGCAATTGCTGGTTCCGCTGTAGAACATCCGTCTGTAGAGCAA
**CoV-bait-3202**	TATCTCGGAATATCTAGCCCACGCCGAATGATACGAAGGATAATATTGTTGCCGGCTCCAACCCTTTATAGCGGTGCGACCTCGGCCACGAGTAGCTTGCTAAGTACAACACGTTCGAAC
**CoV-bait-3203**	ACTTGATGATGTTAAGTGGGCTGTTGCAATAAAGGAGACGCTGGATGTTGGTGGAATTAATATCAACCAACACAGGAGCATTATTAGCAAAGGCAACAGTCCAAAAATCACGGCTGTCAT

**CoV-bait-3204**	TAGTTGATCTGGTCTTGGACCACATCACATGCACTCTCATCAAAATAGAAAAATGGCATTTGTTTGAGCTTAGCAAAAGCTCTCTTATCGAAAGCAGGTGTATGGAAGGCATGGTTGTTC
**CoV-bait-3205**	CAGCAAGGACGCCATCAGCGTCAAATCCATATTGATCTGTTAGAAAGGTGTTTCCTCCACGCTTAGCCTTGGGCTTCGTGGGAAAGCACTTGTCAGGAAAATCTAAATACTCTTCCGCGG
**CoV-bait-3206**	AGATCATAATTTGTACACATGGAAACCTCGTCAACAACCACAATATCAGCATTGCACTCGGGCAGCGCATTAACTGTAGAAAAAATGTACTGCGCACTAGTGTTATTGGGCTTAAAGCCA
**CoV-bait-3207**	AATATCAGTCACATAACCCTTACTTGCATAATCCGCATTATAGCAGACGACACCATCATCACTAAGTATCATCATACTAAAGTGCTTACGCAAGTAAGTATAATATTCTGTAACTAAATC
**CoV-bait-3208**	AATCAAAAAAGCTTAGTGCAAAGAAAACAGCAACTATGAACAGACACAAAAACCAGAAGAATTTCTTAACCTTTGAAAAACTAGGAAGACCTGCACCCTTCTTATTTGCAATGCAAACAG
**CoV-bait-3209**	CAAAGAGCCACACACTTAAGCATAGCATTTGCAACAATGTGTCCTAACTTGGCATATACAACATGAATAGCTTCATTCTCCACAGGGTCATACCAGTCAGGATTATCAAAGAAGGTACTA
**CoV-bait-3210**	CATAGGTAACAGTACGATTAGTGCAGTTACCAACTGCTATAACACATTGATGACATTCATCTGAGTAATAGCAGTAAAGGGCTGATGCTGACAAATAAACACAAGGGTCAGGTCTTGGTG
**CoV-bait-3211**	TGTGCTTATTCAACTTGTCGAATGAAAAAGGACAACTACCTGATCCAACTATAACCTCTGTCCTAGCTGCGCCAGTACAGTCACTGCCATATTCAACGCTGCAATGAGACTCAAATCTAA
**CoV-bait-3212**	ACGGTACCATGAATTACCGAGCGTGGGGTGAGTTCCGGAGTAGGTAACTCGGGGGAGGACAAAATTTAAGGTGTTTGGAGTTCCGTTCGACGCTTGCCGTCCGCTTGTTGATATTTATCC
**CoV-bait-3213**	CCAAGTTCATCCCTAAATGGGAAAGCGAACGGATGTTCAGGTACAGGTGCGCTTTTAACAACGGGACCGAAAGCTTTATTAACTTGTTCAACCTCAGACGTTTGTTCTGTCACGAAGTCA
**CoV-bait-3214**	CATAATGACTGCAAAAACGACTGGTGTATAGGCTTTAAAAACACCTTGAAACCTAATAACCAGTGCAAACACAACCATAACACACAACAATACCAAAAACATAGTTGTTAGGTGCAAATA
**CoV-bait-3215**	CTACATGTTGTGGTGGCATGTAAGTACGGTAATCAGCAACAATAACTTGATAGGCATTTGAGACAAAGTCTCTAATGTCAAGATCTATAATAACTGTACCTTCTGGAAACATTTGTCTAA
**CoV-bait-3216**	AGTACCATAGAATGGAATGTAAAACTCCATGCTATTGATAAATGTAACCACACTAGTAGTGACAATAAACAAAAGAGGTTTAAAATATAACAACACCACTATAAGCGACCATAAGGTAAC
**CoV-bait-3217**	CGGAACATTCCATGTAGAAGCAGGTGTTTCAAAGAGATGCTGTAACGTATATTTATGTAGATCATACGCATGCATAATTATATTATCACGGTGAAACAAATGTCCTGGTATGCCTGGTAC
**CoV-bait-3218**	GGATGCTGTGGCTGGTGTTTCAGGATTGGAGGCTACTAGGGCGTTGATGGCAAGGAGTTTGTCAATGTATGAACCTGTGATGAGGGTTTGCTGTAGTTTTGGTCTGTCTACTGTGTATCC
**CoV-bait-3219**	CCGAGAAAAGGACGTGCGTAAACGTCACACAGGCTACAACTGTAAATGGCCTTTCAGCTTGCGTGATGTTAACAACATCACCCTGTGGGCACGAGATAAGAGTATGTTTATGGCAGGCAG
**CoV-bait-3220**	ATTTGATATCATTGATATGACTGTTGCAGTAAGTGACACTCTTAATTTGTGTGTCTGACACTTCTAAGAGGACTTCCGTAAATGTTGTATAGGCAACAGTCCAAAATGCACCACTACTGC
**CoV-bait-3221**	ACCCTTAGTGGAAATGTAATAAATCATGGCATATGCCAACATGCCTAGAAAATTTTGAGTGACAATGTAGGAAATGTTGTTAACAAGCGTGCAAGCACAAACTGTTGCAACACCTAAAGA
**CoV-bait-3222**	ATGGTGCCTTAACTAAGTGACGGTCAGCTACGGTAAAATGCTGTAAGTGTCTGACGGCTTGATCCCTGCTTACCCTGTAGCATTTTAAGGGGCTTGCGTATTTTACGAAATAGCAATGCG
**CoV-bait-3223**	GCTGCAAGACAACCACCTTCATAACATTCAAAATATTTAAGAACAACTTGTTTTACAAACAAAGCTTGACAAATATCTAACATTATACACTTATTATACCTATAATAATCAAAATCTGTT
**CoV-bait-3224**	TTGAGGACAAGCTCGAGGGCCGGAGGTTAAAATTGCATTAATAGGCTCAGGATCACAAGTGCATGCACATTGAATAGGATTAAAACAAGTGCGATAAGATGTACCTACTGGACAAGTGCC

**CoV-bait-3225**	TATCTATATCTTCTGCAACCTGAACAGGAGATTGTGGTTTAGCATCCTCCTTTGGTGCCTCAACCTCAACCTGCTGTATTTGGTTGTCTTGAAGGACAGTGTATTTATTCTCAAGAGTAA
**CoV-bait-3226**	TGATCATGCACAAAACCATCAGAATCCGCAGATACAACATCTGCTTCATCATACGCTTGTATGTTGACATGAAGTTCTTCCATAGAATCTTCAACCTCAACTATATCTAAGACTTCTGAT
**CoV-bait-3227**	AAGACAGGAATTAATGCCTGCACTCAGAATAAGGGTGATACTAGCTCGCGATAGTTGCTCTGTAAAAGCGAACATGTTACTTTTAATTGTCTTTTCGGGCGATTGGTGTAACCTCTCCTA
**CoV-bait-3228**	CAAATGAAAAATATGCCGACATAAAAGGAGCATGCTCCATAATTATGGCAGAAACAACATAATCCCAGAGAAAGTTGCTGAAAACAACAACCAATGTAGCTGGAACCACATAGCCATAAA
**CoV-bait-3229**	ATAGAATTCCTTGTTAAAATGACCAGGTTGCATAGTTTGCTTAGTCATACCTGTACCTAGTGCAGCGACTGAGAAGCACACAGTACGCTGATCAACCAGGGCTGGTGAAGAAGCAACAAG
**CoV-bait-3230**	GCTAACTTACCTACATAACCCCATTGTTGTACATCTACAAGTCGAGGGTTATATAAATAATCACAACCATAACCATGAAACAAACATTTATACTCTACACTTTCACTATCATAGAATTTA
**CoV-bait-3231**	ACTAACTCATTAGTGTCAACATCCGACTCTAAATCACGCGCTTCTTCGCGAGTTGAACCAACAAACACTTTAAGCACAGCCTCAACGGATGTTCCGGATGCTATTTGCTGAAGTGCTGTT
**CoV-bait-3232**	GCCAACTTTGAAGTAGCATTACTCTTATAATAAACACTATCAGAAGAACAATCAACTTTTTCAAAAACATATTCACCAACTTGAATACGAGTACCTTTTACCATATTATAACAAACAAAA
**CoV-bait-3233**	AAATATTATTTTCTATACAAAAACCTACTGGGTCTTTAGTTCCAAGTGGTACTTGAACAAATCTACCCTTAAATTGACACAAACCATCTACGGTTGGATGTTCAACATAAGCACGACAAT
**CoV-bait-3234**	ACTACCATCATTACAGCATTCTGGGTAATAATATATGACAAATTGTTCACAATAGCCACACAAACAACAGTGCACACTCCCAATGAGAGATCACCGAACATACGTTTAAGTTTTGTGACA
**CoV-bait-3235**	GCAACCGAAGTTCTTGAGGCAAAGTAACAGGTGGTACTTTTGACTTAAAGCCAACAACCCAGTCCGTTGTTAAATTATAATTACAAGTAACCCTATCACACCCAAGTGAGTGCAACCGAT
**CoV-bait-3236**	AATGCACCAGTGTCAACTAAAGCAAATGATGCTTCTCCATCTGTAGTGTCCAATACAAAGAAACCTTTGCCGCTATAAGACACATTGTTATGATTATGGTTGCTGGTGCAATGCCATTTA
**CoV-bait-3237**	CTCCAACTAAACTTAAACACGACTAACAAAAAAAACTTATAATTACAAATATTAAAATAACAACCACCACCAACAAAACCCAGGTAGCCACAAAATAAGAAGAAGAAATAACATCTATAT
**CoV-bait-3238**	TAGACTCTAGCCTAAAATTACACCTACTACCTGGCTTACCATAATAAGAACCTCTATCAAAATCAGATTCTACATAAGTAAAGCTATTCAGAAAATTAAATTTAAAATTAACATAGCCTT
**CoV-bait-3239**	TGATACAACTTTGTACCATATCTGAGCACATTTCTTGTAAAGTAGTAGTTGTGGATGTTCCCTGCCCAGTCAAAACTGAAGGGATCATCAATGATAACTCCACATAGTTGCCTTTTGGGA
**CoV-bait-3240**	GAGAGAACGAAGTCTAATCTGCCGATACTGTAAACGATATTTAAGTTTGTAGTTTCAATGGGTTTACCTTGCCAATGGAAATATGCTGAGTCTGCTGTGATACAGAATCTGACATTGATG
**CoV-bait-3241**	AGGGAATCCGTGTCTCACTCATTCGCGTACAGAAAAACGGGCTGAAGATTAAAATGTCCGACTTCGGCGAATCCGGACGTTTAGCTCGCGAGTCGCGAAACCAGGAGGGCAAAGTAGCAC
**CoV-bait-3242**	TTGTACCACACCTCTGTAGGATAATAGCCACCAACAACTACAGTTCCTTCTTCTTTAAAGTTTTGAAACAAAAAGTCTCTAATGAGATTTTCGTTGCCATCTAATTGTGTTACGTTAACT
**CoV-bait-3243**	TTATAGCGGTAAAAATCGAAGTCTTTAACAGCCGCATCACCTTTCTGAGCAAAGAAGAAATGCTTAAGTGTCAACTCAGACCCCTCTTCAAAGAAACCATGGCCTCTAAGGAAGTTATAA
**CoV-bait-3244**	ATTAGGCGTTTCTTCACCAACTCTTTCCAGCAATGAGGTACCATATCCATTGTGTACTCTACCTGGGGCTCAAACTGCAACTCACTCAAATTATCACTCTGATCTTCACACCTCAAAACT
**CoV-bait-3245**	TTAACTTCACAATGTGCACACCCGCATCTATAACTTCTGGGAACCTAATGTAGCCAGCGGTCTCATAGAATGGGTACAGCACATGTGGTTTAAGCATTCCATAATGTGTAGCATTGGGTA
**CoV-bait-3246**	TTAGCAACTCCATTCATCAATAAACCAAAGACACTTCTGCATGTGTTTTTAGTTAAGACCATAGGGACTTTAACTGACACACGTTGTTTTGTTCTTGTGAACAAGGGTACTAAATAAGAC
**CoV-bait-3247**	TTTAGCTGTACTGGCCGTCGGCTGATTCCTGGCACAACGGCCGGACCAGTGCAGTAATCTCGGTGTATACATATTCGCTAACTACTGAAAGAGGACGGGCTCGTGTGGATAAGATCCACT

**CoV-bait-3248**	AGTTACAGTGTGCAGTACAAAAAGAACGCGCAGACCAAGACATTCCGCTTCCAACAGGTGCTGTCATAGCAATTGCAGAGGCACTAACATTTAAACTTTGACCAAGAGAACCAACAGCAC
**CoV-bait-3249**	GAGGTGTCTTCCATGCTTGCATCAGAATCACTTTTGACATCATCAGCTGTGTTAAGATCGTACTGTGAGATCATAACACCATCTGGGTTATTAATGTCAAATCCACCGCATGTGTCGTAA
**CoV-bait-3250**	TCTGTAGCACGTTTCAACGATTGGGGGGTGAGCGGTGGTTACCCGATAAACACTCCAACCTGTGGCTATATGCATCGGACACAAGTTCAAGTGTCAAGTAACGAACCGAGCAGTATGTCT
**CoV-bait-3251**	TTTGATAGCTCTTCTCAAATCCATTAGCGGCAAGGCCAAGACAAGGAGTGTATTGGCCAGCCTTAACATACTGAAAATTTTTCCCAGTGAAACTGTTCTGGTAACACTCAGACAACCAAA
**CoV-bait-3252**	TCCTGCCAAATCCAGAAGAAAACTGATAGTTGCCTCTGTAGCCTCAAAACAAGTGTCTAGAAGCTTACTGACAGCATTTTGACAGGAAGTAATAATAGTGGACATAAAATCGCCAGTTTT
**CoV-bait-3253**	ACAACATCTTTTATGCCACAGACATTGCACCACTCTCTCCAAACCATGCGTGCAGAACAGCATAACTCAGCCTTTGCAAGCACGGTATGAAGTAACCGAGAGGCATCATCTGGAGCACCA
**CoV-bait-3254**	GTAATTAAGCTTATCATATATGGGTACAGTGGCAAACAAAAATAAATTGTTTGAATTGAAACCATTTTTACGCGAAGTGTACAGGTGGACACCTTGAGCATCCTGCACGATGCCAAACCA
**CoV-bait-3255**	TGTAAGCTTTCTTAGGGTCAACAGTGAAGGTAACAAGAGACAACACTGATGAATTAGAAGCAAACTCAGTATTAGAACCAGCCTGCAATTTAACAGTGGCAGCAATATGACCTAAAAGCT
**CoV-bait-3256**	GAGTCGGTATGGTCTGAAACAGATAATTCCACAATGTAAACTTGGTGCATTGCAAGACTGCGACGTACGACAATACCAATGTAGCTAAAAGCCAGTGAAAGGTACCATACGTCACTTTCT
**CoV-bait-3257**	CGACTGCCAGTAGATATAGAAACAGGTTCTAGAAGTGTCAAATTAAAGTCACCTCCAAAACCTGGTATGATAGGAGATGATTGAGAGCTTTTCACGCTCGCAAACAAATTACGTACAGAA
**CoV-bait-3258**	GATACTGCTAACCGCCTGACAATGTCAATAAAATCAGAGCCACAATAGACACCAGGTCTATTAAGATGGTGGTCATTAAAAATGGCCCACGAGCCATTTGTGGTAATACAAACACCCTCT
**CoV-bait-3259**	GGCAGCAGCTTCGCTTCTCACCAGTTGCTGACTTACAAACGCATTCAACGATGTGAGACGTCCGTTAATAAGGCGGTCTATTTGCACTTCCTGTTCTAAAACATCCAAGCGTTTTAAGAT
**CoV-bait-3260**	TTTTTCATTATAGAGCTCTGCTCGATACTTACCACGCGCACTTTGCGTGGTCACACTAGCCTTGGAAAGCATAGTGTGCCCCGGGTTGCCACGCACCTGACGGAACAGGGTATTGTGACC
**CoV-bait-3261**	AACCTTGGCTTCTTCATCAGGAGTGTAAACAAAACAGCAAGTCCTAACATCATTACAAGTATCTAGCAACATCTTTAGCGACTCCTCTAAAGGAACACCGAAAATGCCACAACTCAATAA
**CoV-bait-3262**	TGCATCTGTTTGACACTCAACATCTCTAGCTAAAACATATGCTGGAGCTGTTGATTTGACATTAAGGCGAGTTGCTCTAGAAAGAGATTCAATTGCCTCAGTAGGTATAAATGTATCTGG
**CoV-bait-3263**	AATTGTGACAGCATCCAACAAACCGAGATGCAAGTATCCAAACCCATTGACATAAACATCACCATACTTGGTGATGACAATTTCCCTGACGGTTGGAGGTAAAACAGCCAAGAATTTATA
**CoV-bait-3264**	TTGCAATTCCAAAATAACATAAGACCATTCATTTGACCATGTACCATATAATCATACTCCAGACATCTAACATTGTTGTTAATAGGATCTCGATCGTAACAAAACCATGGTATTGGTGTT
**CoV-bait-3265**	GAAGTCTACGAAACTTCTGCTCAACTACGGCCCATGATTCAGTTTGGAAGACCTTGTAAAGTGCTGAAACTTCAGGTGTTTCAAAACACTTAAGGTACTCATCTTCATTTGAAACTACTA
**CoV-bait-3266**	GTTGCACTAGTGCATGGCGGTCCCTTGGACAGTATGGGGCATAGTTCCACCAACGCTCAGTTCAGTTTAACTCGAGTCGCACAGCAAAGGCTACAGCGTAGGGCTGTCTGATTCAATGTT
**CoV-bait-3267**	AAATTGCAAAGGTATGAGGATAACTCATCTTTGTGTTGGCGTTTGAGTTTCTGAAGGAGTGTGGTGTGCATGAATCCTGTAACTGGTTTGTTGGTGAGACGTGTGTGTTGTGTGTTCTGT
**CoV-bait-3268**	AGGCACGTAAGTAAAAGTCCCAGGATAAATTAATGGCAGCAAGTAAAACAACCGTAGGTATCAAAAACGTGTACAGGAACAGCAGCTTATGTTTAAGACCAACTGTCAAAACAACAGAAA
**CoV-bait-3269**	AATCAGCAGGGGATGGACCGTAATTAGGTAGCTTATAAGCACTCCAACGGCAAATACGCACTATAGCCCTATACTCACTATTCTTCTGAAAGAAATACATGCCCCAGGAATTATTAGACT
**CoV-bait-3270**	CAACAAAATTGTCTTTGATGGGAGTTGTCTGTTGTTCAACAGTTTCCTCTTCCTCGCTAGTAGTCTCCTCCATTTGTTGGACAGTTTCACTGCTTATTTCGGCAGGTTCACTTTCCTCTG

**CoV-bait-3271**	ACATGCTCAGCTCCTATAAGACAGCCAGCTTGTGTTTGAAACACATTTACTCCAGTGGAATAAACGCGCCAAGCAGGTGTTAATTGATCTGCACGTATTGCTGTAGGGACATCAGTGCAA
**CoV-bait-3272**	GAGAGAGCATTAGGTGTGTAAGCGCTAAGTTGTGAAACGGAAATGCAGTGTGGAGAAAGAACATGTTCATTAAGCGCAGCGATGCAAGATAGAGCGTGATCTGTTGTTTGAGAGATTTGA
**CoV-bait-3273**	CTTGAAGATGTTTATATGTTTCTGGAACGTCGGCTGAGAGGAAGGTGAGGAATTGCACAGTGAGGTGTACGAAGTAGTTGTAGATGGAGTTGGAAAAGGCAGTTGTTGAGTCGCCTGATG
**CoV-bait-3274**	ATGAGTTTTGTGTAATAATGTAAGAAACATTATTAAGTACAGTGCAACAACCAACTACAAAAACTCCATAAGACATGTCACCAAACATACGCTTAAACTTTGTAAACATAAAACAAAGTG
**CoV-bait-3275**	TACAACTTGCCTAACACTAGCAGCAAAGTCATTGTACATTGTCAGAAGAGTGTTTAAAATTTTCCTGTAGCGTTTTGCACATGTGCCAACAAAACCATTAAGTCCAAACTGAATCTTGAG
**CoV-bait-3276**	TATCCAAATACCATTATCACCGGACGAGGTACATTTAAACGCATGACTCGGTGAAAAATTAACGCCTTCATAGAATATTATTTGGTTCCCTTCACCCTGATAGTTGTAGAAGTCAGTGAA
**CoV-bait-3277**	CAAGCAACACAGTTAGGGTCAGTACAACCTAATAAAACATGTCTGAGAAACATAAGAGCCTTGTAGACAATGACGCTAACAATGAGCTCGTCACAGACCAAGTCAAAAGGTAAGACATTC
**CoV-bait-3278**	ATGGCATTAATGTTATCACAGAAACTACCATACTCAACTAACTGTGATTTACATGCTGCATAATCACCACAGACAAATGCAGCACAATCAATAGTAACTTTAGGAGCGCTTGTTTGAATA
**CoV-bait-3279**	CATTATCAATAACAAAAGTTGCTTTTGAAGCGCCTAAGAAAGATGAAAATTTTCCAGCTGTCTTAGAAACTACACGTACATAGCTGTAACATAAGTAACAAACAAACACAACAGCAATAG
**CoV-bait-3280**	ATATAGTCATAATCCAGACATCTAACATTGTTATTAATAGGATCACGATCATAACAAAACCATGGTATTGGTGTTGTAGCACAACGGATGCCTTTTGGATTACCCACATCGTGAATTGCA
**CoV-bait-3281**	GCAAGGAACAATGCCTATAGATGCATGCCATAGACTAGGTCGTCCATTACCCAAGACAGGATTGCAAGCCGTATTAGGGTATTCACACATAGTGTATTGGCATACAGACGCACTCAAGGT
**CoV-bait-3282**	CTACCCATATAGCCTTAAGACTAACAGAGTCTAAGACTGGGTAGTAAATTGGTGCTTTTGAATTTTCGTCATTATCAGGTGTCAAGTTATAAGTAAAACCATCTTTTGACTTGAAGTTGA
**CoV-bait-3283**	GTAAGAGCCAAATCAGCCATACGTTCAAGCTTACGAGCAACAGCTCTATCCCGCTCGTATGCAGACTTAGCAATATTACAGGCTTTCTCTAGCTGTTTAATCTGCTGTTGATTAGCAGAA
**CoV-bait-3284**	TCTATAACAGACACAGCATCAGTGACCTGCATTAAGTCTGCTATTTTAACAAACTTCTTTACAGTGGCATCGGCACTTAGAACATGAACAGGCTCCTTCACAACAACCTGGTCACCTTCC
**CoV-bait-3285**	TGGTGCATTTAAAGGCGTGATAAGGATTAAAATTAACACCCTCATAGAATATAATTTGTTGACCTTCACCTGTGTAATTATAAAAATCCGTGAAGTGGAAACTCCTAAACAAAGAGTTAC
**CoV-bait-3286**	ATAGAGTCGAAAACTGCGTAAATAACAGAGTATTCTCTTTGTGTTCCTTTGGCACCATACCAGTGGAGGTATGCTGGGGCTGTGAGTACGAGTGTGTCTGTACAGAGGAATGTTGTGAGG
**CoV-bait-3287**	GCATCATAACGATTGTGCTCGAAGGCTGTGCTATAACGCAGTCTAAGTCCAAATCCATCGAGTCGTCCATACTCTTGAAGCCTAAGGCAAACCTTCCTAGCGGCTTCTTGTTTTGCTAAT
**CoV-bait-3288**	CAACCATGACATCAGTAAACATAGTGGCAGCAACAGTCCAAACACCATATGACTCACCATTTGGTGGAATATTAATTTGAACTGATGCAAGTTGACCTGTATGAATATAACCATAGCCAT
**CoV-bait-3289**	CACCGTTCGAGACACATACGCTGAATTTTGTACAGTGTAGGCATGCTATAACCAGGGTTCCACTCAGCAGATTGGAGTTGTGGATAGAAGGTTTTGATTTGTGAATTTTCACACCACAAC
**CoV-bait-3290**	AACGCTACACAGGCTCCTGAGATTAATAATGCGGATCTCTACGATAATGGGAGATTTTCCCGACTGTTTCGTTCGCTTCTCAGTGGATAACAGCCAGCTTCTCTGTTTAACAGACAAAAA
**CoV-bait-3291**	AACAACACGACCACCATGAATAGTACCAACAATACACCCAGGTGAAGAGCCTGCATTATTAGATTCGCTAGAAATATTAACTACCGCATAAGCACCCCCGTGTAAATGCCAACCATTAGG
**CoV-bait-3292**	GCCAATTTAATTTCCCCTAATTTGTTCCCCACTCATTTGTAATCTATATGGCGCTTTCCCACTGGGCGACTGCTACACCTACAAAGTAGTACAGTCCTTTAACTTTCTCCATATAGTATT

**CoV-bait-3293**	GCAAAGCCACATAAGCCCTGAGATGCAAGCAAATGCGATGGCAAATCCAAAGGCCACCGGATTTATGGGATAAATAGCGGCAAACACAGCAGCAGCTATGGACAATGGCCATAGTAGCCA
**CoV-bait-3294**	GGAGTGGCCTTTAATTTACAAACCACAGCCGCGTTATCAGCTAGAGGTGAATAGGTTGGTGTTAACGCAATTTCATTGCGCCATCTTATATAAGCAGCATGAAGCATGTGCCCATTCACA
**CoV-bait-3295**	TGCTTATTAATATATAAAGCTGAACCATTAGGTCCAATAAGGTGCTTTTGTCTGTGCGTATCATAACGACACACCAAAGCATTAGCTGGATAAGTGTCAACATTACAATTCCAAAACATA
**CoV-bait-3296**	GGTAACATTGCTAGACGCCATATTTAGAGAGTTCGTTAAACCCAGTCCTCAGGGGGATAGGGTGGATGGCTCTCTTGGAACTTTACATAGGCTGCACGCCCTGTATTATAAATATGAACA
**CoV-bait-3297**	TGATCATTTTGAGGGCTATCAGCACATTTGGTTTTGCGACCGTCACCTCAGAGGGTAGACGTTGAAAGTCGCAAGCACCGAAATGCATGCCTGCCATCTTAAGGTAGCCATCAGTGACCA
**CoV-bait-3298**	TAGCGGCCAGGAAAGCAGGCACGATAGTGTCTGTAGTGATTATGGATGGGATACTGAAACAGAGGTTTCGATCGACCATCAACTTTCTCACCATCAATAGCTATTACCTGTAAGCAAACA
**CoV-bait-3299**	CACTCGAGATAGGTTGCTGAGTGCGCAAAGAAACAATTTGTACAGCGACAACGCTGGTTAAAATTTTTGACAAACCAGGGTTTGCTATAGTTAACAGGTATAACAACCCCGCCTGCAACA
**CoV-bait-3300**	AGTATGTTTGGATACTTGTTGGTAAATCGATTAGGAAGGGGAAGGAGAGTGTGTTGTCGAGATGGACCATGATTTTGCCTGTAGATGTTGGAGTGACAGACATTACCTTGGTTAAAATTT
**CoV-bait-3301**	ATATTACGATATGTAGAACCTGAAGTAGGGTAATAACCATTAAGCAACAACGTAGTATTTAAATACACACGATCTAAAACATAATAAGTACCTAAACCATTAGTAACATCGACAGTATCA
**CoV-bait-3302**	CCATCCGACACGGACAAACTCCATAATGTAGAGGTTGGCCGAGACACAGCGCGTGAACGCTGAGAACAAGACAAACCATTAAATAATAAAACACGCTCAAAATTGGCATGATAACCTACA
**CoV-bait-3303**	TTCAGGTACAAAAGCAATTAGCCATGATGCACAGAGTGACCCATCCGTGTGAGTATCCGGGTTCCATCATGAAGTATCTAAACGTAGCCGTTAGTGGAGGTAGTGTGGTTGTAGGGGTCT
**CoV-bait-3304**	CCTTAGTAACTGTGGAAGGTGTATCTTTAATAAAGGACAAGGTTGCGGCAACTTCCTCAACTTCAGACTCTACATTTGTTTCAGGAGCTACATTAGGACCATCATCAATTGGTGCATCAG
**CoV-bait-3305**	GTATGAATCATTAAGATTGTACAGAACTAATTGCGGTTGCAATGAATAACCATAACCGTCTACACAAATACCAGCATATGCAACAACAGGCTGGAATGTTTGGGGTACAAGAACAGCATG
**CoV-bait-3306**	GTACAAAGTTGCATAGCCCAACATGCCAAGTGTGTTCTGTGTTACAATGTAGGAAACATTGTTCAACAAAGTACAAGCACCGACAGTGAAAACGCCAACAGACATATCACCGAACATGCG
**CoV-bait-3307**	AACAGCGCCAACGTACCACTCGCTAGAAAGCGAACACAGAACCATAATAGCACAATACATAGGGTCACCACCAAGTAAGTAAGTGTAAGCGCTTACAACAAGAGAAGATAAGTAAGCAAT
**CoV-bait-3308**	TCTAGAATATCCAGGTAGTCTGGATTTTCTAGAGTCTCGACGTGTTGGTCAGCCTCAGGTTGTGGGGCTTGTGCAGGAGGAGGATCCAAGAGAAGGACACTCTCCTGTGGAACTTCTTCG
**CoV-bait-3309**	CTACTTTTACAGCACTTCCAGCAACTTGTGGTGGAATGAGCTGGTCATAGTCAGGCACAAGTGTTACATTTTCATCTGGTATGACTCCAAGTTGAGAAGCCACAATAGGTTTAGATACAA
**CoV-bait-3310**	CACAAGTGAAGGTGATAGACAACTCATCGGATCCAGCTTTAAACGCAGGTAAAGAAGCATATTCTCCATCACTAGGATAGTATGCTTTACCATCAGTAGCCATCTGCAGGACAGTGTCAC
**CoV-bait-3311**	GCATACAAAGGTTAATAAAAGAAGTTGATCTATAGCGCATGCACCTAAGAGTATAATTAAGCGCAAGTGCTAAATAGACACAAATGTATAGTCCTACAGTGTAAACTGATAGTCTTTCAG

**CoV-bait-3312**	ACAGCCTTCTATTTGAACAAAATCTGAACTAGTTGCAACTCTAGGCTGATACATAGTTCTGGGAGTCAAATAGAATTTGTCATCTAAATTGCGAAATAGCGTCAGCTGGACATCCTTAAC
**CoV-bait-3313**	ATATTACCTGTGACAAAAAATGGTTCAACTGCTGGTTTTGCAGTAATTAAGGGTTTTACAGTACCTTGGTCAACAGTTATAGATTTGTCCTCTGTGATCTCCTGATTGCGTGTTGCAACA
**CoV-bait-3314**	CAACTACGTGTGAGAATCGCATAGGTGGATCCAAGTTTAAGACAACCTTACCATTATCTGTATGACATGTAACAGTGGAAAGATTATCTTTATTAGAGGTAACGGCAACTAGAATCTTTT
**CoV-bait-3315**	CAGCCACCATAGAACTTTGTAGTACCAATGACGCAAGTCGCTCCACGAGTTGCAGCCATGGACTTAAGCATTTTCTGATGGTACTGGCGATTAGTCATTGTGCTAAGTATGGACACGCCT
**CoV-bait-3316**	TTTTTCTTATAGTGACTTCAGTAATTGAACCATCTGAACACACACCAATATTAGCATTAGTAAGTACTGGTTCAGAACAATTTTGTTGCACATTGGTGTGGTAATAAAATGTTTTCGTAG
**CoV-bait-3317**	GTAGGCACATAGCTGAAGTGTATAAAATATAAGCCATAAGGCGCACTCTGGACTAATGACAATATATGATTGCCATTGCCACAGAAATTAATCCTAGGTGATTGGCTTTTAACACACTCA
**CoV-bait-3318**	GCTTTACCAGCGTAAGGTGACAAGGAACGCCCGCTCCGGTTTAGTCATCTCGAATGGAAATGCTTATGGTTACTAATGTTGGCAGCCTGGTTTGAGAGCTCGCCTAAATCCGCGGCCAAT
**CoV-bait-3319**	TATCTTGCATTATAAAAGTGAAATGGTAGTGACCAGTTTGATGTTTTCTGGCAGGCACAGATGTATTCCCCATCTTTGGTGATTGATGGTGGTAGATATCCGCATGGTTGTCCACAGGGT
**CoV-bait-3320**	GACAATATAGACAAACAGATGCACCACCATAGGAATCCTGGTTTGAAGTAGCTTCTACACCATTTGTAATGGCCATGCCACTACCAGATCCGTTAGCAAGCATTTTAACACAGTTACCAA
**CoV-bait-3321**	GTGACAACGCCATAATTGTGGAATTTCTCCAAAATATATAATTGGCATGCATTATATTTCCATCCACTATAGCCTTGTCACAGTATGGTCCTAAGTAGTTGATACCTATCAGAAAACCTT
**CoV-bait-3322**	GCTGGCAGGCGGGTGTACTACTGCTATGCTTTAGCAGACTTTGCGTCTACAACCCTCCGAGATCATCTCAAAGTCCCAAGCCCTTAGAGGCACCTATACTCCTCCAAGGGCTCGAGGGTG
**CoV-bait-3323**	AATAGGACACGCTATAGACTCCAGATGGGACATCAAATGTGCCATAGGAGCATAGTAGCTGTGTATAATCATTATAACCACAATCAGCAGCCTTATCAATGAAGCCATTAACATTGAAGC
**CoV-bait-3324**	GCAAATGGTATATGTGCAAACAGAAGCCATTATAATATTATTATATGGCTCTAAAACTACGGTATAGGAAGTATGGCCAAACAAACTACCTATAACTATAGTAGGAAAATATGAGGTTGC
**CoV-bait-3325**	AGTCCTAAACCTAATAACAAACAAATTTGTCTTACATTTTATACAGTCACTCTGGTCTAACCTACAAAACCCTTTACCACTAATACAAAATTCCAAACTACAATATTCAAAATCACAATT
**CoV-bait-3326**	GGTTTTTGAAACATCACCATACACAACGTGCTCAAAGCCGTAATCTTCAAGTCCATACTTGTTAATAAACAAACCCATATCCATACTTAGGAAGTCCTTTTCCATGTCGCTACGAGGGCT
**CoV-bait-3327**	TCATTTAACTTCTTAATTGCATCCTGAATCCTGTTCATCTCATAAGTCAGGTCCAGGAAAGTAACATTTAACTTCTCGAAATCGAGGGATAAATCAGGCGCAATAGACGTCTGATTCTTA
**CoV-bait-3328**	GGTTCCTTCCAAATTATGGATGCTGAACGTGCTGAATGTGATCTTGTAACAATTTCAACAGAAACATGTGTTGGTTTTAAATCATCATACTCAAAAAGATGTTTTGCTGTTACAATAGTA
**CoV-bait-3329**	CACCCTCATTGTTATACAAAGCCTTACCATCACAATGAACACCTTCACCTTCAGCACGCACAGCTTTTTGTTTAAGTTTGCCAGGCATGATCTCATTATTTTGCAACTTGACCATTCGCT
**CoV-bait-3330**	AAGAGAACTTCTTCGTCCTCAACATCATCTGCTGTAAAAACTGTCTTGTTCTTTGCAAAAACACCTCCGAGGTGACCCAATGTGTCACCTGGTTTCAAAACAAGAGTTTTAAAAGACGTG
**CoV-bait-3331**	TTACCACGCGCACCTTGCGTGGTCACACTAGCCTTGGAAAGCATAGTGTGCCCCGGATTGCCACGCACCTGACGGAACAGGGTATTGTGACCGAGAAGTACAAGAGACACGATAGCTCAG
**CoV-bait-3332**	GGGAAAAATACACACAAGCATTCTTAACTTGTGCGACATTTGAACCACTATTATTGTAAACAATAAAGTCAGTCGTAACATTACAGTTCTTCAAAGCATCTTTACAAGAATACTTACTCT
**CoV-bait-3333**	TGCAGACAAGTCTAAAATAACTACTATTTCTATAGACATGCCTGTAAAACCAAAGGTTATTTTCATAATGATAAGATAGCTTGCTCCATGCATCATCATCATGCTGGGGAAAATCTTCTC
**CoV-bait-3334**	CTTCAAAGATAACCTTACTATCACAATTGCCACGATCGTGTGCCGCGTTAGCAATGGAATTCTGGTCATCTAATCCGACAGACCGGACTGCCGAGGAACTATCCTGCTGAGGTTGAACAT
**CoV-bait-3335**	ATCATCAACCCAAAATAGGTTTGGAGATGATGATAAAAGCTATTGATAACGGCTGTGTCTGAAGAATCACCTCTATATATATCCTCATAAAGAGCACGATGCAAACATGCAATATCATTA

**CoV-bait-3336**	TGGGCCTCGTTGTAGGCAGATTGTCCCTCATGAGCAATGTCTCCAGGACCAAAATTAACAATGGTCTTGTAACACTGTCTTGAGTTCGGTTTCGCCGCTTTTAGCTTATTATCGTAAACC
**CoV-bait-3337**	AGGAACAAAACAGAGAAGAGAGAGAATAGTGCGAAAACCACTATCGGTCTCGTGACGAGGAAGGGAGCTCAGCGAACCCCAGGCCGTGTGGTGGCTCTGGTAACTGAGTAATCCCGCCCT
**CoV-bait-3338**	CGGACGAAATGCGAACCCTCAGACCCGCAAATTTCCGTTCAAGTTTGGCCCATGACTCAGTGCGAAAAAGCTTATAAAGCTCTTTCACGTCAGGGTCATTAAAACAATTGAGGTATTCCT
**CoV-bait-3339**	AGTGCATAGGAAGATTGTGACTAATATACAGGCGAAAGCAGCTATGGCTAAAACAATAAGCAGCCACACCCACCAGGGCCATTTAATGTATGTTTCAACCTTATTAAGCCACTCTAAATC
**CoV-bait-3340**	GCAACACATCCGAATACAGCTTGTGAGGTACATCTACGGCGTCATAGCAATACAAAGTTCTATTGCCATCACGCTGTAAGTAGGTGCATTTAGCATTAAACAATGCAGGTGACCGTAAGT
**CoV-bait-3341**	TGCTCAAGTCATAGTTAGTGCACATGCTGACCTCGTCCACAACCAGAATATCAGCTGTTGTTTCTGGAAGTGCATTTATAGTAGAAAACACGTATTGAGAGCCTACATCGTTCACCTTGA
**CoV-bait-3342**	TCAGTCATAGTAGCATATTCATTATAAAAATAATAGGTATCTTGGCACCAATCCGTAACTAACTTCAAAGTTGTCATATCTGAAAATTCTTGCTGTCTCTCAATACCACAAAATTCATGA
**CoV-bait-3343**	GGGAGCAGATCTATCACGGCGCTTAGGCTGATTTTGCACAGGTCTGTCATTTGCAGACTGAGATCTAGGTCCATTACCTCTCTCCATGGTTCTTCCACGAGAGTTGAATGGGTCAACTCG
**CoV-bait-3344**	TTCCTTTAGCATTTGCAGGGATAGGCTTGCAGGCTACGGGTTGCATTTTACCGTCCTTCTCAACAGCCGTTGCCGAAGGATAAAGGTAAAAGTTTAAGAACCGCATAGCCGCATTAGCTG
**CoV-bait-3345**	AGGAAGCGCCGTTCTTGAGTTCGAGTTCGAAAAGACTGACCGGTCAGCGCCCTTTATTGCAACGTTCTGCCGTTGCGTAAATCATCTAGCCCGAATGGAAGCTGTGCACAGAGAGTTAGT
**CoV-bait-3346**	CTGTCGTTGCTGTCAGATAGGCACTAAGAGGGGGACATGCAGGACGCCCAAAAAGTGGTTTAGTAATTATAGACAATAAATCATCTACAACTTGTTGTAACACAGTCCACCTTATTGGTC
**CoV-bait-3347**	TCTAATTGAAAACTAGGATAAGCAACATCATAGGTGCAATTTCTAGCATGTTGATAAACCCATGATTTGTAATGCTGATTACGAGACACCGTAAACATGGGCTCAGTACATAAAACAAAA
**CoV-bait-3348**	TGTTCTGGACGGTAGACACAGCAATGTTGCGATTACCACCCAGGCCTAATAGTCTAATGTTGGCAATTAGCACCTCGAACGCATTGCGTGGAGGAGACATTTTAACTGCCATCATGTACT
**CoV-bait-3349**	ACAAACAGTTGCACATTACATATACTTATACAGCAGGGCGTCCCCTGGCTTTGAAGTGGTTATGGTGTGAAGTCCAGCGTTTGAAGCGCGGTCACCCTGGTAGATCAGAACTGCGAACTC
**CoV-bait-3350**	TGCTATGGCTGTGTCTTGAGAGATCCTTCCCTTGGCTACTGTTGCCTGGGGCTTTTTCCCGAGGAGGCAGCACCTCCTTGGGGCCTTTTCCTAGGACTGACTGCCCCTGTCCTTTGTCCT
**CoV-bait-3351**	GCTGACGGGCGGTGCAGACCGGTGTGACTTGAACCCTACGGTGGTCAGTGCTGTCTATAGAGGAGGTAAACGGCCAGAGGAACTGAAAGTAGCTGAAATTTTGTGCAAAAGCCACAAGCG
**CoV-bait-3352**	ATGGAATGCCTGGGTAATATTGCCCATAGCATTATTGAATGATGTAGCCAAGATTTGTTGGTTGCGTTGCAGCACATCCGTTTGAAGAGCAACATAATTCAAACGCGATTGGACTGCTAG
**CoV-bait-3353**	AGGTATGACATCATTATGGCTCTCCCACTTGACATACTTAAGGTTGTTTTCTGCGGCTATGAATGCATACATAAAGTTTCTACCGCTTTCATCTGCCATAAGAGCTTTTCCACTGCCAAA
**CoV-bait-3354**	GTACACCATTCAAATAAATGGTGCCATAACGTGAGACAACAATTTCTTTAACAAGAGGTGGCAAAACACCCACAAACTCTGACACTGTGCCATTAGTGCTATTATAGGATACAAAACACC
**CoV-bait-3355**	GGTTCAGATGTAGGTAAAAACCTAAACACACAACCACTAAGCTCTGATTGGCGCCTTTCACCGTTGTTACAACCACACACAACATCAAATGTTATAAACATTGTGTGCAAGTCTTTAAGA
**CoV-bait-3356**	ACCACACCAGAAGGTTGCGCCATCTTACGCAAACCGGATTGCAATGTTGAATTGTAACTAATGGTTGGTGGTGTGTACAACGTATCTTGGTGATTGGTTCCATAGTCTAAAACAGCTTTA
**CoV-bait-3357**	TAACATTGTTAAACCAATTATTATTGATATTCAAATAATGAGAATCATCACTAATATAGGCTGTAACATAGTCATCATTCCACTCAAGACCAAATAATTTCGTACCATTATCTGTGGGTA
**CoV-bait-3358**	TCTTTACTAGTGTTGTGGATAGTTTAGGTCCACACACTGTTGCAGGTGCATTAAGAAGCTCAAATGAAAGGACAACTACCCTAGTAGCCTGATAGTCAAGTGGCACACTAGGGTAAAAGT

**CoV-bait-3359**	AAATGCGAATATAGCCTGAAGGAATGAAACCTGTTAAAGGACACTCACCTGCCCCACTCTTATAACAATGTGTAACAAACACTGTAAAATCAGTAAAATTACAGTGGGCCGTACAAAATT
**CoV-bait-3360**	TGCGGGCGCTTTTTAGGGGCACGGCGACGAGGAGGAAAGGAAGCGGGCATAGACATAGGCATCATTGGTGGTCCGTAGAATGCCATTGTAATTTGTTTTCGCTAGTGTTTTAAACAGATT
**CoV-bait-3361**	TGATAACGTCTAACTCGTCTAATTAATTCTGTACTTTTACCTGAAAACATGGGGCCGATTATCAACTGAATATGTCCGCCGTTCATGATGACAATAAAGAATTAATTATTGTTCACTTTA
**CoV-bait-3362**	AACCCAAGTGGCCCCCGTGGAGGGAGAGCGTGCACAATACACGTGCCCGGGAGAACTTGTCCGATTCGGAGCTGAAAGACCTCACGGACTTCGAGAGGGGACCCTTTCAGAGACGTGAGC
**CoV-bait-3363**	CAGTACCAAGTCCAAGTCCCAAAAGCGCAGCCTTTACGGCAGCAACAATTGCGTCCTGGTCTCTTGTACCTGAGGAACTACGGGAGCGGTTGTCACCACCTCTGTTCTTAGAACTGTTCC
**CoV-bait-3364**	AGCCATAAAATGCCATTGACAAGAAGTCCATGATCATCAACTAGCTGAAGCATATTCAAGTTCGTCGAGTTGAACATCTGTAAAATTAGAATTTACAATGCCGACAACTTGCTGCCGAGT
**CoV-bait-3365**	GCGTATGAAAAGCATGGTTGTTAACATAAAGAGAACCACCATTACAGCCTTCTAAATTTAACAGGGAACGTTGTCTAGTATCAAAGCGACAAACAATTGAAAACTCTGGATACATATCCA
**CoV-bait-3366**	TATAAAAGAAGTAGGTGCACTAGAGATTGGTGGCCCAACATTAACTAAGATTACAGTACCATTAATACTGACAACCTTATTTATAAAAGTAGAACCATGTTTACACGCACCATCATCAAC
**CoV-bait-3367**	TGCTTGGCCAAACTACGAGAAGCCTGCACTTGAGAATACTTTGTCAAAGTTTGTGAAACAAAGGCATTAAGAGCAGCCAATCTGCCATTAATTAACCTATCTACCTGAGCATCCGCAGTA
**CoV-bait-3368**	GGCATCAGTGGTGGGTACCGCTGGAGTTGCCATTATTGGTTTTGGTGTCAAACTTTAATTCTCTGTTTGTGGTTTTAAAACACACACTACAGCTTAAATGTTAGCACACCTACCCTTAAG
**CoV-bait-3369**	AAGGTTAGTTAGCAAACTTTTAAAGTAGGTGTCTAGCACTTTGGTGGTGTCACAAAAATCAAGACTTGGTTTGGTAAAAACAACAAACCTTGTCACACCATTGTCAACCAATGCTTTAGG
**CoV-bait-3370**	TAGTGGAACCTATGAACACTATGTTTCCTGCAGCACTATCATCACAATCTACTGCAGCAGGCCCATCCGTCGCTAGAAGAAGCAAATATGGGAGAGAAGCTTCTACAACTTCATCTACAC
**CoV-bait-3371**	ACTTCAAATCCTCATAACACGCCTGTTCATGGGCGTAATTAGAGGGAGTCGTCTGTTTCACAACAAAATAAGAGTCCTTGTATTCAAGACCGCGTTTCCAGCTATCTTCGCCTTCAAACA
**CoV-bait-3372**	TATAAACATCTTGCTGAAAACAAAGCTATAGATGTATAGAAACTACCTTCAGTAACAATTGAATCCTGAGTGTAACCAACAATTTCCCTATTAAACCAAGTAGGGACATACCAAGGTCTT
**CoV-bait-3373**	GAGTAACGTGGTGGAGTATACACAATTTCCACACCACTATTTCTATATTGGTCCAAAGCATAAGCTAACCATGCACGACAGGCTTGCAAGTAATCTTGCTCACTGCCTGTACCTGAATAG
**CoV-bait-3374**	CCAACTTAGAAGTGGCTTTTAAGACTTTTGCTGGGACGCCAGGTGTTATTTTGTCAACTCCCTTCAAGAGGAAGAGACCACACACAGTTTCAAATTTGAACTGCTTCCCAGTCTGCAGAC
**CoV-bait-3375**	ATATAAAAGAAATTAACTACACCCTTTTCTATATTAAAACCAACTGAGCCACAAGCTCCCGCAAGGAAAGAAGCTCTAATAGTACCATTAGAACGCATAGTAACAGGGTAGAGTCCCACA
**CoV-bait-3376**	CACTTGACCCAAGTTTCTGGGTCTGGTATTACAAGAGTAAGCTTATTACTACAAACAATTGGAACAGTAGCTAGAGGTACAACACCACTATTAGCCTGATCAAATAAGACATTAAGCTTT
**CoV-bait-3377**	CACAGCACCTCCGATGTTGCATTTTGTGATACAATCTTTAGTAGCTAGTGACACAAGATCCTGTGCAACTCCATCCACTTGAATGGTTTCGCAAGGAGATGAGTCATAGAAAAAGAATGG
**CoV-bait-3378**	TCATAGAGTACCTGGTCAAGTATAAGTATTGGTTTACACAGATATTGCGCGTAATAGATGGAAGCATTCTTTGCGTCCTCTAATGCATGCGCACTCTGTGTATTACACACTATAAAACCA
**CoV-bait-3379**	AAAGACAAGCATGGCTGCTAGGAACTAGGTTGAGCATTGCTGTAACACGCCCATCCTTAATACCTTCCTCATTCATCTTGTCATCACCAAAATTTCCCTCCTTACCTTTAGTACGGGGAC
**CoV-bait-3380**	ATGTCAGTTTAACTACATCTCCTGCTGTAATGGCTCGTGGGCTATACATATCACGTCCTGTGATATAGTAGGTACCATTTACTTCTATAAAAATGCCTCTGCCTACTACAGGCACAAGTC
**CoV-bait-3381**	AATATAATAAAATTGTCTCTATATTGGACATTCCATTTCTGCGCAAGAGTTTGTAAATATATCACATACTTTTGCGCATCTAAATGATAATAATCAAGAACAGTCTTGTCAGTAGTAGGA
**CoV-bait-3382**	CTCCCCACGCTGCTTGCGCCCGTGGCCCAGTTCTGCCGACGAGTTGTCAGTCCCGACTCACAAAGAGTGGGTGCTCAAAGTCTGGTTCAAAAACAATCCTCCCATCAGGACCACGACGCA

**CoV-bait-3383**	TTAAAGTGGTATTAAAGGTGTAACTGACAGGAAGAGCCGGAACACCGTTAATATACCAAGTACCATTACGCAAAAACATAAGAGACTGAATAGTGCCATAAACTAACAGCTGTTTAGCTG
**CoV-bait-3384**	GAGCGACGTGTCTTGGTACACGTATATCTGGCGAAAACAACATGGTACCAAGGACTACGATTATTCACCATTACGGAACGCGTGCTTAATTGCTACATTTTGGCCGTTCGCATAGGTTGG
**CoV-bait-3385**	TGATGTTAACATGTTCAACAGTGCAACTAGTGATCTCAACGAAATCACCAGGAGTGGGCACACGAGGTTGAAACATGGTACGAGGTGTGAGCATATAGTACGTGTCATTGCCATGGAATA
**CoV-bait-3386**	CATCCTCACAAGGCGCTTCAGTCGTAGTCTTTGCGTCCACTAATGCAGCGAACGGAACCGGTGCATCCCCTGAGGGGTACGGACCTCCAACTACTTCTTCCACGTTGTGGTTCTTCTCTT
**CoV-bait-3387**	GCGAATGGCCATGCGAACTATATGAGATTGTCGGACCGGGACGGGTCAACTCGTCCTGAGACGATGCGACCTGCTTATTAGCCATCTAACTCCAACTCTCACGGTATCTCCTGTGCGGCG
**CoV-bait-3388**	AAACAGAAAGGGCATAATCATAATCTATAGTGCTAGTAGTACTAGACGCTATAACATGGCGTGGGCACATAACAGTATCACCAAGCCATAGACCATTGAGAGCCATATTACCATAGCAAA
**CoV-bait-3389**	ATCTTCGCAAATGCCAATTTTACCATAAATAAGTGCTGGTTCTGTGCAATTAGTAGCATTTTCAGCATTAGAATGTAGGTAAAATTTAGGCAATTCAAGAGAATTGTTAAAGCCAAAAGT
**CoV-bait-3390**	CATATAAGAGGGTTTGCCAGCTATTGTAATAGTCAGCCTTAACCTCCTTAAAAACACCTTGACCAGTAATACCGTAGAGGTCATATCTGACACACACGCCAGTAACCACTTCAGTATTAG
**CoV-bait-3391**	TGGCATCAGTCTGGCACTCAACATCTCTAGCAAGTAGATAAGCTGGTGCTGTAGGTTTGACACTGAGCTTAGTAACACGAGAAAGTGATTCTATTGCTTCAGTGGGAATGAATGTGTCTG
**CoV-bait-3392**	GTTCCAGTATAGTAGAAATACCATCTGGGAGCCAGTTGCTTAACTCCATTTCCTGTGTTAACCTTTCTGTCCTGTCTCCGCCAATACCCAGCATTTTGCGCAGGGGTAGAATTGGCATTA
**CoV-bait-3393**	GTAAAATCCGAGAAGTTACAGTGAGCCGTGCAGAATTGCCCAGTTGACCACGTCATCCCTAACTCAGGTGCAGTCATGGCTACAGCAGAAGCAGAGAAATTTTTACTCCAAGAAATAGCC
**CoV-bait-3394**	ACGGCGTTACACATAACACCATAGTCGTCAGCAAACCAAGGCTCTAAACAGTGTCTCACACAAAAAGCCTCCCATTTGTCAGCTACAGGTTTCATGTAAAAGGGCGTGCAAAGCATGTAC
**CoV-bait-3395**	TATGGTACTCAGAAACTGGTTGACCACCATAAGGTTTAAGCCAATTATGAACTGAGTGTAAGTGATGACCAGAACGTAGATACCACATGTTCGGGTCAGAAAATGTAACCCTGATATAAT
**CoV-bait-3396**	AACACTCTCTTCCTTAGCCTTAATGGTTTCAGCAGCAAATAAACGCAAAGAATCTTTAACGTCATTTGCTAACTTATAATCTTTGACATCTGTCCAATCAGAGGTGGCTAACGTATTAAA
**CoV-bait-3397**	CGTTGCAGGCACGAAAACAGTGGAAACATTGCCGTTTAAGGAGAAGTCAGTTACAGAAACTAGGCTAATCTTACTAAAACGGCTAATATAAGTGCACTCTATATAAGTGGAAGAGTGCAA
**CoV-bait-3398**	ACCTATAGCAAGGTAAGATTTCCAAGCATAAGGAAAAGCAAACCAATCGACTGTTAGAGAAGAATAGCACCCAGTTGCCAGAGCTTCGGGTGTAATCTTATCACATACAAACTCATCCAC
**CoV-bait-3399**	GAGCTCCAAACAGTTGTTCAAACTCAGTAAGTGATGTCTTGGGGTTTAAAACCGGAACACCAGGAATTGCTTTCGTGGTTATCAGGTTATAATCACCCAAGTGTGTCAAATCTTTAGGAT
**CoV-bait-3400**	CGTAGTGACCTACGGATGTTTCAGAGCCCAGAAAGACATTAAAGGCCTCAAAATCAGGACTAGTCGCAGTTGAAACGATCTGCTCACTAGGTTTGCCTGAAAGAAGCAGCCAAGGTGTGT
**CoV-bait-3401**	ACCAGAATCCATGTCATAAGGAAAAACTCTGTACAACACGCTGTGAGCATAATAAGTCAGAGTATCCTTAACAGAGTTGCAAACCTTGTACGGTATGGTTGCAACTTTCTTAAAGGACTC
**CoV-bait-3402**	GTAGAAGGCAACACTATAGCCATAGAGCTGCTGAGAGATTGAGGATCCAAACACGTACTGGTAAAAACTACGCAGATAGGTGTTAGGAATCCAGAAGGTATTAGCGCTAACAACCATCAT
**CoV-bait-3403**	GTCCTTGTTTGAGGTTACGGCTACGAGAATCTTCCTACCAGTCTTGGTAATATAGAAAGATTTCTCAGTTGTTTCAGTACCGTTTATATCCGCAGCTGTTGTCTGCGCTGTGAAAACATT

**CoV-bait-3404**	ATTTGCACAATGCAAAATACAACGGTCATCAGGACAACTCTGACAATTGGGATGGTATGGCATATCCCAGTACTTAAAGTACTTTTGGTGCAAGTTGAGTCTATGCTCTGTATAATCATA
**CoV-bait-3405**	CCATAATAGGTTTTGGTGTCAAACTCGTAATTATATATGCATAATTATATATATATATGCAAACAATAATCTATGTCTAGCACTCACACCCTGTATCCACTAGCCCCCGTTAGGACAAGT
**CoV-bait-3406**	GGCAGAAATGCGGACCTTAAGGCCAGCAAATTTGCGCTCTAATCTTGCCCAAGACTCAGTGCGAAAGAGCTTGTAGAGCTCAGCCACAGTTGGGTCATTAAAGCAGTTGAGGTACTCTTC
**CoV-bait-3407**	CCATCAATTGGCACAAAAACATTCTTAGCATTGTTATTGGACGTGTTAAATGGTGGTAAGGTAACACCATGAGGGGCATCGTGGGCCCTGGCAGTGCTAAGGAGTGCGAATACTAAAACT
**CoV-bait-3408**	ATCCAAGAAGGAGGAAAATTTACCGCCGGTCTTGGTAACTGCATAGACATAATTGTAGAAACAGTAGAGTATGCTACAAACAACCACAGCAAGCAACTGCATGTTGCTAATGTGGGGCAG
**CoV-bait-3409**	TGAGACACAGATGCCAGGATTAGTGTTAAAGCAGTGCCCCATACGACAGTAAGAATTACTGACGTAGCGGACAACATGAGGGTAGTAGATGATCTGCTCCGGTATCTTAAAGGGAACAAC
**CoV-bait-3410**	CGTCCCTCCTGCCGAAGCAAGTGGTGACATCTTCAGACTTGTTTGGTACACGCTTCCATCTGGGCTTGTCAACCTGCCTCTTAGAGTCTGATTGATTGGAGGTTTTGCCAGGGGACTTTG
**CoV-bait-3411**	GAGCTTCCTCGCGAGTTGAGCCAACGAACACTTTAAGCACAGCCTCAACGGATGTCCCGGATGCTATTTGATGGAGTGCTGTTGCATACAATGACTTAACCTTATCCATAGCGATGTTAT
**CoV-bait-3412**	TCTTAAAATCATCTTGAACTAAAACAACTGAAGCCCTACCTACCTTAAACTCATAATATTGATTAAATGGTAACTCCAAATCATCAGAACTATCATCTTGCAGGTGAGTTGACAGGTCCC
**CoV-bait-3413**	ATCACCTTTAACATGCTTAACACGCAACACATAGTCTACACCATGTGCACAACAAACGCCATCATCAGAACCGTCTCTACTCAAACTAGCAGAAACGACACTCGCATTTATGGTGCGTTC
**CoV-bait-3414**	AGACCATTATCATTCACTAATTGTAGCATACTCGTCTAGTTGAATTGAGTCAAATGCAGCATTAGTAATGCCAACAATTTGATGTTGCGAAAAGACATAAAGCTTCTTGCCATCAAGAAG
**CoV-bait-3415**	CATCCGAGAAATTACAGTGTGCCGTACAAATTTGTGCCTTGGACCACGACATACCTAATTGCGGTGCTATCATAGAAATAGAAGCCGCTGTAGAATTAAACACATCCTTAATAACACCAA
**CoV-bait-3416**	CGCTCAAATTTTTTGCTACTTGATACAAATGGAACATTTGTGAAGGGGTAATACAGTTGGGTGAAAGAACACCAGATTGTAAAGAATGTATACATGTTATATACATATCTTGTGTCTTAT
**CoV-bait-3417**	GCTTAAAAAGAATGCCAACAATGCTAACAGCATTTCATGTGCCTTCTCAGGGTCATCACAAAGATTGATTTTATTATGTAATGTTACACAATACTCCCAATCTTTGGAATTGCTAGCAAT
**CoV-bait-3418**	ATGAAATGGAGATATAAGATCTTTAGGACTTTGAAGTTCTTCTACACATTCTTGCTTAGCTGGTACAGGTGGCTCAACTACAATTTTATAGTCAGTCTTTACATTGGCATTAAGAAACAC
**CoV-bait-3419**	GTGTGTACTGTAAGTTGTAAAATGGCACCAACCATTTTAATTGATTGAACAGTTAGCTGAATGCCTTGAGCTGGGCAAGTAATTGCAAAGTCACGACAATCAGCTATAGAAACCATATGC
**CoV-bait-3420**	ATGGTAGCCTTTAAGTAAGCAATCATGAGTGCCACAAACCGTTGAGGTTTGCCAGCACGCATTTCAAAAGCCGCTTCACGCACAGCAGGTGTCTCGTATTCGACATTGAGCTGCTGTAGC
**CoV-bait-3421**	AGTGTAAGCGTGGATTGGGCGGACCATCGCTCGCCCCCCCAGATGTACACTATGAGTAGACATCTGACGTACTGGCTACGAAACAGGCCGAACGACCCCGCACGGATCGCTATTCACACC
**CoV-bait-3422**	TTCCTCTCTGGTAGGAACACGCTTCCAACGAGGTTTCTTCAACTGAGAAGGCTTATCAGCCCTGGGTTGAGAAAGAGGCTTATTAGGTTTCTTAGGAGTGGAAGTACCAGAAGAACTAGG
**CoV-bait-3423**	TGGCCTACCATTGTGTGTAAGACTAAACCCTTATCATGCGCTAAACGCATGGGGTTGCAACTAATAATAATAAACATTCAACTGGAATTACAAAACAATTTAATGCAAAACCTCGTTGAC
**CoV-bait-3424**	CACTATTAGTAGTAACAACACCAAACTTAGCCTCATGCCATTGATTAAAATTATCAAAAACATTACGAACACAGTGTAAAGGTGCTTCAAACACCTTCAACTGACCATTCTCAATGTACT
**CoV-bait-3425**	ATGAACTTTAGGAGGCAAATCAACACGTTGCCCCCTGCGCATACGCCAACGCTCTTGAACATTCCAATAACCAATCTGCTCATCTTTATTACCCTTACCAATAGGGACAAGATTCCTGGG

**CoV-bait-3426**	CTACATAAACCATCGTAACCCACAAGGGGATAACGCTGGTAAACATAACCAAGAATGAAACATGCATAATAGCAGCCACATCGCTACTAAAATAGCATGTCACATACAAAAACAACAAGG
**CoV-bait-3427**	TTTTCATTATGATTACAACATGTCACATGTTTAGAACCTAACACCATAGCACTAACCATTCTTAACATATTAGGTAAACTACGATCACATTTAGGATAATCCCAACCCATCAAACAAGGA
**CoV-bait-3428**	CCTCAGTAGTATTTTTCAAATACTCAGAGCGCATCTCAAGGTCAGCAATCTCACCAGTTAAATTAAGATAGGTGGCATTAAAAACATCTAGAGGAAAATTAGGTTCTGTCCTATTAGGAA
**CoV-bait-3429**	TATGCTTCGTCTGTGTGGTTTATGGGTCTTTTAAACTGAGCACTCAAATCTAAGGCTACTGGCCTCGAAATGAAGGTGCTATCAACAGTGTATGTGTCGCAATTGACACAGTTCCAATTA
**CoV-bait-3430**	CGTAGGCCGTGAAGCTAGTAATGAATTTTTGGTGGCCTGATTTTTCGCAACTTTTAAGAGTAAACTGCGCTGCAAATTTACCCTGGTTGCTACACCAAGCCTTTAAAGGAACTCTACAAT
**CoV-bait-3431**	CAGTCAAATCCAGCGCTTGCTTAACTTTGGGGCGGTGTCTCTCCCGCGGAAGGCGATAGTCAGGCAGACGTGGTAAGAAATAACCAGCTTGACTTTCAGTACACTCTCCGACTCGGCAAT
**CoV-bait-3432**	ACCAGCTCTCACCTGACAACACAGCTGCATACAACCACGCCAATACGTTGACTGTGATTGTTACATCTGGAGACGCCAGCTGAGGAACTTGCTTGTCTTCAAAAGGGCCATAAAAGGCAC
**CoV-bait-3433**	GTGCAAACTTGGTTGGAAAAGGTTTACGTGTCATAGACCAAACTTCAGCCAAAACAAACAAGGCAGACTGATTATCTACCTTATTAGGACACGGATAATCTCCAAAATCATAAAGTTGTC
**CoV-bait-3434**	TTGAGGTACTGGGACACAAAGTACATAAGAAAGCATCTAACATAATTGTTACCAAAGACAGCCAAAAATACAAAATAAGACAATTGTACCAGTTTATTCCAGAGTGGATCTGACTTGAAA
**CoV-bait-3435**	ACAGTTTCATAAGCTGTTGGTAATAGTACTGTATGAAAGAAAATCATGCCATTTGGTGCTGCATTTGCTAGTGAAAACAAATGTGTACCATTACCACAGAATCCGAATCTCTGAGACTGA
**CoV-bait-3436**	AGCTACTTCAAAATGTATGTCAGCAAGTGATGCTAGCTCAGGAGCCTTAGCCAAAATGATATCAGATTTCTTCTCAGTGTCGTTCAACACAACATGTAACGTGACATTCATCCAGTTGTA
**CoV-bait-3437**	CGTTCTAACATAATAACATTGTCTGTTTTAACTATGTCATCAACAAACACACCAGCTGACAAAATTCTGGACGGGTCTGGATAGGGAAGATAGTAGTCTCCATCAGGCCCAACAATCTGC
**CoV-bait-3438**	TGCTACTCCAGCTATTACTGCAACTGTCAGGGGACATGTGATAGAATTATCGTAAACACCACCAACATGCTCATGATACCATTGAGTAAAAGACCGGTACTTATTAGCAAAGCACTTATC
**CoV-bait-3439**	AGCTGACCATGAGTCATATAATCATACTCTAACAGCTTGACGTTACTATTAACAGGTTGCTTATCATAACAATACCACCTGGCATCTGTAACAGCACAGCGTATACCTTTAGGATTGCCA
**CoV-bait-3440**	CTATTCACTCTATAGTAGGTGATGTCATAAACAGGGTTAAACCACCACAAGTCTACTAACGTTCCAGCGGTTTCAAGAGTTGTTGCACGCATATCACCAAGTGTGACAGTGCCAGACCAT
**CoV-bait-3441**	TGACGCGCATTTTCATAAGCAATAAAAGACGGCATATTAACAAATGTGGATGCCACACTTTGCAGAATAGAATTGTCTGCAAAGTAAGAATCCAGCAAATCATCAAAGCCAAAATCTTTA
**CoV-bait-3442**	GTGCCAATAACAACAGCGGCGCCTCTTGTGTTAACAATAGACTTCAAATGCTTTTGGTGATACTGCCTGGTAGTCATAGTAGATAGGAGTGAGACACCACCTACAGTACGAGCGCGTTCC
**CoV-bait-3443**	AGGTGACCACACAACTCTCAATTTGAACAAAATCACTAACGGTAGGTTTTCTAGGTTCAAACATACGTCGCGATGAAGCAAAATATTCCGTCGCAGTATGATTTTGAAGTTCATGCGTAA
**CoV-bait-3444**	ACAAATTCCGCCTGTTTCCAGCTTTTGCGCAGTGGTGGAGCATACTATATCCACCTCTGAACTACTAGGGTTATCCAACTTCTCCTCTGCGTCCTCAAACATCCATGGAAATTCTGGAGC
**CoV-bait-3445**	CATACGAAAAGCACAACCCTCATAGATACGTTCACCAGTACCACAACCACAGACTACAGAACAGGTAATCTTAAGTGTGTGTAGGTCCTTAGTCAGAGACTCTAGGCAAGCCGACACATC
**CoV-bait-3446**	ATGCCTAAATTAATATAACCGAACTGTAGGTTAACACCAACATCAGAACTTCTTAGCAAGTAATACCATTGAACAACCAAGTTGTAACATACAAAACACAAAGTTATTAAACAAACAGGA
**CoV-bait-3447**	GTACATAGCCAAAAATATAGAAAACAATAAAGAAATAATAAACACAGAAACATCATCTGTGTAACTTTTAGGTGCGGCGACAGAGCGCTGCACAATAGTACTACAATTAGTGTCTGCTTG
**CoV-bait-3448**	ACTTGTTTTGCCATAGGTACACCATGTCTTTTTCCGTAGTAGAAGATGGAATCTCCCACAGAAAGGACATCAGGTCTGCTGTAAATACTTGTATCTTGTGTTTTAATAAGATCAAGTTCA

**CoV-bait-3449**	TTAAGGTTAAACCTAATAACATCAGCTGTGCTATTAAGTCTGTAACCATTACACTGCGCAAAGTCTTCAGTATTAAAACAAAAGTTTTGCGATGTTTCACTAAAGCTTGGAACCGGCCAT
**CoV-bait-3450**	TTTATAAAAGAACCTCTAATAGTACCCTGAGTACGCATGTTAACACCATAAACACCATTAGGTTTACCATCATAGCAAGCTAAGATGTTAAAACTATCACCAGCTTTAACAGTCCTGAAC
**CoV-bait-3451**	AAGTCACCACAACCCGTATTATTACAATTCCAAAATGTACTATTAATTTTACAATCATAGCCCCAACAATTACCAGAGCCTGTAGCAACAGTTGTATTAATGGCACCTTTACACAAACAA
**CoV-bait-3452**	GACGCGAACGTGGCAGTGTTTAAACCAGCTTCGCGTTTACCTTTAAGCTTTAAATCCTTAAGCATGACAAGAGCATTGTCAAAAAGTAAATACTTGCAACCAAGAACAACACATTTGCAA
**CoV-bait-3453**	AAACTTTACTTGCTTCAGGTGTTAGAGAATTTAATGCACACATTATTTCTTGTTTTGACTTACCAAAAATTTTACTAAACATTTGAAACATAGGTGTCAAAGATTTCGTCTCAAAATTCT
**CoV-bait-3454**	TAATTGACCAAACCATAGTAAGACAGCCAATTATTAAACAACTGTGCAACAAAATACAACATACAAAATCTTACAATATTGTTACCAAAAATCATGAGAGTGCCAAAATAAAATAGTTGC
**CoV-bait-3455**	ACAGTGTCTTCCGTTACCTTGACTGGGATGGTACCTTTGAAAAAATTTTCTACGGCAGCTCTCTCATGGTCAGTGTAAACGAAGACATTCAAATCTCTATCGGTGACAGTCTTAATTAAG
**CoV-bait-3456**	ATTTTGGAAAAATTTCACTAGATGTTGATGAAACGCGTGGAACGATGATCTGTTTGGCGCTTGTTGATGTGTGGATGGTAATGTCACCGACTTCAAGTGTGCAAACTTTGGGTTCAAGGC
**CoV-bait-3457**	CATTGATGCAACCACCGTCGTAACAATCAAAGTACTTACACACGCAAGGTGAGATTCCACGTCCTGCTAACCCGGACGGGTCCACTAGGAGTCAGCAGCGGGTCATGTAGAACTCATTCT
**CoV-bait-3458**	CCGGCAAACCATAAAGCGTGTCTGCTACACAGGACTACTACATAAACCATCGCAACCCACAAGGGGATAACGCTGGTAAACATAACCAAGAATGAAACATGCATAATAGCAGCCACATCG
**CoV-bait-3459**	GCAATGCCATGCTCTCTAAGCACCGACACAACCTCATCATGAAGTGAATCAAGACCCTCAACATCACAAACATAATGTTTACCCATCATATGAGGGTTTGTTATGGTTACCCATGAATTA
**CoV-bait-3460**	AGGAGTAATGGGGAACCACACTCCCGGAGTTGGGTTGAGTAGTTGCAGTCTGCTTTGGCTGATTCCTTCTGCCTCTATTTTGATTATTTGGTCCACGCTCGGTTTGGTCAGCCCAAGTGG
**CoV-bait-3461**	AATCCCAGTGTGGATGTTCCACAAATGTAACCGTCCCCAAACTCTTTATCATAGACAAACCAGTTGTCACCACCAAAGCAAAAACCAGCTTGACTCTCAGTACACTCTCCAACTCTGCAA
**CoV-bait-3462**	ATTAGGTCCAAGGTACTTGAGGACAAATGCTGCTCGGTTTCTCTGGTGTTGTTTTTTAAGATCCTGAACATTTTTGAAGGGATGTCCAAAATACTGGAACTGGTACTCATAAGTCTCGCT
**CoV-bait-3463**	ATGGATGACACGCATTTTAGCTGGAACTGCTATGGTGGTTTTGTTCAAGTACTGGCACAATTGAGTGTACTTGGCAACATTCATAAGTATGCCATTAGGCAATTTAGGAGGTTGACCATA
**CoV-bait-3464**	GCAAGCCAGCGCATTAAAAGACAGATAGTGAGCCCGATACGGTCATGGAGGAGTCCCTTGATAAACGGGAGAGCGCAGTTTCCTACCGGCGTCGCGGAAACACTTTGTAACAACGTCGAT
**CoV-bait-3465**	TGCACTACAGAACTGACATTTTTAGCAAGTAACACAAACTTACCCTTGACAAACTTGTACTTATTACGTACAATAAGACTAATAAAAGCCTTAAGGTCAAATATTGTGTCAATAGTAGAG
**CoV-bait-3466**	CGATAGCTTGATCGTTTGCTGGATTTCTGGTCCCAACTGTTTTAACTGGAACATTAAGGGCACCGTGAGCTGCAACCCAGACAATTCCGTCCTTTTGCTGGAGGTACTTGAGGTTTTGCT
**CoV-bait-3467**	AAATTAATCTGATTAAACACAAACAATATGAGGGTTGTGTGTACAAGGTGCGTAATGCCAAGCACAAAAAATTCTTTGGGTCTAATGGTATACGACGTACTGAAATAGTAGAGTAAGCTTC
**CoV-bait-3468**	AGCGCATTTTTATAGCGCCATGAGTAAAAGCAGACTAAACAAAGCCTGCCAATAAGTGTGCAACAGAAAGAAAAATGACAGCAAAACGCGCTGCCAACATAATATAATTGCGCCTCAAAG
**CoV-bait-3469**	AGTAGAGGCTTTATACAACACTGAGTCGCCATAATCTGACTTCTCAAAGGTGTATTCGCCAATTTGTGTTTTACTGTTTTTAGTTATCCTATAACCAGTAAACACGTAATTTCGGTTTAG
**CoV-bait-3470**	ATGTCATTTAATTCTCTTTCTTTTAGATTAACAACAAGTGCGTTATTACAACGACACTTAAACTTTGGAGTGTCTAGAACTGAATTATGTGACAATGCCATAATTGTGGAGTTTCTCCAA
**CoV-bait-3471**	TTTTCAAAACCTAGACTACCAATTATGCAGCTAACTAGAACAAATCTAGGAAGCAAAAACATTTTCACTGAGTTTAGTSTTCTTCAACTGAAGATTCATAAAACCCCAAACAATTACAAT

**CoV-bait-3472**	TGCCAATATCATAACACAAAGTACATCTGTTGCAGAGCATGGCAGCTTTAAGCATAACACGCTGCAAGACCCTACAAGAGGTGTTAATACTTAACTCATTTGAAATGATGGGATACTCCA
**CoV-bait-3473**	TCAAMGCATACACGCAATTGAAAATAGTTAAGATGATAGTAAGGGGCCACATAAGCCACAAAAKGATCATCTTAATAACATAAACAAACATACTGCGACTTGTGTATCCAAATTGCAATA
**CoV-bait-3474**	ACAACACCCAATGTGCTTAGAACCCAATACCATTGCAGAAGCCATTCGTATCATATTAGGCAAAGCACGGTCGCACTTTGGATAGTCCCATCCCATCAAACAGCCGTTATCTACATCACG
**CoV-bait-3475**	ATCCTCAACTTTAAAATTATAAACGTGTTCAAAACCGACACGTTTATGATCTTTATTGTAGGCACCCTTTTCGCCACACTTACGCAAGTACATAACCCAAGGAACAATAAATTTCCGGGA
**CoV-bait-3476**	ACAAGCAAATCTTTAAGTGATGCACACATAGTCTGAACTGAGATTCCTGTCTGGGCCACCAGTGGGTCTAATGCTGCAACACCGTCTTCAGTAAGACTTTGATACCCGTACTTCAAGACG
**CoV-bait-3477**	AGTATTTTAACACTGACAAGCTTGACAAGTGCGTTATTAAACAGCAAGTATTTAGAACCGAGTATAAAGCACTTAGCCTCCACACCTGCAATTGTAATCGTCTGTGTGAAAACGTCAAAA
**CoV-bait-3478**	AATGAAACATATGCCACCAAATAAGCTGCACACCAAATCCATGCATAATGCAATTGACGTGTGGCAAAGAAGTACAAAACGGCATATGCAACCATTGACACTAAGTTCTGCGTAACTATA
**CoV-bait-3479**	TAGCATCTAAACCCTTAAGGTTGAGTGCCAAATCACACTTTAAGCAACGCCAAAAAGCACTTGCTTTAACATCACACTGACTTAAAAGCCAATAGGCAAAGTCAGCCACATAACCACCTT
**CoV-bait-3480**	TGGCACTTCATTGCCTCGTACGTTTGGGTATTCTTGGTGAAAAGGCAGGGAACACGAGTGACCGAAACAGGGCCCCGACAATCGCGCGCGTACTGGCCTGAACCTTTGATAGTACCCTGG
**CoV-bait-3481**	TGCATATTAGTTATGTGTGGTATAAATACAACACACAAATAAAATAATGCTATATTACGTTTAACGGGTGCAGCTAATAGTGCTATATAAACATAAACAACATAATACACTATAGCTAAT
**CoV-bait-3482**	TAACACCATTACCTAGCATGATCTTAACATTACTTAAGACGGATGTGTCAGAGCATGTTGGCATACATCTATACCAAATGGCACCAAAATCCAATTCTAGTAGAAGGTCAGGGCAAAGAA
**CoV-bait-3483**	AGACATTTCTGCAGGCGCGTGAAACATAACCACCCTGAAGATTAACGCCATACATTTGACGTATAACTTCACCTGTAGTAAACTCATCTGTCAACGAGGTATAGCCAAGAATTTGCTTTC
**CoV-bait-3484**	TTACAACACCCATGACTTGCATATTAACATCATCAGGAGTGAACTCATCCTCCAAAGTAGACTGGCCCAGAATAGTTTTACCCTGAAAACCAGCATGCAGACTCTGGATAGCAGCAAGCA
**CoV-bait-3485**	TTAAATTATAAAATAGGTGTGGTTCACCTTCCGTTTTCATAGCAGAGACACGAATATATCCCTGTTGTATTAAACCAGTTAAAGGACATTGATTTGCACCGCTTTTAAAACAATGTGTAA
**CoV-bait-3486**	CAGTGTCACCAAGCCAAATGCCATTAAGCACAGTATTACCATAGCAAACTCTAACCACACAGCTTTCAATTCTACCAGATGGTTGCGCCATCTTACGTAGACCGGCTTGTAGTGTTGAGT
**CoV-bait-3487**	TCCCCACTACTGGTGCCACCAGGTTTTACGTAAAAACCACCTCCACAGAGGACCATTTCACTCAAAACCTGCGCGCATTCATTAGCCAACCTATAAAATCTCTCTGAAGCATTGCAACAG
**CoV-bait-3488**	GGCAGATAATAAGGTTCACCATCAACCTCTGTAAGAATTGTGTGCTGTGAACAAAATTCATGGGGGCCGACTTTAAGATCAGACTCAACCCAACACTTAGAATCTGCCATATAAACGTTG
**CoV-bait-3489**	AATTACAGTGAGCCGTACAAAACTCAGTACCTGACCATAACATACCAGATGGTGGTGCAGTCATAGCTACAGAGGCTGCACTGAAATTCTTACTCCAAGCAATAGCACCAACAGTGCACT
**CoV-bait-3490**	CAACATTTAATCTCTTTTGTTTTGGCACAGAACCAACTAATCTAAGATTAATTCTTTGATTTCCTTCACCTACAATATAAAAATTATCTACAAAGGAATAGAAAGGATTAGAAAGATCAA
**CoV-bait-3491**	AATTCCTGACTGTGCCCACTCTTTGTACGAGTGACTTTAGCTACAGTAGTACTATCAACCTTAGGTTGTTCATTCGCCGTTTTGGCTTCCACTTCACGTGGAACAGGCGTGTTGAAAACT
**CoV-bait-3492**	TATTCCCACCAACTTTAGTATGAGTATTAGGACAGAATATTTACAGCTTTACAGCACGCCTGTTAGTGTTGATTGTGCCACATATGTTTGTAATGGTAACTCTCGTTGTAAACAATTACT
**CoV-bait-3493**	ATTTGATAATTTCATTATCCACATTGTTAATAGATGCAATATCCCAGAGCGCACCTGCATAATTAAGTGCAGGGTACGTGACAACTTGATTCCAAACGGTGAAGTCTGGAATAACAACTC
**CoV-bait-3494**	TACGAATATAGTTTTGTTGAATAAGACCTGTTAAAGGACACTCGTTGAGTCCGCTTTTAAAGCAGTGTGTAACAAACACTACAATATTACTAAAGTTGCAGTGAGCCGTACAGAATTGCA

**CoV-bait-3495**	CAGAATTGGCATAAGCCGTTGTACCATCACCAGAAGTAGTGCCACCTGGTTTTAGATAAAATCCACCAGTGCAATGTACAACTTCCGTGAGGACTTGTGCCAACTCATTGGCCAACCGAT
**CoV-bait-3496**	ATTGGTGGAACCTATAAATACAACGTTCCCTACAGCATTTTCATCACAATCGACTGTTGCAGGTCCATCTGTAGCTACTAGTAGCAATAAAGGAAGTGTAGCATCAACTACTTCATCTGT
**CoV-bait-3497**	TGCTTTACACGCCTGACCTATTGCATCGTCAGAGGCGTACTGTTCTGACCAAATGCTCTGTACAGCTTGAACAGGAACTGTTATATTCATTGTGTCCATACAATACGACTTTGTGGGATA
**CoV-bait-3498**	ATAACACGTCTATGCGCATCAAACAAAACACCATTAAAATCCACAGTTAACGATTGTATAAGATCACGGTTCACAAGCTTAATAGGTTTACCCAATAGTTGTGACCAGTACACACAAGCA
**CoV-bait-3499**	CACTCCGTACTATGGACGCCATTGAAGGCCGGTGGGTTATAGTCTAGATTCAAGACGACATCATCATTAGTATGCGTCCGTTCGACTTGCTTAATCAATTCTCAGTACCTCTTTAGTATG
**CoV-bait-3500**	GGAACACATTCAGCTATCTGAGGGTAGCATTTGGCAGCGTTACCATTGGCAACGAGATCACTATCACCAAAGTTAGCTGAAGCACTTCTAGCACCATAGAAATTTGTCACATCTCCCTTA
**CoV-bait-3501**	CCGTGCTTTGGGGATCTCTTTTTTTTGGCATAACGACGTTATCGGAGCTGGGTAAAACGCTGTCGTAGGCGTTATTGGGTCTTACGAGACATAGATCTTTGCACCGGCGTGGATGACAGT
**CoV-bait-3502**	AGGCTGATGGTTGACAATAGCGGTAGGAACAACCAGCAGTGTATTAAAACCTTCATTTATGGTGTGTTAACGAAGTGATTACCAAAGTTTAGTAACTTACCATTATTTCTAATGAGCAAC
**CoV-bait-3503**	TTATAAAATAAATAACTATTACCTATCTTCATAGCAGAAATGCGAATATAGCCATTAGGAATAAAACCTGTTAAAGGACAGTTACCCGCACCAGCTTTAAAACAATGTGTAACAAACACC
**CoV-bait-3504**	AACGCTTCATCAACTGCAGCAGCCCACTCGTCGTCAGAAACCTCATGAGAAACGTCAACTTGTTCAGTTGAAGTCTCAGAAATGCACTCTGATTCACCTTCTTCTAAATCTGAAGCCTCT
**CoV-bait-3505**	AGAATGCCGTAATAACAACAATGGAAATAGCCAATGACCAGGTAAAGCAACATAATGCCCTTAAGCTGACCTATAAGTTGTACTAAGCCAGTGTGTGCAACAAGATGTACAGCAGCCTTA
**CoV-bait-3506**	TATGCGTGTTGGTAACACCAGCCTTGTCAAGGAGGTTAGCAACAACAACAGCAGTAGCTTTTGCATCTAATACTTTGTTTTGTACAGTAGAAATGGCAATATTACGAGTACCGCCAAGTC
**CoV-bait-3507**	TAATAATCACCATCCGGTCCTACAATCTGTAAGGTGTGTTGCGAACAGAATTCATGTGGTCCAACATTAAGATCTGGTTCTACCCAACACTTAGCTGTAGACATAAATACGTTATTCTGA
**CoV-bait-3508**	TTAACAAAGCAATCATAGATAGCCAAACAACGGGTCATTATAGCATCACCAGAAGCAACATGTTCATTACGATGAACATTACAATGTTCATGATGATTGGAACTCAAGGAACCTGTATAA
**CoV-bait-3509**	CAAGACTAAGTGTGAACAAGCCTAGAGCCATTACTGAATATGGATTTTTTCAACGTCGTAATAAGGAAGTTTAGTTGATTCACAACAACCCCTACAAGACGACGACAAACAACTACAGCA
**CoV-bait-3510**	AAGCTCCAAAACAACACAATTTCCATCTTCTTTTACTATCTTAGTGTACTTAAGACCATCGACATCACTAAGAATAGCATATATAATTCTACCCGTGTTACTATTATTATAATAACACTG
**CoV-bait-3511**	CGGGGATACCCTGATCTGCATCATCAAACTCCAGCTGATCATTGACTGCCTTCTCTTCCTGAGTCATGCCATCAGCTACATCAGAATCCTTACTCTTCTGCTTCTTATCTTTCTTCTGCC
**CoV-bait-3512**	GATGTTTGTGTAAAAATGACATAATCATATTCACTGCCTTGCGATGAATCGACAGTTTGTATCTGAAGACCGAGAGCTCTACTAGCCACATAATTTTGACTGTTGTATGGTGAAATAAAC
**CoV-bait-3513**	ACCTTAGCTACAGTAACATATACGGGCAAATCTGAAAGAGTATAACCAGATTGTGTATGCGCGTGTCGGGTGGGGCTCAGAGCTAAGGCGCGGAATCCGCATTACCCACCATCAGTATTC
**CoV-bait-3514**	GTAAGACACGACTTATCGCCACTGGCAGCAGCCACTGGTAACTGGCTTCAGCAGAGCGAGGTATGTAGGCGGTGCTACAGAGTTCTTGAAGTGGTGGCCTAACTACGGCTACACTAGAAG
**CoV-bait-3515**	TTCCAGTCTATTGTAAATTTCAGCAATAGAGCTGCTGATAGCCTGAAAATTTTTCTGTAACTGACTAGTAAGTTGACTCAACGCTTCACCCTGTTGATTAACTACACTCTGAATCTTAGT
**CoV-bait-3516**	CTACATAACCCGTCGGTACCGCTACTAAATTTACACCCGTCGAAAAACCTAATTGTAACGGTAAATTCGTACCTACCGCATCACGCGTCGCATGACAACCTTCTACATCAAAACCTATCC
**CoV-bait-3517**	GGAATGACTGTGATTTTACACACTCGTTAACTTTTTCTTGTGCCAGCTGTCTAGAGGCTTTCACCTCAGTATAGCGGGTCAATTGTTGAGCAACAAAGGCATTAAGTGCAGCCAAACGAC

**CoV-bait-3518**	CGGTGCGCTTCTGGCGGGGCTTCTTGCTGGCCTCGGCGGCGCTCTTCTTGGTCACGGTCTGGCCCTGCTGCTGCTGGCCCTTGCCGGACACCTTGCTCTCCAGCTGGTTCAGGCGGTCCA
**CoV-bait-3519**	GAGATCTGTGTTGTCTTCATTTGCAAAGGCATTCAGCTGTTGTAAGAATGCACCAAGGTCAGGGTTATCTTTGCTGGTTTGCACTTTGTATTTGAACTCAAGGACAACATCTTTTCCAAC
**CoV-bait-3520**	TCGGATAGATCCTTCAGGTGGTCACATACCATTTGAACTACACGCTTACGTACAACGGCCCAAGGCTGTCCCTTACGTAAGAGCGGCACAAGATGTGTAAATTGCTCACCTGGAGGTGCT
**CoV-bait-3521**	ACACCACTACCAAACTCAGGGTAACCAAAAAGATCTATGGTACAGGCACTAGCCAAAAGGCTGGTGGAAACACAAAATTTGCTAAAAGACAGGTAATCATTAACAGATTGCAAGGTGAAA
**CoV-bait-3522**	TCTTCCGGTGAAAGTGTCCACTCATTCTTTTCACATCATAATCCACAAAAACCCTTTCACACTGAGAACCAATATCAAGTTTCTCATTCTTTGCATTAACTTTCACAGTTAAACACATT
**CoV-bait-3523**	GTCTTGCGGGTAGTTAGATAGCAAGTCTGGTTTCTTTAACTGATCAACAGTACGAACATCTGTGTACTTATTACAAGTCACTATGTTCTTACCATTAATAGTAACATTGGCCATAAAGAC
**CoV-bait-3524**	AAACCAGCTATAGCCTGTATTTCTATGGTTGTTTCTGGTTGCAGGACAGTGTGGAAGAAGATGAATCCTTTTGGTGCTGCATTTGCTATTGAGAACAAATGGTAGCCATTACCGCAGAAT
**CoV-bait-3525**	GTTACTATTGAAATTAATGTTAAAACTAAAACAACACATTGCATCTTGTGGGTGGCTTTACAAGCAACTTACCCTCTGCAACAAGTAGTTTTAGTATAGGTTTTTTCACGTATTCCTTCT
**CoV-bait-3526**	CTCGATAATCTGTCCCATGCAGAAAATAGCCCTTCAATTACGAGCTGAATTAAACCAATCAGGTGAAACCTAGATGAAGACGAAGACAGTAGGTTATATGGGGTGGATCACAGACGTACG
**CoV-bait-3527**	GACCGTATTGTATGCATAATGGCTTTTCTACAATTAGAACAGCCACCATACGGAAATGGTGTAGCAACAGGCATAAACCTGAAGAAACTCCCAGCGAATTCTGCCCTACTAACTTTGCAC
**CoV-bait-3528**	TTAAAGGCATTAGCAAGTATTTGTTGATTACGCTGGAGTACATCAGTTTGAAGTGCTACATAGTTAAGTCGTGCTTGTACAGCTGTAGCAAAAGGTATAGCAGCAGCAGACGTGAGTCCA
**CoV-bait-3529**	GAAAAGTCAAATCCCAATCTACCTTATCAAAAAGATGGTTTATAGCAACACATATAGTCATCTCTGCATCTGATGAAGCTACATGTGCATTTTTGTGATAATGACAGATCGGGTCATGAT
**CoV-bait-3530**	GCTGTAAGAAACACACAAAGTACTAAACCCATAGCTAAAGATGTGCTAAGTTGAAAATACGTAACTGGTTGAAATAAAGATATGGCCAACCGTCGTACTATATCAAAATAATCATCACCA
**CoV-bait-3531**	GTGCGATTATGTGAAGGGGTACTGTTTTATCACTAGAAGGGACAAAAACAGTAGAAACATTACCACCTAAAGAAAAATCACAAACTGAAACAAGTATGGATTTCTTTAGGCGACTAATAA
**CoV-bait-3532**	ATCTGACGCTTGAAACTATCAGAAAGCTTCATATAATCACCACTATTCCAAACACATGCACCATTTGCATTTCGCATACTAGTTTGGTTAACAGAAGCAGCTTTAAGGTCAATCAAACAA
**CoV-bait-3533**	GGCACAAGCCTCTCTATAAGAAGCCGTGTCCATAGCACCTGAATAATACTTGTACTTGTTAAATAATGACAAATACCTAACAAAGGAATCTTGTGTTATAGCATTACGTAAAGCGACATA
**CoV-bait-3534**	GTGCAAAAGGTATAGCGGCAAAGGATGAAAGACCAGCTGTCCATCCAGCACCTGCTATACTACCTAATAGTGAAGATGTGTAAGCAGCTTCCATATTAGGGTCATAAAGTGGTGGTAAAA
**CoV-bait-3535**	TTTCTCTTGGATTGTGATTTTACACACTCGTTGACTTTGTCACTAGCCAACTGAGCAGATCTAGCAGCAGTTTCAGAACGCACAAGTTGTTGTGAAACAAATGCGTTAAGAGAAATTAAG
**CoV-bait-3536**	CTATGTAGTTATCTATTGTAAGATCAACCACGGAACACACATTTTTAGAGCTTCCATTATCAGCTGTTACAATCATGTTCTGTACTGGGTTGAAAAGATGTGTGACAAAATCTGAACTAA
**CoV-bait-3537**	GGTGGATACAGGTAATGATACTGACTAGGAGTCTCTTTATAAGACTTAATTTGCTTCTGCCAAAATTGGTTAAGCAAAGAGACGTAGTCCATAAGTTCGTTAAAATCAATTATACGGAAA
**CoV-bait-3538**	AAGTTACAGTGCGCCGTACAAAATTGACTAGTTGACCAAGACATACCTGTACCAGGTGCTGTCATGGCTACAGAAGATGCAGTAAAATTTTTACTCCAATAAATAGCCCCTGCAGTACAT
**CoV-bait-3539**	TAGCTACACAGTTGGGCGGAACCCTCTCGGAACCGCCTAGACTGTTTCACCGAAGTTTATATAGATGCACTTCACATAAAAGTTTAGAGTGGCCACATGAAGCCAGCCGAGTCCTTTTAC

**CoV-bait-3540**	CGTAACGTGAGCTGAAGAGGTGAACACCTTGGGCGGTCTGTGTGATTCCGAACCACTCCAGGATTTCGTCCTCAGTGATGTTGTAGGTGTACATGAATGTGCAGTTGCGCAGGTTGAAGT
**CoV-bait-3541**	ATTACCCTGCTCATTAACAGCATTCTGCACTTTATCCAAGGCCTGAGCCACAGTCTGCAAACCTTGAGAAGTCTGTTCAATAGCATTACCAACGTTTTCAAAAGCATTAGTAATGTTGCC
**CoV-bait-3542**	TACGGCAAGGGTGTGGCCATTGACTATAAAACCAAAAGGTATCATAATCAATTGCTTACCAAAAGAGTTCAAAGCTTTGCTAACATGCTGTAAAGGTGTATCACGACTGTAACCATAAAA
**CoV-bait-3543**	CGACGCCTGTGTAATTAGTAAAAACTGAGACCATAAACGTAAGGTAGCTGATAGCTACAGTATCAGCACTACCAGTCCAGCAGTTATACAACCAACCTACTGCAGTACAGACGTACCAAA
**CoV-bait-3544**	CCTCAAATGCCTCTGTTGGCGAATTTGAGGCTAATATTTCATTGTGCAACTTAACGCACAGTGCCCATAGTTTACTACTGGATTCAACACGTAATTGTTGTAATACAGACAGCAAAACAA
**CoV-bait-3545**	TCCTAGGTATGACACCAAGTGGTACAACACCATTACGCGCATTGTTCAAAATGTTACTAAGAGCGTCACTATCAAGACGACGTATCATGTTGAACAACATAGTTTGCATAGCAGCCGTAA
**CoV-bait-3546**	GCTGTAATAGCCAACCCTGTACCAGTATGGGGAGTTAACATCTTAACGCAGTTACCTATAGGGGAACCACCATTATCTAAATAATCCTTATACGTAGCCTCAGGGTCTACACTAAAGGAA
**CoV-bait-3547**	CTACTTTAGTGTTACAAATGTCAAAAGCCCTTAATACTACATCTGGTTGAACACCAGAACCTAGGGGTACTAGACGGGCTACTCCACTAGTACCCCTAACTCGTTTAAAAAGCACTCATC
**CoV-bait-3548**	CATTTAAATCCTGGTTATCCAGGGTTAAAACACCAATAAGACCTTGTTCAACCATAGAATCTGCCATCTTCACAGCAGAAAGTACAGCCTTTCTAACAGTTTCACCTAACTTATGGTAAA
**CoV-bait-3549**	AATTTTTAAGAGGATCTATTTGATCATCACTAACAACTTCTACAAGTTGTTTTTTATCAGCCAACTGTTGCTGGCAATCCTCGTGCGGTTTATCCTCTTTAAGTGATGGCTCTGGGCTCA
**CoV-bait-3550**	TTAGACCTTGTGGTATTACCACCTTTGTTAACTCTTCATATGTATTAGCAGAGTTTACACAGACCAGAAACCTAGTCTTTGCAACCTGCTGTAACATTTGCAAAGAGACTTTAGGGTCAA
**CoV-bait-3551**	GCAACTTGACTGCAGACGCTACAGCTCTAGTGCATTCTAATAATAATGGCCAAGTTAGACTCTCATTGGTATCTACCACATCAGAAGGCCTAACCACTTCACCATCAACATTATTTATAA
**CoV-bait-3552**	TCAAGGTTTACATTCTGTACTTTGAACAAAGAAGGCATGGCTAAACCAGGCTTCCAATCATTAGAGGCCTGAAGTCTAGGATAAAAAGTTTTAACTTGGCCTTCTCTGCACCATAACATA
**CoV-bait-3553**	TCTCAACATCAGACTCAACATTAGCATGCACACGTGCAGCTTGTATGAATGTTTTAAGGACACTCTGCAAGTCTTCACCTCTTTTAACACAATCCTTAGCTGTAGAAATCAATTTCTCCA
**CoV-bait-3554**	CGAAGCATAAAATCTTAATGAAACTAAATGCTTTCAAAAAGATATCTTTATTACAACAAATTTTTCGTACTGTTGAAGATACAACACTATTGACACACTTAGTGGCTACAAAAGTACGAC
**CoV-bait-3555**	TATTCTTAGTTTCAACAGGCTGTATGTCAAATATTTGACGTAACGAAGCTCTGTTATACTTATTAAGCTTGCTATCATATAAACCATTATTAACCCACAAAATGGGCTTGCCCTTATATG
**CoV-bait-3556**	CACCATGCAAATTTTCCCATTAATTCATACAATTCTGCGCTCCATGAATGTTCTGTTATTTTAATAGCAACAGATCCACCAAGAGCCAGATTTCTATTAATAAAATTGCAGAGATATGTA
**CoV-bait-3557**	ACGTGTATCCAGCACCTCTCTTACTCGCTATTGCTACCGTCGGCACACTAGTATGCATACGAAAATCGTTAAACGTCAACATAAATGTAAGACCCTTAACTTTTGTCGTACGCACAACAT
**CoV-bait-3558**	GCAGGCTCTAACCCGGTGCCTGTGGGAATTTCTCCGATATTATGCGTGCCAAGGGGAAATAGACAAGATCTTCCTACCACAGTACTCTATCATATCGGCGCAACCTTCTCGAAAGGGCGC
**CoV-bait-3559**	CCTGAACCTGGCTTTGGTGTTTCCCACAAAAACCAGTATTCGTCCTCATCATAATCATCCTCATTAGTCTTACCTTCACCTGGGTTTAACTGAGCAAGATTATTGTAACTAGCAGTATCA
**CoV-bait-3560**	CTACACGCAGGTCCGGTTTGTCCGAGATGAAAGCATACATAAAAGTACGTCCACCCTCATTATTGTAAAGTGCCTTACCTTCTCCAACTATGCCATCTCCTTCTGCCTTAATGGAGCGCT
**CoV-bait-3561**	CTTAGGCTTGGCATCCTGTACATTATCCAAGACCGTAGGACGAGACTCACTAGGAGTGTCATCCACAGCTAAGACACTAAATTTATTTTCAGAAACCAAACTAGGTCTATTAAAATATGT
**CoV-bait-3562**	CGGGTTGGAAAGGCCGGAATGTGGAGACATAAGACTACCCGACATATTTGATGGGCCATCGTACTATCCAAAATGCACGGGCTTAAAAGTGTCACTCCTATAGACATGTCTTAGGGAGTT

**CoV-bait-3563**	ACACTAACAACAAAAACAAAAATTGCTCTCTTAACATTAACCACCACCCTAACAACAACAAATAAACACATTATTGCAACTACAATTATCAAACTCATGCGATTAACAACACGATCTGAC
**CoV-bait-3564**	TCTCTTAAACTTGGTCACAAGCATACAGGCAAAAATAGCAAACAATGCTACTGCACAATTCAGCACAATTTGACCAGACATAGCCATAACAGAGAAAGATGATGAGAAAACACTTAAAAG
**CoV-bait-3565**	CCATACTATTACACACTGCACACCAGCGTTCCAACGGTTCAGAACATGGCATGAACCTGAAAACACAACCTTCAATAGTAAGGTCTTTGGTACCACAGTCACAAACAGCGTCACAAACAA
**CoV-bait-3566**	CGAAGTTTATTTGAAGCACACAGTGGAATGACACTAAGAGGTATACAACCATTCCTAGCGTTAGAAATGATACCATTAAGAACATCGTTGTCGAGCTTCTTAATCATACCAAACAACATA
**CoV-bait-3567**	ATCCATACTAAGAAAATCTTCTTCCATTGTACTACGAGGTTTGAAGGTTTCAAAGGTACGTCCCTGTGTATAATAACTATCGATTTGTTCGACGTACTCACCATTCTTGCGCACATAGAT
**CoV-bait-3568**	AGATGTGGCCGTTGTCCGGGGTACATTTTTACTGTATAGCTAAACTTAATGCTCGATGCCTTCATAAATGCATCTGACATCAAAACAGTGGCATTGAAGTTGCCATTAGAATACCTCAAC
**CoV-bait-3569**	TTAAAACAGCAATAGGTATGTGCTAAACTATTATAACAAGTAGCTGTTTTGCCACAATCACAATGTTTTGTTGGTCCAATTTTAACAAAATAACGCATGGTCGTTAATTCAAGACCACCA
**CoV-bait-3570**	GGAAGTATCGCCGCTTTAGGGGTGGCAAGGCATTAATTGAGTTTGGTATTCTTGAACCCCTATTCATTGGTGGACGGGCGTTTTCGCATTTGATATCCTTGCGGACCCCGGACTCAGCAT
**CoV-bait-3571**	ACATCCATAAGATATATTATAGACTGAACATTAAGAGCAGTCTGTTTAGCATGGGGAACGTCCATATCACGTGCGACTTCCCAGGCATGCTTGTAAGTAATGTCATCAATAACAATATCC
**CoV-bait-3572**	GAAAATAGTATAACGGCAAGAAAAATTGCTAGCCACACATACCATGGCCACTTAATGTATTGTTCGAACCTGTTGAGCCACTCAAGATCAACATAAGTCTTATTGAGATTATCAATAAGT
**CoV-bait-3573**	TGAAACCATGAGGCTTGTCCCGTTCCTCCAACTTTGGGTGGTGTTGATCCTGTATTCTTTGATAACTTAAGAATGGGGGCCGGGGAGTCAGATTTTGATTTTCCGGCAGACATGATTGTA
**CoV-bait-3574**	CCTCTTCAGAAATAGTAAGCATAGAGTTAACTTCAGCAGACTCAAGCCTAGCGCTGAGTTGTAATGCTGACTCTATGGTCTTACATGCTGCAGTGTACTGGGTGAGTAATTGTTTACAAC
**CoV-bait-3575**	TAGCTTTACCACTAATAGCGTACTTAAGATTAAGCTGTGTCATTGTAGGCAAAACATTTCTTTTGGTAAGAGCATAAAGGGCATCTTGTTCTTCATAAGTCATAGACTCATAATAGAGCC
**CoV-bait-3576**	GTGGTAAAACAGTAGTAGCATTACTCATCTTATAAACTATAAAACGCTCTATAGGTTTGGTATAATTTACAATAGGATATAACCCATCTGTAAATGTACCAGTATTATACTGACAAGCTA
**CoV-bait-3577**	GGGTTTTCAACTCCTTCAATCAGGTTTTTAAGCATGTTGTCCCAGCCACCATAAAACTTAGTTGTACCTATAACAACGGCGGCATTACGCGCATTGACAATAGATTTAAGATGTTTTTGG
**CoV-bait-3578**	TACAGAACTGGCTGGCTGACCAAGACATACCTGTATCAGGTGCTGTCATGGCTACAGAAGATGCACTAAAATTTTTACTCCAATAGATAGACCCTGCAGTACATTGACCTGAGCCTGCGT
**CoV-bait-3579**	CAGACATAGGGGCTTATGGACAAAATAAACTTGTGTGGTGTACCGACAACATGGTCATATGCACACTTTGTACACAACAATGGACGCCGGATACAATCACCACATCTCAAAACTGTTTGA
**CoV-bait-3580**	AGAGGACTGTGTAAATCCATAGGATTTAAGTGGTGCGAAACAGTTAAGATTGTTAATAGTAGAACAAGTACCACTAGGACTGTAAGGAATATTGGAAAGGTCACGGCCATATGGTTTGAT
**CoV-bait-3581**	CAAAAGGTATAGCACCAGCTGCAGTAATACCACCAAAAGCCATAGAAGCTACTAGAGAACTAGTATACAAAGCTTGCATTTCTGCTGTTATGATAGGAGGCAACACAAGCAAACCATTAT
**CoV-bait-3582**	GAAACCCATAAACGAGATAACATGCTCATATTTAATGGGTCCATTAACACCTATTTGAACAGCAAGATCTTGATCAGTCTTAAAATTGTCCGCTAAAGACATGAAGGTGTTAGCGTGAGA
**CoV-bait-3583**	AACACCCTTAACAGATACAACTCTATCTATCAGGGGCGCGCCATGAACACAATTGCCATCCTCAATACCTACGCGTAAGACGCTGGAATTAACCACAGTGGAGTTAATAGTGCGCTTACG
**CoV-bait-3584**	GGGGGTGAAAGAGATAGCTTGGTGAGATGTTTGGTATCTAGCACCGCCAATTCTAACTACTGCTGCTTGTGCGCTGATGGTGGATTTGCGCAAGACAGCAAGAATTGTAGACATGTTAGC

**CoV-bait-3585**	TTTGAAGTGGCTTTCAAAACTTTAGCTGGGACCCCTGGTGTTACTTTATCCACTCCTTTAAGAAAGAATGGACCACACACAGTTTCAAATTTAAACTGCTTCCCAGCGCCCAGATTACGA
**CoV-bait-3586**	GCACAAACATCCTTAGTGAAATGTGTAAGTGGGCGCTCAGCATCAAAATCCCACAAAACACACTTGCTAGTACACACAACACCAAGGTTACGCAAAATGGTAATAGGAGGTGTCAAACCC
**CoV-bait-3587**	CAGAAGACGCTTCAGCTGGCGCACAGCACGGCCGTGAACCACCAGCATGTAGGCGATGCGTACCAGAGGGCCACTCACAGGCTGCTGGGCCCGGACCTCTTCCCACTGGATGCCAGGGCT
**CoV-bait-3588**	ACATCCACCTCTAAATTACGTAGCAAATTCAAATCAGGATGAGAGCGAACAGCTCTCTTAGCATACAATTCAAATGCTACATTTGTAGGAAGTGCAGTTTCATTCTTAAAGATGCACACA
**CoV-bait-3589**	GCTGGCTGCATTACATATACAACTATATAATAAGCAAATTTGTATGATGCAAAAACAACAAACCAATCCTTTGTAGTAAGACTTATCAACATGGTAAGCATGTTTACAAGACACAGCAAA
**CoV-bait-3590**	TGGAATGCACATAATCTGTGACATTTTGAACAACATCATTAATGAGTTGTGCCTCATCTGATGATATAGTGGGTGGTGCTTGATATGCATCAACCACTAGTCTATACAAATTCATGCTGT
**CoV-bait-3591**	TGATCTTCCTTCAAACTCATAACAGGAGTGCCTTTCAAGCGCAATGGGAAACGCTGCAAATCATACAGACTATAACTAGACAATTGCATAACAGTGCTATTACGCCAAAAGACGTAATTA
**CoV-bait-3592**	AAAATCAAAATCACTAGGTACCGACGGGTTCACCACAGTGTAGTTAACATGTGAATAATTCAAGCCACATGTAGATAAAACCTGCGTGTTCTCAACTGTAATACTAACAGGATTATAAAA
**CoV-bait-3593**	CGTATAACAGATCAGAGAAAGCAGACCTATAGGTGGTGGCACCACAATTAGGGCCTAAACAACCCATAAGGCCAGTAAAATTATAGTCCCCATCAAAAGAAAATGTAGTGACTTCATCCC
**CoV-bait-3594**	AGTGGCATTACGAACATTATGCACGCAACCCGAAAGCGTGTCAAATGTAACTACACCAGTACTAGCATTATCTGCAACAGCAGCTACCCAGTAATCAGAGGCTGGTAAGCTGACAGCTAC
**CoV-bait-3595**	CTAAACCCTCTAAGCGGCTGAAAATTTCATTAATACTAGAAGAAATTGCACCAAAAGAATTACCCAATTGTACCACAAGGGTGTGCAACTGCGCAGCATGTTGATTAATAACATCCTGCA
**CoV-bait-3596**	AATCCGCACAAAAACCATCACCTAATGGATTTGAGCAGTTAACAAAGGTGTCATTAGGTGCATATGTAGCATTAAACAAACAACCTAATGCAGTAGCTGTACCATTATGTTGAGGTAATA
**CoV-bait-3597**	TATCACCTTCAAAGAGCTGCGTAGAGATCTTAAGCTTGAGAATGGCAGGCAGCACATCAACGGCTGTAGCGCCCAAATACCAAGCAACCAAAAACCACGGTGCTAAGGTAAAGTAGGCCA
**CoV-bait-3598**	CTGTTAAAGTCACAGGCGCATCATAGTCACCATCTAACACCTCGACCTCCATCTTTTGCTGTCTAGGTGTCAATAAGAGGCCCAGGCGCTTAGCACACACCTGAACCTCGGTAAGCAATC
**CoV-bait-3599**	ATTATAAGGCTGTCTACCTCCTGGTGTAATGACAGTGGCCGGGTAATGAGTCGCTAAAGCATAGCGGCCATCAGTAACATTAATATAATATGCGTGAACACAACCATAAAAATTGGTGGG
**CoV-bait-3600**	CACAAACAGGGCTACCTATAGGTGTAAAGCGTATTGTCATGAATAGTAATGCATACAATGCATAAATACCCAAACTAAACTTACCAAGAACTTTGAACCAATAAAGCTTTAGCTTGAAAA
**CoV-bait-3601**	CGGCAAAAGGCAACGCCGGATGTCCATGAGTGTGCTGCCACACACGGTGTGTTGGGATCTGCGCGTGGCTATAAGCAAGACAGCAGCCGACCACCGGCGTTGAAATCTCACAGTGTTTCG
**CoV-bait-3602**	GTAGTCAACTCTAGTCCACCCGCCCATAACACAAAGATTATGATGTCGGACATACCAGACATACAATCACAACACATTTGTACTATACGCTTACGCACAACTGACCAAGGTTGGCCCTTG
**CoV-bait-3603**	TTGAGAACAAAACTCATGAGGACCTTTAGTAAGGTCTTCTTCGACCCAACACTTAGAGGTGCTCATAAAAACATTGTTCTGATAATACAACGTGGCCTTAAATGCGTTAATATCGGCTAC
**CoV-bait-3604**	TGTCGTAAGTGCAAGAATCCAATGTGACATCAGAGTATTGGTCAAAGGTTGGGGTGGCAACAAAGCCATCAACGGCCTGGGTTTTGAGGACGCGCCAACATGCAATGTTTTGAACAGTAA
**CoV-bait-3605**	AGAATGCAGCAGATTGTTATAATCCACAAACAAACTAGCATTAACATCATCATAAACACGTTGTCCATCCCTATCGTAGAACAAACGCATAGAACAACCAACCTGCTTAACATCTGTAAC
**CoV-bait-3606**	CCACCACCAAAATTCTGATTGGGGCCTCTTTTCCCAAAACACTGCTGAACAGTGCATTGTTTATTGGGGCTCCTCTTTTGGCGGGGTTTGTTCAAAATTTTCTGTCTGACTTCTTTGGCT

**CoV-bait-3607**	AAAGTACCATCACTACGTATACCAGTATGCAACTTCCTGAAGTAACTAAATACCCAGAAAGCATGGTTTGAAACAACAGCTGCAACATAAAGTATACAAAACCATATTGGCATAATTGTA
**CoV-bait-3608**	AACCACACTGTATACAAAACATAACTTATAACAAGTTCCAACACCAGGACTGCAAAGCTCTTATAGTCAAAACCAGAAAAGCCCGCAACATGATACTGCACAAAGTCTATAGCCTTGTAC
**CoV-bait-3609**	AATGCATTAGCAATAAGCTTTTGGTTTTGACTTAGCACATCCATGGTGACACCAAGCCCATTAATACGAAACTGAACATTTAAATAAAATGGTACACCTGCTGCTGCTGTCCAAGGAGGG
**CoV-bait-3610**	AGTCTTCAGGCTGTATATCCAGCATGCTAAGACTAATGTGTGGCGCTGTTTGTATTTTACAATCCACTCCTATATCAAGAATTTGAAATTGTAAACCTTTATAATTTAACACAAAACCCT
**CoV-bait-3611**	TGTTTGTTTACATGCTGAGCTGGTCAACCAAGGATAACCAGGACAATCATATATAGGTTGTCCACTAGTTGATGTACCAATTTGAGGTCTTTCAACAGTACCAGTGGGGCAACCAAGTTT
**CoV-bait-3612**	ACAAGGCCAATCATACTGAACAACCATACAAAAACTGCTGCAAAGTTACGTGAGACAACGTCACCAAATGAGAAAAACTTCTCTGATGCATTGTCTAATTTATTCATAACCTCTGCAATT
**CoV-bait-3613**	CTAAGTATCCTCACAAAATTGCGAGCATAATCAGGCACACTTCCAACAAAATGGAAACCATCTTCTAAAATTAGATAAGCACCGTTTATGACATGAAGGACAACCCAAGCAAGAGCACAA
**CoV-bait-3614**	ATCGACAACCTGTACTGGCGTAAAGCTCTTCGGCTTTGTCAGACAGATAGCCTTCGTCGGAACTGTGAGAAGCAACTCATTAGAAACAGATTTATAAATTCCTGAAGGCAAGGTAAGATA
**CoV-bait-3615**	TCCTCTAACAGAGTAACTGAGAAAGATTCAAGCACGCCTTGATTAAGTGTGTTGTGGAGGTGTTTAACCCACTCAAGAAGTGTATAAAACACTTTCCTGTACTCAGGATTGTGATGTTTA
**CoV-bait-3616**	GACATCTGGCCCAACAGCACACATCCTCTGAGTTACAACATTGTAGTCCTTAGGTTCAAGAACACCTTTAGTAATCATAGTACGTGGCGCGGGTAACTGTTGTGGGTCTCCAACATATAC
**CoV-bait-3617**	AAGCGGTGCTGACATGTTATTTCGCCCAGGGTGTTAAGGTCCGCCCGAGTAGTGGTTACTTACACTTACTTCAGTTTTTAGTCAGTAACAAGCGGGTGTAACCAGGCACAATATGTGGTC
**CoV-bait-3618**	TTAACACCAGCCAATTGTTGATAAACGTCAGAAGGTGCCAATTCATCTTCAAAAGTACAACTTCCTAGTATTTGACGACCTTGAAATCCCATATATAGACGCTTAATAGCAGCCAATAAA
**CoV-bait-3619**	CTAGTGTGGTTTCTATAAATAGTTCCCGGTTGAATTTACTTTAGCGCAGAGTGAGAAGGTCCTGCGGGTCGTGGAAAGCATGTACATATGGCTCTCGCGCAGGGGAAAGGCGATAAGAGC
**CoV-bait-3620**	AAATATACAGATGTCGCATTATTAACACATTGAAATGATTTAAATTTAGTGTACACAGAAGCACTAACTGTTAAATTATAAAATAAATCATTCCCAGACATAGCAGAGATGCGAATAGAA
**CoV-bait-3621**	TCAGCAATTACAGCAGTTGGTAAGTCACCTTTTAAACCAGTGAAGGCACCTTTCTTAACGACATTGAAAGCCACATTTTCTAGACTCTGGAGTGCTTTGCTATTAACAAAACCGTGCCAC
**CoV-bait-3622**	CCTTAACGGCACCATCAAAAGCATCATTAATGACATACATATCATCACCAGATGGATAATGATAAATGTCTATAGCACATTTGTGAACGAGACGCCCACAAAAAACACTATATTCATCAT
**CoV-bait-3623**	AGGTCTACCTAGATTGAGAGGCTTATTTATTCCTGCCGCCTGGGACCCCCACGCATAAATGAGTCGCTTCATTCATGTTACTTTGTCTGATATTGCTCCTTTTGATCCGCTACCGTCCCT
**CoV-bait-3624**	ACGTGCACGCTCCTTACCAGATATGGCGTACTTGAGATTTAACTGGGTCATAGTAGGGAGAATGTTACGCTTTGTTAATGCAAACATAGCATCTTGCTCCTCATAACTAATAGACTCATA
**CoV-bait-3625**	ATCTATTTTGGAAACAAGAAGACAATAACCTGATTTTCAAGCCAGCATGGGTAGAAGAATTTAAGAAATTTTTCCACAAACCTGTCTCTTGAAAGAGAAACGGAATGTAAGGTAAACAAA
**CoV-bait-3626**	AACTGTTAAATTATAAAATAGAGAACCATTTCTCATAGCAGAAATACGAATATGGCCCTTATCTATGAGACCTGTTAAAGGACAATAGTTTGCAAAACAATGTGTAACAAACACTGTAAC
**CoV-bait-3627**	TGAACACTTAAAGAAAATGGCACACCCGCGGCTGCAGACCAAGGTGGGAACATAGCAGACGCAGTAGCACCAGCTGTATATCCGGAGATCTGACTCTCGGATAATACAGGAGGCAGCACT
**CoV-bait-3628**	ATGAAGCCATTTGGAAGTTCTTCAAAGAATTTAGAAACAGCACCATTAATACTTGCAAGACAAGTTCCAGCAAACTCCCTAATCGTAAGGGCATGTCCAGAAACCACAACTGTAATTGAT
**CoV-bait-3629**	AATGAGCTGTACAAAACTGTGCATCTGACCAAGCCATACCAGCAGGTGGTACTGTCATGGCTACAGAAGCAGCAGAAATATTATAGCTTTCATAAAAAGTACCAGCAGTGCAATCAGATA

**CoV-bait-3630**	TATTAGCATAGGTTATGATAGGTTCACAATCAACATCGTTACTGTCAATTGCAGTGCCACGAGTCCTCTCATTTGTATAATTATATATAGAGTAGTAATAAAAATTAGGTGTTGTTGTCC
**CoV-bait-3631**	TGTACGGGACAAAACTCCGGACGTAACTCCCGACATTCCCGCTCGGAATTGTCTAAGGTCAATCCCTATTGAGTGTCCTTTTCAAGCGCAACAGGCCCCCTTTACATCTGAACGATGGCG
**CoV-bait-3632**	GAGTTAAGGCAAAGGAAACAGGTTCCCTATCTTTGTCAATGTGAACAAAGCGGCCTTTGTAAGGGCACCGTCCATCAACACCAGGGTGTGGTATATGTGCACGGCAATAGAGACAGATTG
**CoV-bait-3633**	GATTCTTCGCGCTAATAGCATACTTCAAATTCATCTGGGTAATAGTAGGCAACACATTACGTTTTGTGTATGCAAACAAATCATCTTGGTCTTCAAAGCTCAATGACTCATAATAGACAC
**CoV-bait-3634**	TCACCTTGTTTTACTAACATCGTATGTTGCGAACAAAATTCATGCGGACCTTTCGTTAAATCCGTTTCCGTCCAACATTTCGCTTCCGACATAAATACATTATTTTGATAATATAATACC
**CoV-bait-3635**	TGTCACATGTTTGGTCCTAGGGTCATACATATCGCTAATAATAAGGTCCCATTTATTAGCCGTATGTACTGTTGCACAGTCTCCAATTAAAGTAGAATCTGCGTCGGAGACGAAGTCATT
**CoV-bait-3636**	TCAGAAGAAAGGTCTCTCTCAAAAGGTTTAAGTTTAGTCTTCCGGTGAGACCTGTAGTAATACTGACCTTGATCCTGCTTTGCAGTATTCCAGGCTATTACGCAACCAGTAAAATCATCA
**CoV-bait-3637**	CATCTGAAACGTAGCTGTCAATATCATTATCTACAAGAAGTGTGCCTTCAGGAAGCCATTGTTTAAGAACAGTAGTTCCCGGAGCAACACCCTTGTCACTTCCTGCACCAAAATGCATGA
**CoV-bait-3638**	ATTACTGTATGTGATGTTAACACAAAGTAATCACCAACATTCAATTTATAAGTTGTAGTACCTCTGTACACAACAGCATCACCATAGTCACCTTTTTCAAAGGTGTACTCTCCAATCTGT
**CoV-bait-3639**	CCAGTAATATCATAACAGGTAGCAGAATTTGCAAAAACTGATTTTGTAGCAAAAACAAGGGTTTTATCAACCAACGAAACACCAGATGCAACACCAGGTATCACACGGGCTTCATCATTG
**CoV-bait-3640**	CATAAACCAACATGGTTTTTAAGACAAGCTGGCATTTTGTATTTCTTAAGATGTGCCTCAACTAGAATGTTGTCACCATCCTTAAGTGCTCTGAAACACAACTTTTCTTGTCTAAACTCT
**CoV-bait-3641**	CGGTTTGTAATTCTTTTTTCAAGTTTACAACTGGCGTCGCTTTTGTTTTCAATTCAAACTTAGCAACGTCAAATATACTAAATGCGGAGGTTTGCAAATAGCTAACATTCCGCCAAAATA
**CoV-bait-3642**	CTAACTACTCCCAACTATATAGGTACAAGCACTAGCTGCTTACCGTGATGCATACTACGAGTTACGTAGCCCCACCCAACGGCCCGCTTTATATAGCACGCCTGGCGTGGGAGGTTGTTA
**CoV-bait-3643**	AACACCTCAAAAATAACACTTATAATCTAATAAAACTAAAAAAAAACATACTACCCCAACTTAAACAACCCTATATATTCATTAAACATTCTAATACCTTAAACACCAATCACAACCACA
**CoV-bait-3644**	AAGTCAAAGACTTCATCAGAACCAGTTGCCATAGCCTTGTATAGACCAAAAACATTACCATTTGAGCAGAGCGCAAAGGATAATTTAGGCCGATGGTCTACACAATAGAAGCTCATGCCA
**CoV-bait-3645**	CGTACTGTGCACAATCAATAGACACTGCCTCCGATGACATTTGCAGGTACTCAACCTGCACAGAAATGGTAAAATTACTAGGGATTGTAACATTACCAGTGCTGATAGGTTGTACCGGGT
**CoV-bait-3646**	TATTAACTGCTCTTGCCTCTTTGTACATCTGTGCTGCAGCCTGCTCCGCCATTCTATCAAGCTTACGCTGAGTAGAAGCCTCACGGTCAAATTCACTCTTTGCCACATTCATAGCATGGC
**CoV-bait-3647**	AATGTTCGATCACACTTGGTATAATCACTCCCAAAAACATCAAATTGTTCAATAGCACCATACTTGTCCTTAAGAAACTTGGAAAATTTCAAACCATACTTGGAAACACCTATAAGACAA
**CoV-bait-3648**	AACTAGACCATTCTTATAACAGTAGACAGCAGTTCCACCCAAACCAGATAATGTGGTGCAAGCCGAATTAAAAATGCAAGCGCCCTTATCAGCAACGCCACTAGCATCAAAGCACAAACC
**CoV-bait-3649**	GGCTGTTAGAAAACTACCGTTCTCTTCTAGCGATTTATTCAATAAATTCGGCATCTGAACAATCGTCAGCTGACTCTATTTCATAAAAACTACCACTTGAATAATAACAATACTCAAATG
**CoV-bait-3650**	AGGTGGAAGCAGACTGATGTGCATCTTTTTGGTTGTTCGTAGAGGAGTACTTTGGATGAGAAAACGTACAGGGTTCCTTTGAGAATGTCACCGAATGTTGGAATATCCAGTTTATTGAAT
**CoV-bait-3651**	GTATCGTTAAATACCCTGATAAGGTTGCAAGCAGACAATTAAGACATGGTTGGAAACATTCGATCAAAAACTACGGACCTTTATGTCGTTCGTTTATCCAAGTTCCCATGGGGAATGGAC
**CoV-bait-3652**	GGGGTATTTGACAAACAAAATCCCACAGGGTCACGGACACACTGAGCAGGGATTTGGACAAACTTACCCTTATATTTACAAACACCAGAGACATCAGGATGTTCTATATGCGCACGGCAA

**CoV-bait-3653**	ACATTCTTACGCAAGGGCTGAGCATCATAATAATGCCAGTCGACCGCAGGGTCATCAACAATAGGAATTCCCTTAGGGTTGCCTATATCATAGACGCGTTCAAATGCGCCAGCAAGTAAT
**CoV-bait-3654**	CCAGCTAAAATTGTAGCTGGTGAATATGTTACGGGTGGTTCACTTGTAAAGTATTTACCATCCTTAACATAGAAAGCAGATAGGTCGGGATCAACCTCTGTTCTGAAATTACCGTCCAAA
**CoV-bait-3655**	TGATGTACCAGGAGCGATACCTTTATCAGAACCAGCGCCAAAATGTATAACACGCATATTGGCAGGCACGGCTAATGTGCAAGTATTTAAATACTGGCACAATTGCATATATTTAGCGAT
**CoV-bait-3656**	GACTGTGTTGTCAAGCCAAAGACCATTAAGAGTCATGCTACCGCAGGTAACCTGAACCATACAAGCCTCAACATCTCCACTGGGATGTGACATTTTCACCAAACCGCTTTGCAACACGCC
**CoV-bait-3657**	AGCCTCACTGGGGTCTGTTGCTGCCAATATATCATTATGGCATTTAACACAGAAAGCCCAGGCCCTACTATTAGCCTCTAAGTGTAACTGTTGGAGCACAGAGAGGAGAACCACAGATGT
**CoV-bait-3658**	TCTGATGATATTGTCTTGTTGTCATAGTACTAAGTAAGGAGACACCACCAACTGTCCTAGCTCTAGCTTTACCACTAATAGCGTACTTAAGATTAAGCTGTGTCATTGTAGGCAAAACAT
**CoV-bait-3659**	GGAGCTCAGTCCAATTGGTCTTTTGCGGGGTCTGCCGGGCTAACTTTGTTCCGTGCCGGGATTTAAATGTATGAGTGGGGCTACAAGGCGGTATCAGCTATAGAAATAAAGCTTTTAAAT
**CoV-bait-3660**	TCCAAAATGTCCAGATAATCTGGGTTTTCTAAAATCTCAATGTGTTGGTTAGCCTTAGGTTGTAAGGCCTGTACAGGAGGTGGGTCCAAATGGAGCACACTATCCTGTGGCAGTTCTTCA
**CoV-bait-3661**	CTGGGGAAACGAGACGCATTAAAAACTCTATCAAAAGGGCAGCGGTTTGTAATGTTAGGAAATCTAACAACCTCTGTGGATGGGGAAACCCTGAAGTTAGAAGTCTGATAAATGCCCTTG
**CoV-bait-3662**	ATTGCTACGTTCATAATCCCAAAGGACGAACTTGTAGCAAATGTCTGCTTGTAAATTGTGTAGCAATTTGAAATCGGGATGCGAGCGTACAGCACGCTTAGCATAGAGTTCAAAAGCTAT
**CoV-bait-3663**	TGAACAAGCTACTACAATACCTGGTACTTGTAGATCTGACAACCCAGTAAACGTGTCACATTTTACACAGTGTACTGTAGGTTTGAAATCTAAAAGAGTAGTTGTAAATATCATACCATC
**CoV-bait-3664**	ACACAACATCACTAATGGATGAATCTTTTAAATTAATGACAACTGTAGCCTTATGCTTACAATTGAACTTGCTTAAATCAAGTACACTATTGTAAGACATAGTCATAATTGTGGAATTAC
**CoV-bait-3665**	TGGTGTAAAATCAAGATCAAAAGTTGACATACCTAATACACTAGCAGAGCCCCAATTAGTATGTAGTGTTACTAATGTTGAGAGTACTCTCTTAATAGAAACACCTGTAGACTTTGCTAA
**CoV-bait-3666**	ACAAAGATCCATCATAGTATATTTGGTAAGATTTTTCCTACAAATATTACCAAATTCTTGTCTACCATGATGAAATTTAAAGAAATCATGTTCTGCAACACAATTACTATCTTTAAAACT
**CoV-bait-3667**	AAATGCAACAGTCCAAAAATCAGTGGTGACGGCAGTTGTAATATTAAAGTTTACGGCGATAACATCTCCCAATTGGAAGTAACGGTAACCATTAATATAAACTTCACCAGTTCTCGAAAT
**CoV-bait-3668**	GGTCCAACAAGAGAAGCATCGCCCATTCCCATGGTTGCCAAAAAATGCAACTGTTCATTATCAAGGCTGTCAAGATTAATTGACATCTGAAAAGCTCTTTCACTATGTAACAATGCCACA
**CoV-bait-3669**	CAACACCAAGACCTTGTAACTGATAGTTAGTTATAAAGTCTTCTGGAGACATTTCAAGAAAGTCTTTTTCCATTGGTGTTTTAGTGATAAATTTGTCAACTGACCTACCCTGGGTAAGAG
**CoV-bait-3670**	TAAAATTATGCAAAACACCATATTCAATACTAGTAGTTACACGTGGCACATAAAGCACTTTACTAACTGGAGTATTCTCATAAAACGGATATAGCCCATCTGTAAAGTTACCTGTGTTAT
**CoV-bait-3671**	TACGATAACCTTAGAGTAGACTGCCTGTGGACAAATCAGTGCCCCTTCTTGGTGAACCAACCCAGGGCGTCTCACTCATCCGGCTTGCGTCCTTGGGGGTGGAAGACCGAACCGACACCT
**CoV-bait-3672**	GCCTTTGCTAAGTGGCAGCAAGCTGCTTCACGATAGCTGGTAGTATCTAAGGCTCCACTGAAATACTTGTACTTGTTATATAGAGCAAGATACCTGTTATACTGTGTAAGTGGCAACAGT
**CoV-bait-3673**	TTTCATAATCCCATAAAACTTGTTTATAAGTGCATGTTACGCCTAAATGGCGCAGCAGTTTACATTCTGGAACAACTTTAACTGATCGCTTCGCCCACAACTCAAATGCTACGTTAGTTG
**CoV-bait-3674**	CATGTCCATTTCTAAGAAGTCTTTTTCCATATCACTACGCGGCAAAAACTCTGCTATGTTACGACCTTGTGTGTAGTAACCGTCATACTGATCCACAAACTGTCCATCCTTACGAACATA
**CoV-bait-3675**	ATATATGGATTGCTCATGTTCCATGACAGACTTAGGACATCTCTTTATAACAAAGTATGCATCCTTATGTAATTTGTTTCTGAAACGAACACAGTTCGTTTTCAAAAACTTACCAAGACA
**CoV-bait-3676**	AACTAGACCTGAATCCAATAACATAGTCGCTGTATAACCAAAAAGCGCTGAGCGTACAAAGTTATACAAAGCGTACAATGCCTGCATATTAGATCTAACAACTATAAGTGACTTAACAGC

**CoV-bait-3677**	ATTGCGAACACTAAGGATCTACCAACAAGTGCTACATAGGCTGGCACATCAGGAATTGGTCTCATATTACCTAGATCAAATACAGTTCCTAAAACAATAGGACAAGACTTATCAAATGTA
**CoV-bait-3678**	GGATCTTGACAGTTGATAGTAACATTAGGTGTGCATGTTTGAACCATAGTGTGCCATCTATGAAAAGGTAAAACCTTTCCTAGAGCACAAAGCCAAGCAGTGCTATAAGTATTACCCCTA
**CoV-bait-3679**	AACCAACGTCAGCTATGTCCTGTGCAACGCCAAGTTTACCCACACAATTTTTGTAATCCTGGTCAACGGTACCAAGACCACTAGTAACAACTTTATCAAAAAGTATGTCTTCAACAACAC
**CoV-bait-3680**	GAACGGGTGGAACGCGCTGCGATCCAAACGCAGGCGTACTGGACCCCACAGAACTGGGCCTCCATAAACCCAACACGCTTACCTGCCTCGTGTCTTACACCGCAAACCCACAACTTCCAC
**CoV-bait-3681**	TTATGTAGTCTCAAACGATTGAGGGTAAATGGAGGAAAAGATGGTCCAAGAGGCGCCCACAGAGAACCGCGGGTCACGAACGATTTTATCTAATACAGAGTGATGCACTTTTATGATAAA
**CoV-bait-3682**	TAAAAACGCTTCGGATGAAGACGTATTAACACTGGTACAAAACAATGTCCAAAACTCAAATCGTTGAACTAACTCATAAAGTTTCTTGTTCCACGAATATTCAGTTACCTTTATAGCAAC
**CoV-bait-3683**	CAAAAGCATTCAACGCACTAAGACGACCCGTTATCAAACGGTCTGTTTGTGCGTCAGCAGCAAGTTCATCAAGTCTATTATAAATATCAGCTATAGAATGCGAAATAGCCTGAAAATTTT
**CoV-bait-3684**	ACACCATCATCAAAACCACTCTTAAGGATACTAGCTTCTACCACAGGTGTAGTAAGAGTACGTTTGCTGTTACAACAAACCGAATCTTTTGAAATGGTGACTGTACCACTATTCACTAGA
**CoV-bait-3685**	CAATTATTCAAACTGCAAACACCAACTTGCTCGTCAAGTACAACATCAGTCTTAACCTTGACGGTGTGCACATCACGACCGTCATGTGTAACTTTAACTTCTACTACTGCAGACTCAAAA
**CoV-bait-3686**	ACAAACCCATGGAACCATACTCTAACACGGGTGTCTGACAACCACCAAAACCATTAGTGTGGTAGTAAAAAGATGGTAGCACTGTGGTGTTAACAAAACCAAAAGTGACATTAGCAGAAG
**CoV-bait-3687**	GAAACTACAAACTGCTGGTTGACATCTTCGCAAGGGTTAACCTTATAATAATTAAGACCATATTCACTTTGTACGACAAAGATGTCTATGGAACCAGATGTATCTAAAATAGCCAAACCT
**CoV-bait-3688**	ATTAATGCAACCACCATCATAGCAATCAAAATACTTACACACAATTTTATAAACAAATTGCGCTTGACAGATGTCTAAAACAGTAACTCTGTTATAACGATAATAATTAAAATCTGTCAT
**CoV-bait-3689**	CATTTCATCTCTGTAAGTTTTGACTGTACCGTTGAAATTTTGATGTTTCTCTTACCTCCAACACCAATCAATTTAGCACTAAGAATCATAGCGCCATATGCGTTCTTAGGTGCAGAAAGG
**CoV-bait-3690**	ACAGGGTGAATAATCGCAGCTCAGTGGCAACGTACTAATTGTCAACAGCATGAGATGACCTACAAGCTCTTCAGACCATGAGTGAATGCGTTGTCCGCGGTCGACGGTTACTTTTGGCAG
**CoV-bait-3691**	ACCATTTGGTTTTGTGGAAAAACACAAAGAAACCAAACCTAGGTAGTTGTTTAAATTGTTGTAACTAAAAAGACAAGTTCCAGCTTGTATGTCAACAGCAGTACTGAAAGCAGAAAATGC
**CoV-bait-3692**	AGCACATAACCACCATAGAAATGAACTACTAACACCAGCACCCTTCTTGTTAACAATACTAATAGCAGGTAGTTTAACATTCATAGCATTTGTATTAAATGTCAATAAGAATGTAACACC
**CoV-bait-3693**	GAGAGTTACCAATCTTACGCTTAGCATAGAGTTCAAAAGCTATATTTGTCGGTAGAGTGGTCTTGTTAGTAAATATAACATTATCAACCACACCATCACGAACAAAAACTTTGTCAGACA
**CoV-bait-3694**	ATTTGTAGAAATGTTGAAAATAGCACTTTCAACGCGATCCACAGAAAATATCCTGTAGCCATTCAGGTGAAAATCACCATTACGCATAATTACAAACTCTTTAACCTGTGGTGGTAAAAT
**CoV-bait-3695**	AACTGACACACCATCAACCTTAGAGACTCGTGCTATATAAGCATTACCATGCTTACAATAGCCGTCATTACAACCACTCCTTAAAACACTAGCATTGACAACAAAGCTATTAATGCGATG
**CoV-bait-3696**	TTCTTCAATGGAAGACTTACCATTGAAATCACAGTTGACACCCAAGTCAGCAACTAAAAGTTGTAGTGCTGATGGATCAAACACTGGCCATTGAGAAATCATAACAGTGTTAGTTAAGTC
**CoV-bait-3697**	GAGCAAGTCCTCAATAAAAGACCTCTTAGTTGGCTTTAGAGGGTCAGGTAATATTTGTGAAAAATTAAAACCACCAAAATATTTCAAAGTTGGGGTTTTGTACATTTGTTTGACTTGAGC
**CoV-bait-3698**	TGTCATCATGAGGCTGACCCCATTTAAGGTCAGCAAAAGGGCCTGTGCCAGTGTAATAAAAGTACCACGCATCTGGGACTGGTTTTCTTCCACCTTTGCCTGGCTTATACCTGGCTTGGC
**CoV-bait-3699**	GTCAGTAGTTTTACTTAACAAACCACTTCTTTTCAGAAAATTATAAGTGGCTGTTATTAATTTTGTGGTGTACTGTACAACTTTTCCTGTGGCAAAGAGCACAGTACCCAGTAGTTTAAA

**CoV-bait-3700**	GACCACACTGATAGTTACCAGTGTACTCATTCGCACATAAGAATGTACCTTGCTGTAATTTATACTCAGCAGGTGGTGCAGACATCATAACAAAAGAAGACTCTTGTTGTACTAGATATT
**CoV-bait-3701**	AATATGAAAACTAGCTGCGCCAACAAAACCATAATGCACGTCCCAATCAACAGCAGGAGCAACAGTAACCTTAGAAATATCACTAGTTGGACGCTGATCAGAGACATTAGTGTTATCAAC
**CoV-bait-3702**	AGCGCATATGATGAGGCACACACATAGTGGTACTATTCAGGTATTGGCACAACTGTGTATACTTGACAACATTAAACATAATGCCGTCAGGTAACTTAATGCCAGCACCATAATTATACA
**CoV-bait-3703**	TACAAGGAAGTAGGCAACAAAGTAACCTATGTGCCAAATCCAAGCATAACGCACGGTCTTTGTGGAGAAGAAATACAAAACAGTGTAGAAAACAAGACCCATATAATTCTGAGTTATTAT
**CoV-bait-3704**	ACACTTAACAAGCGCATGCAAAAACACAGGTTCAGTAAGAATAGATGCAAGTGTATCACCATTCTTAACAAATGGTGCACCCAAGCCCTCTAACAAAGGCTTGTAAACATCTGAGAGAAC
**CoV-bait-3705**	GATGACTCTACTCTAAGTTGTTGAAGAACCGAGAGCAGTACCACAGATGTGCACTTTACGTCAGACATTTTAGACTGTACAGTAGCAACCTTGATACATGGTTTACCTCCAATACCCAAC
**CoV-bait-3706**	GCACAGAGTAATCAGCAACACAATTAGAAATTTTTTTCTCTCCCATGCATAGACAGAAGGGAATTTAGTAGCATTAAAAACCTCTCCAAAAGGACACAAGTTTGTAATATTAGGGAATC
**CoV-bait-3707**	TGCACTACCAATGGCGTTGTCCAAAGCATTTAGAGCCTCTTCCTGTTTTGCAGACAGATCTTCATCTAAAGACACACCATTAGGTTCGGTCTCATGACTTTCATCTGTCGATGCACTATC
**CoV-bait-3708**	AGGAAGAGGTATTGTTCTAACGTAAAAACACTGTTAAACGATACATGGTGTCCATATTGTTGCGGTTATCCCTCGACGCCCACGAATAGGTAGACTCCAGCGACTCTAGGCCTGAAGTCC
**CoV-bait-3709**	ATAAAGAAATGCTACAACATTCTCAGATACAAGGTTACTAACACCTTCTATCTGGAAAGAGGGTTGGTCTTCATATCCACCATACATAGCACCATTCATGTCGCTACCCACATGACAACC
**CoV-bait-3710**	CACAATGTCAACAAAGAAGAGTTGACAGCAAGCTCTGTTTGTTTACCAGCCTGAAGCCTCACAGTAGCACCTATAAAACCTAATACAGCACCACGCCGCAAGGTATTAAGATTTTTGACA
**CoV-bait-3711**	GTGGTAGCTTTGGCAACAGTTACGAATTCAGGTAGTTGTCCCACCGTGATTCCGTTAGCAACCGGTATGCCCTCCACAAGGAGCTTTCCACTAAGAACTGTCAACGTAATGCCAGTCGGA
**CoV-bait-3712**	TCCATCATGGTATATTTTGTAAGATTACACCTACAAACATTACCATAGATTGACCTACCTTCCTTCCAGGTGAAGAAATCATGTTTTGCAACTGCATCACATTTTGCAAGTTTGTCATAT
**CoV-bait-3713**	TATGAATGTTGCGTATAAACCTTATACCCCGGCACATATATTTCAGACCCTAATATACACAACTTATCCTGATCCCATAGATAAGTATTATTACACTTTAATTTAAAGAATGGGTAGTTA
**CoV-bait-3714**	AACTGCTAAATAAGTAACACCAGCTACAGCTTCAGAAACATAATCAGTACCATCAAATAAGTTCAACTGTCTTATCTGATCTTTACTTAAAGCTCCTGTGTATAGTTGGTCCATATCATA
**CoV-bait-3715**	AACCACCAACCATTAAAATAAAAACCATTACATAAAACCAGGGTAAAATAAACAAACTCAAAATTGCCAAATACAATAAAAACACAACACTATCACATAACCAAAGTGCCAAAACAAATA
**CoV-bait-3716**	AACCAAAAGAAGCTTAAATATTGAACATAATCACAATCAATGCCTGTAATAATTGATAGGAAAGCACAGAAATAGTGGCTAACAAAATGTACCAAAAAATGCAGGTCCATTATGTTACTC
**CoV-bait-3717**	CAAAATAGTGTGATAAGCATACCCAACAAATTGTCCATGCACTCTCCAACATCATCAGATGCAAGGATTTTATTGTGTAACTCAACAAGATAAGCATGCATTTTTGAATTTGCTTCAACA
**CoV-bait-3718**	AACTAGTATACCACGCTTAGCTCTTGTAAGCGCTACATTAAATCTGTTAATATTCAGTGCATGTTGAGAATCTGCAGTTACACAGAAAATAACATAATTATACTCTGAACCTTGAGACGA
**CoV-bait-3719**	CTAAATATGATGCCACTTCCAGTCTTGGGCATTACACCATACTTTCCAGAAGGTGTTGTTACACATAGACCAGGCACTGCTTCAATAGTAACATAAGCAGTGGGTTGGTACGTGTAGTGG
**CoV-bait-3720**	TTACAGCCATACTTGGCATCTGTAACATCAAAGTTATAGCGCCAAAAGGTTTCACCACTATAAAGATAGTAGAACCCATTTTCAAATGAAACTTTATCGACTTCGATAAATGAATCACCT
**CoV-bait-3721**	TGAGTAGTAAGTTGGCAAGGTGAAATACTAAAGATTTCACCAGTGGTAGAGTTCTTAAAACCAAGCAAATTACCAGTGACAGAAGTATAAAACAAGCCAGAAACAAAACTGCTATTCGAC
**CoV-bait-3722**	ATATTTTGGGTTTTCTATTGGGTCGTACCAATCCTTATTCTCTTCAAACCACTTAGGATGATAATCTTCTATACAACCATAAGTGACAAGTATTTCTTTAAGAACTTCGCAATCCTTTGG

**CoV-bait-3723**	CCTCAAAACAAATACTCTTCCGCGCTCTTAAAATAGAGGCACTATTGAAGAATAATTTTAAATACTCTCTACACACACACATTTATGTGTAAAGGCTACTTCCAGACGTTGCTACACTTT
**CoV-bait-3724**	AGCATTTTAATGCCATACACGAACCAGCTGAATTGAGGTCTTCCATATTGTAGCACAGTAATAAAAATAATCAATATTATAGACCAGCTGAAGTTCCAGTTTGCAAGATGCCAAATAAGA
**CoV-bait-3725**	TGTCCATCTTGCTGGCCACCCTGACCAACTTGTTCAACAGGTTCAAACTGTTGATCACATTCAACATGCTCTTGACTAAAATCATCTTCACACTTTTGATCACACTCCTGAACAAATGTC
**CoV-bait-3726**	CCGATTTTAGGGATAATCCATAGCTCAAGATCAGCGCACTGCTAATCTTGTTGACAACAAGCTTATTGCTGCTAATTTTTAGCCCCAACAGCTTGCTTAAGTGGTTTTCTTTCAGCAACA
**CoV-bait-3727**	TAATATAAAAGTGGGCAATAAAGAACAATGACAGCAAAACGCGCTGCCAACATAATATAATTGCGCCTCAAAGAAGACGCTTTAAACAGTGCAAAGAAGTAGATAAAAACACTGGTGAAA
**CoV-bait-3728**	GTGGAAGTAGACTTATAACTAACAGCGTCATTGTCATATTCCAACTTTTCAAATGTAAACTCACCAATTTGAAACTTAGTGTTCTTGGTGATGTGATAACATGTGAAAATAGAGTTCCTA
**CoV-bait-3729**	ACTCGTCATCAGCGAATGCGTCTATATCAATAGGCCGCACATCAGCTGGACAATCCTCAAACAGTTCGCGCAGTTTTTCAGCAACCGCTTCTTGAACAACAAGTGCAAGGTCTTCAAGCT
**CoV-bait-3730**	TCACTAAAATCATAGTGCAGCAAGTCATAGGTTTTAAAATCATCACCAAAAATGTCACTTTTCATAAAACATTCTCCTGCAAGACAATTGGTCATACCCATAACTGGCATCATGTATGAA
**CoV-bait-3731**	TTCGCACACAAAGGGAATGATATTGGTGGTCTGTGTTCTCTACAATAATAAGACATGCCTCCCAAATACAATTGGGTTACATCTGCAACATCACAACTAGGGTGGTTACAAACATATGGT
**CoV-bait-3732**	TAGGCTCAACACCACTGAAATCATAAAAATTATGAGTACCAATAGTGCGAAATGGAGGAACAACATTAACTGTTGTCTCCTCAATTAAAGGTGTTCCTACTGTCTCAACAGATACAACAG
**CoV-bait-3733**	AGTAATGTCATAGTTATAGCGCCAAAATGTGTCACCACTGTACAACCTATAAAACCCGTTTGAAAAATCAACACGGTCTATTTCAATATAAGCTGGACCTGTAGGACGCACTGACGTCTT
**CoV-bait-3734**	CCTCCTAAGTTAACAACTATACCTTCATCTCTTGGGGGTTGGTATTCACATTCCTCTAAAACAACATCACCCCGAACGTCATCTTCAAAATCAGAAACGTTCACAGGCACTGTGGCACCT
**CoV-bait-3735**	ATGTTTAACCCAATCAAGAAGAGCATAAAACACTCTTTGATATGCAGGTTTGGGGTGTTTTGTGAGTGGATATGCATCAATTGCTAAAGACACATAACGTTCAAGCATAATGACGTTGTC
**CoV-bait-3736**	TAAACATCCTTATCAAAAGTCGAATTTTCATAGCCACTTATAGTGTCACTACAAAAAAGACTATTAAAAGGATTAAGTTGCACAACCATAAAAATAGAGGCGTACATGGTATATAACAAC
**CoV-bait-3737**	GAGCCCCTCCTTTTTTCTTGCTTGAACCAGAAGAATTGACAGTAGATGCTACATTCCCAAGTCCACAACCAGGAAGTTCCTTTAACCCAGAATCCATGTTCCCCAAACATTGCAGATTCT
**CoV-bait-3738**	AGCGGGACTAACTTTAAATTCATAAAGTCCCATTGTGCACTTAAAGAAGCGGTTAAACCAGTAGCATATGCCATAATACATACAGCATAAATAACCACAAGTAACATACATGAAAAGTAT
**CoV-bait-3739**	TGCCAGTACTAAATCCTACTTGCAATGGGAGGTTAGTGCCGACATTTGGTTTTATCGCATGTGCTGCTTCTACGTCTATGCCGATCCAACTCTGAACATAGGCGCGACATGCATCACGTG
**CoV-bait-3740**	TCGAAAATGACGCACTTGGAGACATTGCCTCAAACAAAGAAAATTGCCTCTTAGGGGTGGAAAACGCGCAATTTCGTACCTTTGTGGCACAGAGTAAATTAAGAGTTTTGTTATGCCTGG
**CoV-bait-3741**	GCAGAGGTGTATTGCTGAAGTAACTGTAAGCAGCGAGGGTTGCCATTGCAAACATACTGCCGGCAGTCAACTACAACAGGCTGTTGCTCGATCTGTATGTATTCAGTTTGAATAGATACA
**CoV-bait-3742**	AAAAGGTTCCAAAGAAAAACAAATGGATTCAAAACTAATGTAATTGTTAAGCTTATCAAAAGTAAAAGGACAGTCAACATTAATAATTCTGGCATTGAAACCAGTAACGTCAGCAGTGAC
**CoV-bait-3743**	GATAACAACAGTCACAAAGACTATATATGTAAAGAGCAAAGTGATTACTTCATATATACTCTTAAAATTCATTCTGTTCGTGATCTCTTACGATACAACTAGCAGATCTTCTTGATCTTC
**CoV-bait-3744**	ACCCTTGATGAAGACTGGAACAGTCTTGCAAGCCACGGGGAGAAGTCTACCGCCTTGCTCAACAGCCGTCGAGGAAGGGTATAGGTAGAAATTCAGAAACTTCATGGCAGCATTGACAGG
**CoV-bait-3745**	CGTCACCCATTAAGGCCATTCGTGACCGCAGTATCGTATGGTCTAGAGTCCATTTGGTTATCAGAATAAAGTATCGCAATGGATATGTCGATGTGTTGTCTCCGGGACCTTACGGGATAG

**CoV-bait-3746**	GTGCAGTGGTGTCAAACATCAACTCACCAAGCTTATGTAAAGTCAACTCAGCATCACCGG GTTGTCCTTTCTTTAAGCCTGAGACGTGATATAGCATATGGACAAATCCAGCAGTTTTAC
**CoV-bait-3747**	GTAAGGTGATACAGACAAGACCAGTTTGTGTGGCGTTGAAAGGACGTGATCATAACAAC ATTTGCAACACAAAAATGGACGTCTAATACAGTCACCGCAACGCAAAGATGTTTGTGAAT T
**CoV-bait-3748**	GATAACCTACTTAATTTTTCAGTACTAATAAAAACACCATTTCTTGCTTTTCTAAAAGCTTC TAAAGCGCCAGTGTCACGACCATCAAAAAGTATATTCAAATTTTCGATGAACTTCAAA
**CoV-bait-3749**	TGGTGACCCGTGCGACTCACTACTTCACACGTAGACCTCAGTACGGGGTCCGACTTTGTG GCCTGGAGCCCTATTGTGCATATAACGTGTGCATTCGGTGACTCGCAGTTTAGGGGCGG G
**CoV-bait-3750**	AACCTCCTCCTTGACATTGGTATGGGATTGTCAAATTATTAATGCCATCACCAAAGGCTTT GGAAAGAATTGCTAGGTCTATTAGTCTAACACCCCAAGTAGTATGTGGGAGCACACCAT
**CoV-bait-3751**	CTCCAACATAACACCTGCTCCAACCTTAAGTGGACCATTACTACTAATGTAATCTTTAGAT AATGACTGAAGTTGACCTTCAGTCAAAATATCAATAGCACGTGCAACACCGCCTCCATG
**CoV-bait-3752**	CACCTGTAGTCTTAAACTGATCTGACAACGACAAGAAAGTGTGTGCATGAGTTGGTGGT AAATTAAGAGGAGTGCGTGTACAATTTTTAAACAAGCCACAAACCTGGCTAGAGTGTAA AT
**CoV-bait-3753**	ACTCAAGAGTAGTAGTAATAGAGTTCTGTTCAAAAACAACAAAAGTTTTATTGACTACTA TTTCAGAATTAAATGGATACAATCCATCAGAAAAATTGCCTGTGTTATATTGACAAGCTA
**CoV-bait-3754**	TGCATTAACCCAACAATTATTGTCGGTGGTCCCAAGAACTCTAAAACCACCAACAAAATC ATTAGGGTAACCATATGGCTTTACATCCAAAGAAGCAAAAAGTGCTGCCTTTTCAAAACC
**CoV-bait-3755**	CAACACACATATAAATGGTACAACTCTCAACAACAAGTTTAACCAAATTACCAGCTGGTA CAGTTTTAACTGTAGCAAGATAATCAAGAGCTGCCTGTTGCAAAATACCATTAAGTTTTG
**CoV-bait-3756**	AATAACATATGCAGGAGCTGTGGGTTGAACAGCTGTTTTAACAACATTACCAAGCTCTCT TGCAATATCACCATTAATAAAAGTATTACCAGGCCCAAAAGAATCACAATTAACACAAAA
**CoV-bait-3757**	ATCCACCTTAAACTTTTGATTTTCGGCTGAGTCACCTGTAACAATGGCTTCCCATGTAACA ACTGTATCTCCTATTTTAGGATAGTATCCCGCACTAAGAGTTGTGGCATTGAAAGTTGG
**CoV-bait-3758**	GTGGCAGAATTACCACAATAACAATATTTAATTGGCCCTATTTTTACAAAGTAACGCATTG TAGTTAATTCCAAACTACCTGCCCACAAAACAAAGACAAGAATGTCAGACAAATTGGAC
**CoV-bait-3759**	TATCTTCAGGTTTAGCATAAGAAATAAAAAAATTATTAAATGACAAATCTGAAAGCAAAA CGTCATACCTATGTGCATTGGCAACAGCTGAAACAAAATCATCATCAGAAACAGTCAAAC
**CoV-bait-3760**	GATGAACTAAAGATTGAGAGTACATTAACAAGAAGGTCTATAAGACCATCACCACAAAT GTAACCGTCATCAACACGTCCATCATTAACATACCATTTATCAAAACCAAAACAAACACCT
**CoV-bait-3761**	AATATAAACCATAAGGCGCATTCTGGACTAGTGATAATATATGATTACCACTGCCGCAGA AATTAATACGCATGGTTTGACTCTTAACGCACTCATTGACCTTTTCTATGGCCTGAGCAG
**CoV-bait-3762**	TCCCAGTGAGGCATGAGACAAACGTAGAGAGCGTTCGGGTAATCGGTAAAAAGGTAGG CTTCGTACAGAGGTAGAAAGACCATCGCGCCTGTTCAACTTTGTGCGGCTCTATAATAAC CG
**CoV-bait-3763**	CCCCACGAGGTACTCTCTCCAGCAATTCGTAGAGTTTTCCCTCCTATTGATTCGCTTCCCCT ATAATTGTGGAGTGGGATCCTGGACGTTTTTGTGTTTAATCGGCAATGGTGACTAAAA
**CoV-bait-3764**	ACAAAACACGGGTACACACACCACTAGCCATGTAGGGCTGTCCAATGGAACAACCAGGT TTGCCCCCACCGTGGCATGGGTAATTACCACAATTATCCACTCTAACAGTGCTGTACCAAC
**CoV-bait-3765**	GCAGCAGAGAGAAAAGCTTCAGCACGTAATTTCACATACACATCTCTTGTGATCATGAAT GTGGATAATGCGGCCTCAGCAAAACTGCTAAATGTAGCATCATGTACAACAACATTTTTC
**CoV-bait-3766**	GAAACGTAATCTCTCATATCATTATCAACCAATATGGCATCATCTGGCAACCATCTTTTTA GTACAGTGGTACCAGGTGCAACACCATTAGCACCTGCCGCTCCTAAATGCAGAACACGC
**CoV-bait-3767**	ACATGATGTACACCACCAATATCAGTGCCAACTTCAGTAAAATCACCTGCAAACACATGTT TATTTTTTGGTATAGTCTCAACTGGTAACTGATGAACATTAAAATTTAAAGATGGGTCA
**CoV-bait-3768**	AGGTCTAGGTTAAAATCCTTAGAAACATATTCAAATGTGCAATTGAATGCATTATTAAAT ATGTAAGATGGTATTTGAGTGTTGTTAGATTTCAACACAACAAAAAATGGATTGTCACAC

**CoV-bait-3769**	AAGAAACTCCACACCTTCCTTGAATTTTTCTTCAAGCCAATCAAGAACCGGTTTAAGTTTTTCATAAACAGTGCCGAAGATATTTGTTAGCCACTGTGAAGTCATTTGTACGACACCACC
**CoV-bait-3770**	AGAAGAATTTCTTCCTCTAAGTTAGAACCCATGTACCACAAAGAGACAACAGAAATCACACGGCCAACAAACATTATAAAAGAGAACAAATCATGGTTAATGCTACGTAATGTAACAAAG
**CoV-bait-3771**	GCATTATACACTGGTATGTAAATAGTATTACTCATAAGCCTATGACAAGAAAAACATAGCTGTATAAGTTTAATAACAGTGATAGCCACCAAGAGAACAAGTATCAACACTAAAAGCCAC
**CoV-bait-3772**	AGACACACAGCACCGTTGACAAAGCAAAGTGTAGGTGCATTAAAAAGTACAAATGCAAAACTCCTGTAGCAAAAAGCATAATAACTGGTATACAGTAACCTGCTCACAACAGCAACAAGA
**CoV-bait-3773**	CCTGGGCAGCATTCTGATTAACAACAGCTTGAATAGCCTGTAATGCACTAGAAGTAGTCAAAGCGAGCTTTCCAACACTGGCAGTAAGCTGATTAAAGCCGTTAGCTAACTTATTAACGT
**CoV-bait-3774**	AATAGAGCTTGTCTTAGCAGCAATAGTGGAATACAGACCACTAACATCACTGTCAAGCAAAACACCAGCACCCCGAATGTCAGCCTCAATTTTCTGACAAAAACCACCATACCTAAATAA
**CoV-bait-3775**	CCAAAATAAAGTACATTATAATACTGGGCAAATGTAAAATTACTCTCATAGAGAACACCCTGAAACGTGACCTTCTCAATAGTGTAAGTAATACAAACACCAGTTCGTGGCGCATCAGGA
**CoV-bait-3776**	ATCGGCGCAATACGAATACCTAGTAACAGCACCACTTTGATCTGTTAAAACTAAAACACTAGAAATCTCTGTCACAACATTAAATTTGGTCATACGCCCAGCATTAACTGGACCAGTAAA
**CoV-bait-3777**	GGCGTCTGTTTCGGGGTTAAATGCCCAAAACGTTCGCGTTCTGCGATAAAGCTTAAAACTATTAATTATGTACATAAGCCAGAGAACTAGCGTTATGACAAGCATCAGAATGCTGAAGGA
**CoV-bait-3778**	CGTTGGCCTTTTTGCATTCGCCAGCGTTTTTGTTCAACCCAATAACCAATCTGTTGATCTGGATTGCCTCTGCCTGTTGGAACTCCATTTCTAGGCATGACATTCCAAAAGTTTTTGCCA
**CoV-bait-3779**	AACGACGGCCCTCATAATTCTATCCAGCTGCGGCGGCGCACAGGGCACTAGAGGTCTAGCGAGGCGATCAAAGGCGGCAAAGTGGAGGCCACGGTCCATGGTAGGTATCACCTACGATTA
**CoV-bait-3780**	CGCCATTACCAGGTTGTAGTGGACCATGCTCGCGAATGTAGGCAGTTGATTCAGCCTGCATTGCGTTGTCAGAGGCGCGGTTTAGAACTCCCGCCACACCGCCGCCATGATGTAGGTTAA
**CoV-bait-3781**	AGAAGCCGCCCAAGAAGTTTAAAAAAACCGATGGGCTGCTTTGTTTTTCTAATGGGTTGGGTGGGTTGCATAGAGAACACAAGAATTTATTGTGTAGGCATGGTGGGTTATCTGTGAGTT
**CoV-bait-3782**	AGCAACAGTGGCAAGACCTTGTGATGTCTGATGTATAGCATCATTAACCTTGCCAAATGCCTGTGTAATGTTACCAATAGCTTGGTTAAAAGCATTAGCCAGGATCTGCTGGTTCTTGTT
**CoV-bait-3783**	TAATTCTTCAACCACACTGTGTTAGAGATTGAGTACAATCCCTGTAAGCTAGTGTACTTAAAGAAAAAATGCAAGTTGGCGGGTCTGGACACAGAAATTAGTGACATTCTTAACTTCACCA
**CoV-bait-3784**	AAGTTTGTGTATATATAACAAAGTCGTACTCACTACCCTGTGAGGAGTCAACAGTCTGTGTCTGTAGACCCAAAACACGACTAGCTACATAATTCTGACTATTATAAGGTGAAATAAACA
**CoV-bait-3785**	CACCCTCAAAAAGTTGTGTTGAAACCTTAAGCTTAAAAAGGTTAGGCATAAACTCAAAAATGGCTGAAAAGGCATAAACCATCAAAACCCACCATGGTGCAATAAGTATATATGAGATCA
**CoV-bait-3786**	TTAAGCTTTATATATATGGACTGCTCGTGGTCCATAACACATTTAGGACACCTCTTGATAACATAAAAAGCGTCATGCTTATCCAAATTCCTAAAGCGAACACAATTGACCTTAACAAAT
**CoV-bait-3787**	TTACAAACATCTTTTGTAAAAGTAGTAAGTGGTCTATTGGCTTCATAATCCCACAATACACATTTATGTGTACACAAAACACCTAAATTCTTAAGTATTGTAAGTGGTGGGGTAAGGCCA
**CoV-bait-3788**	TGATTCTGCAAATGAATCCAAGCGAAAACATAAACAGAACCACACCAATAGTTGCTTTAAGCAGCAACGTAGGTGTGTCTAGGAATAATCCCAGACAAAGAAAGCCTCCAGTTAGAAGGC
**CoV-bait-3789**	CACCTCTGGTTGAAATATAATAAATCATGGCATAAGCTAACATTCCAAGAAAATTTTGAGTGACAACATAGGAGACATTATTAATTAAGGTGCAAACACAAACAGTGAAAACACCAAATG

**CoV-bait-3790**	TAGTAAAGTTAATAGGTATCTGTAGTGTTGAGTTATCAAATGCCACAGACTGTTCAGACTGAGACGGGAGTTCTTGTGCAAAATACATGTAATTAAATGTTAGGGCTCTATCGGCAGCAC
**CoV-bait-3791**	CAAGGGCTGCAGATGTAGTGTAAGCAGCTACATGTGCATCAGTCATTTGTGGTGGTAACACCATTAAACCGTTAAAACTTTGAGCACAACCAAGGTCCTTGACAACAGAACCTCCAGTGC
**CoV-bait-3792**	AATAACATAGATTGCATAGCAGATGTGACGCGAGCACGTTTATCTTCAGCACGCGCTTCTTTGTACATTTGTGTCATGGCAAGGTCTGCCATACGCTCTAACTTTCTTGCCACAGCTGCA
**CoV-bait-3793**	CACACCCAATATGAAGGAAATGGATATTATAATTGTAAAAACCATAGTCCAAAAATACGCAACATCATTACAAGGTGATTGCGTTATGGTCGTATAGTTGTTACAAGGAGGTAAGGTACT
**CoV-bait-3794**	TAAACCACCGATCTGTTTGTGACTAAAGTCACCATAAACAATGTGCTCAAAAGCATATTTTTCAAGATTGTAGCGTTGAATAAATCCATGCATGTCGAGCTCTAAGAAGTCACGCTCCAT
**CoV-bait-3795**	TCATCCGCTGGAACTTGCCTGTCGCTCCTGGTCTGGCAGTCACGATCAGGTCTCAACGGAACTGTTAGATGTAGTTGCCCAGACTCGGGTAGGTTCGCGACGCTGAAACTATTCGAATGG
**CoV-bait-3796**	GTGCACTAACGCAAAGAATTGCGTTAGTCTCAGGGTTAAAGGACCACCAAGACTTAGTCCTTCTGTACAACTGAATGGATCTTACGAAATACATAATCCAAAGTACAACTGTAACAATTG
**CoV-bait-3797**	TTGTCTTTTGGCCATTCCTAAGATTTATGGACTAGGCCAATTTTTCTCCTTTAATCAAACGATCGATGGTGTTTGTAATGGAGCTGCTGTGCAGCGTGCACCAGAGGCTCTGAGGTTTAA
**CoV-bait-3798**	TTCGGTATCACGGGATCCAAAAACCTGATCAACAGGCTTATTGGGTGGCAAGGTTCGCTTGCTATACGCACGTGATACCATCAAAGCTGCCTTCTCTTTGGTAATTTTACCACCAGAGCC
**CoV-bait-3799**	AAAGACTCTTAACTTTTGTCGTGGAAATGTAAACGCCATTATTAGAGCGTTTAAAAGCTTCAAGAGCACCATTATCACGACCATCAAAAAGGACATTCAATTTATCAATGAACTTTAAAT
**CoV-bait-3800**	ACAATACGCAGAGACAGGACACTCATAGCTATGACCACAGCAAAAAGAGCGATAAGTTCTCTTATAGAATAGCCGTAGAACAGCTGAATTGTATCAGTCAAGTCAACATACTTGCTGACC
**CoV-bait-3801**	CTGCAAACTCAGTAGCGCTAACTAGGGCTCTATTAAAAATAGGTCCTAGCTTTTTATAAACATTAATAATATCAGGATTTTCAACAAAATCATACCAATCCTTCTTAGTAAAGTAGGATT
**CoV-bait-3802**	TAACGGCTTGTGGCATCAGCTCGTTATTCTGCAGTTTAACGGGAGAGGTAGCAACCTGCCTGACTGCGGTTATAACCAAAGGCCAAGCAAGGTTAGCGCTATTCTCACGCGTTATGTCTG
**CoV-bait-3803**	GATAGTATGGGACAAGCGTAGCATAAGCGTCACTAATATTGAATGTAGGTTTCAATTTACAAATAGAAGCATAACGTTCCTGGTTTGCAATAGTGGGAGCTTTGAGAGGCTGTATGTTAT
**CoV-bait-3804**	CATTTGTTTCAGGAGCTACATTAGGACCATCATCCTCAGGCTCAGAGTTAGCCACATCTGCCACCTTATCAGGGTCAGAGGAGTCTACTTCATCACCCTCGAAATCAATTGGTGCATCAG
**CoV-bait-3805**	TTCATTTTTGAACGTAACCTTAGGAGTTGAATTGCCAGAACGGGGCTTATTCCGTCCAGACATAATTGGTTCGTTCAAATATAGAAGGAAATATATACAACATGTGTTTTATTTGTTTAA
**CoV-bait-3806**	AATCTTCACTAACAGAAGAAGTTCTATAACAGTTATCATAGATTGCACGCTGTAAATCTTTAACAGCAATATTCTTACAAACATTGCTGTCAACAGCTAACAACCTATTAACATTTGCGC
**CoV-bait-3807**	TCTTCATAGGATAGTGACTCGTAATAAAGACCTGCCTTACCAAACTTGTTAAGTGGCCAACCTGCACTCTTGTTAGGATTGGCAACAACAACGTCTTTAGCAGTAATACAACCACCCTCA
**CoV-bait-3808**	AATAGACATACAATCTGAACATGGGCATAGGTGGTCTAGCATGTAGGGTAAAGGGTGACACAACCTTTGAGAAAACACTCTGAAAACTGAAGTTTGAAGGCTGGTAGTATGCTGTTTCAC
**CoV-bait-3809**	TCTATAGACACAAGCTCAGTTGAAACTTGAACGTACTCCATTTGAACTGATATTGTGAAATTACTAGGGATTGTGACATTGCCAGTGCTAATAGGTTGTACATCACCATTTGTGTGAGTG
**CoV-bait-3810**	GTTTCTTTCTGTGGGAGCCACTAACCAACTTAACAAAGAACTGGTCTTCAACTTGCTGGGAACCAGTTTTATTTAGTAATGTAAGTATACCTACAATATTACCTCCAGACACCACAAATT
**CoV-bait-3811**	TTGGAATAAACCCTGTTAGCGGGCATAGACCGTTACCGTGTTTAAAACAATGCGTAACGAACACTGTAAAATCCGAGAAGTTACAGTGAGCCGTGCAGAATTGATTAGTTGACCACGTCA

**CoV-bait-3812**	ACACTCATTAATTTTCTGAGTAGCTAACTCACGCTGTTGTGACACTCTAATATACTCCGCCTGCTTAGCAGATGCTAAAACAGAAAGTGATGACAATCTACCAGTTATAAGACGATCCAC
**CoV-bait-3813**	GTTAAAGCATAATGTGCTTTGCAGGCGAACTGTACTCCATTAGTGAAGCAAGCACGTCTACCACAATAACACTGCTCTTCAACGCCAATTTTAACAAAATAACGCATTGTTGCGAGCTCA
**CoV-bait-3814**	GTACAACAAAGTCAACACCATTAGAAAATCCTAATTGTAAAGGTAAATTAGTACCCACGTTAGCACCAACAACATGAGCACCCTCAACATCAAAACCTAACCAACCACGTACATGACGTT
**CoV-bait-3815**	TTGTAAGTCAGAGGATTTAATAATGCCTGTGCCTGAAATACCATAAATGGTATAATCAGTGCACACACCTTCAATAATAGAAGATGTGTCAATAGCATTAGGAGTTGTTGTAGAGGTGCC
**CoV-bait-3816**	CTGTCGGCCCATCACAGAAGTAGTGAGAAGCGCATCTGTTTCAGGATTGAAAGACCACCAAGAATGTGTCCTGCGCCACAACCGAATGCTATTGACAAAGTACATTATCCACAGCATAAG
**CoV-bait-3817**	ACCCTCGATCGTACTCCGCGTGGCCTCGACACATTAAATGGTATAATTGTCCTTCTTGCACTGACAATTATCGAGATGTGATTCTGTGCAAGAGTAGCCAGCCCTAGTTTGCATGGTAGC
**CoV-bait-3818**	ACTATTTCATTACGCCAACGAATGTATGAGGCATGCAAATTGTAACCATCAACGTTCTCTTTTGCATGTCCTAAGTAGTTAAAGCAACAGAGAAATGCTTCTGAAGAAGTTGCATTTACT
**CoV-bait-3819**	CAGTAAAGCGGTTTATGATCCACATGAGACCAAATCGCATACAAATAACATAGCCTATAACTGTGTAAGTAAACACATGCACAGCTACATCAGGTACAAAAAGTGGTAGTGGTATTCTAC
**CoV-bait-3820**	CCTTGCGCAACTTTGCTAAGAAGTGGTCTACATCCGGAAGGTCCCATTGCTGAGGAATAGGTCCTTCTTCATCATCGAAGTTTGTGGCAGGCTGATCACAGTAGTCATCATCCTCCACAT
**CoV-bait-3821**	AGCCGAAACCCAATAACATTATAGCAAGGCTCAAAGAGTCATAGCCATAATAGAATGTGTAAAGAACTGTGCACATAGATAATACATAAGTGCTGTAAGAACGCAATTGTGTGTCCAAAA
**CoV-bait-3822**	TCAACACGTATTTTACTAAAGGACTCCAAATCAGGGGTAATAACTGTAAGTTTTGTAGCTGCAGCAGCAGGTATAATAGCCATAGGTATGCAACCGTCTTTTGCAAGTTCCATAAGCTGA
**CoV-bait-3823**	GGAAAAATGCTACTAATGCCAAAAGTTTTTCAAGGGCCTCTTCAGGGTCACTAGTAAGGTTAATCTCATTATGCAAATTAACACAATAAGACCATTCTCTACTATTAGCTGCGATATTCA
**CoV-bait-3824**	GGTCAATACTAGCATCTGGTTTCGCAGTTAATGCAAGACCAGTTCCAGTATGAGTGGTTAACATTTTAACACAGTTACCAATTGGTGTACCACCATTATCTAGGTAATCTTTATAAGTAG
**CoV-bait-3825**	GCTGCATACTCCTTGTTGTAACAAACAACGCCATCATCAGACAAAATCATCATGGAAAAATGCTTCTTAAGATAGTTATAAAAGTCTGTAACAACATTATCATCAACAGCGCTAGATCTA
**CoV-bait-3826**	ACCCATGAGACATGGATTCTCAACATCATCAATAAGTGTGCGAAGCATATTATCCCAACCACCATAAAACTTTGTTGTGCCAATGACAACAGTGGCATTACGCGTGGCGACAATGGACTT
**CoV-bait-3827**	ATCAATGGCCTGTTCAAAAACAGTTCTCATCCGGATCTGACCTTTACCAACTTCATCCGTTTCACGTACAACATTTTTTAGAACCATGCTTCCCCAGGCATCCCGAATTTGCTCCTCCAT
**CoV-bait-3828**	AAAACCAAATTATCAACAGTGCCATCTCTGACCAACACACGGTCATTAACAATGGCAACTGGGAGTTCACCCTCTTCACCAACAAAGGAACCCTTCTTGAGGACATTGAAAGCAATGTTC
**CoV-bait-3829**	CACAATGACGCGGGAGCTTATTGTGGCCGTTTCTGATGGCTCGGACGCCTAATCCCACGGAGAGCCCCGACAGTGTGCTGTGAGCCTGGGTATCGTTGGGGATCATGGGATTCGGACTTG
**CoV-bait-3830**	TAAAGTCCTTATTAAAACTATTATTAAGCACTTCAAGGAAAGCACTATGTAAAGCACCATTAAAATCAACATCCAGAGTGGAAAGCAAAGCACTGTCAACTAACTTAATTGGCTTGCATA
**CoV-bait-3831**	CGAAAAGGATTATCTTTACTATTATTCATACCAGCGATTGGTTGGGAGTATATGCCAACCTATAAACTAAATCTAATTGAACTAAAAGCACTCGCCTAGCACATATAAAGCTGCGTCTAG
**CoV-bait-3832**	AATAATAATTTCTACGTCAATAGGTCGGTTGCACTGTCGGCTAGGGTGGTAACTCCCACAGGTAGCATCGTGTATTTACGGTTTACCTTAAGGCTGGGTTGCTATCGCGGCGGCAGAGAA
**CoV-bait-3833**	CCAGAATAGTTAGAGGTTACAGAAATAACTAAGTGATTTAGCGGGTATACATGAATACTAAGCTGTACACGTACTAATCGTAGGCCGCGAGGGACATCTAGCCCTGTACGCGTCCAACAA

**CoV-bait-3834**	GAGAACTTCGATTCAACATTAGTCCTATCCTGGGCTAGCGCAGCCCACGGTAGGGTCAGGCGTGTACACGTAGGGCCCACTGATTCAGCCTTACAGCATCATCAACATGCCGAGAGGCAG
**CoV-bait-3835**	TCTACAAACGAATCATCTACTGAAGAAGATCTGTAGCAACAGTCATACAACTGACGTTGCAGACTCTTAACACCAATATTATTACACACATTGCTATCAATGCTGAGCAACCTGTTAATG
**CoV-bait-3836**	TACCATAAACATTATAATCAACACACAAACCCAAATTAATATTAACAACATTATTATTAGTATAAACAGGAGGGGATGTGGCAGTTCTAATTCTTGAACCATCAGTCTTACTAATAAAAA
**CoV-bait-3837**	TAATAGAAATTGTACAGCTAGAACCAACAGGTTGTAGATTGAAACAAATACTATCGAAGCTGAGGAAGTTGTTTAAACTATCAAAATTAAAAGGGCAATCACCATTTTGCATCACTGCTT
**CoV-bait-3838**	TCATTTAAACCAGATACCAATAACCATGGTGCCGAGTGTTCCACCAATTGACGGTGTTTTACACTACCACACACACAAGTTTCATTAAAAACTGATTGTAATTCTTCCATCGAATTAACA
**CoV-bait-3839**	CGAGGGCAAAATGCATCTTCAGTATGGACTGTTTTAGTCCGAAATAAAGAAGCTGAAAAAGCCATAAGTTCGTTAATGGCCTAAACACATTGATAAAGATAAGGAATGAATTTAAACTCA
**CoV-bait-3840**	CTGCTGTACCACATTGTTTTACTATATTAGTCGTAACAATGCCAGCAGTTCTTAAACCGCTAACACCGATGTCAACAGTTGGTTGTTTCTTAGGCCATAAATAATTAAATGGTAAGAAAA
**CoV-bait-3841**	AATATCGTTGCAAAAATGTAACATCAGATGTACCGTAATATTCTTTCAGTACAACTTTCTCCGATTCTGATAAATTATCAGCCACAAATAGAGACTCACCTCCCATTTTCACAGTAGGAA
**CoV-bait-3842**	GGAATAATAAACACAAGCTGACCTAGCTAAATTTTCCTGGCCACGATCATTAGTGTCATAAATTAGAAAATTGTGTTCAGGTATACCTGTAGGAGTTTTACAAACCTCTTTGAACTTCAA
**CoV-bait-3843**	CACAGACCCCATTATTAACCCACAAGATAGGCTTGCCTTTAAGCATAACGCCTTTCTTATAAACTGGGTTATAATTACTAAACTCAGATAATAAAACATCGCCATCTTCATTAGGCAACA
**CoV-bait-3844**	TGAATCCATTAGGCAGTTCTTCAAAGAACTTGGCAATGGCCCCGTTAATACTTGCAAGACAAGTCCCAGCAAACTCTCTGACTACGAGAACCCTCTCAACAACCACAACTGTAATTGATT
**CoV-bait-3845**	AATTGAAAAACAAGTCGGCAGCTGCACTACCTCCTTTTCTCATAGCAGCAATGCGTACAAAGCCCTGAGGTATTAGACCTGTTAGAGGGCATTTACCCGTTCCAGCTTTAAAACAATGTG
**CoV-bait-3846**	TGTAGTGGGAGGTTAGTGCCAATGTTGGGACCACATGCATGTGCACCCTCAACATCAAACCCTATCCAACCACGTACCTCCTTAATAGCCTGCTCACGAGTTATAAACAACTTACTATAA
**CoV-bait-3847**	CCACATGTGCACCCTTATGGACATCGCAGACATTATCATGGTTTACCTGTAGATTACCTACATAACCCCATTGTTGTACATCCACCAAAAATGGGTTATACACAAAGTCTGCACCGCCAA
**CoV-bait-3848**	ACGTTTGACTTGAGGGGTACATAATCTACTTCACTGACATAATTAGTAGACACACCAGTAGCATTTTCACATGCTGTATCTGAATAGTAGAAGAATGGGAGTGGTTTCAAATTAACAAAA
**CoV-bait-3849**	AATGGTGCATTATTCTCATAATCCCAGATGACTAAATTATATGTACACGTAACACCAAGGTTACGTAGTAATTTAACTTCTGGTACCACATTTACAGAACGCTTTGCCCACAGCTCGAAA
**CoV-bait-3850**	ATGTATTAACTATAGTAGTATTACGTAGTTCAACATTAACCACGCAACCAGATGGTGTGTCAAATGCATCCTGAGCCGTTGCACTAGCAGACCATAAAACAGATACGGGCTCCGTCACGG
**CoV-bait-3851**	TCCCCTAGGGCTTCCATCGCACAGAATCACGTCCTGGGCTGTGCCATTCACGAAGTAGGCCAGGGCCTTCACCTCCCTCATCACTTTATAGGTGATAGGTCCGCCGGCCTTAAAGTACAC
**CoV-bait-3852**	GTAAGGGTTACAGGGGCATCAAAATCACCATCAAGCACCTCAACCTCCATCTTCTGCTGTTTAGGAGTTAACAACAGGCCAAGGCGCTTAGCACATAGCTGCACTTCTGTGAGCACACCT
**CoV-bait-3853**	TAAATTGCGTACATTATAAACACAACCAGAAGGTGTGTCAAACACACCACCATCAGGTGGGGCTGCGTTTCTAGACACCGATAGCCAATAAGTTGACGCCGGCATATTAACAGCAACTGC
**CoV-bait-3854**	CATGTTGTCCGTTGGTGGCAACACAGTATAATTAACTGTAGTATAGTTAACACTACACGTAGTAAGAACCTGAGTGTTACTATTAGTAAGTCTGACAGGATTATAAAAATTCATTTTAGT
**CoV-bait-3855**	ACCTCATCCTTGTACTCTTTAACTTCGACCCGAGAACCGAAGAAAAGAGCAGCAGGGGTTGGGATCAGGTCAGCTATCTGTGGGAAGTGAGGCGCATCTACTCCAAGCGCAACCACGGAT
**CoV-bait-3856**	CATTACCTGTACACCACTTACCTTCATTACAGTTACCACAATCCACAAAGGCAGAGTGGTAAATATAAGAAGGTTCTTCAACAGCATCTTCACCATACATTGTATACAAAATTTTTGCTT

**CoV-bait-3857**	ATTTAAATCTAAAAGACTTCTTCCTTGGTTCATCATCAGACGAACTATCAGGGAGAAATTGTTTATTTAAAAGAGTCTCCTTGGGTTTTCGCTCTTCCACCTTTTGAGGCTTCTGAGTTT
**CoV-bait-3858**	ATTAGTACGAAACTCATTAGTAACGTTGGTACAGTTAAAAGGGAAATAATCAATATCCCCTAACTCGCCCAACGTTCTATTAGTGAAATTACCTATGCAATTATCCCCTACCATGGTTAT
**CoV-bait-3859**	GTAATGGGTAGTGGGCGGCTTACTCTTCAACTCACCTAAGGACTAGGTGATCCAGATCCTTGGCCAATCTGATTGTTACTCGGTTATCTCTTATTCGGACGTCATTAGAACTGGTCTCGA
**CoV-bait-3860**	ACTGCAAAGCTGGTATATTAAATCTAACATCTCTCAAGCAATCCAACATCATAACCGCCGCATTCAAATAGCAATTATTACTGTTAAGACAGAGAGAAGGTGAACCCTCACACATAGCCA
**CoV-bait-3861**	TTTTACTGTACCTTTTTAAGGTTCTACATCGACCATTGGACCAACTGTAGGCTTAGGAGGGCGTGACCTCTGGGTAATACTACCTTTTGGAACACGCTGTGCCTTCGGCTGGTTGTCTGG
**CoV-bait-3862**	CACCCTTAAGTGTAAACATACATGGTACTGCCTTGCCATTTGTGCACATAGGATAGAATGCTAATTGTCCATCTTCCTCACTACGTACAAAAACCTTATCACTAATAATAACATAATCCC
**CoV-bait-3863**	CTTGGTGTGGAAATAACCCTTCAAGCGTTATTGTGATATTAGAATAAGTACGCCCAACAGGGTATAAAACACCATCTGCTTTACTCATATTAATTGGCATAGACCAATTTTTGATAAAAT
**CoV-bait-3864**	TATTTCTGTAGGTAGTCGCGAATAATCGCACTGACCAAAATGCATACCTGCCACTTTCAAATAACCATTAGTAACAACAGCTGTGACACTAGTAGAATCTTCTACTAGAGGTCTAACAAC
**CoV-bait-3865**	CTAAAGCAAATGTGTTATTAACATTGGTACTACGACTGGAAAAAGAAGGTGGTTTTGCACAGAGAGATTGGCCTAAAGGTAAGGTACAATCTTCAACAGTGTCACTTGTGTTACGGAATC
**CoV-bait-3866**	TAAGAGGGCATACAGTATCAGGGCCTTTATCATATTTAGGCGTTATTACATAAACCATTTGAACGTCGCCCTCATCGGTTTTAGGTTTATAAGATTTACGCCCTAAATAATAGTTGCGTC
**CoV-bait-3867**	TATATAGTTCGTTAAAACCAATCTGCTGGCGGCAGTTCTGGTTTACTATTCTGATACTTTACATAGACAGAATGTCCCATGTTATAAACATATATGGCTGGTTGAACTAACAAGGTATTA
**CoV-bait-3868**	TCAAGAGAAAGCAGTTACCAGATCAAACGAAGTGCACTGACTAGGAAATCTGCTTTCTGGCCGCCAGAATATAGCGGCTGTCTCCAGTGGACCGGGGCAGGCCTCTAGCTAGCAAGATCG
**CoV-bait-3869**	TAATTAAAAGCTGTCTGGCCTCATTGCTAAGACTAATAAAATCTTTAGCATACCATACTATGGGTACATTTTCTTTTATCAAAACATTATGACTAACAACCTTTGCACCAGATTTAATGC
**CoV-bait-3870**	TGGTTAACACAAAAATAGGACATACCACCAAGATAGAGTTTAGTAACATCTGCTTCACCACAGCCTGGGTGTGTACAGATATAAGGATTTATAGAAAGAACATTTTTGTGATTTGTGTGC
**CoV-bait-3871**	CACCAGTTAGTTGAGTACAAGCAGAATTAAAAACACAAGAAGCTTTAGCAGCAACACCAGCCCCGTCAAAGCAGAGATCCGACGCACCAAAAGCTGTATTAACGGCAAAAACTAAAGTTT
**CoV-bait-3872**	TTAACCGCACGCGTTACTATCCTCGGAAGGACGTTTATTTCGGTAGGTATTATATAACTAACACCCCACTTATTGCTTGGCTTTGCTAGAACCGAGTATCAAGCATATGGCTCAAAGGTA
**CoV-bait-3873**	GGTGTAGGTTCAGGTTGAACTTCTGTGGTGACTGTTTCTTCTGTTTGAAGGGTGAGGTTTTCATAGGACCCGAAGTCAATTTCAATTTCTACTGATGGTGTTGGTGATGTTGGGTGTTCG
**CoV-bait-3874**	GACAACATGGTTGTACTCTCCAAACGCACGCCTAAACTTCATAAAGTAGTAAGCAACACATGTAACAAGGATGGCTATAAGGCCACCTGCTACAACAGAAGCTGAAATGTCAAGTGTGCC
**CoV-bait-3875**	GGATTGGCGATAAAAAACAACTGCTGACGCCGCTGCGCGATCAGTTCACCCGTGCACCGCTGGATAACGACATTGGCGTAAGTGAAGCGACCCGCATTGACCCTAACGCCTGGGTCGAAC
**CoV-bait-3876**	TTACTAACAGACTCAGTTAGGTTATAAAACAAACAAGAAGGACCACTACACCCTTTTTCCATAGCAGAAATACGAATATAACCTGACTGAATGAGACCTGTTAACGGACAAGAACCTGAT
**CoV-bait-3877**	AGTACGAGCTCTAGCCTTTCCAGATATAGCATATTTCAAATTCATTTGAGTCATTGTGGGTAGAACATTTCTCTTTGTTAAAGCAAAAAGTGCATCCTGCTCCTCATATGATAGAGTCTC
**CoV-bait-3878**	GCCCTTCACTTCGCTCGCTTCCGATGAGAGAACCTTGAGGATGAAGGGATTGTCCCGTGCGGAGCCGTCTCTGACTTCAGTGATTCTAAGACGAAGTTCCCCACTTATTAGGTCTTTATC
**CoV-bait-3879**	TTTGAAACAACAGTACGTATGCAGCGCACTATTGTAACAAGTAGCAACCTTACCACAATCACAACTCTTACTTGGTCCAATCTTGACAAAATAACGCATAGTTGTCAACTCCAAACCACC

**CoV-bait-3880**	GGTACGCACGCCATCGTAATTCCCCGGTGTATGAATAAAAAATTTCAGCTGCGAGCCGCTCCTCTCAATACTTCCTATCGAATTAAACGATCCCGAACCGTGAGCCTGAGGTGCTTGATG
**CoV-bait-3881**	AAAACAATCATCAACAGTGCTTTTAGCACTATCATGAACCAAAAATGCGTAATTTGAAGGAAAGGTTTTTAATGCCATTTGCAAGACCTGTTTAATAGGTGTTGTACAAATGACCCTACC
**CoV-bait-3882**	TGCTCTTGGAGACAGACTTTGCGACATCCAACACAGTGCCACTAGGCAACACATGCTGAACACCACACAACCATTCAATAGAGCTTATAGCAGAAACAGACTGAGCCTTATATGGAACAT
**CoV-bait-3883**	GCCTCCTCACAAGGAACGCCTGGGCTAATCAACTTCGACGTCTTGCGGTTCCATACTATGAGACAGTTTTGGAGCGTAATCCTGTAGTCTTCTAGTACTAGTCCAACAGGTAGACTAAAG
**CoV-bait-3884**	CAATTCTTAAAGAGCCCACTAAGAACCGACTGTAGATCGGTCGTAGTTATCTCCTGAAACTCGAGAGCATTAAACAAATTAGCATCATTCATAACACAAAATATTCCTTTCTTGGCTCTT
**CoV-bait-3885**	CTCCTGCTGCAGTAATCCCACCAAACATCGTAGAAGCTACCAACGAACCTGTATACATAGCCTGCATCTCTCCTGTAATAACGGGGGGCAAGACCATAATGCCATTATAATACTGTGCAC
**CoV-bait-3886**	AAAGCAGTAGAAGGTATACACATAGCAAACAAAATATTAAAGTTTGCACAATGCAAAATACACCTATCGTCTTGGCAATCAATAGTATTAGGATGATAGGTTCTGTTCCAAAAAGGAAAA
**CoV-bait-3887**	AGAGCGACCATTAGAACAGCGCTTATAGTCTTCATCAACAGTACCAAGGCCATTAGTAACCACTTTATTAAAAAGCAGGTCTTCAATAAAAGACCTTTTTTGTACCACCCTGCCACTTGC
**CoV-bait-3888**	AAGCTTTAAATCTATAACAGAACACACCGCCTTAAAAGCCGCTGTGTTAGTCTCAGTAATAAAATAGTTATGAATAGTTGAGCTACCTTTTAGCATTTCTTCCATAATAATATGACCTTC
**CoV-bait-3889**	AAATAAATGTATTACCTTCACCAAATACATCACAATTCTTGCAGCACCACATATGCTTACTGCACATCTTAGCTACACTATTAGCATACACATAGTAGGGAAACCTTTTACTTCCAACTA
**CoV-bait-3890**	CAATGGCAGAAAAACTGGATTCTAAAATCGGTTTTCCTGCTTTAGAACTCATAGAATCATAACTATACATTGCATTAGTACCACAATAATGTGAACCATGCTTAAAAACGTTTGCACAAA
**CoV-bait-3891**	AAACCCGCCATTGGAATGTGGGTGAATAACCAGAAGGCACTAGACACAGCATAATTGTATGACCGCCAGTCTACAAATATGGAAGTCTGTGCAAAGAAATAATGCAATGTACCTGCCAAC
**CoV-bait-3892**	ACCAATTGTCGAAACTAAAATCACCAGGAATTTTGCCACCATCTAGAACTGGAAAAACATGTGCTGGAAAGCCATTGCATTCTTTGCAAACACTATAATTTTTAATCAAACCTTGACCAT
**CoV-bait-3893**	GTTCCTTGATTGCACATGCTGGTAGATTATTGTCTACTATAAATGGTTGGTTAACAATAGTTTCCAGCAATGGGTCTTCTTCACCATTTTCCGTACTATCAGTAATAATAGTTGAAACAT
**CoV-bait-3894**	ACTTCTTCACACTAGTAGCCAATGTGCTGTCACTAGTGTGGTCAAGTATTTCTAGTCTACACTTATCAAAAGTACTAGTGGCTACTATGTCTTTTGCATAGGTAGCCATAACACGCCAGA
**CoV-bait-3895**	ATCATTAGAACCATAGGTTCTCTGATCTACACGTAAATGCTTGTACAAATGTAAAGAATCTTGACCATCAAAACATGCGAGGCAAGTTAAATCTCCTGCACAAAATGATTGATAGTCATA
**CoV-bait-3896**	ACGTTCCCAATTGAGAGGGGATGGCACATACTTATCATTGAGCCAATTCTCAATCCCACAATCGGGAAGATTAGGAACACGGCGATACACTGTAGCTACAGGTTGAACAGTGAAGCGTGA
**CoV-bait-3897**	CTCTTCACTACAATGACTAATACAAGTGACATACTAATGTAGACACCAATATCAGTATGTGTAGTGCGCATATAAGCATTAGTGGGGGCAAGCCAATTTGCAACTGCAATCAGCGCTGAC
**CoV-bait-3898**	ACTAATCAGTGTTAACATCAATCATTGGACCAGGCTGAACACTTGGACGGGTGCGAGTGCGCTGAGTGATGCTACCTTGCACACGCTGCTCCTTTGGAGGTTCAGACATTTGGTCTGTTG
**CoV-bait-3899**	AATTTACTACCCAACATGATTAATATAAATAGGTGTAGAGCTTATCTTAGGAGGCTGAGGTGCACGGTACTTCTTAGTACTCGGCTTAAGTTTAATTTCCTCGTAAAATGGCTCTTCCTT
**CoV-bait-3900**	TGTTGTAGCACCAAAATTATAATTTAACTGTCCCAACAACTTAGTCAATGCCTGTGATTGAGCATTCACTGCATTTTGTACCTCAGTTATTGCGCTAGACACTGCACTAAAGCCCTCATT
**CoV-bait-3901**	TATTTTGGTCAAAATGTCTCAAAGCGTAAACTAAATCCATCATAGTGTACTCAGTTAAACGCTGACGCACAATATGTGGAGTTTTAGTTTTATCCACGTCAAAGACGAAAAAATCATGAG
**CoV-bait-3902**	CTAATTTCTTTATCAACAATCTTGTTGCCATTAGCACCCATAAGTGCGCTCACATTAGCAGTTGTCGCCTGCAAAATGTTAAACACACTATTGGCATATGCGGTTGTGGCATCACCACTA

**CoV-bait-3903**	TCAGGATACGGTAAGTATCTCATCTCTCCATCTATTTCAACTAGCATTGAATGCTGTGAACAAAACTCATGTGGCCCAATCTTAACATCTTCTTCGGTCCAACATTTGGAATCGGCCATA
**CoV-bait-3904**	TGGTCTATTAATGGCAGAGCTAGCATCATGCGTCACATTGCCCTTATAGAGTATTTTAAAGCACTGGCCTGAAAGCTCCTTCTTGGCTAACAATTTATTATTGTAGACAAGAGCGCTCAC
**CoV-bait-3905**	AAGCAAAGAACCACCCCTGCAAACAGCTGGTGGTTGAGAGGGCATGGTGTTGAACCCTCTAGCTATAATTACGAGACTCACACTACGTGCACCCCAACCTATGTTGCCATAAGTACTATT
**CoV-bait-3906**	ACTCCTCGTCAAAGTTATTGTAGAGAGGTTCCTGTGGAGATAAAATAGTGTAATTAACAGTAGTATAATTAACACTACACGACTCTAAAACCTGAGTATTAGAATTGCTAATATTAACAG
**CoV-bait-3907**	TAAGGTCAACTGGTGCATCAGGTGTGCCTTCCAGTACCTCCACTTCCATTTTCTGCTGTCTAGGTGTCAACAAGACTCCTAGTACTTTAGCACAAACCTGTGTCTCTGACAACACACCAC
**CoV-bait-3908**	CAACTTGAAAAGGCGCCATGACTTTACCAAGGATTACATCGAGTCCTTCGCAAACGTCATAACTAAAATTTACAGTCACTGTTTTAGGGATTGTGCGAACCTCCTCATCACCAAAGGTAA
**CoV-bait-3909**	AGCCGTTGATCAATGAAAAGCACGTTTTTAGTACAAACGAAATAATGGTCCCGTAACATAAATATTTAGTGCCCCATCGGCCGATAACCAGTCTAGGTGAAACCACAGATAGGATAGGTA
**CoV-bait-3910**	GCGACATGTTGCACCACAATAGATGTATCTGATTCTTGTTGTTCAACAGCCTCTGCCCAATCAACCACTTTTGATGTGGGGTCTGGTTCAGGTACTGGATGTAAATCAACAGCCAGAGCA
**CoV-bait-3911**	CTGTATTATAAGCCACTATACCTGACACTTTAAATATAGGTGGTTTTGTAACTACTAACCAGCAACGCACAAGTAACTGGTAAAACAAACACAATAAACCCACACACTTCGCGCCAGGAT
**CoV-bait-3912**	AGTAAATCCTGAACAGGCTTTACTAGCACTCCATTATTGTCAAACCCATACTGGTCTGACAGATAGACATCACCGCCACGCTTAGCCTTAGGCTTTGCAGGACGGCTATTGTCAGGAAAA
**CoV-bait-3913**	CGTAGTGGACACATCCCTCACGCTGGATACGATTATAAGAATCGATATCAGAAGTAACAATGTTAAGCTTAGTAGCACTGACTGCCGGTATGACAGACAGAGGTACAACCCCATCCTTTG
**CoV-bait-3914**	CACATATGTGCGATTACCAGGTATAAGGCGCATGCCTGGACTCTTGTCCAACAACATGACACAACCTGGTTTAATCACACCTGCAGACTCTGCATCTACGCACGATGACGTGAGTTGAAA
**CoV-bait-3915**	GACATGACTGCCAAACAGCAATGCATGAGATGAGGGGACTAGGTTCAGCATAGCTGTAGTACGCCCATCTCTAATTCCCTCTTCAACCATCTTATCATCACCAAAATTGCGGTCTTTACC
**CoV-bait-3916**	CTATTGCTGACAACACCACAAGTAGATGTCAAAACAACAGAACCTTGGACCTTAGTGACACGTGTTGTAACGCTCTTACCATGGGTACACACCATGTTATCCCCTTTGGCTTGTAGAGGC
**CoV-bait-3917**	AGTCTAGGTAAAGTGCATGACAAACAATGTCAGTCTCCTTAACAATGGTCTTACAACCTACAACACCTGAATAAACCATATTATCATATGATTTAACAGAAGACTTAACTATGGGAATCC
**CoV-bait-3918**	TTTGGTGAAACTGACGAGTCGTCATAGTGGACAAAAGTGATACACCCCCAACAGTGCGTGCACGTTCCTTACCACTAATGGCATACTTAAGATTAAGTTGTGTCATTGTCGGGAGGATAT
**CoV-bait-3919**	GCTCCGTGGAGTGGGATGAGGATTGAGTTTTCCAGGGTTGTAGGTTGAATGTATGGTTCTGCGTTGACATTTCCGACCATTGTTGGGTTGAGACCTGCATATGGTGTTTTACCGACTTGA
**CoV-bait-3920**	CTTTATACTTATCAAAGAATCCCAAATCTTTAGAACCACTCAGAATAATTTCCCAGAGGCCTGTATGATTTTTAAGACATTTAGGCATCTTATACTTATGCTTGAAAGGCTCGACAATGA
**CoV-bait-3921**	AGGACAAATAACCTCATCACCAAGCCAAAGACCATTAAGAACCATGTTACCATAGCTAACGCGCACAATGCAATGTTCAATAACACCTGAAGGCTGCGCAAATTTACGCAGACCAGCTTG
**CoV-bait-3922**	ACAAAAATAAGAATGCATAGTTTTTCCAAATGGGACGTGAGAGACACTGTTATTTGCAGGCTGAAAATCAGTGACATTAGCACTGTTATAACAGGCTATGGATGCAGTTGTGTTATCCTC
**CoV-bait-3923**	TGGATGGATCGTACATGTCGGAAATTAGTAAATCTACTTGTTGTCCAACACGCACAGTAACGCAGTCGCCAAAAAGAGTAATATCCGCGTCGGACACATACTCGTTTAGGTCATTATCAA
**CoV-bait-3924**	CATATTCTGCAAAGTCTGATGTGTGGAGTATTTTATATGGTGTTACAATTTCAAAACCTTCTGAATGATTATCTTGGTTGAAAATTGGTGAAACATATTCAGCGAGCGAATCCATATCTT
**CoV-bait-3925**	AACATTTGCAGATAAAACAACACCTACATTAGTGCCTTTGAGATTACCCTTAGCACAACCAGTCTCAATTAAAACAGACTGGTCTTCAGCTGAGAGTGTTGAAAAGTCAAAAGCATAATG
**CoV-bait-3926**	AAAGTGCTACAAATGCTACAAATATGTAAAGTGCTGTTAGAAAACTACCGTTCTCTTCTAGCGATTTACTCAGTAAATTCGTCATCTGAACAATCGTCAGCTGACTCTATTTCATAAAAA

**CoV-bait-3927**	CAAGCGCATTTATTGGGTTGGAAGTGAAATTGAGGTTGAATCTCAATGCACCCGCTGTTTCATTGATAGAATGACCGTTACACTTGTCAGCATTAACACCACTTAAAGAAATAACTTCTT
**CoV-bait-3928**	GTGCGCTAACATCCCGCATCCGCGTATGCTTCCGAAGTGTCATCGCATGGGAACCCGTGAGCACTGCTAGACTGAGGTGACAGCCGCAGTCTTTAGGATGTGTGGGACAACCGTTGACAG
**CoV-bait-3929**	ATGGTGTTAAATTCGACATCAAGATCATACTCGACCGTGACCTTCTTCTTTGCAGCAATGATTTTAACGTCATCTGCTGCAGCAAAGGACACACTCTTACCTGCACAAGGAAAGCGCCAA
**CoV-bait-3930**	ACAATGTTGTCTACAGCCCAGGGATCCATGTAGGGGTTAGCCTCCTAAATTTCGGCAGGTGCCGAAAATTATAAGGTTTACCCGTTGTAAGAATAGGCTTGGTACTGGTACTATAATCAG
**CoV-bait-3931**	AGAGCCCAGGAACCATTAAAGGTAAAACAAATACCCTCCTCAGCAGACTCACACAACCCTACCCTGCAATAAGTCATTGCTTTGGTGCGAATGACCCTAACAAGACCTTCTCGCAGTACC
**CoV-bait-3932**	ACACCACCAATACCAATCAACTTAAAATTAAGAATCAAGGCTTCAAAACTATTACGTGGTGGACGCAACCCATTGGCATTCATATAACGAAGTTCCTGTACAGAAATTTTATAATTATAA
**CoV-bait-3933**	AACCAAGAGCACATTAGCACATAGAACAAGCGCAACGCATTCATCGGAGCCAAAACAACACGTTTTATGCTCTTCTGCACTACATCACACTTGTCATCACGCCTTGAAAAGAGTTCCACC
**CoV-bait-3934**	CAACAACTGCGATAGCTGTAATAAGCAAGAAGCTGTTTCTGGTCAAACCCAAATGATTGTTTAACAGATTCTAATTCAGCTTCAGACAAGTTCTCAAACTGATAGAGTACTTTGCCTGCA
**CoV-bait-3935**	GGCAACATGAGCTCCCTTATGAACGCTACAATACATATCATGATTGCTAGATAAAGAACCTGTATAGCCCCACTGCTGTATATCTACAATAAGTGGGTTATACACATAATCACAAGACAA
**CoV-bait-3936**	TCAGGGTTACCCAACTTCTCCTCTGTGTCCTCAAACATCCACGGAAATTCTGGGGCCCATTTTAGCGTGAGGCCGTATTTGTTGACTCGCTTAGCCATGATACCTCCTATGGGTGGGAGG
**CoV-bait-3937**	CATATTATGGTACGTTTCTATTTTTAATGAAGGTAGAAACCCTAGCGCAGAAACTAAGGTTTTATATGAAATAAGTGTGCCATCAGACACACCTACGAGTTTTGCAAGTGGTTCATCACA
**CoV-bait-3938**	TTAGCAGTACCGTCCACCCATATTGCCTTAAGACTTACAGAGTCCAAAGTAGGGTAGTAGACAGGTACTTTTGAATTTTCGTCAGTATCAGGTGTCAACTTATAAGTAAAGCCATCCTTT
**CoV-bait-3939**	ATGTGTTGCGTTGGCATTCAGAGACGGATCGATAGACGCATTCACTATCAAGCGAGTGAGCCAGGTCTTGCAGTGACGTTGACAACTGCACCCCGGGTGGAGAACATCAGGGGCACACAA
**CoV-bait-3940**	TCAACACCATTCATAAGAGTACTAAGCATGTTGTTCCAACCACCATAAAACTTAGTGGTGCCAATAACAACAGCGGCGCCTCTTGTGTTAACAATAGACTTCAAATGCTTTTGGTGATAC
**CoV-bait-3941**	AAGTTAGATCCCTCTCAAAAGGTTTAAGTTTTGTTTTTCGGGAGGATCTATAATAATACTGGCCCTGATCTTGTTTAGCAGTGTTCCAAGCTATGACACAGCCTGTAAAGTCATCAGGCA
**CoV-bait-3942**	TACCGCCCGTCCACCAACAATAGCTTGTTCAGCCATTACATGACAAATACCAGTAGGATCCTCAACTGGTTTCTCAAATGTAACTTGGGTAGGTTCCTGTCGACACAATTGTTCTTGTGC
**CoV-bait-3943**	AATAAATAACAAAACATCCTCTTCTAAATTCGACCCAAAATACCACATAGAAATTAAAGTAACTATTCTACCAACCAAAAACATCAAAGAAAAAATGTCATGATTGATACTATGCATAGT
**CoV-bait-3944**	ACACGCCCGAAGAGGGCAAATCGGTTTGAATCGGTTAAAGCCGCAGCGAGGCGCAGGAGTACTAGTATCGCGACGACCTGCTCGCCGATATCCTGCATCCGAGACGACAACATATTGAGG
**CoV-bait-3945**	GCCGCGGTGACGGGTAACGACGGATGCTAGTCGCTCACCAAAAAGACATTAAACAGATGAAACTGCGGGACGTTATTTCCCCTATGCTTAATATAAACCCCAAACTATTCACATCGGCCG
**CoV-bait-3946**	TGATAGCACCACCGACCATAGCACCAGTCAAAGAGGCAGTGTACATAGCCATCTTTTCAGCATCAACGACACCAGGTAAAACCATAATGCCATTGTAATACTGAGCACAAACGGTGTCAG
**CoV-bait-3947**	TGTAATGTGGAGTTATAACTAACTGTTGGTGGTGTGTACAACTTATCCATGTGGTCATTAGCAAAATCACTCATAGCTTTAGCCAGATGTGCAAAGCATGCAAGCCGGTAATCTGCCTCA
**CoV-bait-3948**	CATAGCTGGATCAGCAGCATAAACTAGTAATTCTCTTAAAGAGAGGCGTTGTACGTGATGTTTTACATCTTGATTCATTACAACACCTAACTCACGATAGTGGTAACCTGTCGATACCAC
**CoV-bait-3949**	CGCATTTAAAGCTAGAGCCTGCTCAATTGTTGAACACGCACTAGTATATTGTAGTAGTAGTTGCAGGCAACGAGGGTTGCCATTGCAAACATATTTTCTGCAATCCACCGTAATAGGATG
**CoV-bait-3950**	CACCATCTATAACTTGTCCAGTAGACCTATCATACACCATATAATGCCCACCATCCACTGGACCGGTAAAAGCAACATATGCTGTACCACACAATATTTCAGATGTTTTATATGTCCGCA

**CoV-bait-3951**	AGTCAGAAAAATTACAGTACACCGTGCAGAGACTAGTTGCGCTCCACGACATACCTTGAGAAGGAGCTGTAATTGCTACGGCACTAACATTAAGCGTATGGCCATAGTTAATACTACCTA
**CoV-bait-3952**	GGCGAGATTACGCAATATAAAATCGCCAAAATCAAAGAACTTCTTAGCACCGTTGTCCAAGTCTGTCATAACACGATCAAGCTTGATCTTTTTAGGCATAGAACAAACGTAGCCCCCTTT
**CoV-bait-3953**	CGTCTTTATTCCACACTAAACCTAATTGTTTAAAATGGTAACCGGCCGTAACAACTACTGGAACTCCATCAATAAAAACTTTCCTGACAAGTGGTCCAAATGCTGTTACAGGTATTGTTG
**CoV-bait-3954**	TTGAGAAAAGAATTAAATAGATCTGCAACAGCTCCTGATGGTTTATAATTAACCATCTGCATGAATGCTATAGCGGATGCTACATAACAATTATTATGGCTTTGTTTCAAAACCATAATA
**CoV-bait-3955**	AGTAGCACAATCACCTGTAATGCTAAAATCTGCATCACTAACGTAATCATTGATATCATTGTCAATGATTATTGCATCAGGTGGTAGCCAACGTTTTAAAACAGTTGTACCAGGTGCCAC
**CoV-bait-3956**	GCAAGACTACGCCTCCGAAGATTTTAATGATCAGCAGAGTTCCTGAAGCGTAAATGGAAACAGCAACAAGCAGAGTCGAGATAAACCAACAAAGGTTTTTAATTGTTGGCAGCAGTGGAA
**CoV-bait-3957**	TTTAGTTCCACAATGTGTTTGTAAGATTTCACGTAGTAGCTCTACGCTATCAGACAAATGACGGAGAGCATATACTAAATCCAACAACGTATATTTAGTCAAATACTGACGTACAACATT
**CoV-bait-3958**	AATCACAATTTAAACAAAAGAAATTGTGCTTCTTACAGAACTTAGAACCACCATTGGCATGTACGTAGAAGGATTTGCTAGTACCCTGAAAAATAGTCTGGACAGGAACGCGCTTAAGGC
**CoV-bait-3959**	TTTTTTTTTTTTTTTTTGTGAATCCCTATCAAAACCACACCCATCTACAACTACTAACTATAGCATGTAGCACATATGTTCTAGCTCTTCCATATAGGCTCGTCTAAGCGGATCTTTGAT
**CoV-bait-3960**	AAGACCTGCTTGTTTAACACCCTTAATCTTACCACTGACAAGACAAGCGAGTGCGTTTTCAAAAAGGATGTAATCACCAGCACGCTTGAATTTTTTGTTGGCGATCTTAAAGTGATCAAC
**CoV-bait-3961**	TAGACACATCACCAAAGACAATGTGTTCAAATGCATAGTCTTCAAGACCATACTTAGAGATGAATAAGCCCATGTCTAGATTTAAGAAATCAGACTCCATCTCACTACGTGGTTCAAAGA
**CoV-bait-3962**	AGGTTTCATGAAGGAAAGTGCATGATTAACAGCTTCAATGTCAGAAGAGACATCAATAACCTCTTCAGGTTTGATAATGTCCTCTTCGATAGCTGAAACAACAGTCACTTCTTCTACTTG
**CoV-bait-3963**	AAATGCCTCAAAGCCAACATCTGCGACATAATCCTTACAGAGGTCAACAAATATAGCCTTGTCTTCAACAAAGCTAGGTGCATTGAAGACATACCTATGTGTGACAACACATTTTCCATC
**CoV-bait-3964**	CGTCACCATCCATTTTAACTAGCATTGTATGTTGTGAACAAAATTCATGAGGTCCATGGTTTATGTCATTTTCAACCCAACATTTGGCTTCAGACATAAAAACGTTATTTTGGTAATATA
**CoV-bait-3965**	AAGCTGTTCATCAAACGTAGGTGATGCCACAAAACCATTAAAGGCTTGAACACCAACAGCACGCCAGCAGCTAACACCTTCCCAGTCACCAACATATTGCAAAGTCAGACCATTAAAATA
**CoV-bait-3966**	AACCTGAGCAAGAAAGATTTCAAGGTTCTTGTCGTCCTTAGGGACTAACATTTTATATGTATAGGTGATTTCAACACCATCAGCCATTTCCTTGGTGGCAACATTACCACCAAAAAGCAG
**CoV-bait-3967**	CCTTAAAGTCAATAACATCATAAGGCACTGCAGTCATTGTGTACCATGGAGTAAACCATTGAGGGTTTAGCACCACTAACAACATAAGCACGTGCACAAGAATGCTGCGCCACGGTATAA
**CoV-bait-3968**	TCACCAGTAACAACATCTTCATCGTCATTGTCACCAGTAACAACATCTTCATCGTCATTGTCACCAGTAACAACATGTTCATCGTCATTGTCACCAGTAACAACATGTTCATCGTCATTG
**CoV-bait-3969**	CACAATAACAAAAAGTGATAAAAGGCTTAACAAAGGGATACAAATCTAGTAATCTAATGTCAATGGCAAAAATTGCTAGATGCAGGTTGACTAAAAAGTCCAATAGCATTTGTTCTCCTT
**CoV-bait-3970**	AGTCTGATTAGTCATACCGGTGCCAAGTGCAGCTACAGAAAAGCAAACTGTACGTTTGTCAACTAAAGCCGGAGACGATGCTATAAGCAGTGACGGGTCAGCACAGAATTGTAATAATTC
**CoV-bait-3971**	GCCAAGTAATGGATTCAATGGCACCAGTAGACTGAAACTGGTAGGGCAGGTTGGTTCTAACGACGTCCCATGCTTTAACATGACGAACACCGTTAACAACCAACTCTGTCTCATCACCAA
**CoV-bait-3972**	CCCTTAGGCACTTTATAACTGTACTTAACAGTTAAGACAACATCATCACCCTCTTCTTTAGAAGTCCACTCACCTCCAAATAAAATGGCGGCGTTACTAGGAACCATCTCAGCTAGTTGA

**CoV-bait-3973**	GTTGAAGGACATGAGTCCAACAACGTTAGAGTTGAATGGAAGAAAAAGATCTTGTACCAAGTGGTGAGCATCTGAACGGTATATATCATCAGGGTAATAAACACCACGTTTAGACGATGA
**CoV-bait-3974**	ACTGTAATAGTGGTAAAATTACAGTGTGCGGTACAAAATTGGTTAGCTGACCACGACATACCTTCATCTGGTGCAGTCATGGCTACAGAAGAAGCGCTAAAATTTTTACTCGCATAAATA
**CoV-bait-3975**	AAGTTGAATTTGCATAGCCCTCAACGTAGTCTTTGCACAAAGTCTCATTAGGTGGACCAAAACGTACAAACATAAACAACAGTGCGTAACCTGTATACAACAATAGGCAAAACTTGAGAA
**CoV-bait-3976**	AATACAATGTGGTAATGTAATGAGTGTCTGAACTGCGTTTAACATACACCTCCACCTGACAAGCTTCAGCTAGAGTGTGATTAGAAACACAGAACTTGCCAAAAGGCAAGTAATTATTAA
**CoV-bait-3977**	ATTACCGTAACAGACACGAACAACACACTTTTCAACTGCGCCAGACGGCTGCGCCATCTTACGAAAACCAGATTGAAGAGTGGAATTGTAAGAGACTGTCGGCGGTGTGTACAACTGATC
**CoV-bait-3978**	AGGTATACACATAGTAGTACTATTCAGATACTGACATAGCTGAGTATATTTGACAACATTGAACATTATGCCATCTGGTAGTTTAATACTAGCACCATAGTTGTACAAATTACATGGCTC
**CoV-bait-3979**	TAATTTAAAGCAATTGCCTTTCTTTTACTATTCTGATAGTCCTTGTGAGTCTCATGGCAAACAAGTAGTGTCGGATATTGATTATGTTCCACTCAAATCTGCTACGTGTATTACACGATG
**CoV-bait-3980**	TAGGAAAGGCAAACCCAGTATGGTCTAGCATGTGGAACTCACCAGCCTTATCAAAACCATAATGTGAATCCCAATTAGCACTTGGTACAACCTTAGCTACAACATCAGCAACAACAGGTT
**CoV-bait-3981**	TTACCGCTGTAGTCTTACTCTTAAAAGCGACAACCCAATCTGATGAAATGGTATACTTGCAGCTCTGAGTTGCCTTATCACAACCATATGCATGAACTCTATATGGTGTCCAAGAACCAC
**CoV-bait-3982**	GCATTATTAATACCAGTGTCTGTGGAAATCTGGGTAGATTCAAGCTGGTAATAAACTTCAGTGAAAGGACCAATAAGTGTAAGATAGTCCTTAACACCAGATTCCCATTTTTCGAAGTAA
**CoV-bait-3983**	GTTATCTACAGTTGTGAACACCTTAATAGTTTTAACCTCTCTTAAAGCTAAGAGAGTTTTTAACTTCTCAAGAGGAAGAACTTCACCTTCCATGTGAAATTCTATAACATTTCCTACTGT
**CoV-bait-3984**	TCAGCTCCGCACGCTCCGGGGGGTCAGGGTGCTGGGGGTGTCGGGCAGGGCGCACAGGCTCTGGCCCAGGGGCAGCTTGCAGTCCTCGACAAACAGACTAGAGTTCACCAGTCCCAGGAC
**CoV-bait-3985**	GGCTGTAGACAAAATTATACCAGCCACAATTAACACCTGCTCCACAATAAGGTAGATAACCACCTAGAATGGTTTCTGTGTTTGGTGGTATATCCCAAGTTTGTAGGAAATTGTAAAAAT
**CoV-bait-3986**	TTTACAGACAGTGTTTTCTAGACAGTATCTTATAGGATCCAATGTTCCTATTGGCACTTGAACATATTTGCCTTTAAGTCTACAAAAACCATCCATATGTGGATGCTCAACATGCGCTCT
**CoV-bait-3987**	CATCAATCAACTGAAGAGACTTAAGAGCTTCTTTACAACTGTACTTCTTGTCTGTGATGTCGTAATTATAACGTGTATACTCATCACCAGTGTAAAGTCTATAAAACCCTTCCGAACACT
**CoV-bait-3988**	TGCAGCCTGCGATGAAAAGCAATGCAGAGGTGTAAGCAATGATTTACTAGTGCCAATATTCCAGACACCAGGTTTAAACTTGCCCTTGGTCACTTTATAATTACCACAAGACTCAATGAT
**CoV-bait-3989**	TGATGTTATCAATAGTAGCTTGGAGATCCTTCGAGAGATTCATCAAATACTCGGACTTAGCCTCGAGTTTGTGAATCTCGGAAGACAGATTGAGATAAGTCATATTGAGCAAAGTGACAT
**CoV-bait-3990**	CTAGGAATGTTGTGTAGGAAACACCCTTAAGAAAGAGGTGCGATGGTGCCACTACAGGTTTTCCAACATTGACAACAATTGTAGTACCTTTAACTACAACAACTTTGTCAATGTAGTTAA
**CoV-bait-3991**	ACCGCAATAGCCAAACATGCGATGACAATGTTAGCCAACATTGCACCAGATGTAGCTATCACAGACATGTTAGAGTTAAAGAGCGCGAACACATTTTTGAGGAATCCAATTGCAGAACTT
**CoV-bait-3992**	TGGATGCCTTAGGTAACTTTGGCATAGATAGCTTCACATTAAAAGTAGTCTTTGGTGTTTTAACATTTGAAATAAGTGGTGTTCGTGATGAAAATGAACGCCAGGATGAAGTGGATGACA
**CoV-bait-3993**	ACTGCTTCATAAAACCTGGGTCAGCAAGTGTGACTTTGTTGTAAAGCAAGTCCTCAATAAAAGATCTACTAGACGGTTTAGCGGGGTCTGGCAAAATCTGTGAAAAATTAAAACCACCAA
**CoV-bait-3994**	TCTCCACCCAAAAGTTGTGCCCATGCTTCTGCAAGAAAACCTTTAAACCTAATCTTAGCAGCTTGAAGGAGCACTACTGCTGCATTAATCCAGCAATTTCCATCACGCCACTCAAGTGTA

**CoV-bait-3995**	GGAAATTAATACAACTCTCTGCATTCTGGTGGTCTAACAAGTAGTCTACCAGAAGACACAAGAAACTTGAGAATTGGTTTCTTTTCAAGTTCTTTGAGAGATATTATAGGAGTTGCCTTT
**CoV-bait-3996**	AATCATACCTGGTATCAATTTATAAGTACTTGTGCTCTTATAATAAACAGAATCACTACCGTACTCGGATTGCTCAAACACAAATTCACCTAATTGAATTTTAGTATCCTTACTTATCTG
**CoV-bait-3997**	TTGTCAACACCATCAATTAGATTTCTGAGCATATTGTCCCATCCACCATAGAACTTGGTAGTCCCAATGACAACAGTAGCATTACGAGTGTTGACAATGGACTTTAAGTGCTTCTGGTGA
**CoV-bait-3998**	AAAACCAAGTTGTCTACAGTTCCATCACGTACAAGTACGCGGTCATTGACAACAGCAACAGGTAATTCACCAGCTTCACCTACAAATGAACCTTTCTTAATAACATTATAAGCAATGTTT
**CoV-bait-3999**	GGACCGTTGCGTGGAAACTCGCTAATAATAACGGCTTCTAGCTCCGGATTTGTTTTTCTTTTACCATATGTATTATTATAAATAAAACGTACACCCCTTGCACCAGGCAGAGCACACCAA
**CoV-bait-4000**	ACAGACACCAAGACCACCATACATTAAACTTGGATTCTTACAAAGCTCAGCATTAGAATTTTTATAATGATAATAAAAAGAGCCCAAACTGACATGAGTGTCAAAAGGCAATGTAACTTC
**CoV-bait-4001**	AACCAAAGCTATCATTTAAACTCTGTAACGAAATAGAGATGTTATCAAAACTATTTAAGTTATCAAGAAAATCGGTAACACTGACATCATCTGCCATGTTAACGCCATTAAGTAAACGTA
**CoV-bait-4002**	ACAAACTGGCTTTACAATAGCACGTGCAGTATTAAAAGGTTTTGGGTCCTGACAAAAGAAGCCAGTACCAGTCATAGTTCTAATCTTAAGCTTGAGTGTTTTACCACAAACACCACAAGC
**CoV-bait-4003**	TAGACTCTGTGAGTACACTCTAGATGTGTACCACTAACAGGGTGTTGAAAATCCTGGTCATGGTATATCTGAGTTCCAAATGGAAGGTCGACTGCAAATGATGTATGACAACCAACATTT
**CoV-bait-4004**	TAATTCTAATTTACTGTTAGTAAGAATGACAATATTGTCAGCACCAACAGCAAAACTGTCTTCAATATTATAATAAGACACACCAGCAACCTTTAAAACAGGTTCATTTACCTCAACAAT
**CoV-bait-4005**	GAAACCAATTGGTTAAAGGAAAATCACCTGGAATTCTGCCACCGTCAAGGGTGCTGAACACATAACCAATAAAGCTAGAACAATTAGTGCAAACGGTAAAATTACTGATAGCACCGTAGC
**CoV-bait-4006**	CTTTTCCACACGGGCAGACTATACTTTCTGGTTTGTACAAATCATCCAATTGTACAGTGCCATAGTGTAGACAAGCCGGTACTCCCTCAATGATCTCCTGAGACGAACCACAGTTTTCAC
**CoV-bait-4007**	AAGGTACTCAATGCTAAGAATAGTGCTAGCATTCTGAAACTGGTATGGGACATCACCACGAGTGACATTCCATGCCTTATGATAGGCTTGACCATTTACAGTGATGTTATCTCCATCATC
**CoV-bait-4008**	GAAGACCCCCATGTGTAACGGCTTGATATGCCATAGATACTGAATCCAGAAGCGTTCCTCCAGGGGGCAGAGGTGAAGCGTTCACTAACACGATCTTCCAACGTGCAAATTTCGCAGATA
**CoV-bait-4009**	TTTCTTCTGAGAGTCTTGTGACCAGAGGGTGGGCTGTTTCTGAAACTCTGGCGCATGAGTGGTGTTTAATGATTTCAAATGAGCCGCCGTCTTTAAAGGCATTGAGTGGGATGTCATGTT
**CoV-bait-4010**	TGGTTGTGTTGTGTGCTGGAAATGCTGTTGTTGATGCATTAACCAGTTCTCTAACAATTGTGGAACAATTGTTATCTTTCACAATAACCAGTTTTAAGTCTGGTTTTGATTGAGGTGTTG
**CoV-bait-4011**	TATATACGCCACGCCGGCGTTAATTGATCCGCATGTATCGCCGTCGATACATCCGTACAATTTACATCTTGATATAATACCGCTACTTCCGACGACGCATTCGTACCCGGCGTTATTACC
**CoV-bait-4012**	TTTATATAAACAAGAGTATCATTTATACCATCACGAACGAAAACTTTGTCACCAACAATAGCAACAGGCAACTCACCCTTGACACCAATAAAAGAACCTTTTCTGATAACATTATAAGCT
**CoV-bait-4013**	CGAGTGGTAGTGCTAAGATCTGATGTGTTGTAATGTAAAGAGTATGTCCTACAAAACGTTGTAATAATTCCCACGTTAAGACTGGATGTTCATGAAGGTCATTTTGCACGGCAGTAGCTT
**CoV-bait-4014**	CATTGACGTAATACCGCCGTACCCGGCGCTACACCTTTATCCGAACCCGCACCAAAATGTATTACACGCATATTATACGGTACCGCTAACGTTAACGTATTTAAATATTGACATAATTGC
**CoV-bait-4015**	CACTCCAACTAACAATATTTTTAGGACTCGCGTGTTTAGGTGCACTTAATGATAAAGACCCATATATACACACTTTCGCCAGCTGCTTCTTCCGTTCAACATTTAGAATTTCTCTCGCGT
**CoV-bait-4016**	GGTGCAATGGTAAGTCTACTTCGTGTTGTTTTAGTGCAATGTTAAGGTAAACCTTACTTGAGTGGTGAGTGATAGAGGGGGAGAAGGGTTCCGGTTAAGCGTCGATGCAAGCAAAGTGGC
**CoV-bait-4017**	TAGACAGCGCGATGACCAGCAATTCCTCGGAAAAACCAGTTTTAAACATCGGGTTGCCTTATAACTCAATTTTATGATGTTGGTTGATTTTGTCGAAGCCCTTCTTGCTTCTGAGTTGCT
**CoV-bait-4018**	ACAACAAACACAGAAAATCTGTATGCAAACGCGCCTATATACCAATTATTATTGATATTAAGCAGGACCATCATAGCCAAAGAAAGAAAGTCACCACAACACCAGTAATTGTAAACTGAG

**CoV-bait-4019**	AACATTGGGGGTAGCCATAAACCTATAGTAACCCTCACTACAGAAAAAGGCCTTACCACCAATAATGGCAGTATATCCAGTATTCAGAACGGGCACGTCATAGTCACAAAGCACAAGGCC
**CoV-bait-4020**	CTAACAAGTATGACATTGTTAAAAACGCTAGGCACACGTTTACTCTGAAGACTGACTCCATACATTTGTTTTACAACCTCGGTAACAGTAAACTCGTCAGTGAGTGATGCATAACCAATG
**CoV-bait-4021**	ACAGGAACTCTTGGTTCAAATAGAAGCCTAGGTGTGATTAAATACTTATTAGTATTAGGCTTTTGAAACAAAACCTCATCACGACCTCTAAGAACCAATGCTCTGCCATCGACGCAAACT
**CoV-bait-4022**	TTTGTGTCATGAATGTCTTTGACGCTATCAATATTGTCTACACAGAACTTGTTAAAAACACTCAACAAGCGCTTGTGCATATCAAGTGTACTAAAAGAACAAACTGATGAACCCTTAAGT
**CoV-bait-4023**	AACCCAATCCAAAAGAACATAGAATACTTTTCTATATTCAGGGTTAGGATGCTTGGACAATGGATATGCATCAATTGCCAAAGAAACATAACGTTCTAGAAGTATAACAGCATCAGTTTT
**CoV-bait-4024**	TTGACGTACTGGGCAAGAAAGTAACAGACAAGACCTTTAACGAAGATACCTCCAAAAATGAAAAGGAAAGCCAAGTACAAGAACGGTAACCAATTAACAAAAATTGGAAAACCAACATAT
**CoV-bait-4025**	CCATGTGCTTTGACATACTTTGCTAGCATGTTCAGGGCATTTTCAGCATCACCTGCATCACCTTTAGTAGCTCGAACTAGCCAATACAACCAGTGACAGAACTTTGCTACGTTGCCAACT
**CoV-bait-4026**	ATAATTTCGCTACCGATTTTACCGAATTTTTCGCTATCGTCGTCGGTAACGACGCACGTATACGCGAATTCGTCGATTTCGTAAACGTACATAATTGAAATAATAACGTAAATACATACG
**CoV-bait-4027**	AGTGGAAACTGGAACACGCTTCAGCCTTGCGCTCTTAGAGCAAGCCAGACACGAGGGTTTATCGCAACCAAACAACACGTGTTTGAGAAACATAAGCACGCGTACTACTAAAAATAGCAC
**CoV-bait-4028**	GCACAAAATAATGGTACATGAACACCACAATATCTTACGCAAGTTTTTAAAAAATGCAGAAACTGTACTACCACCCTTTTTACCTGAAATGGCAACAGATGGTATAGTTGTGTGCATCCG
**CoV-bait-4029**	TGACAATCCTTTCAGTGTATCACTGAGCATTTGTACTATCTTAATACGCACTACATTCCAGGGCAAGCCTTTATACATGAGTGGTATAAGATGTTTAAACTGGTCACCTGGTGGAGGTTT
**CoV-bait-4030**	CAACATAACCAGTCGGTACAGCTACTAAGTTAACACCTGTAGAAAATCCTAGCTGGAGAGGTAGGTTAGTACCCACAGCATCTCTAGTTGCATGACAGCCCTCTACATCAAAGCCAATCC
**CoV-bait-4031**	GTCACCTTCTGCTTTAACAGAACGCTGTTTAAGCTTACCAGGCATAACTTCGTTATTCTGTAACTTAACTATACGTTCTGCATTAATACACAATGGCCAACTCAAGTTCTCAGCATTAGA
**CoV-bait-4032**	CACCACACCGTAATACAGTTTGAGACGAACAAACAACACACAGACCTGCCGACTGCAAAACTGCAGAGCGTTCATACATGTTAGAATAAAACTTTTCATCCCAAAACTTACAAGTTGAGT
**CoV-bait-4033**	TGTTGAATGTGTGACAGTGACTGCACTATCACTTAGCATCAACTCACCAAGCTTGTGCAAGGTCAACTCAGCATCACCAGGTTGTCCTTTCTTTAAGCCTGAAATGTAATATAGCATATG
**CoV-bait-4034**	GGTTAGGATTTCAACAAAAGCACTATGAATAGATGTGCTAAAATCAACACCCAAAGTTGACACAAGTGTAGAATCTACAATCTTAATAGGTTTACACAACAATTGGCTAAAATAAACACA
**CoV-bait-4035**	TTTGAAGAATAGCATTACCAACAACAACACTAACAACCTCTAGAGCCCTAGTAGGTTTGTCATCAGTTGGATGTAAGCTATCTTCATGGTATATTTTGTCTGTTGTAAGGTCAACAGGGT
**CoV-bait-4036**	CACGTCTAGTCGTCACGGTGGGCAAAGCTGATAGGTTAAGAGTTGGCGGGTGGGTAGGATGGGGCTACGTAAGAGCAGGCGTAGGGGCACACAAGTGGTCCGGGTGAGGGTACCTTTGCT
**CoV-bait-4037**	CAGCCATAAAGGCCACAATCTTACAGCTGAGTGCACTGAAGGTCCACGAGGAGCCATGGCGCACAGAAGCCAGTACAAAGGGGAAGCAGATGGCAGAGCGTATGCCATCGGCTAGGCACA
**CoV-bait-4038**	CAGAAGAATAAACAATGTTAGCACAATCATGGCACGTTAACCAAGTGATTACCAAAGTTTAGTAATTTGCCATTATTTCTAATGAGCAGCTTACCCTTCCTTAGTAATCCAACGACCATT
**CoV-bait-4039**	CTCGAAATTCAGGTCCAGCTCGCCGGAGTACACTCCCTTGCACAGGCTCGGTCCGCCATAGGAGTAGGCATAACAGCAGGTGGCCCTTCCGCTAAACACGGACTGCTTACAGCCGCCCTG
**CoV-bait-4040**	AGTGACAGGTGTCTTGCAGGTATTGCAGATTAAACCTTGGTCGGGATTGAAATTGATGATATCAGTGAAGCAACCGTCGCAAACAAGATTTATTTTGTAAGCGCGAGCAGTCATTTCAGC

**CoV-bait-4041**	CGGAACGGAGCCTAAGTTATGCAACATGTTCTTCAGAAGAAGCGGGACTAACGTGGCCGTGGCTCCGAACAGATAGCTCCGAAGGTGCTGTATGTGGCCCTGAAACCGACGCCGAGGTTC
**CoV-bait-4042**	AGAGTAATTCCTCCAGAAAATATAATTGGCATGCATTATGTTGCCATCAATCTTTTCAACCTCTTTACCGACGTAGTTTACGCCGATTAGGTATGCTTCCGATGATGAGGCATTTACATT
**CoV-bait-4043**	AGAAAAGAATCCGACACATACTCGAAAGTGCAATTGAAGGCATTTTCAAATAATATCGTTTCTATCTGCTGCGAAGTGGGTCTAATGACAGCAAAAAATGGGTTGTCACATAATTGAAAA
**CoV-bait-4044**	CAAAAACTATGACATTAATAGGCAGTGAACCTTTAGTGTTGTTAGCTCTCAAATTGTCTAAATTGACAAAATGGGAGAGCGGATGTCTCTCATAGGTCTTTTGACCAGCCTTGTCAAAGT
**CoV-bait-4045**	AGCATACACTAAAAGTTCCTTGAAACTGAGACGCGAGCTATGTAAGTTTACATCCTGATTATGTACGACTCCTAACTCACGAAAATGGTATCCAGTTGAAACAACAAAAGGAACACCATC
**CoV-bait-4046**	CGTTGGTGCCGGGGGTGATCACGCTCACGCCGCCGAAGCTGCAGGGGCTGATGTCCAGGATCTCGCTGGTCTTGGGGTCGCGCACGCTGTCGGTGAAGTCGCTCACGTCGCGGCCGAACT
**CoV-bait-4047**	ATGTCAGGATTCTCTACGAAGTCATACCAATCCTTCTTATTGAAATAATCATCATCACAGCAATTGTATGTGACGAGTATTTCTTTTAATGTATCACAATTACCCTCATCAAAATGACGT
**CoV-bait-4048**	AACAGTTATTATCACTGCTCGCTAATGCACGCTTACCGTCAACGAAATCGCTGTCATAAGCGTAGGCATTATGATCAAGCACATGAAAAGTACCAGCATCCTTAAAGCCATAATGACTAT
**CoV-bait-4049**	AGTTCAGCAAGTGGGTTTTGAGCACAATCAATAGCATTGGTAATAGTCCCATTTTCATTAAAACTAAGCATGAAAGTGGTGTATTTTAAATTACCAACATAATAAGCAGCACTTTCTGGT
**CoV-bait-4050**	CTGTAGAACTAAGTGCAGAAAAATCTTGACTTAACCATACAACACTCTTACCACGTTGTGTTAAAACCTTTGCATTGACAATCTTAGCATCAAAAGTCATGCACTTACCAATGTCCATAG
**CoV-bait-4051**	GCAAGTGGGCTTTATTGCATAAGTGACATCGTCCTTTTCCCCAAGATAGAAAGGCAGGAGAGTGTCTTCTGCATGAATATGAAGATCTGGTACCCATCCGTGATACATTGAGGCTGTTCC
**CoV-bait-4052**	GACACCATTTTCTTCTGATGAAAGGTCTCTTTCAAAGGGTTTTAATTTGCTAGAGCGATGAGACCTATAAAAATAGCTACCGACGTCTTGCTTAGCAGTGTTCCAAGCTATAACACAGCC
**CoV-bait-4053**	CAGAAGAATACTGAGTTAAAAGAGTGAGGCAACGAGGGTTTCCATTACAAACATAAGTAGCACAATCAACACTCACAGGTCTGGAACTAAGTTGAAGATACTCAACTTGAACTGAAGTAG
**CoV-bait-4054**	TTTAGACTAAATGTAGAATTTTCTCACTATCTGTTACAACCTCTGTTGGATTCGCAGCAGCATAGTAGTCGCCATGCTTGGACTTCACGAAGAAAGCCCAGCCAGTACTTGAATTTGCAT
**CoV-bait-4055**	TGCTTGTTGGATGATTGGGCGTGAGAGTGTGAGTCCTAGTTTTGCTAGATCTTCGTAGAGTTCAGTGATTACTGCGATGGTGTTAGCGCAGAATGTGCAACCCTTGCCTTTGATTTTGAC
**CoV-bait-4056**	AATTGGCATTATTCTTGGACATATTAAAATACACGGGTAAAGTATTACCCAATAAGGATGGAATAGGCCATAAACAAAACAATTGCAAAGGCTGGCTAGAAGAAAACCTGCCAGACACAG
**CoV-bait-4057**	CACTTCTTGAACATCAAAACACTCTGTCACTGACGCTTTTGTATCTTGCGAAACAACGAAGGAACAATTACAACATTTAAGCATGTTTTTCAAGACACTTGTAGGTGGTACAGTGGAGAA
**CoV-bait-4058**	TTACCAGGCACCCACACGCATGCTCGGACTTCTGTCAACAAGCATAACGGCACCCTGCTTAATCAAACCGGCAGACTCAGCATCAATGCAAGAAGATGTAATCTGATGCACAGCTGCACAG
**CoV-bait-4059**	TGGCTTGGAAGTTGTGTTGCAGCTGTACGGTAAGTTGAGTCAAAGCTGCACCCTGCGAGTTAACAACCTCTTGAACCTTAGTAAGCGCATGAGCCACAGTGTTCAAACCCTTGGAAGTTT
**CoV-bait-4060**	ACGGTCTTACATTATTCAAACAAAGTGCTTTATCCAAGTAGCTACCAGACAATAAAATGTTCTGAAAAAAACATCTGATAGTCTGTTGGTATTTCCATCTCATTAGCAAGAAGCCTTGCAA
**CoV-bait-4061**	GCCATCAGTAGCCATCTGAAGGACTGTGGATCCCACAACTACATCAACCGCCTCTAGAGCCCTAGTAGGAACGGCATCAGTAATTGCAAGTGAGTCCTGTGTGATGACTTTATTTGTAGT
**CoV-bait-4062**	ACTTGGCGGTCTTCATTTAATGATAGGCTTAGCCAAGCGCTCACAAGATTCACCACTTAAATTAGAGGATTTTATCCCTATGGACAGCACAGTGAAAAATTACTTCATAACAGATGCGCA

**CoV-bait-4063**	TAGTTTGTCAAGTGAAAGAACCTCACCGTCAAGATGAAACTCGACGGGGCTCTCCAGAGTGTGGTACACAATTTTGTCACCACGCTTAAGAAATTCAACACCTAACTCTGTACGCTGTCC
**CoV-bait-4064**	TTATCCTGCATTGGTAGTGTGATGTTACTTAGTGTCGAGGCTATGGGTTGACCATAACCACTAAGCTTCATACCAAGATCAATGGTTATATTGACAGCGGTGTGTGTGAAAAAGCTAGGT
**CoV-bait-4065**	GCATGAGCAAGCATTTCTCTCAAATTCCAGGATACAGTTCCTAGAATCTCTTCCTTAGCATTAGGTGTTTCTGAAGGTAGCACGTAAAATGCAGATTTGCATTTCTTAAGAGCAGTCTTA
**CoV-bait-4066**	TAGCAAGTCTATAAAACCGCTCTGAGAAATTACAACAAGTCTTATGCTTACGAGCAAGTATTAGACTTGCAAAAATACGTAACATATTAGGCATAGCACGATCACACTTAGGATAGTCCC
**CoV-bait-4067**	CAAATACTATTACATTTATCGGTAACGAACCTTTCGTATTATTCGCACGTAAATTATCTAAATTTACAAAATGCGATAACGGATGACGTTCATACGTTTTTTGACCCGCTTTATCAAAAT
**CoV-bait-4068**	ATTGTTGCCGGGAAGCTAGAGTAAGTAGTTCGCCAGTTAATAGTTTGCGCAACGTTGTTGCCATTGCTACAGGCATCGTGGTGTCACGCTCGTCGTTTGGTATGGCTTCATTCAGCTCCG
**CoV-bait-4069**	TTCGCTAAACGATAAAAACGATGCGATAAATTACAACACGTATTATGTTTACGCGCTAATACTAACGACGCCATTATACGTAACATATTCGGCATCGCACGATCACATTTCGGATAATCC
**CoV-bait-4070**	TAGGTATATGAACTCCCAGGGGCTTTTGCCTCCTAAGAGTAGTATTGATGCTTTCAAGCTTAACATTAAGTTGTTGGGTATTGGAGGTAAACCATGTATCAAGGTTGCTACTGTACAGTC
**CoV-bait-4071**	AAACATTAAAGTTTGCGCAGTGCAAAACACACCTATCATCTGGACAATCAACTGTATTAGGGTGATAAGGACGGTCCCAATATGTAAAATACTTTGAGAACAAACTAAGTTTGAACTCAG
**CoV-bait-4072**	CCCCTGCAAAAGCACTTTTGCTTCCCTCGGGGGCACAATACAAGGAACGGGTGATCGACGTACAAAGCATAAAAAGGGCGTTTGCGAACGGAAGGCACGGTGCAGGTAAATATCAGTGAC
**CoV-bait-4073**	TATGGATTTAGTAGTAACATCTGCAACACCAACTACAACCGGACAAGCGGGATCATTGGTGTACTCTGCCTGTATGTAACCATCAAAAGACACAAATTTATTTGAAAAACAAGTGTCTGT
**CoV-bait-4074**	CTAATTGCACACCCTCTCTAAGAGAAGCATGAGCAATATTAACAAAATTATTAAAACTCTTTCTATCAACATCAAAATGAGACATAAAAGTATCAACATAAACATCAAACATAGTCTGGG
**CoV-bait-4075**	CTGCCGTAATAATAGGGGGCAGTACTAACAAACCATTATATTCTCTAGCGCAAACAAGGTCTTTAAGAGTACCCAAAGGTCCTGCTGTGCATTTTTTATATTCAGCATCAGTTGGCAAAC
**CoV-bait-4076**	ACGACCCTTCCACACATACGCCCGATAGGGAGTCGGAGGGCACAGTAGAGTGCGACTGCAATCCGACTGCGACTCATGGCTACTAAGAGTGCGAATTAGATTGAGAGCACCCGCCTACAT
**CoV-bait-4077**	GAATTAGGAGACCAAGACTTCATAAAAAGAAGATGTTGGCCACGAGGTTGCAAAAGCAGTGATCAGGATTGCCACTTCTGACGGCAGTAATTTCTCGCGTGGAAGGGCGTTTACGCACTG
**CoV-bait-4078**	AATAAAACATGCCATAATTGCAAGACCTGCAACAATAACATTAAACATCATTTGACCTGACAACGCTGCTGTACTAAAGTCTGTATTAAAGACTCTAAAGACATTGTACAGAAGCTGTAA
**CoV-bait-4079**	TGCTTAGCACCATTTTGTATAAGAACACCTAAATCAGCTGCGACAATATTATCACTCTTTATATATGTTGGAACCAAATTGTTGAAACTTTCGTCCGTGAATTCCAAACCGGCAGCCACT
**CoV-bait-4080**	TGTGGACTGGGAGAATCGTTATTCTAAACGTAATATCGTTATATGAGAAGAATGAACCTCCATCGTAAATGTACCATAGCAAACCCCGCTGAATGCCCACGAGGCTGCCAGACGTTCCCG
**CoV-bait-4081**	AAATAATTCTTATCAAAATCAGATGCACCAGCAACTAACTGAACAGAAGGTTTTGGTTGTCTAAGTGCATGACACTGACACCCATGACCTATCCAACACTGACAAACAGTGCAGACCTTA
**CoV-bait-4082**	GTAATCACCATACCTATCGTCATACAGAACTATTAGACCATTTGGCTCAATGTCTGTGTACGCACATACCTTAACAGTATTACGATATAATGGTGTTTGGTTCTCATAGTCCCAAATTAC
**CoV-bait-4083**	CAAAGGGATGAGATGCTTAAATTGTTCACCAGGAGGAGCTTTAGCCACAGCCTTTTTAAAGCTGTAACCATCTCTATCAGCAAACAAACCAGTGGCTTCCACAACAAAATCAATTCCTGT
**CoV-bait-4084**	ATTCATTACCAACTTAAACGAATATTGTGGCTTATGATCTTCACAATAATATGACATACCACCTAGATACAATTTGGTAACATCATTTACATCACATCCTGGTGCGTTACACACATATGG
**CoV-bait-4085**	CAGGAACAGCTACCATCTCGCCAAAAGCCACAAACCTGACAAATATCATGCGTCAACACATATGACACAGGATCTTTTATGCCTAAGGGCACTTGGACAAACTTGCCGCGTAATTTGCAC

**CoV-bait-4086**	TGTACGATACGATTGACAAGAGTCTGATCGGAACACGGTAGATCGTGTAGGTAGAGCATACGTTTGAAGACTGGGTCTGAGCCGTCATAGTAGCTATGCACTACACTTTTGCGGGATAAC
**CoV-bait-4087**	GAGCACTCAAATCTAAGGCAACAGGCCTCGAAATAAAGGTGCTATCCACAGTATATGTGTCGCAATTAACACAGTTCCAATTATGTTTAGTACAGTAGTATGTCCCACCATTTGCATTCA
**CoV-bait-4088**	GACATCCCACACGCGAACGTATGTTTTGCCGGGTTCCACATAGATGTCCCCCCATTCATAAAGTAATCCTTAAACTCCAGACCCTTCTTAACAGGAGTTCCATCTGGGCCACATAGGTA
**CoV-bait-4089**	CTGTGATTTGGTTTCCCACAATTTCTGTAGTTCCATATCACACCATTTAAGATCATCACCAGCGAGCCTAAACATTTGCCCAACAAAGACAGCTAGAGTTCTAATAGTACAATTCCATTC
**CoV-bait-4090**	TTGTAGAGGGAGAGAGAGGTCTTTACATGAGCGGATGATCACGCCATTGTTCTAAAATTCGCTGGGGTAGTACCAAGACGGAGGAGTCTTGCGTGACGGGGACCAAAGTAAGGTACAAAG
**CoV-bait-4091**	TAGCAGCTGCGCCATTTTGAGCAAAGAAGAAATGTCGCAAAGTCAAGTCGGAACCTTCATTAAAGAAACCTCTTTCAAGAAGAAAGTCATAGAATTCTTTATTAAAGTAACCAGGCTTGA
**CoV-bait-4092**	CACCATTACAGAAATCCTTCTTAACGAAAGTAGAATTAGCATAACCATCAACATAACTCTTACAAACGTAACCATTAAAGAAACTAAAACGTACTAGCATAAACCCAGCAGCATATATGG
**CoV-bait-4093**	GAACAGCCATGACCTGAAATTTCCGAGTCGCTTGCAACAGCAAGCGTCAACTGGTTGTAAGCCATGAGGGACAGTTGTTGGTTTCCACAGCGACGGAACACTGCTTCCACACTAGCCAGT
**CoV-bait-4094**	AACTTTGTCAATGTAGTTAAGACCATGTGGACAAAGACCATCCTCGACGCCAAGCTTCAACACGCTAGCATTCACAACTGGTGCAGTGACAACACGCTTACTACAACAACAACCGTCATG
**CoV-bait-4095**	TTTTGATCTAACGACTCCATCAACAAGTGATGAACTATTAGTCAAAAGAAACCAGTTATTAAAACTGAAAGAAGAGGGTATTTTACCACCATCTTCCACAGCAAAAATGTTTTCAGCATA
**CoV-bait-4096**	TTGTTAAGAATGTAGACTGAGCCTTGGAGTTCAGTGGTTGGGGCAATGTCGTCTGAAAGGCACATGTATACTTGGGCACATTTATACCAAAGGGTAGCGTGGTGGTTTTGCACTGCGAGG
**CoV-bait-4097**	ACTGATCTTCAGCATCTTTTACTTTCACCAGCGTTTCTGGGTGAGCAAAAACAGGAAGGCAAAATGCCGCAAAAAAGGGAATAAGGGCGACACGGAAATGTTGAATACTCATACTCTTCC
**CoV-bait-4098**	TGCTCTTCATATGAAAGAGTTTCGTAGTAAAGTCTAGCTTTACCAAATTTGTTCAAAGGATAGCCAGCACTCTTGTCATAGTTTGTAACAACAACTTCACGAGCATTAATGCACCCACCG
**CoV-bait-4099**	TTAAACTCAGCTGCTGTTATACCTAATTTAGTTACAAACCAGCTCTCACCTGACAACACAGCTGCATACAACCACGCCAATACGTTGACTGTGATTGTTACATCTGGCGACGCCAGCTGA
**CoV-bait-4100**	AAATAGTGGCAATTACAGTAAGTGCCCAGTAGCCAGTAAAGGTCATACTAGGAAGCCACCAATAAGCAATTGCCAAAGATGTAAGGCTGAGAAAAAGCCAAAAATTTTCCAGACCCCTCC
**CoV-bait-4101**	AACACCGACGTCTGTCAAAGACAACTTACCATCGGTAAATACAACAACTGAACTAACATCAGTGGCAAGAATGTCTGCAACAGAACAGTCATAAAAATTGAATTTACCTTCAACTCTATA
**CoV-bait-4102**	ATCTGAAAAACCATAAAACGACACCCAATCAACATCTATAGCTTTAGTAAGCAAATCAGATGTGTTGAGGTCACTTGGCACTGCACCAGAAAGGTCCACATCCTTGTCCGCAACAACACC
**CoV-bait-4103**	CAAATAAAAATCACTAAAAATTACATTGGCATATATAAAATTAAACCACAACAGAAATATAGTCGCTATAATGCAAGCACCTCTAGCAACCACACTCCACTTAAATGTCAAAAATGCATT
**CoV-bait-4104**	GAGTCAACTTCTTCTTTTTCACTTGATACACTAACTTCCGATTTTTCATCAGCAGTAATATCATACTGAGAGATCATAATACCATCGGGATTTTTTATATCAAATCCACCACAAGTGTCA
**CoV-bait-4105**	CTTGACATATGTTAAAAACTGAATTAGCAAAAGCAGTAGTTGCATCACCACTACTAGTGCCACCAGGCTTAACATAATAACAGCCACCACACATAACAATTTCACTCAAAACTTGTGCGC
**CoV-bait-4106**	ATAACAGCACTCATAAAGCTTACGCTGCAATTGCTTAACTTCTAAATTATGACAGACGTTGCTGTCAACACTAAGAAGTTTGTTAACATTGGCACTTACTGCTTGGAAGATATTAAAAAC
**CoV-bait-4107**	CAAGTCAGGTTTATACTCAATTTTCTTAACATCATCCAAATAATAGGTGGTTAACACCGATTTAAAAGTTTGTTTCAAATTTTTAAGATACAAACAATCTGACAAATTGCCAGTAACGTC

**CoV-bait-4108**	AAATCCGCTCAACAGCTTCAATTCGCGAATATCGGTATGTAGAGTCGGTCAAAATACCTAGTCGACACTTCGCTTATCTCACATGACAAAGCACACCATCAGCGTGCCACAGGGAAATTG
**CoV-bait-4109**	AAGCAAGCTAACACACATCTACTCCAAAACCAAGATGTGGCTTTTCTTACTATAGAAAACTGTAATCTAACACCACCTGCCTGATTAAAAACAGACTCTGGTGTCAATTCATCTTCAAAA
**CoV-bait-4110**	GAACACAAGACAAAGCACTAGTAAACTCCTTAAGTTGGCCATTTTCTATGTACTTGAAATCGTAACCCGCAAAACTACTTACAGTCGTGTTAAACACAACATAACTAGTAGCATAATAAC
**CoV-bait-4111**	TAACAGCTCTCCCGACCAGGGATGCAGCTTGGAAGGTACGGCTAGCAAATTCCTTGAACAAAGCAGAAGCCCTAAAAAGCGCACGGACTTTCTTTGTCAAAGACCCATTAGCTGCAATAA
**CoV-bait-4112**	ATGCCATCCTGAGCAAGACCATGTCCGCTCAATAGAACGCCATTGCCAGGTTGTAGTGGACCATGCTCGCGAATGTAGGCAGTTGATTCAGCCTGCATTGCGTTGTCAGAGGCGCGGTTT
**CoV-bait-4113**	TGCTGATGCGTGTTGGATGTCAATGGCTTGAGCGTTGATTGTTCCCATGAGTGGAAATTGAGATGGGGCGTGATGGTTGCAAAAAGTATGTACAATTTCACGATGAATTGTGTAGAAGGT
**CoV-bait-4114**	TGCCAACCATGTCCTGGCCTAAAAGCACTCTGGTAGTAATACACAAAAGATTCGTTGTCATATAAATTAGCACTACATAGTGCAAACAAAATGGTCACTAGAAACAGTGACTTCACCAAC
**CoV-bait-4115**	GAACACCTGCATTCAAATTTTTCTGTAAATGTTTAACCCAATCAAGAAGGGTGTAAAACACTTTTTGATAAGCAGGCTTAGGATGTTTTGTGAGTGGGTATGCATCAATAGCCAGTGACA
**CoV-bait-4116**	TGTTGACTGGTTCAATCAAGGTCTTTCCGACAGTAACACTTGTCTGTTGTGTAACTTGGGTTTCTGGCATGTAGTTTGTGATGTCTGTTGGTGTGGGTTTACCGGCTTCAATGTGGTTAA
**CoV-bait-4117**	GGTTCGTAGGTCGTGGGCTGACAACACCTCCTGTATGACGAAGATAGCGACTGTCACGTCTTTATCGAGATACGATTCTCCCTCATCCACCCGGCCATAATCCTTACCAAGATATCGATG
**CoV-bait-4118**	GATCTTGGCTGTTACTTGAAGGACTAGTAACAAGGGAACTGGGTTAGTGATGGTTTTGTTTAATGGCAGAGAGAGGGGCCTAGAAGATCGTGGGATTCCTGACTAGCTCGCATTCAATTC
**CoV-bait-4119**	GTCTGTAAGAGCTTGACTAAGACGACCTTCTCTGGCGCCAGCTTGCAATATTTTGGGAGGTGTCGGTCGGCGGTTAGTTCTGATCCTTTCAGCTAGTAATTGCGGGGCCATCTGCTATGG
**CoV-bait-4120**	TTTTGCATTCCCAGTTTTAAATGCATCCACCTGTGAAACAAGAAGCTCAACATTTGGATCAGACTTTGGCACAGTCATTTTATAGTTATATGTAATCTCGTAAGAGTCCGCTAGCTCACG
**CoV-bait-4121**	CCTGCGCCACGGAGACCGAAGGCTTGAACCATGTTGAAACTTTTGGTGGCGACACGCTTATGGCGCATCTTATTTTTAGCAGCAGCAGCATCCTTTTTAGTAATGACTTTAGGCTGTGAC
**CoV-bait-4122**	GTAAAGATTGACTAGAGTAGCTGCAAATTTCTTGAAACGTTTTGTGGCTTTAACCACAACACCATTGAGGTCAAAAGAAACTTTAATTGATTTTACAAATTCAACAGCATCTGTGACAGT
**CoV-bait-4123**	ATCCTGGACCCATTGCAGCAACCATCTGCACACAGCCCTGTATAGGCTTACCACCGTCAGCTAAGTGCTTCAGATAGGCAGCCTTAGGGTCGACAGCGAATGCTAAATAGGTCAACAATG
**CoV-bait-4124**	AAATGCGTTGTCCCACTCTAACTGATCCGGGTCAGCTGAGTTGTCCTTTTCAGGAGCTTTGTCTGCCTCAGCAGCTTTCTTGGTTTTAGGTTTCCTCGGCTGTTTGGGCTTGGAGGATTG
**CoV-bait-4125**	CGTTCAGAGCAACGCCATTATATACAACTGACACCATGCATCTCTCTACAACGCCTGAAGGATGTAGGAGGAGCTTAACGCCAGCTTGTAGTTTTGAAACAACGCCCTGAACTACTGCCA
**CoV-bait-4126**	CACCAGATGGTGTTATCTTTTGTAGTGTGAAATACTTCTCCATACGAGCAGCAACCTCATCATAAGTATTGAGAGGCCTAGAATAAGCCTGATCAATAATAATAATGGTGCGTTTAAGAT
**CoV-bait-4127**	GGTCATACTGGGAAGCCACCAATAGGCAATTGCAAGGGATGCAAGACTCAAGAAAAGCCAAAAATTCTCCAAACCGCGCCATATAGGCGACCAATGCAGAGCTGGAAACATAGTACATAG
**CoV-bait-4128**	TATAGCATCACCCATACAATTATAGGTGGCTTGTTCATTCTGTGTGAGGTGCATGTTTAGCGTGTTGTTGACAATGTCGGTGAAACATGCAAAATATAATGGTTGGTAACTCCTATGCAA
**CoV-bait-4129**	CTGTGTGTTTTATTGTAAGAACTAAAAATACCGCGATAGCAGCTGCAAAAGGCAGGGCTAGCGGGTATAGTGTAACAGGTAATACATGTAGTGCAGCTAACATAATGGCAATAGCCATAA

**CoV-bait-4130**	AATGTTTGAACATGCTGACGTGTACTGGGAAAGCAACCGTAAGCAACGGGGATTGCCATTGCATACATACTGGGCACAATCTATAACAACCTGCTCAGACTGTATCTGAAGATACTCAGA
**CoV-bait-4131**	TTTTTGCATATAAAAACTGCCAAGTGCTTTAGCATTATACTTAACGCTTCTGCGTAATATAATACCAGTGCGTTCAGGCACACCGGCTAACACTTTAACATCACGATTTTTAGCTGCCCT
**CoV-bait-4132**	AGGTGGTAATTGAAAATAGTTGTAACCATTGATGTAGACAGAGCCCCACTTGGTAAAAACAAACTCCCTAAGTATACGCGGCAAAACACCCAGAAAACTGGCTTGTTTAACCGTACTGTT
**CoV-bait-4133**	TGGGTTACAACCCCGGAGTATTTCTCAGAAAGCTAGACTGAAAGACCAGTCACGTCAAGCGCACGTTCGGGAGGGGGTTATGACCGTTCCGGAGGGCGGTTTGACCCGCTTGTAAGTACT
**CoV-bait-4134**	GGTTGATGTCATAGCTACAAGTCTAAGCTTTTTCTTGGGTGTTTTTGGTGTTGTTCCAGCCCATTGTCGTGTTTCGCCAATTGTGCGAGTTTTCAAGCCGATTTTGTTAAAGTCGTAGAT
**CoV-bait-4135**	CCACAGACAACACAGACTTCGTTTTCAATACAAAACCGAATTGGATCTTGTGTGCCAGTTGGTATTTGCACAAACTTACCTTTATAGCGGCACAATCCATCAATAGCAGGATGTTCAACA
**CoV-bait-4136**	ACTTTGGTTGTTGGTGTTGGTGCATTGATGTTTCCACAGAGTGATACTTCTGTTGTGTAGAGTGTTAGGTCAAGACACATGACTGAAGATGCTGAGCTGGTTGGGAGTAGGGTATTGGTG
**CoV-bait-4137**	GTTGTACAGGTTTAAACAAGGCCGCAGAAAAGGCCATAGTTCGTTAAAATCCTGGATCATGTAAAATGGGGTGTGACTGAATAATTTGTTTGGTACCTTCAGCTGCAGATCGTATAGAAA
**CoV-bait-4138**	TAACACCATGTAGGAATGCATACCAAGACAACAGCATAGCATCTGCATGTGATGATGGACCACACATAACTGTTTCATTAGGTTTAAACAATGAAAATTTAAAATTTGCCCAAGTCATAG
**CoV-bait-4139**	AAAGGTTAATGAGTGCGTTAAATCGCAATCTCAGCGTTATGGTTTTTGTGGTGGTGATGGCGAGCACATTTTCTCTCTGGTACAGGCAGCACCTCAGGGCCTGCTGTTTTTACATACAGT
**CoV-bait-4140**	GTTATCCGGACGTTACTCCATGAAATCGCGGAACAGTTTAGAGTTACGAGCGATAAGTCACGTATTCGACACTGGTGGTGCCCGGATCGCCGCTTGCCTGTGTTTCAGAACGTTAGGAAA
**CoV-bait-4141**	CACACTTGCTTAGTGAAATTTGAGAAGGGGCATTCAGCTTCATAGTCCCACAACACAAATTTGTATGTTGCGACAACACCTAGATTTCTAAGTATTGTTAATGGAGGTGTGAGTCCAAGT
**CoV-bait-4142**	CCGGCGAGGTAAGATTACGGCTCGTACATTTTTTGAACAAGTGGATAGTTGTTTACAGAGATTGGTAAAACAAATTTACCTTTAAAGCGGCAAAGACCATCAATTGCAGGATGTTCTACA
**CoV-bait-4143**	CCAGTAGTAATAAATATGACACTCTTAACTTTAGGAAAACCAGTAAAATTCTTAGAATTAACAAAGAAGGTGCCTAATGTTAAGTTAAAAGTAGGGTCGTTTTTTGAATTCAAAACAGTA
**CoV-bait-4144**	GTACTGGGTTGTATATGTGGTTAACTAGTTCAAAGGAAAATTTATTCCTGACTAGTGATATTAAAGCGTGAGTACCACCTATAACAGGTGTATTATCCTCACCATAAATAATGTGCTCAA
**CoV-bait-4145**	ATTACTCCAGTCACAAGTAGCTAGACTATTAAAGTCCGCCACATCCGGACTACCAGTACATATATTTTTGTAAAGACCAAACACCTGACCATTCGCACACAAAGGGAACGATATTGGTGG
**CoV-bait-4146**	GACATAGTCGTCAGGGACTAAAACTGTGTGCAAAAACAGAAGCCCTTCAGGTGCGGCATTGGCTATAGAAAACAAATGTGTTCCATTACCACAAAAACCATACCTGTCAGACTGTGATTT
**CoV-bait-4147**	TCATTGTCAACAATTACTGCGTCGTCAGGTAACCAACGACGGAGTACAGTAGTACCAGGGGCAACACCTTTATCAGAGCCCGCACCAAGATGCAAAACTCTCATATTATGTGGTACACAC
**CoV-bait-4148**	GCTCAGGTGCATGCTTACCCTTACGTGGACCAACAACATTAAAGACCTTAAGACCTAATGCCTCCAACATGACACCACGTCCAACTTTAATGGGACCGTGTGCTTTAATATAATCATTTG
**CoV-bait-4149**	CAGCAGCTGCAGACCACGGTGGGAACATAGCTGCGGCAGTAGCACCCGTCGTGTAACCGGAGATTTGACTCTCAGACAACACAGGAGGTAGCACTTTGATGCCATTAAAAGATTGCACAC
**CoV-bait-4150**	CCATCAGGTAATTTCAAACCAGTGCCATAATTATAGAGGTTACATGGCTCTAGGCACATTCGTTGAATCTTGTAAAGAGAAGGCATTGAGTAACCACACTTCCACTCAGCTGATTGCAAC
**CoV-bait-4151**	ATATGGCTTAGTAACACAGAATGTGTCATTGCCATTAATGTTTATAGTTGCAGATGCCTCTCCAGCTGAATACCTGGCATCAATGGTTAGATTAACCATTTCAAGCTCAGCAACTTTAGG

**CoV-bait-4152**	TTCCAGGACACACACCTTTATCGCTACCAGCACCAAAGTGCATTACACGCATTTTAGCAGGTACAGACAGCGTACACGTGTTAAGGTATTGACAAAGTTGTGTATACTTAGCCACATTCA
**CoV-bait-4153**	GTTCACATACCCACTCAACACATTGCTTAGTTGGGCAGTCATAGAAGTCATCAAAACAATTAACTAAATCCTGTACAAGCTGTTCCTTTGTGGCAAAACTCTTTAAAAGCTGGTAAATGG
**CoV-bait-4154**	ATGTAACACACAACGGTCATCAGTACAATTAACGCAATTTGCGTGATACGTCTGATCCCAATATTTAAAGTACTTCTCAAAGAGTTGTACCTTATAATCAGTAAAATCATACTCAGTAAG
**CoV-bait-4155**	ACTCATTCTTGTCAAAACTAGAATTAGCATAACCAGCAACAACATCATCACAAACAGGGCCACCTATAGGTGTAAAGCGCATTGTCATGAATAGTAATGCATACAATGCATAAATACCCA
**CoV-bait-4156**	ATCAGCACATTGGGTTTGGCTACGGTGATTTCATTAGGAAGTCTATCGTAGTCACATGCACCGAAATGCATACCAGCCACTTTGAGGTAACCGTTGGTTACAACAGCAGTAACACTAGTA
**CoV-bait-4157**	CTAGAAAGACAGTCAAAACTAGGTTCCAACTGAAATTCCAATTGCGTAAATGTTCATAAACTTCTGTGAGAGGTATACTCTCATTTGACATCGTTGTCGACATCGTTTAGTTTAGACATT
**CoV-bait-4158**	ACACAAAAGCCTTACAAACCACCAGTTTAACAAGGCTCAACAGATCATTTAAAAATTTTACAGTGCATAATGTAGCACGCAAACAAACATCAATAATGTTATCTGCAGCTGTTTGAACAG
**CoV-bait-4159**	TGCGCTTTTTAGGTGTCTTTACCTTAGGTCGCACAACTGGTTGTTCAACCTCCACGTCAGCTTCATAGTTGCTAGAAGCCGGAATTTCCGGTTTAGCAGTAGCTGCTGGAGTCACGGCTC
**CoV-bait-4160**	TCAGTTGAAGATTACCGGTACAACGGGTTTACAGTCGGAGTTGCTTTTTCGGATGGCTGGCCAAATGAGTCCCAAGTGCCGCATTGCTCCTGTAGTCATTGAACCATTCATCGATCCACA
**CoV-bait-4161**	GATACGGGTAACCAAGGTGTTGAAAAACCGTATGTTTGACTAATTTGCAGGTTTTTGAGAACTGCTGGGTTAGGTATTGGTTTGTTACTCCGTAGGGAATAGTTGTGGATTGATGTTTCA
**CoV-bait-4162**	CGCTACATTATCCGTTAATTTTAAATAACCCGTAAATTGATTTACCGGTTCTTCCGGCGTCGGTTCCGGTTCCGGTTCTATTTCCGATTGTTCCGTCGTATCATCTAACCAATCTTCTTC
**CoV-bait-4163**	TGTAGAACGTGTAACCATCCAAGTTAACAACTACACCTTCAGCTTTTGGAGGAAGGTATTCACATTCTTCTAAAAAGACATCATTCCGAACAACATCTTCAAAATCTGAAACGGCTACAG
**CoV-bait-4164**	ATTATTAACAACATGGTTACTAACAATTACAAAACTCAAAGTATTAAGAAAAAGTCCACCAATCATTTTCGCATGGAATTTAGAATGGCAGTTTTCCTATAATCTGTCTTTGCTTGTTTT
**CoV-bait-4165**	CTAGTCTGACTAATACCTGCTGTCACAGCCATTCTCTTAACGGCTTGTGGCATCAGCTCATTATTCTGCAGTTTAACGGGAGAGGTAGCAACCTGCCTGACTGCGGTTATAACCAGCGGC
**CoV-bait-4166**	AGTGTTGGCATGTCATCGCACGTCTACCGGCTCACCCATGTTCCTCCAACGTAGCCCTGTAGAGAAACCCTAACGCGACATGGATCTATAGTAAACGCCATTATGGACTAGGTGTGTGCC
**CoV-bait-4167**	TCTGCAGGTTCCAACAGAATGGGGTCTGACCAGTCATAACTCACCACCATACCTTTGTTATTCACAGGTACACTCGCAGTTTTAAAGGAGAATGCTTCACCATATTCAACTACGGAGTAA
**CoV-bait-4168**	GACCGTTCGACCTGATCCCAATAGCGATAAGCTGCATTTACTACGAATGGATTTGCGTGGCGGCCCTCAATCATCGATGAGCGGCCCGGCGATCGGATCGGTGTTATGCCATATGGTCAG
**CoV-bait-4169**	CATTACGGGGGTCGATCCAATCAAGGGTGATCGACACCATAGATCTAAGCTTGTTAGTGCCTAAAGCACCCTCCATCACTGGCGCGGGCGGAACCACACCAGGCTCACTACCGCGGGAAC
**CoV-bait-4170**	AGCCCAAGCCAGAAGTACCTTCAGAGTCATGTAGTGCCTCCCGATAGCGGAGTCTAAATATCCCCCCGGTTGCTGCGTTGAGTGTGTTTGAACGATTATGCAATTACGATCGGAGCCTTC
**CoV-bait-4171**	GGCACATGAGTATGTTAAGCAGACCCTGAATATCAAGACTAAAGAATGACGCTGTATAGTCAAAATACTGCACTAACAGCTGTGTCACAACATAGTCCCAAATAAAATTGTTAGCAGCCA
**CoV-bait-4172**	AACACCCATAGAGGACATACGGGGGGCACCTGATTGATGGTTGAGCTTTTCGTCTGTTGAGATGATTTTCATGAACTTGTCACGCATGTATGTGTAGACTGACTTCAGATATGATGGATC
**CoV-bait-4173**	AATCTTTGGGATTGAGACCTAACGCATTCATTAACTTTGTCTTTAGCAAGTTGTCTACTAGCCCTAACCTCTGCTTGTCTGGTTAAAGTCTGAGACACAAATGCATTAAGTGCTGTAAGT

**CoV-bait-4174**	TATGTGATGACAAAGAGTTGGACTGTTACTGCGAGTGGTGTTGAGACTGTTGTTGCGAGAAGGGTGAGGAGTTGGATGACAAATGACATGATTACGCTGTTGGTGTAAAACTTGAAGATT
**CoV-bait-4175**	ACAATTTCTTTTTCACCACAGCCACACTTAGCTGCAAGTACTATCTTGGCTGTGCTATAATCATTGAGCATAAGTTCAAGAAGATAATCTGCATCACCTGAATGACCTCTTGCTAGTGTT
**CoV-bait-4176**	ACGACCAGAATTAAGATGAGGTTTAGAACTAGTAACATAATCTAGGGTCTTAGGCCCAAATGTACTATAATCAAAACCTCTTGGACTACCAAGATAAACTCTTGTATAAGAGCTAGTGGG
**CoV-bait-4177**	TAGCCTGAATAAACACACGGGCATCCTCATCTAAATTTATATCATGTCGAAGAAGCTGAACTTCTTGCAACACCTCAAAAGTAGAAACAAAGCAATTATCACCACTCAAGAGTAGCTTAT
**CoV-bait-4178**	TTACAACCTTAGAAAAAAGCAAGTCCTCTATAGCTGAACGATACTTACGGTTACTAGTATCGGACGGCAGTATGTATTTTAGACCTTCTAACCAAGAGCCACCTATGTTAGGCCAGTCTT
**CoV-bait-4179**	TGTGTCCTCTATTTAACTAAGATGGGGGCAAGGGGGCCCGTAGCAGCCGCCATAGCGCTAGAGCGTTGTGAGTTGTCCAGCCCTGATTCTTTTCCAATTCGTCGGCGCCTGCCGCGTGCT
**CoV-bait-4180**	CTTATTTGCAATGCAAACAGTAGGTATGGTAGTATGCATACGACAATCATTAAAGGTCAACATGAAGGTTATACCCTTAACTTTAGTCGTACGAATAATGTACTTCCTAGTTTCTTCAGA
**CoV-bait-4181**	AAGACTCGTCACAACAACCTGTCAAGACAAGAATCTCTTTAAGAGTTTCACAATTACGTTCATCAAAGTTACGAAGGGCATAACACAGATCCATCATAGTATACTTTGTAAGATCCTGTC
**CoV-bait-4182**	ATAATATAGTCCAAGACCAATGACGCAATGAGATTTACCACTACCAGGAGGTCCCTGAATGGTTGTAATCATCTGCTTGCCGATCATTTGGTAATAAGGAACAAGATTGGCATAATCATC
**CoV-bait-4183**	TGGACCAATCTTTACAAAATAGCGCATAGTGGTCAATTCTAGACCACCTGCCCATAACACAAAGATTAAATTGTCAGACAAGCCGGCAAGGTAATCAGCAACCATCTGAACAATACGTTT
**CoV-bait-4184**	AGAGGGCTTATGTTATGTGATGTAAGCACAAACAAATCGCCTACAGCCAATTTAGACGTTGAAGTAGCCTTATAATACACCGACTGCTGGGAGTCACTTTTCTCAAAGGTGAATTCACCT
**CoV-bait-4185**	ACCATTCTTCATAGCAGCACTCTTTAATTTAATTATAGTCTTAACAGACAATTTAACAAGTAAATTGTATGGTAAGATAATGTTGTAACTAGTACCTTTTAACACTGAACTTCCAATACC
**CoV-bait-4186**	CCACTCAATGATAAAGCAAAAAGCTGTTCATTTGTATCTGCATCAACCATCCAATGTGTAGGCTTCATATCAATGACCCACTTATTATCTTTTAAAGGTGTATTATGAATAAACCATTCA
**CoV-bait-4187**	ACCCAAAGTGGTCTTAGACACATCACCATAAACCACGTGCTCAAAAGCAAAGTCCTCAAGACCATACTTAGAAATGAAGAAACCCTCATCCATATTGAGAAAGTCTTCTTCCATCTGGCT
**CoV-bait-4188**	CACTCAACAGTTTTATAGCCTATATTTGCCTGTTGTTTAAACACACCTTCTAAATTAAATATAAAAGGATAAGCTGACAATGGCTGTGTTGAAGAATAGGCAAGACCGGCTAAATTGCTT
**CoV-bait-4189**	TCCCCTTGAATTCAAGCTTCAAGTGCTGTAACATAACTAGGGCAGTAGACACAAAACAGTTATTATTTTTCTGTTTTAAATATCTCAAACCACCACTCTCAATAATGGGGTGTTCTCTTG
**CoV-bait-4190**	TTGTATGATGTAATTGATATCACCAATGCGAACCACATCTCCTGTGACAGGGGTTTTTATAGTGGTTTTTGTTGGTGCTAATTTAATGTTCTTTGTACCAATGGCATTACCTTCTACTAC
**CoV-bait-4191**	CACCTAAAAATAGTTTGCAGACATTGGATTCTCCACAACCAGCAACACTACAAACATAAGGATTAATGCTGAGAACATTCTTATGCTCCGTTTCCATAACGTGATCATAACAACATTTGC
**CoV-bait-4192**	CACCTATAAGACTCATCTTAAAGGACTCTTGCATAAAGTTAAAGAACCTCTGAATCCAAGAAGGAGGATTACCACCATTCTTCTTAGGCATCTTCTGGGTCTTGCTAACTCTATCTTCTA
**CoV-bait-4193**	CTTTGCAACATATTGCTGCACTGCTGCTGATAGTGTTGCTCCAGTATTACTCTTAATAACAGACATTTTTACCTCAGCATCACTGATGCCGTAATAATCATCACAAAGATCTAAAAGACG
**CoV-bait-4194**	CTCTGGTGTTCTACCTCTTTCTTTATTATCTCTAACAGCACGTCTAGAAAGTGCCACACTCATCGGTACCTCTAGAAATGCTGTAATATCATACAATGGAAAAAGCTTGGGGTGGAGTGC
**CoV-bait-4195**	TCTTATAGGCATCATAAGCATGGCGTGCAGGTATTACTATAATAGTCTTACCTAAATTGCAACACACCATGCACAACTTAATAACATTTAGCAATGCTATTGAAAACAATATCAATGTAA
**CoV-bait-4196**	TCAAAACCGTTACGGTAGCAGTACTTAATTTCCTGCAGTACGTCACGGAAAGGAAGGCTAGGCACGTTGACTTGATAGTCCTCATTAACGAGGATCTTAAATTGTTTGGAACTCATTCTC

**CoV-bait-4197**	AACCCGTATTATAAGCTATAATACCGGAAACTTTAACGATAGGTGGCTTCTTAACTAAAAGCCAAAATCGCATAAAGAGCTGATAAAATAAACACAAGAGGCCTACACACTTTGAACCAG
**CoV-bait-4198**	GCTAAGTATACGCAACCATTTATAGGTGCACACACTTTACCGAAAAACTGAGCAAAGCGCTCTTCTTCAGTACGGACAAAATCTCTAAAAGCTTCAAATGTACGGGATGCCAAGGCCCCA
**CoV-bait-4199**	CAAACTCTTTTTGAAAATCGTAATCACCCGGTACACTAGGTTCGAGTACTGTGTAATTTACGCTAGTATAATTAACACTACAACTAGTAAGAACTTGAGTATTACTAGCAGATATATTAA
**CoV-bait-4200**	ATGGCAGCCACTGCTTAAGCACAGCTGTACCCGGGCACACGCCTTTGTCACTACCAGCACCAAAGTGTATAACACGCATCTTCGCTGGCGTGGCTAGCGTACACGTGTTCAAATACTGAC
**CoV-bait-4201**	AACCACACTAACTGTTGGCTTAATATTCTGTAAAATAACACTGTTAATATAAATAAATACAGTGTAATCATAACCATGTTCAACTCTATCTAACAGTAGAGAATGAGACTTAAACAAACT
**CoV-bait-4202**	AGTCTGTTTCTGAACTGCAGACCAAGAACCTTTATAACCTGGTACTATAGTAACAATAGCGATACGGTCGTACTCAAATGTCGTACCAAACGCAACGCCAAATGGTTCCTGACAATAAGT
**CoV-bait-4203**	ATCACACGCCTGTTGCGTCACAACAGGCGCATCAGACTCTTGCAACTCCACAGCCGCAGCCCAATCTAGCGTGGCGGTTGATGTATCAGGCGCAGGAGCGGGCTTCACATCTACGGCTAA
**CoV-bait-4204**	CATAAACGAAAACAGTGTAATCATAACCATGCTCTACTCTGTCAAGGAGTAAAAGCTCCTTAGAAAATAAACTATAAATAATAGCTGTATCAGAAACAGCTATAGACCCCTTAACATGGT
**CoV-bait-4205**	AAACTATATTTTGCAATGAACACGTCATCATCTAAATCCATAAAATCTTTCTCCATCTCCGATCGAGGTGTGAAAGAGGATAATAATCTGCTTTGCGTAAAGATAGTGCCACGTGCTAGA
**CoV-bait-4206**	TTTGATGTGTTGTTGGGTGTGATTGTAACTTGTGTTCGTTCACAGAAAGTGGGCACATAACGAAGTATTTTCCACTTGTGCAGTAGAATTCTTCACCTGTTTGAACATCAATGTAACGAT
**CoV-bait-4207**	ATGATTACCAAGCTCGCATATAGTCCGTCATGCGGCCGAGGGCACAGCGCGTTCATCCAGCTGTGCGATGTACAAACACAGGTTTAGATGATGCTACCTGAATTCCTTGGCGGGGGAGCT
**CoV-bait-4208**	TAGCCCGTCTGGTTTCGCAACGAATAAAACAGGTCTACGCCCAGACTAAGCATAATGAACCCCACCAGAGATTCCACGGAACGCAAAAAAAGTAGGTGTACTCGCCGGTTCGTAAGGACT
**CoV-bait-4209**	GTCAGTACCTAGCACGGTCTCCATAACTAAATGCGCCGTGCGCTTACGGCGTACGGCACCTATTAATAAATGCAAGCCGCCTATGGTTCCATCAAATTGACCATACACGATATGTTCTAA
**CoV-bait-4210**	GCATGCGTTCTACAGAGACACCTGTTTTAGCTGCAAGTGGTTGCAAAACTTGCAAGCTCTCACTAGTTACTGACTGACACATGTACTTAACAGCACAATTGTTAAATTCAGCCGGTGAAA
**CoV-bait-4211**	GTATTTAGATACGCTAACTGTTAAATTATAAAATAAAAAACCATTTTTCATTGCAGAAATACGAATATGACCACTTGGAATCATGCCTGTTATAGGACAAGACCCAATACCACTACTATA
**CoV-bait-4212**	CTACGGGCTCCAGTCCCGGCTTACTTCATGATCCACTAGGTCCAGCAAGCACGTTCCCAATTCAACGATACCCTTGGCATGGGAGCTCATAGTTATCAAACAAGCAGTCTGCCCGGTAAA
**CoV-bait-4213**	CCATACCACCCATCAAAGATGCACTATACATGTGAACTTTTTCAGCGTCAGCAACGCCAGGAAGCACCATGATGCCATTGTAGTACTGCGCACAAACGAGGTCTGAAATAGAAAGGCCAT
**CoV-bait-4214**	TCTGATGGAACTGGCGTGTGGTCATAGTGGACAACAACGACACCCCACCAACAGTACGAGCACGCTCCTTACCGCTAATAGCGTATTTAAGGTTAAGCTGTGTCATGGTGGGGAGAATGT
**CoV-bait-4215**	TCACCTGCATCATACGAACAGTCTGCTACTGCGGTGGGAAACCCATTGTCATCATAGGAAACTAACAGTTCTCTACGAGTGACATAGGCTAAATAAAGATTGTTCTGAAACGCCAAACAA
**CoV-bait-4216**	GCCCGTATTCACATGCGTTCGCTCTTGCAGGGAAAAAATCAAATCACGGAAACTTAGATGAAGTATGGCCCGAAAATATATCAACATACTGATAGGCGAAGCCCACACATCCCACGACTT
**CoV-bait-4217**	TACTAGTAACATTAAAATCTGGGCAGGAAAAGGGAAAACCTTTAAAATCGAAACCCTTATACTCAGTAAGCGTCTCATTTAAATTTGCGGTGCAATTCCTGCCAACAAGACTAACATTAA
**CoV-bait-4218**	TGTCCTTATTAGAGCAACAACTAACATGTTTAGCACCTAAAATCATAGCCGAAGCCATGCGTATCATATTGGGCAGGGCACGATCACACTTAGGATAGTCCCAGCCCATAAGACAGGGAT

**CoV-bait-4219**	GACCAGATGAAAAATGTACGGCGAGCTCCCAGATACCATTTGGATAGGAGCCAACTAAATTGGCCGCACCCAAGGCCGGCCTGATCTACCAAGCGTTGGTGGGCATCACTACTGCGTATT
**CoV-bait-4220**	CTTGCAATATTGGACAAGCGAACTGAGCTGTAAAAGACAGTTGCACATTATATGTACTTGTACAGGCGAGCGTTGCCTGAACCTGATGTAGTGATTGAGTGAAGTCCAGCGTTTGATGCA
**CoV-bait-4221**	ACGCATTTTATGTGGAACACACAGTGTAGTAGTGTTGAGGTATTGACACAACTGGGTGTACTTAACAACATTAGTAGTAATGCCATCAGGTAACTTCACTTGTGCACCATAATTATAGAG
**CoV-bait-4222**	CACAACCATTTGCTTTCTCAACAATCTTAATATTATCCAATCCTGTTATAGGTCTCATAAGTACAAGTTTCCGTGGTAAATCAGTGGATGTCAATAGGCATGATGCATCATTTGACTCAA
**CoV-bait-4223**	AGATTCCATACTATACCAAGTTGTTTGAAATGGTAACCTGCAGTAACAACTACTGGTACACCATCAATATGAACTTTACGGACAAGTGGTCCAAAAGCTGTCATTGGTATTGTCATAGAA
**CoV-bait-4224**	ATACAGATGCACCACCATAACTATCTTGATTTGTGTTAGATTCAACACCAACAGTTATAGCTTGACCCGTACCTGCACCGTTAGACAACATCTTGATACAATTGCCAACTGGTTTGACAC
**CoV-bait-4225**	CCTCGTACGTACATCTTAAAACACTGTTTTGACTCAGGCTTTACAGGCTTGAATTTGTTCTCATAAACCAAAGCAGAGACTGTATTAACAATTTCAGCTGGACACCTAAAACACTTGTGT
**CoV-bait-4226**	CGAGCACTTTGTCATTAACCACAGCTACAGGAAGCTCACCTTCAACACCAACAAAGAATCCTTTCTTTACGACATTAAAAGCAATGTTCTCAAGGCCTTGCAAATTGTTACTAAATGTCT
**CoV-bait-4227**	GTAACAGCACGGCGGTGGCCGGATGTCACAGACCCCTTGTTACCACCCTTACGAAGAAGGATGGACCAGGGCTGCAACCACTGCTTCGCATACTCCGGTATGGCTGTGACTGGAACAAAC
**CoV-bait-4228**	TATAAGCTTTTTACGCCCGCATGAGTCTCATAGGATGAAGCTATAATTCCGGTAACGCGAAAGTTATATAAAACGTCTTTGGCACGCTGGCGATTCGTAAATTAAGACTTGCAAGCTAGA
**CoV-bait-4229**	CACCTAAGATGGCAGATGCCGAAACTGTCATATTGGATTCTCGAGTGATAGACGTATAACGGAGGCCTTACCAAAACCTAAATTTGACGCTCCGTCTGATCTCTTCACGCGGTCTCTCCC
**CoV-bait-4230**	CCTTGATTCTTCACCATATCGGCAGTTTCTTTAATAAAAAACTTGCCCGCCGCCTTAGCTATAGCACCTGCAACACCTGCACCATGAGCCATCCGCCCATTAGCAGGGTTAACAATGACT
**CoV-bait-4231**	ACATGAATAGACTATGCTGGGCTTCGCACAAGGGCAATATGAAGCTCCAACAGTGAAACATGCCTCTGCATAAGCCACGTCATGTATACCACTACCAAATGTAGCCACGTCATTAAATCC
**CoV-bait-4232**	ATACTTGTATAATACAGGCATAACATAGCCAGGTTTCCAATCAGCAGCAGCCTGCAAACGAGGATAGAAAGTCATAACCTTCTCCTCATTACACCACAACATAAACTGGAAATCTTTAAA
**CoV-bait-4233**	GACACACGCCTATCTACAACGTGTTGAACAACATTTATAGCATCATAGCTGTCCAGCATATCAAAACCTGAGAAGCATAGCTCACATACCATACTTCCATTACAAAAAGAACTTCTATAG
**CoV-bait-4234**	CTATAGTCACCTCTATTTACAAGCAATTGTCTATGATAGACATCGTCCAATCCAAGAGTAGCTCTCTGATCGAGATTGCCACACTTGTGCTGAACGTAGTCCATATATAGAGACCGGCAT
**CoV-bait-4235**	CTTGAACAAACTTATACTTAGGAGTACTAGCATTAACAGCTGCTGTCTGAAGTATCAGGATAGCACCCTGTAATTTCCTACTAACAACCTGCAAAGATGCACCATCAGGGCCAATAACCT
**CoV-bait-4236**	TTGAAATTTTATACCACTTGTAATGGCAGCTACACTAAAACAACAAGTTCGTAAATCAAGCAGAGCACTAGCAGATGCAACATGCATAGCAGGATCTGCTGCATAAAGAAGTAAATCTTT
**CoV-bait-4237**	ACCCCACTAGGGTTCCATCCCGAATCGTAACACCAGTAGCACATGAAAGGACGAGCTTGACGCCTCACCAACCCTTACGTGAGGGCCATTTTATCGCTCACCTGTTAATCAACCATGCTT
**CoV-bait-4238**	TTTCTGGCCTCGAGGACACGTGAGGGACGGCCTACGAGATGCATTGTAAGACCTTTTGATACAGGAGTGTTATTTACCCACAATCGAAAACGTTTTTTAAGAAATTCTGTAGCGCATTGT
**CoV-bait-4239**	AACTTAATGGCACTAAGCCCTTTAACAACGTTATTAGAAATAAGCACTGCATCTCTTGTAGTAGTAAAACGCTCAAAAGAACCAGCAATACTATTATCAAAACATGTTACAACTTCACTA
**CoV-bait-4240**	TATAAAGCTGCTTACTCCTATCATACAAATAAATAGAAGGGGACAGCACCAAAGTATTACATAAACCGCAAAGTTGAATACAAAGTTTGATAGACGCAAGGAAGGCAACCACAATTATGG
**CoV-bait-4241**	CATTATTACTATCCCATTTAATACAAGTTAAACCATCTTTTTCTGCTATTAAAGCATAGACAAATCCTTTACCTTGTTCATTCGTATAAAGTGCTTTGCCATCAATGGAAAAACCTGTAC

**CoV-bait-4242**	GATGTTAAAATAATGCACCTAACAATGTATAGCAGGAGAACCCTAGAAGAACTAGAAAGATCACTCGCAGTACCACTACTAGTAATCTGGTTTCTGCTAAAACTTTGTGTAAGATGTTAA
**CoV-bait-4243**	TAACAATATACACGCTCACCGCCCAAACCATTAAGTGTGGTACAGGCAGAGTCGAATATACAGCGGTCCAAAGGAGCTGGTCCAGTCATGTCATAGCATGTGTGTGAACCTCCAAAAACT
**CoV-bait-4244**	CGGATTCTCGACATCAGCGCACAAGGTGCGTAGCATACGATTCCATCCACCATAAAACTTAGTTGTGCCTATAACTACAGATGCACCACGAGCAGCTGCAATAGACTTAAGCATCTTCTG
**CoV-bait-4245**	TCATTAATACCGTCACGTACAAACACCTTATCGCCCATAATAGCAACAGGTAGCTCACCCTTAACACCGACAAAAGAACCCTTTCTAACAACATTATAAGCAATGTTTTCTAGATTCTGC
**CoV-bait-4246**	TAGTTTCAAAACCTGAAAGTGTACTATCAAATCTAATCGCGCCACTATAGCGCAATTCATAAACATCCTTCTGGGGTTCATCAGCATTCCCAGACAAATTCTGCACTTTGGCCAACTCTA
**CoV-bait-4247**	CCTTACGCTTACTAAGACAGATTGCTTTCGTGGGAACAGTTAATAATAGCTCATTTGAAATGGCTAAATAATTACCAGAGGGTAAAACTAAATAATGACAATTAAAATCAAAACCAGTGG
**CoV-bait-4248**	AAAGTCTTACGTTCCCAATTTAATGGAGAGGGCACCGACTTATCATTAAGCCAAGCCTCTATATTACAATCGGGAAGATTAGGTATACGTCGGTAAACATCTGCAATTGGCTGAACAGTG
**CoV-bait-4249**	ACTCTGAATTTGCCAGAAGCTTCAAGCAAACACTTGAAGGTTTCAGCTGTCAGTCCTTTACAGTTCTTAATGAACTGGTCCACTGAACCTGAAACTGCTGCTACAAGAGCAACCACAGTC
**CoV-bait-4250**	TGTCACATGAGAGCGGTAAATACAACCCTGCCAACTGGTTAGCTACAATAACAGCCTGAGTAGATACTGTAGAACACGGTTTGACAGTATACAAAGAACCATTATTACGAAAGGCCAATA
**CoV-bait-4251**	ATTGTCTAAAGCTAAACCACGGTCATCCAAAGTAAGCATAGAATCAGTAAACAGAACAATAGGTTTGCCAACAGCGACACACAACAAGTCGTCTGTAAAGTAACTAAATTTACCATCTAC
**CoV-bait-4252**	CTTTCTGACTGATTAATAGCGTGATAGGTAGAAGAACGGAATAGGCCGCCGATGCATCGGGTTTGTCTCCGCCCACCATTAGCGCGTGATTATGTCGCGCATGAAACCGAGTCACGCGTT
**CoV-bait-4253**	CAGGCCTCCTAGGACGCACACATCCAATGAGAGCACTGTTCATGACATAAGCGTAACGACAGCTGCTTGACTCTTAGAGGCCATACCGCCCTAGCTTAATTCGTCCGATAATGATTCTAT
**CoV-bait-4254**	GACAAAAGATGCCGGTGCCAACAATACTTTTAAAAGTGTGCATCAAAACTTGAGCACACTGAGCACAAGCACCATATGGGAACGGTTCCAAAGTTGGCGTCATACGAAAAGCACAACCCT
**CoV-bait-4255**	ACCGCCATTAGGGCGCGTTGAAAAACATAATGAGCCAAAACTTAAATAATTATTTAAGTTTTGGTAGCTAAAAGGACAATCACCAGCTTGAATGGAAACAGCAGTGCTGAAAGCTGAAAA
**CoV-bait-4256**	TCTGCAATGTTGGTTGATCAACATAACCCCCATACATCACACCATCTAAATCACATCCAACATGGCAACCAGAACCTAATTCCAATTGATGTAAATAGTTAAACTCCACAACACCATTAA
**CoV-bait-4257**	ATGGTCACAACAAGGGTAAAAGGCTGGAGTAGCCAAAGTAATATTAATTTGATAATGTAAATTAAGCGGGCTCTAGTAGCATAGCCACCTTGGAGAATAACTCCAAGAGCCCAAATAATA
**CoV-bait-4258**	AACTACATTCTGTGCTGATATACAACCACCCTCATAAACTGAGAGGTAGCAATTAGCTACTTCAACACAAAATAAAAACATTGATATGTCCACCATGGTTGGAGTATTGTACCTATAGTA
**CoV-bait-4259**	TTAGCTGTACTATACTCACTCGGCTCGGATAGCTAGGGAGCTAGACGCCTGTTTTGTAGCTTCTTGGTGCGCCTTGAAAGCCCGCACTAGGGAGTGATTTGAACGTCAGTCGTCAGAGAA
**CoV-bait-4260**	AACAGTTACTAGCAATGGCTGCGGGGCTGATCTGGGAGCATGTAAAGTCATTCACGCTGAACAGTGACAGCAGCTTGGTCAGATTGTAGTTACAGTTAGTAAAGACCAGCCGCTTGAAGT
**CoV-bait-4261**	CAAAAGAGCTGAGTCAACAAGCTTAATAGGCTTACATAGAAGCTGAGAGAAATAAACACAAGCATTCTTAACATTAGCGACGTTCGTACCACTATTGTTAAAAACAATAAAGTCAGCACA

**CoV-bait-4262**	GTCTGCATCCTTATCAGCCACCCAAATGACATCATCATTCTGCTGTCCCCACTTCAAATCTGCAAATGGTCCAGTACCAGTATAATAAAAGTAGTACGCATCAGTAACTGGAGTTCTTTT
**CoV-bait-4263**	TGTGCAGTATCAGACGTCTGTGCATAGACGACATAATCATACTCACTGCCTTGTGAAGAATCAACAGTTTGGGTTTGAATACCCAAAAATCGTGCAGCTACATAATTTTGAGAGTTGTAT
**CoV-bait-4264**	GTCCATTACCGGAGCCATTGGTAAGCATCTTAACACAGTTACCCACTGGCTTAGCACCACTGCGAACAGCATCAACATACGTCTTAGCTGGATCGACAGAGAATGCACAAGCTGTTAGTA
**CoV-bait-4265**	TTATTTCCTGCTCTACAACAACAACTTGTTCTGTCGCTGCATCGTCAGCATCAGTAGATCTCTCAACTGACTCTTTAACAGTTTCAGGCGCTTGTTCACACAATTTTTCTTGTGCATCAA
**CoV-bait-4266**	CACACGTTGAGTGACATGAATGAAACATCAACCAGCCCCTCTAATGGCTTTGGCGTTCCAGTGACAAAGTCACCATCAGTAGTGGTGAAGTACAGAGTTGCAGCTAAATAATACTTGTAA
**CoV-bait-4267**	GGAAAAGTTATATGAGCCGACATTAGCTTGAGGTGTTTCACTCCACATCACACCGGCAGTAAGCTCTGAAACCATAGTCACCATAGATGAATCAATAATAGCACTGACGTCGGCAAGAGC
**CoV-bait-4268**	GTTCATGATCCCAAACGACACAATCTTTAGTACAAGTAACACCCAAATTGCGTAACAATTTACATTCTGGCATCACTTTAATACTACGCTTCGCCCAAAGTTCAAATGCTACATTAGTAG
**CoV-bait-4269**	TCAACTTAATTAAGGTAATAGAGATAAGCAAGAGGAATAAAATCACAATTAGCCATAAAAGGGTACTAAGTACCATTCCATGATCATTGACTAATGTCAACATTGTGAAGTGTAATAAAG
**CoV-bait-4270**	CGTGTGGAATGCATGATTATTAACATACAAGGAACCACCATTACAGCCTTCAAGATTCAAAGAAGACTTGCAGCGTGTATCAAAACGACATACAATGCTAAACTCAGGATACATGTCTAC
**CoV-bait-4271**	AGAACAGCATGGAAGAACATCAGGCCCTGTGGGGCGGCTTGCGTCACAGAAAAGATGTGATCACCATCGCCTCCGCAAAATCCATATCTGGTAGATTGCGATTTGACGCATTCATTAATC
**CoV-bait-4272**	CGACGTTATTGCACAACCGGTGGCACGCAAAACAGAGGTTTATAAGTTGGACGAAAGTAATGCTTATAATAAGCAAGAAAAAGAGTACAAAAAGCCAGAGAATAACATTGACTACAAGAC
**CoV-bait-4273**	CAATCTCGCCCATTGGTGTACATATTAACGGTACTACCAAGCCCATACTGACGCGGCCACTTACAGACACGCAAATATGCGATCTGCTCAGTGCCTGTTTCACCACCAAGATTCTTATAA
**CoV-bait-4274**	TGTTGAGATTATCAATCAACTGTTGTAAAATAATGGTTTTATTGTGGAGACTTTCAGCTTTTTCATACAGCTTGCTAATGTCTTCGGTCAAGTTCAAGACAGTCTGGTTGTAAAGATCTA
**CoV-bait-4275**	TGCCTAGGTTCTAAAGAACCCTTGCTCAACAGCACACGTGGTGCAGGTAATTGAGCAGGATCTCCAATATATACATAATGTTTAGCTTTAATACGAGCATTTATAACAGACAATTCATAA
**CoV-bait-4276**	TATCCCCGTCTTCGCGGGCGTTCTGGACTCTATGATTGCCAACCTTGTACTTAATGTACACAATGATGGCAGATTCTTTGCCTATGGCTCTAGTTAACGCCTTCTTGTACCAGTGTAAGG
**CoV-bait-4277**	AAGACCACAAGCCGCAAGGACCTGCGTATTAGAAACTGAGATGTTTACGGGATCGTAAAAGCTCATTTTAGTGAATAGCCACCTGTCTGTGTTGTTAGGCAACACAAACAAACCTCCTAG
**CoV-bait-4278**	CCGCCACAGACATACATAGCACAATCAATGCTCACAGGTGTGTTAAAAAGCTGCACGTACTCAGCACGGAGCCCCATGGTGAAATTTGTAGGAATACTGACATTACCAACTATCATAGGC
**CoV-bait-4279**	CGCATGCAGTGTCAGAATAGTAGAAAAATGGCAAAGGTTTCAAATCAACAAAAGCCATTTTATCAAAAGCTTCCGTATGGAATGCATGTTGGTTCACATATAGTGAGCCACCATTGCACC
**CoV-bait-4280**	CACCAGCAACAATGATGCCATCTAAAATAGCACCACAATAAAATAAAACTGGTGTGTAAATGAGTAGAAGCAAGACGCGCGCAGCTAGCAAAATAGCGTTGCGCCTAAGATTTGAAGCTT
**CoV-bait-4281**	TCAATGTAAGAGTTGAAAAACACCAAAATTGGTGTGTACAACAGCAAAGTGCAAACGCGCAAGGCAAAAAGTGCACAATTTGTTCTGAAACAAGAGGCTTTGTACAATGCAAACCAAGTC

**CoV-bait-4282**	CCAGCAGCAAAATAGTGGTGCTAAACAGACTTACAATACTAGCATTATCGGACAGCGCTGTTGGTCCCTCTACATGGATGCCGTTACAGAAGACACCACGGGAGGTAGTTAACACAACAA
**CoV-bait-4283**	GAGCGGGTTGCTCGGATGGCTTAGGAGTCACCAGCAAGTCACGGAACAAATTATAGAGATTCATGCCGTTCGTTTTCCATGCACACGAACAGCAGGATACACATCCTCATGCTCCTCATA
**CoV-bait-4284**	CATGGTAGTGCTATTAAGATACTGGCAAAGTTGAGTGTATTTTATAGTGTTGAACATAACACCATCAGGTAATTTAAGTCCTGCACCATAATTGTACAAGTTACATGGTTCCATACACAT
**CoV-bait-4285**	AATGGTAAGGGGCGGAGTCAAACCAAGCTTACGTTTGGCGTACAACTCAAAAGCAACGTTTGTAGGAAGTGTGGTTTTATTTGTAAAAACCAGAGTGTCTGTTAAACCGTCTCTTACCAT
**CoV-bait-4286**	ACAAAGTTCTCTACGCGATTAAGCCACTCAAGGTCTACAAGTGTAGAATTGATGTTATTAATGAGGTGGCGTAGATCCTCAGTCACATTGTGAAGAGCATCAGCACGCACTTCAAGGTCA
**CoV-bait-4287**	CCATCATCGAGCTCAAAGGACAACTGACTGCATTTCAAAGAACGCACGGGATTGTCACAATAGAGCATTTTCACAATGTTCGTGCCATTAACCTGGGTCATGACCTCCACTAGCTTGGTG
**CoV-bait-4288**	ACATCGGTTTGTAAAGCAACATAATTTAGGCGCGCCTGCACGGAATAGCTGAAGGGCAAGGCAGCAGCAGCTGTCAAACCTCCCATGACCATGCCCCCTGTGAGTGCAGCAGAATAAGTG
**CoV-bait-4289**	ACCATAAACAACATGTTCAAAATTAAAATCCTCCAAGCCATATTTTGAGATAAAGACACTAGTGTCCATGGACAAAAAGTCCTTCTCCATTTGGCTACGAGGTAAGAAATTTGCTGAGTT
**CoV-bait-4290**	ACAGCATTCATACTAGCACCATTGCAAGACATGTGTGAATTGTTGCCACTAAACTCAAAGTTGACAGCCTGTCCATTATAGACAGGAACTGACCAAAGGCAACTAACGAGCATGGGCTGG
**CoV-bait-4291**	GTACTTAACAAGCAGCTCTTTAGGCTGTACAGGGGCTATGTACAAATTATTATGTAGGAAAGCGCATGCACTATCACGTGCACGAGCGACAGACTGAAAGAATTGAGCAAACGGTAAATT
**CoV-bait-4292**	CAACATCTGGAGAACCAGTAGCTGAATTCTTATACAAGGCAAAGACATTACCGCCAGAACACAATGGGAAAGACAATGTTGGTTTGTGATCAGCACAGAAATAACTCAAACCGCCAAGAA
**CoV-bait-4293**	GACGGACAGTCTGAAATGTTAGACCTGTCGTGAGTGCGGCAACACTAAAACATGGTGTTCTTAAATCCCAGAGGGCGGAGGCTGAAGCTATGTGCATGGCTGGGTCAGCTGCATACATCA
**CoV-bait-4294**	TACAGCCACTGCATATGGAGTAGTACTATTAAGATGATAAGCATGCACGCAACCATAAAAGTTGCTAGGCAAGGCATAATTGTAAATACTAACAGCATCCGGCACAGATCCAAGCATGTT
**CoV-bait-4295**	CCCACAGCGGTTTACATCTAGTTGGGTAACCCCACTGGAGCAAAGCTGTGATAAATAGTAAGACGACTGAAATTGCAAAGTTCCAGTCGCGCAGAGCCGCCATAACCTCTGAACGAGGTA
**CoV-bait-4296**	GCTGGGAACCTGGGGTCATCAGTGCCTAGTTTAAGCATTTCTAGGTCACCAAAGTTGCCTTCTAGGTCACCAGGTCCTCTACGGCCAAAATTCTGAGTAACGCCACTACCTTTATGAGGT
**CoV-bait-4297**	CTCAGAACCGCGAGCAGTAGTGCTAGCGGCTGCGGTCCAATATTCGGAAGCTGGCATGCTTACAGCATTCGCGCGCGCACTACAGTCGAGACCGTTGAACACAAGCGCACGCTGATGTCC
**CoV-bait-4298**	AACTAAAGTACTCAATTGCTGCGCATGCTGGTTGATGACACTCTGCATTTTGGCTAAAGCCATGTTGGTAGCGTCAAAACCCTTCTGTATGCCTGTGAGGGCATTGTTGAATGCGTTGGC
**CoV-bait-4299**	CGCCCAACCCACAAAATGTCTGGTTGACGGGTGCCAAACCGAAGATCGGCATAGCGACCAGTCCCAGTGTAGTAAAAATACCAGCGCGGGTTAGCAGTCAACGACTTGCCACCCTTGGTA
**CoV-bait-4300**	AAGCACGAACTTCTGTGTACTTAGTCAAAGTCTGAGTCACAAAAGCATTAAGTGCAGCAAGCCTACCTGTAATAAGCCTATCAACTTGTGCATCTGCCTCTAAACCGTTCAATCTATTAT

**CoV-bait-4301**	GCCAAACACAACGCCAAATGGCTCTTGACATGTCCTATTAGGGTTATCCAAGTGTGCAAACCGATTCATGTAGGTATGCACAAGGAGCCCGTCAAAGACTGGTAGAAAAGACGTGTTGTA
**CoV-bait-4302**	ACTCAATATTACGACGGACAAGACCGATTAATAGATGCAAACCACCTATAATACCATTGAATTGCCCGTAAACAATATGCTCCATACCCAAGTTACTCAAATCGTACTTAGCTATGAAGT
**CoV-bait-4303**	ACAATCTTAACTTCCTCAAATATATAACCAACACATCCTTATCCAAAATACATAATTATATACTCATCAACTAACACTTTCTTCAAAACTCTTAAAAACATCTCAATAATACCACCAACC

Supplementary table 2: Primers for detection of coronavirus, Japanese Encephalitis virus, Rabies-related lyssavirus, Leptospirosis and Hantavirus.

Target pathogen	Forward primer (5’-3’)	Reverse primer (5’-3’)
Coronavirus	GGGTTGGGACTATCCTAAGTGTGA-	CCATCATCAGATAGAATCATCATA
Japanese Encephalitis	AGAGCGGGGAAAAAGGTCAT	TTTCACGGTCTTTCTACAGT
Rabies-related lyssavirus	ATGTAACACCYCTACAATT	CAATTCGCACACATTTTGTG
Leptospirosis	GGCGGCGCGTCTTAAACATG	TTCCCCCCATTGAGCAAGATT
Hantavirus	TYTTTGARTTTGCHCAYCAYTCWGATGATGC	TCATGNARRTTRAACATRCTYTTCCACA
